# Enantioselective Rh(I)-Catalyzed C–H Arylation of Ferroceneformaldehydes

**DOI:** 10.1021/acscentsci.3c00748

**Published:** 2023-09-28

**Authors:** Chen-Xu Liu, Fangnuo Zhao, Qing Gu, Shu-Li You

**Affiliations:** New Cornerstone Science Laboratory, State Key Laboratory of Organometallic Chemistry, Shanghai Institute of Organic Chemistry, University of Chinese Academy of Sciences, Chinese Academy of Sciences, 345 Lingling Lu, Shanghai 200032, People’s Republic of China

## Abstract

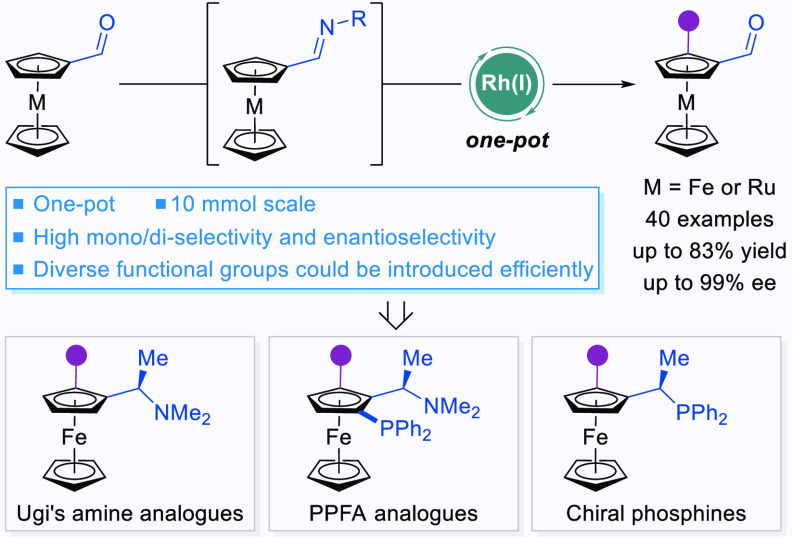

As an important class of platform molecules, planar chiral ferrocene carbonyl compounds could be transformed into various functional groups offering facile synthesis of chiral ligands and catalysts. However, developing efficient and straightforward methods for accessing enantiopure planar chiral ferrocene carbonyl compounds, especially ferroceneformaldehydes, remains highly challenging. Herein, we report a rhodium(I)/phosphoramidite-catalyzed enantioselective C–H bond arylation of ferroceneformaldehydes. Readily available aryl halides such as aryl iodides, aryl bromides, and even aryl chlorides are suitable coupling partners in this transformation, leading to a series of planar chiral ferroceneformaldehydes in good yields and excellent enantioselectivity (up to 83% yield and >99% ee). The aldehyde group could be transformed into diverse functional groups smoothly, and enantiopure Ugi’s amine and PPFA analogues could be synthesized efficiently. The latter was found to be a highly efficient ligand in Pd-catalyzed asymmetric allylic alkylation reactions. Mechanistic experiments supported the formation of imine intermediates as the key step during the reaction.

## Abstract 2

Various planar chiral ferrocene carbonyl compounds, as an essential class of platform molecules, have been synthesized efficiently via Rh(I)-catalyzed asymmetric C−H arylation.

## I Introduction

Over the past decade, transition-metal-catalyzed enantioselective C–H functionalization reactions have contributed tremendously to the improvement of molecular complexity from readily available chemical feedstocks.^1−9^ Notably, Rh(I)-catalyzed asymmetric C–H functionalization reactions have progressed rapidly.^10−13^ In 2004, Bergman, Ellman, and co-workers made a breakthrough in Rh(I)-catalyzed intramolecular asymmetric C–H alkylation reaction using an imine as a directing group.^14^ Later, several elegant works for Rh(I)-catalyzed asymmetric C–H functionalization reactions have been demonstrated.^15−26^ In 2016, Glorius and co-workers achieved remarkable progress in the combination of a rhodium(I) precatalyst with a chiral N-heterocyclic carbene (NHC) or monodentate phosphonite ligand enabled asymmetric C(sp^3^)–H arylation in good yields and enantioselectivity.^27,28^ Our group recently explicated the mechanism of Rh(I)-catalyzed asymmetric C–H arylation, which first occurs by a directed C–H activation through a concerted metalation–deprotonation (CMD) pathway and the reductive elimination is the turnover-limiting step.^29^ In these examples, a variety of strong coordination directing groups such as pyridine, thione, thioamide, etc. have been employed to warrant high efficiency and stereoselectivity for Rh(I)-catalyzed asymmetric C–H functionalization reactions; however, these directing groups in general are difficult to remove or undergo subsequent conversions. To date, there are rare examples of rhodium-catalyzed intermolecular asymmetric *ortho*-C–H activation of aldehyde derivatives, likely due to the weak coordination ability of the carbonyl group and the potential competitive aldehyde C–H cleavage by rhodium catalysts.^11^

**Figure 1:**
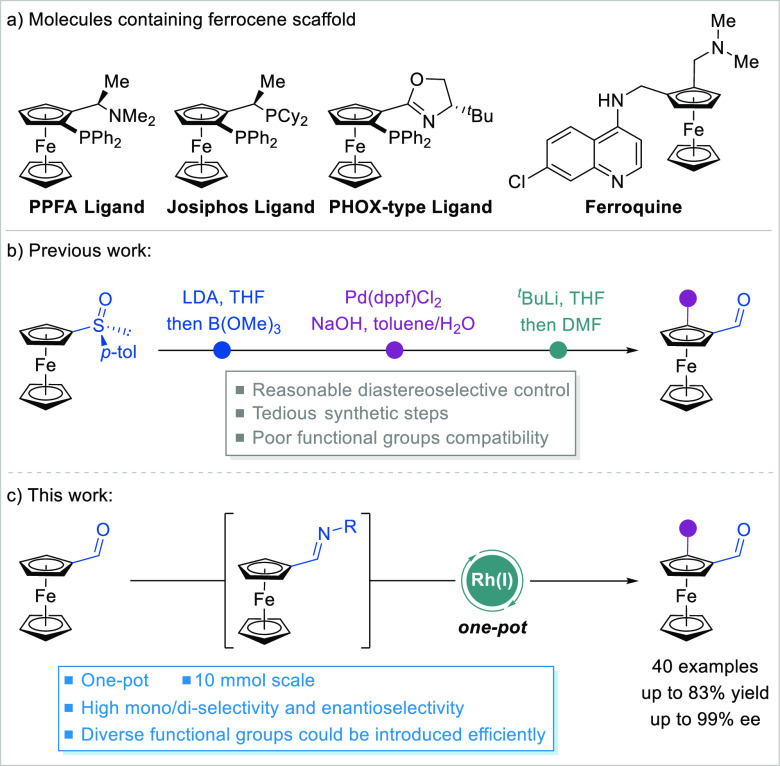
Enantioselective synthesis of planar chiral ferrocenes. (a) Molecules containing a ferrocene scaffold. (b) Previous synthesis of planar chiral ferroceneformaldehydes. (c) Asymmetric C–H arylation of ferroceneformaldehydes.

Ferrocene derivatives have received extensive attention in materials science, medicinal chemistry, and asymmetric catalysis because of their unique electronic and structural properties (Figure 1a). Planar chiral ferrocene carbonyl compounds are an essential class of platform molecules that can be transformed and applied in the synthesis of chiral ligands and catalysts, such as Ugi’s amine, PPFA, Josiphos, PHOX-type ligands, and so on.^30−37^ Traditional methods for the synthesis of planar chiral ferrocene carbonyl compounds usually relied on preinstalled chiral auxiliaries, and tedious synthetic steps were needed (Figure 1b).^38^ The utilization of various reactive metal reagents also resulted in poor functional group compatibility. Meanwhile, transition-metal-catalyzed enantioselective C–H functionalization is emerging as a powerful tool for synthesizing planar chiral ferrocenes.^39−42^ However, directing groups often need to be preloaded and are difficult to remove.^43−57^ Therefore, the development of an enantioselective and efficient synthesis of structurally diverse planar chiral ferroceneformaldehydes is highly desirable.

Inspired by these above pioneering results, we recently found that Rh(I)-catalyzed asymmetric C–H arylation of ferroceneformaldehydes was realized by a strategy of the *in situ* formation of imines (Figure 1c). In the presence of a Rh(I) catalyst derived from chiral phosphoramidite, direct arylation of ferroceneformaldehydes with readily available aryl halides proceeded in excellent enantioselectivity. Herein, we report the results of this study.

## II Results and Discussion

### II.1 Reaction Development

Imines are known as efficient directing groups for Rh(I)-catalyzed C–H functionalization reactions.^58−60^ To overcome the weak coordinating ability of aldehyde, *in situ* generated imine from ferroceneformaldehyde **1a** and benzylamine was examined in the presence of 5 mol % [Rh(C_2_H_4_)_2_Cl]_2_, 20 mol % **L1**, and 2.0 equiv of LiO^*t*^Bu. To our delight, the arylation product **3aa** was obtained in 66% NMR yield and 99% ee (Table 1, entry 1). Other aryl-substituted (TADDOL)-derived phosphoramidite ligands such as 3,5-Me_2_-C_6_H_3_ and 2-naphthyl reduced the yield of the reaction (entries 2 and 3, 22–24% NMR yields, 98% ee). Meanwhile, the substituents attached to the nitrogen atom in the ligand were also investigated, but the yield and enantioselectivity decreased when more bulky substituents were introduced (entries 4 and 5, 21% NMR yield, 50–71% ee). Chiral phosphonite ligands **L6** and **L7** also enabled the reaction to proceed smoothly (entries 6 and 7, 53–60% NMR yields, 99% ee). The utilization of diastereoisomers of the Feringa ligand (**L8** or **L9**) resulted in a marked decrease in both yield and enantioselectivity (entries 8 and 9, 12–24% NMR yields, 42–73% ee). Unfortunately, SPINOL- or BINOL-derived chiral phosphoramidite ligands **L10** or **L11** failed to give **3aa** (entries 10 and 11). In addition, NaO^*t*^Bu as the base also led to excellent enantioselectivity but a moderate yield (entry 12). Then, screening a variety of solvents disclosed that dioxane could provide better results, leading to **3aa** in 80% NMR yield and >99% ee (entry 13). When (*R*)-phenylethylamine was used instead of benzylamine, together with either configuration of **L1** or PPh_3_ as ligand, the target product was not formed, likely due to the steric hindrance of the chiral imine. Overall, the optimized reaction conditions were obtained as the following: [Rh(C_2_H_4_)_2_Cl]_2_ (5 mol %), (*R*, *R*)-**L1** (20 mol %), and LiO^*t*^Bu (2.0 equiv) in dioxane at 80 °C.

**Table 1:**
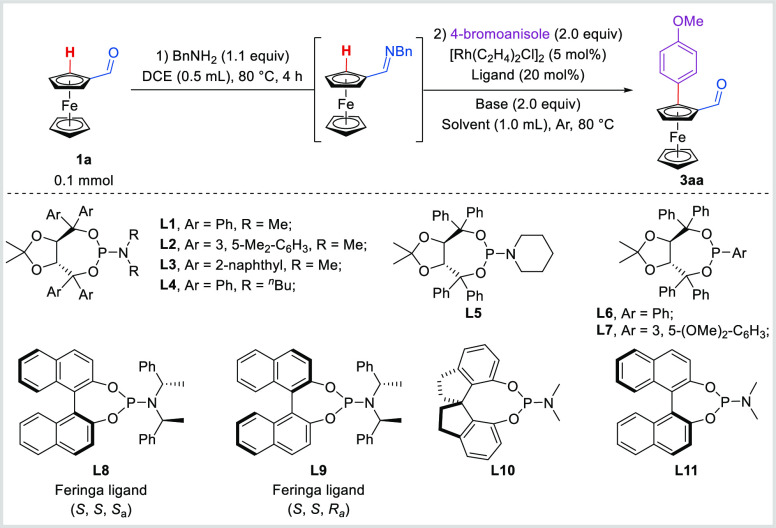
Optimization of the Reaction Conditions^a^

entry	ligand	base	solvent	NMR yield (%)^b^	ee (%)^c^
1	**L1**	LiO^t^Bu	THF	66	99
2	**L2**	LiO^t^Bu	THF	24	98
3	**L3**	LiO^t^Bu	THF	22	98
4	**L4**	LiO^t^Bu	THF	21	71
5	**L5**	LiO^t^Bu	THF	21	50
6	**L6**	LiO^t^Bu	THF	60	99
7	**L7**	LiO^t^Bu	THF	53	99
8	**L8**	LiO^t^Bu	THF	12	73
9	**L9**	LiO^t^Bu	THF	24	–42
10	**L10**	LiO^t^Bu	THF	<5	
11	**L11**	LiO^t^Bu	THF	<5	
12	**L1**	NaO^t^Bu	THF	20	98
13	**L1**	LiO^t^Bu	dioxane	80 (76)^d^	>99
14	**L1**	LiO^t^Bu	toluene	20	99
15	**L1**	LiO^t^Bu	DCE	8	
16	**L1**	LiO^t^Bu	MeCN	<5	
17	**L1**	LiO^t^Bu	MeOH	<5	

a Reaction conditions: **1a** (0.1 mmol), BnNH_2_ (0.11 mmol) in DCE (0.5 mL) at 80 °C for 4 h, then **2a** (0.2 mmol), [Rh(C_2_H_4_)_2_Cl]_2_ (0.005 mmol), ligand (0.02 mmol), and base (0.2 mmol) in solvent (1.0 mL) at 80 °C.

b Determined using 1,3,5-trimethoxybenzene as an internal standard.

c Determined by HPLC analysis with a chiral stationary phase.

d Isolated yield.

### II.2 Scope and Synthetic Applications

**Figure 2:**
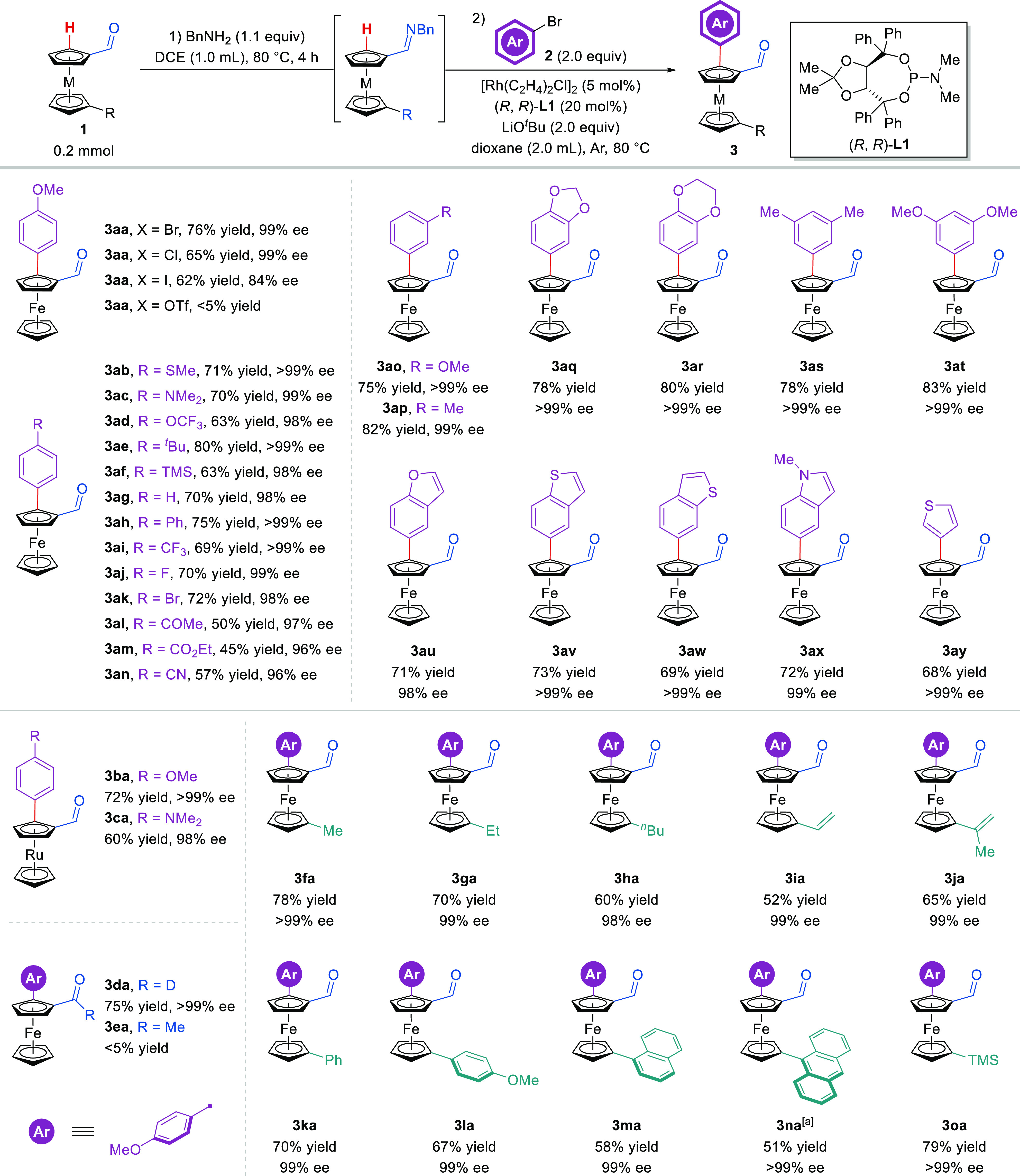
Scope of Rh(I)-catalyzed C–H arylation of ferroceneformaldehydes. Reaction conditions: **1** (0.2 mmol), BnNH_2_ (0.22 mmol) in DCE (1.0 mL) at 80 °C for 4 h, then **2** (0.4 mmol), [Rh(C_2_H_4_)_2_Cl]_2_ (0.01 mmol), (*R*,*R*)-**L1** (0.04 mmol), and LiO^*t*^Bu (0.4 mmol) in dioxane (2.0 mL) at 80 °C. Yields of isolated products are reported. ee values were determined by HPLC analysis on a stationary chiral stationary phase. Note: (a) [Rh(C_2_H_4_)_2_Cl]_2_ (0.02 mmol) and (*R*, *R*)-**L1** (0.08 mmol) were used.

With the optimized conditions in hand, the substrate scope of this reaction was investigated (Figure 2). First, a series of aryl halides was examined. Interestingly, the utilization of 4-methoxychlorobenzene could afford a moderate yield and excellent enantioselectivity (65% yield, 99% ee), and 4-methoxyiodobenzene was also tolerated (62% yield, 84% ee). Unfortunately, 4-methoxyphenyl 4-methylbenzenesulfonate led to no reaction. The chiral catalytic system readily promoted the reactions of ferroceneformaldehydes with various *para*-substituted phenyl bromides, and the desired planar chiral ferroceneformaldehydes **3ab**–**an** were obtained in reasonable yields (45–80%) with high enantioselectivity (96–>99% ee). For *meta*-substituted phenyl bromides, the reaction proceeded smoothly to generate the arylated products **3ao**,**ap** (75–82% yields, 99 to >99% ee). Good yields and excellent enantioselectivity were obtained for multisubstituted phenyl bromides (**3aq**–**at**, 78–83% yields, >99% ee). When 5-benzofuryl-, 5-benzothienyl-, 6-benzothienyl-, 5-(*N*-methyl)indolyl- or 3-thienyl-bromides were applied, the corresponding products **3au**–**ay** were delivered in 68–73% yields with 98–>99% ee. Meanwhile, the ruthenoceneformaldehydes participated in this reaction to give the products with excellent enantioselective control (**3ba**,**ca**, 60–72% yields, 98–>99% ee). Deuterated aldehyde substrate **1d** could lead to comparable results (**3da**, 75% yield, >99% ee). However, acetylferrocene was not reactive under the standard conditions likely due to the difficulty in the formation of ketimine.

Next, the reactions of ferroceneformaldehydes bearing diverse substituents, including alkyl (methyl, ethyl, and *n*-butyl), alkenyl (vinyl and 2-propenyl), aryl (phenyl, 4-methoxyphenyl, 1-naphthyl, 9-anthracenyl), and trimethylsilyl on the other Cp ring, were explored under the optimized conditions. Generally, the asymmetric induction of these reactions was very high, and excellent enantioselectivity was obtained for the corresponding ferroceneformaldehyde products **3fa**–**oa** (98–>99% ee). However, the increased bulkiness of the other Cp ring reduced the reactivity of these ferroceneformaldehyde derivatives. For example, the reaction of 1′-(9-anthracene)ferroceneformaldehyde **1n** could give acceptable results (**3na**, 51% yield, >99% ee) only with increased catalyst loading (10 mol % [Rh(C_2_H_4_)_2_Cl]_2_ and 40 mol % (*R*,*R*)-**L1**).

**Figure 3:**
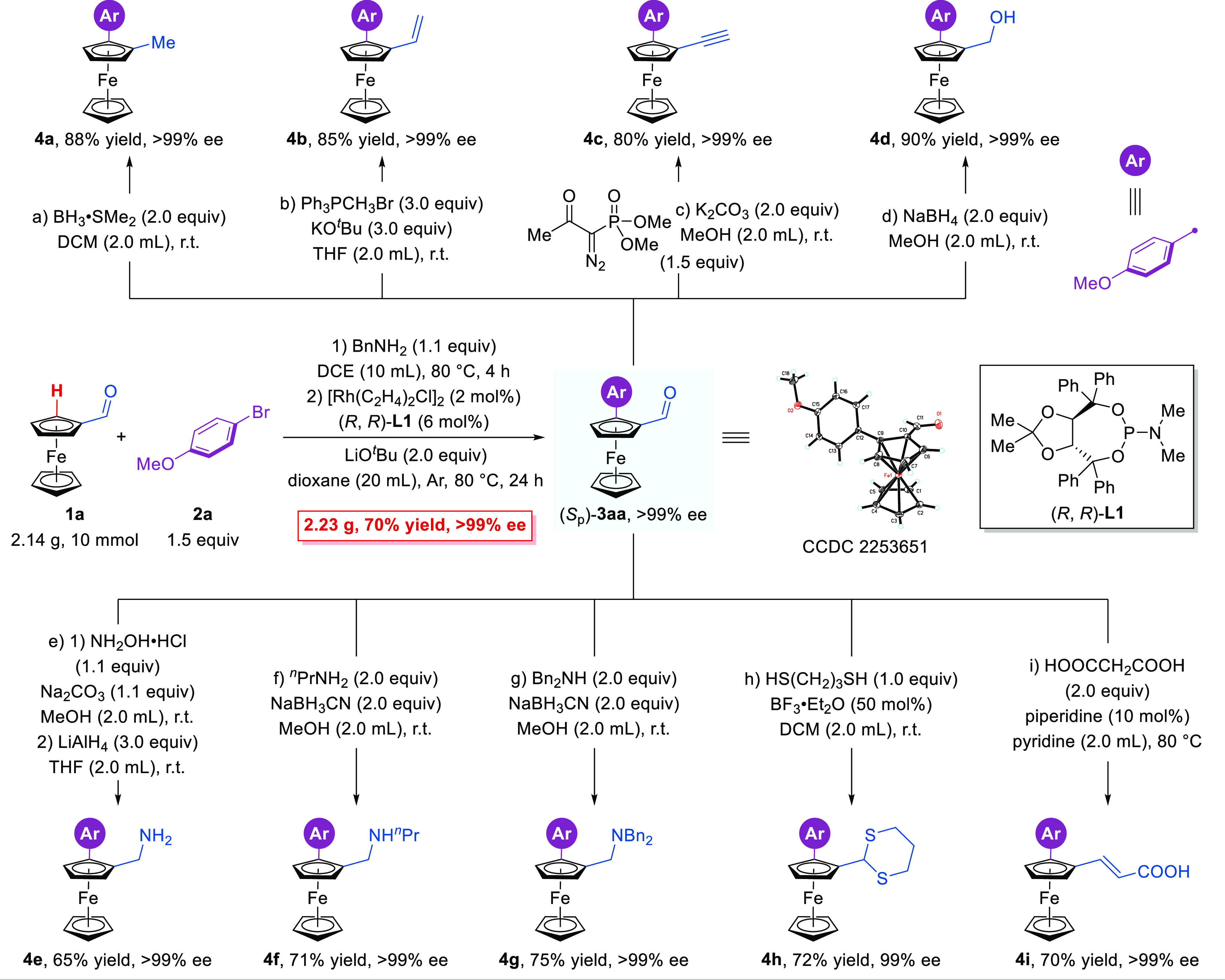
Gram-scale reaction and transformations of (*S*_p_)-**3aa**.

As a further demonstration of the utility of this method, a gram-scale reaction of **1a** (10 mmol) and **2a** was carried out. Pleasingly, the corresponding product **3aa** was obtained in 70% yield and >99% ee with only 2.0 mol % of [Rh(C_2_H_4_)_2_Cl]_2_. The absolute configuration of **3aa** was assigned as *S*_p_ by the X-ray diffraction analysis of an enantiopure sample. To further demonstrate the potential utility of this reaction, various transformations of product (*S*_p_)-**3aa** (>99% ee) were carried out (Figure 3). The aldehyde group could be transformed into diverse functional groups smoothly, such as methyl (**4a**), vinyl (**4b**), acetenyl (**4c**), hydroxyl (**4d**), primary amine (**4e**), secondary amine (**4f**), and tertiary amine (**4g**) without the loss of enantioselectivity. Meanwhile, the aldehyde group on **3aa** could be protected by thiols via simple protocols (**4h**, 72% yield, 99% ee). In addition, the Perkin reaction could be conducted to generate carboxylic acid **4i** (70% yield, >99% ee).

**Figure 4:**
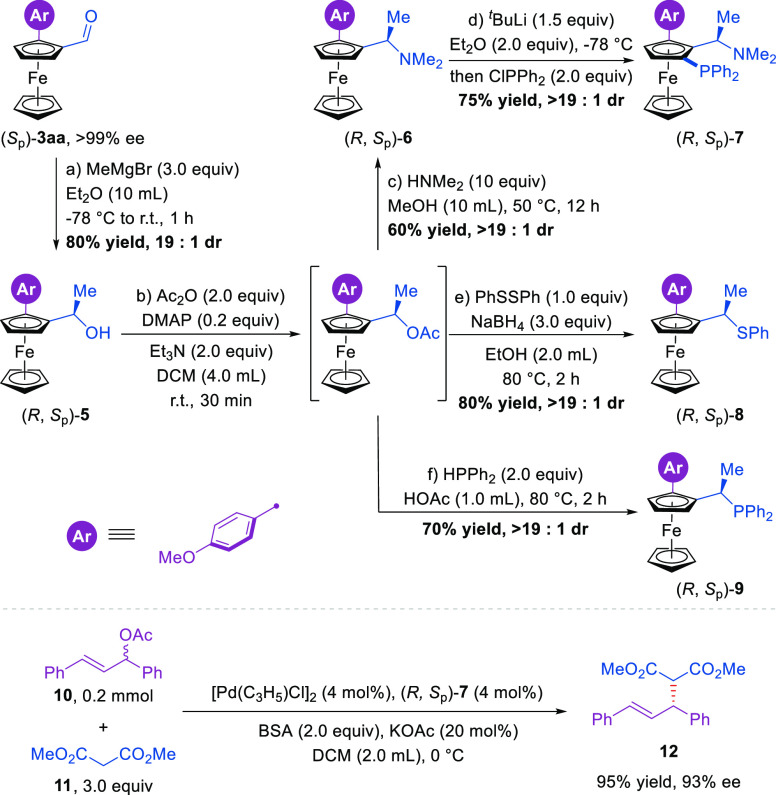
Product synthetic utility.

Subsequently, starting from (*S*_p_)-**3aa**, chiral benzyl alcohol (*R*,*S*_p_)-**5** could be synthesized efficiently in 80% yield and 19:1 dr via Grignard addition (Figure 4). (*R*,*S*_p_)-**5** could be transformed into Ugi’s amine derivative (*R*,*S*_p_)-**6** by a one-pot procedure, and its absolute configuration was confirmed by comparing with the literature^61^ (see the Supporting Information for details). Meanwhile, the chiral PPFA ligand derivative (*R*,*S*_p_)-**7** could be synthesized in 75% yield and >19:1 dr and was found to be an efficient ligand in a Pd-catalyzed asymmetric allylic alkylation reaction (95% yield, 93% ee). In addition, chiral thioether (*R*,*S*_p_)-**8** and monophosphine (*R*,*S*_p_)-**9** were synthesized in good yields and diastereoselectivity (70–80% yields, >19:1 dr). These transformations further enhance the synthetic utility of the current method.

### II.3 Mechanistic Studies

**Figure 5:**
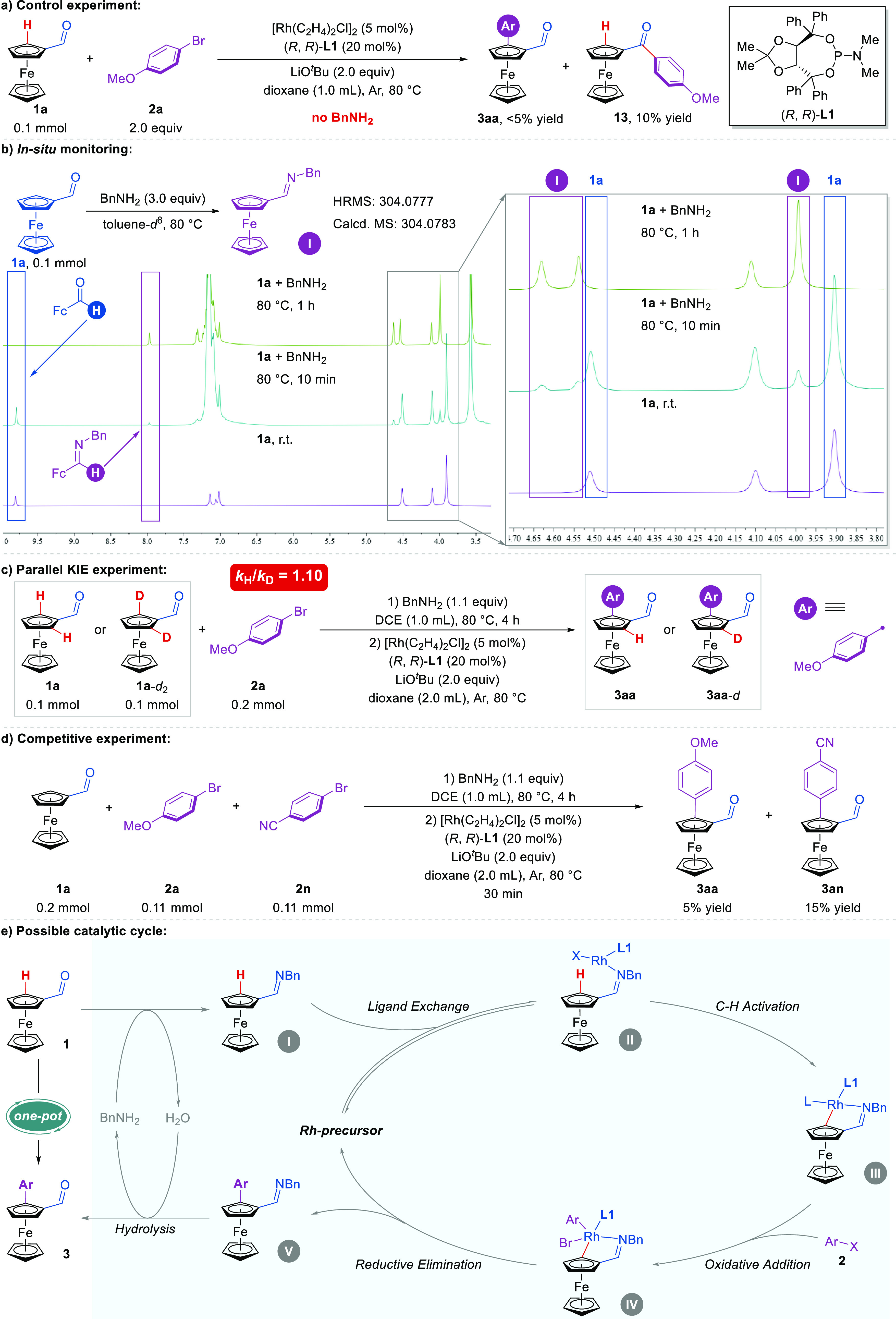
Mechanistic studies.

Preliminary experiments were carried out to gain insight into the reaction mechanism. Product **3aa** was not observed when the reaction of **1a** was carried out in the absence of benzylamine, and only product **13** was obtained in 10% yield (Figure 5a). This shows that the formation of an imine is necessary. *In situ* NMR and HRMS experiments further confirmed the formation of imine intermediate **I** (Figure 5b). Notably, a parallel KIE experiment in which the kinetic isotope effect of **1a** is only 1.10 (*k*_H_/*k*_D_) suggests that C–H cleavage is not the rate-determining step (Figure 5c). The competitive experiment between 4-bromoanisole **2a** and 4-bromobenzonitrile **2n** revealed that substrates bearing an electron-deficient group are more reactive than those with an electron-rich group (Figure 5d). Based on the above studies and a previous report,^27^ a plausible catalytic cycle was proposed for this enantioselective Rh(I)-catalyzed C–H arylation of ferroceneformaldehydes. As shown in Figure 5e, ferroceneformaldehyde **1** is dehydrated with benzylamine to form imine intermediate **I**. The coordination of **I** with the Rh precursor generates the intermediate **II**. Then, the intermediate **II** first is generated by directed C–H activation through a concerted metalation–deprotonation (CMD) pathway, delivering the intermediate **III**. Next, the oxidative addition of aryl halide **2** and reductive elimination proceed to give intermediate **V**. Finally, product **3** is obtained via hydrolysis of intermediate **V**.

## III Summary and Conclusions

In conclusion, we have developed a highly efficient synthesis of planar chiral ferroceneformaldehyde derivatives by enantioselective Rh(I)-catalyzed C–H arylation with readily available aryl halides under mild reaction conditions. These processes occur with excellent levels of monoarylation selectivity, enantioselectivity, and efficiency. Diverse functional groups were tolerated, and chiral Ugi’s amine and PPFA ligand analogues could be synthesized efficiently through simple protocols. The obtained chiral ferrocene ligand was found to be efficient in a Pd-catalyzed asymmetric allylic alkylation reaction. *In situ* NMR and HRMS experiments confirmed the formation of the imine intermediate. Further studies on enantioselective Rh(I)-catalyzed C–H functionalization reactions toward more diverse chiral molecules are ongoing in this laboratory.

## Supporting Information Available

(PDF) The Supporting Information is available free of charge at https://pubs.acs.org/doi/10.1021/acscentsci.3c00748.

Experimental procedures, complete characterization data, and copies of ^1^H, ^13^C, and ^19^F NMR spectra (PDF)
Crystallographic data for **3aa** (CIF)
NMR fid files (ZIP)

## Supplementary Material

oc3c00748_si_001.pdf

oc3c00748_si_002.cif

## References

[ref1] Davies H. M. L.; Beckwith R. E. J. Catalytic Enantioselective C-H Activation by Means of Metal-Carbenoid-Induced C-H Insertion. Chem. Rev. 2003, 103, 2861–2904. 10.1021/cr0200217. 12914484

[ref2] Newton C. G.; Wang S.-G.; Oliveira C. C.; Cramer N. Catalytic Enantioselective Transformations Involving C-H Bond Cleavage by Transition-Metal Complexes. Chem. Rev. 2017, 117, 8908–8976. 10.1021/acs.chemrev.6b00692. 28212007

[ref3] Saint-Denis T. G.; Zhu R.-Y.; Chen G.; Wu Q.-F.; Yu J.-Q. Enantioselective C(sp^3^)-H Bond Activation by Chiral Transition Metal Catalysts. Science 2018, 359, eaao4798 10.1126/science.aao4798. 29449462 PMC5862070

[ref4] Gandeepan P.; Ackermann L. Transient Directing Groups for Transformative C-H Activation by Synergistic Metal Catalysis. Chem. 2018, 4, 199–222. 10.1016/j.chempr.2017.11.002.

[ref5] Li G.; Jiang J.; Xie H.; Wang J. Introducing the Chiral Transient Directing Group Strategy to Rhodium(III)-Catalyzed Asymmetric C–H Activation. Chem. Eur. J. 2019, 25, 4688–4694. 10.1002/chem.201900762. 30784129

[ref6] Yang K.; Song M.; Liu H.; Ge H. Palladium-catalyzed Direct Asymmetric C-H Bond Functionalization Enabled by the Directing Group Strategy. Chem. Sci. 2020, 11, 12616–12632. 10.1039/D0SC03052J. 34123236 PMC8163320

[ref7] Wu Y.-J.; Shi B.-F. Transition Metal-Catalyzed C-H Activation via Imine-Based Transient Directing Group Strategy. Chin. J. Org. Chem. 2020, 40, 3517–3535. 10.6023/cjoc202003057.

[ref8] Zhan B.-B.; Jin L.; Shi B.-F. Palladium-catalyzed Enantioselective C-H Functionalization via C-H Palladation. Trends Chem. 2022, 4, 220–235. 10.1016/j.trechm.2021.12.005.

[ref9] Liu C.-X.; Yin S.-Y.; Zhao F.; Yang H.; Feng Z.; Gu Q.; You S.-L. Rhodium-Catalyzed Asymmetric C-H Functionalization Reactions. Chem. Rev. 2023, 123, 10079–10134. 10.1021/acs.chemrev.3c00149. 37527349

[ref10] Colby D. A.; Bergman R. G.; Ellman J. A. Rhodium-Catalyzed C–C Bond Formation via Heteroatom-Directed C–H Bond Activation. Chem. Rev. 2010, 110, 624–655. 10.1021/cr900005n. 19438203 PMC2820156

[ref11] Willis M. C. Transition Metal Catalyzed Alkene and Alkyne Hydroacylation. Chem. Rev. 2010, 110, 725–748. 10.1021/cr900096x. 19873977

[ref12] Kim D.-S.; Park W.-J.; Jun C.-H. Metal-Organic Cooperative Catalysis in C-H and C-C Bond Activation. Chem. Rev. 2017, 117, 8977–9015. 10.1021/acs.chemrev.6b00554. 28060495

[ref13] Davison R. T.; Kuker E. L.; Dong V. M. Teaching Aldehydes New Tricks Using Rhodium- and Cobalt-Hydride Catalysis. Acc. Chem. Res. 2021, 54, 1236–1250. 10.1021/acs.accounts.0c00771. 33533586 PMC8486976

[ref14] Thalji R. K.; Ellman J. A.; Bergman R. G. Highly Efficient and Enantioselective Cyclization of Aromatic Imines via Directed C–H Bond Activation. J. Am. Chem. Soc. 2004, 126, 7192–7193. 10.1021/ja0394986. 15186153

[ref15] Tanaka K.; Otake Y.; Sagae H.; Noguchi K.; Hirano M. Highly Regio-, Diastereo-, and Enantioselective [2 + 2 + 2] Cycloaddition of 1,6-Enynes with Electron-Deficient Ketones Catalyzed by a Cationic Rh^I^/H_8_-binap Complex. Angew. Chem., Int. Ed. 2008, 47, 1312–1316. 10.1002/anie.200704758. 18183562

[ref16] Li Q.; Yu Z.-X. Enantioselective Rhodium-Catalyzed Allylic C-H Activation for the Addition to Conjugated Dienes. Angew. Chem., Int. Ed. 2011, 50, 2144–2147. 10.1002/anie.201005215. 21344571

[ref17] Tran D. N.; Cramer N. Enantioselective Rhodium(I)-Catalyzed [3 + 2] Annulations of Aromatic Ketimines Induced by Directed C-H Activations. Angew. Chem., Int. Ed. 2011, 50, 11098–11102. 10.1002/anie.201105766. 21976453

[ref18] Kuninobu Y.; Yamauchi K.; Tamura N.; Seiki T.; Takai K. Rhodium-Catalyzed Asymmetric Synthesis of Spirosilabifluorene Derivatives. Angew. Chem., Int. Ed. 2013, 52, 1520–1522. 10.1002/anie.201207723. 23239057

[ref19] Filloux C. M.; Rovis T. Rh(I)-Bisphosphine-Catalyzed Asymmetric, Intermolecular Hydroheteroarylation of α-Substituted Acrylate Derivatives. J. Am. Chem. Soc. 2015, 137, 508–517. 10.1021/ja511445x. 25545834 PMC4304441

[ref20] Murai M.; Matsumoto K.; Takeuchi Y.; Takai K. Rhodium-Catalyzed Synthesis of Benzosilolometallocenes via the Dehydrogenative Silylation of C(sp^2^)-H Bonds. Org. Lett. 2015, 17, 3102–3105. 10.1021/acs.orglett.5b01373. 26061112

[ref21] Zhang Q.-W.; An K.; Liu L.-C.; Yue Y.; He W. Rhodium-Catalyzed Enantioselective Intramolecular C-H Silylation for the Syntheses of Planar-Chiral Metallocene Siloles. Angew. Chem., Int. Ed. 2015, 54, 6918–6921. 10.1002/anie.201502548. 25907416

[ref22] Shibata T.; Shizuno T.; Sasaki T. Enantioselective Synthesis of Planar-chiral Benzosiloloferrocenes by Rh-catalyzed Intramolecular C-H Silylation. Chem. Commun. 2015, 51, 7802–780. 10.1039/C5CC00723B. 25687020

[ref23] Lee T.; Hartwig J. F. Rhodium-Catalyzed Enantioselective Silylation of Cyclopropyl C–H Bonds. Angew. Chem., Int. Ed. 2016, 55, 8723–8727. 10.1002/anie.201603153. PMC4976637 27253898

[ref24] Zhao W.-T.; Lu Z.-Q.; Zheng H.; Xue X.-S.; Zhao D. Rhodium-Catalyzed 2-Arylphenol-Derived Six-Membered Silacyclization: Straightforward Access toward Dibenzooxasilines and Silicon-Containing Planar Chiral Metallocenes. ACS Catal. 2018, 8, 7997–8005. 10.1021/acscatal.8b01992.

[ref25] Mu D.; Yuan W.; Chen S.; Wang N.; Yang B.; You L.; Zu B.; Yu P.; He C. Streamlined Construction of Silicon-Stereogenic Silanes by Tandem Enantioselective C-H Silylation/Alkene Hydrosilylation. J. Am. Chem. Soc. 2020, 142, 13459–13468. 10.1021/jacs.0c04863. 32697094

[ref26] Ma W.; Liu L.-C.; An K.; He T.; He W. Rhodium-Catalyzed Synthesis of Chiral Monohydrosilanes by Intramolecular C–H Functionalization of Dihydrosilanes. Angew. Chem., Int. Ed. 2021, 60, 4245–4251. 10.1002/anie.202013041. 33164311

[ref27] Kim J. H.; Greßies S.; Boultadakis-Arapinis M.; Daniliuc C.; Glorius F. Rh(I)/NHC*-Catalyzed Site- and Enantioselective Functionalization of C(sp^3^)-H Bonds Toward Chiral Triarylmethanes. ACS Catal. 2016, 6, 7652–7656. 10.1021/acscatal.6b02392.

[ref28] Greßies S.; Klauck F. J. R.; Kim J. H.; Daniliuc C. G.; Glorius F. Ligand-Enabled Enantioselective Csp^3^-H Activation of Tetrahydroquinolines and Saturated Aza-Heterocycles by Rh^I^. Angew. Chem., Int. Ed. 2018, 57, 9950–995. 10.1002/anie.201805680. 29883522

[ref29] Liu C.-X.; Xie P.-P.; Zhao F.; Wang Q.; Feng Z.; Wang H.; Zheng C.; You S.-L. Explicit Mechanism of Rh(I)-Catalyzed Asymmetric C-H Arylation and Facile Synthesis of Planar Chiral Ferrocenophanes. J. Am. Chem. Soc. 2023, 145, 4765–4773. 10.1021/jacs.2c13542. 36787487

[ref30] Chiral Ferrocenes in Asymmetric Catalysis; Dai L.-X., Hou X.-L., Eds.; Wiley: 2010.

[ref31] Richards C. J.; Locke A. J. Recent Advances in the Generation of Non-racemic Ferrocene Derivatives and Their Application to Asymmetric Aynthesis. Tetrahedron: Asymmetry 1998, 9, 2377–2407. 10.1016/S0957-4166(98)00251-1.

[ref32] Colacot T. J. A Concise Update on the Applications of Chiral Ferrocenyl Phosphines in Homogeneous Catalysis Leading to Organic Synthesis. Chem. Rev. 2003, 103, 3101–3118. 10.1021/cr000427o. 12914493

[ref33] Fu G. C. Asymmetric Catalysis with “Planar-Chiral” Derivatives of 4-(Dimethylamino)pyridine. Acc. Chem. Res. 2004, 37, 542–547. 10.1021/ar030051b. 15311953

[ref34] Gomez Arrayas R.; Adrio J.; Carretero J. C. Recent Applications of Chiral Ferrocene Ligands in Asymmetric Catalysis. Angew. Chem., Int. Ed. 2006, 45, 7674–7715. 10.1002/anie.200602482. 17115468

[ref35] Schaarschmidt D.; Lang H. Selective Syntheses of Planar-Chiral Ferrocenes. Organometallics 2013, 32, 5668–570. 10.1021/om400564x.

[ref36] Yoshida K.; Yasue R. Planar-Chiral Ferrocene-Based N-Heterocyclic Carbene Ligands. Chem. Eur. J. 2018, 24, 18575–18586. 10.1002/chem.201803903. 30277615

[ref37] Cunningham L.; Benson A.; Guiry P. J. Recent Developments in the Synthesis and Applications of Chiral Ferrocene Ligands and Organocatalysts in Asymmetric Catalysis. Org. Biomol. Chem. 2020, 18, 9329–9370. 10.1039/D0OB01933J. 33155613

[ref38] Urbano A.; Hernández-Torres G.; del Hoyo A. M.; Martínez-Carrión A.; Carreño M. C. Mild Access to Planar-chiral Ortho-condensed Aromatic Ferrocenes via Gold(I)-catalyzed Cycloisomerization of Ortho-alkynylaryl Ferrocenes. Chem. Commun. 2016, 52, 6419–6422. 10.1039/C6CC02624A. 27094457

[ref39] López L. A.; López E. Recent Advances in Transition Metal-catalyzed C-H Bond Functionalization of Ferrocene Derivatives. Dalton. Trans. 2015, 44, 10128–10135. 10.1039/C5DT01373A. 25973598

[ref40] Arae S.; Ogasawara M. Catalytic Asymmetric Synthesis of Planar-chiral Transition-metal Complexes. Tetrahedron Lett. 2015, 56, 1751–1761. 10.1016/j.tetlet.2015.01.130.

[ref41] Gao D.-W.; Gu Q.; Zheng C.; You S.-L. Synthesis of Planar Chiral Ferrocenes via Transition-Metal-Catalyzed Direct C-H Bond Functionalization. Acc. Chem. Res. 2017, 50, 351–365. 10.1021/acs.accounts.6b00573. 28121428

[ref42] Liu C.-X.; Gu Q.; You S.-L. Asymmetric C-H Bond Functionalization of Ferrocenes: New Opportunities and Challenges. Trends Chem. 2020, 2, 737–749. 10.1016/j.trechm.2020.05.003.

[ref43] Gao D.-W.; Shi Y.-C.; Gu Q.; Zhao Z.-L.; You S.-L. Enantioselective Synthesis of Planar Chiral Ferrocenes via Palladium-Catalyzed Direct Coupling with Arylboronic Acids. J. Am. Chem. Soc. 2013, 135, 86–89. 10.1021/ja311082u. 23253097

[ref44] Pi C.; Li Y.; Cui X.-L.; Zhang H.; Han Y.-B.; Wu Y.-J. Redox of Ferrocene Controlled Asymmetric Dehydrogenative Heck Reaction via Palladium-catalyzed Dual C-H Bond Activation. Chem. Sci. 2013, 4, 2675–2679. 10.1039/c3sc50577d.

[ref45] Luo S.; Xiong Z.; Lu Y.; Zhu Q. Enantioselective Synthesis of Planar Chiral Pyridoferrocenes via Palladium-Catalyzed Imidoylative Cyclization Reactions. Org. Lett. 2018, 20, 1837–1840. 10.1021/acs.orglett.8b00348. 29537285

[ref46] Xu J.; Liu Y.; Zhang J.; Xu X.; Jin Z. Palladium-catalyzed Enantioselective C(sp^2^)-H Arylation of Ferrocenyl Ketones Enabled by a Chiral Transient Directing Group. Chem. Commun. 2018, 54, 689–692. 10.1039/C7CC09273C. 29303517

[ref47] Xu B.-B.; Ye J.; Yuan Y.; Duan W.-L. Palladium-Catalyzed Asymmetric C-H Arylation for the Synthesis of Planar Chiral Benzothiophene-Fused Ferrocenes. ACS Catal. 2018, 8, 11735–11740. 10.1021/acscatal.8b03912.

[ref48] Cai Z.-J.; Liu C.-X.; Wang Q.; Gu Q.; You S.-L. Thioketone-directed Rhodium(I) Catalyzed Enantioselective C-H Bond Arylation of Ferrocenes. Nat. Commun. 2019, 10, 4168 10.1038/s41467-019-12181-x. 31519893 PMC6744407

[ref49] Jia L.; Liu X.; Zhang A.-A.; Wang T.; Hua Y.; Li H.; Liu L. Synthesis of Planar Chiral Ferrocenes via a Pd(0)-catalyzed *syn*-Carbopalladation/asymmetric C-H Alkenylation Process. Chem. Commun. 2020, 56, 1737–1740. 10.1039/C9CC06529F. 31938796

[ref50] Liu L.; Song H.; Liu Y.-H.; Wu L.-S.; Shi B.-F. Achiral Cp^x^Ir(III)/Chiral Carboxylic Acid Catalyzed Enantioselective C-H Amidation of Ferrocenes under Mild Conditions. ACS Catal. 2020, 10, 7117–7122. 10.1021/acscatal.0c02049.

[ref51] Chen H.; Wang Y.-X.; Luan Y.-X.; Ye M. Enantioselective Twofold C–H Annulation of Formamides and Alkynes without Built-in Chelating Groups. Angew. Chem., Int. Ed. 2020, 59, 9428–9432. 10.1002/anie.202001267. 32154983

[ref52] Liu C.-X.; Cai Z.-J.; Wang Q.; Wu Z.-J.; Gu Q.; You S.-L. Rhodium-Catalyzed Pyridine-Assisted C-H Arylation for the Synthesis of Planar Chiral Ferrocenes. CCS Chem. 2020, 2, 642–651. 10.31635/ccschem.020.202000157.

[ref53] Lou S.-J.; Zhuo Q.; Nishiura M.; Luo G.; Hou Z. Enantioselective C-H Alkenylation of Ferrocenes with Alkynes by Half-Sandwich Scandium Catalyst. J. Am. Chem. Soc. 2021, 143, 2470–2476. 10.1021/jacs.0c13166. 33529525

[ref54] Zhang P.-C.; Li Y.-L.; He J.; Wu H.-H.; Li Z.; Zhang J. Simultaneous Construction of Axial and Planar Chirality by Gold/TY-Phos-catalyzed Asymmetric Hydroarylation. Nat. Commun. 2021, 12, 4609 10.1038/s41467-021-24678-5. 34326337 PMC8322429

[ref55] Liu C.-X.; Zhang W.-W.; Yang P.; Zhao F.; Feng Z.; Wang Q.; Zhang S.-Z.; Gu Q.; You S.-L. Pd-catalyzed Asymmetric Oxidative C-H/C-H Cross-coupling Reaction Between Dialkylaminomethylferrocenes and Indolizines. Chem. Catal. 2022, 2, 102–113. 10.1016/j.checat.2021.11.001.

[ref56] Zou X.; Li Y.; Ke Z.; Xu S. Chiral Bidentate Boryl Ligand-Enabled Iridium-Catalyzed Enantioselective Dual C-H Borylation of Ferrocenes: Reaction Development and Mechanistic Insights. ACS Catal. 2022, 12, 1830–1840. 10.1021/acscatal.1c05299.

[ref57] Liu C.-X.; Zhao F.; Feng Z.; Wang Q.; Gu Q.; You S.-L. Kinetic Resolution of Planar Chiral Metallocenes Using Rh-catalysed Enantioselective C-H Arylation. Nat. Synth. 2023, 2, 49–57. 10.1038/s44160-022-00177-3.

[ref58] Dongbang S.; Confair D. N.; Ellman J. A. Rhodium-Catalyzed C-H Alkenylation/Electrocyclization Cascade Provides Dihydropyridines That Serve as Versatile Intermediates to Diverse Nitrogen Heterocycles. Acc. Chem. Res. 2021, 54, 1766–1778. 10.1021/acs.accounts.1c00027. 33740369 PMC8026680

[ref59] Duttwyler S.; Chen S.; Takase M. K.; Wiberg K. B.; Bergman R. G.; Ellman J. A. Proton Donor Acidity Controls Selectivity in Nonaromatic Nitrogen Heterocycle Synthesis. Science 2013, 339, 678–682. 10.1126/science.1230704. 23393259 PMC3809088

[ref60] Walker M. M.; Chen S.; Mercado B. Q.; Houk K. N.; Ellman J. A. Formation of Aminocyclopentadienes from Silyldihydropyridines: Ring Contractions Driven by Anion Stabilization. Angew. Chem., Int. Ed. 2018, 57, 6605–6609. 10.1002/anie.201800596. PMC6040663 29570926

[ref61] Plevová K.; Mudráková B.; Rakovský E.; Sěbesta R. Diastereoselective Pd-Catalyzed C-H Arylation of Ferrocenylmethanamines with Arylboronic Acids or Pinacol Esters. J. Org. Chem. 2019, 84, 7312–7319. 10.1021/acs.joc.9b00953. 31042391

