# Supplementary material for: Enantioselective Rh(I)-Catalyzed C–H Arylation of Ferroceneformaldehydes
Source: ACS Cent Sci. 2023 Sep 28;9(11):2036–43. doi: 10.1021/acscentsci.3c00748 (PMC10683487; doi:10.1021/acscentsci.3c00748)
Supplement: Supplementary file 1 — oc3c00748_si_001.pdf [file oc3c00748_si_001.pdf]

Supplementary Information for

Enantioselective Rh(I)-Catalyzed C-H Arylation of Ferroceneformaldehydes

Chen-Xu Liu,[†,‡] Fangnuo Zhao,[†,‡] Qing Gu,[†] Shu-Li You[*,†]

[†]New Cornerstone Science Laboratory, State Key Laboratory of Organometallic Chemistry, Shanghai Institute of Organic Chemistry, University of Chinese Academy of Sciences, Chinese Academy of Sciences, 345 Lingling Lu, Shanghai 200032, China

Email: slyou@sioc.ac.cn

[#]These authors contributed equally to this work.

Table of Contents

General methods S3

General procedure for the synthesis of substrates..S4

General procedure for enantioselective Rh(I)-catalyzed C-H arylation of

ferroceneformaldehydes..S12

Transformations of (S_p)-**3aa** S38

Synthetic utility of product S50

Determination of the absolute configuration of (R, S_p)-**6** S57

General procedure for H/D KIE experiments S60

X-Ray crystal structure of **3aa**..S62

References..S65

Copies of NMR spectra and HPLC chromatograms..S66

General methods

Unless otherwise noted, all reactions were carried out in flame-dried glassware under a dry argon atmosphere. All solvents were purified and dried according to standard methods prior to use. All aryl halides were obtained from the commercial sources and used directly without further purification. Rhodium precursors and LiO^{t}Bu were obtained from Strem, Alfa, Adamas, Bidepharm and J&K and used directly without further purification. Ligands **L1-11** were prepared according to the reported procedure.[1-3] ^{1}H and ^{13}C NMR spectra were recorded on a Bruker instrument (400 MHz and 100 MHz, respectively) or an Agilent instrument (400 MHz and 100 MHz, respectively) and internally referenced to tetramethylsilane signal or residual protic solvent signals. ^{19}F NMR spectra were recorded on a Bruker or Agilent instrument (376 MHz) and externally referenced to $CFCl_3$. ^{31}P NMR spectra were recorded on a Bruker or Agilent instrument (162 MHz) and externally referenced to 85% H_3PO_4 aqueous solution. Data for ^{1}H NMR are recorded as follows: chemical shift (δ, ppm), multiplicity (s = singlet, d = doublet, t = triplet, m = multiplet or unresolved, br = broad singlet, coupling constant (s) in Hz, integration). Data for ^{13}C NMR, ^{19}F NMR and ^{31}P NMR are reported in terms of chemical shift (δ, ppm).

General procedure for the synthesis of substrates

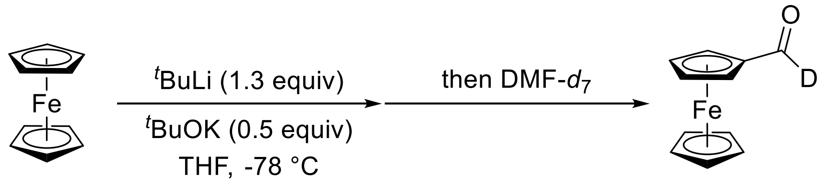

1d

t-BuLi (1.3 M in pentane, 10 mL, 13 mmol, 1.3 equiv) was slowly added to a solution of ferrocene (1.86 g, 10 mmol, 1.0 equiv) and potassium *tert*-butoxide (561.1 mg, 5 mmol, 0.5 equiv) in THF (30 mL) at -78 ℃ in a dry flask. After the mixture was stirred at this temperature for 2 h, then *N*, *N*-dimethylformamide-d_7 (1.5 mL, 20 mmol, 2.0 equiv) was added. After 5 min, the mixture was warmed to room temperature and stirred for an additional hour. The reaction was then quenched by adding $NaHCO_3$ solution (aq. 2 M, 20 mL). The mixture was extracted with ethyl acetate (20 mL × 3). The combined organic phases were washed with brine and dried over anhydrous sodium sulfate. After filtration, the solvent was removed under reduced pressure. The residue was purified by silica gel column chromatography (hexane/ethyl acetate = 20/1, v/v) to afford product **1d**.

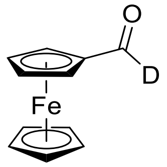

1d. Foamy dark red solid (1.41 g, 66% yield).

Analytical data for **1d**:

^{1}H NMR (400 MHz, $CDCl_3$) δ 4.80-4.78 (m, 2H), 4.61-4.59 (m, 2H), 4.27 (s, 5H).

^{13}C NMR (101 MHz, $CDCl_3$) δ 79.2, 73.2, 70.0, 69.6.

IR (neat): ν_{max} (cm^{-1}) = 1657, 1450, 1411, 1371, 1250, 1107, 1066, 1008, 825, 716.

HRMS (ESI) calcd for $C_{11}H_9[^{56}Fe]OD$ $[M]^+$: 215.0138. Found: 215.0140.

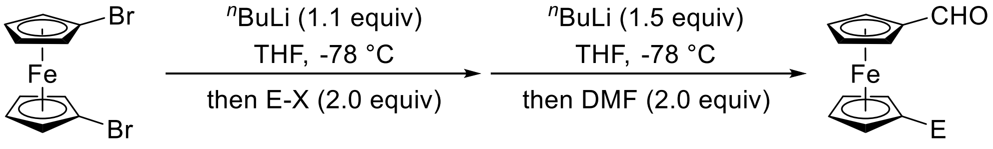

1

Step 1. *n*-BuLi (2.5 M in pentane, 1.1 equiv) was slowly added to a solution of 1,1'-dibromoferrocene (1.0 equiv) in tetrahydrofuran (5.0 mL) at –78 °C in a dry flask. After the mixture was stirred at this temperature for 2 h, then the corresponding electrophilic reagent (2.0 equiv) was added. After 5 min, the mixture was warmed to room temperature and stirred for an additional hour. The reaction was then quenched by adding $NaHCO_3$ solution (aq. 2 M, 20 mL). The mixture was extracted with ethyl acetate (20 mL × 3). The combined organic phases were washed with brine and dried over anhydrous sodium sulfate. After filtration, the solvent was removed in vacuo to give a yellow oil, which was used without further purification.

Step 2. *n*-BuLi (2.5 M in pentane, 1.5 equiv) was slowly added to a solution of the above yellow oil in tetrahydrofuran (5.0 mL) at –78 °C in a dry flask. After the mixture was stirred at this temperature for 2 h, then *N*, *N*-dimethylformamide (2.0 equiv) was added. After 5 min, the mixture was warmed to room temperature and stirred for an additional hour. The reaction was then quenched by adding $NaHCO_3$ solution (aq. 2 M, 20 mL). The mixture was extracted with ethyl acetate (20 mL × 3). The combined organic phases were washed with brine and dried over anhydrous Na_2SO_4. After filtration, the solvent was removed under reduced pressure. The residue was purified by silica gel column chromatography (hexane/ethyl acetate = 10/1, v/v) to afford product **1**.

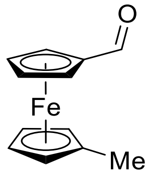

1f. Dark red oil (0.68 g, 60% yield, 5 mmol scale).

Analytical data for **1f**:

^{1}H NMR (400 MHz, $CDCl_3$) δ 9.92 (s, 1H), 4.72-4.70 (m, 2H), 4.55-4.53 (m, 2H), 4.17 (t, J = 2.0 Hz, 2H), 4.13 (t, J = 2.0 Hz, 2H), 1.91 (s, 3H).

^{13}C NMR (101 MHz, $CDCl_3$) δ 193.5, 86.1, 79.6, 73.9, 70.6, 70.2, 68.8, 14.2.

IR (neat): ν_{max} (cm^{-1}) = 3085, 2952, 2919, 2820, 2757, 1677, 1659, 1475, 1453, 1407, 1386, 1368, 1331, 1244, 1199, 1035, 1026, 920, 820, 741, 729, 646, 617.

HRMS (ESI) calcd for $C_{12}H_{12}[^{56}Fe]O$ $[M]^+$: 228.0232. Found: 228.0229.

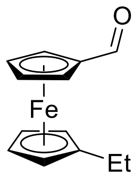

1g. Dark red oil (1.34 g, 55% yield, 10 mmol scale).

Analytical data for **1g**:

^{1}H NMR (400 MHz, $CDCl_3$) δ 9.92 (s, 1H), 4.71 (t, J = 2.0 Hz, 2H), 4.55 (t, J = 2.0 Hz, 2H), 4.16 (s, 4H), 2.27 (q, J = 7.6 Hz, 2H), 1.12 (t, J = 7.6 Hz, 3H).

^{13}C NMR (101 MHz, $CDCl_3$) δ 193.4, 93.2, 79.5, 73.7, 70.0, 69.0, 68.7, 21.7, 14.6.

IR (neat): ν_{max} (cm^{-1}) = 2966, 1679, 1455, 1403, 1370, 1316, 1245, 1036, 906, 826, 743, 619.

HRMS (ESI) calcd for $C_{13}H_{14}[^{56}Fe]O$ $[M]^+$: 242.0389. Found: 242.0390.

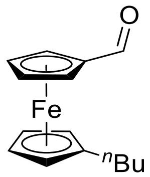

1h. Dark red oil (1.36 g, 50% yield, 10 mmol scale).

Analytical data for **1h**:

^{1}H NMR (400 MHz, $CDCl_3$) δ 9.91 (s, 1H), 4.70 (t, J = 2.0 Hz, 2H), 4.54-4.52 (m, 2H), 4.14 (s, 4H), 2.24 (t, J = 7.2 Hz, 2H), 1.46-1.38 (m, 2H), 1.35-1.25 (m, 2H), 0.89 (t, J = 7.2 Hz, 3H).

^{13}C NMR (101 MHz, $CDCl_3$) δ 193.4, 91.6, 79.5, 73.8, 70.0, 69.6, 68.7, 33.2, 28.6, 22.4, 13.9.

IR (neat): ν_{max} (cm^{-1}) = 2956, 2930, 2861, 1680, 1455, 1370, 1245, 1033, 925, 825, 744, 617.

HRMS (ESI) calcd for $C_{15}H_{18}[^{56}Fe]O$ $[M]^+$: 270.0702. Found: 270.0702.

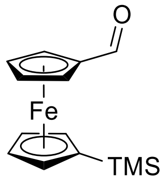

1o. Dark red oil (0.79 g, 55% yield, 5 mmol scale).

Analytical data for **1o**:

^{1}H NMR (400 MHz, $CDCl_3$) δ 9.95 (s, 1H), 4.76 (t, J = 2.0 Hz, 2H), 4.57-4.55 (m, 2H), 4.47-4.45 (m, 2H), 4.18-4.16 (m, 2H), 0.23 (s, 9H).

^{13}C NMR (101 MHz, $CDCl_3$) δ 193.4, 79.2, 74.7, 74.3, 73.3, 72.3, 69.7, -0.4.

IR (neat): ν_{max} (cm^{-1}) = 2953, 2896, 1681, 1664, 1520, 1479, 1455, 1406, 1383, 1371, 1333, 1244, 1182, 1162, 1036, 900, 873, 823, 740, 692, 647, 628.

HRMS (ESI) calcd for $C_{14}H_{19}[^{56}Fe]OSi$ $[M+H]^+$: 287.0549. Found: 287.0546.

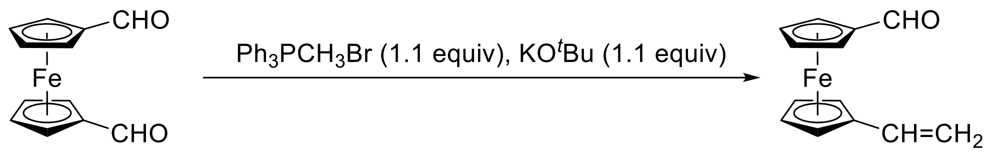

1i

t-BuOK (0.25 g, 2.2 mmol, 1.1 equiv) was added to a suspension of methyltriphenyl-phosphonium bromide (0.79 g, 2.2 mmol, 1.1 equiv) in dry THF (5 mL) at 0 °C and then stirred for 0.5 h at room temperature. Then 1,1′-ferrocenedicarboxaldehyde[4] (0.48 g, 2.0 mmol, 1.0 equiv) in dry THF (5.0 mL) was added into the above-mentioned solution at 0 °C. The reaction mixture was stirred for another 3 h at room temperature. After the reaction was complete (monitored by TLC), the reaction was quenched by adding $NaHCO_3$ solution (aq. 2 M, 20 mL). The mixture was extracted with ethyl acetate (20 mL × 3). The combined organic phases were washed with brine and dried over anhydrous Na_2SO_4. After filtration, the solvent was removed under reduced pressure. The residue was purified by silica gel column chromatography (hexane/ethyl acetate = 10/1, v/v) to afford product **1i**.

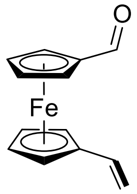

1i. Dark red oil (0.14 g, 30% yield).

Analytical data for **1i**:

^{1}H NMR (400 MHz, $CDCl_3$) δ 9.87 (s, 1H), 6.34 (dd, J = 17.6, 10.8 Hz, 1H), 5.38 (d, J = 17.6 Hz, 1H), 5.14 (d, J = 10.8 Hz, 1H), 4.71 (t, J = 2.0 Hz, 2H), 4.53 (t, J = 2.0 Hz, 2H), 4.45 (t, J = 2.0 Hz, 2H), 4.30 (t, J = 2.0 Hz, 2H).
^{13}C NMR (101 MHz, $CDCl_3$) δ 193.7, 132.8, 113.3, 85.5, 79.7, 74.4, 70.6, 70.2, 68.0.
IR (neat): ν_{max} (cm^{-1}) = 2983, 2904, 1679, 1453, 1400, 1248, 1052, 892, 826.
HRMS (ESI) calcd for $C_{13}H_{12}[^{56}Fe]O$ $[M]^+$: 240.0232. Found: 240.0235.

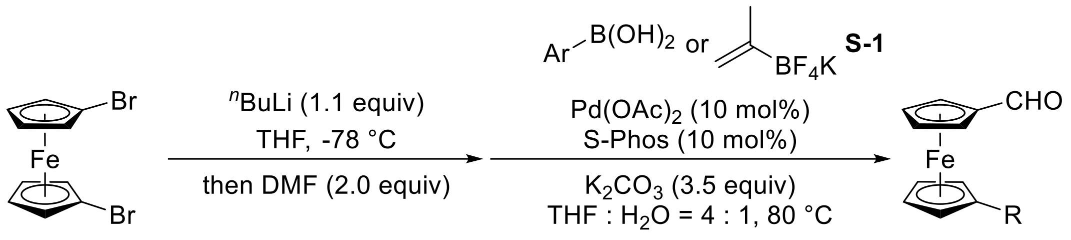

1

Step 1. *n*-BuLi (2.5 M in pentane, 1.1 equiv) was slowly added to a solution of 1,1'-dibromoferrocene (1.0 equiv) in tetrahydrofuran (5.0 mL) at –78 ℃ in a dry flask. After the mixture was stirred at this temperature for 2 h, *N*, *N*-dimethylformamide (2.0 equiv) was added. After 5 min, the mixture was warmed to room temperature and stirred for an additional hour. The reaction was then quenched by adding $NaHCO_3$ solution (aq. 2 M, 20 mL). The mixture was extracted with ethyl acetate (20 mL × 3). The combined organic phases were washed with brine and dried over anhydrous sodium sulfate. After filtration, the solvent was removed in vacuo to give a yellow oil, which was used without further purification.

Step 2. A standard 100 mL Schlenk tube was charged with K_2CO_3 (3.5 equiv), **S-1** (2.0 equiv), S-Phos (10 mol%), and $Pd(OAc)_2$ (10 mol%). Then, the flask was evacuated and backfilled with argon for 3 times, and followed by addition of THF (20 mL), H_2O (5 mL) and the above yellow oil. The mixture was stirred at 80 ℃. After the reaction was complete (monitored by TLC), the mixture was cooled to room temperature. The reaction was then quenched by adding $NaHCO_3$ solution (aq. 2 M, 20 mL). The mixture was extracted with ethyl acetate (20 mL × 3). The combined organic phases were washed with brine and dried over anhydrous Na_2SO_4. After filtration, the solvent was removed under reduced pressure. The residue was purified by silica gel column chromatography

(hexane/ethyl acetate = 10/1, v/v) to afford product **1**.

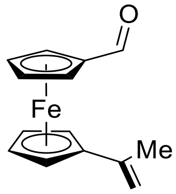

1j. Dark red oil (0.60 g, 59% yield, 4 mmol scale).

Analytical data for **1j**:

^{1}H NMR (400 MHz, $CDCl_3$) δ 9.88 (s, 1H), 5.17 (s, 1H), 4.93-4.92 (m, 1H), 4.71-4.69 (m, 2H), 4.53-4.52 (m, 2H), 4.49-4.47 (m, 2H), 4.33-4.31 (m, 2H), 2.01 (s, 3H).

^{13}C NMR (101 MHz, $CDCl_3$) δ 193.5, 139.6, 110.3, 88.4, 79.7, 74.4, 70.6, 70.1, 67.2, 21.3.

IR (neat): ν_{max} (cm^{-1}) = 2926, 2868, 1722, 1680, 1663, 1455, 1370, 1334, 1245, 1202, 1034, 989, 906, 829, 743, 699, 626.

HRMS (ESI) calcd for $C_{14}H_{14}[^{56}Fe]O$ $[M]^+$: 254.0389. Found: 254.0390.

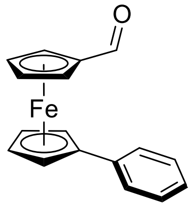

1k. Foamy dark red solid (0.42 g, 72% yield, 2 mmol scale).

Analytical data for **1k**:

^{1}H NMR (400 MHz, $CDCl_3$) δ 9.81 (s, 1H), 7.45 (d, J = 6.8 Hz, 2H), 7.34-7.30 (m, 2H), 7.25-7.22 (m, 1H), 4.75 (t, J = 2.0 Hz, 2H), 4.64 (t, J = 2.0 Hz, 2H), 4.45 (t, J = 2.0 Hz, 2H), 4.43 (t, J = 2.0 Hz, 2H).

^{13}C NMR (101 MHz, $CDCl_3$) δ 193.6, 137.0, 128.6, 126.8, 126.1, 87.4, 79.8, 74.9, 70.9, 70.4, 67.7.

IR (neat): ν_{max} (cm^{-1}) = 2973, 2904, 1682, 1528, 1458, 1408, 1373, 1336, 1290, 1248, 1184, 1044, 1032, 887, 826, 791, 739.

HRMS (ESI) calcd for $C_{17}H_{14}[^{56}Fe]O$ $[M]^+$: 290.0389. Found: 290.0391.

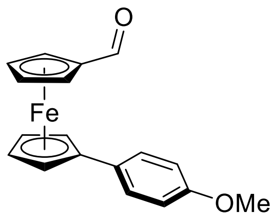

1l. Foamy dark red solid (0.49 g, 77% yield, 2 mmol scale).

Analytical data for **1l**:

^{1}H NMR (400 MHz, $CDCl_3$) δ 9.81 (s, 1H), 7.38 (d, J = 8.8 Hz, 2H), 6.86 (d, J = 8.8 Hz, 2H), 4.67 (t, J = 2.0 Hz, 2H), 4.63 (t, J = 2.0 Hz, 2H), 4.45-4.44 (m, 2H), 4.39-4.37 (m, 2H), 3.82 (s, 3H).

^{13}C NMR (101 MHz, $CDCl_3$) δ 193.7, 158.6, 129.0, 127.1, 114.1, 87.8, 79.7, 74.8, 70.9, 70.0, 67.3, 55.3.

IR (neat): ν_{max} (cm^{-1}) = 1683, 1609, 1529, 1460, 1443, 1370, 1290, 1249, 1211, 1184, 1114, 1087, 1028, 887, 822, 792, 741, 647.

HRMS (ESI) calcd for $C_{18}H_{16}[^{56}Fe]O_2Na$ $[M+Na]^+$: 343.0392. Found: 343.0395.

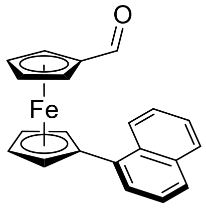

1m. Foamy dark red solid (0.41 g, 60% yield, 2 mmol scale).

Analytical data for **1m**:

^{1}H NMR (400 MHz, $CDCl_3$) δ 9.99 (s, 1H), 8.37-8.33 (m, 1H), 7.89-7.79 (m, 3H), 7.52-7.45 (m, 3H), 4.81 (t, J = 2.0 Hz, 2H), 4.75 (t, J = 2.0 Hz, 2H), 4.60-4.58 (m, 2H), 4.53-4.51 (m, 2H).

^{13}C NMR (101 MHz, $CDCl_3$) δ 193.6, 134.0, 133.8, 131.7, 128.6, 128.1, 127.7, 125.8, 125.7, 125.4, 125.2, 89.3, 79.7, 75.2, 71.7, 71.0, 69.6.

IR (neat): ν_{max} (cm^{-1}) = 2983, 2903, 1680, 1454, 1392, 1246, 1052, 893, 833, 802, 778, 737.

HRMS (ESI) calcd for $C_{21}H_{16}[^{56}Fe]ONa$ $[M+Na]^+$: 363.0443. Found: 363.0436.

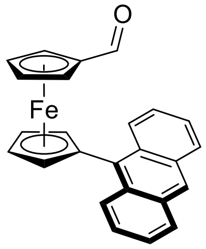

1n. Foamy dark red solid (0.38 g, 49% yield, 2.0 mmol scale).

Analytical data for **1n**:

^{1}H NMR (400 MHz, $CDCl_3$) δ 9.99 (s, 1H), 8.98 (d, J = 8.8 Hz, 2H), 8.45 (s, 1H), 8.01 (d, J = 8.0 Hz, 2H), 7.53-7.43 (m, 4H), 4.89-4.87 (m, 4H), 4.70 (t, J = 2.0 Hz, 2H), 4.60 (t, J = 2.0 Hz, 2H).

^{13}C NMR (101 MHz, $CDCl_3$) δ 193.8, 131.6, 130.5, 129.6, 128.8, 127.6, 127.1, 125.0, 124.8, 86.7, 79.5, 75.6, 74.4, 70.9, 69.3.

IR (neat): ν_{max} (cm^{-1}) = 1682, 1609, 1529, 1460, 1444, 1370, 1337, 1290, 1248, 1184, 1113, 1086, 1029, 887, 824, 792, 740, 651.

HRMS (ESI) calcd for $C_{25}H_{18}[^{56}Fe]ONa$ $[M+Na]^+$: 413.0599. Found: 413.0597.

General procedure for enantioselective Rh(I)-catalyzed C-H arylation of ferroceneformaldehydes

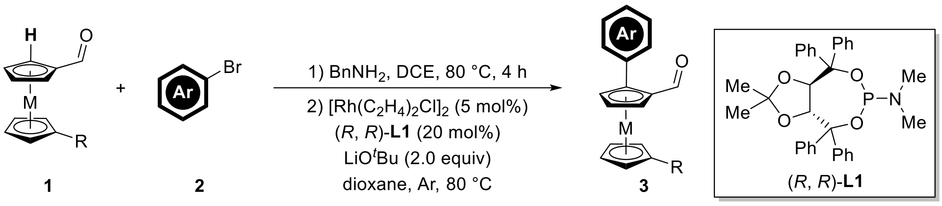

A 10 mL Schlenk tube was charged with ferrocene substrate **1** (0.2 mmol, 1.0 equiv). Then the flask was evacuated and backfilled with argon for 3 times, and followed by addition of DCE (1.0 mL) and $BnNH_2$ (24 μL, 0.22 mmol, 1.1 equiv). The mixture was stirred at 80 °C in 4 h. The solvent is drained directly through the oil pump. Next, LiO^{t}Bu (32.0 mg, 0.4 mmol), (*R*, *R*)-**L1** (21.6 mg, 0.04 mmol), $[Rh(C_2H_4)_2Cl]_2$ (3.9 mg, 0.01 mmol) and aryl bromide **2** (0.4 mmol, 2.0 equiv) were added to the Schlenk tube. The mixture was stirred at 80 °C. After the reaction was complete (monitored by TLC), the mixture was cooled to room temperature. The mixture was diluted with ethyl acetate (5 mL × 3), and filtered through a pad of celite. The filtrate was evaporated under reduced pressure. The crude mixture was purified by silica gel column chromatography (petroleum ether/ethyl acetate = 20/1).

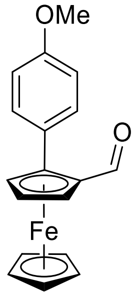

3aa. Dark red solid (48.5 mg, 76% yield, 99% ee).

Analytical data for **3aa**:

m.p.: 109.3-110.8 °C.

$[\alpha]_D^{26}$ = -214.8 (c = 0.1 Acetone).

^{1}H NMR (400 MHz, $CDCl_3$) δ 10.19 (s, 1H), 7.45 (d, J = 8.4 Hz, 2H), 6.90 (d, J = 8.8 Hz, 2H), 4.96 (dd, J = 2.8, 1.6 Hz, 1H), 4.78 (dd, J = 2.4, 1.2 Hz, 1H), 4.68 (t, J = 2.4 Hz, 1H), 4.23 (s, 5H), 3.84 (s, 3H).

^{13}C NMR (101 MHz, $CDCl_3$) δ 193.2, 159.0, 130.7, 127.8, 113.8, 92.7, 76.5, 74.4, 71.8, 71.0, 68.2, 55.3.

IR (neat): ν_{max} (cm^{-1}) = 2932, 2835, 2762, 1661, 1608, 1573, 1519, 1433, 1411, 1397, 1357, 1327, 1293, 1266, 1244, 1217, 1175, 1129, 1105, 1053, 1029, 1001, 961, 825, 797, 760, 728, 666, 630.

HRMS (ESI) calcd for $C_{18}H_{16}[^{56}Fe]O_2$ $[M]^+$: 320.0494. Found: 320.0490.

HPLC The enantiomeric excess was determined by Daicel Chiralcel OD-H (0.46 cm × 25 cm), Hexanes / IPA = 90 / 10, 1.0 mL/min, λ = 254 nm, t (major) = 12.38 min, t (minor) = 14.44 min.

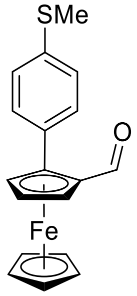

3ab. Foamy dark red solid (47.8 mg, 71% yield, >99% ee).

Analytical data for **3ab**:

$[\alpha]_D^{26}$ = -90.6 (c = 0.1 Acetone).

^{1}H NMR (400 MHz, $CDCl_3$) δ 10.19 (s, 1H), 7.45 (d, J = 8.4 Hz, 2H), 7.24 (d, J = 8.4 Hz, 2H), 4.99 (dd, J = 2.4, 1.2 Hz, 1H), 4.83-4.81 (m, 1H), 4.70 (t, J = 2.8 Hz, 1H), 4.24 (s, 5H), 2.52 (s, 3H).

^{13}C NMR (101 MHz, $CDCl_3$) δ 192.9, 137.7, 132.7, 130.0, 126.3, 92.0, 76.5, 74.8, 72.0, 71.1, 68.7, 15.7.

IR (neat): ν_{max} (cm^{-1}) = 3085, 2985, 2919, 2848, 2765, 1661, 1596, 1504, 1429, 1401, 1352, 1326, 1301, 1279, 1256, 1217, 1106, 1093, 1052, 1028, 1001, 960, 908, 818, 774, 727, 664, 647.

HRMS (ESI) calcd for $C_{18}H_{16}[^{56}Fe]OSNa$ $[M+Na]^+$: 359.0164. Found: 359.0166.

HPLC The enantiomeric excess was determined by Daicel Chiralcel OD-H (0.46 cm × 25 cm), Hexanes / IPA = 90 / 10, 1.0 mL/min, λ = 254 nm, t (major) = 12.63 min, t (minor) = 14.71 min.

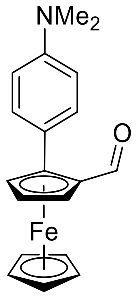

3ac. Foamy dark red solid (46.6 mg, 70% yield, 99% ee).

Analytical data for **3ac**:

$[\alpha]_D^{26}$ = -305.8 (c = 0.1 Acetone).

^{1}H NMR (400 MHz, $CDCl_3$) δ 10.23 (s, 1H), 7.40 (d, *J* = 8.8 Hz, 2H), 6.72 (d, *J* = 8.8 Hz, 2H), 4.95 (dd, *J* = 2.8, 1.2 Hz, 1H), 4.76 (dd, *J* = 2.8, 1.6 Hz, 1H), 4.66 (t, *J* = 2.4 Hz, 1H), 4.23 (s, 5H), 3.00 (s, 6H).

^{13}C NMR (101 MHz, $CDCl_3$) δ 193.6, 149.8, 130.4, 122.9, 112.1, 93.8, 76.6, 73.8, 71.6, 70.9, 67.6, 40.5.

IR (neat): ν_{max} (cm^{-1}) = 2885, 2849, 2802, 1660, 1611, 1528, 1480, 1436, 1410, 1396, 1350, 1330, 1297, 1266, 1222, 1195, 1168, 1129, 1115, 1106, 1057, 1028, 1001, 944, 908, 816, 778, 726, 667, 646, 631.

HRMS (ESI) calcd for $C_{19}H_{20}[^{56}Fe]NO$ $[M+H]^+$: 334.0889. Found: 334.0893.

HPLC The enantiomeric excess was determined by Daicel Chiralcel OD-H (0.46 cm × 25 cm), Hexanes / IPA = 90 / 10, 1.0 mL/min, λ = 254 nm, t (major) = 12.10 min, t (minor) = 13.83 min.

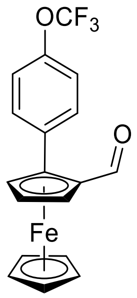

3ad. Foamy dark red solid (47.2 mg, 63% yield, 98% ee).

Analytical data for **3ad**:

$[\alpha]_D^{18}$ = -66.6 (c = 0.1 Acetone).

^{1}H NMR (400 MHz, $CDCl_3$) δ 10.16 (s, 1H), 7.56 (d, *J* = 8.8 Hz, 2H), 7.21 (d, *J* = 8.0 Hz, 2H), 5.00 (dd, *J* = 2.8, 1.6 Hz, 1H), 4.83 (dd, *J* = 2.4, 1.2 Hz, 1H), 4.73 (t, *J* = 2.8 Hz, 1H), 4.26 (s, 5H).

^{13}C NMR (101 MHz, $CDCl_3$) δ 192.6, 148.34, 148.32, 135.0, 130.9, 120.8, 120.4 (*J* = 258.3 Hz), 90.7, 76.5, 75.3, 72.2, 71.2, 69.4.

^{19}F NMR (377 MHz, $CDCl_3$) δ -57.8 (s).

IR (neat): ν_{max} (cm^{-1}) = 3090, 2924, 2852, 1666, 1516, 1434, 1412, 1250, 1203, 1154, 1105, 1054, 1028, 1018, 1002, 963, 921, 823, 808, 769, 735, 685, 652.

HRMS (ESI) calcd for $C_{18}H_{14}[^{56}Fe]O_2F_3$ $[M]^+$: 375.0290. Found: 375.0289.

HPLC The enantiomeric excess was determined by Daicel Chiralcel OD-H (0.46 cm × 25 cm), Hexanes / IPA = 90 / 10, 1.0 mL/min, λ = 254 nm, t (major) = 8.20 min, t (minor) = 9.75 min.

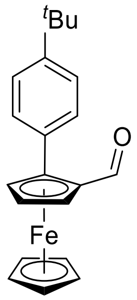

3ae. Dark red oil (55.4 mg, 80% yield, >99% ee).

Analytical data for **3ae**:

$[\alpha]_D^{26}$ = -249.3 (c = 0.1 Acetone).

^{1}H NMR (400 MHz, $CDCl_3$) δ 10.22 (s, 1H), 7.46 (d, *J* = 8.4 Hz, 2H), 7.38 (d, *J* = 8.4 Hz, 2H), 4.99 (dd, *J* = 2.8, 1.6 Hz, 1H), 4.81 (dd, *J* = 2.8, 1.6 Hz, 1H), 4.69 (t, *J* = 2.8 Hz, 1H), 4.25 (s, 5H), 1.36 (s, 9H).

^{13}C NMR (101 MHz, $CDCl_3$) δ 193.4, 150.4, 132.8, 129.4, 125.3, 92.7, 76.5, 74.8, 71.9, 71.0, 68.2, 34.6, 31.3.

IR (neat): ν_{max} (cm^{-1}) = 2960, 2903, 2866, 1665, 1522, 1462, 1430, 1409, 1362, 1328, 1309, 1288, 1266, 1218, 1202, 1106, 1052, 1027, 1001, 909, 822, 774, 729, 666.

HRMS (ESI) calcd for $C_{21}H_{23}[^{56}Fe]O$ $[M+H]^+$: 347.1093. Found: 347.1094.

HPLC The enantiomeric excess was determined by Daicel Chiralcel OD-H (0.46 cm × 25 cm), Hexanes / IPA = 98 / 02, 1.0 mL/min, λ = 254 nm, t (major) = 11.35 min, t (minor) = 12.88 min.

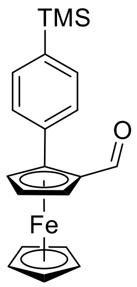

3af. Dark red oil (50.7 mg, 70% yield, 98% ee).

Analytical data for **3af**:

$[\alpha]_D^{26}$ = -201.1 (c = 0.1 Acetone).

^{1}H NMR (400 MHz, $CDCl_3$) δ 10.22 (s, 1H), 7.51 (s, 4H), 5.01 (dd, *J* = 2.8, 1.6 Hz, 1H), 4.85 (dd, *J* = 2.8, 1.6 Hz, 1H), 4.71 (t, *J* = 2.8 Hz, 1H), 4.26 (s, 5H), 0.31 (s, 9H).

^{13}C NMR (101 MHz, $CDCl_3$) δ 193.2, 139.6, 136.5, 133.3, 129.1, 92.6, 76.5, 75.2, 72.0, 71.1, 68.5, -1.2.

IR (neat): ν_{max} (cm^{-1}) = 2953, 1666, 1599, 1429, 1411, 1397, 1327, 1307, 1276, 1248, 1217, 1107, 1050, 1002, 909, 838, 820, 758, 725, 663, 647, 624.

HRMS (ESI) calcd for $C_{20}H_{23}[^{56}Fe]OSi$ $[M+H]^+$: 363.0862. Found: 363.0871.

HPLC The enantiomeric excess was determined by Daicel Chiralpak AD-3 (0.46 cm × 25 cm), Hexanes / IPA = 90 / 10, 0.7 mL/min, λ = 230 nm, t (minor) = 6.37 min, t (major) = 6.74 min.

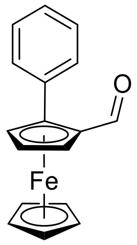

3ag. Foamy dark red solid (40.7 mg, 70% yield, 98% ee).

Analytical data for **3ag**:

$[\alpha]_D^{26}$ = -117.9 (*c* = 0.1, Acetone).

^{1}H NMR (400 MHz, CD_2Cl_2) δ 10.18 (s, 1H), 7.58-7.54 (m, 2H), 7.40-7.29 (m, 3H), 4.97 (dd, J = 2.8, 1.6 Hz, 1H), 4.87 (dd, J = 2.8, 1.6 Hz, 1H), 4.73 (td, J = 2.8, 0.8 Hz, 1H), 4.26 (s, 5H).

^{13}C NMR (101 MHz, CD_2Cl_2) δ 193.5, 137.0, 130.6, 129.0, 128.0, 93.2, 77.5, 76.0, 72.8, 71.9, 69.5.

IR (neat): ν_{max} (cm^{-1}) = 2983, 2903, 1661, 1613, 1532, 1440, 1401, 1354, 1251, 1226, 1200, 1169, 1060, 893, 813.

HRMS (ESI) calcd for $C_{17}H_{14}[^{56}Fe]ONa$ $[M+Na]^+$: 313.0286. Found: 313.0286.

HPLC The enantiomeric excess was determined by Daicel Chiralpak AD-H (0.46 cm × 25 cm), Hexanes / IPA = 95 / 05, 1.0 mL/min, λ = 254 nm, t (major) = 6.94 min, t (minor) = 7.47 min.

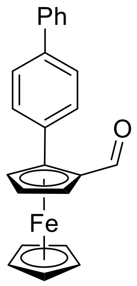

3ah. Foamy dark red solid (54.9 mg, 75% yield, >99% ee).

Analytical data for **3ah**:

$[\alpha]_D^{26}$ = -186.9 (c = 0.1, Acetone).

^{1}H NMR (400 MHz, $CDCl_3$) δ 10.26 (s, 1H), 7.65-7.59 (m, 6H), 7.49-7.44 (m, 2H), 7.40-7.35 (m, 1H), 5.03 (dd, J = 2.4, 1.2 Hz, 1H), 4.89 (dd, J = 2.4, 1.2 Hz, 1H), 4.74 (t, J = 2.4 Hz, 1H), 4.28 (s, 5H).

^{13}C NMR (101 MHz, $CDCl_3$) δ 193.1, 140.5, 140.2, 135.1, 130.1, 128.9, 127.5, 127.0, 127.0, 92.1, 75.1, 72.1, 71.2, 68.7.

IR (neat): ν_{max} (cm^{-1}) = 2972, 2905, 1663, 1520, 1449, 1431, 1412, 1325, 1297, 1265, 1242, 1175, 1105, 1062, 1027, 865, 832, 798, 757.

HRMS (ESI) calcd for $C_{23}H_{18}[^{56}Fe]ONa$ $[M+Na]^+$: 389.0599. Found: 389.0592.

HPLC The enantiomeric excess was determined by Daicel Chiralpak AD-H (0.46 cm × 25 cm), Hexanes / IPA = 95 / 05, 1.0 mL/min, λ = 254 nm, t (major) = 18.12 min, t (minor) = 22.40 min.

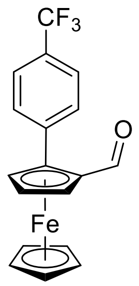

3ai. Foamy dark red solid (49.3 mg, 69% yield, >99% ee).

Analytical data for **3ai**:

$[\alpha]_D^{18}$ = -145.2 (c = 0.1 Acetone).

^{1}H NMR (400 MHz, $CDCl_3$) δ 10.17 (s, 1H), 7.66 (d, J = 8.0 Hz, 2H), 7.61 (d, J = 8.0 Hz, 2H), 5.03 (dd, J = 2.8, 1.6 Hz, 1H), 4.89 (dd, J = 2.8, 1.6 Hz, 1H), 4.76 (t, J = 24 Hz, 1H), 4.27 (s, 5H).

^{13}C NMR (101 MHz, $CDCl_3$) δ 192.4, 140.6, 129.8, 129.3 (J = 32.7 Hz), 125.2 (J = 3.8 Hz), 124.1 (J = 273.1 Hz), 90.1, 76.4, 75.7, 72.5, 71.4, 70.0.

^{19}F NMR (377 MHz, $CDCl_3$) δ -62.5 (s).

IR (neat): ν_{max} (cm^{-1}) = 3091, 2926, 2852, 2767, 1667, 1615, 1574, 1527, 1433, 1411, 1320, 1267, 1218, 1162, 1119, 1104, 1068, 1029, 1016, 1003, 962, 909, 842, 824, 784, 759, 731, 692, 683, 662, 630, 609.

HRMS (ESI) calcd for $C_{18}H_{14}[^{56}Fe]OF_3$ $[M+H]^+$: 359.0341. Found: 359.0338.

HPLC The enantiomeric excess was determined by Daicel Chiralcel OD-H (0.46 cm × 25 cm), Hexanes / IPA = 90 / 10, 1.0 mL/min, λ = 254 nm, t (major) = 9.72 min, t (minor) = 11.63 min.

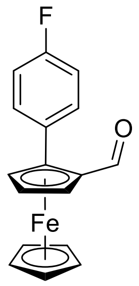

3aj. Foamy dark red solid (43.2 mg, 70% yield, 99% ee).

Analytical data for **3aj**:

$[\alpha]_D^{18}$ = -239.8 (c = 0.1 Acetone).

^{1}H NMR (400 MHz, $CDCl_3$) δ 10.16 (s, 1H), 7.50 (dd, J = 8.4, 5.2 Hz, 2H), 7.08-7.03 (m, 2H), 4.98 (dd, J = 2.8, 1.6 Hz, 1H), 4.80 (dd, J = 2.8, 1.6 Hz, 1H), 4.71 (t, J = 2.8 Hz, 1H), 4.25 (s, 5H).

^{13}C NMR (101 MHz, $CDCl_3$) δ 192.8, 162.1 (J = 248.0 Hz), 131.9 (J = 3.2 Hz), 131.2 (J = 8.2 Hz), 115.3 (J = 21.6 Hz), 91.5, 76.5, 75.0, 72.0, 71.1, 68.9.

^{19}F NMR (377 MHz, $CDCl_3$) δ -114.6 (m).

IR (neat): ν_{max} (cm^{-1}) = 3084, 2849, 2762, 1663, 1601, 1517, 1433, 1409, 1359, 1326, 1288, 1263, 1218, 1157, 1106, 1094, 1052, 1028, 1001, 909, 827, 813, 763, 729, 664.

HRMS (ESI) calcd for $C_{17}H_{14}[^{56}Fe]OF$ $[M+H]^+$: 309.0373. Found: 309.0370.

HPLC The enantiomeric excess was determined by Daicel Chiralcel OJ-H (0.46 cm × 25 cm), Hexanes / IPA = 90 / 10, 1.0 mL/min, λ = 254 nm, t (minor) = 15.64 min, t (major) = 18.23 min.

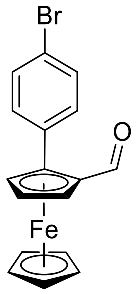

3ak. Foamy dark red solid (53.1 mg, 72% yield, 98% ee).

Analytical data for **3ak**:

$[\alpha]_D^{27}$ = -67.9 (c = 0.1, Acetone).

^{1}H NMR (400 MHz, $CDCl_3$) δ 10.15 (s, 1H), 7.48 (d, J = 8.4 Hz, 2H), 7.41 (d, J = 8.8 Hz, 2H), 5.00 (dd, J = 2.8, 1.2 Hz, 1H), 4.83 (dd, J = 2.8, 1.2 Hz, 1H), 4.73 (d, J = 2.8 Hz, 1H), 4.24 (s, 5H).

^{13}C NMR (101 MHz, $CDCl_3$) δ 192.6, 135.3, 131.4, 131.2, 121.2, 91.0, 76.4, 75.1, 72.2, 71.2, 69.3.

IR (neat): ν_{max} (cm^{-1}) = 2967, 2905, 1663, 1520, 1448, 1430, 1411, 1325, 1296, 1262, 1242, 1175, 1071, 1026, 865, 831, 798, 757.

HRMS (ESI) calcd for $C_{17}H_{13}[^{56}Fe]OBrNa$ $[M+Na]^+$: 390.9391. Found: 390.9384.

HPLC The enantiomeric excess was determined by Daicel Chiralcel OD-H (0.46 cm × 25 cm), Hexanes / IPA = 80 / 20, 0.7 mL/min, λ = 254 nm, t (major) = 6.03 min, t (minor) = 6.75 min.

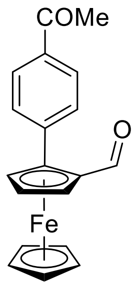

3al. Foamy dark red solid (33.2 mg, 50% yield, 97% ee).

Analytical data for **3al**:

$[\alpha]_D^{26}$ = -70.5 (c = 0.1 Acetone).

^{1}H NMR (400 MHz, $CDCl_3$) δ 10.19 (s, 1H), 7.95 (d, J = 8.4 Hz, 2H), 7.63 (d, J = 8.4 Hz, 2H), 5.05 (dd, J = 2.7, 1.5 Hz, 1H), 4.93 (dd, J = 2.6, 1.5 Hz, 1H), 4.78 (t, J = 2.7 Hz, 1H), 4.26 (s, 5H), 2.63 (s, 3H).

^{13}C NMR (101 MHz, $CDCl_3$) δ 197.5, 192.4, 142.0, 135.8, 129.7, 128.3, 90.3, 76.5, 75.8, 72.6, 71.4, 69.9, 26.6.

IR (neat): ν_{max} (cm^{-1}) = 2988, 2921, 2848, 1664, 1602, 1562, 1425, 1412, 1394, 1359, 1325, 1298, 1270, 1214, 1189, 1106, 1074, 1048, 1023, 1006, 990, 961, 909, 852, 841, 823, 777, 732, 699, 662, 602.

HRMS (ESI) calcd for $C_{19}H_{17}[^{56}Fe]O_2$ $[M+H]^+$: 333.0573. Found: 333.0574.

HPLC The enantiomeric excess was determined by Daicel Chiralcel IG (0.46 cm × 25 cm), Hexanes / IPA = 85 / 15, 1.0 mL/min, λ = 254 nm, t (major) = 49.10 min, t (minor) = 55.48 min.

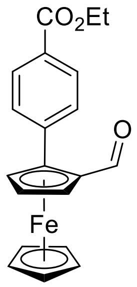

3am. Foamy dark red solid (32.5 mg, 45% yield, 96% ee).

Analytical data for **3am**:

$[\alpha]_D^{26}$ = -79.8 (c = 0.1, Acetone).

^{1}H NMR (400 MHz, $CDCl_3$) δ 10.19 (s, 1H), 8.03 (d, J = 8.4 Hz, 2H), 7.60 (d, J = 8.0 Hz, 2H), 5.04 (t, J = 2.0 Hz, 1H), 4.92-4.91 (m, 1H), 4.77 (t, J = 2.8 Hz, 1H), 4.40 (q, J = 7.2 Hz, 2H), 4.26 (s, 5H), 1.41 (t, J = 7.2 Hz, 3H).

^{13}C NMR (101 MHz, $CDCl_3$) δ 192.5, 166.3, 141.6, 129.5, 129.5, 129.3, 90.7, 75.7, 72.5, 71.4, 69.6, 61.0, 14.4.

IR (neat): ν_{max} (cm^{-1}) = 2983, 2904, 1667, 1603, 1426, 1361, 1269, 1215, 1106, 1070, 1008, 962, 849, 826, 735.

HRMS (ESI) calcd for $C_{20}H_{18}[^{56}Fe]O_3Na$ $[M+Na]^+$: 385.0498. Found: 385.0491.

HPLC The enantiomeric excess was determined by Daicel Chiralpak IG (0.46 cm × 25 cm), Hexanes / IPA = 70 / 30, 0.7 mL/min, λ = 254 nm, t (major) = 27.66 min, t (minor) = 29.55 min.

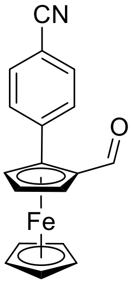

3an. Foamy dark red solid (35.9 mg, 57% yield, 96% ee).

Analytical data for **3an**:

$[\alpha]_D^{27}$ = -44.0 (c = 0.1 Acetone).

^{1}H NMR (400 MHz, $CDCl_3$) δ 10.14 (s, 1H), 7.68-7.61 (m, 4H), 5.04 (dd, *J* = 2.8, 1.6 Hz, 1H), 4.91 (dd, *J* = 2.8, 1.6 Hz, 1H), 4.79 (t, *J* = 2.4 Hz, 1H), 4.26 (s, 5H).

^{13}C NMR (101 MHz, $CDCl_3$) δ 192.0, 142.3, 131.9, 130.1, 118.8, 110.6, 89.1, 76.3, 76.0, 72.8, 71.5, 71.0.

IR (neat): ν_{max} (cm^{-1}) = 2970, 2920, 2848, 2226, 1665, 1603, 1562, 1516, 1445, 1427, 1411, 1394, 1360, 1325, 1298, 1270, 1257, 1214, 1189, 1107, 1049, 1023, 1003, 961, 841, 823, 777, 731, 699, 663, 602.

HRMS (ESI) calcd for $C_{18}H_{14}[^{56}Fe]NO$ $[M+H]^+$: 316.0419. Found: 316.0410.

HPLC The enantiomeric excess was determined by Daicel Chiralcel OD-H (0.46 cm × 25 cm), Hexanes / IPA = 85 / 15, 1.0 mL/min, λ = 254 nm, t (major) = 18.84 min, t (minor) = 21.51 min.

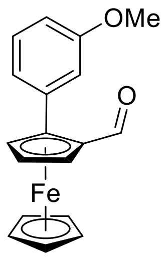

3ao. Foamy dark red solid (48.0 mg, 75% yield, >99% ee).

Analytical data for **3ao**:

$[\alpha]_D^{26}$ = -174.5 (c = 0.1 Acetone).

^{1}H NMR (400 MHz, $CDCl_3$) δ 10.19 (s, 1H), 7.23 (d, *J* = 7.6 Hz, 1H), 7.09-7.04 (m, 2H), 6.83 (ddd, *J* = 8.4, 2.4, 0.8 Hz, 1H), 4.96 (dd, *J* = 2.8, 1.6 Hz, 1H), 4.80 (dd, *J* = 2.8, 1.6 Hz, 1H), 4.67 (t, *J* = 2.8 Hz, 1H), 4.22 (s, 5H), 3.81 (s, 3H).

^{13}C NMR (101 MHz, $CDCl_3$) δ 193.1, 159.4, 137.5, 129.3, 122.3, 115.8, 112.5, 92.5, 76.6, 75.1, 72.9, 72.0, 71.2, 68.5, 55.3.

IR (neat): ν_{max} (cm^{-1}) = 2937, 2834, 1664, 1599, 1577, 1498, 1462, 1435, 1411, 1325, 1279, 1267, 1235, 1205, 1178, 1106, 1088, 1041, 1001, 979, 908, 874, 857, 821, 782, 760, 727, 698, 674, 647.

HRMS (ESI) calcd for $C_{18}H_{17}[^{56}Fe]O_2$ $[M+H]^+$: 321.0573. Found: 321.0572.

HPLC The enantiomeric excess was determined by Daicel Chiralcel OD-H (0.46 cm × 25 cm), Hexanes / IPA = 95 / 05, 1.0 mL/min, λ = 254 nm, t (major) = 18.04 min, t (minor) = 20.52 min.

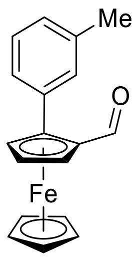

3ap. Foamy dark red solid (49.9 mg, 82% yield, 99% ee).

Analytical data for **3ap**:

$[\alpha]_D^{25}$ = -302.9 (*c* = 0.1, Acetone).

^{1}H NMR (400 MHz, $CDCl_3$) δ 10.20 (s, 1H), 7.33 (d, *J* = 7.6 Hz, 1H), 7.29 (s, 1H), 7.23 (d, *J* = 8.4 Hz, 1H), 7.12 (d, *J* = 7.6 Hz, 1H), 4.98 (dd, *J* = 2.8, 1.6 Hz, 1H), 4.81 (dd, *J* = 2.8, 1.6 Hz, 1H), 4.68 (t, *J* = 2.8 Hz, 1H), 4.23 (s, 5H), 2.38 (s, 3H).

^{13}C NMR (101 MHz, $CDCl_3$) δ 193.3, 138.0, 135.9, 130.4, 128.3, 128.1, 126.9, 92.9, 76.5, 75.0, 71.9, 71.1, 68.2, 21.4.

IR (neat): ν_{max} (cm^{-1}) = 2974, 2920, 1669, 1603, 1581, 1497, 1456, 1431, 1410, 1328, 1288, 1263, 1229, 1190, 1105, 1058, 1003, 908, 823, 789, 760, 731, 704, 656.

HRMS (ESI) calcd for $C_{18}H_{16}[^{56}Fe]ONa$ $[M+Na]^+$: 327.0443. Found: 327.0446.

HPLC The enantiomeric excess was determined by Daicel Chiralpak AD-H (0.46 cm × 25 cm), Hexanes / IPA = 95 / 05, 1.0 mL/min, λ = 254 nm, t (major) = 6.94 min, t (minor) = 7.46 min.

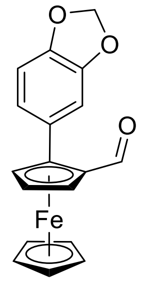

3aq. Foamy dark red solid (52.1 mg, 78% yield, >99% ee).

Analytical data for **3aq**:

$[\alpha]_D^{26}$ = -217.5 (c = 0.1 Acetone).

^{1}H NMR (400 MHz, $CDCl_3$) δ 10.18 (s, 1H), 7.06 (s, 1H), 6.95 (d, J = 7.2 Hz, 1H), 6.78 (d, J = 8.0 Hz, 1H), 6.00 (s, 2H), 4.97-4.93 (m, 1H), 4.77-4.75 (m, 1H), 4.67 (t, J = 2.8 Hz, 1H), 4.24 (s, 5H).

^{13}C NMR (101 MHz, $CDCl_3$) δ 193.0, 147.6, 147.0, 129.6, 123.2, 110.0, 108.1, 101.2, 92.8, 76.6, 74.6, 71.8, 71.1, 68.4.

IR (neat): ν_{max} (cm^{-1}) = 2888, 2773, 1664, 1607, 1507, 1457, 1432, 1411, 1341, 1319, 1233, 1210, 1157, 1105, 1035, 1001, 932, 909, 886, 851, 811, 767, 726, 680, 671, 665, 649, 626.

HRMS (ESI) calcd for $C_{18}H_{15}[^{56}Fe]O_3$ $[M+H]^+$: 335.0365. Found: 335.0363.

HPLC The enantiomeric excess was determined by Daicel Chiralcel OD-H (0.46 cm × 25 cm), Hexanes / IPA = 90 / 10, 1.0 mL/min, λ = 254 nm, t (major) = 15.99 min, t (minor) = 18.71 min.

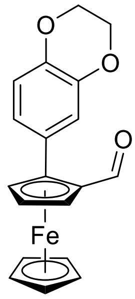

3ar. Foamy dark red solid (55.7 mg, 80% yield, >99% ee).

Analytical data for **3ar**:

$[\alpha]_D^{26}$ = -143.5 (c = 0.1 Acetone).

^{1}H NMR (400 MHz, $CDCl_3$) δ 10.20 (s, 1H), 7.06 (d, J = 2.0 Hz, 1H), 6.97 (dd, J = 8.4, 2.0 Hz, 1H), 6.84 (d, J = 8.4 Hz, 1H), 4.96 (dd, J = 2.8, 1.6 Hz, 1H), 4.76 (dd, J = 2.8, 1.6 Hz, 1H), 4.66 (t, J = 2.8 Hz, 1H), 4.29 (s, 4H), 4.24 (s, 5H).

^{13}C NMR (101 MHz, $CDCl_3$) δ 193.2, 143.2, 143.0, 129.0, 122.9, 118.4, 117.1, 92.5, 76.5, 74.5, 71.7, 71.0, 68.2, 64.4.

IR (neat): ν_{max} (cm^{-1}) = 2978, 2928, 2874, 1663, 1580, 1516, 1449, 1432, 1411, 1324, 1305, 1281, 1246, 1214, 1184, 1126, 1106, 1067, 1029, 1001, 929, 908, 893, 877, 846, 816, 766, 726, 681, 671, 665, 647, 628.

HRMS (ESI) calcd for $C_{19}H_{17}[^{56}Fe]O_3$ $[M+H]^+$: 349.0522. Found: 349.0524.

HPLC The enantiomeric excess was determined by Daicel Chiralcel OD-H (0.46 cm × 25 cm), Hexanes / IPA = 95 / 05, 1.0 mL/min, λ = 254 nm, t (major) = 39.11 min, t (minor) = 43.18 min.

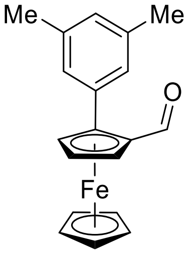

3as. Foamy dark red solid (49.7 mg, 78% yield, >99% ee).

Analytical data for **3as**:

$[\alpha]_D^{26}$ = -330.0 (c = 0.1 Acetone).

^{1}H NMR (400 MHz, $CDCl_3$) δ 10.22 (s, 1H), 7.13 (s, 2H), 6.97 (s, 1H), 4.99 (dd, J = 2.8, 1.6 Hz, 1H), 4.81 (dd, J = 2.8, 1.6 Hz, 1H), 4.69 (t, J = 2.8 Hz, 1H), 4.25 (s, 5H), 2.36 (s, 6H).

^{13}C NMR (101 MHz, $CDCl_3$) δ 193.4, 137.9, 135.7, 129.1, 127.6, 93.2, 76.5, 74.9, 71.9, 71.1, 68.0, 21.3.

IR (neat): ν_{max} (cm^{-1}) = 2916, 2857, 1664, 1599, 1486, 1442, 1411, 1377, 1331, 1293, 1245, 1198, 1107, 1033, 1001, 908, 849, 822, 728, 703, 667, 663, 646.

HRMS (ESI) calcd for $C_{19}H_{19}[^{56}Fe]O$ $[M+H]^+$: 319.0780. Found: 319.0774.

HPLC The enantiomeric excess was determined by Daicel Chiralcel OD-H (0.46 cm × 25 cm), Hexanes / IPA = 98 / 02, 0.5 mL/min, λ = 226 nm, t (major) = 20.61 min, t (minor) = 23.41 min.

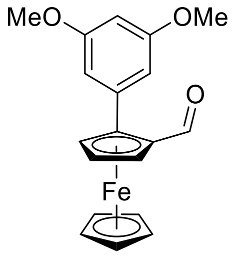

3at. Foamy dark red solid (58.2 mg, 83% yield, >99% ee).

Analytical data for **3at**:

$[\alpha]_D^{25}$ = -269.1 (*c* = 0.1, Acetone).

^{1}H NMR (400 MHz, $CDCl_3$) δ 10.24 (s, 1H), 6.68 (d, *J* = 2.0 Hz, 2H), 6.43 (d, *J* = 2.4 Hz, 1H), 4.99 (dd, *J* = 2.8, 1.2 Hz, 1H), 4.84 (dd, *J* = 2.8, 1.2 Hz, 1H), 4.69 (t, *J* = 2.8 Hz, 1H), 4.26 (s, 5H), 3.82 (s, 6H).

^{13}C NMR (101 MHz, $CDCl_3$) δ 193.1, 160.5, 138.1, 108.2, 99.1, 92.7, 76.6, 75.2, 71.9, 71.2, 68.5, 55.4.

IR (neat): ν_{max} (cm^{-1}) = 2974, 2906, 1661, 1592, 1459, 1429, 1353, 1254, 1204, 1157, 1103, 1063, 1049, 1007, 928, 896, 859, 837, 819, 744, 691, 672.

HRMS (ESI) calcd for $C_{19}H_{18}[^{56}Fe]O_3Na$ $[M+Na]^+$: 373.0498. Found: 373.0491.

HPLC The enantiomeric excess was determined by Daicel Chiralpak AD-H (0.46 cm × 25 cm), Hexanes / IPA = 70 / 30, 0.7 mL/min, λ = 254 nm, t (minor) = 5.41 min, t (major) = 5.98 min.

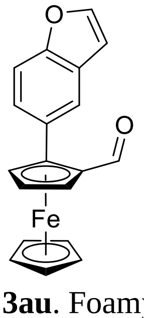

3au. Foamy dark red solid (46.9 mg, 71% yield, 98% ee).

Analytical data for **3au**:

$[\alpha]_D^{26}$ = -187.9 (c = 0.1, Acetone).

^{1}H NMR (400 MHz, $CDCl_3$) δ 10.23 (s, 1H), 7.72 (s, 1H), 7.66 (d, J = 2.0 Hz, 1H), 7.50 (s, 2H), 6.79 (d, J = 2.0 Hz, 1H), 5.02-5.00 (m, 1H), 4.86-4.84 (m, 1H), 4.72 (t, J = 2.8 Hz, 1H), 4.27 (s, 5H).

^{13}C NMR (101 MHz, $CDCl_3$) δ 193.3, 154.3, 145.8, 130.4 127.6, 126.3, 122.2, 111.2, 106.5, 93.5, 75.1, 71.9, 71.1, 68.3.

IR (neat): ν_{max} (cm^{-1}) = 3671, 2983, 2904, 1663, 1519, 1450, 1408, 1246, 1175, 1060, 894, 798.

HRMS (ESI) calcd for $C_{19}H_{14}[^{56}Fe]O_2Na$ $[M+Na]^+$: 353.0235. Found: 353.0236.

HPLC The enantiomeric excess was determined by Daicel Chiralpak AD-H (0.46 cm × 25 cm), Hexanes / IPA = 90 / 10, 1.0 mL/min, λ = 254 nm, t (major) = 8.60 min, t (minor) = 9.50 min.

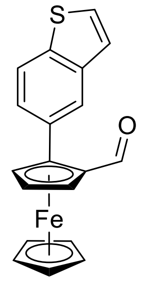

3av. Foamy dark red solid (50.5 mg, 73% yield, >99% ee).

Analytical data for **3av**:

$[\alpha]_D^{25}$ = -177.7 (c = 0.1, Acetone).

^{1}H NMR (400 MHz, $CDCl_3$) δ 10.26 (s, 1H), 7.92 (d, J = 2.0 Hz, 1H), 7.87 (d, J = 8.4 Hz, 1H), 7.57 (dd, J = 8.4, 1.6 Hz, 1H), 7.50 (d, J = 5.2 Hz, 1H), 7.35 (d, J = 5.2 Hz, 1H), 5.03 (dd, J = 2.8, 1.6 Hz, 1H), 4.90 (dd, J = 2.8, 1.6 Hz, 1H), 4.74 (t, J = 2.8 Hz, 1H), 4.27 (s, 5H).

^{13}C NMR (101 MHz, $CDCl_3$) δ 193.1, 139.7, 138.8, 132.1, 127.4, 126.2, 124.4, 123.7, 122.3, 92.9, 75.1, 72.0, 71.2, 68.5.

IR (neat): ν_{max} (cm^{-1}) = 3105, 2974, 2919, 1662, 1598, 1495, 1427, 1325, 1302, 1261, 1219, 1106, 1082, 1051, 1002, 881, 823, 759, 733, 700, 662, 623.

HRMS (ESI) calcd for $C_{19}H_{14}[^{56}Fe]OSNa$ $[M+Na]^+$: 369.0007. Found: 368.9999.

HPLC The enantiomeric excess was determined by Daicel Chiralpak AD-H (0.46 cm × 25 cm), Hexanes / IPA = 90 / 10, 1.0 mL/min, λ = 254 nm, t (major) = 10.10 min, t (minor) = 10.83 min.

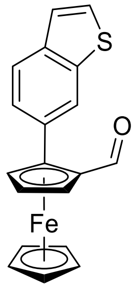

3aw. Foamy dark red solid (47.7 mg, 69% yield, >99% ee).

Analytical data for **3aw**:

$[\alpha]_D^{25}$ = -301.1 (*c* = 0.1, Acetone).

^{1}H NMR (400 MHz, $CDCl_3$) δ 10.26 (s, 1H), 8.01 (s, 1H), 7.81 (d, *J* = 8.2 Hz, 1H), 7.63 – 7.54 (m, 1H), 7.48 (d, *J* = 5.4 Hz, 1H), 7.35 (d, *J* = 5.4 Hz, 1H), 5.03 (dd, *J* = 2.7, 1.4 Hz, 1H), 4.91 (dd, *J* = 2.5, 1.5 Hz, 1H), 4.74 (t, *J* = 2.7 Hz, 1H), 4.28 (s, 5H).

^{13}C NMR (101 MHz, $CDCl_3$) δ 193.0, 140.0, 138.8, 132.2, 126.9, 126.4, 123.6, 123.3, 123.1, 92.7, 75.2, 72.1, 71.2, 68.7.

IR (neat): ν_{max} (cm^{-1}) = 2921, 1662, 1598, 1495, 1427, 1325, 1302, 1262, 1219, 1106, 1084, 1049, 1002, 881, 823, 760, 733, 700, 662, 624.

HRMS (ESI) calcd for $C_{19}H_{14}[^{56}Fe]OSNa$ $[M+Na]^+$: 369.0007. Found: 368.9998.

HPLC The enantiomeric excess was determined by Daicel Chiralpak AD-H (0.46 cm × 25 cm), Hexanes / IPA = 90 / 10, 1.0 mL/min, λ = 254 nm, t (major) = 9.13 min, t (minor) = 9.93 min.

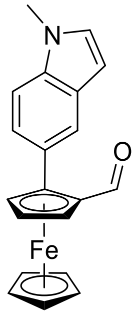

3ax. Foamy dark red solid (49.4 mg, 72% yield, 99% ee).

Analytical data for **3ax**:

$[\alpha]_D^{25}$ = -195.9 (*c* = 0.1, Acetone).

^{1}H NMR (400 MHz, $CDCl_3$) δ 10.29 (s, 1H), 7.75 (d, *J* = 2.0 Hz, 1H), 7.43 (dd, *J* = 8.4, 1.6 Hz, 1H), 7.33 (d, *J* = 8.4 Hz, 1H), 7.10 (d, *J* = 2.8 Hz, 1H), 6.50 (d, *J* = 2.8 Hz, 1H), 5.00 (dd, *J* = 2.8, 1.6 Hz, 1H), 4.86 (dd, *J* = 2.8, 1.6 Hz, 1H), 4.70 (t, *J* = 2.8 Hz, 1H), 4.26 (s, 5H), 3.83 (s, 3H).

^{13}C NMR (101 MHz, $CDCl_3$) δ 193.9, 136.1, 129.7, 128.4, 126.3, 123.8, 121.9, 109.1, 101.0, 95.0, 76.8, 74.7, 71.7, 71.0, 67.6, 32.9.

IR (neat): ν_{max} (cm^{-1}) = 2973, 2904, 1663, 1610, 1520, 1449, 1430, 1411, 1325, 1296, 1264, 1243, 1175, 1104, 1061, 1028, 962, 866, 832, 799, 758.

HRMS (ESI) calcd for $C_{20}H_{17}[^{56}Fe]ONNa$ $[M+Na]^+$: 366.0552. Found: 366.0542.

HPLC The enantiomeric excess was determined by Daicel Chiralcel OD-H (0.46 cm × 25 cm), Hexanes / IPA = 80 / 20, 0.7 mL/min, λ = 254 nm, t (major) = 9.78 min, t (minor) = 10.86 min.

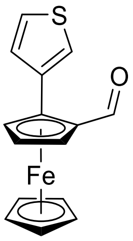

3ay. Foamy dark red solid (40.3 mg, 68% yield, >99% ee).

Analytical data for **3ay**:

$[\alpha]_D^{25}$ = -79.3 (*c* = 0.1, Acetone).

^{1}H NMR (400 MHz, $CDCl_3$) δ 10.24 (s, 1H), 7.37-7.34 (m, 2H), 7.30-7.27 (m, 1H), 4.95 (dd, *J* = 2.8, 1.2 Hz, 1H), 4.83 (dd, *J* = 2.8, 1.2 Hz, 1H), 4.69 (t, *J* = 2.8 Hz, 1H), 4.24 (s, 5H).

^{13}C NMR (101 MHz, $CDCl_3$) δ 193.1, 136.3, 128.7, 125.7, 122.5, 86.1, 76.8, 74.1, 72.1, 71.0, 69.1.

IR (neat): ν_{max} (cm^{-1}) = 3104, 2922, 2853, 1666, 1434, 1407, 1314, 1266, 1224, 1204, 1106, 1082, 1055, 1032, 1002, 890, 844, 823, 786, 749, 698, 670, 627.

HRMS (ESI) calcd for $C_{15}H_{12}[^{56}Fe]OSNa$ $[M+Na]^+$: 318.9851. Found: 318.9845.

HPLC The enantiomeric excess was determined by Daicel Chiralpak IG (0.46 cm ×25 cm), Hexanes / IPA = 95 / 05, 1.0 mL/min, λ = 254 nm, t (major) = 22.76 min, t (minor) = 24.28 min.

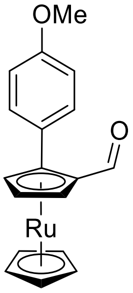

3ba. Foamy dark red solid (52.7 mg, 72% yield, >99% ee).

Analytical data for **3ba**:

$[\alpha]_D^{25}$ = -49.7 (*c* = 0.1, Acetone).

^{1}H NMR (400 MHz, $CDCl_3$) δ 9.89 (s, 1H), 7.33 (d, *J* = 8.8 Hz, 2H), 6.83 (d, *J* = 8.8 Hz, 2H), 5.24 (dd, *J* = 2.8, 1.6 Hz, 1H), 5.04 (dd, *J* = 2.8, 1.6 Hz, 1H), 4.91 (d, *J* = 2.8 Hz, 1H), 4.64 (s, 5H), 3.82 (s, 3H).

^{13}C NMR (101 MHz, $CDCl_3$) δ 190.3, 159.0, 131.5, 126.9, 113.5, 96.0, 82.8, 76.6, 73.2, 72.7, 69.7, 55.3.

IR (neat): ν_{max} (cm^{-1}) = 2983, 2903, 1662, 1613, 1532, 1440, 1401, 1354, 1251, 1226, 1060, 893, 813.

HRMS (ESI) calcd for $C_{18}H_{17}RuO_2$ $[M+H]^+$: 367.0267. Found: 367.0263.

HPLC The enantiomeric excess was determined by Daicel Chiralcel OD-3 (0.46 cm × 25 cm), Hexanes / IPA = 95 / 05, 0.7 mL/min, λ = 254 nm, t (major) = 17.66 min.

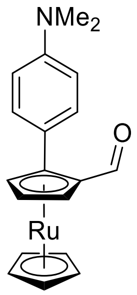

3ca. Foamy dark red solid (45.5 mg, 60% yield, 98% ee).

Analytical data for **3ca**:

$[\alpha]_D^{25}$ = -103.7 (*c* = 0.1, Acetone).

^{1}H NMR (400 MHz, $CDCl_3$) δ 9.93 (s, 1H), 7.27 (d, *J* = 8.4 Hz, 2H), 6.65 (d, *J* = 8.8 Hz, 2H), 5.23 (dd, *J* = 2.8, 1.6 Hz, 1H), 5.03 (dd, *J* = 2.8, 1.6 Hz, 1H), 4.88 (t, *J* = 2.8 Hz, 1H), 4.62 (s, 5H), 2.97 (s, 6H).

^{13}C NMR (101 MHz, $CDCl_3$) δ 190.7, 149.8, 131.1, 122.1, 111.9, 96.9, 82.9, 76.2, 73.0, 72.6, 69.3, 40.5.

IR (neat): ν_{max} (cm^{-1}) = 2983, 2904, 1661, 1612, 1531, 1480, 1440, 1401, 1353, 1263, 1225, 1199, 1169, 1098, 1062, 813, 739.

HRMS (ESI) calcd for $C_{19}H_{20}RuNO$ $[M+H]^+$: 380.0583. Found: 380.0584.

HPLC The enantiomeric excess was determined by Daicel Chiralpak AD-3 (0.46 cm × 25 cm), Hexanes / IPA = 90 / 10, 0.7 mL/min, λ = 254 nm, t (major) = 12.62 min, t (minor) = 13.22 min.

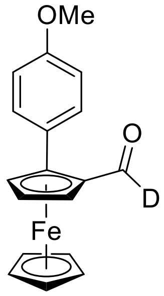

3da. Foamy dark red solid (48.2 mg, 75% yield, >99% ee).

Analytical data for **3da**:

$[\alpha]_D^{26}$ = -257.9 (*c* = 0.1, Acetone).

^{1}H NMR (400 MHz, CD_2Cl_2) δ 7.48 (d, *J* = 8.8 Hz, 2H), 6.91 (d, *J* = 8.8 Hz, 2H), 4.93 (dd, *J* = 2.8, 1.6 Hz, 1H), 4.81 (dd, *J* = 2.8, 1.6 Hz, 1H), 4.70 (t, *J* = 2.8 Hz, 1H), 4.24 (s, 5H), 3.84 (s, 3H).

^{13}C NMR (101 MHz, CD_2Cl_2) δ 159.8, 131.6, 128.8, 114.5, 93.3, 75.3, 72.6, 71.8, 69.2, 56.1.

IR (neat): ν_{max} (cm^{-1}) = 2972, 2904, 1663, 1520, 1449, 1431, 1412, 1265, 1242, 1175, 1105, 1062, 1027, 832, 799, 757.

HRMS (ESI) calcd for $C_{18}H_{15}[^{56}Fe]O_2DNa$ $[M+Na]^+$: 344.0455. Found: 344.0448.

HPLC The enantiomeric excess was determined by Daicel Chiralcel OD-H (0.46 cm × 25 cm), Hexanes / IPA = 90 / 10, 0.7 mL/min, λ = 254 nm, t (major) = 14.91 min.

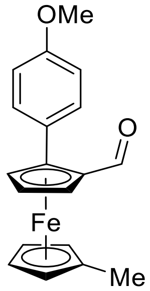

3fa. Foamy dark red solid (51.9 mg, 78% yield, >99% ee).

Analytical data for **3fa**:

$[\alpha]_D^{19}$ = -259.9 (c = 0.1 Acetone).

^{1}H NMR (400 MHz, $CDCl_3$) δ 10.14 (s, 1H), 7.45 (d, *J* = 8.8 Hz, 2H), 6.90 (d, *J* = 8.8 Hz, 2H), 4.87 (t, *J* = 1.6 Hz, 1H), 4.73-4.71 (m, 1H), 4.62 (t, *J* = 2.8 Hz, 1H), 4.12-4.09 (m, 4H), 3.84 (s, 3H), 1.84 (s, 3H).

^{13}C NMR (101 MHz, $CDCl_3$) δ 193.2, 158.9, 130.7, 127.8, 113.8, 92.5, 86.5, 75.1, 72.8, 72.2, 71.7, 70.6, 70.2, 69.1, 55.3, 13.7.

IR (neat): ν_{max} (cm^{-1}) = 3082, 2921, 2835, 2759, 1659, 1609, 1573, 1520, 1450, 1434, 1413, 1397, 1370, 1327, 1293, 1266, 1244, 1217, 1175, 1129, 1105, 1029, 961, 909, 829, 797, 761, 727, 666, 647, 629.

HRMS (ESI) calcd for $C_{19}H_{19}[^{56}Fe]O_2$ $[M+H]^+$: 335.0729. Found: 335.0723.

HPLC The enantiomeric excess was determined by Daicel Chiralcel OD-H (0.46 cm × 25 cm), Hexanes / IPA = 90 / 10, 1.0 mL/min, λ = 254 nm, t (major) = 10.44 min, t (minor) = 12.82 min.

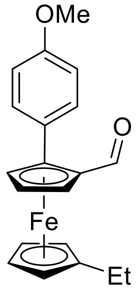

3ga. Foamy dark red solid (48.7 mg, 70% yield, 99% ee).

Analytical data for **3ga**:

$[\alpha]_D^{26}$ = -37.8 (c = 0.1, Acetone).

^{1}H NMR (400 MHz, CD_2Cl_2) δ 10.05 (s, 1H), 7.39 (d, J = 8.8 Hz, 2H), 6.81 (d, J = 8.8 Hz, 2H), 4.75 (dd, J = 2.8, 1.6 Hz, 1H), 4.67 (dd, J = 2.8, 1.6 Hz, 1H), 4.55 (td, J = 2.8, 0.8 Hz, 1H), 4.08-4.06 (m, 1H), 4.05-4.01 (m, 3H), 3.75 (s, 3H), 2.14 (q, J = 7.8 Hz, 2H), 0.98 (t, J = 7.8 Hz, 3H).

^{13}C NMR (101 MHz, CD_2Cl_2) δ 193.6, 159.8, 131.6, 128.8, 114.4, 94.3, 93.0, 77.6, 75.8, 73.5, 71.6, 71.2, 71.2, 71.0, 70.0, 56.1, 22.2, 15.3.

IR (neat): ν_{max} (cm^{-1}) = 2966, 2905, 1663, 1610, 1520, 1448, 1431, 1412, 1325, 1296, 1263, 1243, 1175, 1103, 1071, 1026, 962, 864, 830, 799, 757, 677.

HRMS (ESI) calcd for $C_{20}H_{20}[^{56}Fe]O_2Na$ $[M+Na]^+$: 371.0705. Found: 371.0706.

HPLC The enantiomeric excess was determined by Daicel Chiralcel OD-H (0.46 cm × 25 cm), Hexanes / IPA = 90 / 10, 0.7 mL/min, λ = 254 nm, t (major) = 11.75 min, t (minor) = 12.30 min.

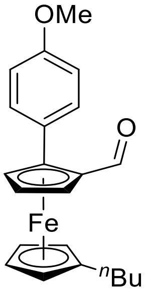

3ha. Foamy dark red solid (45.1 mg, 60% yield, 98% ee).

Analytical data for **3ha**:

$[\alpha]_D^{26}$ = -38.1 (*c* = 0.1, Acetone).

^{1}H NMR (400 MHz, CD_2Cl_2) δ 10.13 (s, 1H), 7.48 (d, *J* = 8.8 Hz, 2H), 6.90 (d, *J* = 8.8 Hz, 2H), 4.83 (dd, *J* = 2.8, 1.6 Hz, 1H), 4.75 (dd, *J* = 2.8, 1.6 Hz, 1H), 4.63 (td, *J* = 2.8, 0.8 Hz, 1H), 4.13 (t, *J* = 2.0 Hz, 1H), 4.12-4.10 (m, 3H), 3.84 (s, 3H), 2.20 (dd, *J* = 8.8, 7.2 Hz, 2H), 1.43-1.33 (m, 2H), 1.33-1.23 (m, 2H), 0.88 (t, *J* = 7.2 Hz, 3H).

^{13}C NMR (101 MHz, CD_2Cl_2) δ 193.6, 159.8, 131.6, 128.8, 114.4, 93.0, 92.7, 77.6, 75.8, 73.5, 71.9, 71.6, 71.5, 71.2, 70.0, 56.1, 34.0, 29.0, 23.3, 14.5.

IR (neat): ν_{max} (cm^{-1}) = 2968, 2906, 1663, 1610, 1520, 1449, 1431, 1413, 1325, 1296, 1264, 1243, 1175, 1104, 1026, 962, 864, 831, 799, 757, 673, 629.

HRMS (ESI) calcd for $C_{22}H_{24}[^{56}Fe]O_2Na$ $[M+Na]^+$: 399.1018. Found: 399.1015.

HPLC The enantiomeric excess was determined by Daicel Chiralcel OD-H (0.46 cm × 25 cm), Hexanes / IPA = 90 / 10, 0.7 mL/min, λ = 254 nm, t (major) = 9.64 min, t (minor) = 10.78 min.

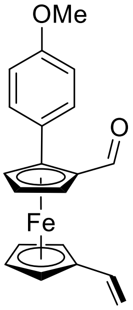

3ia. Foamy dark red solid (40.8 mg, 52% yield, 99% ee).

Analytical data for **3ia**:

$[\alpha]_D^{27}$ = -29.7 (*c* = 0.1, Acetone).

^{1}H NMR (400 MHz, $CDCl_3$) δ 10.09 (s, 1H), 7.45 (d, *J* = 8.8 Hz, 2H), 6.90 (d, *J* = 8.8 Hz, 2H), 6.29 (dd, *J* = 17.6, 10.8 Hz, 1H), 5.36 (d, *J* = 17.6 Hz, 1H), 5.14 (d, *J* = 10.8

Hz, 1H), 4.89-4.87 (m, 1H), 4.70 (t, J = 2.0 Hz, 1H), 4.60 (t, J = 2.8 Hz, 1H), 4.37 (dd, J = 8.4, 2.4 Hz, 2H), 4.27 (dd, J = 5.2, 1.6 Hz, 2H), 3.84 (s, 3H).

^{13}C NMR (101 MHz, $CDCl_3$) δ 193.5, 159.0, 132.5, 130.7, 127.5, 113.8, 113.7, 92.9, 85.9, 75.6, 73.4, 71.8, 71.7, 69.5, 69.5, 69.2, 55.3.

IR (neat): ν_{max} (cm^{-1}) = 2968, 2919, 1663, 1604, 1520, 1427, 1325, 1298, 1264, 1243, 1219, 1175, 1105, 1050, 1028, 1005, 825, 759, 733, 701, 662, 624.

HRMS (ESI) calcd for $C_{20}H_{18}[^{56}Fe]O_2Na$ $[M+Na]^+$: 369.0548. Found: 369.0542.

HPLC The enantiomeric excess was determined by Daicel Chiralcel OD-H (0.46 cm × 25 cm), Hexanes / IPA = 80 / 20, 0.7 mL/min, λ = 254 nm, t (major) = 10.27 min, t (minor) = 11.72 min.

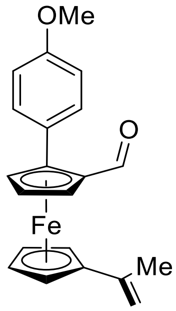

3ja. Foamy dark red solid (52.8 mg, 65% yield, 99% ee).

Analytical data for **3ja**:

$[\alpha]_D^{26}$ = -13.8 (c = 0.1, Acetone).

^{1}H NMR (400 MHz, $CDCl_3$) δ 10.12 (s, 1H), 7.44 (d, J = 8.8 Hz, 2H), 6.89 (d, J = 8.8 Hz, 2H), 5.15 (s, 1H), 4.94 (dd, J = 2.8, 1.2 Hz, 1H), 4.86 (dd, J = 2.8, 1.2 Hz, 1H), 4.70 (dd, J = 2.8, 1.2 Hz, 1H), 4.58 (d, J = 2.8 Hz, 1H), 4.43-4.39 (m, 2H), 4.28-4.26 (m, 2H), 3.85 (s, 3H), 1.92 (s, 3H).

^{13}C NMR (101 MHz, $CDCl_3$) δ 193.3, 159.0, 139.3, 130.7, 127.6, 113.8, 110.7, 92.8, 88.9, 75.5, 73.5, 71.92, 71.86, 69.6, 68.4, 68.3, 55.3, 21.2.

IR (neat): ν_{max} (cm^{-1}) = 2973, 2920, 1663, 1605, 1520, 1427, 1325, 1298, 1264, 1243, 1218, 1175, 1105, 1049, 1028, 1005, 825, 759, 733, 701, 662, 626.

HRMS (ESI) calcd for $C_{21}H_{21}[^{56}Fe]O_2$ $[M+H]^+$: 361.0886. Found: 361.0880.

HPLC The enantiomeric excess was determined by Daicel Chiralcel OD-H (0.46 cm × 25 cm), Hexanes / IPA = 80 / 20, 0.7 mL/min, λ = 254 nm, t (major) = 10.08 min, t (minor) = 11.94 min.

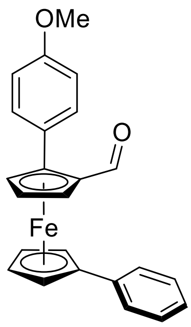

3ka. Foamy dark red solid (55.5 mg, 70% yield, 99% ee).

Analytical data for **3ka**:

$[\alpha]_D^{26}$ = -230.2 (c = 0.1, Acetone).

^{1}H NMR (400 MHz, $CDCl_3$) δ 10.07 (s, 1H), 7.38-7.22 (m, 7H), 6.82 (d, J = 8.0 Hz, 2H), 4.81 (d, J = 2.4 Hz, 1H), 4.71 (s, 1H), 4.66 (s, 1H), 4.62 (d, J = 2.8 Hz, 1H), 4.48 (d, J = 2.8 Hz, 1H), 4.36 (d, J = 3.6 Hz, 2H), 3.83 (s, 3H).

^{13}C NMR (101 MHz, $CDCl_3$) δ 193.4, 158.9, 136.3, 130.6, 128.5, 127.2, 126.8, 126.2, 113.7, 92.9, 87.9, 76.1, 74.0, 72.3, 72.0, 70.0, 68.9, 68.7, 55.3.

IR (neat): ν_{max} (cm^{-1}) = 2975, 2904, 1668, 1610, 1520, 1437, 1400, 1332, 1297, 1248, 1178, 1105, 1034, 961, 887, 834, 793, 761, 739, 669.

HRMS (ESI) calcd for $C_{24}H_{20}[^{56}Fe]O_2Na$ $[M+Na]^+$: 419.0705. Found: 419.0700.

HPLC The enantiomeric excess was determined by Daicel Chiralpak IG (0.46 cm × 25 cm), Hexanes / IPA = 70 / 30, 0.7 mL/min, λ = 254 nm, t (major) = 15.78 min, t (minor) = 16.37 min.

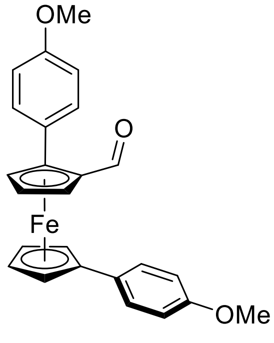

3la. Foamy dark red solid (57.1 mg, 67% yield, 99% ee).

Analytical data for **3la**:

$[\alpha]_D^{26}$ = -121.6 (c = 0.1, Acetone).

^{1}H NMR (400 MHz, $CDCl_3$) δ 10.07 (s, 1H), 7.32-7.27 (m, 4H), 6.81 (dd, J = 8.8, 3.6 Hz, 4H), 4.80 (t, J = 2.4 Hz, 1H), 4.63 (dt, J = 3.6, 2.0 Hz, 2H), 4.59 (t, J = 2.0 Hz, 1H), 4.49 (t, J = 2.8 Hz, 1H), 4.33 (t, J = 2.0 Hz, 2H), 3.83 (s, 3H), 3.82 (s, 3H).

^{13}C NMR (101 MHz, $CDCl_3$) δ 193.5, 158.9, 158.7, 130.6, 128.2, 127.3, 127.2, 114.0, 113.7, 92.7, 88.5, 76.0, 73.9, 71.8, 71.5, 69.9, 68.5, 68.2, 55.3, 55.2.

IR (neat): ν_{max} (cm^{-1}) = 2984, 2904, 1668, 1610, 1520, 1437, 1400, 1332, 1297, 1248, 1178, 1105, 1034, 886, 834, 793, 761, 739, 670.

HRMS (ESI) calcd for $C_{25}H_{22}[^{56}Fe]O_3Na$ $[M+Na]^+$: 449.0811. Found: 449.0812.

HPLC The enantiomeric excess was determined by Daicel Chiralpak IG (0.46 cm × 25 cm), Hexanes / IPA = 70 / 30, 0.7 mL/min, λ = 254 nm, t (minor) = 21.13 min, t (major) = 23.25 min.

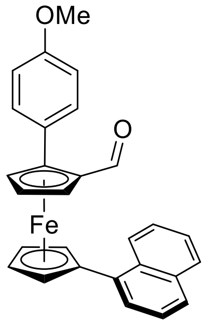

3ma. Foamy dark red solid (51.7 mg, 58% yield, 99% ee).

Analytical data for **3ma**:

$[\alpha]_D^{26}$ = -122.6 (c = 0.1, Acetone).

^{1}H NMR (400 MHz, $CDCl_3$) δ 10.26 (s, 1H), 8.32 (d, J = 8.4 Hz, 1H), 7.86 (d, J = 8.0 Hz, 1H), 7.77 (t, J = 9.2 Hz, 2H), 7.47-7.38 (m, 6H), 6.84 (d, J = 8.0 Hz, 2H), 4.98 (s, 1H), 4.76 (d, J = 8.8 Hz, 2H), 4.70 (s, 1H), 4.62 (s, 1H), 4.44 (s, 2H), 3.83 (s, 3H).

^{13}C NMR (101 MHz, $CDCl_3$) δ 193.7, 159.0, 133.8, 133.6, 131.6, 130.8, 128.6, 128.2, 127.7, 127.3, 125.8, 125.6, 125.5, 125.2, 113.8, 92.8, 89.4, 76.5, 76.3, 74.5, 72.9, 72.4, 72.1, 71.7, 70.4, 55.3.

IR (neat): ν_{max} (cm^{-1}) = 2973, 2904, 1668, 1610, 1520, 1437, 1401, 1332, 1297, 1248, 1178, 1104, 1034, 887, 834, 792, 761, 739, 669, 624.

HRMS (ESI) calcd for $C_{28}H_{22}[^{56}Fe]O_2Na$ $[M+Na]^+$: 469.0861. Found: 469.0853.

HPLC The enantiomeric excess was determined by Daicel Chiralpak IF (0.46 cm × 25 cm), Hexanes / IPA = 80 / 20, 0.7 mL/min, λ = 254 nm, t (major) = 15.97 min, t (minor) = 17.61 min.

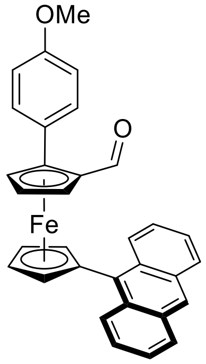

3na. Foamy dark red solid (50.6 mg, 51% yield, >99% ee).

Analytical data for **3na**:

$[\alpha]_D^{26}$ = -227.9 (*c* = 0.1, Acetone).

^{1}H NMR (400 MHz, $CDCl_3$) δ 10.29 (s, 1H), 8.91 (d, *J* = 8.4 Hz, 2H), 8.44 (s, 1H), 8.00 (d, *J* = 8.8 Hz, 2H), 7.46-7.37 (m, 7H), 6.85 (d, *J* = 8.4 Hz, 2H), 5.09 (t, *J* = 2.0 Hz, 1H), 4.92-4.91 (m, 1H), 4.85-4.83 (m, 1H), 4.77 (t, *J* = 2.0 Hz, 1H), 4.67-4.65 (m, 1H), 4.59 (t, *J* = 2.8 Hz, 1H), 4.52 (d, *J* = 2.4 Hz, 1H), 3.84 (s, 3H).

^{13}C NMR (101 MHz, $CDCl_3$) δ 194.0, 159.1, 131.6, 131.0, 130.5, 129.4, 128.7, 127.6, 127.3, 127.1, 125.0, 124.8, 113.8, 92.8, 86.7, 76.1, 75.9, 75.0, 74.5, 72.6, 71.1, 70.0, 55.3.

IR (neat): ν_{max} (cm^{-1}) = 2974, 2904, 1668, 1610, 1520, 1437, 1400, 1333, 1297, 1248, 1178, 1036, 887, 834, 792, 760, 740, 670.

HRMS (ESI) calcd for $C_{32}H_{24}[^{56}Fe]O_2Na$ $[M+Na]^+$: 519.1018. Found: 519.1018.

HPLC The enantiomeric excess was determined by Daicel Chiralpak AD-H (0.46 cm × 25 cm), Hexanes / IPA = 70 / 30, 0.7 mL/min, λ = 254 nm, t (minor) = 5.76 min, t (major) = 6.70 min.

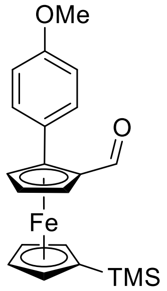

3oa. Dark red oil (61.9 mg, 79% yield, >99% ee).

Analytical data for **3oa**:

$[\alpha]_D^{29}$ = -146.6 (c = 0.1 Acetone).

^{1}H NMR (400 MHz, $CDCl_3$) δ 10.19 (s, 1H), 7.45 (d, J = 8.8 Hz, 2H), 6.90 (d, J = 8.8 Hz, 2H), 4.91 (dd, J = 2.8, 1.6 Hz, 1H), 4.76-4.74 (m, 1H), 4.62 (t, J = 2.8 Hz, 1H), 4.38 (q, J = 2.0 Hz, 1H), 4.33 (q, J = 2.0 Hz, 1H), 4.24-4.22 (m, 1H), 4.14-4.12 (m, 1H), 3.84 (s, 3H), 0.22 (s, 9H).

^{13}C NMR (101 MHz, $CDCl_3$) δ 193.3, 159.0, 130.7, 127.8, 113.8, 92.3, 76.3, 75.3, 75.2, 75.1, 74.8, 74.6, 74.3, 72.0, 68.6, 55.3, -0.4.

IR (neat): ν_{max} (cm^{-1}) = 2952, 2835, 1666, 1610, 1520, 1434, 1399, 1328, 1294, 1267, 1245, 1217, 1176, 1162, 1106, 1034, 901, 826, 754, 729, 694, 666, 629.

HRMS (ESI) calcd for $C_{21}H_{25}[^{56}Fe]SiO_2$ $[M+H]^+$: 393.0968. Found: 393.0964.

HPLC The enantiomeric excess was determined by Daicel Chiralcel OJ-H (0.46 cm × 25 cm), Hexanes / IPA = 90 / 10, 1.0 mL/min, λ = 254 nm, t (minor) = 8.23 min, t (major) = 14.15 min.

Transformations of (S_p)-3aa

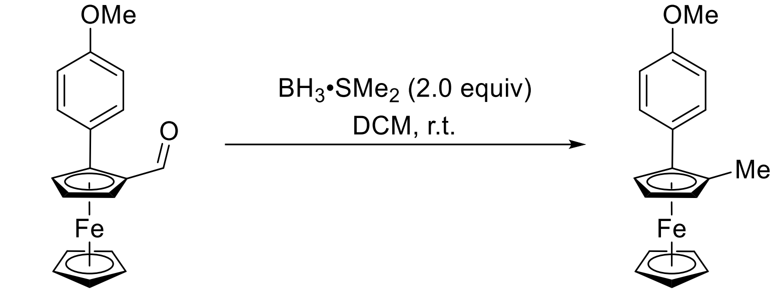

BH_3/SMe_2 (0.1 mL, 2.0 M in THF, 2.0 equiv) was slowly added to a suspension of (S_p)-**3aa** (32.0 mg, 0.1 mmol, 1.0 equiv, >99% ee) in DCM (2.0 mL) at 0 °C. Then the reaction mixture was stirred at room temperature. After the reaction was complete (monitored by TLC), it was quenched by adding $NaHCO_3$ solution (aq. 2 M, 5 mL). The mixture was extracted with ethyl acetate (5 mL × 3). The combined organic phases were washed with brine and dried over anhydrous sodium sulfate. After filtration, the solvent was removed under reduced pressure. The residue was purified by silica gel column chromatography (hexane/ethyl acetate = 50/1, v/v) to afford product **4a**.

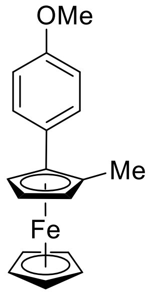

4a. Orange yellow oil (26.9 mg, 88% yield, >99% ee).

Analytical data for **4a**:

$[\alpha]_D^{24}$ = +152.9 (c = 0.1, Acetone).

^{1}H NMR (400 MHz, $CDCl_3$) δ 7.49 (d, J = 8.8 Hz, 2H), 6.90 (d, J = 8.4 Hz, 2H), 4.34 (dd, J = 2.4, 1.2 Hz, 1H), 4.19 (t, J = 2.0 Hz, 1H), 4.12 (d, J = 2.4 Hz, 1H), 4.05 (s, 5H), 3.85 (s, 3H), 2.18 (s, 3H).

^{13}C NMR (101 MHz, $CDCl_3$) δ 157.9, 131.0, 129.8, 113.4, 86.9, 81.8, 70.2, 70.0, 68.6, 65.8, 55.2, 14.7.

IR (neat): ν_{max} (cm^{-1}) = 2960, 1611, 1522, 1460, 1410, 1378, 1296, 1269, 1244, 1178, 1146, 1105, 1035, 1001, 828, 658.

HRMS (ESI) calcd for $C_{18}H_{18}[^{56}Fe]O$ $[M]^+$: 306.0702. Found: 306.0700.

HPLC The enantiomeric excess was determined by Daicel Chiralpak OD-H (0.46 cm × 25 cm), Hexanes / IPA = 98 / 02, 1.0 mL/min, λ = 254 nm, t (major) = 7.20 min, t (minor) = 8.67 min.

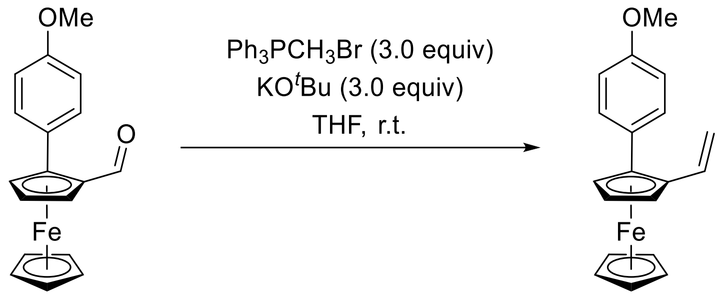

(S_p)-**3aa**, >99% ee　　**4b**

t-BuOK (33.6 mg, 0.3 mmol, 3.0 equiv) was added to a suspension of methyltriphenyl-phosphonium bromide (107.2 mg, 0.3 mmol, 3.0 equiv) in dry THF (2.0 mL) at 0 °C. The reaction mixture was stirred for 30 min at room temperature. Then a solution of (S_p)-**3aa** (32.0 mg, 0.1 mmol, 1.0 equiv, >99% ee) in dry THF (2.0 mL) was added into the above-mentioned solution at 0 °C. The reaction mixture was stirred for another 3 h at room temperature. After the reaction was complete (monitored by TLC), the reaction was then quenched by adding $NaHCO_3$ solution (aq. 2 M, 5 mL). The mixture was extracted with ethyl acetate (5 mL × 3). The combined organic phases were washed with brine and dried over anhydrous sodium sulfate. After filtration, the solvent was removed under reduced pressure. The residue was purified by silica gel column chromatography (hexane/ethyl acetate = 50/1, v/v) to afford product **4b**.

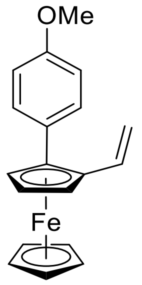

4b. Foamy orange yellow solid (27.2 mg, 85% yield, >99% ee).

Analytical data for **4b**:

$[\alpha]_D^{19}$ = -436.3 (c = 0.1 Acetone).

^{1}H NMR (400 MHz, $CDCl_3$) δ 7.47 (d, J = 8.4 Hz, 2H), 6.90 (d, J = 8.8 Hz, 2H), 6.72 (dd, J = 17.6, 10.8 Hz, 1H), 5.44 (dd, J = 17.6, 1.6 Hz, 1H), 5.12 (dd, J = 10.8, 1.6 Hz, 1H), 4.60-4.58 (m, 1H), 4.45 (dd, J = 2.4, 1.2 Hz, 1H), 4.30 (t, J = 2.8 Hz, 1H), 4.07 (s, 5H), 3.85 (s, 3H).

^{13}C NMR (101 MHz, $CDCl_3$) δ 158.2, 133.9, 130.5, 130.2, 113.4, 111.9, 87.7, 81.6, 70.5, 70.2, 67.4, 65.2, 55.3.

IR (neat): ν_{max} (cm^{-1}) = 3003, 2965, 2934, 2839, 2338, 1627, 1609, 1519, 1448, 1435, 1414, 1299, 1290, 1265, 1241, 1174, 1125, 1103, 1065, 1029, 998, 988, 955, 936, 885, 823, 793, 724, 672, 667, 620.

HRMS (ESI) calcd for $C_{19}H_{19}[^{56}Fe]O$ $[M+H]^+$: 319.0780. Found: 319.0780.

HPLC The enantiomeric excess was determined by Daicel Chiralcel OJ-H (0.46 cm × 25 cm), Hexanes / IPA = 80 / 20, 0.7 mL/min, λ = 254 nm, t (minor) = 8.89 min, t (major) = 9.83 min.

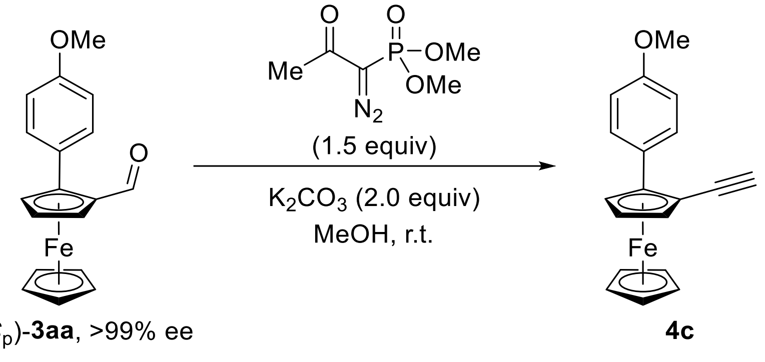

(S_p)-**3aa**, >99% ee **4c**

The dimethyl *P*-(1-diazo-2-oxopropyl)phosphonate (28.8 mg, 0.15 mmol, 1.5 equiv) was slowly added to a suspension of (S_p)-**3aa** (32.0 mg, 0.1 mmol, >99% ee), K_2CO_3 (27.6 mg, 0.2 mmol, 1.0 equiv, 2.0 equiv) in MeOH (2.0 mL) at 0 °C and then stirred at room temperature. After the reaction was complete (monitored by TLC), the reaction was quenched by adding $NaHCO_3$ solution (aq. 2 M, 5 mL). The mixture was extracted with ethyl acetate (5 mL × 3). The combined organic phases were washed with brine and dried over anhydrous sodium sulfate. After filtration, the solvent was

removed under reduced pressure. The residue was purified by silica gel column chromatography (hexane/ethyl acetate = 50/1, v/v) to afford product **4c**.

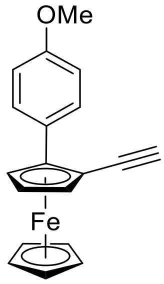

4c. Foamy orange yellow solid (25.3 mg, 80% yield, >99% ee).

Analytical data for **4c**:

$[\alpha]_D^{19}$ = +21.2 (c = 0.1 Acetone).

^{1}H NMR (400 MHz, $CDCl_3$) δ 7.75 (d, J = 8.8 Hz, 2H), 6.90 (d, J = 8.8 Hz, 2H), 4.58 (dd, J = 2.4, 1.2 Hz, 1H), 4.55 (dd, J = 2.8, 1.6 Hz, 1H), 4.29 (t, J = 2.4 Hz, 1H), 4.13 (s, 5H), 3.84 (s, 3H), 2.90 (s, 1H).

^{13}C NMR (101 MHz, $CDCl_3$) δ 158.4, 129.6, 128.9, 113.5, 88.4, 82.8, 76.0, 72.8, 71.5, 68.2, 68.1, 62.4, 55.3.

IR (neat): ν_{max} (cm^{-1}) = 3287, 3005, 2965, 2922, 2840, 2338, 2107, 1608, 1519, 1450, 1437, 1409, 1292, 1239, 1173, 1126, 1104, 1068, 1056, 1022, 1000, 956, 935, 884, 826, 792, 728, 663, 622, 607.

HRMS (ESI) calcd for $C_{19}H_{17}[^{56}Fe]O$ $[M+H]^+$: 317.0623. Found: 317.0621.

HPLC The enantiomeric excess was determined by Daicel Chiralcel IC (0.46 cm × 25 cm), Hexanes / IPA = 98 / 02, 1.0 mL/min, λ = 254 nm, t (minor) = 9.22 min, t (major) = 10.52 min.

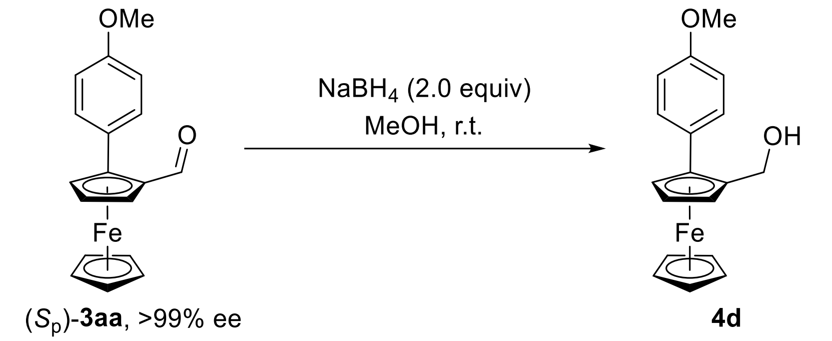

NaBH$_4$ (7.6 mg, 0.2 mmol, 2.0 equiv) was slowly added to a suspension of (S_p)-**3aa** (32.0 mg, 0.1 mmol, 1.0 equiv, >99% ee) in MeOH (2.0 mL) at 0 °C and then stirred at room temperature. After the reaction was complete (monitored by TLC), it was quenched by adding NaHCO$_3$ solution (aq. 2 M, 5 mL). The mixture was extracted with ethyl acetate (5 mL × 3). The combined organic phases were washed with brine and dried over anhydrous sodium sulfate. After filtration, the solvent was removed under reduced pressure. The residue was purified by silica gel column chromatography (hexane/ethyl acetate = 10/1, v/v) to afford product **4d**.

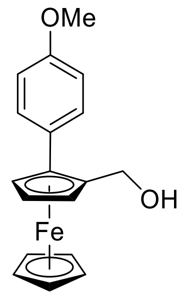

4d. Foamy orange yellow solid (28.9 mg, 90% yield, >99% ee).

Analytical data for **4d**:

$[\alpha]_D^{19}$ = +160.9 (c = 0.1 Acetone).

^{1}H NMR (400 MHz, CDCl$_3$) δ 7.54 (d, J = 8.8 Hz, 2H), 6.89 (d, J = 8.8 Hz, 2H), 4.59-4.51 (m, 2H), 4.47 (dd, J = 2.8, 1.6 Hz, 1H), 4.35 (dd, J = 2.4, 1.6 Hz, 1H), 4.25 (t, J = 2.4 Hz, 1H), 4.10 (s, 5H), 3.83 (s, 3H).

^{13}C NMR (101 MHz, CDCl$_3$) δ 158.2, 129.9, 129.8, 113.6, 87.8, 84.7, 70.1, 69.7, 69.4, 67.2, 59.8, 55.2.

IR (neat): ν_{max} (cm^{-1}) = 3329, 3080, 2932, 2834, 1609, 1521, 1454, 1438, 1411, 1281, 1243, 1177, 1140, 1105, 1054, 1031, 996, 937, 829, 794, 730.

HRMS (ESI) calcd for $C_{18}H_{18}[^{56}Fe]NaO_2$ $[M+Na]^+$: 345.0548. Found: 345.0551.

HPLC The enantiomeric excess was determined by Daicel Chiralcel OD-H (0.46 cm × 25 cm), Hexanes / IPA = 90 / 10, 1.0 mL/min, λ = 254 nm, t (minor) = 16.72 min, t (major) = 25.20 min.

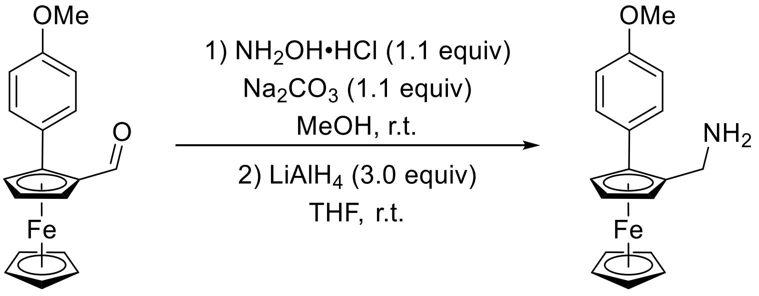

(S_p)-**3aa**, >99% ee **4e**

Na_2CO_3 (11.7 mg, 0.11 mmol, 1.1 equiv) was slowly added to a suspension of (S_p)-**3aa** (32.0 mg, 0.1 mmol, 1.0 equiv, >99% ee), NH_2OH/HCl (7.6 mg, 0.11 mmol, 1.1 equiv) in MeOH (2.0 mL) at room temperature. After the reaction was complete (monitored by TLC), it was then quenched by adding $NaHCO_3$ solution (aq. 2 M, 5 mL). The mixture was extracted with ethyl acetate (20 mL × 3). The combined organic phases were washed with brine and dried over anhydrous sodium sulfate. After filtration, the solvent was removed in vacuo to give a yellow oil, which was used without further purification.

Then, $LiAlH_4$ (11.4 mg, 0.3 mmol, 3.0 equiv) was slowly added to a suspension of the above yellow oil in THF at 0 °C and then stirred at room temperature. After the reaction was complete (monitored by TLC), it was then quenched by adding $NaHCO_3$ solution (aq. 2 M, 5 mL). The mixture was extracted with ethyl acetate (5 mL × 3). The combined organic phases were washed with brine and dried over anhydrous sodium sulfate. After filtration, the solvent was removed under reduced pressure. The residue was purified by silica gel column chromatography (DCM/MeOH = 20/1, v/v) to afford product **4e**.

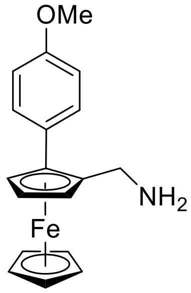

4e. Foamy orange yellow solid (20.9 mg, 65% yield, >99% ee).

Analytical data for **4e**:

$[\alpha]_D^{24}$ = -37.7 (*c* = 0.1, Acetone).

^{1}H NMR (400 MHz, CD_2Cl_2) δ 7.46 (d, J = 8.8 Hz, 2H), 6.88 (d, J = 8.8 Hz, 2H), 6.43 (br, 2H), 4.67-4.65 (m, 1H), 4.47-4.45 (m, 1H), 4.30 (t, J = 2.4 Hz, 1H), 4.21 (s, 5H), 4.10 (d, J_{AB} = 14.0 Hz, 1H), 4.00 (d, J_{BA} = 14.0 Hz, 1H), 3.80 (s, 3H).

^{13}C NMR (101 MHz, CD_2Cl_2) δ 159.5, 131.3, 129.6, 114.5, 89.3, 79.7, 71.1, 70.8, 69.3, 68.6, 56.1, 39.2, 30.5.

IR (neat): ν_{max} (cm^{-1}) = 2920, 2853, 1645, 1608, 1574, 1520, 1460, 1441, 1294, 1269, 1246, 1179, 1107, 1032, 1005, 834, 628.

HRMS (ESI) calcd for $C_{18}H_{19}[^{56}Fe]NO$ $[M]^+$: 321.0811. Found: 321.0811.

HPLC The enantiomeric excess was determined by Waters Trefoil CEL 2 (SFC:3.0 mm, I.D.:150 mm), CO_2 / MeOH = 60 / 40, 1.0 mL/min, λ = 214 nm, t (minor) = 3.46 min, t (major) = 4.29 min.

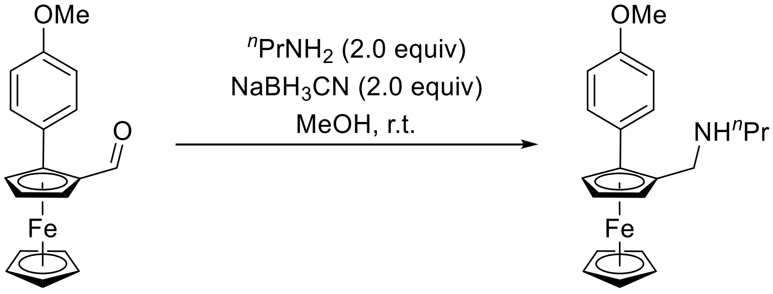

(S_p)-**3aa**, >99% ee **4f**

$NaBH_3CN$ (12.6 mg, 0.2 mmol, 2.0 equiv) was slowly added to a suspension of (S_p)-**3aa** (32.0 mg, 0.1 mmol, 1.0 equiv, >99% ee), *n*-$PrNH_2$ (16 μL, 0.2 mmol, 2.0 equiv) in MeOH (2.0 mL) at 0 °C and then stirred at room temperature. After the reaction was complete (monitored by TLC), it was quenched by adding $NaHCO_3$

solution (aq. 2 M, 5 mL). The mixture was extracted with ethyl acetate (5 mL × 3). The combined organic phases were washed with brine and dried over anhydrous sodium sulfate. After filtration, the solvent was removed under reduced pressure. The residue was purified by silica gel column chromatography (hexane/ethyl acetate = 10/1, v/v, 2% Et_3N) to afford product **4f**.

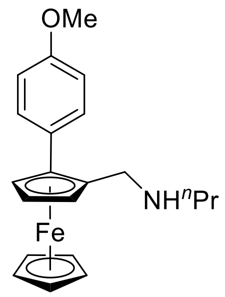

4f. Foamy orange yellow solid (25.8 mg, 71% yield, >99% ee).

Analytical data for **4f**:

$[\alpha]_D^{24}$ = +65.4 (*c* = 0.1, Acetone).

^{1}H NMR (400 MHz, $CDCl_3$) δ 7.53 (d, *J* = 8.8 Hz, 2H), 6.88 (d, *J* = 8.8 Hz, 2H), 4.39 (t, *J* = 2.0 Hz, 1H), 4.35 (t, *J* = 2.0 Hz, 1H), 4.19 (t, *J* = 2.4 Hz, 1H), 4.07 (s, 5H), 3.83 (s, 3H), 3.79 (d, J_{AB} = 12.8 Hz, 1H), 3.67 (d, J_{BA} = 12.8 Hz, 1H), 2.59-2.55 (m, 2H), 2.08 (br, 1H), 1.54-1.43 (m, 2H), 0.88 (t, *J* = 7.4 Hz, 3H).

^{13}C NMR (101 MHz, $CDCl_3$) δ 158.1, 130.4, 130.0, 113.5, 87.4, 84.0, 69.8, 69.4, 69.2, 66.7, 55.2, 51.3, 47.7, 22.8, 11.7.

IR (neat): ν_{max} (cm^{-1}) = 2958, 2930, 2834, 1671, 1611, 1574, 1522, 1457, 1411, 1379, 1289, 1246, 1178, 1105, 1035, 1001, 972, 831, 735.

HRMS (ESI) calcd for $C_{21}H_{25}[^{56}Fe]NO$ $[M]^+$: 363.1280. Found: 363.1276.

HPLC The enantiomeric excess was determined by Daicel Chiralpak OD-H (0.46 cm × 25 cm), Hexanes / IPA = 80 / 20, 0.7 mL/min, λ = 254 nm, t (minor) = 4.92 min, t (major) = 5.56 min.

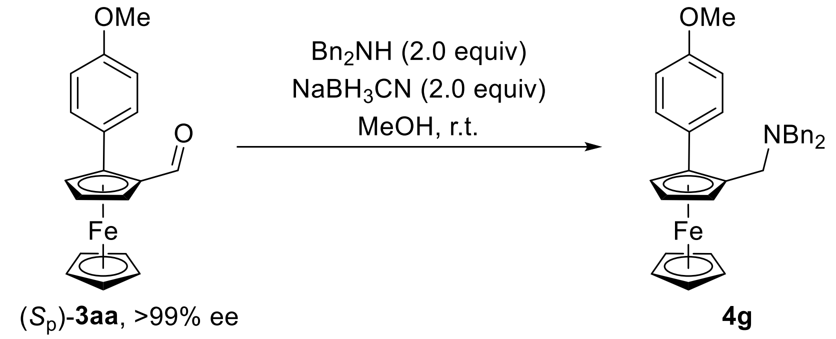

$NaBH_3CN$ (12.6 mg, 0.2 mmol, 2.0 equiv) was slowly added to a suspension of (S_p)-**3aa** (32.0 mg, 0.1 mmol, 1.0 equiv, >99% ee), Bn_2NH (38 μL, 0.2 mmol, 2.0 equiv) in MeOH (2.0 mL) at 0 °C and then stirred at room temperature. After the reaction was complete (monitored by TLC), it was quenched by adding $NaHCO_3$ solution (aq. 2 M, 5 mL). The mixture was extracted with ethyl acetate (5 mL × 3). The combined organic phases were washed with brine and dried over anhydrous sodium sulfate. After filtration, the solvent was removed under reduced pressure. The residue was purified by silica gel column chromatography (hexane/ethyl acetate = 10/1, v/v, 2% Et_3N) to afford product **4g**.

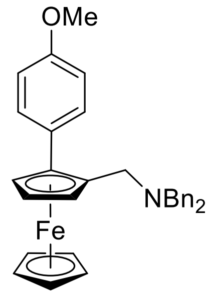

4g. Foamy orange yellow solid (37.6 mg, 75% yield, >99% ee).

Analytical data for **4g**:

$[\alpha]_D^{24}$ = +79.4 (c = 0.1, Acetone).

^{1}H NMR (400 MHz, $CDCl_3$) δ 7.55 (d, J = 8.8 Hz, 2H), 7.30-7.28 (m, 8H), 7.25-7.20 (m, 2H), 6.79 (d, J = 8.8 Hz, 2H), 4.41 (t, J = 2.0 Hz, 1H), 4.39 (d, J = 2.0 Hz, 1H), 4.19 (t, J = 2.4 Hz, 1H), 3.97 (s, 5H), 3.85 (s, 3H), 3.79 (d, J_{AB} = 13.6 Hz, 1H), 3.60 (d, J_{AB} = 13.6 Hz, 2H), 3.51 (d, J_{BA} = 13.6 Hz, 1H), 3.38 (d, J_{BA} = 13.6 Hz, 2H).

^{13}C NMR (101 MHz, $CDCl_3$) δ 157.9, 139.7, 130.7, 130.2, 128.9, 128.1, 126.7, 113.3, 88.0, 82.5, 71.4, 69.9, 68.9, 66.6, 57.7, 55.2, 52.6.

IR (neat): ν_{max} (cm^{-1}) = 2925, 2795, 1647, 1610, 1575, 1522, 1496, 1452, 1359, 1304, 1283, 1244, 1208, 1181, 1144, 1100, 1074, 1029, 1001, 951, 915, 857, 832, 768, 745, 702, 659, 620.

HRMS (ESI) calcd for $C_{32}H_{31}[^{56}Fe]NO$ $[M]^+$: 501.1750. Found: 501.1752.

HPLC The enantiomeric excess was determined by Daicel Chiralcel OD-H (0.46 cm × 25 cm), Hexanes / IPA = 90 / 10, 1.0 mL/min, λ = 254 nm, t (minor) = 4.60 min, t (major) = 5.14 min.

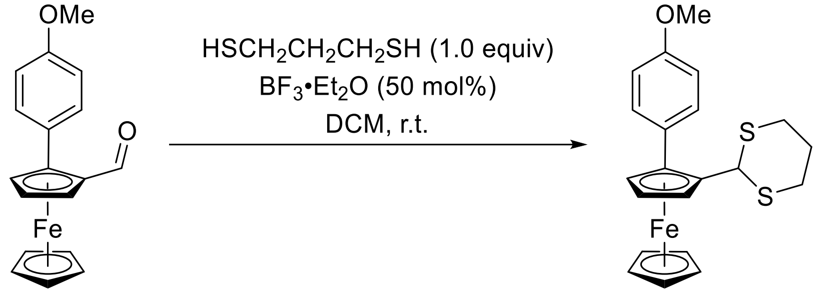

(S_p)-**3aa**, >99% ee   **4h**

BF_3•Et_2O (3 μL, 0.15 mmol, 0.5 equiv) was slowly added to a suspension of (S_p)-**3aa** (96.0 mg, 0.3 mmol, 1.0 equiv, >99% ee), 1,3-dimercaptopropane (60 μL, 0.6 mmol, 2.0 equiv) in DCM (2.0 mL) at 0 °C and then stirred at room temperature. After the reaction was complete (monitored by TLC), it was quenched by adding $NaHCO_3$ solution (aq. 2 M, 5 mL). The mixture was extracted with ethyl acetate (5 mL × 3). The combined organic phases were washed with brine and dried over anhydrous sodium sulfate. After filtration, the solvent was removed under reduced pressure. The residue was purified by silica gel column chromatography (hexane/ethyl acetate = 20/1, v/v) to afford product **4h**.

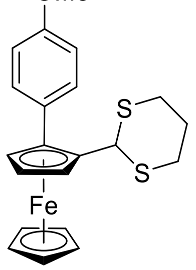

4h. Foamy orange yellow solid (88.6 mg, 72% yield, 99% ee).

Analytical data for **4h**:

$[\alpha]_D^{23}$ = +38.6 (*c* = 0.1, Acetone).

^{1}H NMR (400 MHz, CD_2Cl_2) δ 7.60 (d, *J* = 8.8 Hz, 2H), 6.91 (d, *J* = 8.8 Hz, 2H), 5.06 (s, 1H), 4.46 (t, *J* = 2.0 Hz, 1H), 4.42 (t, *J* = 2.0 Hz, 1H), 4.29 (t, *J* = 2.0 Hz, 1H), 4.22 (s, 5H), 3.83 (s, 3H), 3.08 (ddd, *J* = 16.4, 12.4, 2.4 Hz, 1H), 2.96-2.88 (m, 2H), 2.70 (dt, *J* = 14.4, 4.0 Hz, 1H), 2.12-2.06 (m, 1H), 1.86-1.74 (m, 1H).

^{13}C NMR (101 MHz, CD_2Cl_2) δ 159.2, 131.1, 130.6, 114.3, 87.6, 86.5, 71.4, 69.7, 68.4, 68.0, 56.0, 46.3, 33.4, 33.2, 26.2.

IR (neat): ν_{max} (cm^{-1}) = 1611, 1522, 1453, 1416, 1299, 1274, 1251, 1175, 1105, 1035, 1002, 908, 878, 838, 822, 743, 640.

HRMS (ESI) calcd for $C_{21}H_{22}[^{56}Fe]OS_2$ $[M]^+$: 410.0456. Found: 410.0451.

HPLC The enantiomeric excess was determined by Daicel Chiralcel OD-H (0.46 cm × 25 cm), Hexanes / IPA = 90 / 10, 1.0 mL/min, λ = 254 nm, t (minor) = 6.74 min, t (major) = 8.83 min.

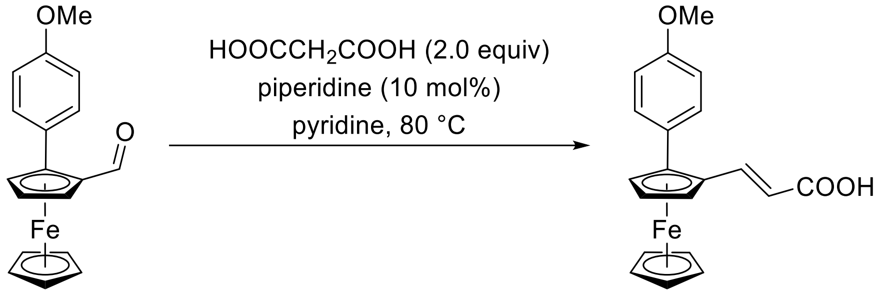

(S_p)-**3aa**, 99% ee **4i**

Piperidine (1 μL, 0.01 mmol, 10 mol%) was added to a suspension of (S_p)-**3aa** (32.0 mg, 0.1 mmol, 1.0 equiv, >99% ee), and malonic acid (20.8 mg, 0.2 mmol, 2.0 equiv) in pyridine (2.0 mL) at room temperature and then stirred at 80 °C. After the reaction was complete (monitored by TLC), it was quenched by adding NH_4Cl solution (aq. 2 M, 5 mL). The mixture was extracted with ethyl acetate (5 mL × 3). The combined organic phases were washed with brine and dried over anhydrous sodium sulfate. After filtration, the solvent was removed under reduced pressure. The residue was purified by silica gel column chromatography (hexane/ethyl acetate = 5/1, v/v) to afford product **4h**.

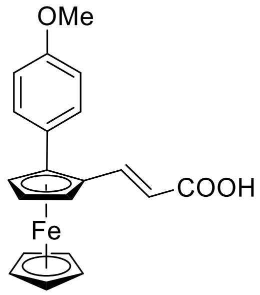

4i. Foamy dark red solid (25.3 mg, 70% yield, >99% ee).

Analytical data for **4i**:

$[\alpha]_D^{24}$ = -548.3 (*c* = 0.1, Acetone).

^{1}H NMR (400 MHz, $CDCl_3$) δ 7.95 (d, *J* = 15.6 Hz, 1H), 7.42 (d, *J* = 8.8 Hz, 2H), 6.92 (d, *J* = 8.8 Hz, 2H), 6.12 (d, *J* = 15.6 Hz, 1H), 4.70 (dd, *J* = 2.8, 1.6 Hz, 1H), 4.65 (dd, *J* = 2.8, 1.6 Hz, 1H), 4.53 (t, *J* = 2.8 Hz, 1H), 4.14 (s, 5H), 3.85 (s, 3H).

^{13}C NMR (101 MHz, $CDCl_3$) δ 172.0, 158.7, 147.5, 130.7, 128.8, 114.2, 113.8, 91.3, 76.2, 72.5, 71.0, 70.0, 66.1, 55.3.

IR (neat): ν_{max} (cm^{-1}) = 2924, 2849, 1674, 1609, 1522, 1435, 1413, 1303, 1274, 1244, 1213, 1181, 1106, 1032, 995, 937, 895, 858, 834, 651.

HRMS (ESI) calcd for $C_{20}H_{18}[^{56}Fe]O_3$ $[M]^+$: 362.0600. Found: 362.0596.

HPLC The enantiomeric excess was determined by Daicel Chiralpak IG (0.46 cm × 25 cm), Hexanes / IPA = 70 / 30, 0.7 mL/min, λ = 254 nm, t (major) = 7.75 min, t (minor) = 11.21 min.

Synthetic utility of product

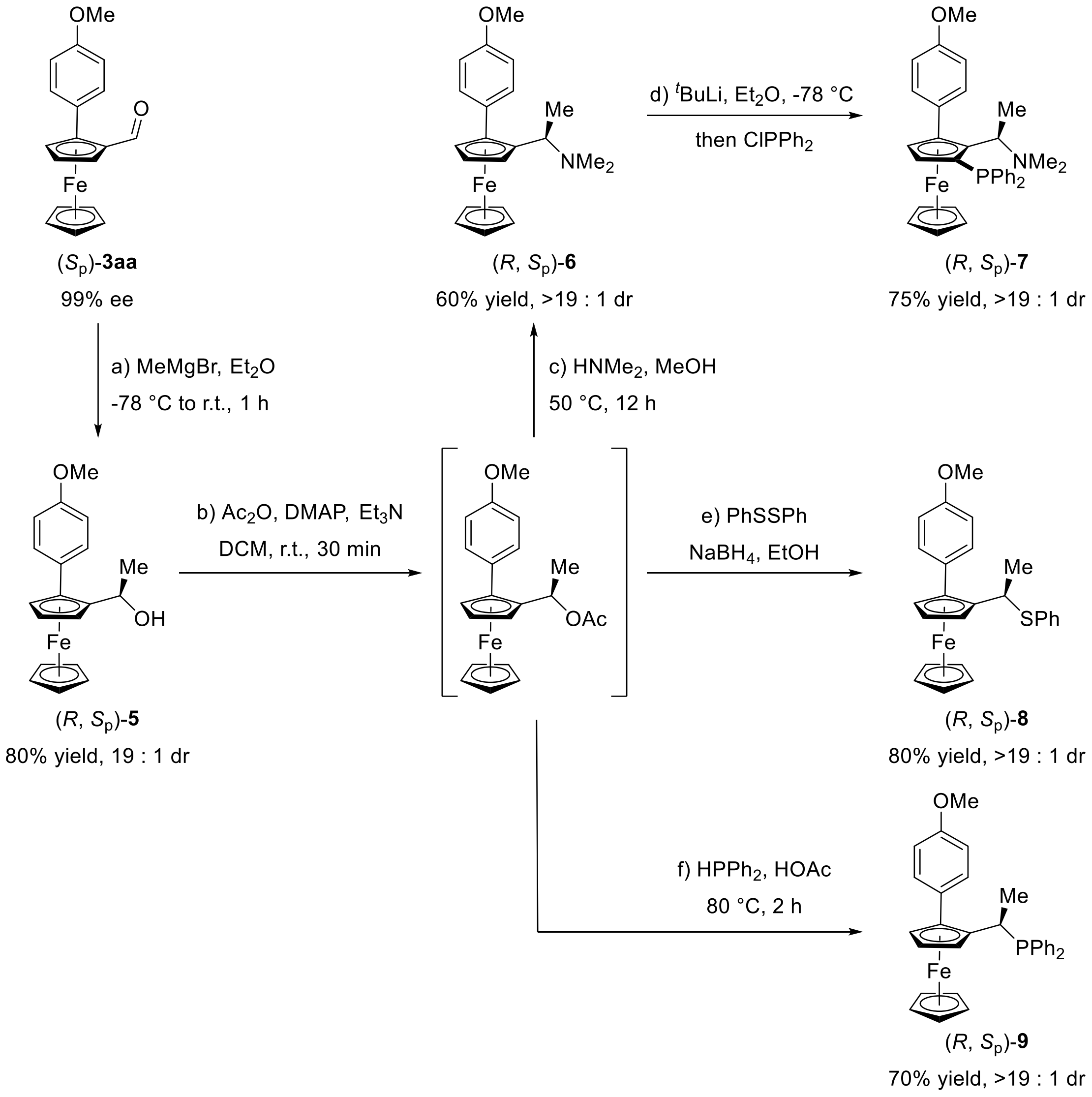

Step a: MeMgBr (3.0 M in THF, 3.0 mL, 9.0 mmol, 3.0 equiv) was slowly added to a suspension of (S_p)-**3aa** (960.0 mg, 3.0 mmol, 1.0 equiv, >99% ee) in Et_2O (10.0 mL) at -78 °C and then stirred at room temperature. After the reaction was complete (monitored by TLC), it was quenched by adding $NaHCO_3$ solution (aq. 2 M, 10 mL). The mixture was extracted with ethyl acetate (10 mL × 3). The combined organic phases were washed with brine and dried over anhydrous sodium sulfate. After filtration, the solvent was removed under reduced pressure. The diastereoselectivity of the reaction was determined by ^{1}H NMR analysis of the crude product. The residue was purified by silica gel column chromatography (hexane/ethyl acetate = 5/1, v/v) to give **5**.

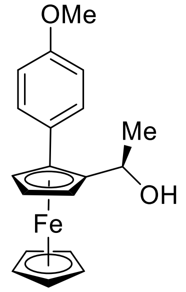

5. Foamy orange yellow solid (806.9 mg, 80% yield, 19:1 dr).

Analytical data for **5**:

$[\alpha]_D^{21}$ = -65.0 (c = 0.1, Acetone).

^{1}H NMR (400 MHz, CD_2Cl_2) δ 7.45 (d, J = 8.4 Hz, 2H), 6.88 (d, J = 8.4 Hz, 2H), 4.81 (q, J = 6.0 Hz, 1H), 4.43 (dd, J = 2.4, 1.6 Hz, 1H), 4.38 (t, J = 2.4 Hz, 1H), 4.26 (d, J = 2.4 Hz, 1H), 4.21 (s, 5H), 3.83 (s, 3H), 2.16 (br, 1H), 1.32 (d, J = 6.4 Hz, 3H).

^{13}C NMR (101 MHz, CD_2Cl_2) δ 159.2, 131.1, 130.7, 114.2, 94.9, 87.9, 70.3, 70.3, 67.5, 66.0, 64.6, 56.0, 25.0.

IR (neat): ν_{max} (cm^{-1}) = 2971, 2932, 2836, 1611, 1573, 1521, 1438, 1411, 1370, 1294, 1245, 1177, 1141, 1104, 1073, 1035, 1002, 955, 927, 889, 829, 736, 704, 653.

HRMS (ESI) calcd for $C_{19}H_{20}[^{56}Fe]O_2$ $[M]^+$: 336.0807. Found: 336.0813.

Step b: Ac_2O (408 mg, 4.0 mmol, 2.0 equiv) was slowly added to a suspension of (R, S_p)-**5** (672.2 mg, 2.0 mmol, 1.0 equiv), DMAP (50.0 mg, 0.4 mmol, 0.2 equiv) and Et_3N (0.56 mL, 4.0 mmol, 2.0 equiv) in DCM (4.0 mL) at room temperature. After the reaction was complete (monitored by TLC), it was quenched by adding H_2O (5 mL). The mixture was extracted with ethyl acetate (10 mL × 3). The combined organic phases were washed with brine and dried over anhydrous sodium sulfate. After filtration, the solvent was removed in vacuo to give a yellow oil, which was used without further purification.

Step c: $HNMe_2$ (2.0 M in THF, 10.0 mL, 20.0 mmol, 10.0 equiv) was slowly added to a suspension of the above acetylated ferrocene compounds in MeOH (10.0 mL) at room temperature and then stirred at 50 °C. After the reaction was complete (monitored by

TLC), it was quenched by adding $NaHCO_3$ solution (aq. 2 M, 10 mL). The mixture was extracted with ethyl acetate (10 mL × 3). The combined organic phases were washed with brine and dried over anhydrous sodium sulfate. After filtration, the solvent was removed under reduced pressure. The diastereoselectivity of the reaction was determined by ^1H NMR analysis of the crude product. The residue was purified by silica gel column chromatography (hexane/ethyl acetate = 10/1, v/v, 2% Et_3N) to give **6**.

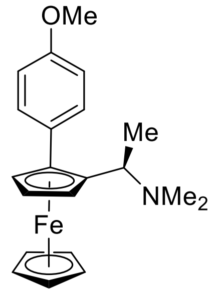

6. Foamy orange yellow solid (450.3 mg, 60% yield, >19:1 dr).

Analytical data for **6**:

$[\alpha]_D^{22}$ = +186.6 (c = 0.25, CH_3CN).

^{1}H NMR (400 MHz, $CDCl_3$) δ 7.60 (d, J = 8.6 Hz, 2H), 6.86 (d, J = 8.4 Hz, 2H), 4.29 (t, J = 1.6 Hz, 1H), 4.20 (t, J = 2.0 Hz, 1H), 4.18 (d, J = 2.4 Hz, 1H), 4.13 (s, 5H), 3.84 (s, 3H), 3.36 (q, J = 6.8 Hz, 1H), 2.35 (s, 6H), 1.12 (d, J = 6.8 Hz, 3H).

^{13}C NMR (101 MHz, $CDCl_3$) δ 158.1, 131.3, 131.2, 113.0, 91.2, 87.7, 70.1, 69.5, 69.1, 65.8, 59.1, 55.2, 43.2, 17.4.

IR (neat): ν_{max} (cm^{-1}) = 2933, 2857, 2815, 2766, 1611, 1574, 1521, 1454, 1368, 1344, 1292, 1245, 1178, 1148, 1105, 1082, 1037, 1003, 967, 931, 815, 729, 640, 613.

HRMS (ESI) calcd for $C_{21}H_{25}[^{56}Fe]NO$ $[M]^+$: 363.1280. Found: 363.1281.

Step d: t-BuLi (1.3 M in pentane, 0.5 mL, 0.65 mmol, 1.5 equiv) was slowly added to a dry flask containing (R, S_p)-**6** (182.0 mg, 0.5 mmol, 1.0 equiv) in Et_2O (2.0 mL) at -78 ℃. The reaction was stirred at this temperature for 2 h, then $ClPPh_2$ (0.18 mL, 1.0 mmol, 2.0 equiv) was added. After 5 min, the mixture was warmed to room temperature and stirred for an additional hour. The reaction was then quenched by adding $NaHCO_3$ solution (aq. 2 M, 2 mL). The mixture was extracted with ethyl acetate (5 mL × 3). The

combined organic phases were washed with brine and dried over anhydrous sodium sulfate. After filtration, the solvent was removed under reduced pressure. The diastereoselectivity of the reaction was determined by ^{1}H NMR analysis of the crude product. The residue was purified by silica gel column chromatography (hexane/ethyl acetate = 20/1, v/v, 2% Et_3N) to give **7**.

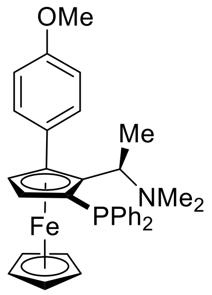

7. Foamy orange yellow solid (206.5 mg, 75% yield, >19:1 dr).

Analytical data for **7**:

$[\alpha]_D^{21}$ = -179.7 (*c* = 0.1, Acetone).

^{1}H NMR (400 MHz, $CDCl_3$) δ 7.60 (d, *J* = 8.8 Hz, 4H), 7.37 (d, *J* = 4.8 Hz, 3H), 7.29 (td, *J* = 7.6, 2.0 Hz, 2H), 7.23 (d, *J* = 6.8 Hz, 3H), 6.84 (d, *J* = 8.4 Hz, 2H), 4.33 (d, *J* = 2.4 Hz, 1H), 4.11-4.03 (m, 1H), 3.99 (s, 5H), 3.94 (d, *J* = 2.4 Hz, 1H), 3.84 (s, 3H), 1.91 (s, 6H), 1.06 (d, *J* = 6.8 Hz, 3H).

^{13}C NMR (101 MHz, $CDCl_3$) δ 158.3, 140.8 (*J* = 7.9 Hz), 139.3 (*J* = 11.2 Hz), 135.5, 135.2, 132.6, 132.4, 132.1, 130.7, 127.9 (*J* = 7.7 Hz), 127.4 (*J* = 6.9 Hz), 127.2, 112.7, 95.8 (*J* = 20.4 Hz), 91.8, 72.0, 70.8, 70.3 (*J* = 5.3 Hz), 58.9 (*J* = 8.0 Hz), 55.2, 41.0, 14.1.

^{31}P NMR (162 MHz, $CDCl_3$) δ -21.8.

IR (neat): ν_{max} (cm^{-1}) = 3053, 2956, 2831, 1663, 1610, 1520, 1436, 1410, 1373, 1289, 1246, 1178, 1107, 1033, 1001, 937, 884, 829, 744, 697, 660, 634, 614.

HRMS (ESI) calcd for $C_{33}H_{35}[^{56}Fe]NOP$ $[M+H]^+$: 548.1800. Found: 548.1807.

Step e: $NaBH_4$ (12.0 mg, 0.3 mmol, 3.0 equiv) was slowly added to the above acetylated ferrocene compounds (0.1 mmol, 1.0 equiv) and diphenyl disulfide (22.0 mg, 0.1 mmol, 1.0 equiv) in EtOH (2.0 mL) at room temperature and then stirred at 80 °C. After the reaction was complete (monitored by TLC), the reaction was quenched by

adding $NaHCO_3$ solution (aq. 2 M, 10 mL). The mixture was extracted with ethyl acetate (5 mL × 3). The combined organic phases were washed with brine and dried over anhydrous sodium sulfate. After filtration, the solvent was removed under reduced pressure. The diastereoselectivity of the reaction was determined by ^1H NMR analysis of the crude product. The residue was purified by silica gel column chromatography (hexane/ethyl acetate = 20/1, v/v) to give **8**.

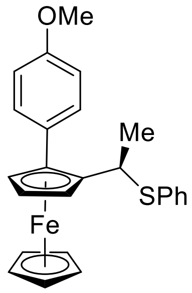

8. Foamy orange yellow solid (34.4 mg, 80% yield, >19:1 dr).

Analytical data for **8**:

$[\alpha]_D^{21}$ = +10.7 (*c* = 0.1, Acetone).

^{1}H NMR (400 MHz, CD_2Cl_2) δ 7.59-7.55 (m, 2H), 7.53-7.49 (m, 2H), 7.38-7.32 (m, 2H), 7.30-7.25 (m, 1H), 6.94-6.89 (m, 2H), 4.44 (q, *J* = 6.8 Hz, 1H), 4.36 (dd, *J* = 2.4, 1.6 Hz, 1H), 4.35 (dd, *J* = 2.4, 1.6 Hz, 1H), 4.26 (t, *J* = 2.4 Hz, 1H), 4.24 (s, 5H), 3.85 (s, 3H), 1.26 (d, *J* = 6.8 Hz, 3H).

^{13}C NMR (101 MHz, CD_2Cl_2) δ 159.2, 137.4, 133.0, 131.5, 131.0, 129.7, 127.7, 114.2, 90.4, 88.4, 71.1, 69.4, 67.8, 67.5, 56.1, 42.9, 25.2.

IR (neat): ν_{max} (cm^{-1}) = 2959, 2921, 2851, 1658, 1633, 1611, 1579, 1521, 1437, 1370, 1293, 1246, 1177, 1105, 1032, 1002, 941, 880, 822, 747, 693.

HRMS (ESI) calcd for $C_{25}H_{24}[^{56}Fe]OS$ $[M]^+$: 428.0892. Found: 428.0900.

Step f: $HPPh_2$ (79.3 mg, 0.4 mmol, 2.0 equiv) was slowly added to the above acetylated ferrocene compound (0.2 mmol, 1.0 equiv) in HOAc (1.0 mL) at room temperature and then stirred at 80 °C. After the reaction was complete (monitored by TLC), the reaction was quenched by adding $NaHCO_3$ solution (aq. 2 M, 10 mL). The mixture was extracted with ethyl acetate (5 mL × 3). The combined organic phases were washed with brine

and dried over anhydrous sodium sulfate. After filtration, the solvent was removed under reduced pressure. The diastereoselectivity of the reaction was determined by ^1H NMR analysis of the crude product. The residue was purified by silica gel column chromatography (hexane/ethyl acetate = 20/1, v/v) to give **9**.

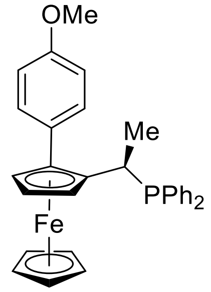

9. Foamy orange yellow solid (70.7 mg, 70% yield, >19:1 dr).

Analytical data for **9**:

$[\alpha]_D^{21}$ = +62.7 (c = 0.1, Acetone).

^{1}H NMR (400 MHz, CD_2Cl_2) δ 7.69 (d, J = 8.4 Hz, 2H), 7.66-7.61 (m, 2H), 7.46-7.39 (m, 5H), 7.33-7.31 (m, 3H), 6.93-6.89 (m, 2H), 4.21 (s, 1H), 4.09 (s, 5H), 4.02 (d, J = 2.8 Hz, 1H), 3.85 (s, 3H), 3.75 (t, J = 2.0 Hz, 1H), 3.61-3.53 (m, 1H), 1.11 (dd, J = 11.5, 7.2 Hz, 3H).

^{13}C NMR (101 MHz, CD_2Cl_2) δ 159.2, 139.5 (J = 15.3 Hz), 138.1 (J = 18.1 Hz), 135.2, 135.0, 134.5, 134.3, 132.3 (J = 4.4 Hz), 131.6, 129.8, 129.3, 129.1 (J = 7.3 Hz), 128.9 (J = 6.8 Hz), 113.8, 91.3 (J = 17.4 Hz), 89.0 (J = 4.3 Hz), 71.1, 70.5, 70.3 (J = 6.5 Hz), 66.6, 56.0, 32.2 (J = 14.5 Hz), 20.7 (J = 13.0 Hz).

^{31}P NMR (162 MHz, CD_2Cl_2) δ -1.6.

IR (neat): ν_{max} (cm^{-1}) = 3054, 2933, 2833, 1609, 1520, 1436, 1372, 1290, 1246, 1178, 1107, 1033, 1001, 937, 828, 744, 697, 656, 637, 613.

HRMS (ESI) calcd for $C_{31}H_{29}[^{56}Fe]OP$ $[M]^+$: 504.1300. Found: 504.1299.

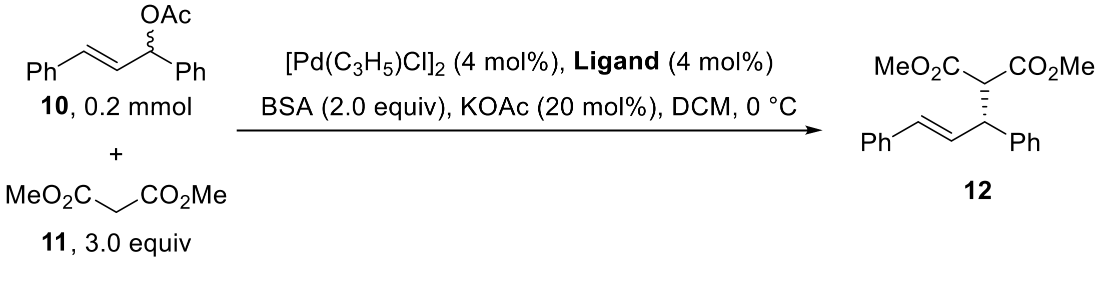

A mixture of ligand (*R*, S_P)-**7** (4.4 mg, 0.008 mmol, 4 mol%) or (*R*, S_P)-**9** (4.0 mg, 0.008 mmol, 4 mol%) and $[Pd(C_3H_5)Cl]_2$ (2.9 mg, 0.008 mmol, 4 mol%) in dry DCM (2.0 mL) was stirred at room temperature for 20 min, Then **10** (50.4 mg, 0.2 mmol, 1.0 equiv) was added. After an additional stirring for 20 min, dimethyl malonate **11** (79.0 mg, 0.6 mmol, 3.0 equiv), BSA (81.4 mg, 0.4 mmol, 2.0 equiv) and KOAc (3.9 mg, 0.04 mmol, 20 mol%) were added. The reaction was stirred at 0 °C. After the reaction was complete (monitored by TLC), the reaction was then quenched by adding NH_4Cl solution (aq. 2 M, 5 mL). The mixture was extracted with ethyl acetate (5 mL × 3). The combined organic phases were washed with brine and dried over anhydrous sodium sulfate. After filtration, the solvent was removed under reduced pressure. The residue was purified by silica gel column chromatography (hexane/ethyl acetate = 50/1, v/v) to afford product **12**.

12. Colorless liquid (61.6 mg, 95% yield, 93% ee). Analytical data for **12**:
$[\alpha]_D^{22}$ = +4.7 (*c* = 1.8, DCM).
^{1}H NMR (400 MHz, $CDCl_3$) *δ* 7.34-7.27 (m, 6H), 7.28-7.26 (m, 1H), 7.26-7.24 (m, 1H), 7.24-7.17 (m, 2H), 6.48 (d, *J* = 15.6 Hz, 1H), 6.33 (dd, *J* = 15.6, 8.8 Hz, 1H), 4.27 (ddd, *J* = 11.2, 8.8, 0.8 Hz, 1H), 3.96 (d, *J* = 11.2 Hz, 1H), 3.70 (s, 3H), 3.52 (s, 3H).
HPLC: The enantiomeric excess was determined by Daicel Chiralpak AD-H (0.46 cm × 25 cm), Hexanes / IPA = 95 / 05, 1.0 mL/min, λ = 254 nm, t (major) = 10.99 min, t (minor) = 14.93 min.
The absolute configuration of product (*R*)-**12** was assigned by comparing the optical rotation with that reported in the literature.[5]

Determination of the absolute configuration of (*R*, *S*p)-6

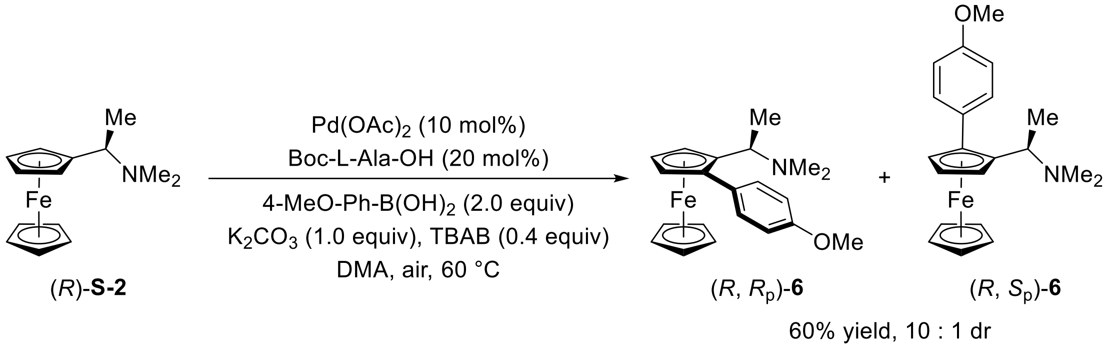

To a solution of 4-methoxyphenylboronic acid (608.0 mg, 4.0 mmol, 2.0 equiv) in DMA (10 mL) at room temperature were added stepwise Boc-L-Ala-OH (76.0 mg, 0.4 mmol, 20 mol%), $Pd(OAc)_2$ (44.9 mg, 0.2 mmol, 10 mmol%), K_2CO_3 (138.2 mg, 2.0 mmol, 1.0 equiv), TBAB (257.9 mg, 0.8 mmol, 0.4 equiv) and (*R*)-**S-2** (516 mg, 2.0 mmol, 1.0 equiv). Then, the reaction mixture was stirred at 60 ℃. After the reaction was complete (monitored by TLC), the reaction was quenched by adding $NaHCO_3$ solution (aq. 2 M, 10 mL). The mixture was extracted with ethyl acetate (20 mL × 3). The combined organic phases were washed with brine and dried over anhydrous sodium sulfate. After filtration, the solvent was removed under reduced pressure. The diastereoselectivity of the reaction was determined by ^{1}H NMR analysis of the crude product. The residue was purified by silica gel column chromatography (hexane/ethyl acetate = 10/1, v/v, 2% Et_3N) to give **6**.

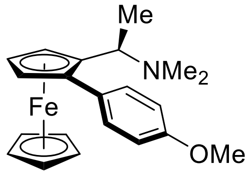

(*R*, *R*p)-**6**

(*R*, *R*p)-**6**. Foamy orange yellow solid (396.1 mg, 54.5% yield).

Analytical data for (*R*, *R*p)-**6**:

$[\alpha]_D^{21}$ = -67.8 (*c* = 0.25, CH_3CN).

^{1}H NMR (400 MHz, $CDCl_3$) δ 7.54 (dd, J = 8.4, 1.6 Hz, 2H), 6.87 (dd, J = 8.4, 1.6 Hz, 2H), 4.39 (dt, J = 2.8, 1.6 Hz, 1H), 4.26 (q, J = 2.0 Hz, 1H), 4.21 (dt, J = 2.8, 1.6 Hz, 1H), 4.08 (s, 5H), 3.86 (q, J = 6.8 Hz, 1H), 3.83 (d, J = 1.5 Hz, 3H), 1.91 (s, 6H), 1.57 (d, J = 6.8 Hz, 3H).

The absolute configuration of product (R, R_p)-**6** was assigned by comparing the **^{1}H NMR** with that of the known compound in the literature.[6]

^{1}H NMR of (R, R_p)-**6**:

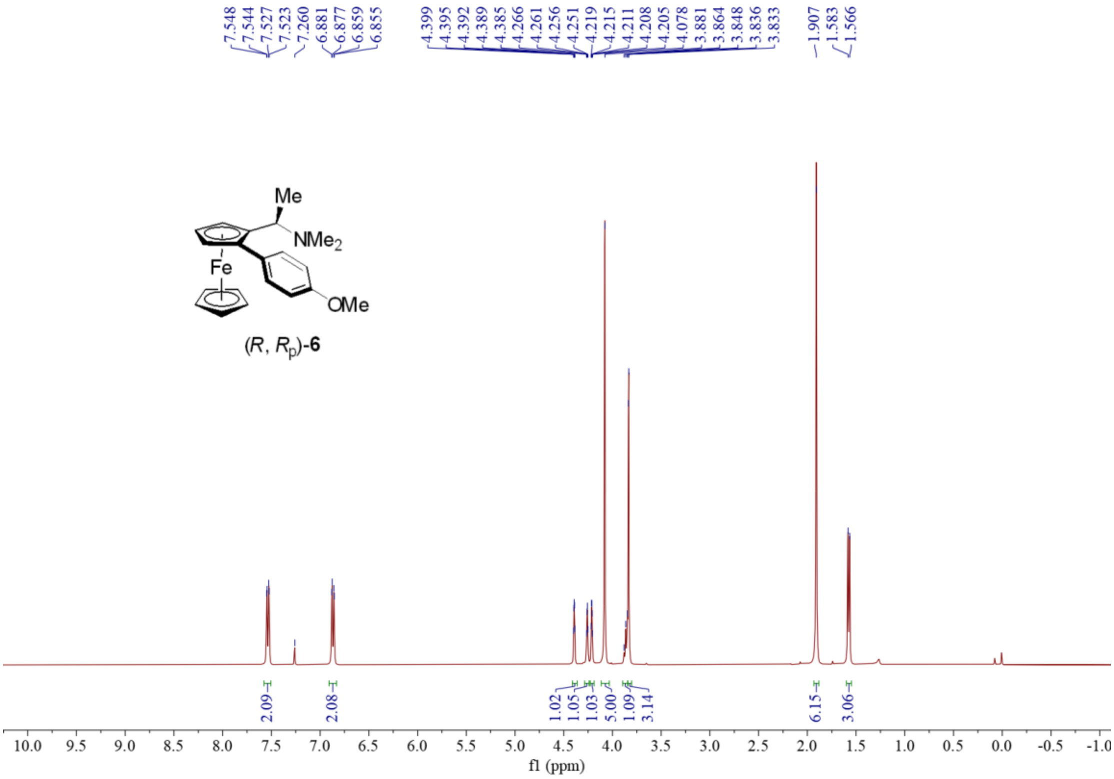

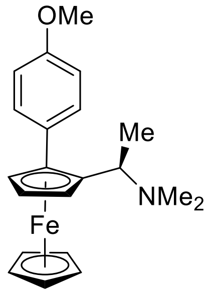

(R, S_p)-**6**

(R, S_p)-**6**. Foamy orange yellow solid (39.0 mg, 5.4% yield).

Analytical data for (R, S_p)-**6**:

$[\alpha]_D^{22}$ = +131.5 (c = 0.25, CH_3CN).

^{1}H NMR (400 MHz, $CDCl_3$) δ 7.60 (d, *J* = 8.8 Hz, 2H), 6.86 (d, *J* = 8.8 Hz, 2H), 4.29 (t, *J* = 2.4 Hz, 1H), 4.20 (d, *J* = 2.4 Hz, 1H), 4.18 (dt, *J* = 2.4, 0.8 Hz, 1H), 4.14 (s, 5H), 3.83 (s, 3H), 3.37 (q, *J* = 6.8 Hz, 1H), 2.36 (s, 6H), 1.13 (d, *J* = 6.8 Hz, 3H).

^{1}H NMR of (*R*, *S*$_p$)-**6**:

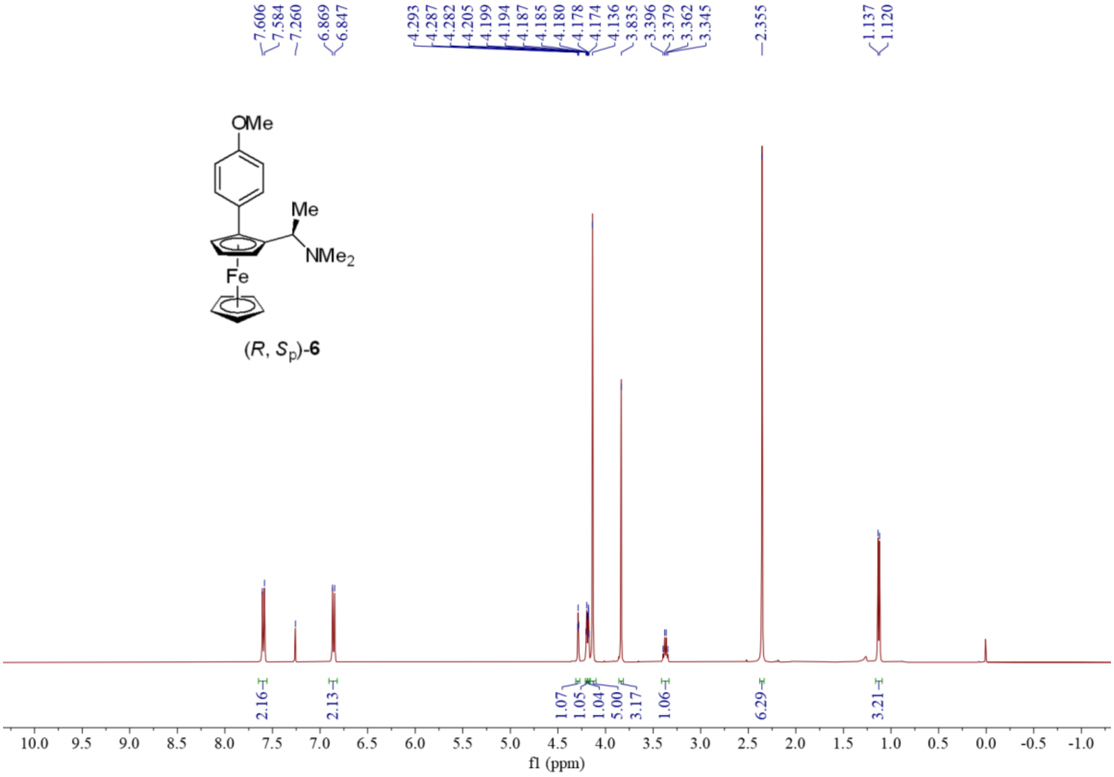

General procedure for H/D KIE experiments

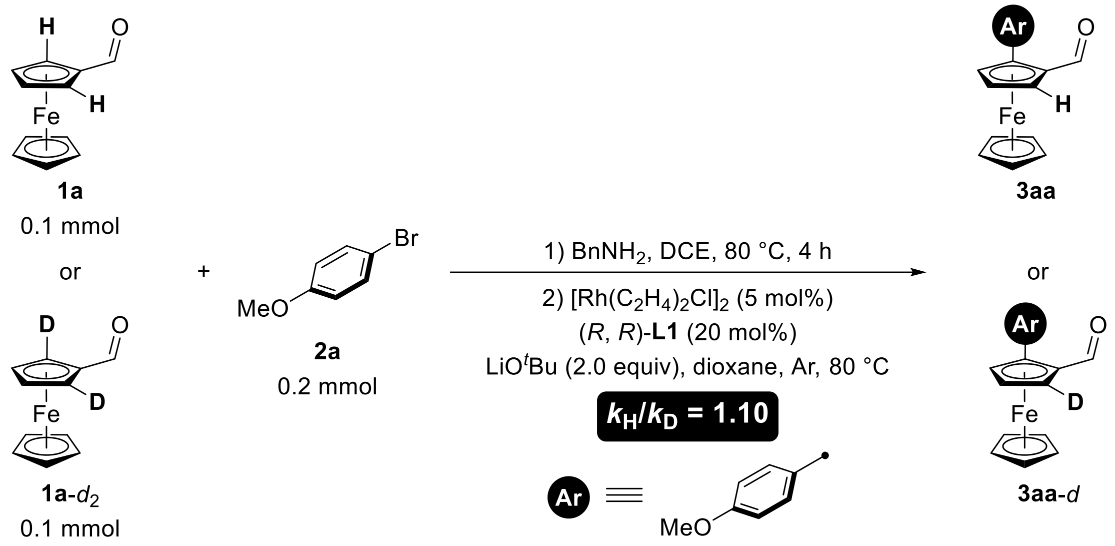

In 4 parallel experiments, ferrocene substrate **1a**-d_2 (0.1 mmol, 1.0 equiv) was added to a dry sealed tube under argon. Then, the tube was evacuated and backfilled with argon for 3 times, and followed by adding DCE (1.0 mL) and $BnNH_2$ (24 µL, 0.22 mmol, 1.1 equiv). The mixture was stirred at 80 °C in 4 h. The solvent was removed directly under vacuum. Next, LiO^{t}Bu (32.0 mg, 0.4 mmol), (*R*, *R*)-**L1** (21.6 mg, 0.04 mmol), $[Rh(C_2H_4)_2Cl]_2$ (3.9 mg, 0.01 mmol) and 4-bromoanisole **2a** (0.4 mmol, 2.0 equiv) were added. The mixture was stirred at 80 °C. After every 30 min, one reaction mixture was quenched by adding water, filtered with diatomaceous earth, concentrated under reduced pressure with a rotary evaporator, and subjected to ^{1}H NMR to determine the conversion of **1a**-d_2. Similar parallel experiments were performed in another 4 dry sealed tubes with **1a** (26.3 mg, 0.1 mmol) instead of **1a**-d_2. The plots of the yields of **3aa**-d_2 and **3aa** depending on the reaction time are shown in Figure S1, based on which the parallel H/D KIE value was calculated.

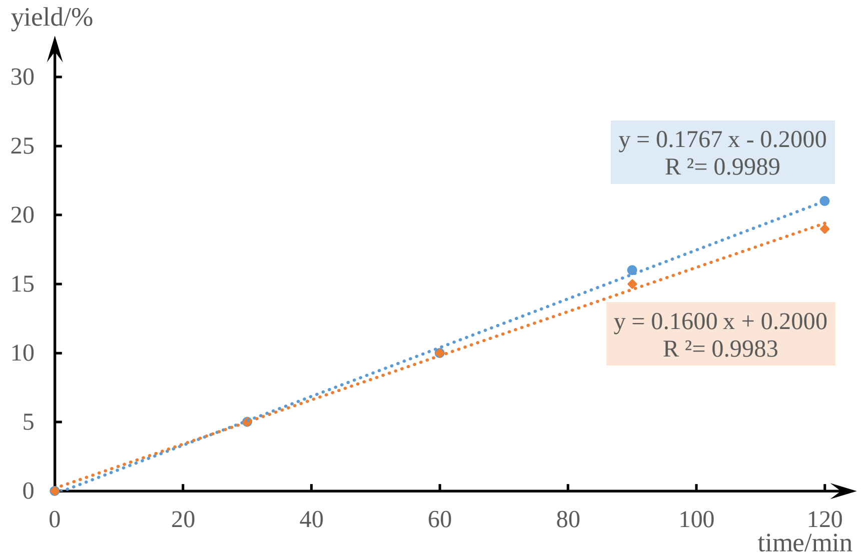

Figure S1. The plots of the yields of **3aa**-d_2 and **3aa** depending on the reaction time (blue, **3aa**; orange, **3aa**-d).

X-Ray crystal structure of 3aa

A single crystal of (S_p)-**3aa** (CCDC 2253651) was obtained through slow evaporation from its solution in ethyl acetate. The structure and absolute configuration of **3aa** were then determined by X-ray crystallographic analysis.

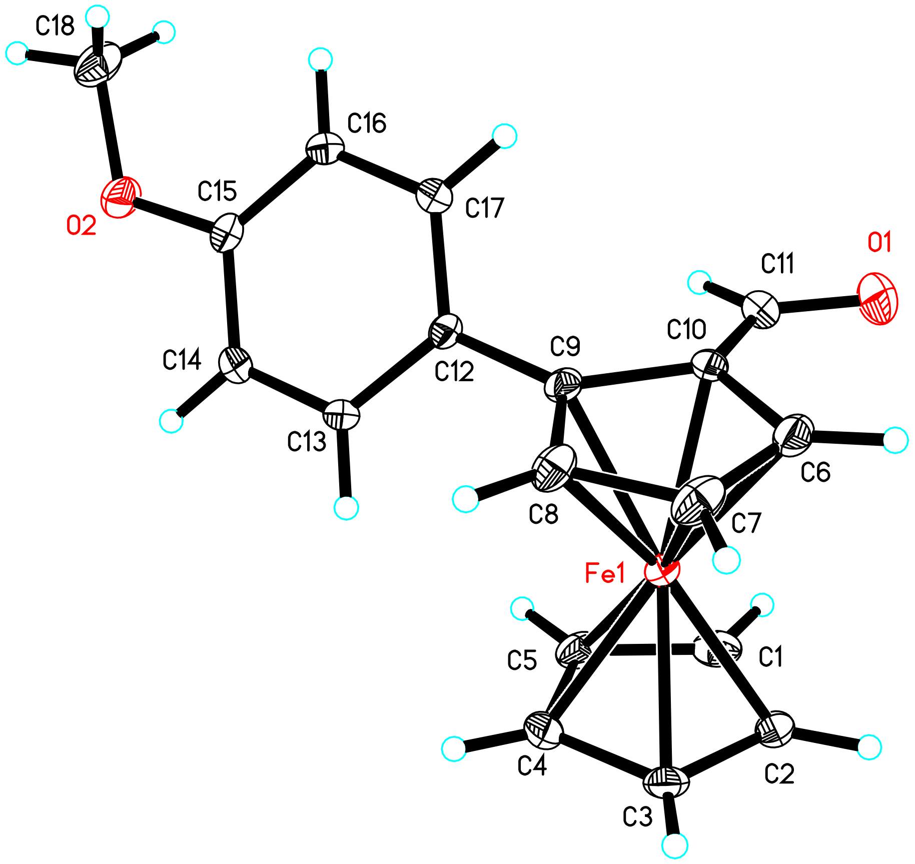

Table S1. Crystal data and structure refinement for mo_d8v22115_0m.

Identification code	mo_d8v22115_0m	
Empirical formula	C18 H16 Fe O2	
Formula weight	320.16	
Temperature	213(2) K	
Wavelength	0.71073 Å	
Crystal system	Orthorhombic	
Space group	P 21 21 21	
Unit cell dimensions	a = 9.3803(3) Å	α= 90 °.
	b = 9.7528(3) Å	β= 90 °.
	c = 15.5830(5) Å	γ = 90 °.
Volume	1425.60(8) Å^3	
Z	4	
Density (calculated)	1.492 Mg/m^3	
Absorption coefficient	1.058 mm^{-1}	
F(000)	664	
Crystal size	0.190 x 0.150 x 0.060 mm^3	
Theta range for data collection	2.464 to 25.986 °.	
Index ranges	-10<=h<=11, -12<=k<=11, -16<=l<=19	
Reflections collected	7062	
Independent reflections	2778 [R(int) = 0.0502]	
Completeness to theta = 25.242 °	99.3 %	
Absorption correction	Semi-empirical from equivalents	
Max. and min. transmission	0.7456 and 0.4750	
Refinement method	Full-matrix least-squares on F^2	
Data / restraints / parameters	2778 / 0 / 193	
Goodness-of-fit on F^2	1.061	
Final R indices [I>2sigma(I)]	R1 = 0.0265, wR2 = 0.0655	
R indices (all data)	R1 = 0.0276, wR2 = 0.0665	

Absolute structure parameter	0.04(2)
Extinction coefficient	0.012(3)
Largest diff. peak and hole	0.270 and -0.212 e.Å^{-3}

References

1. Greßies, S.; Klauck, F. J. R.; Kim, J. H.; Daniliuc, C. G.; Glorius, F. *Angew. Chem. Int. Ed.* **2018**, *57*, 9950–9954.
2. Feringa, B. L.; Pineschi, M.; Arnold, L. A.; Imbos, R.; de Vries, A. H. M. *Angew. Chem. Int. Ed. Engl.* **1997**, *36*, 2620–2623.
3. Fu, Y.; Hou, G.-H.; Xie, J.-H.; Xing, L.; Wang, L.-X.; Zhou, Q.-L. *J. Org. Chem.* **2004**, *69*, 8157–8160.
4. Liang, H.; Vasamsetty, L.; Li, T.; Jiang, J.; Pang, X.; Wang, J. *Chem. Eur. J.* **2020**, *26*, 14546–14550.
5. Du, X.; Liu, H.; Du, D.-M. *Eur. J. Org. Chem.* **2011**, 786–793.
6. Plevová, K.; Mudráková, B.; Rakovský, E.; Šebesta, R. *J. Org. Chem.* **2019**, *84*, 7312−7319.

Copies of NMR spectra and HPLC chromatograms

^{1}H NMR spectra of 1d

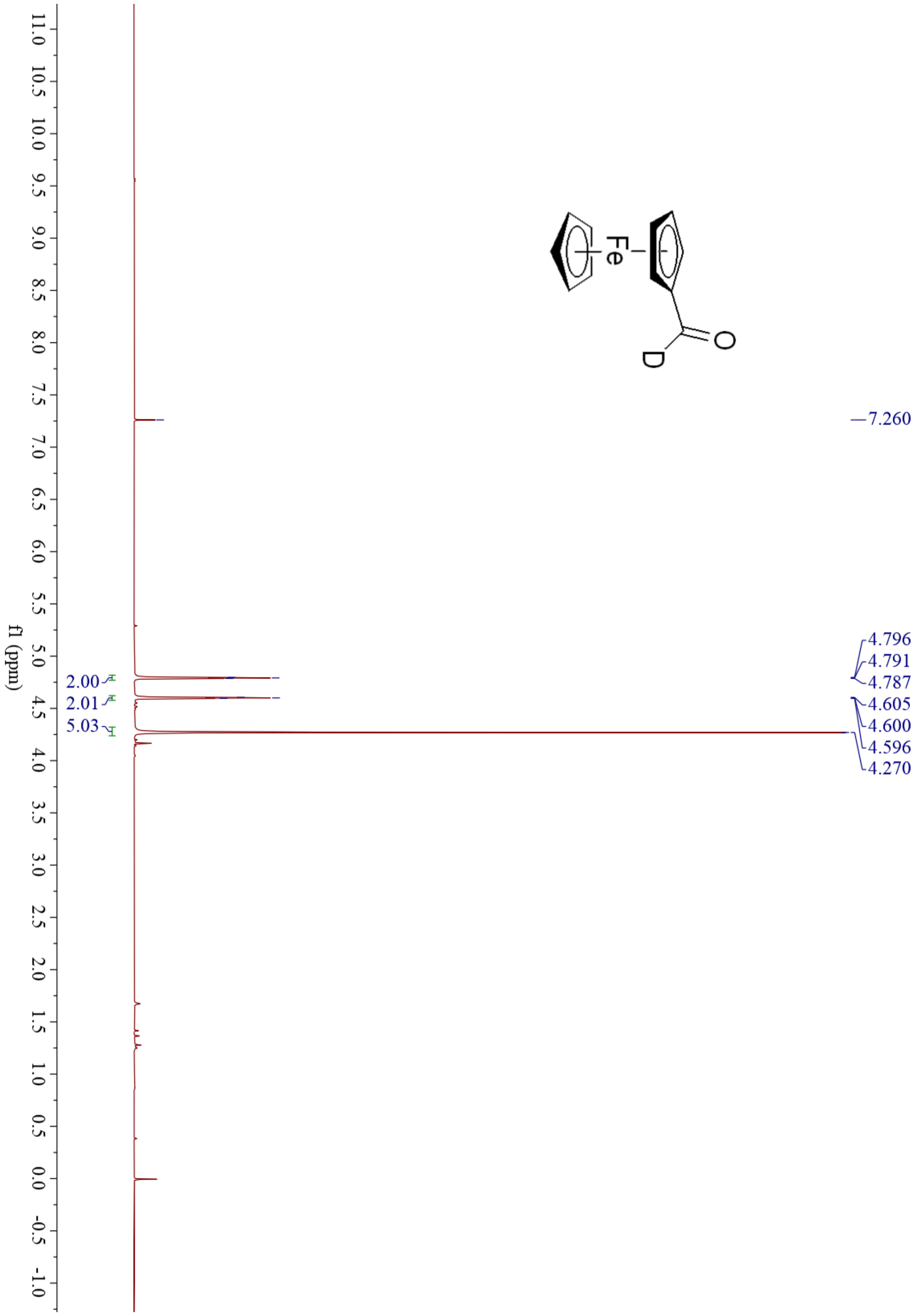

^{13}C NMR spectra of 1d

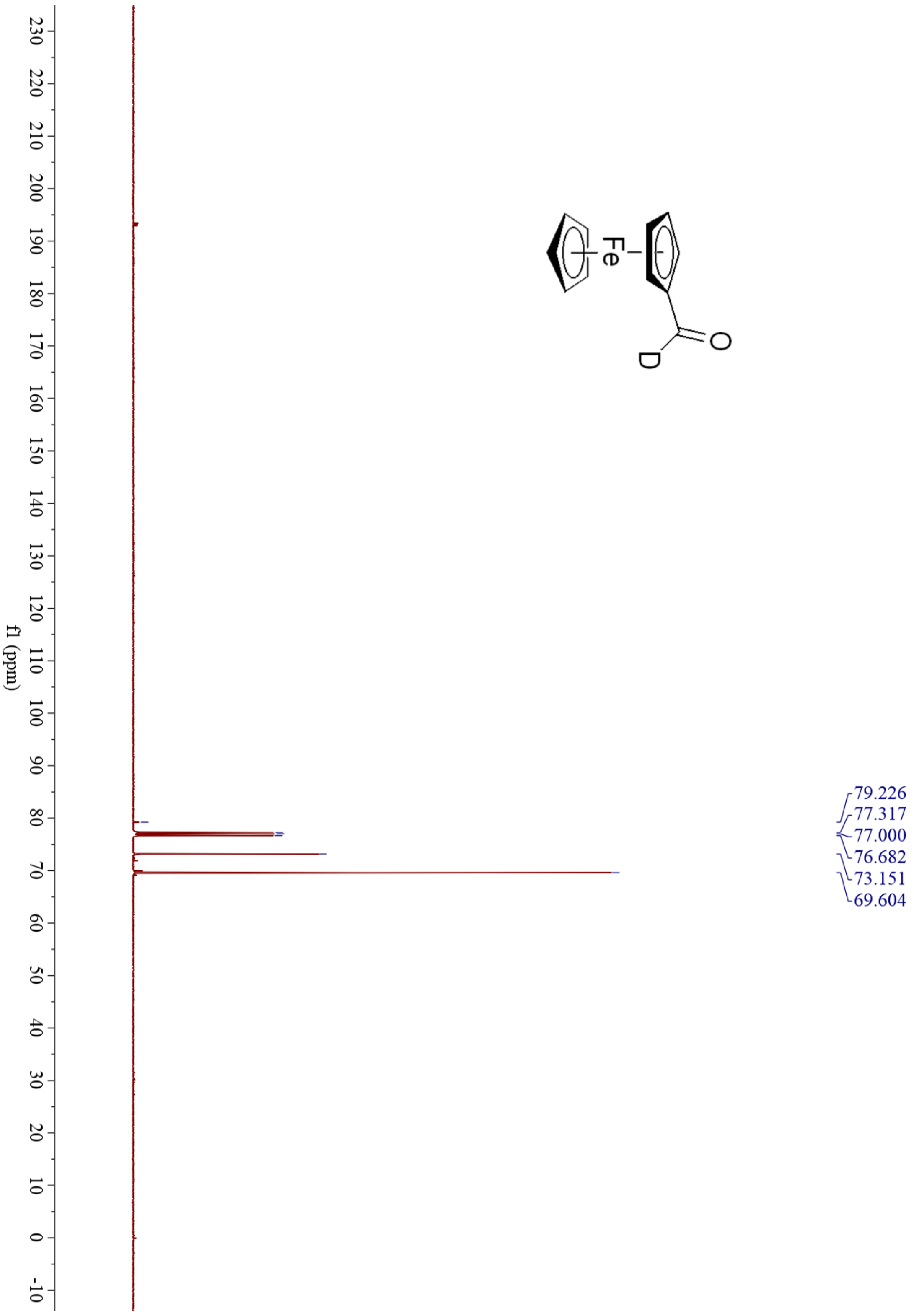

^{1}H NMR spectra of 1f

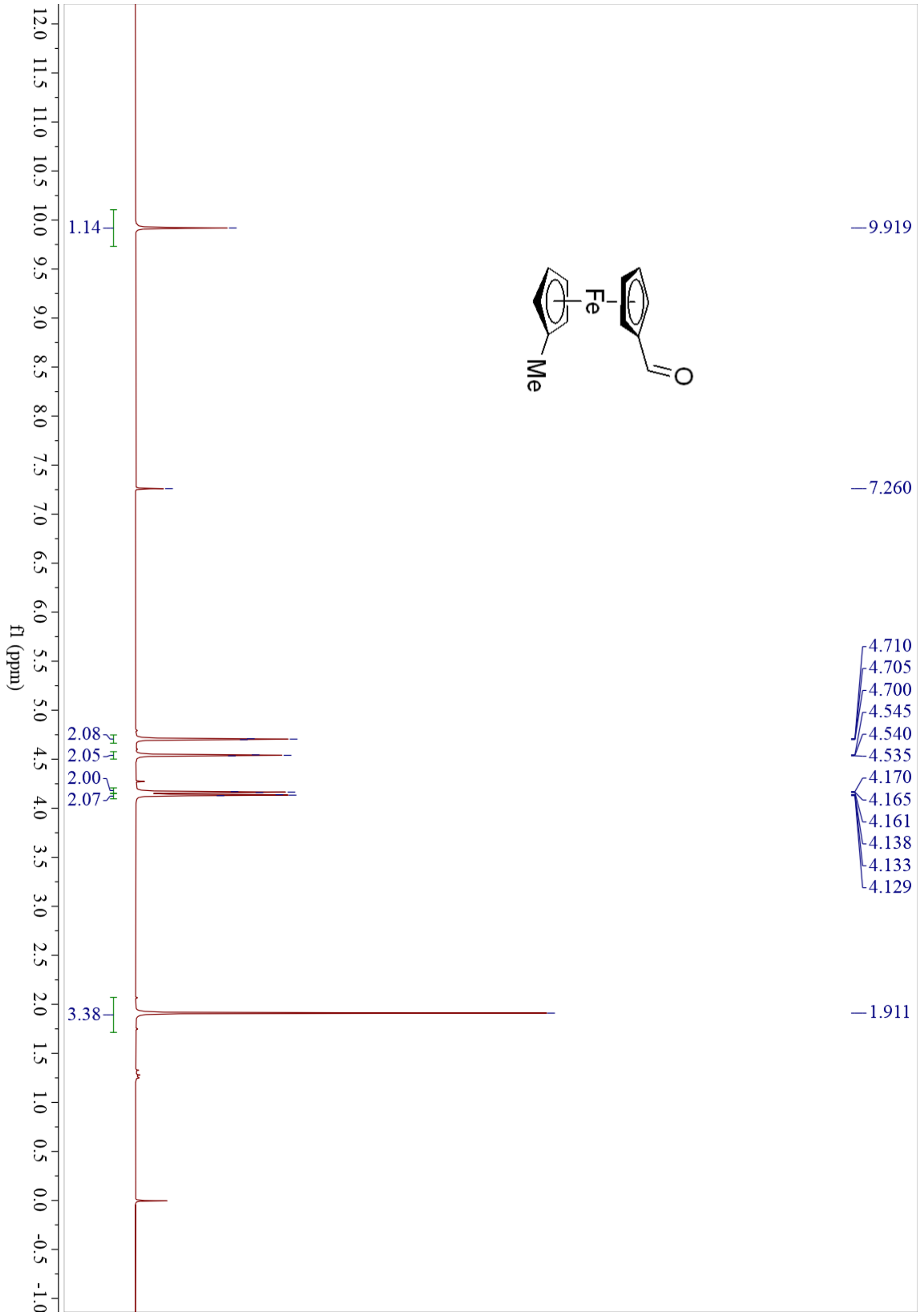

^{13}C NMR spectra of 1f

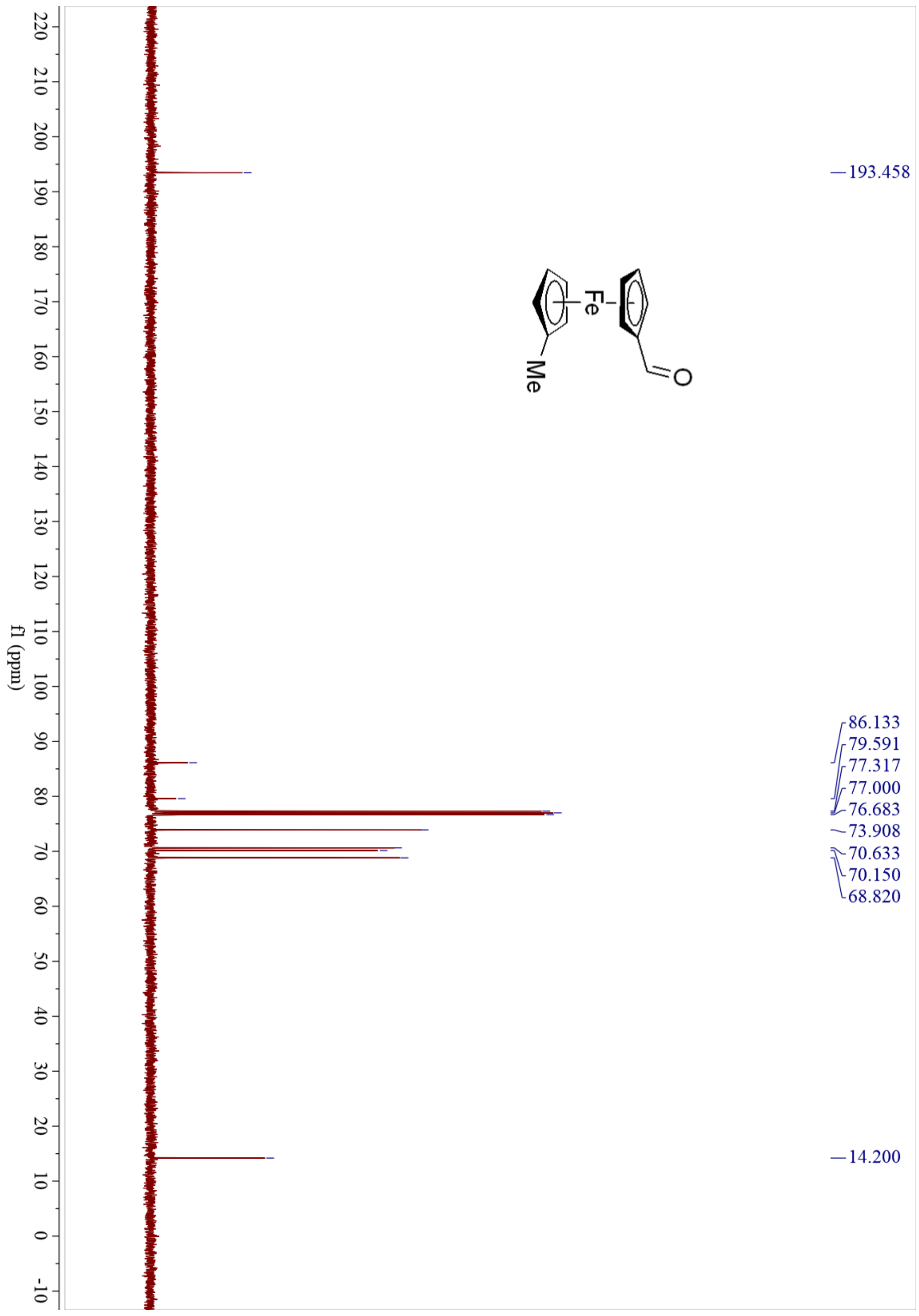

^{1}H NMR spectra of 1g

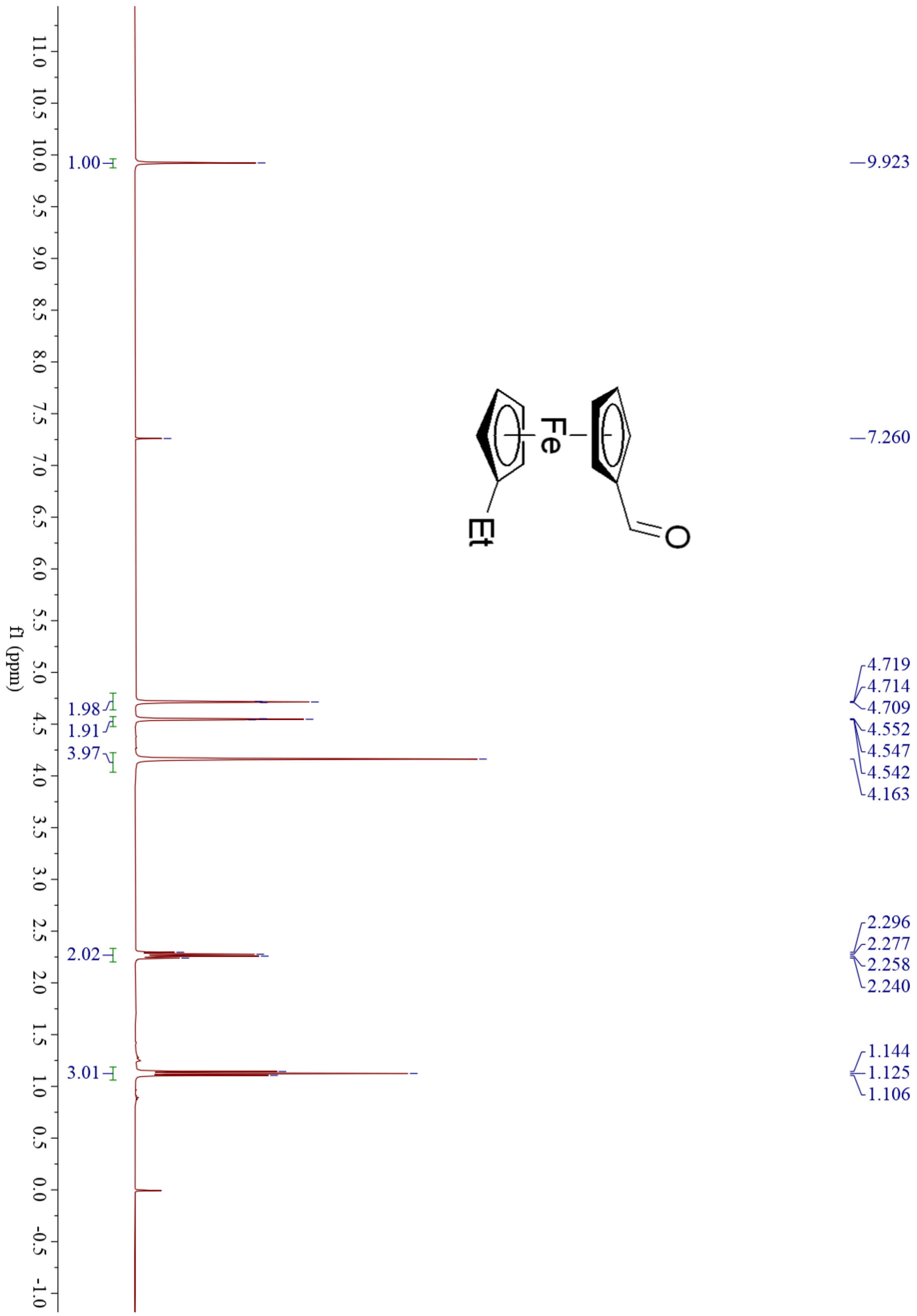

^{13}C NMR spectra of 1g

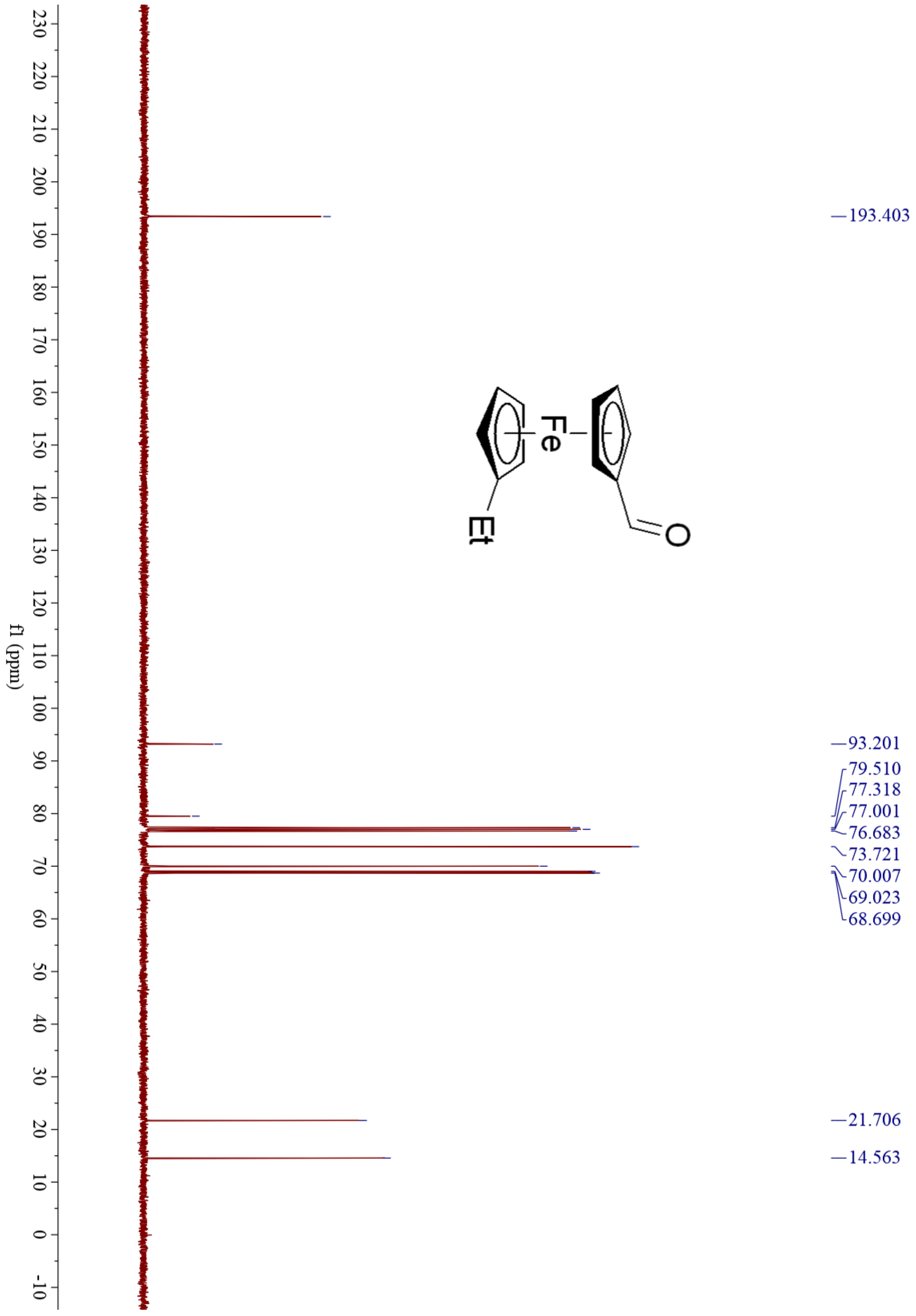

^{1}H NMR spectra of 1h

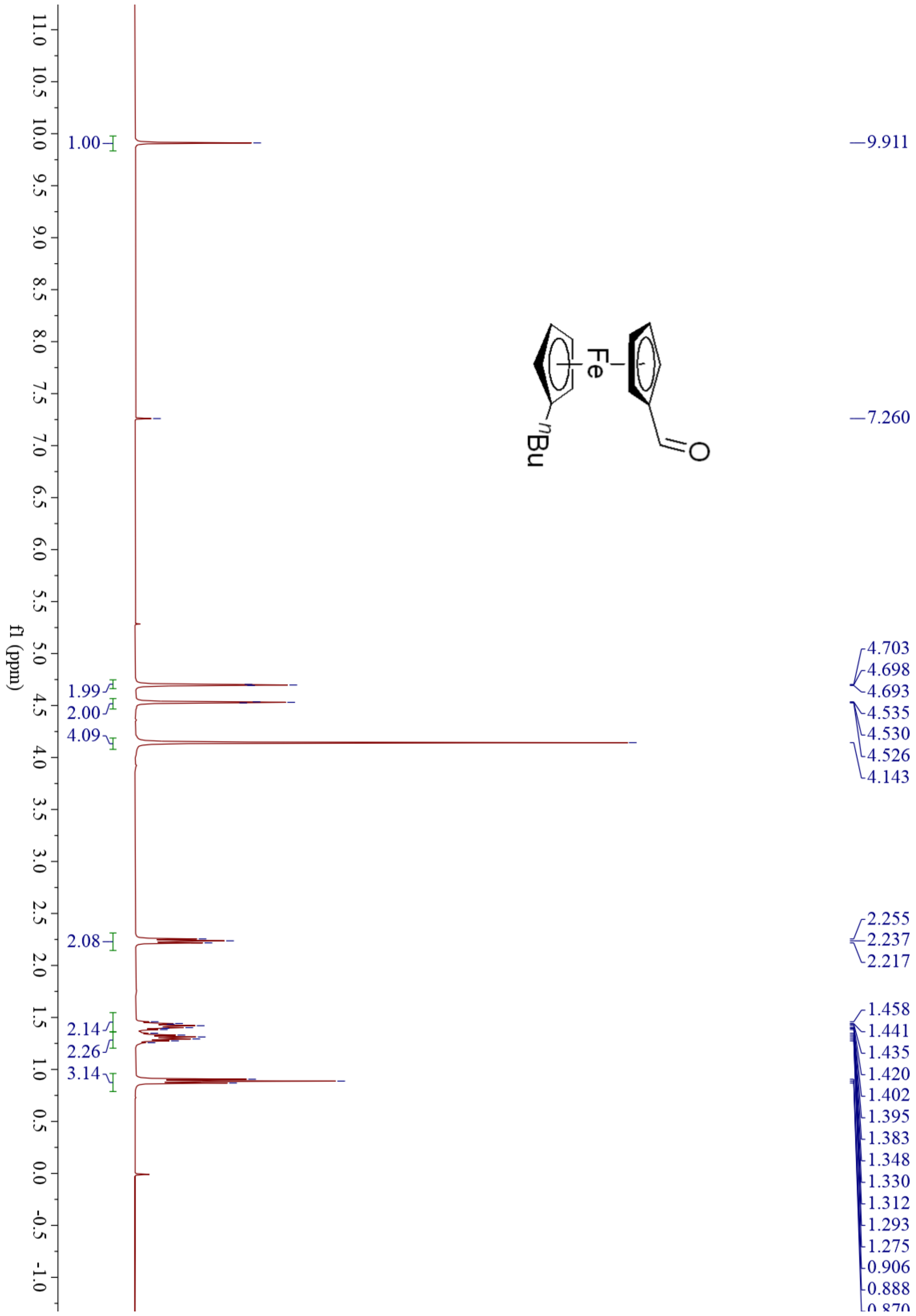

^{13}C NMR spectra of 1h

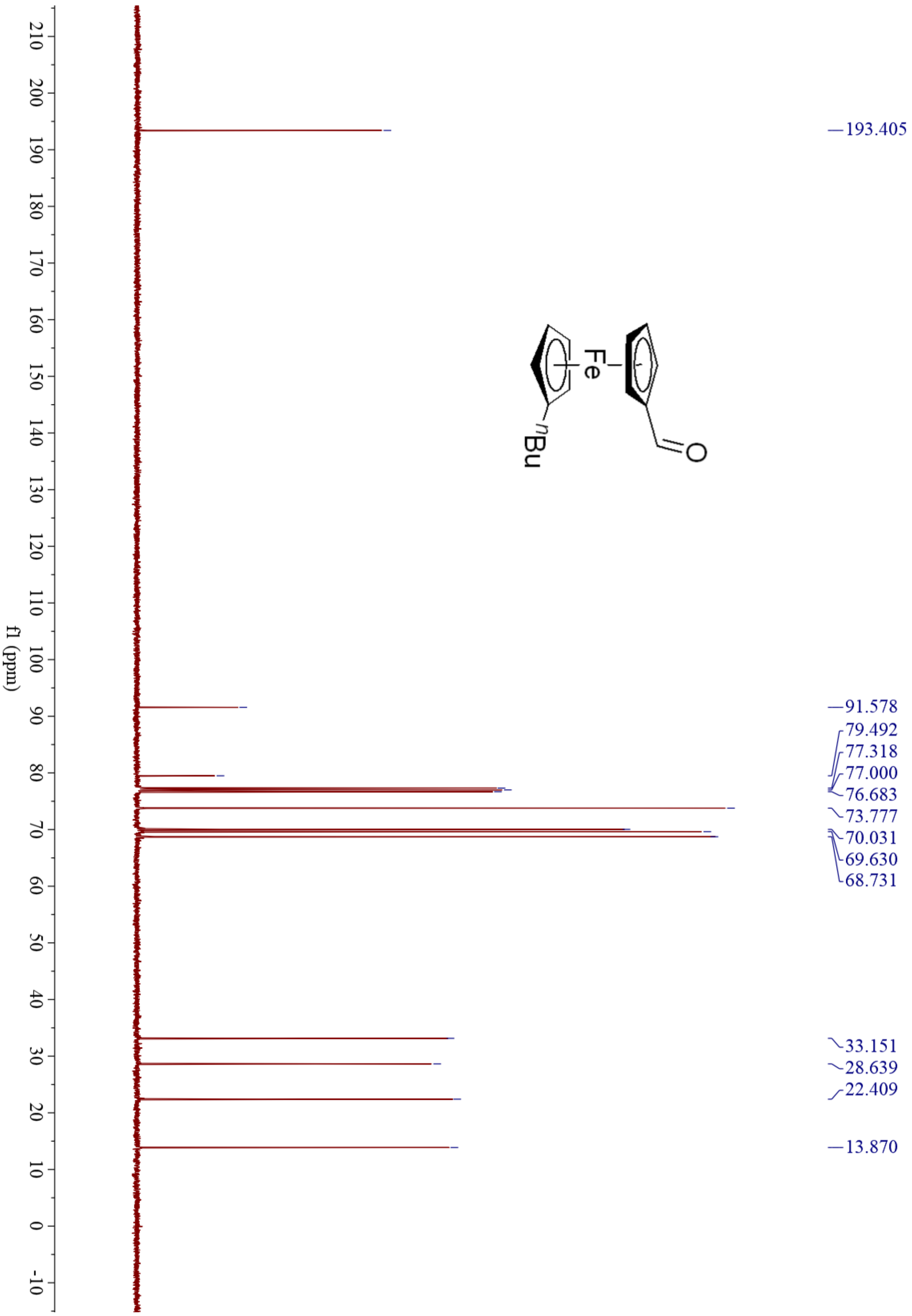

^{1}H NMR spectra of 1i

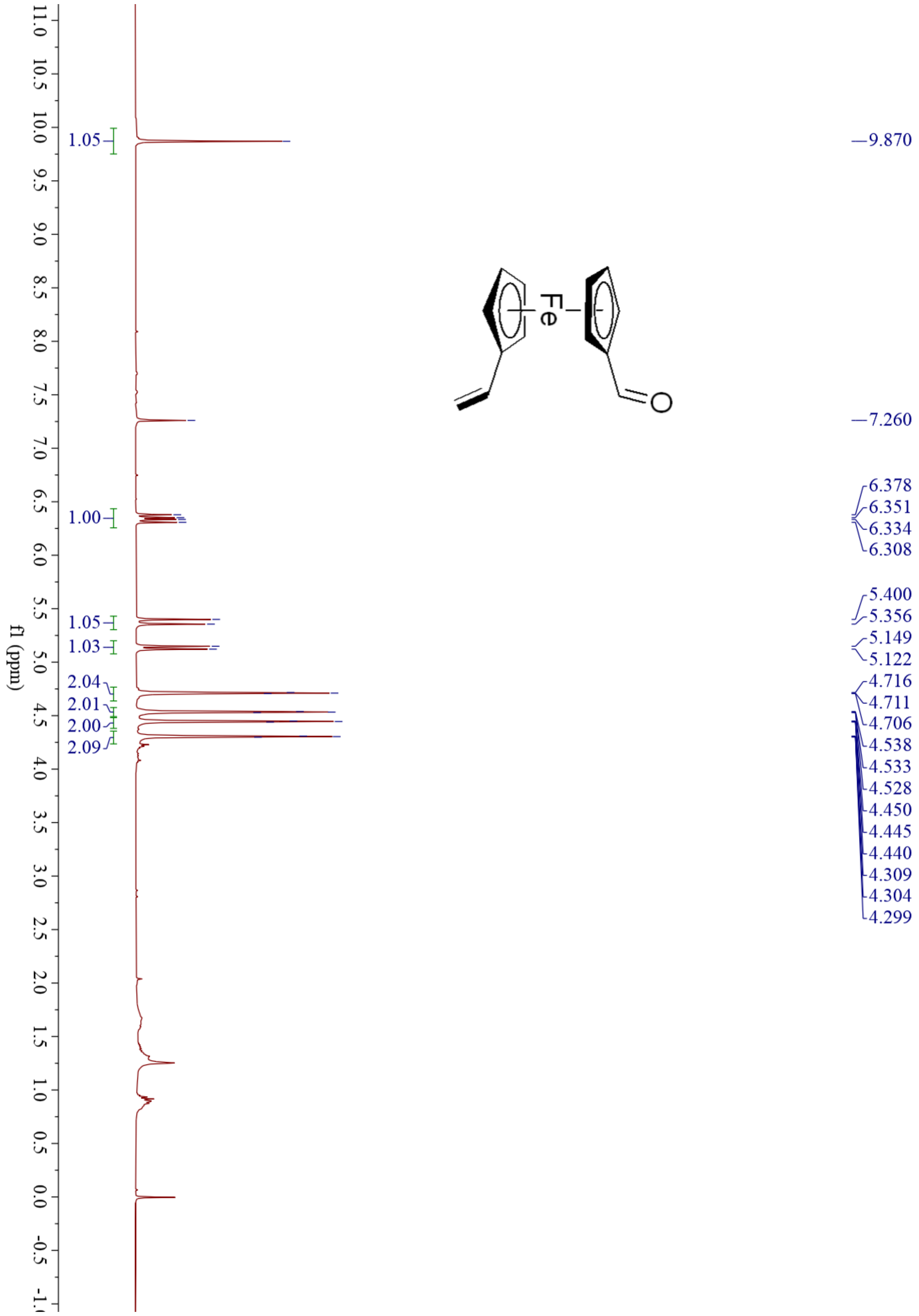

^{13}C NMR spectra of 1i

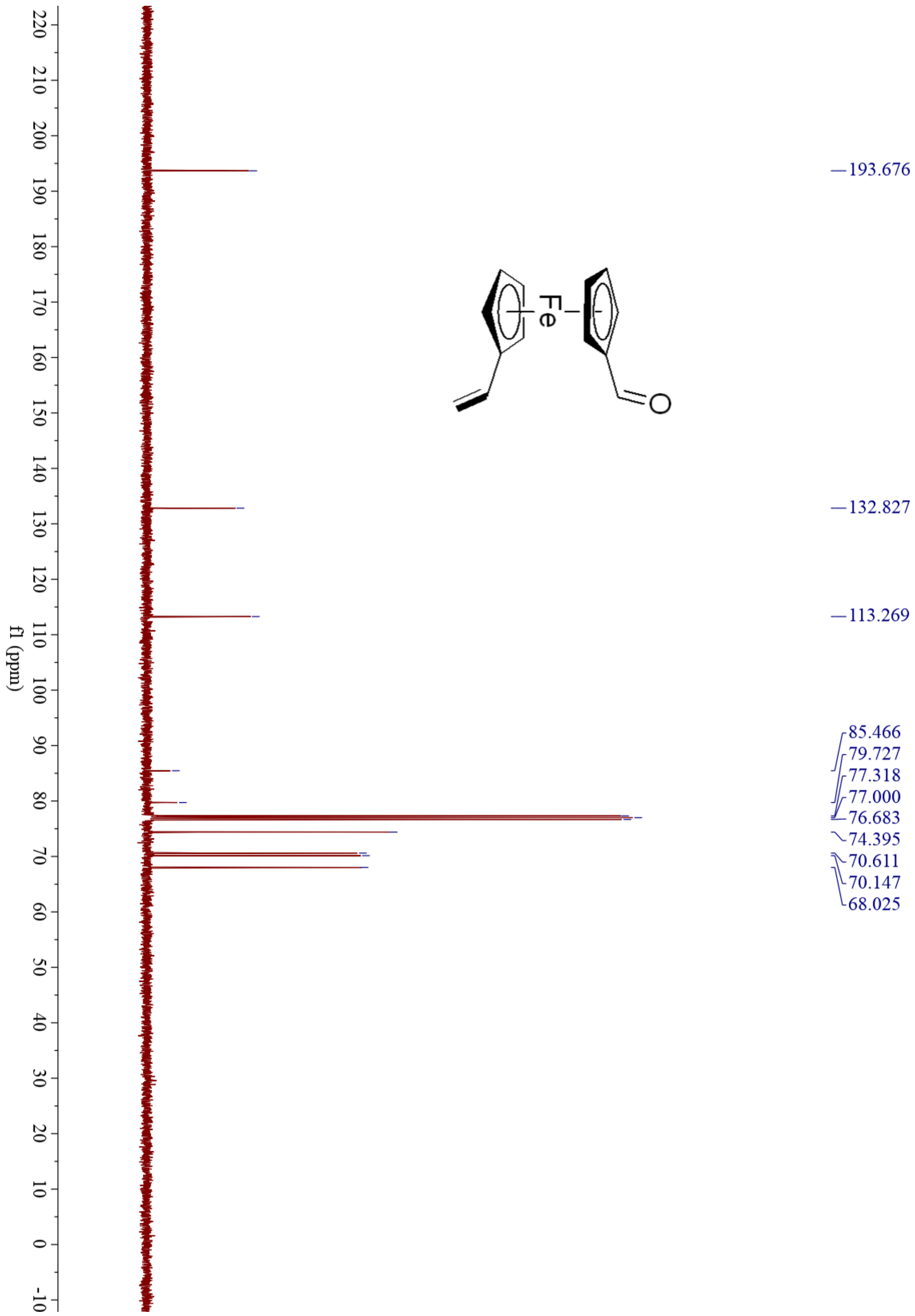

^{1}H NMR spectra of 1j

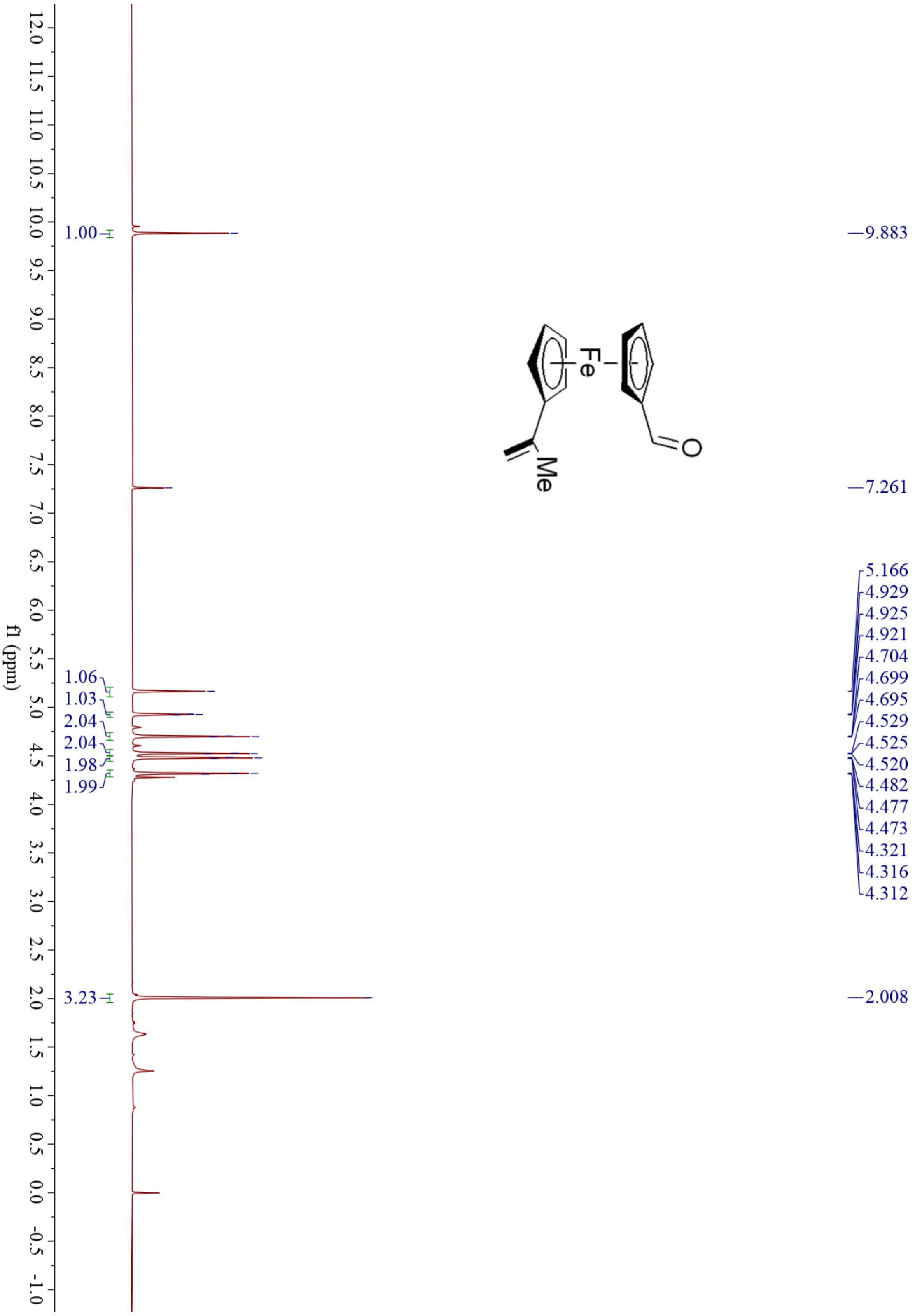

^{13}C NMR spectra of 1j

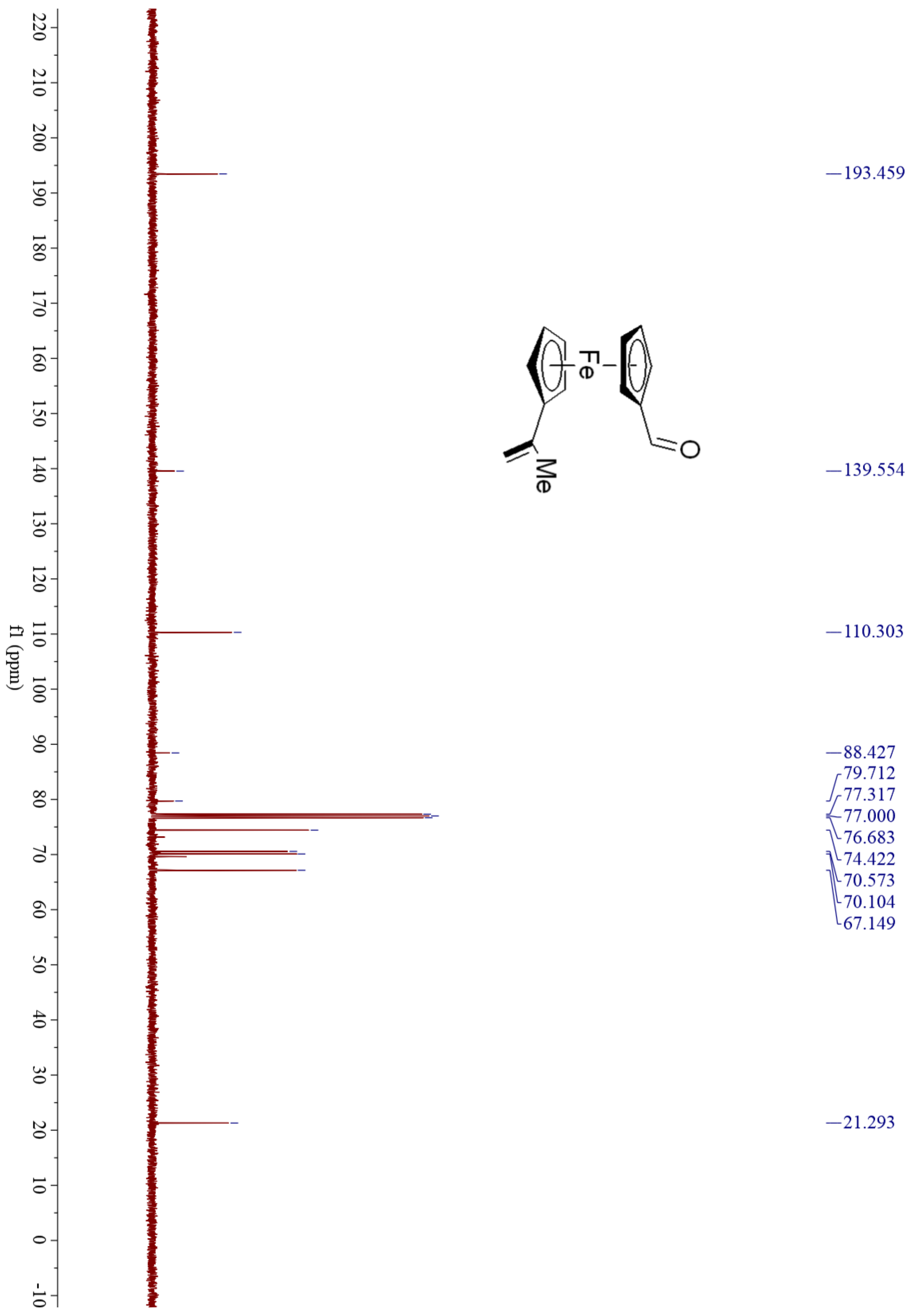

^{1}H NMR spectra of 1k

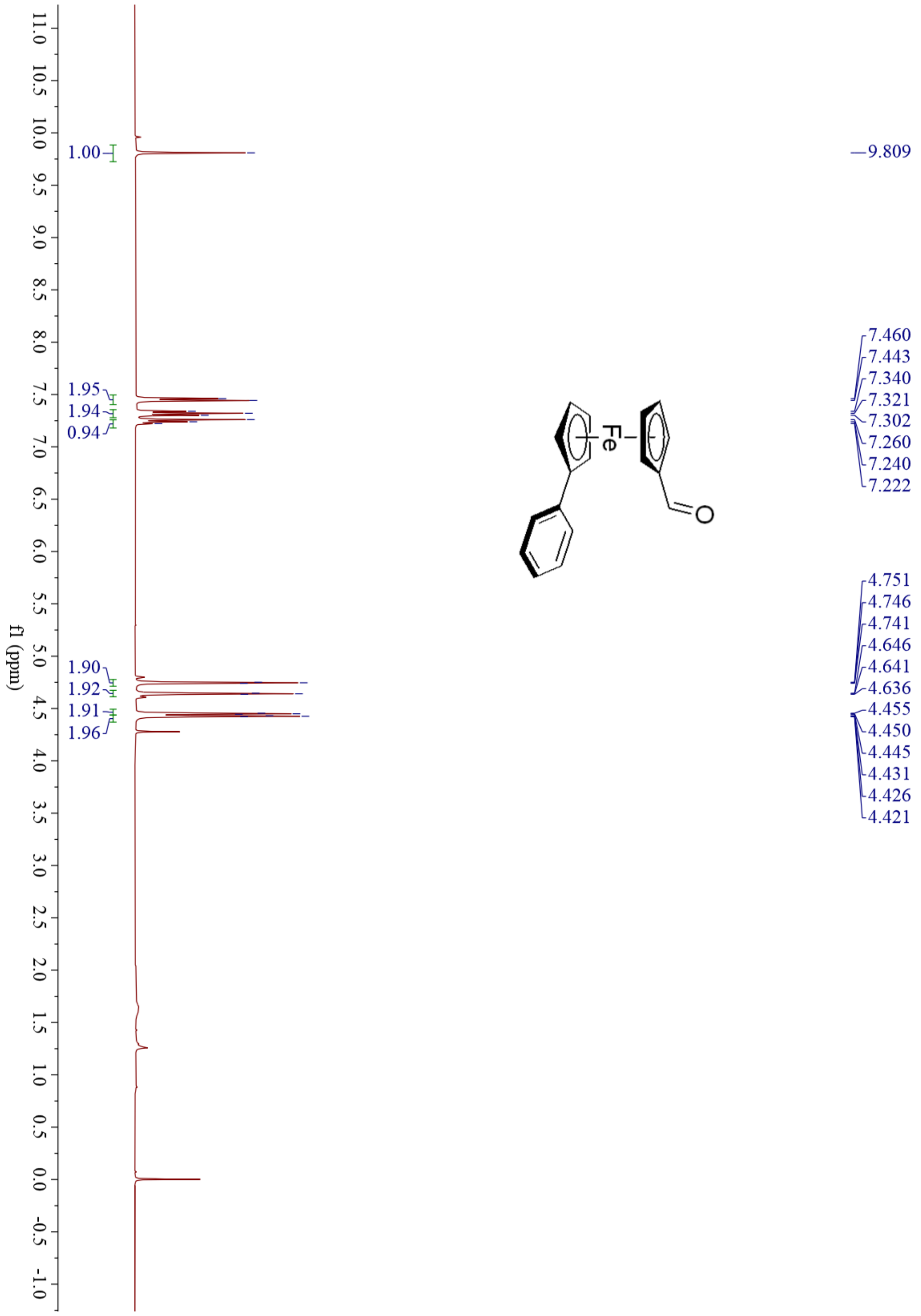

^{13}C NMR spectra of 1k

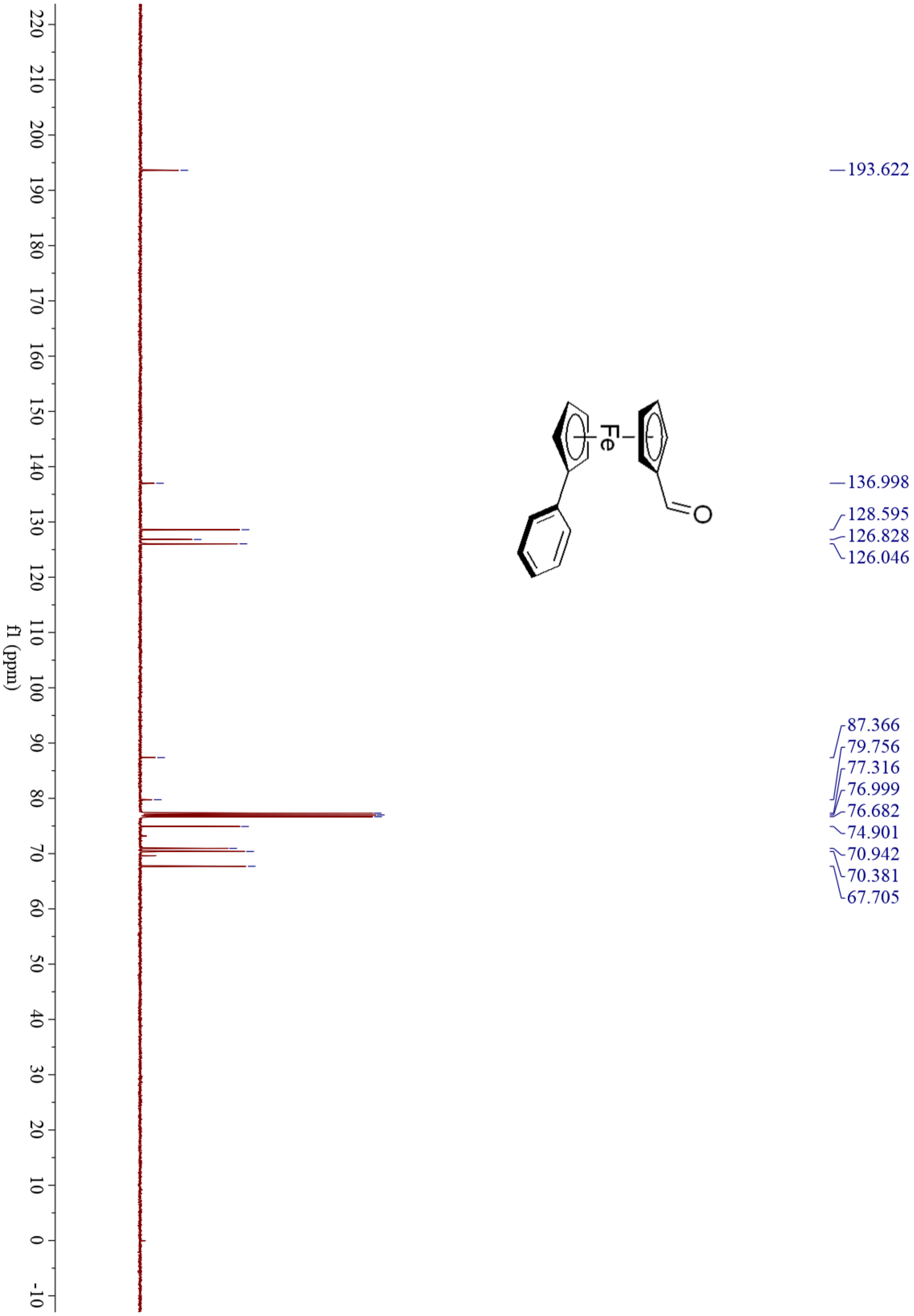

^{1}H NMR spectra of 1l

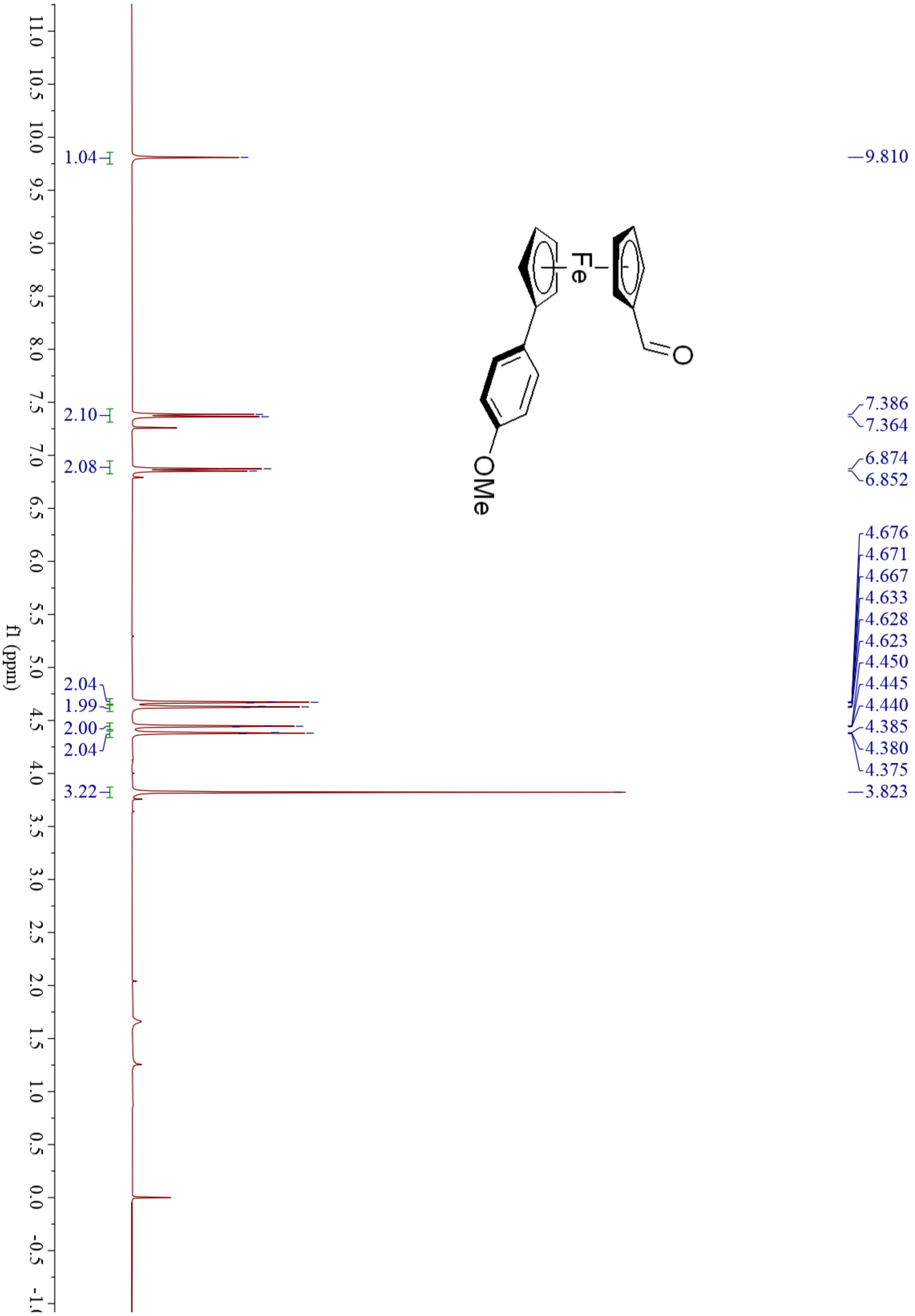

^{13}C NMR spectra of 1l

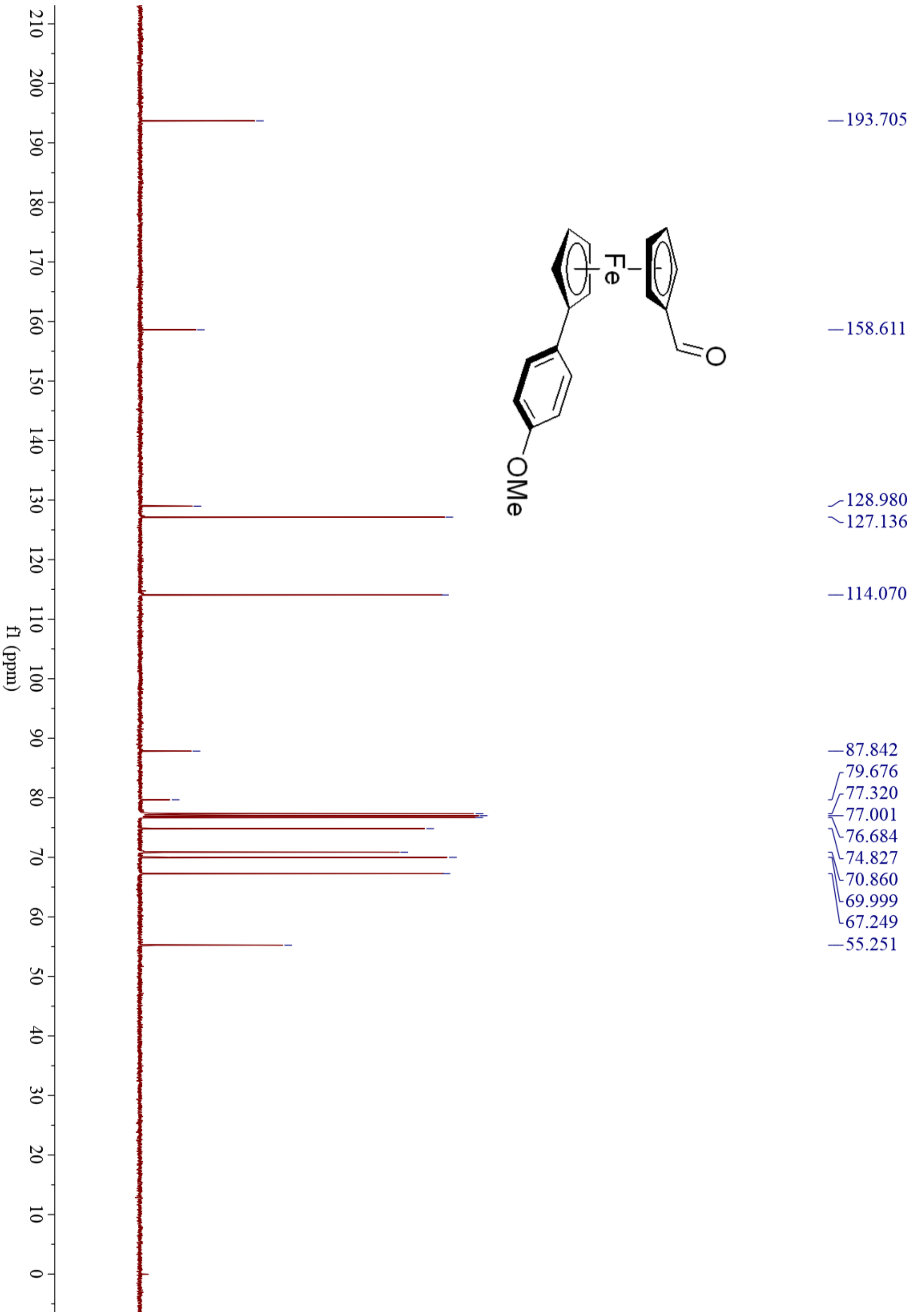

^{1}H NMR spectra of 1m

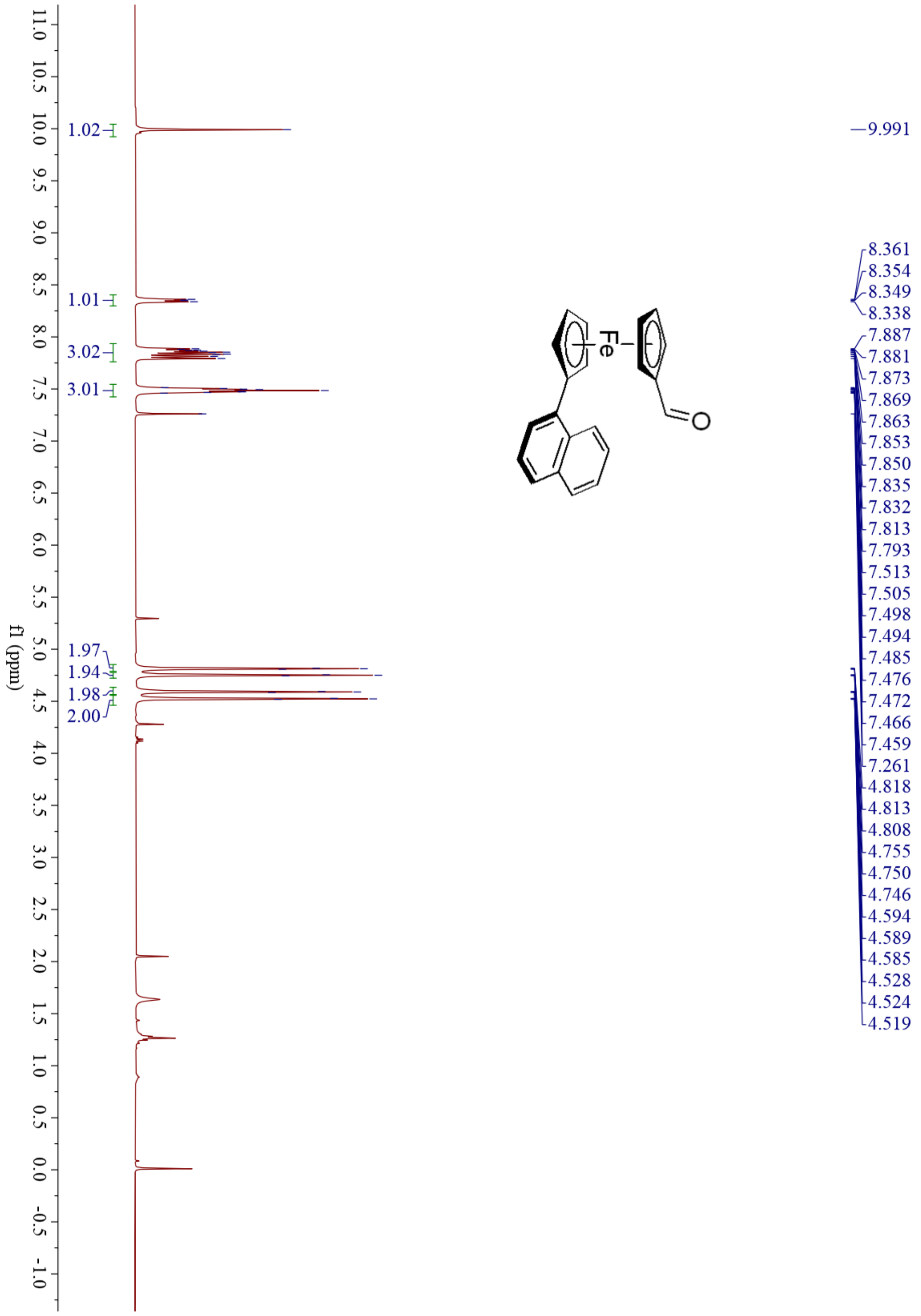

^{13}C NMR spectra of 1m

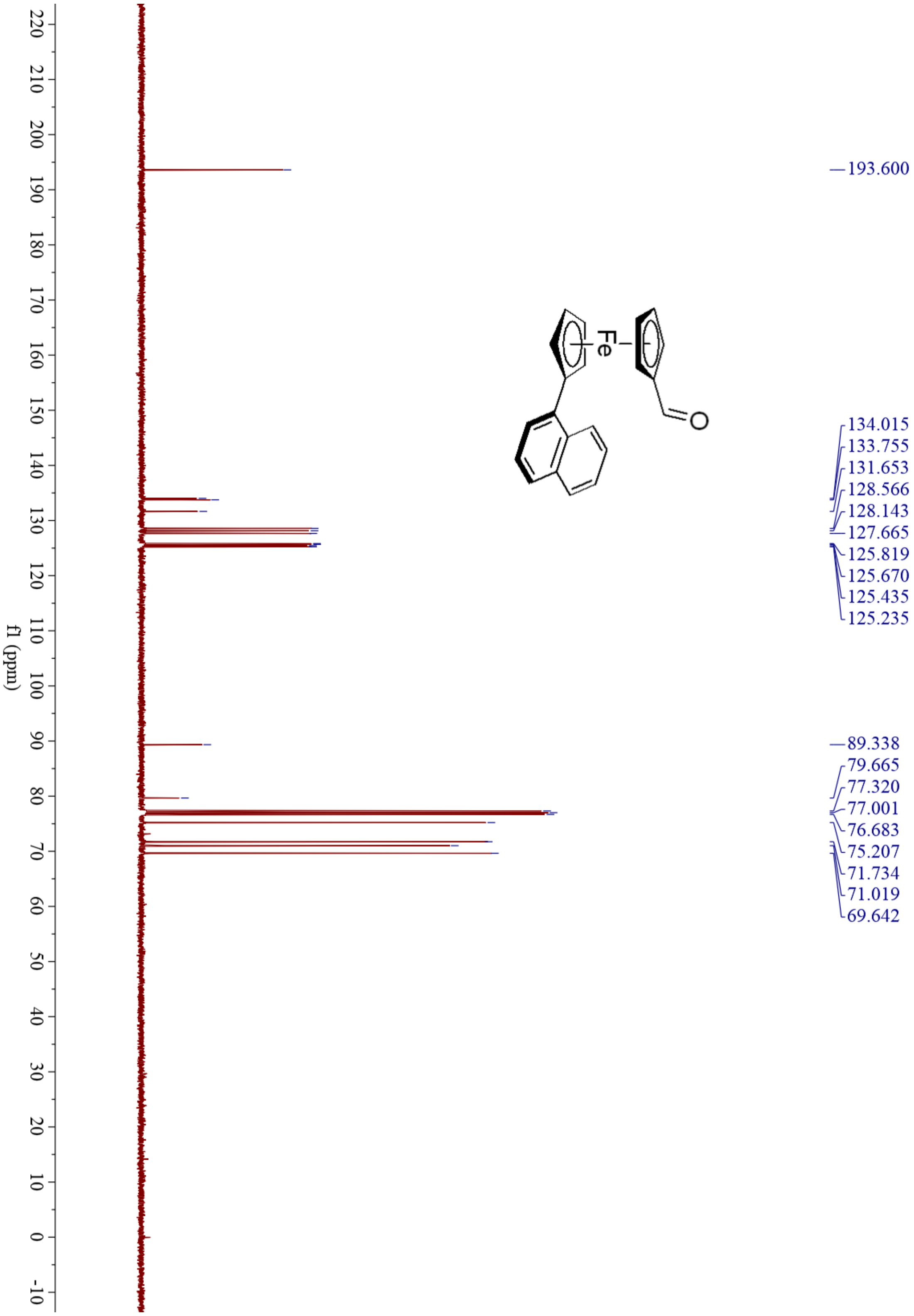

¹H NMR spectra of 1n

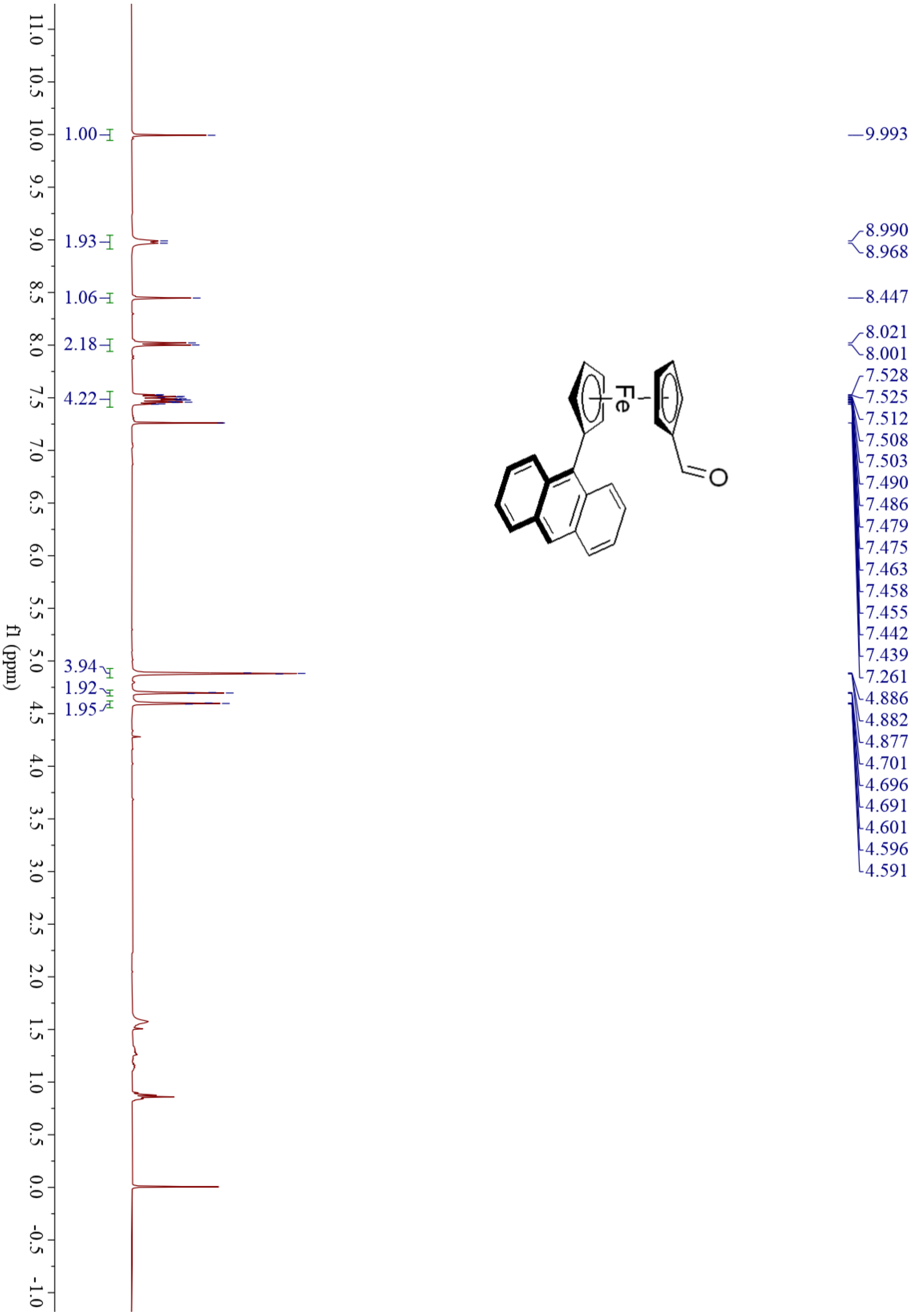

^{13}C NMR spectra of 1n

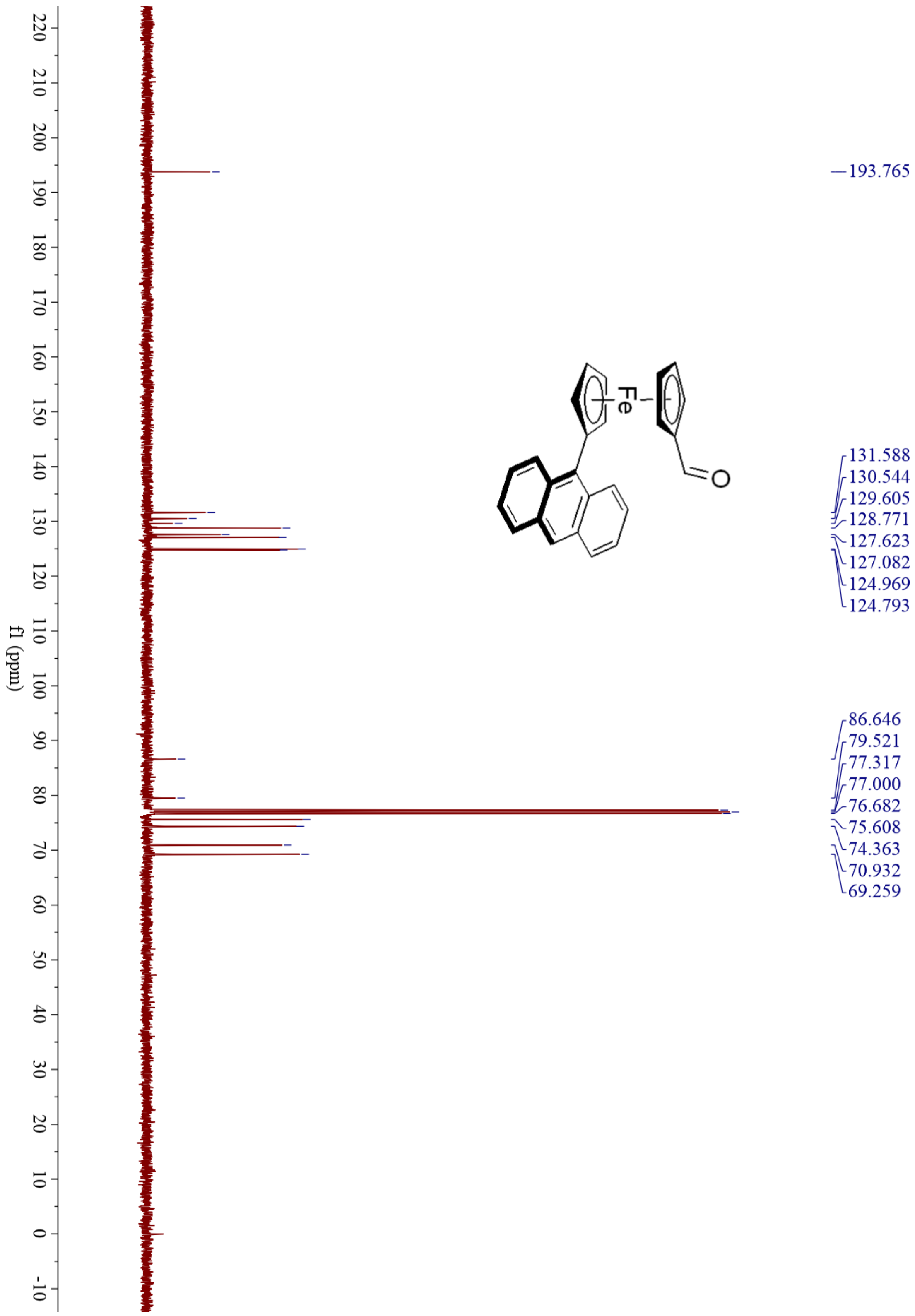

^{1}H NMR spectra of 1o

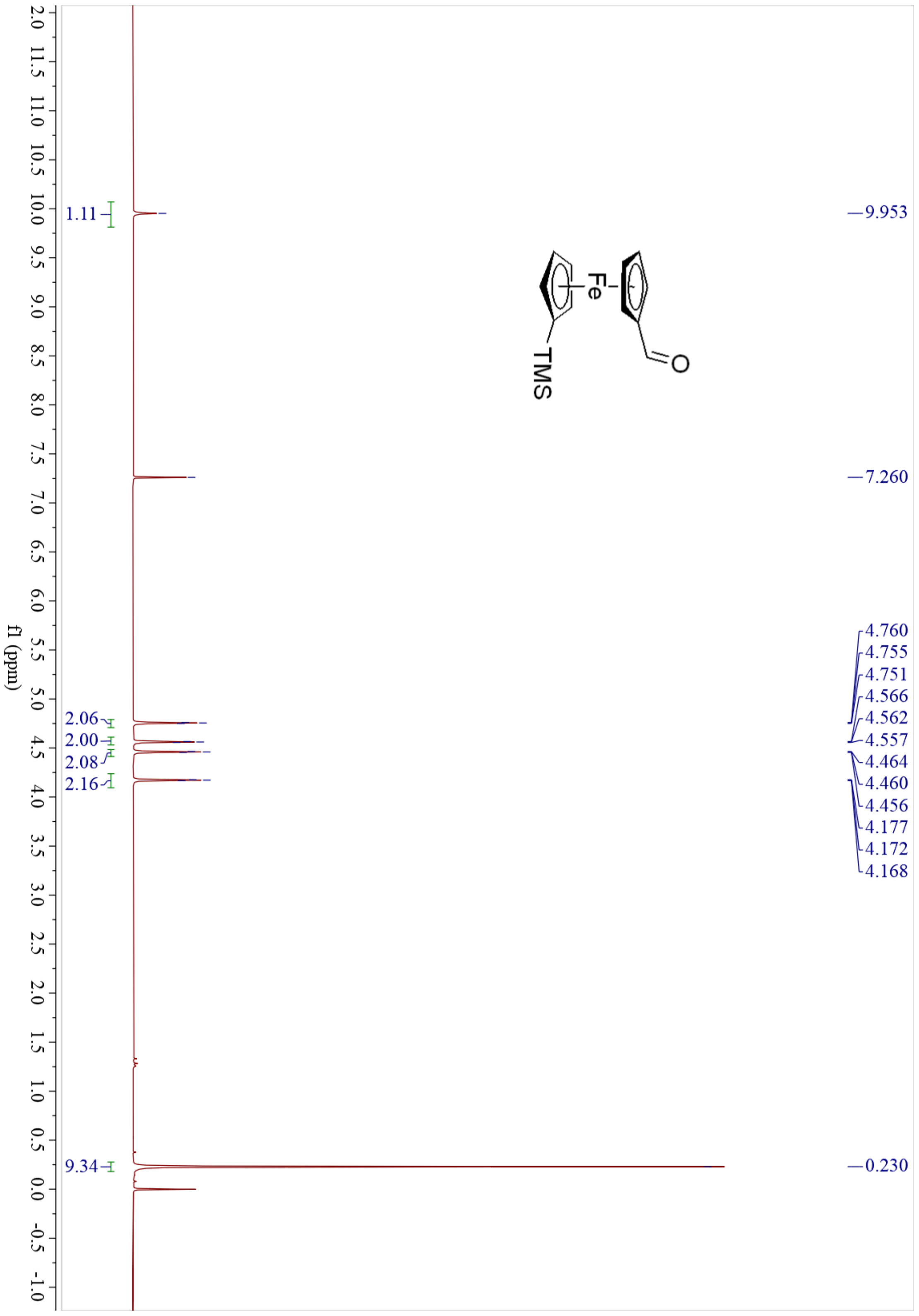

^{13}C NMR spectra of 1o

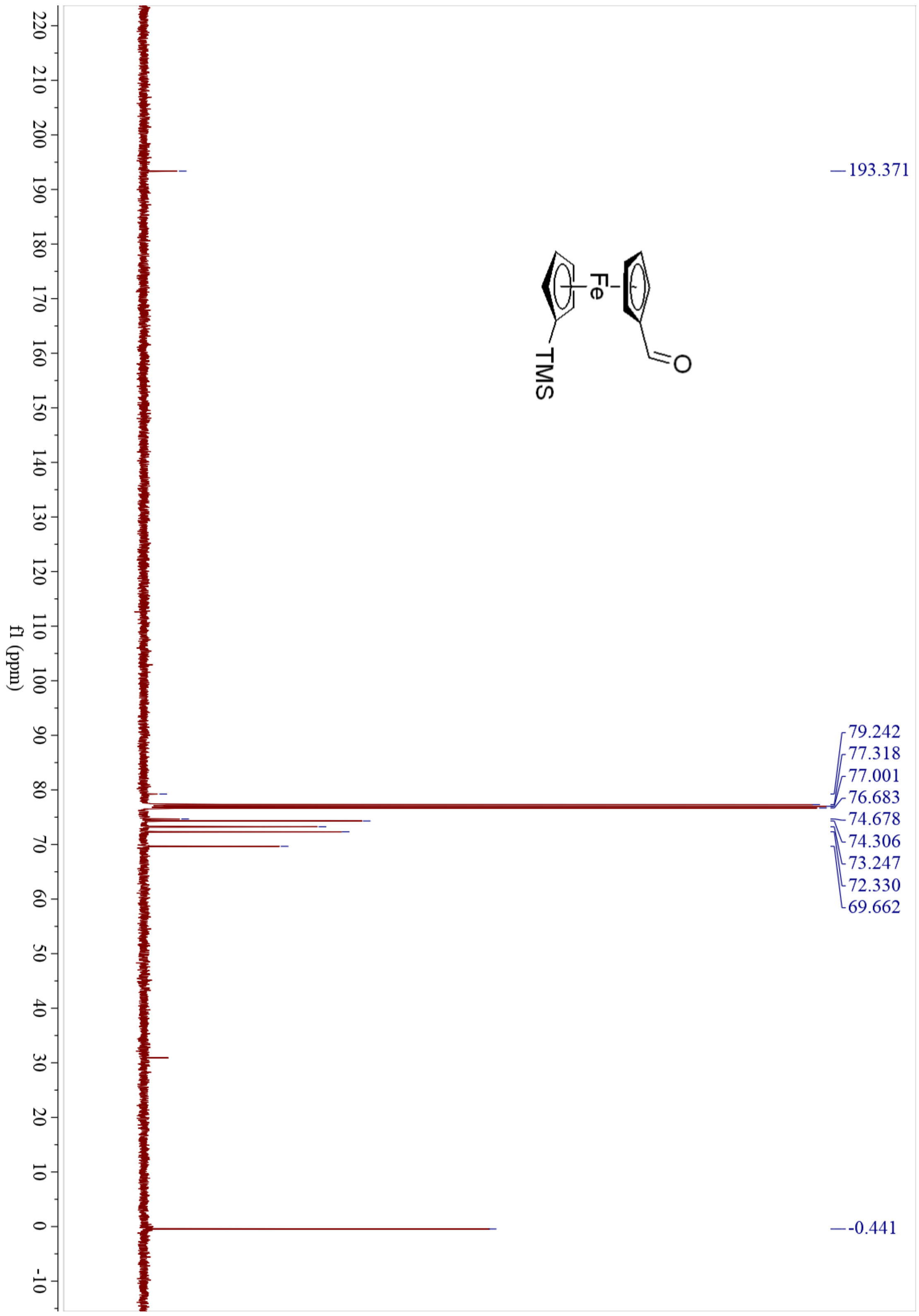

^{1}H NMR spectra of 3aa

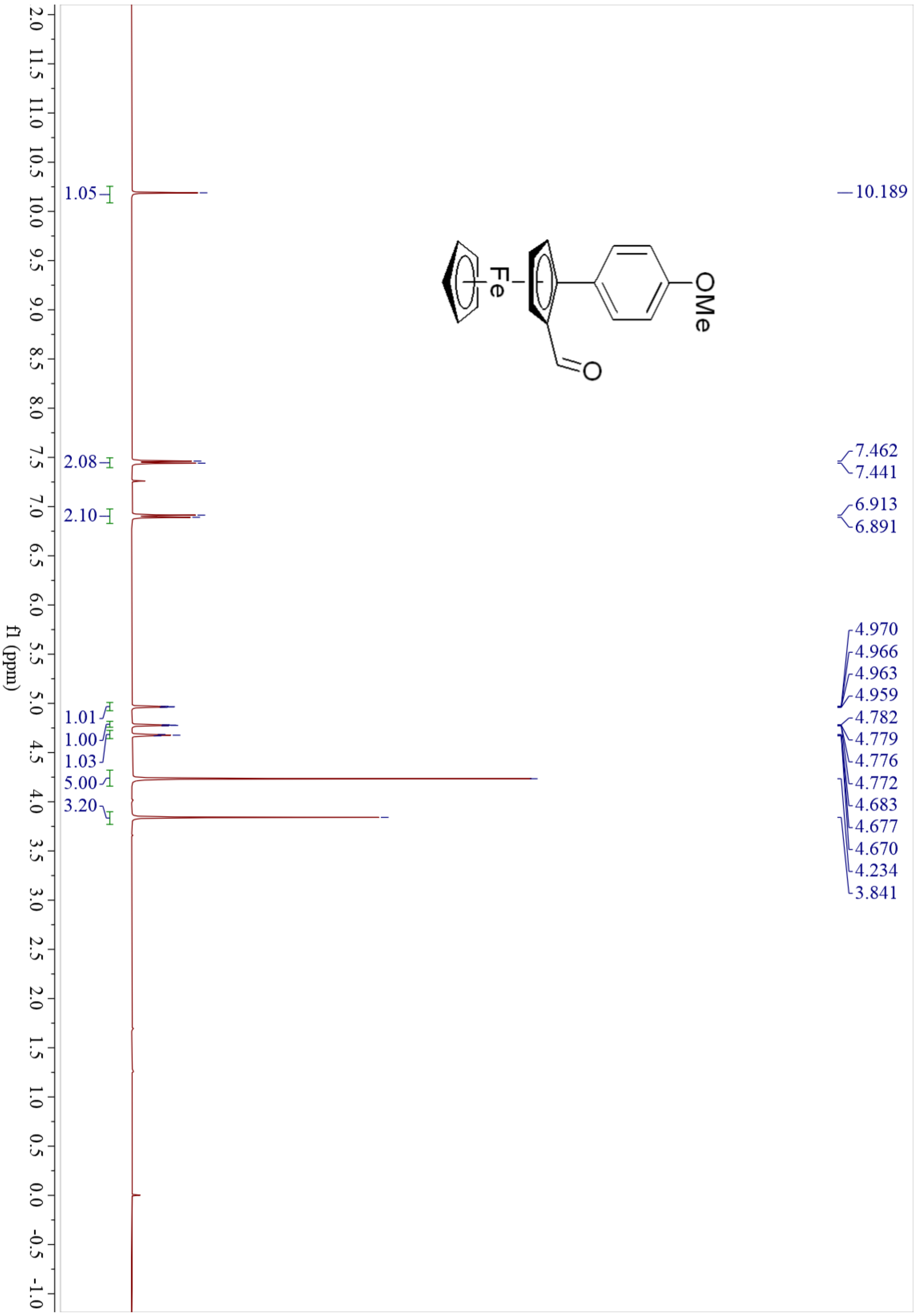

^{13}C NMR spectra of 3aa

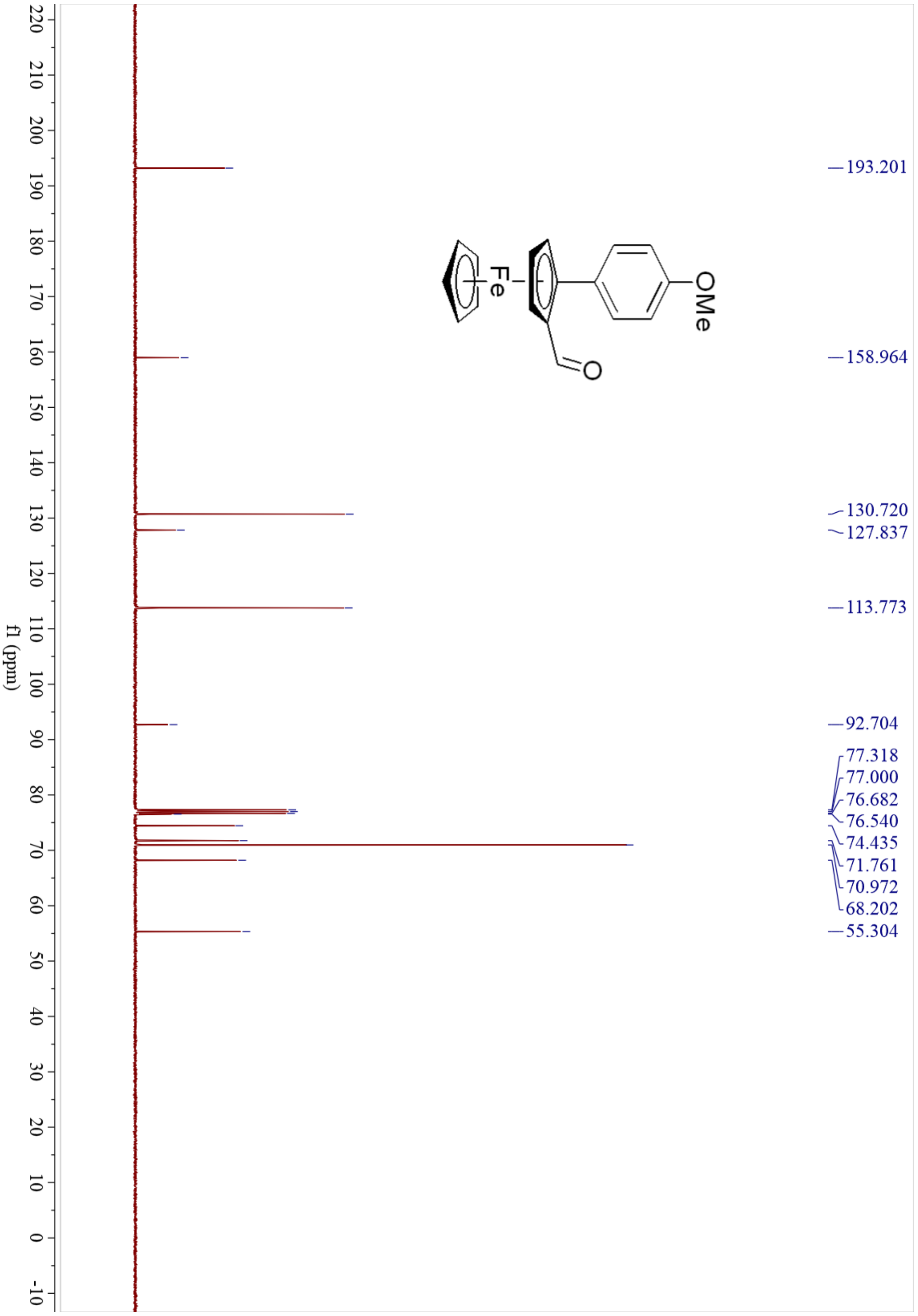

HPLC analysis of 3aa

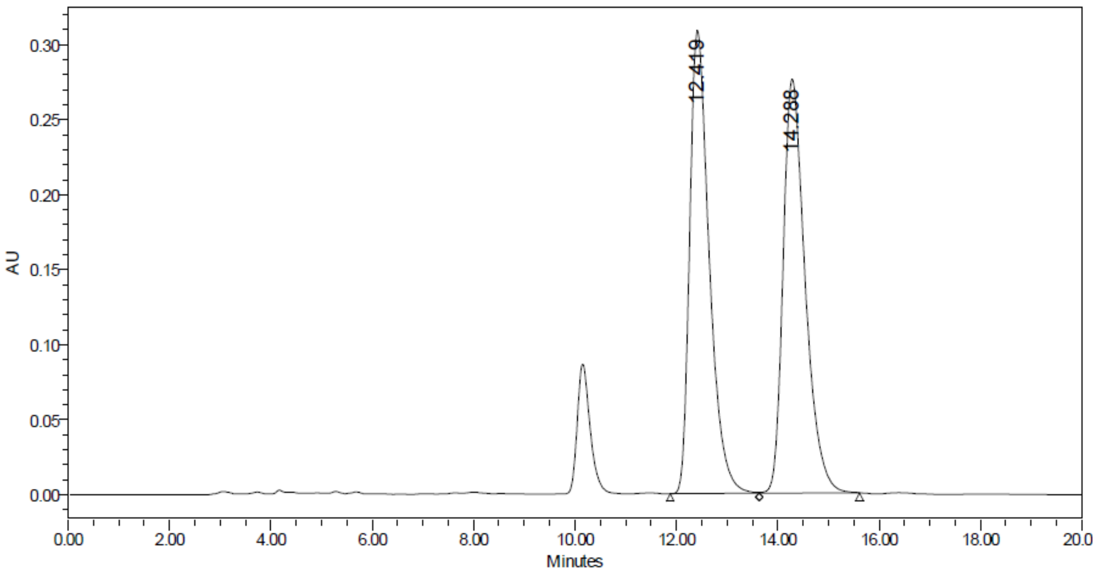

	RT	Area	% Area	Height
1	12.419	8284084	49.84	308903
2	14.288	8336534	50.16	276079

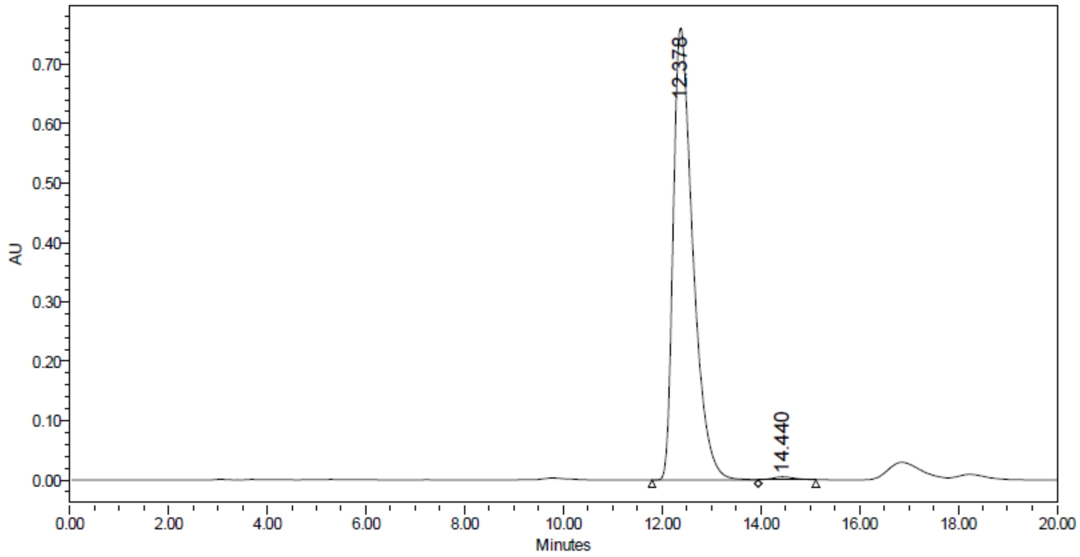

	RT	Area	% Area	Height
1	12.378	21031600	99.27	760855
2	14.440	154400	0.73	5024

^{1}H NMR spectra of 3ab

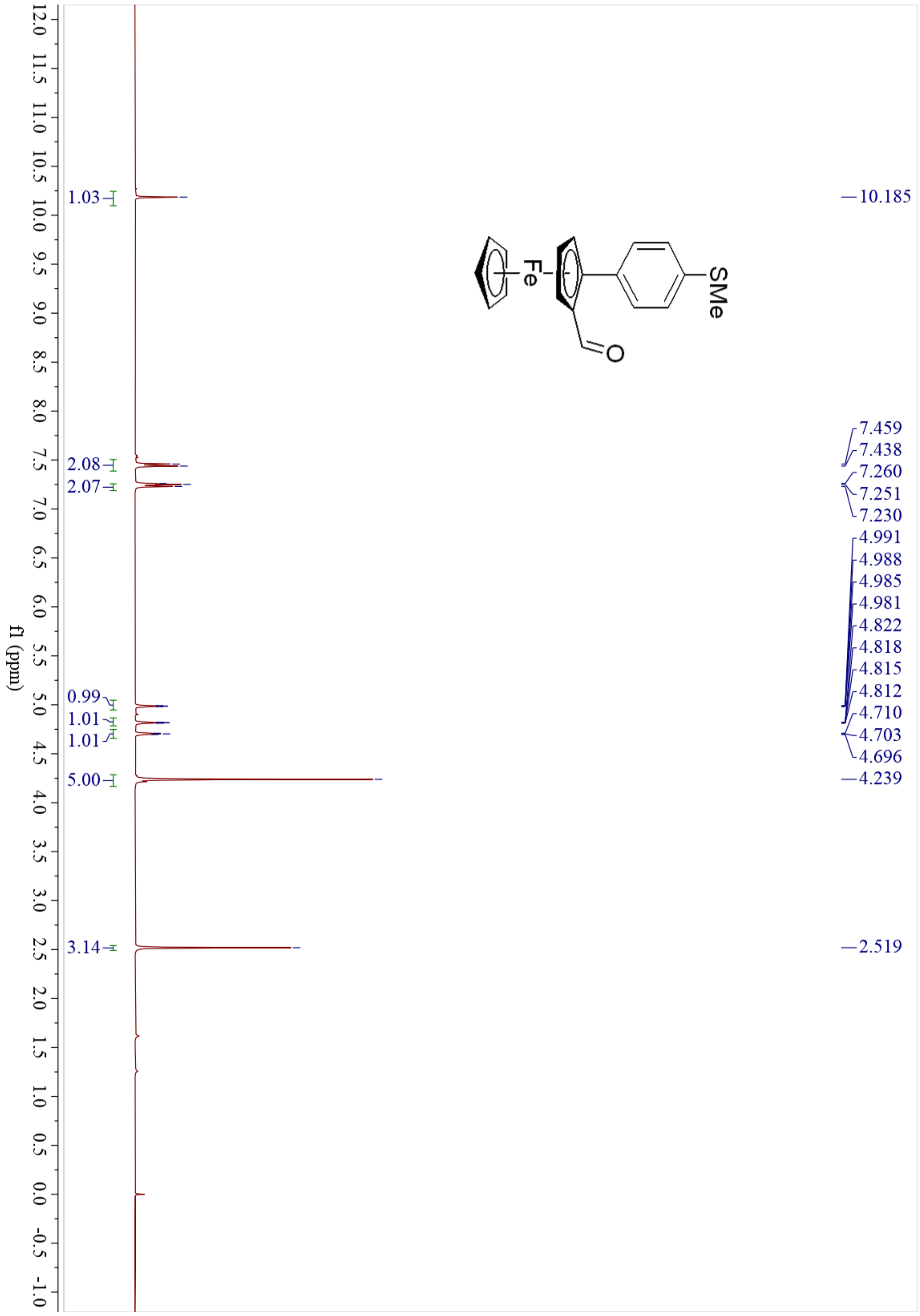

^{13}C NMR spectra of 3ab

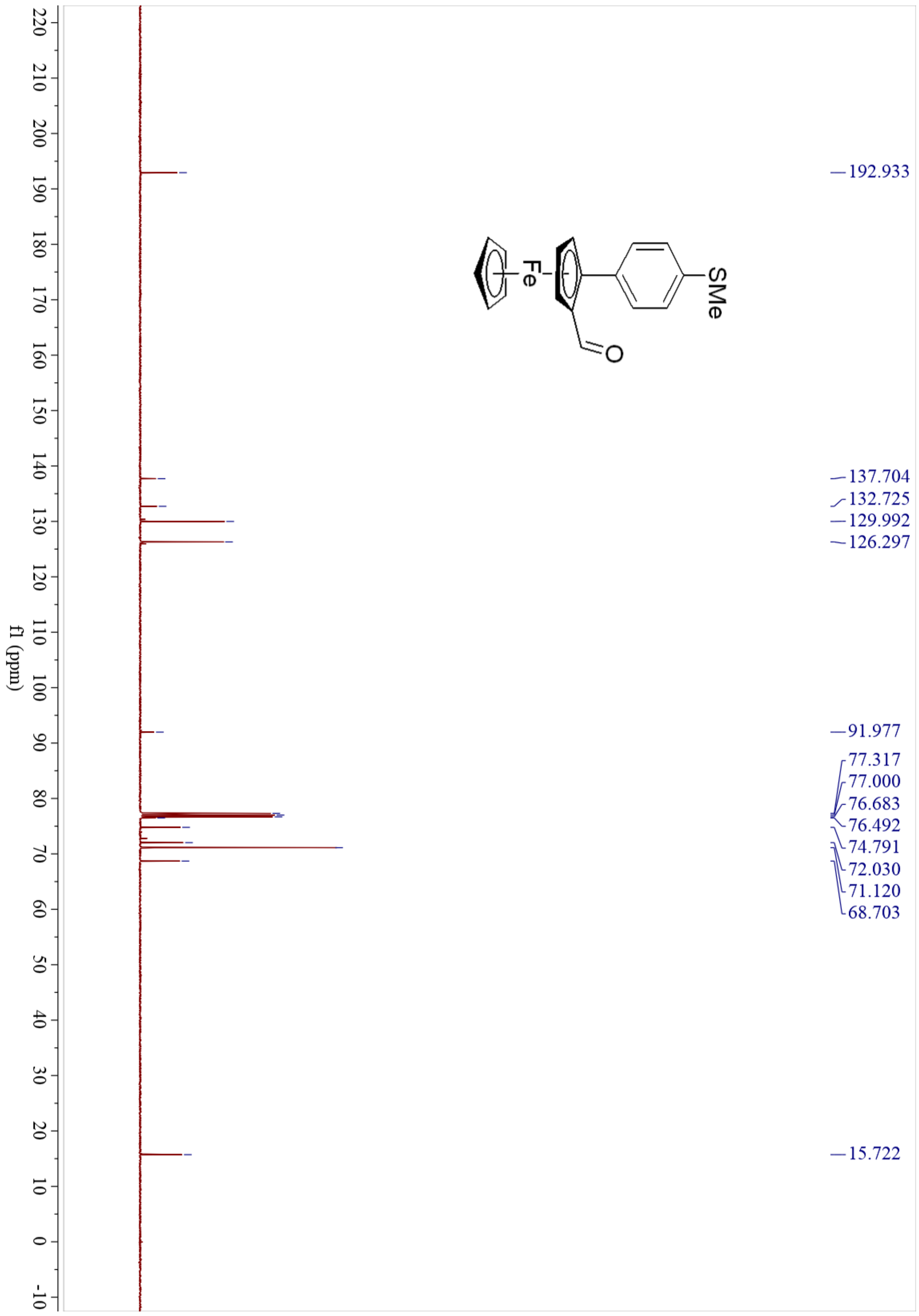

HPLC analysis of 3ab

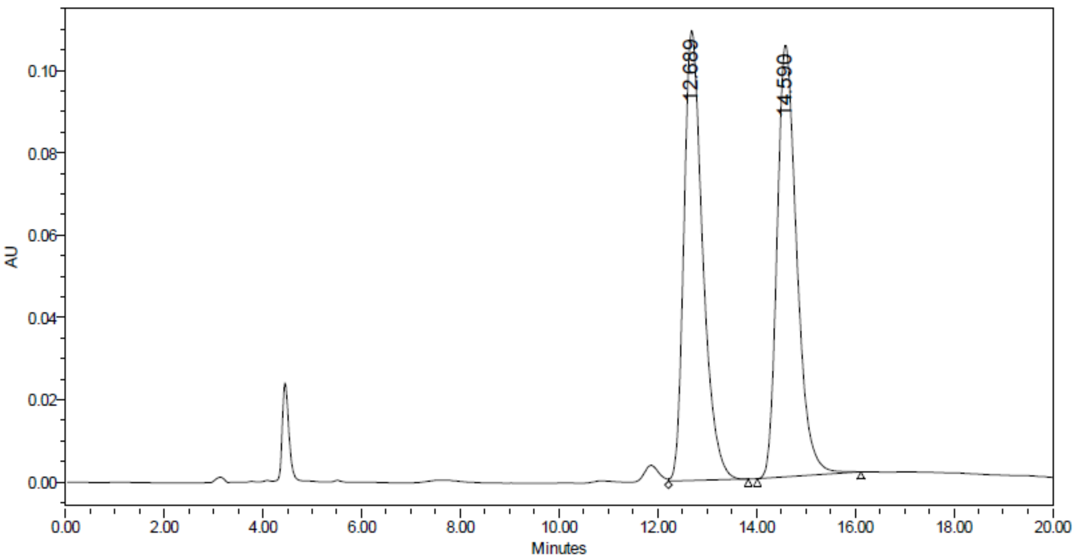

	RT	Area	% Area	Height
1	12.689	2891903	49.84	109321
2	14.590	2910556	50.16	104833

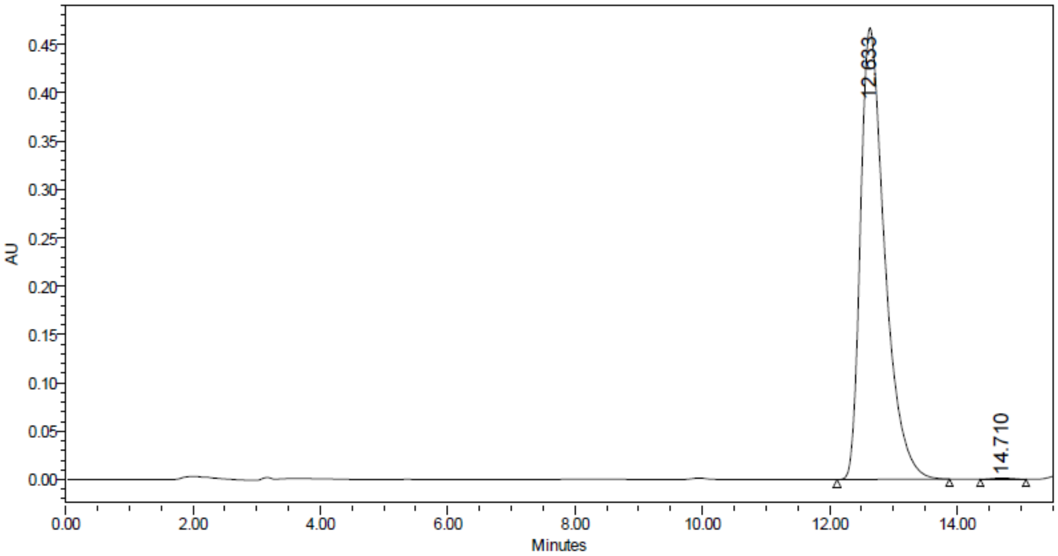

	RT	Area	% Area	Height
1	12.633	12341310	99.78	466841
2	14.710	26804	0.22	1169

¹H NMR spectra of 3ac

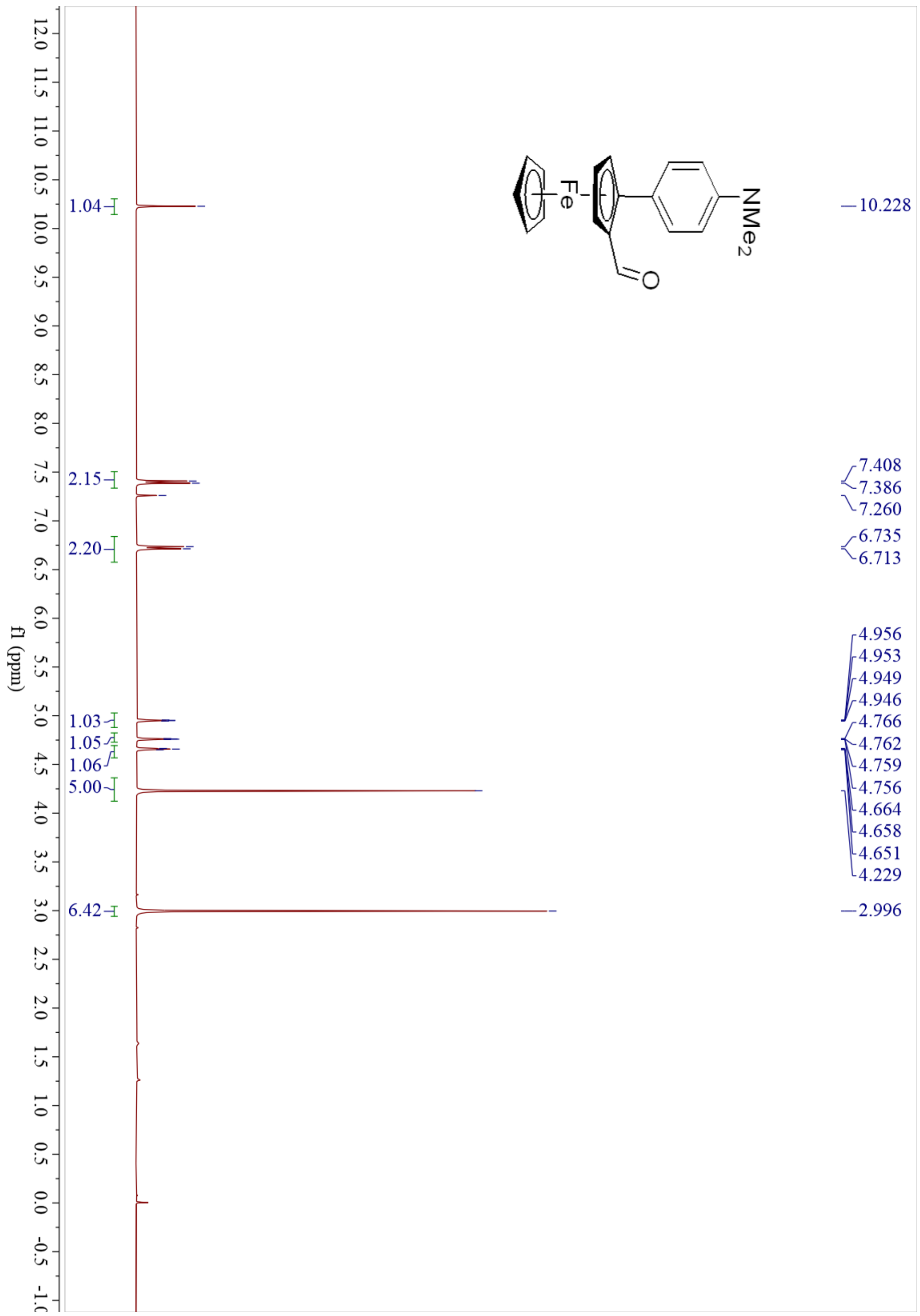

^{13}C NMR spectra of 3ac

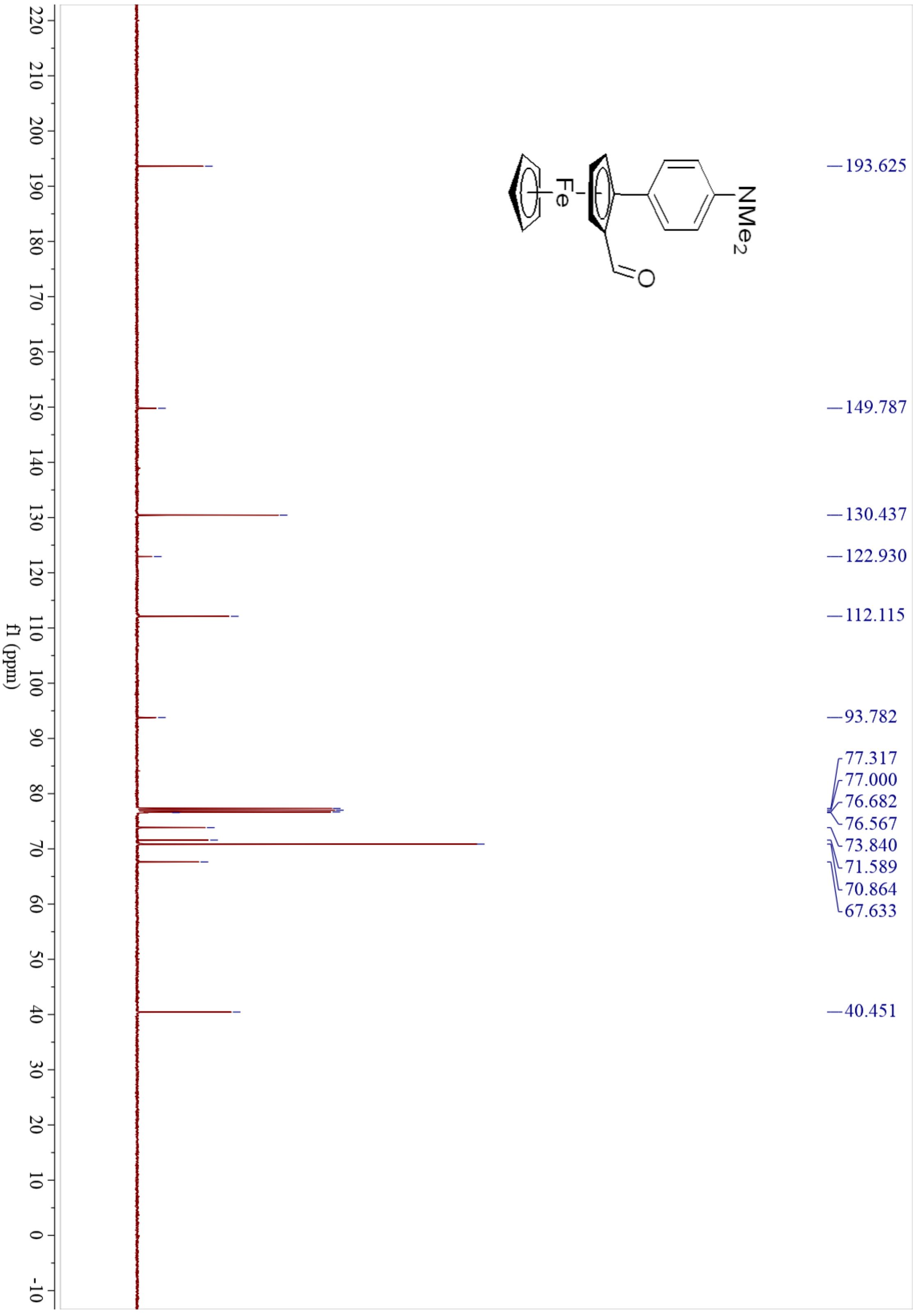

HPLC analysis of 3ac

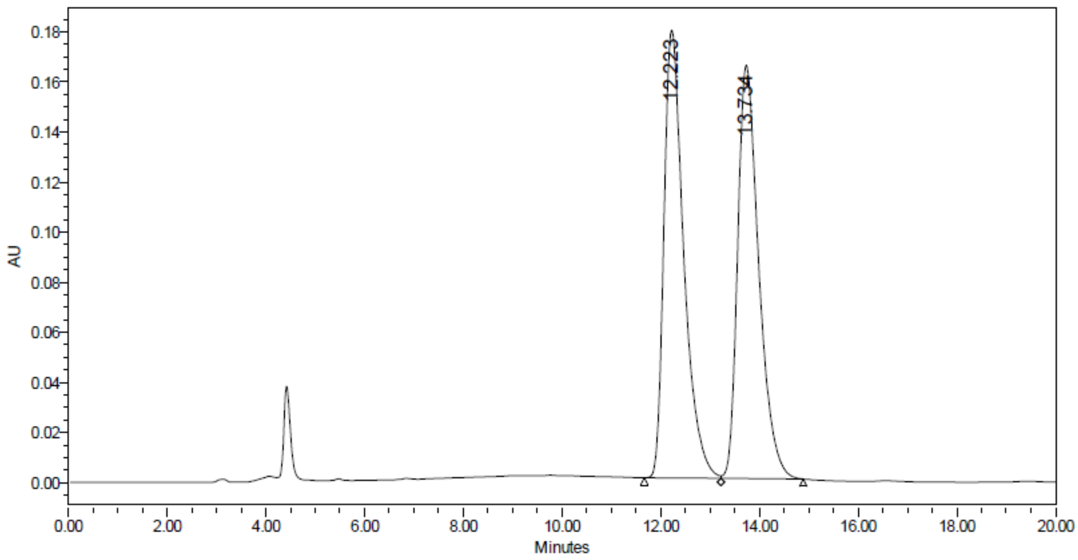

	RT	Area	% Area	Height
1	12.223	4980520	50.38	178993
2	13.734	4905617	49.62	165122

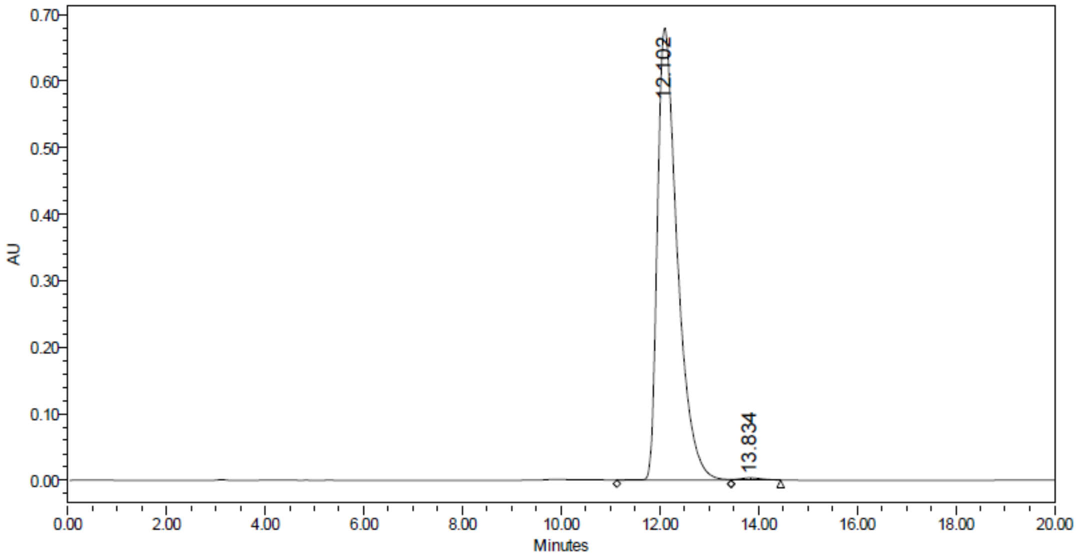

	RT	Area	% Area	Height
1	12.102	19001591	99.44	678903
2	13.834	107260	0.56	3515

^{1}H NMR spectra of 3ad

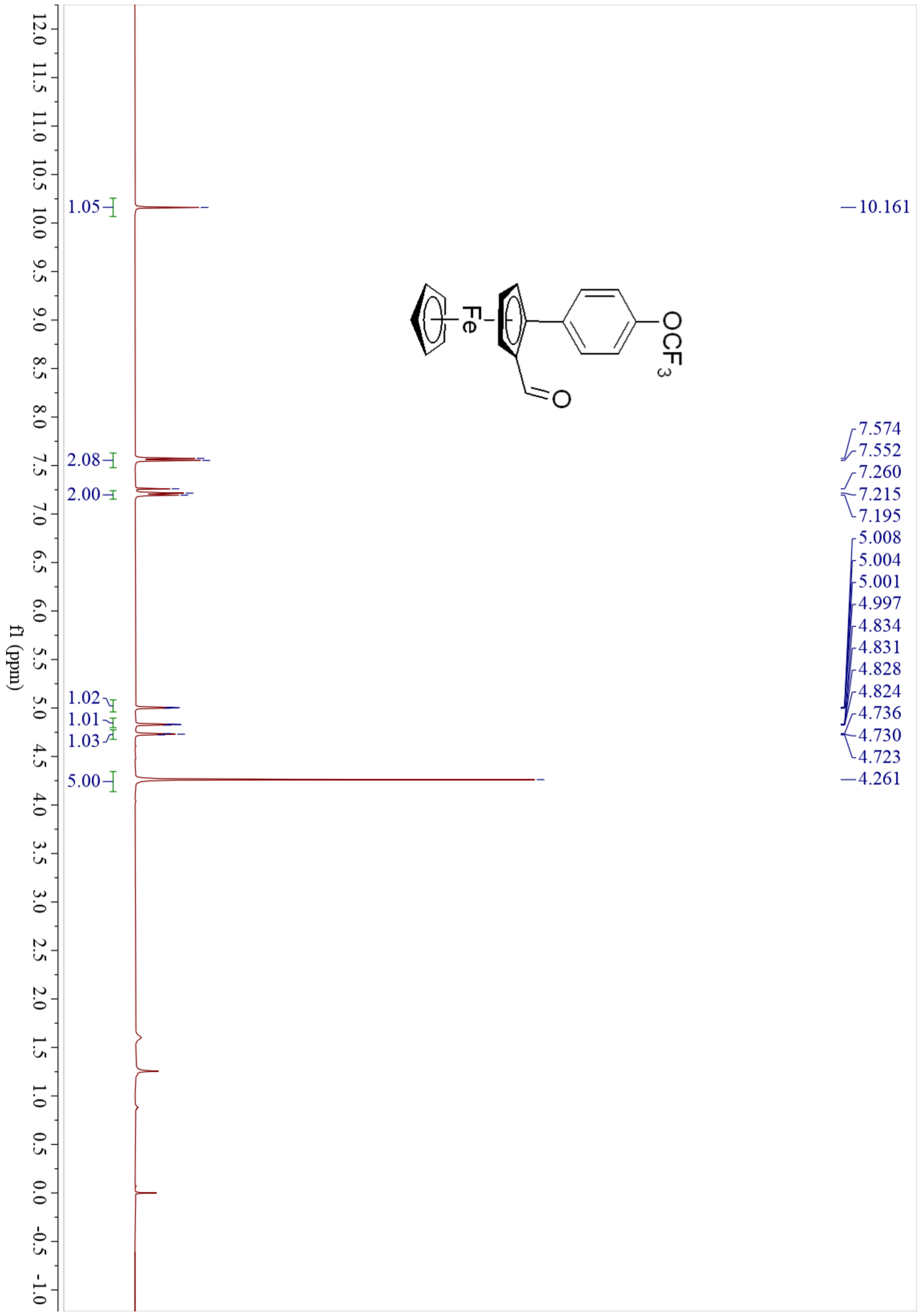

^{13}C NMR spectra of 3ad

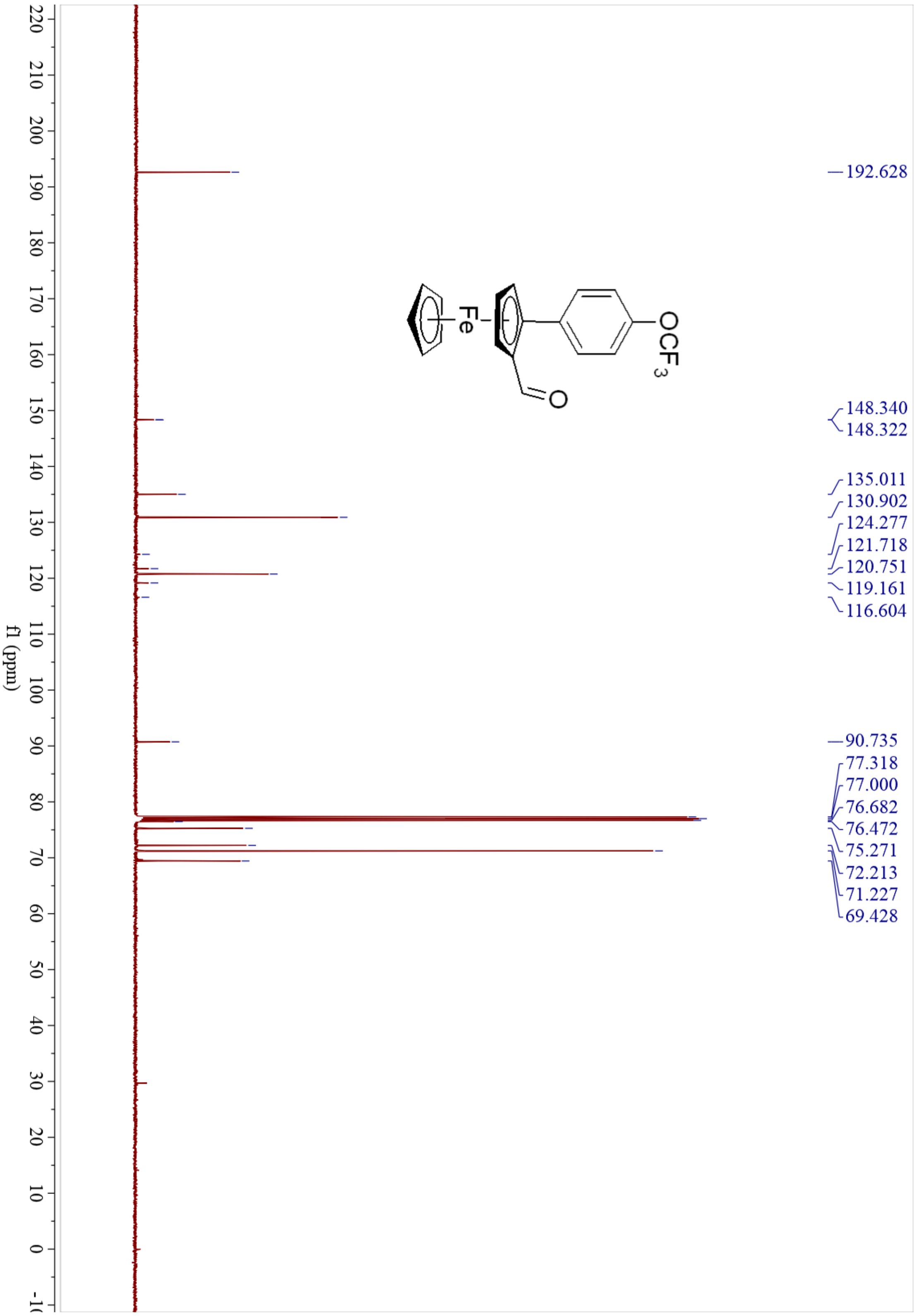

^{19}F NMR spectra of 3ad

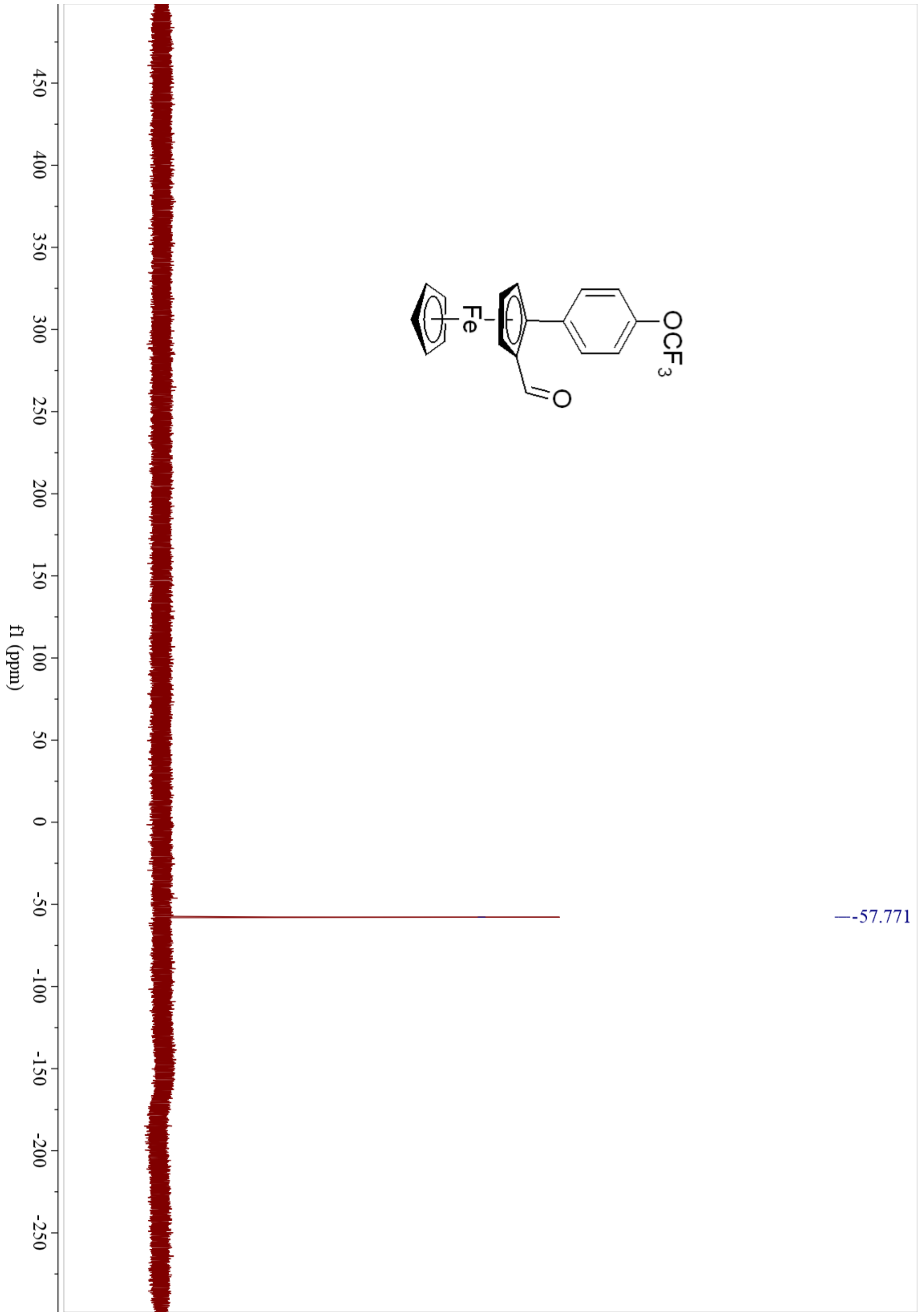

HPLC analysis of 3ad

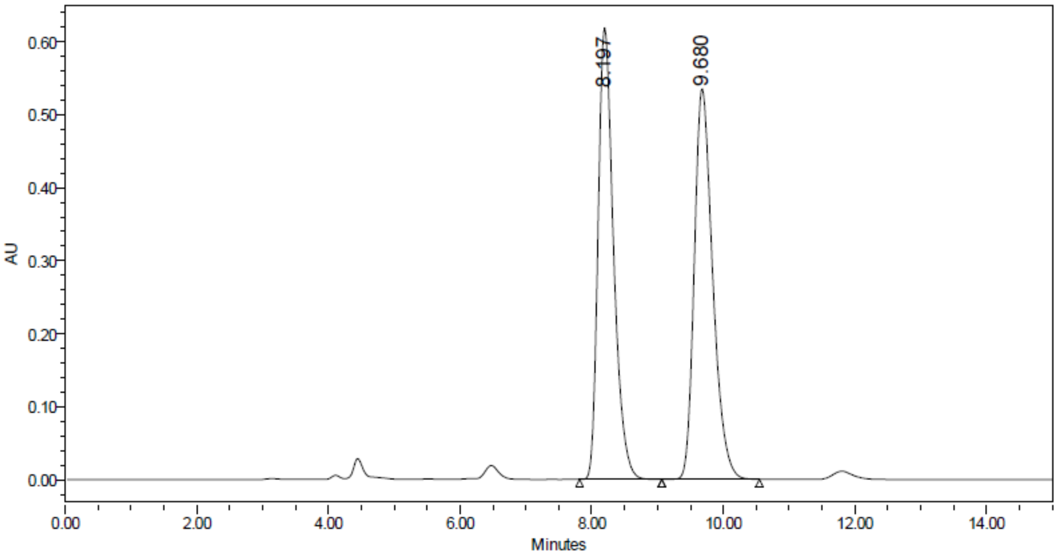

	RT	Area	% Area	Height
1	8.197	10103334	49.12	618833
2	9.680	10466389	50.88	535529

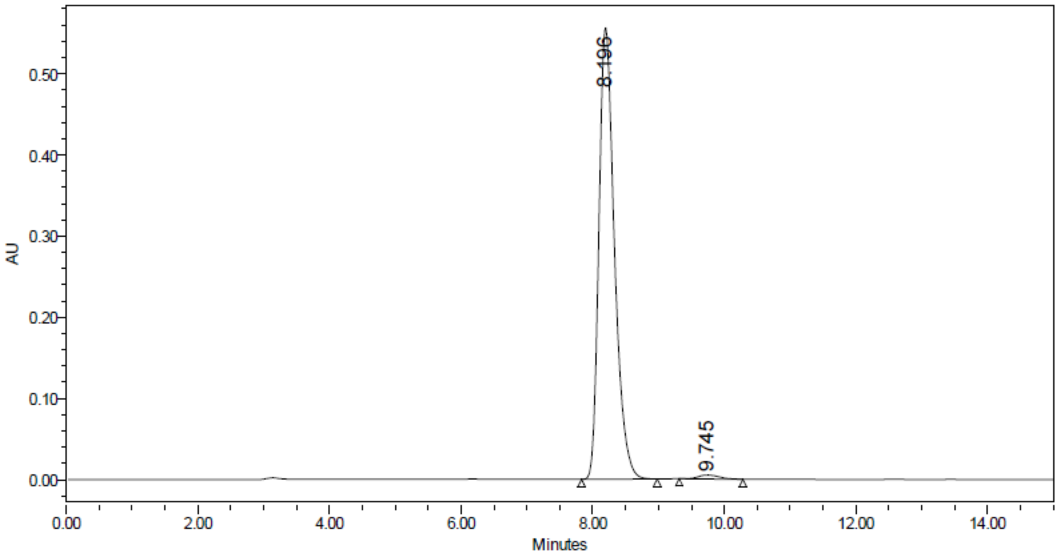

	RT	Area	% Area	Height
1	8.196	9117525	98.88	556153
2	9.745	103197	1.12	4617

^{1}H NMR spectra of 3ae

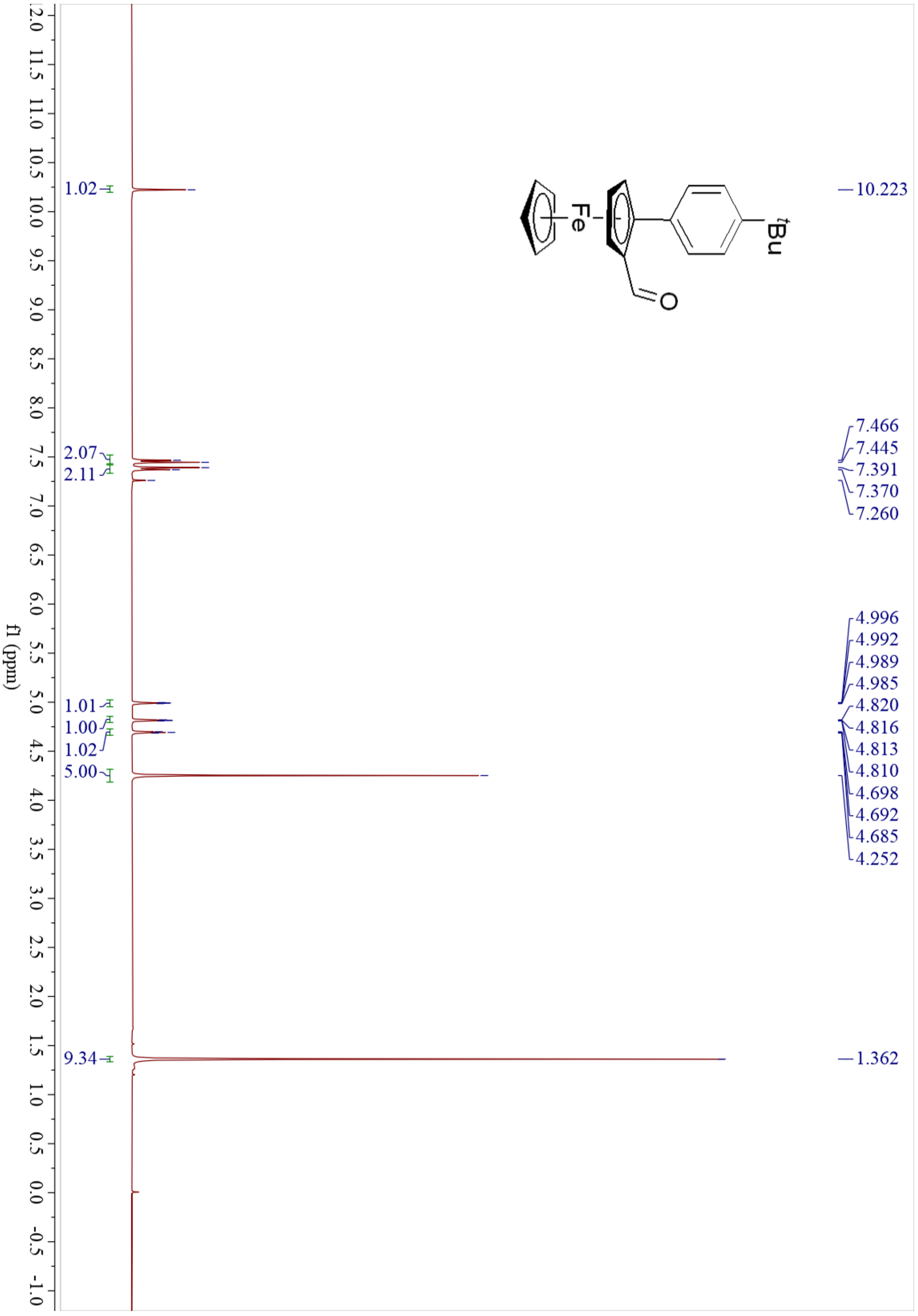

^{13}C NMR spectra of 3ae

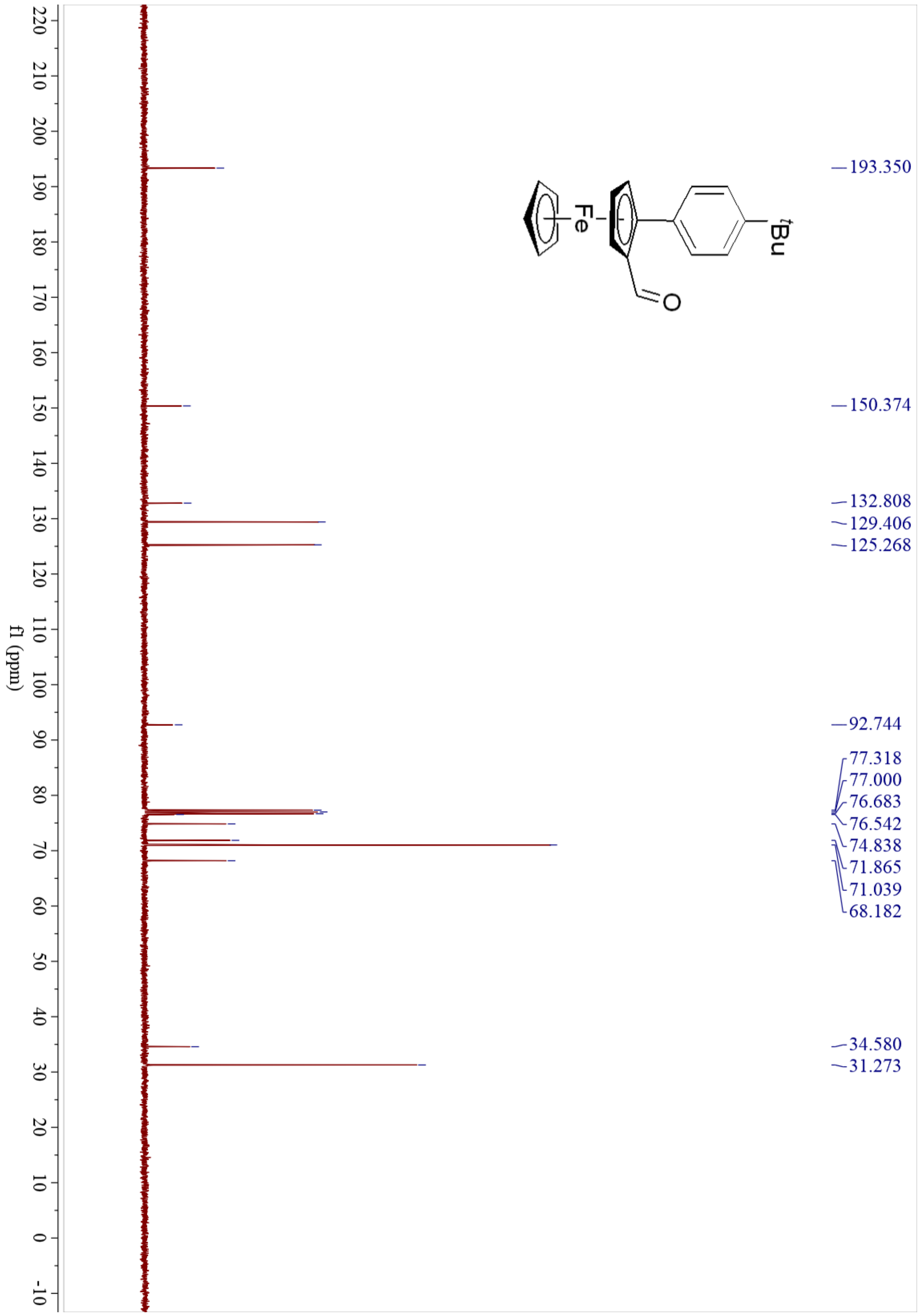

HPLC analysis of 3ae

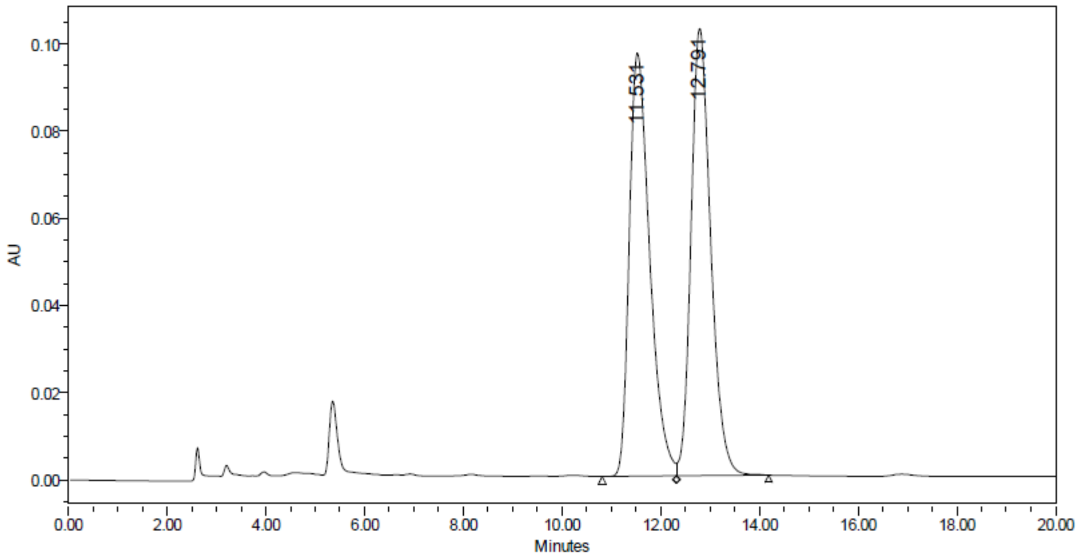

	RT	Area	% Area	Height
1	11.531	2791051	49.54	96893
2	12.791	2843338	50.46	102507

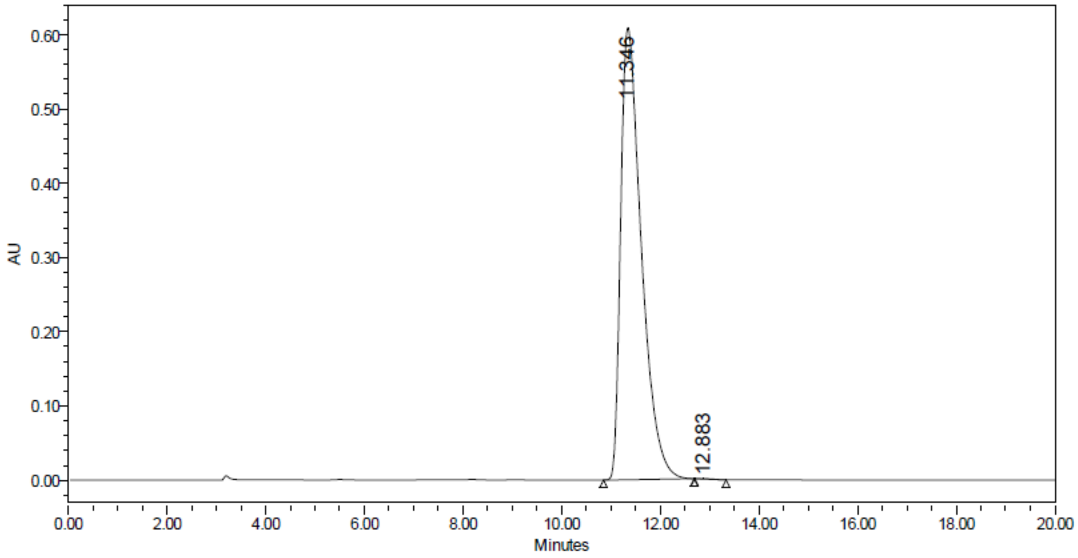

	RT	Area	% Area	Height
1	11.346	17715930	99.97	608697
2	12.883	5870	0.03	362

^{1}H NMR spectra of 3af

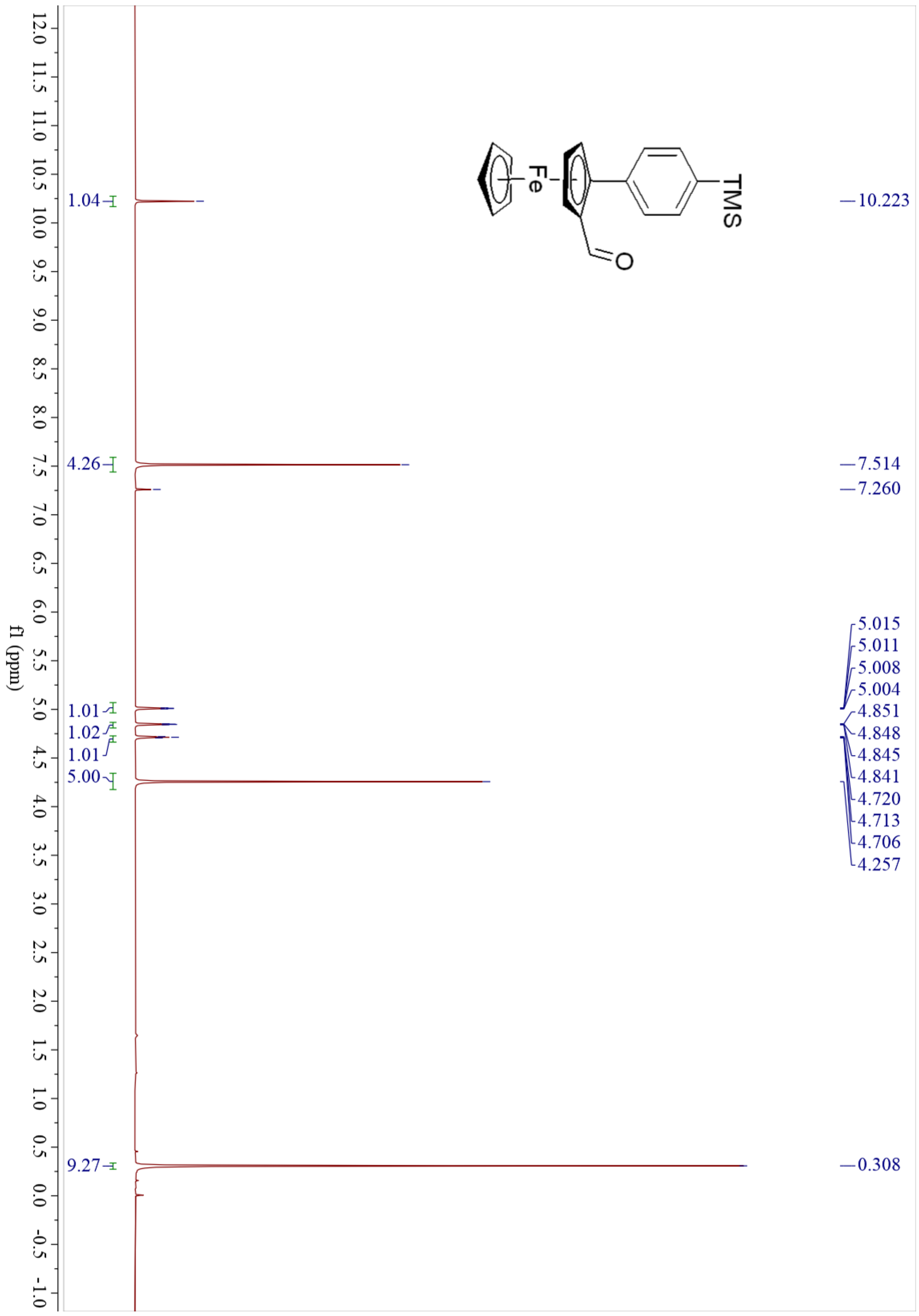

^{13}C NMR spectra of 3af

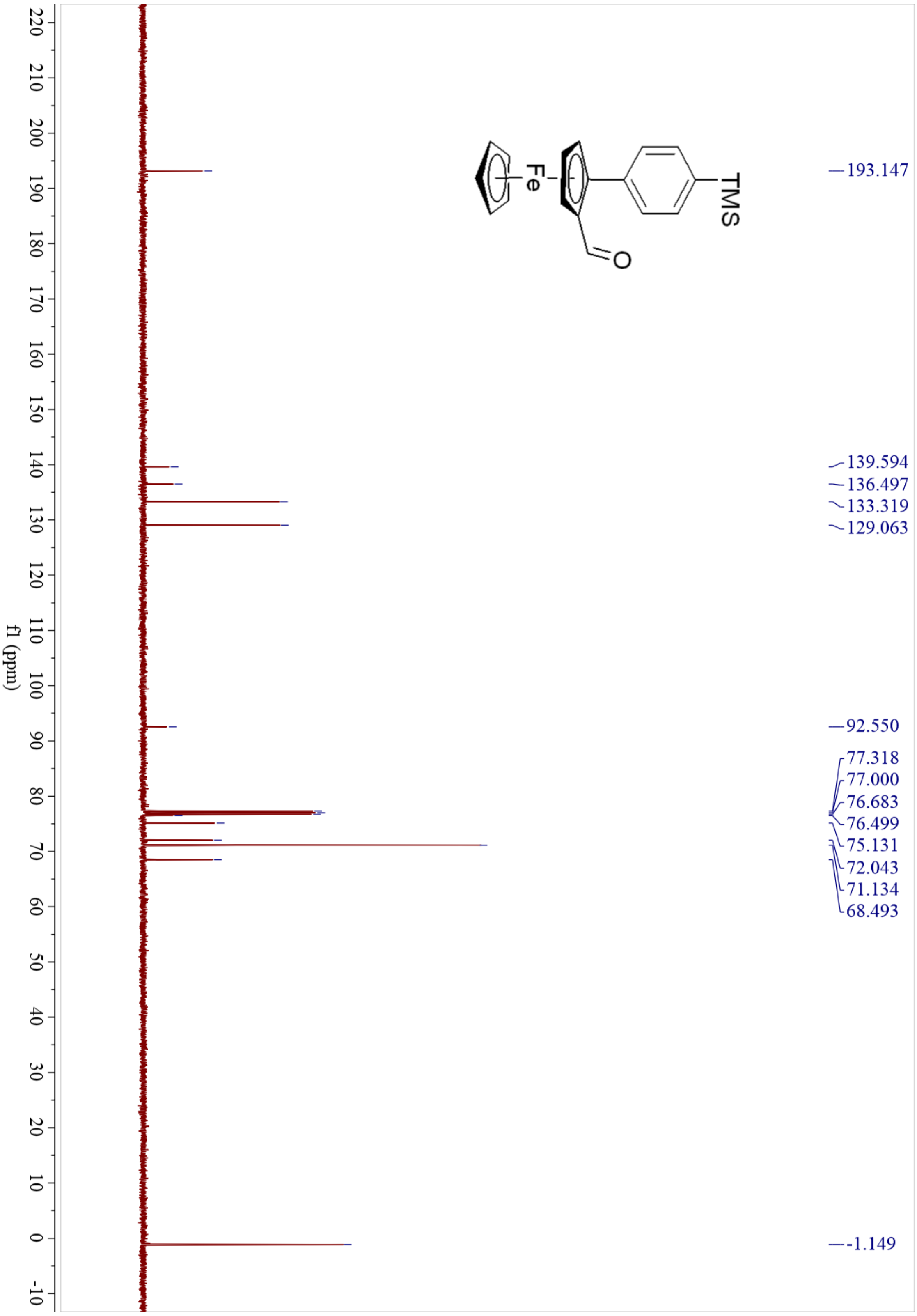

HPLC analysis of 3af

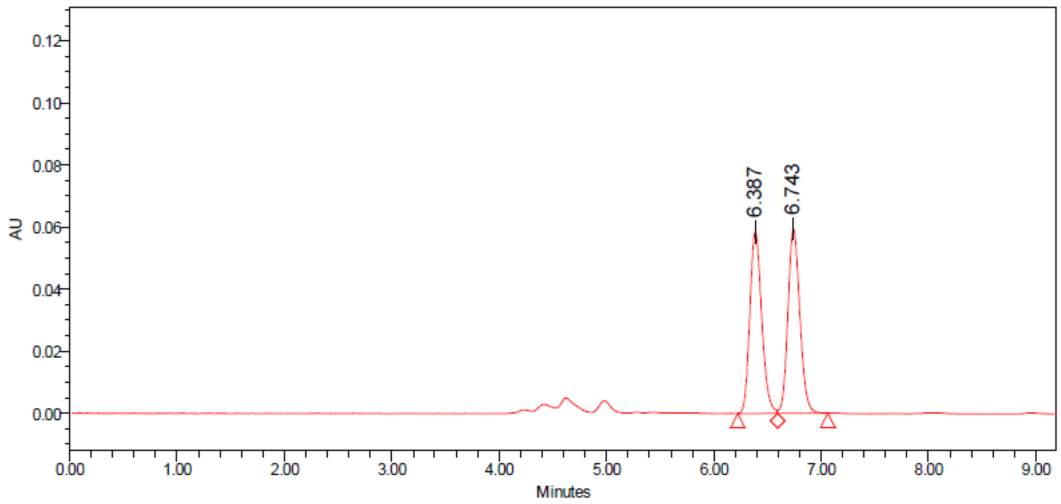

Peak Results

	SampleName	RT	Width (sec)	Height	Area	% Area
1	LCX-22-TMS-RAC-2022-3-8	6.387	22.300	58475	449350	49.57
2	LCX-22-TMS-RAC-2022-3-8	6.743	28.000	59374	457214	50.43

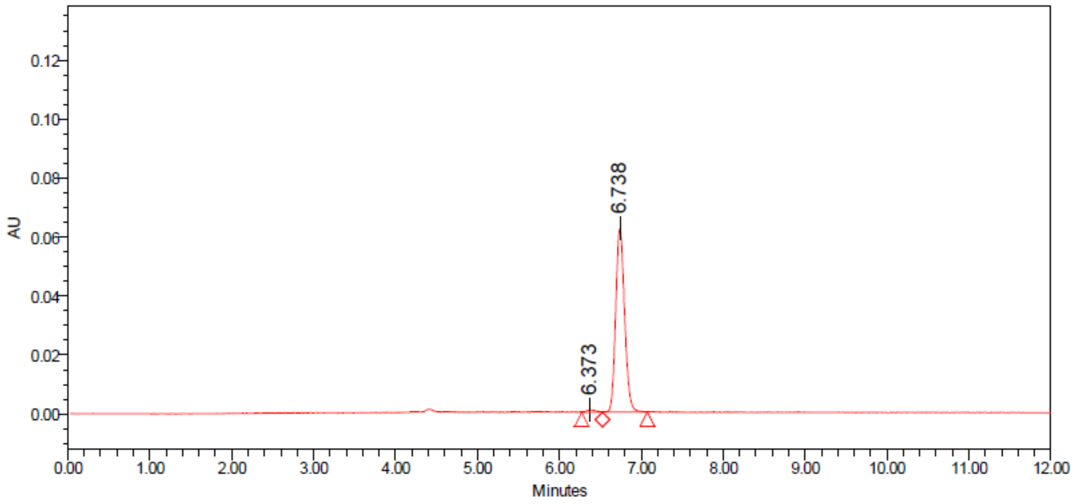

Peak Results

	SampleName	RT	Width (sec)	Height	Area	% Area
1	LCX-22-TMS-CHIRAL-2022-3-8	6.373	15.300	754	5968	1.25
2	LCX-22-TMS-CHIRAL-2022-3-8	6.738	32.700	62307	470437	98.75

^{1}H NMR spectra of 3ag

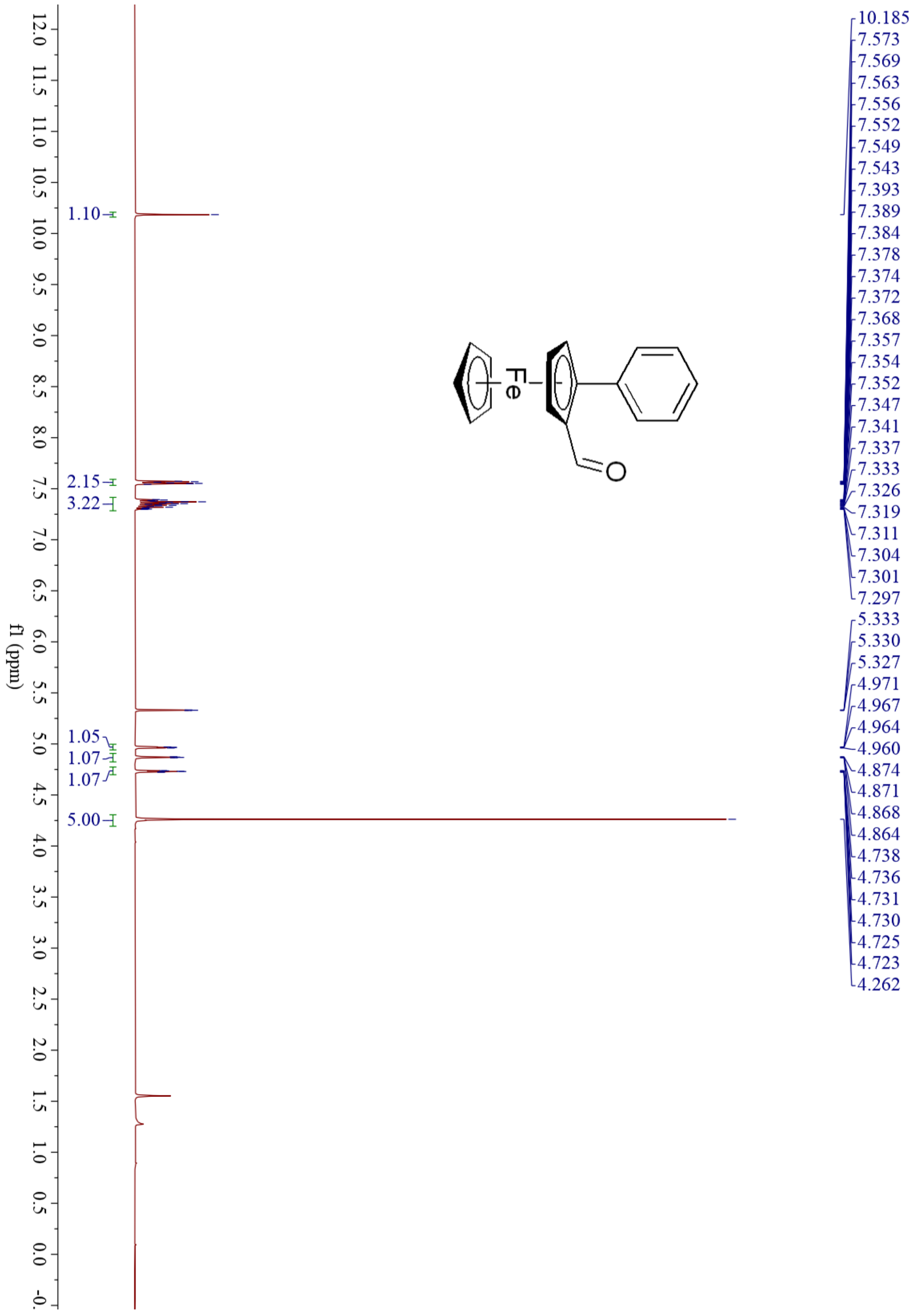

^{13}C NMR spectra of 3ag

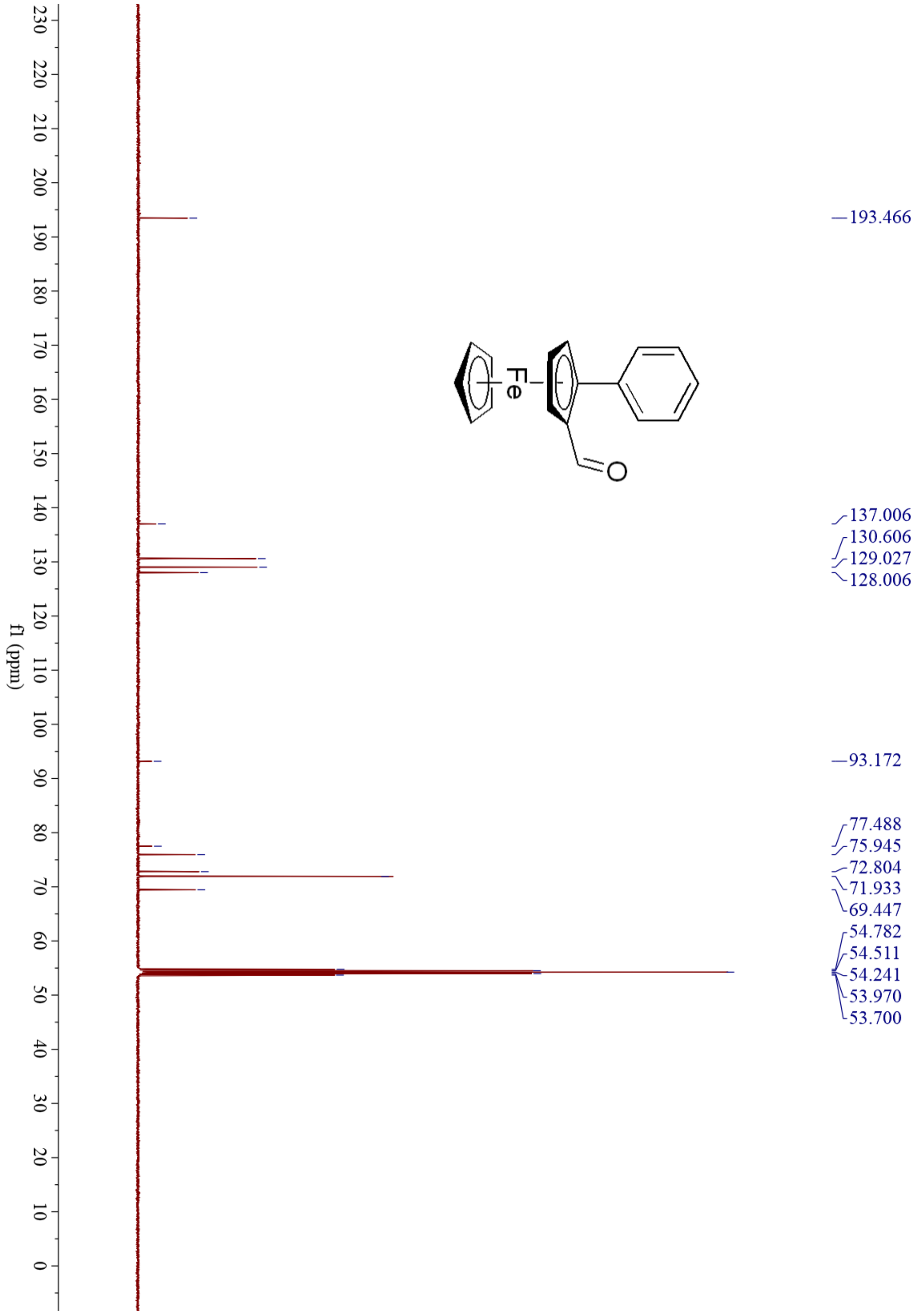

HPLC analysis of 3ag

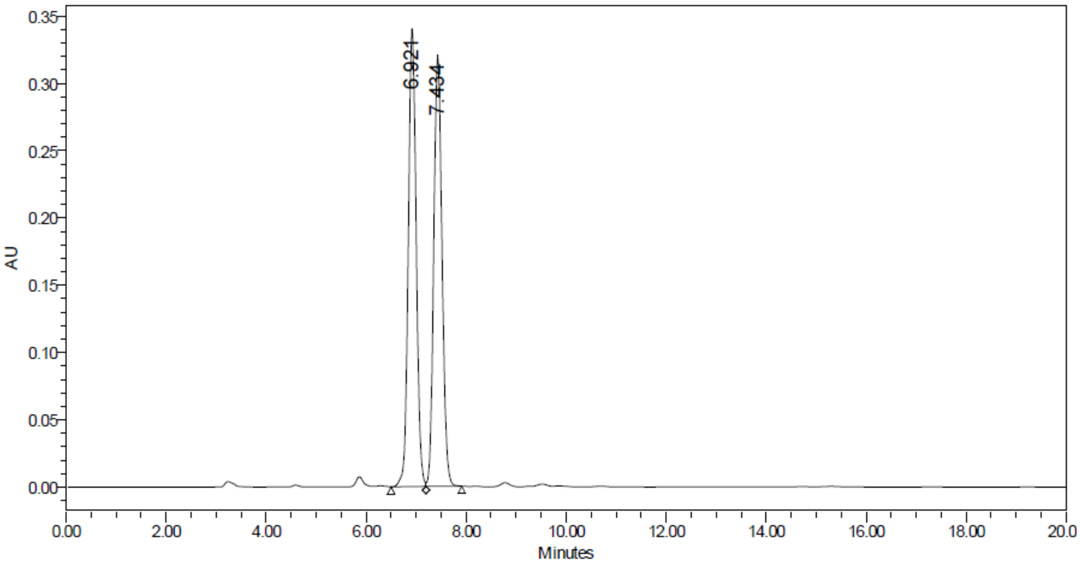

	RT	Area	% Area	Height
1	6.921	3745853	50.44	340941
2	7.434	3680079	49.56	320260

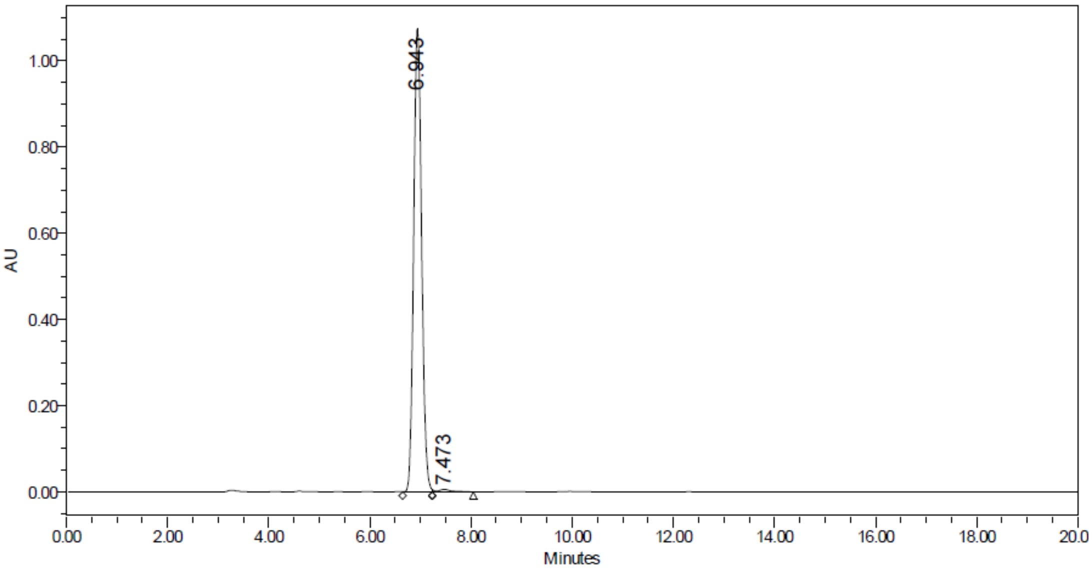

	RT	Area	% Area	Height
1	6.943	11813740	99.11	1078095
2	7.473	105809	0.89	5845

^{1}H NMR spectra of 3ah

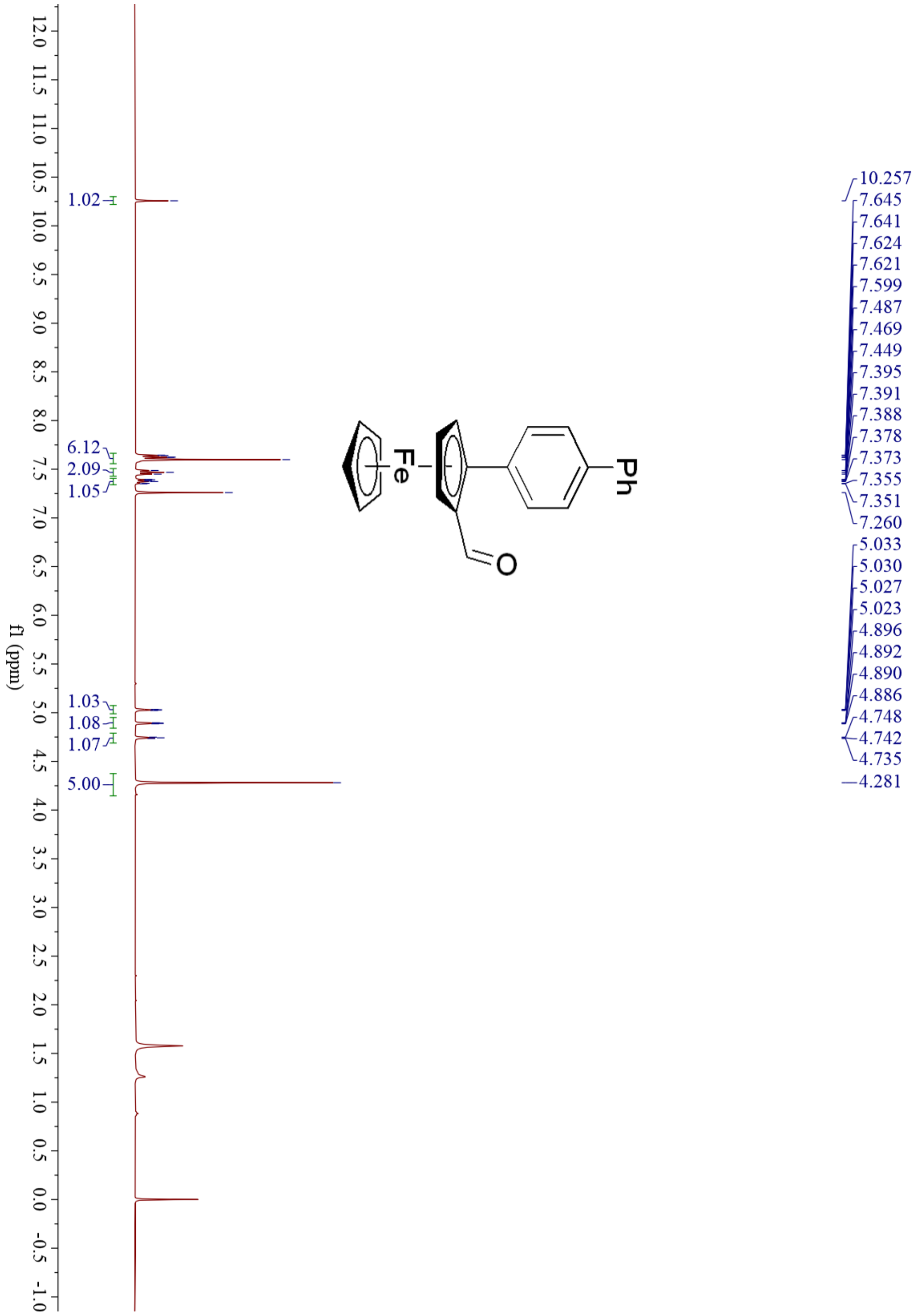

^{13}C NMR spectra of 3ah

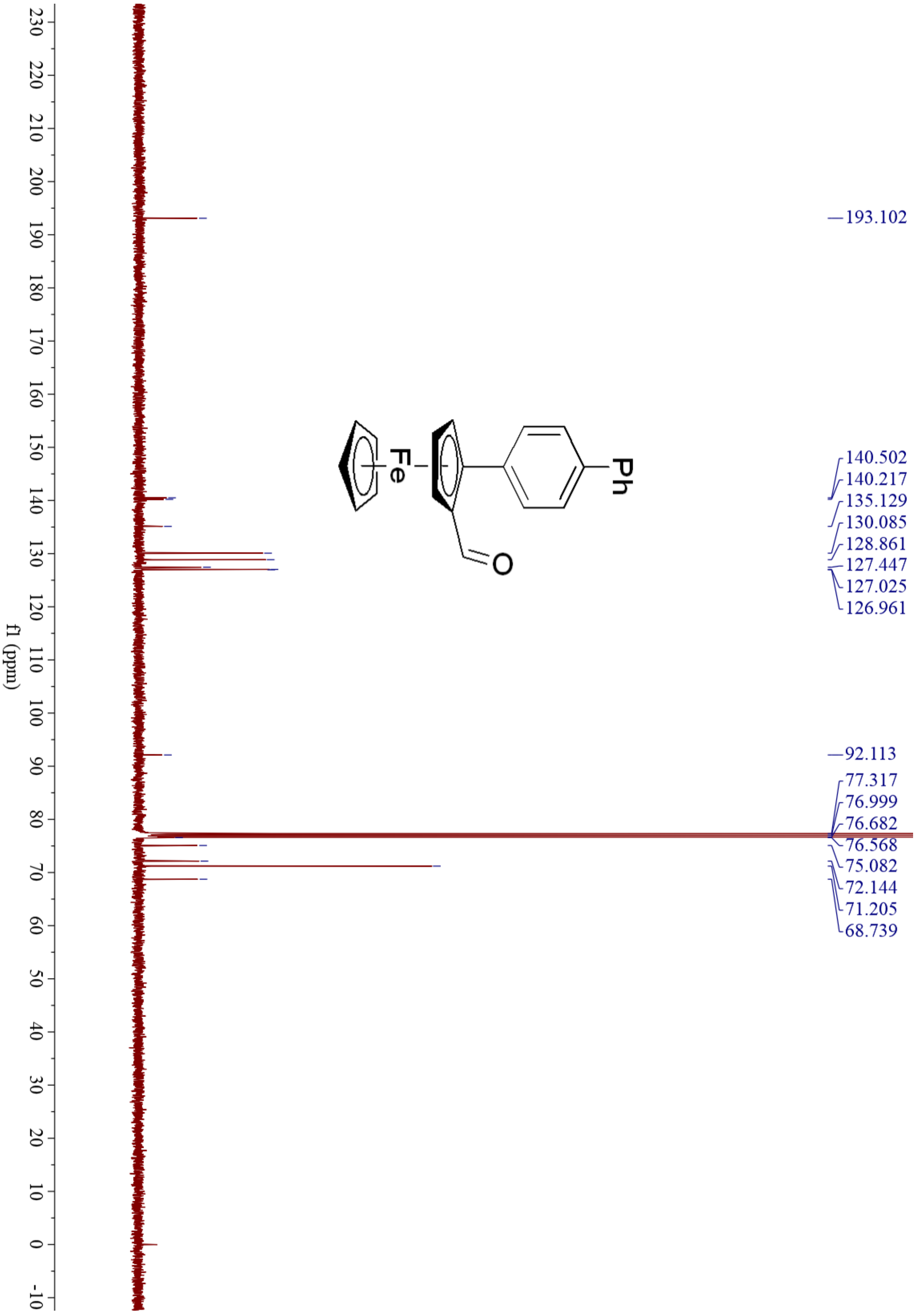

HPLC analysis of 3ah

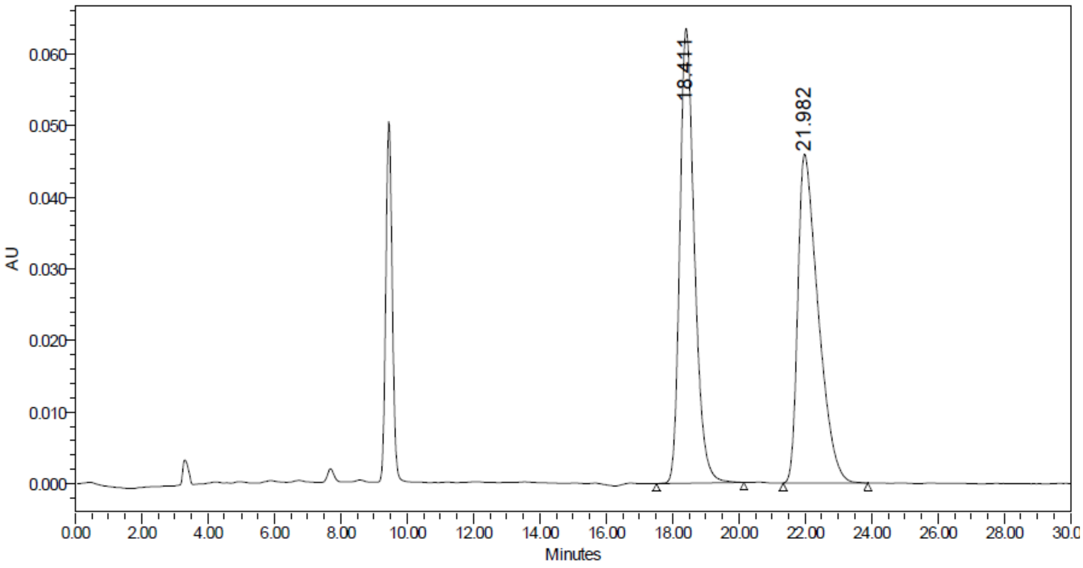

	RT	Area	% Area	Height
1	18.411	1946384	49.87	63467
2	21.982	1956533	50.13	45883

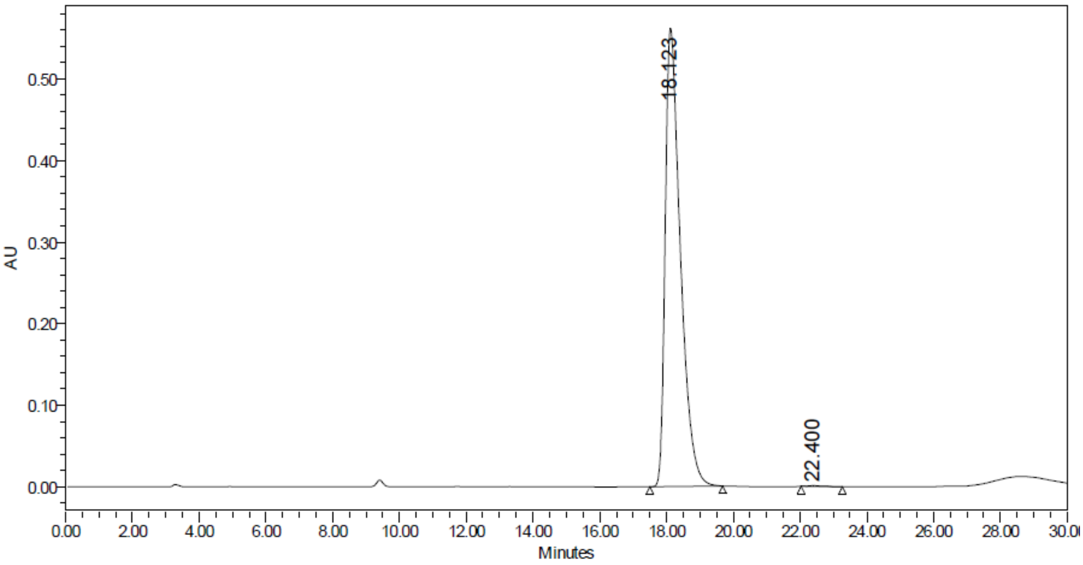

	RT	Area	% Area	Height
1	18.123	17667729	99.83	561909
2	22.400	30839	0.17	982

^{1}H NMR spectra of 3ai

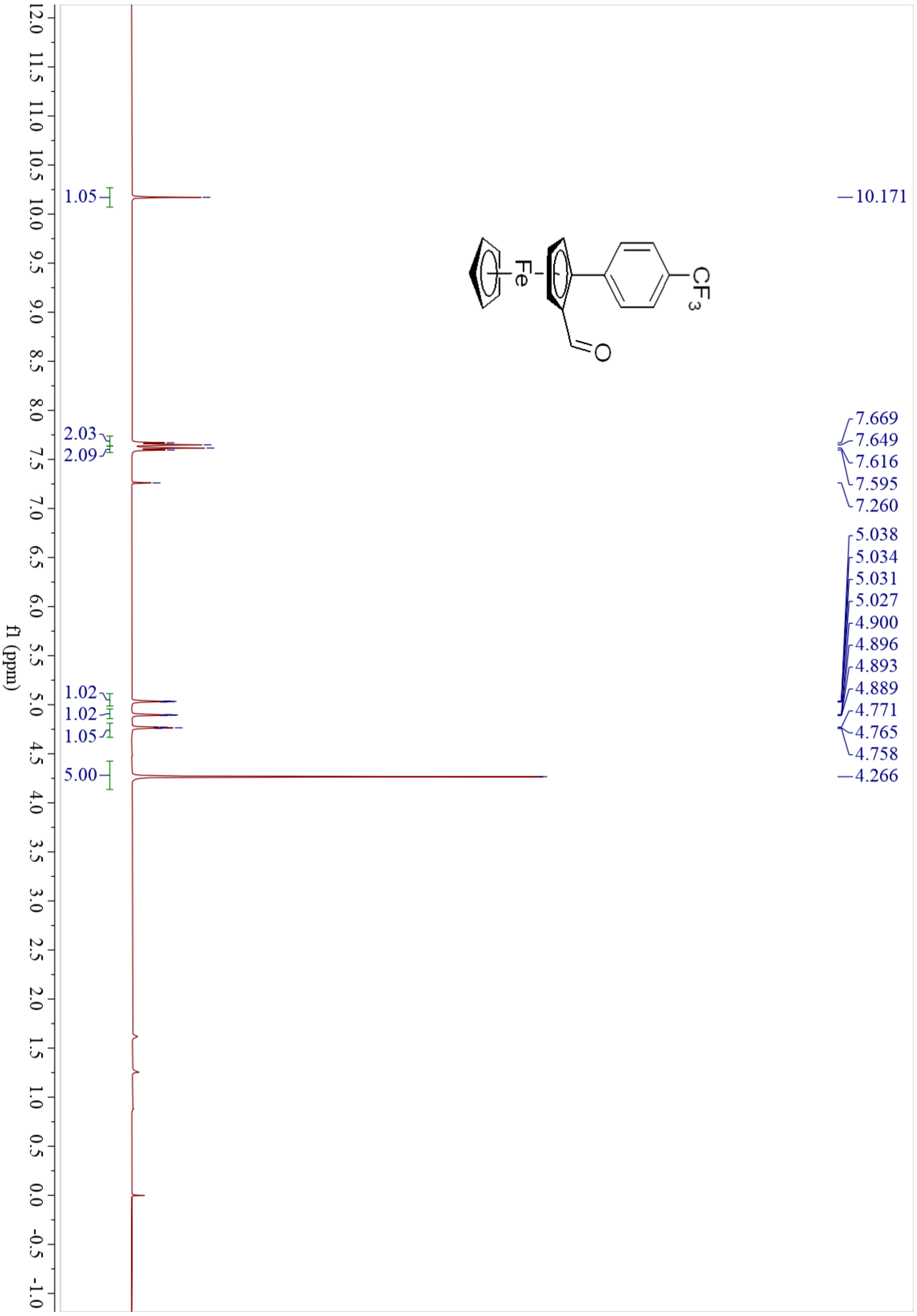

^{13}C NMR spectra of 3ai

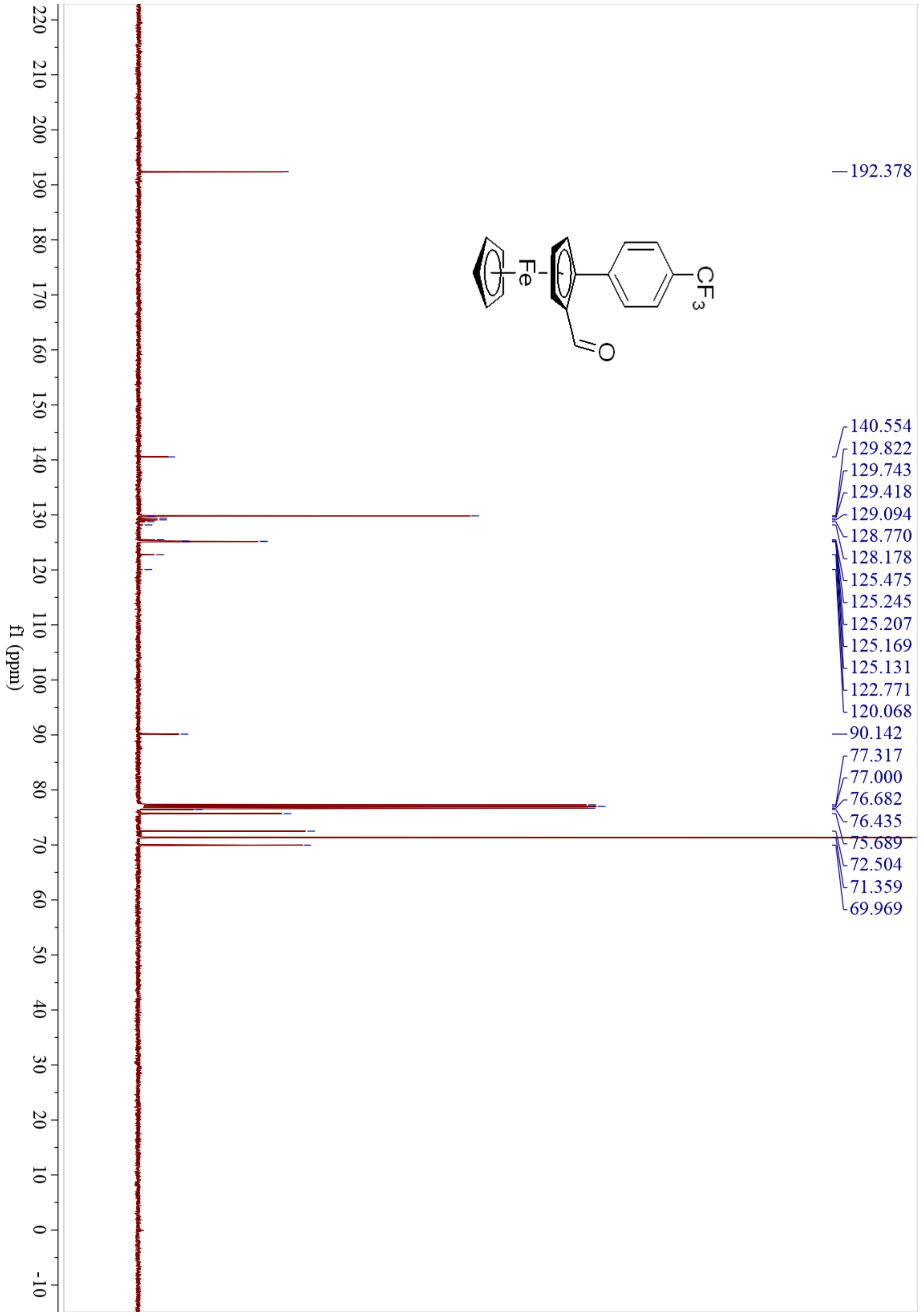

^{19}F NMR spectra of 3ai

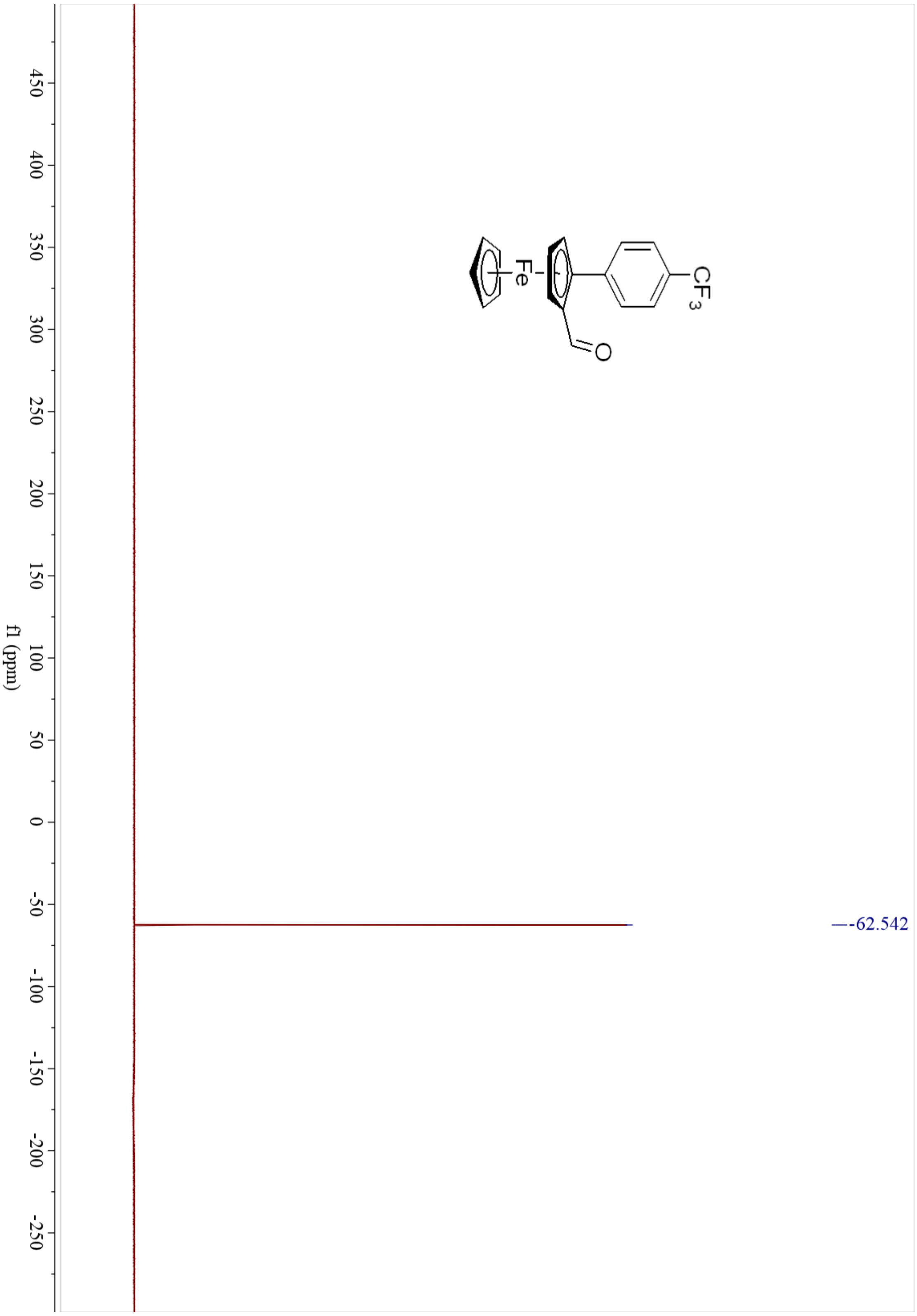

HPLC analysis of 3ai

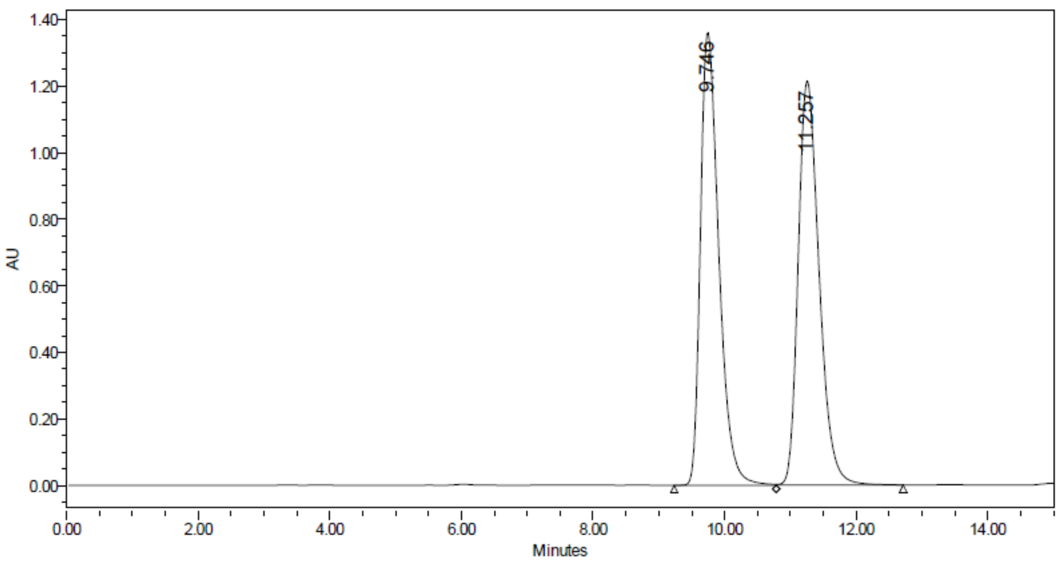

	RT	Area	% Area	Height
1	9.746	26468455	49.62	1360036
2	11.257	26875018	50.38	1215491

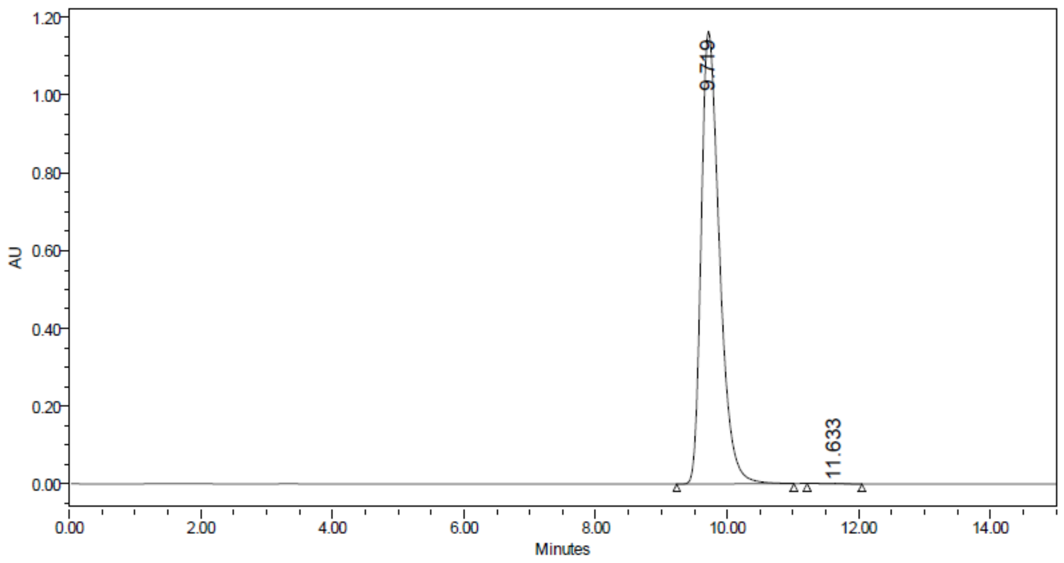

	RT	Area	% Area	Height
1	9.719	22530346	99.96	1163729
2	11.633	9865	0.04	-353

^{1}H NMR spectra of 3aj

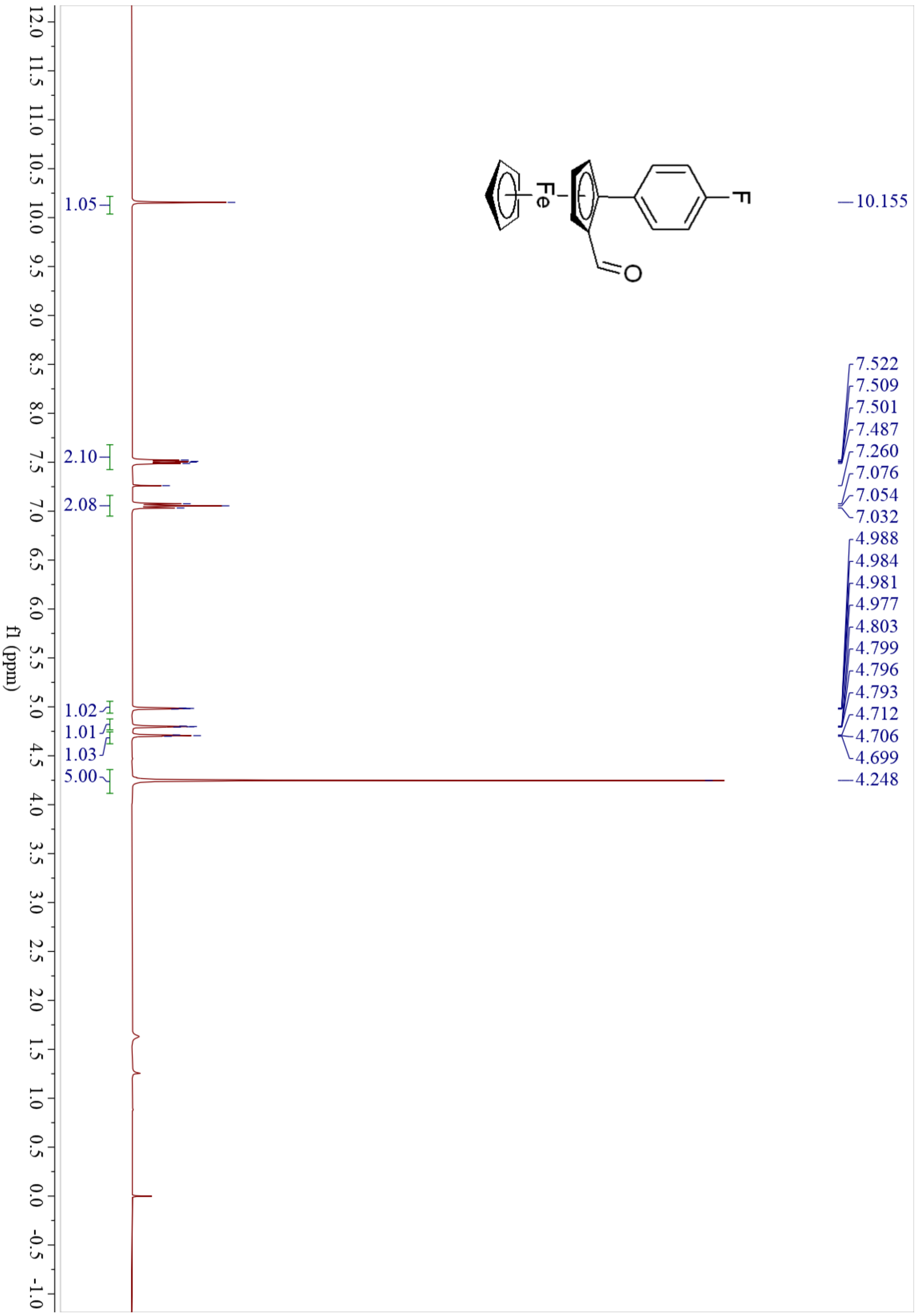

^{13}C NMR spectra of 3aj

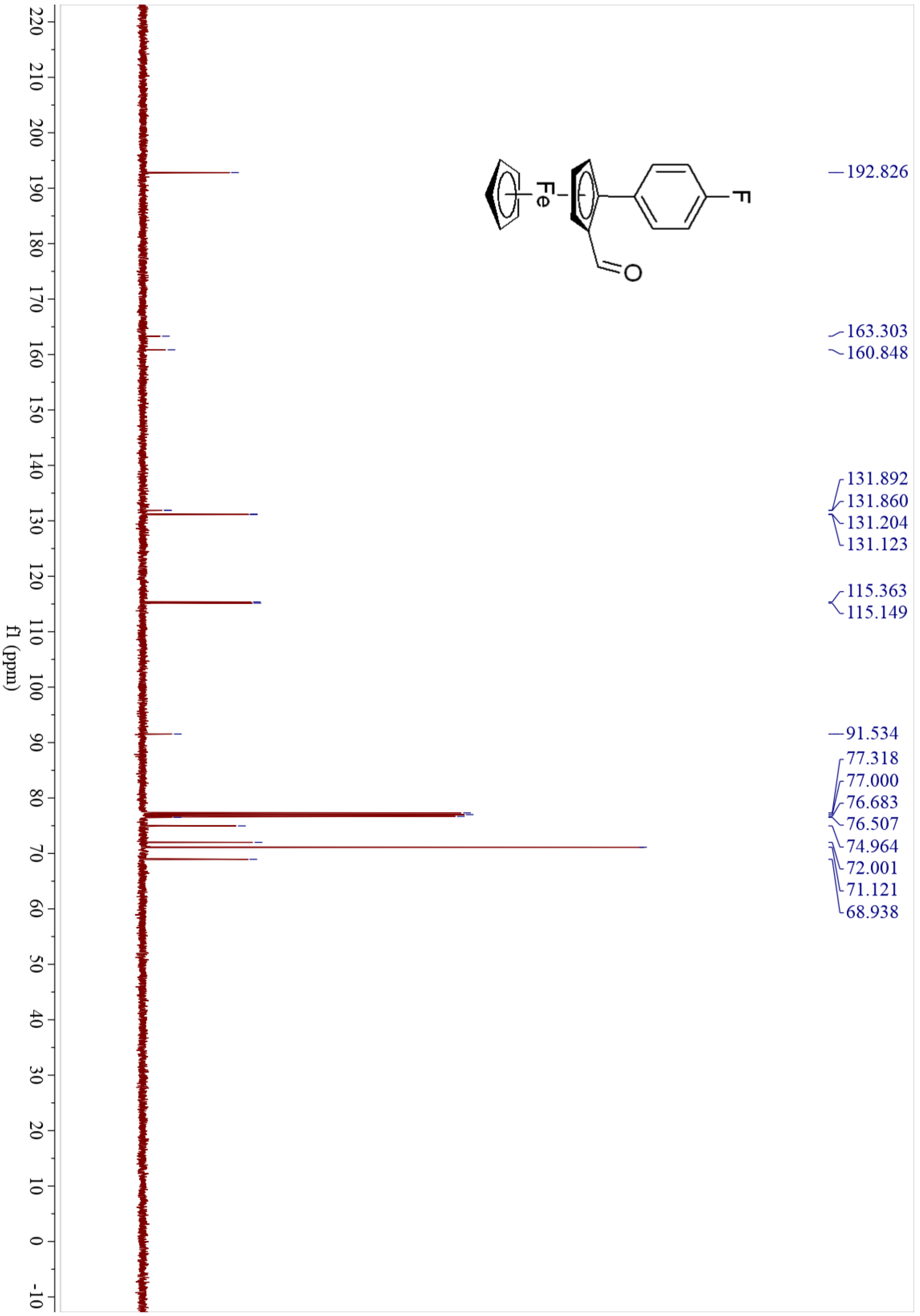

^{19}F NMR spectra of 3aj

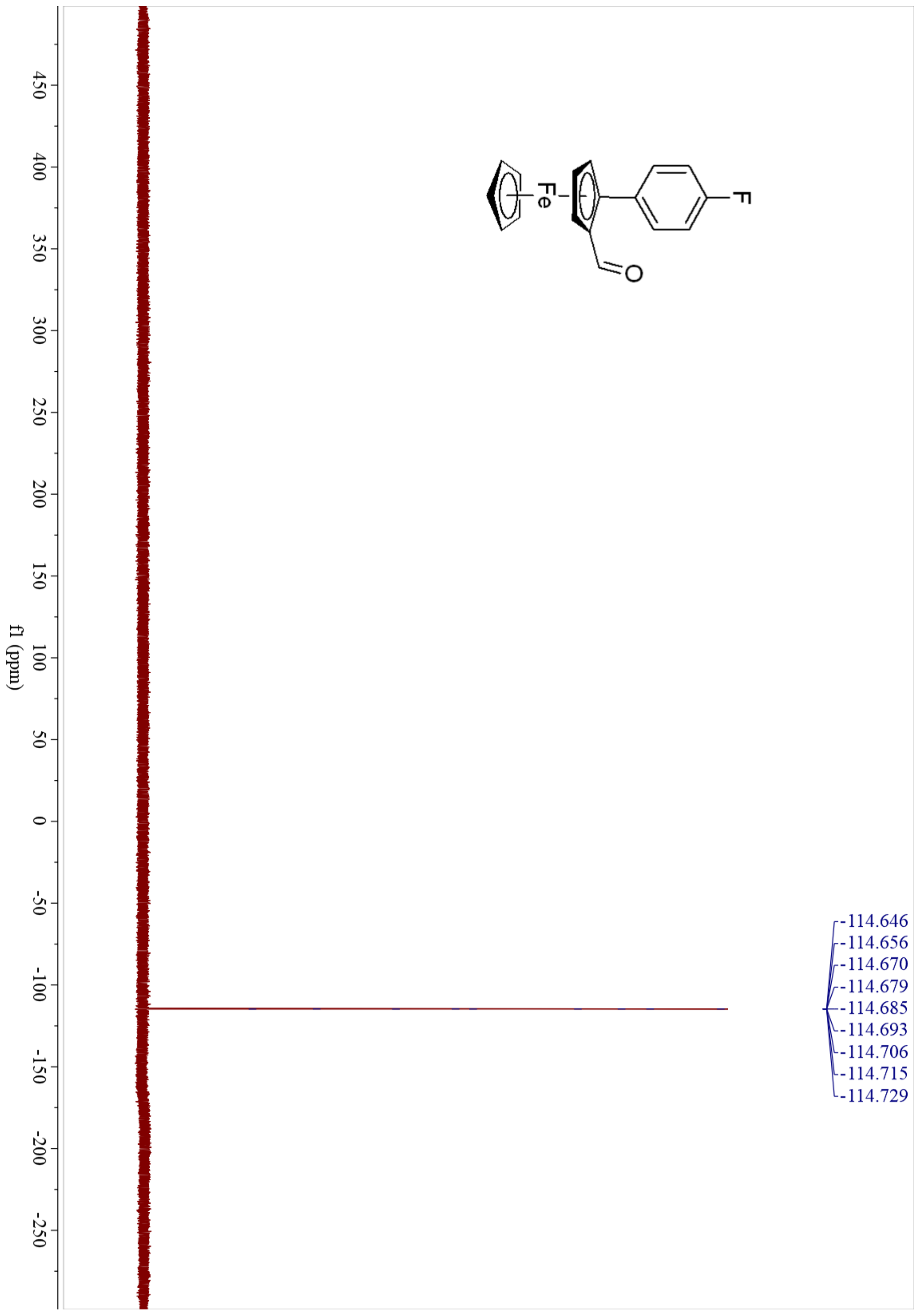

HPLC analysis of 3aj

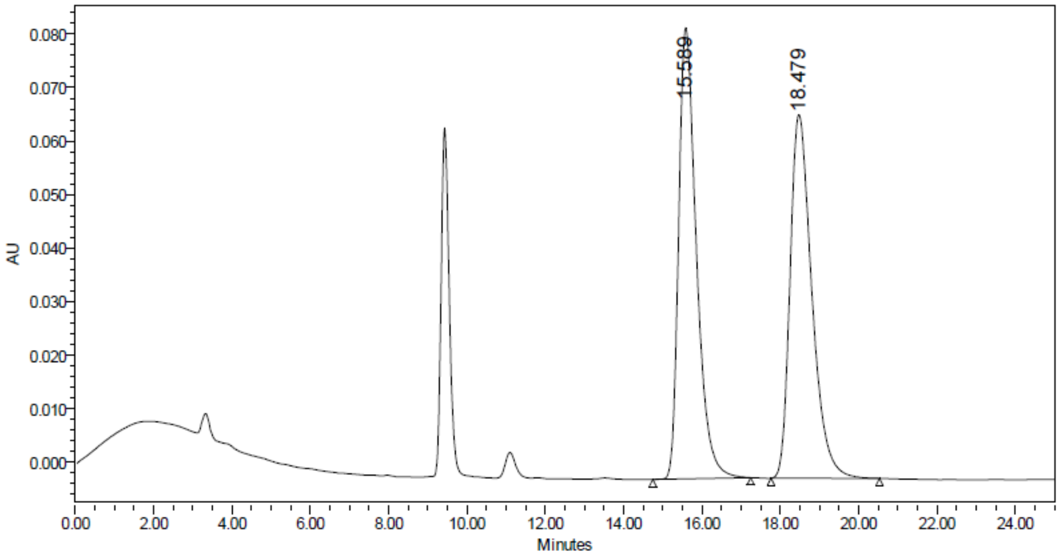

	RT	Area	% Area	Height
1	15.589	2647094	50.23	84274
2	18.479	2623219	49.77	67958

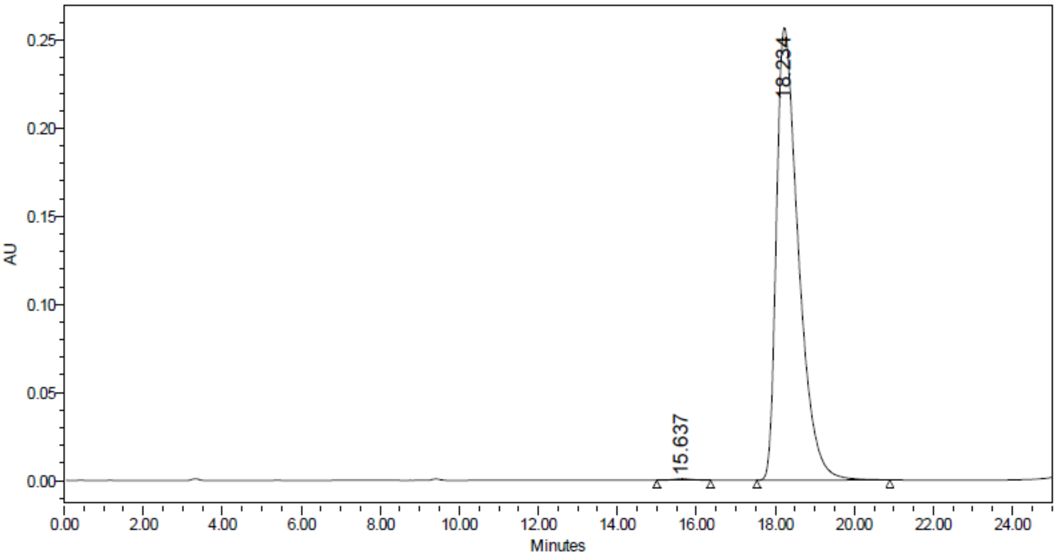

	RT	Area	% Area	Height
1	15.637	27183	0.27	877
2	18.234	9917817	99.73	256733

^{1}H NMR spectra of 3ak

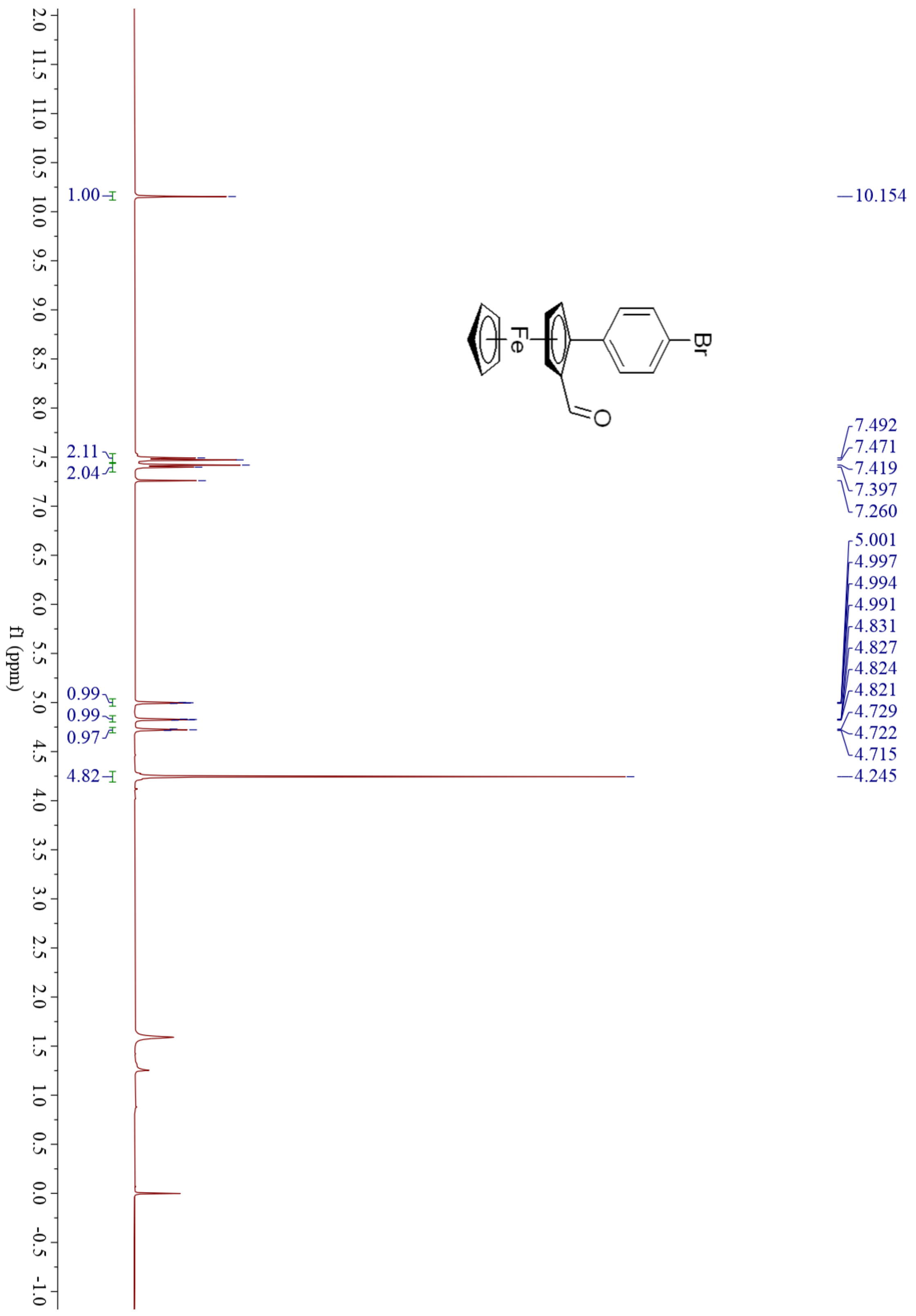

^{13}C NMR spectra of 3ak

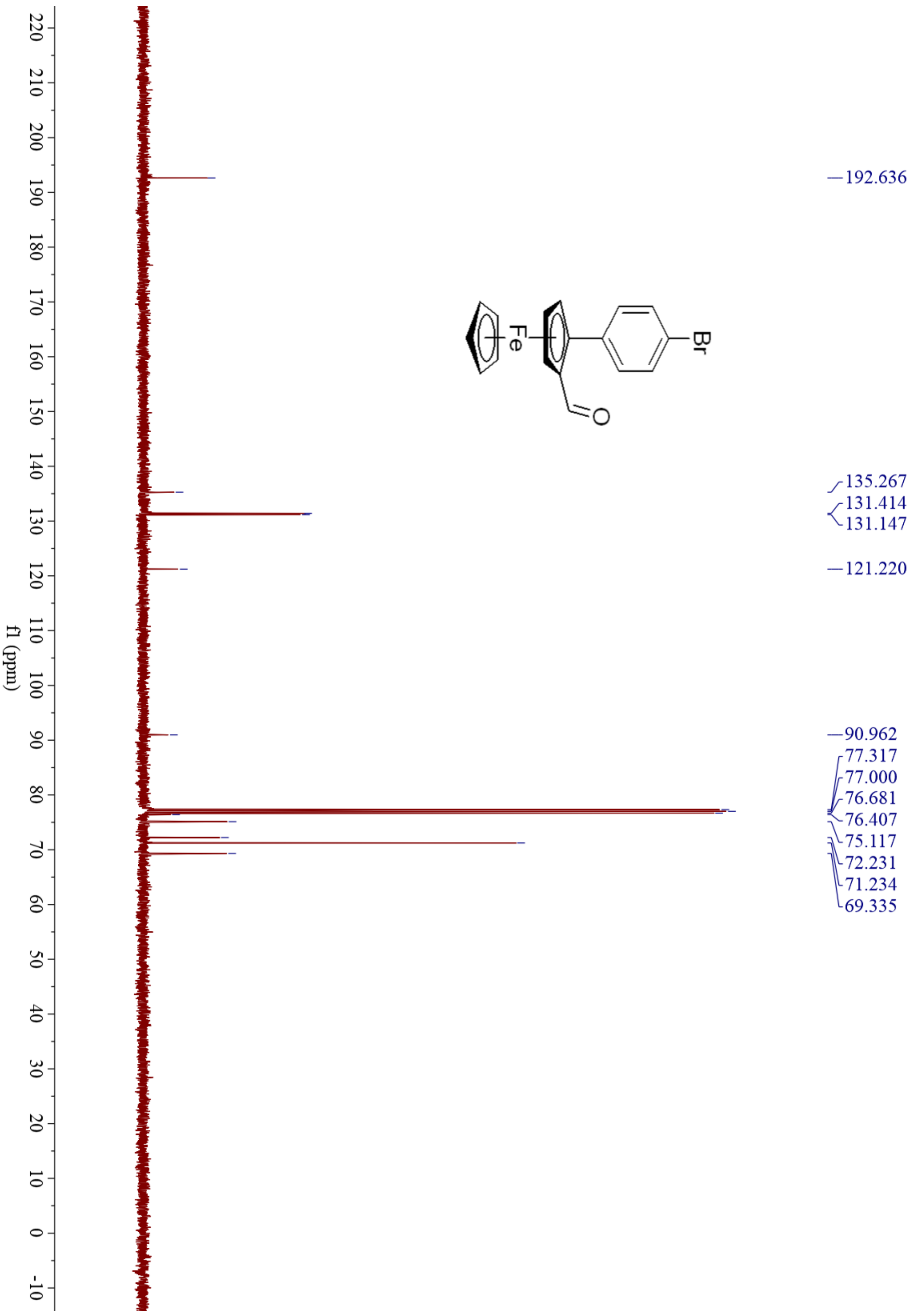

HPLC analysis of 3ak

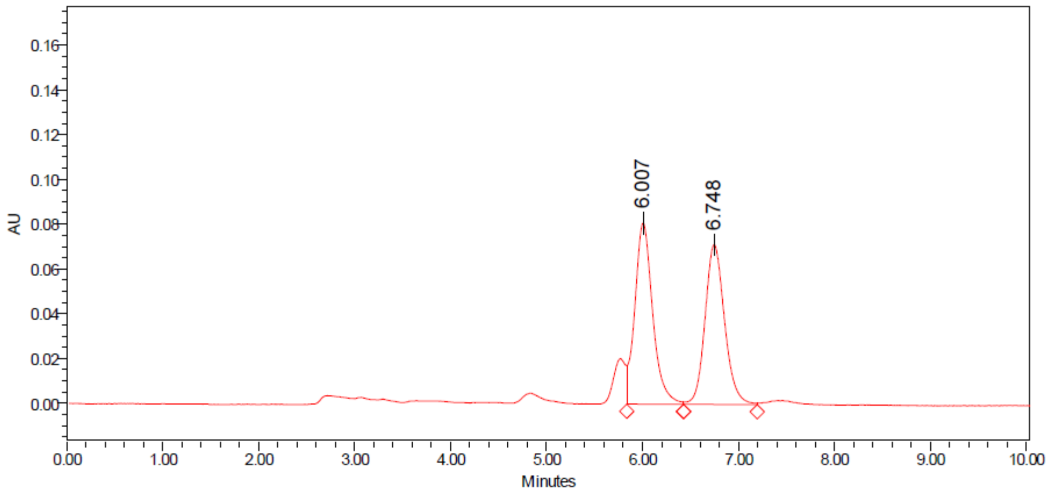

Peak Results

	SampleName	RT	Width (sec)	Height	Area	% Area
1	LCX-23-61-RAC	6.007	35.500	80860	1048393	50.97
2	LCX-23-61-RAC	6.748	46.000	71317	1008595	49.03

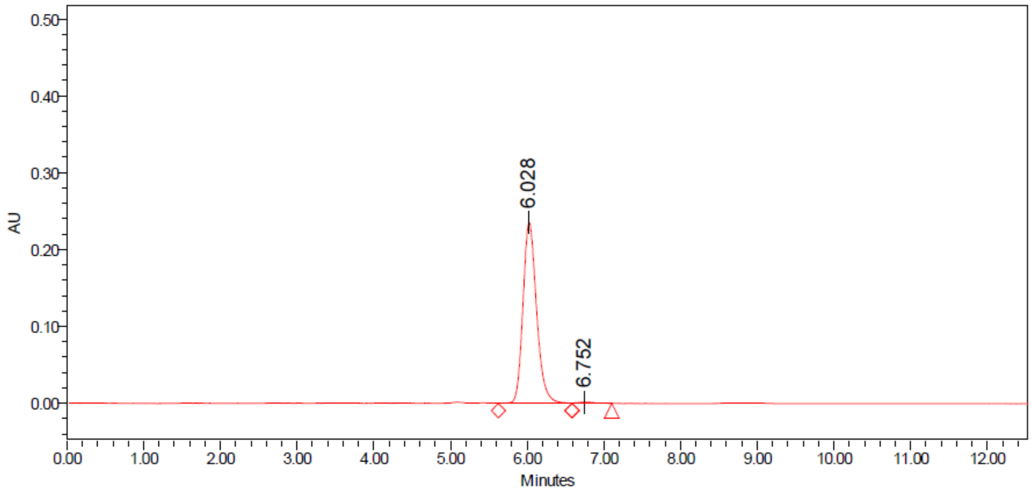

Peak Results

	SampleName	RT	Width (sec)	Height	Area	% Area
1	LCX-23-61-chiral	6.028	57.600	235264	2904950	99.15
2	LCX-23-61-chiral	6.752	31.000	1649	24808	0.85

^{1}H NMR spectra of 3al

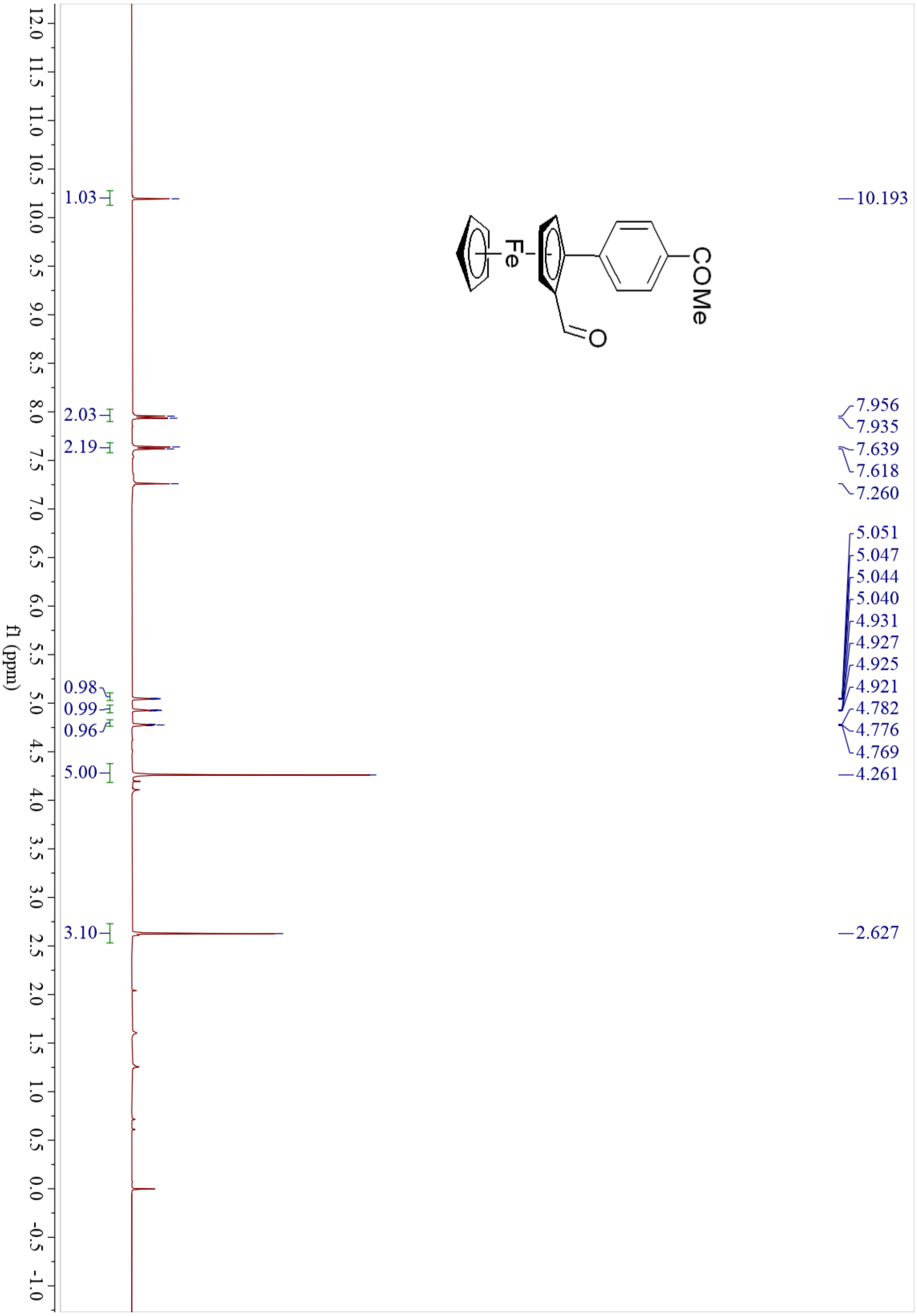

^{13}C NMR spectra of 3al

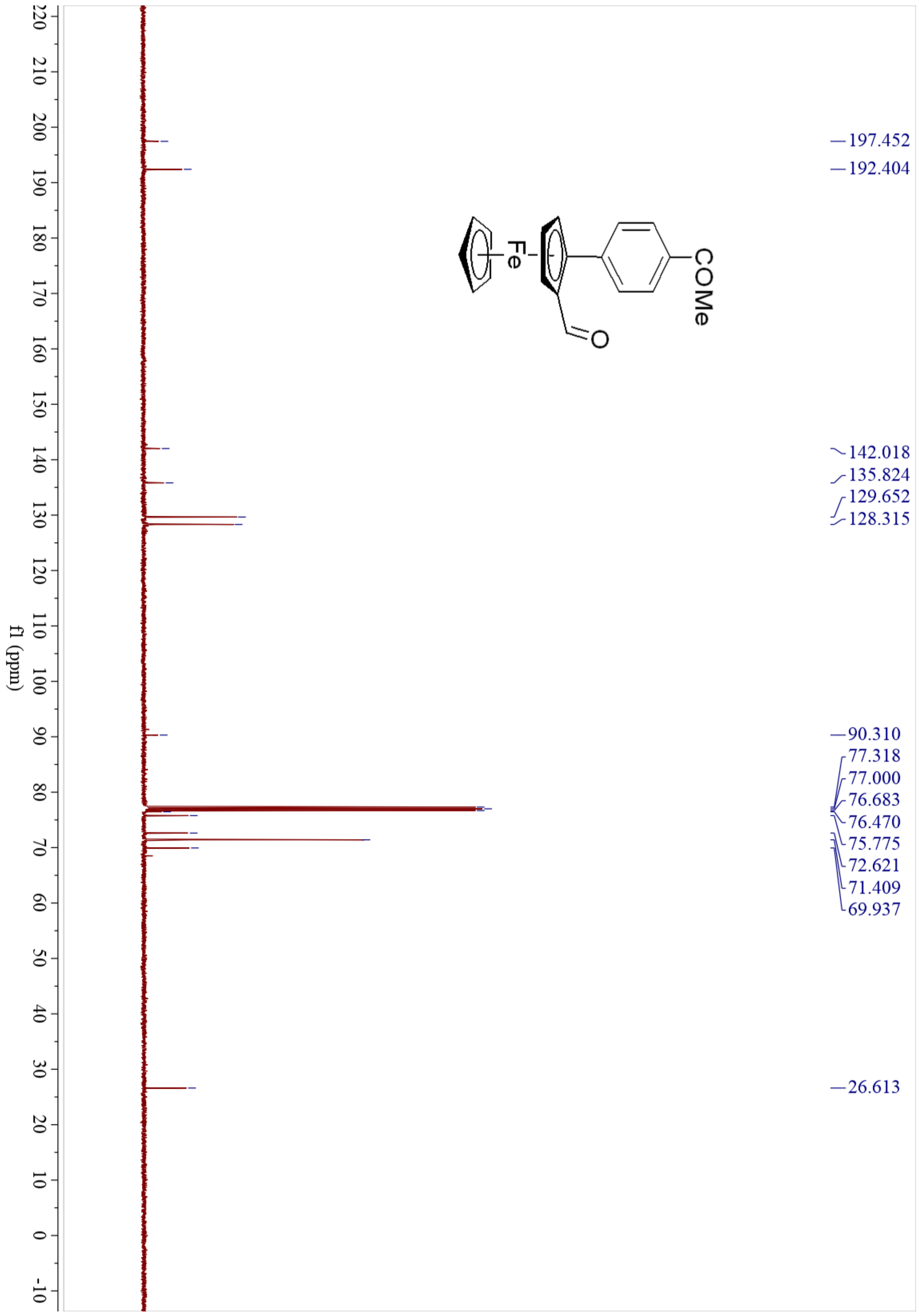

HPLC analysis of 3al

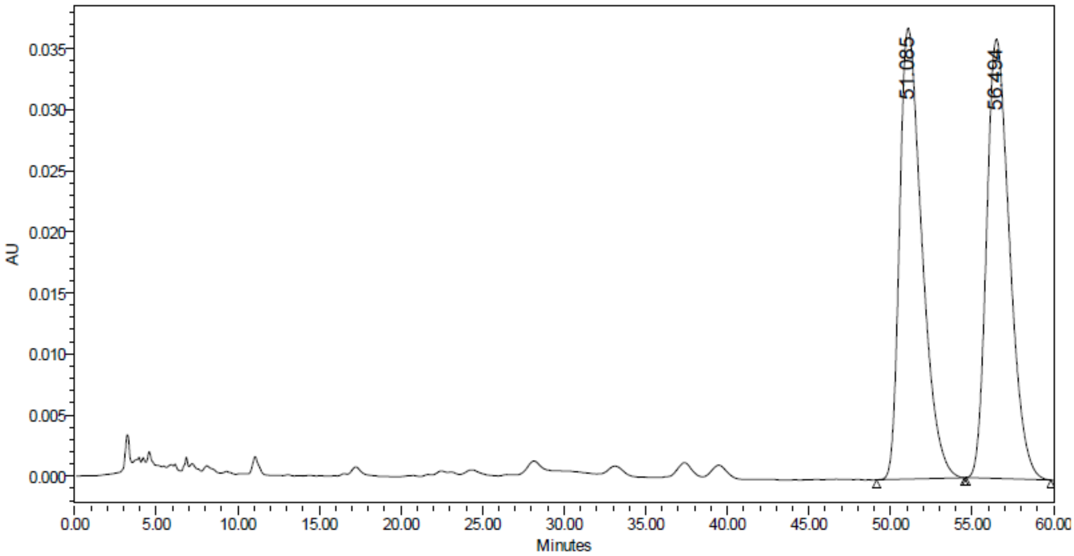

	RT	Area	% Area	Height
1	51.085	3517091	49.96	36916
2	56.494	3522125	50.04	35966

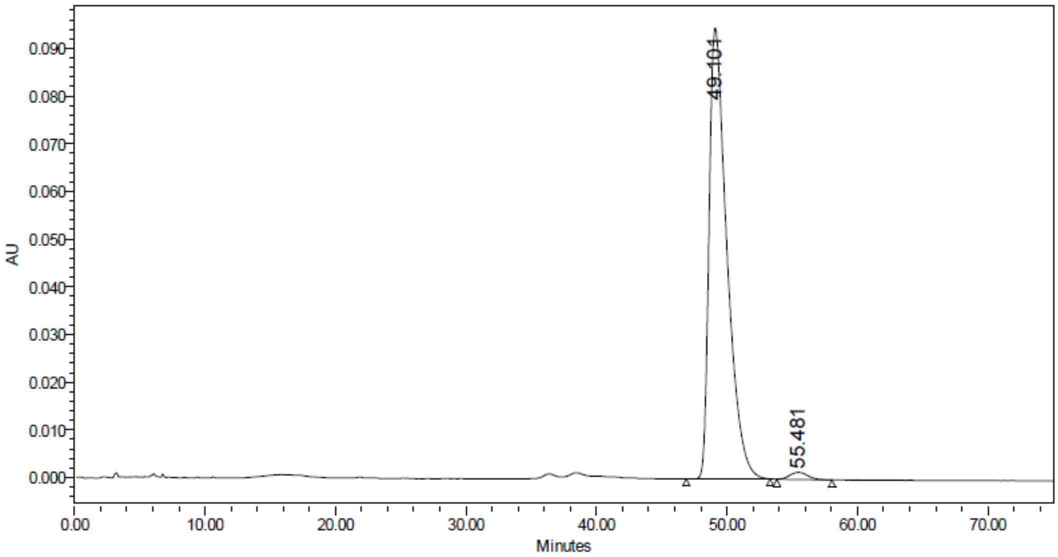

	RT	Area	% Area	Height
1	49.101	8934578	98.43	94467
2	55.481	142846	1.57	1509

^{1}H NMR spectra of 3am

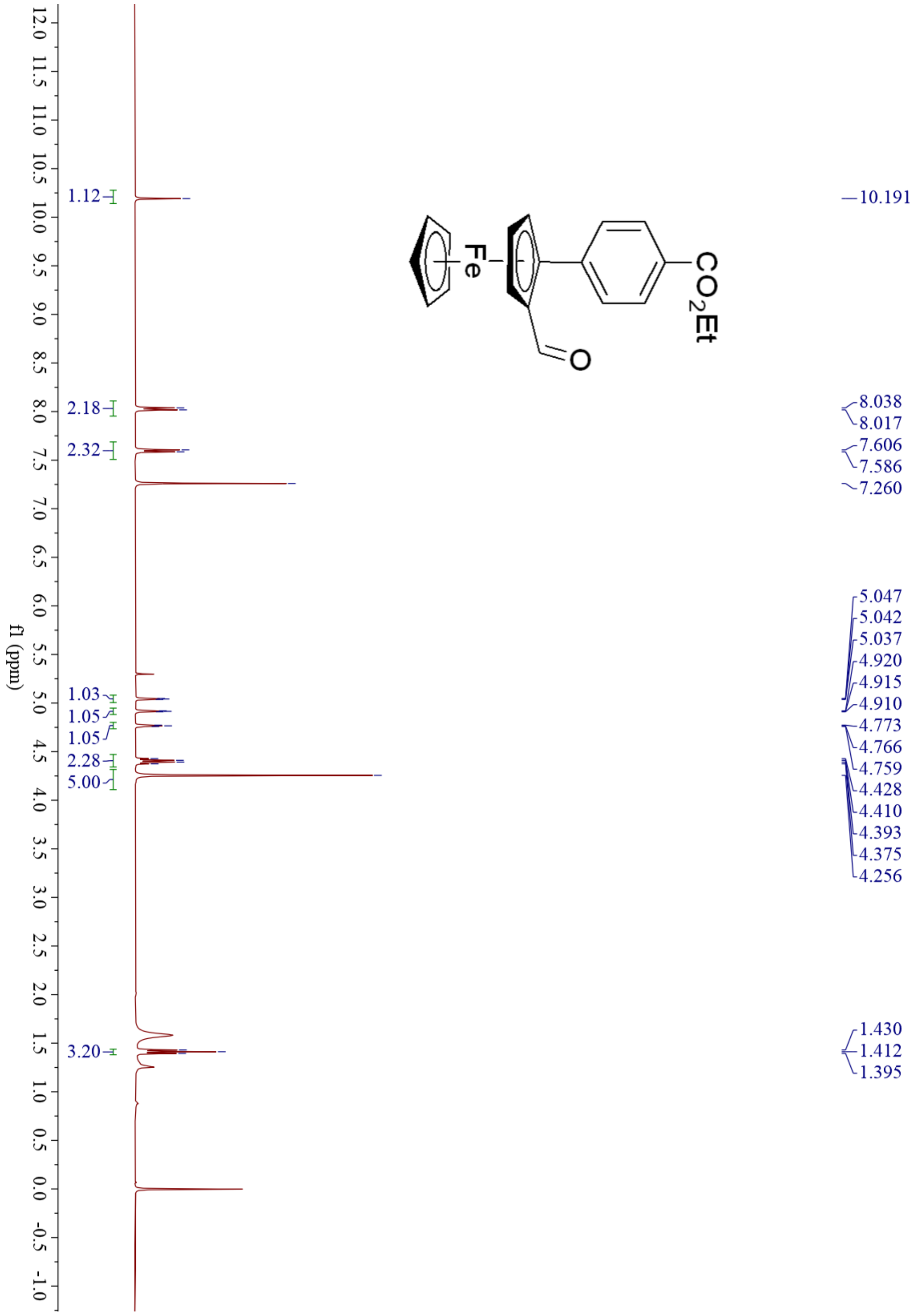

^{13}C NMR spectra of 3am

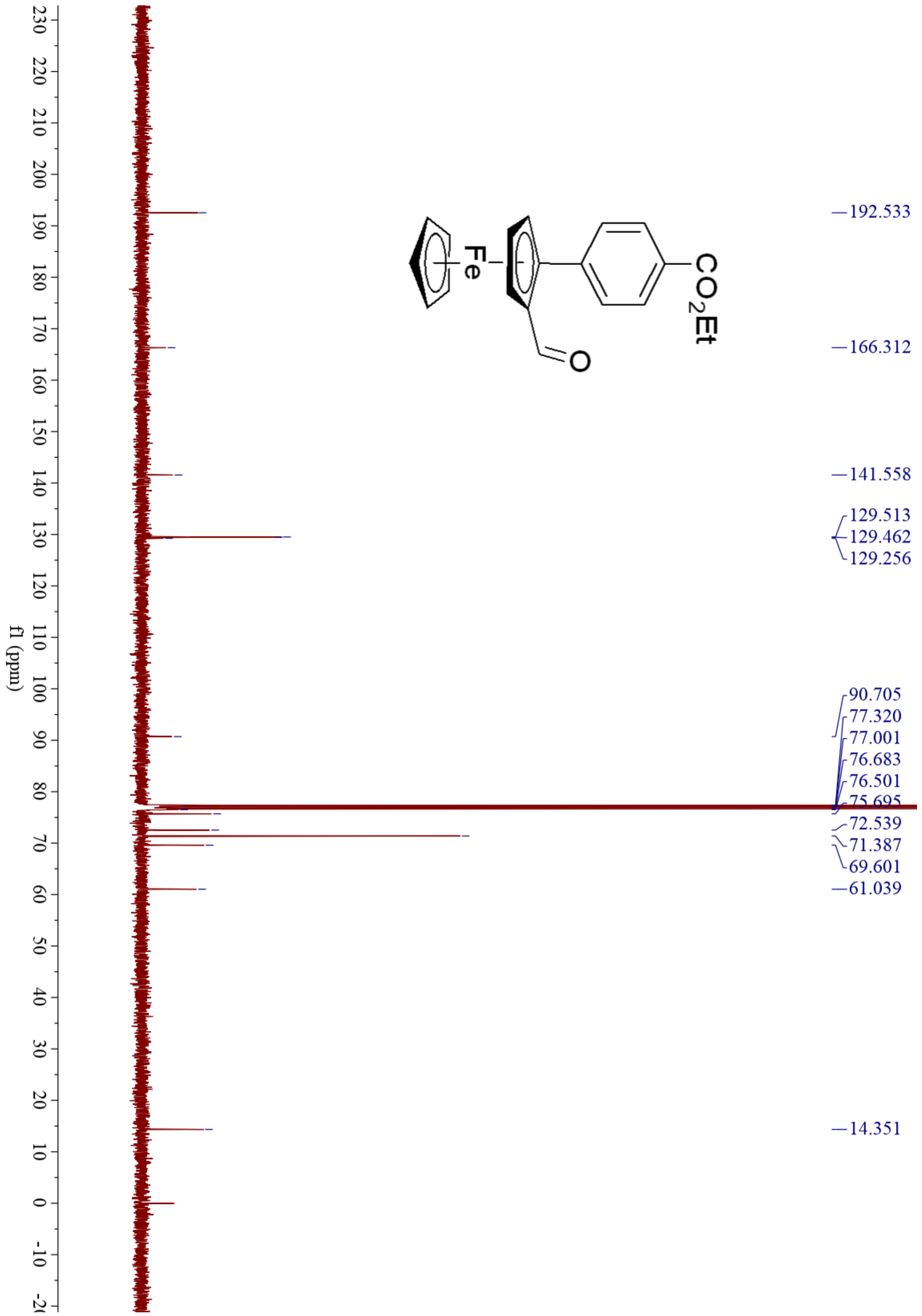

HPLC analysis of 3am

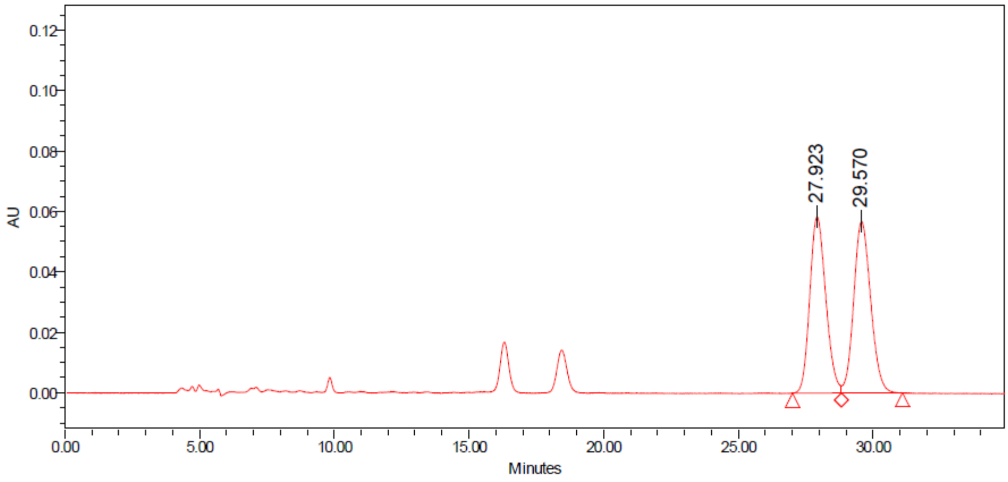

Peak Results

	SampleName	RT	Width (sec)	Height	Area	% Area
1	LCX-23-4-co2Et-rac	27.923	108.900	58334	2414727	49.29
2	LCX-23-4-co2Et-rac	29.570	136.200	56764	2484425	50.71

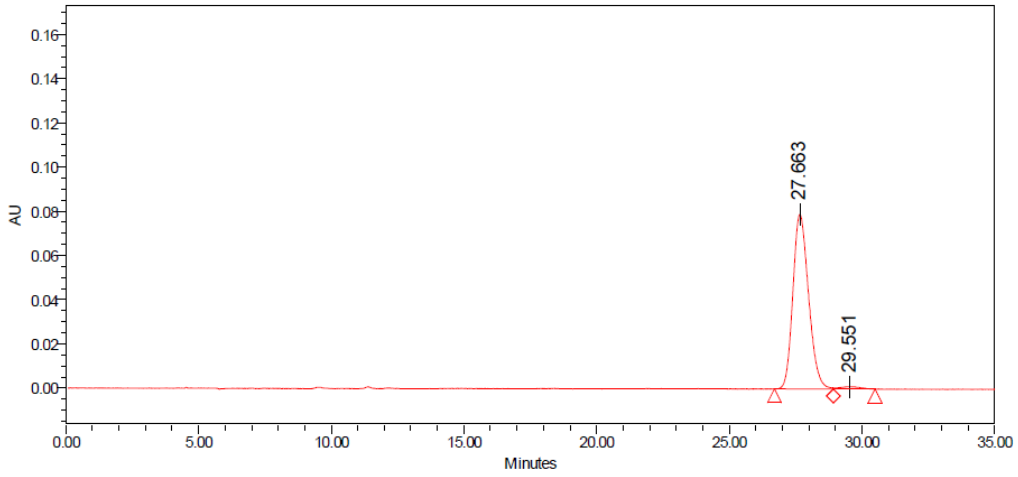

Peak Results

	SampleName	RT	Width (sec)	Height	Area	% Area
1	LCX-23-4-co2Et-chiral	27.663	133.600	79097	3253808	98.23
2	LCX-23-4-co2Et-chiral	29.551	93.900	1100	58768	1.77

^{1}H NMR spectra of 3an

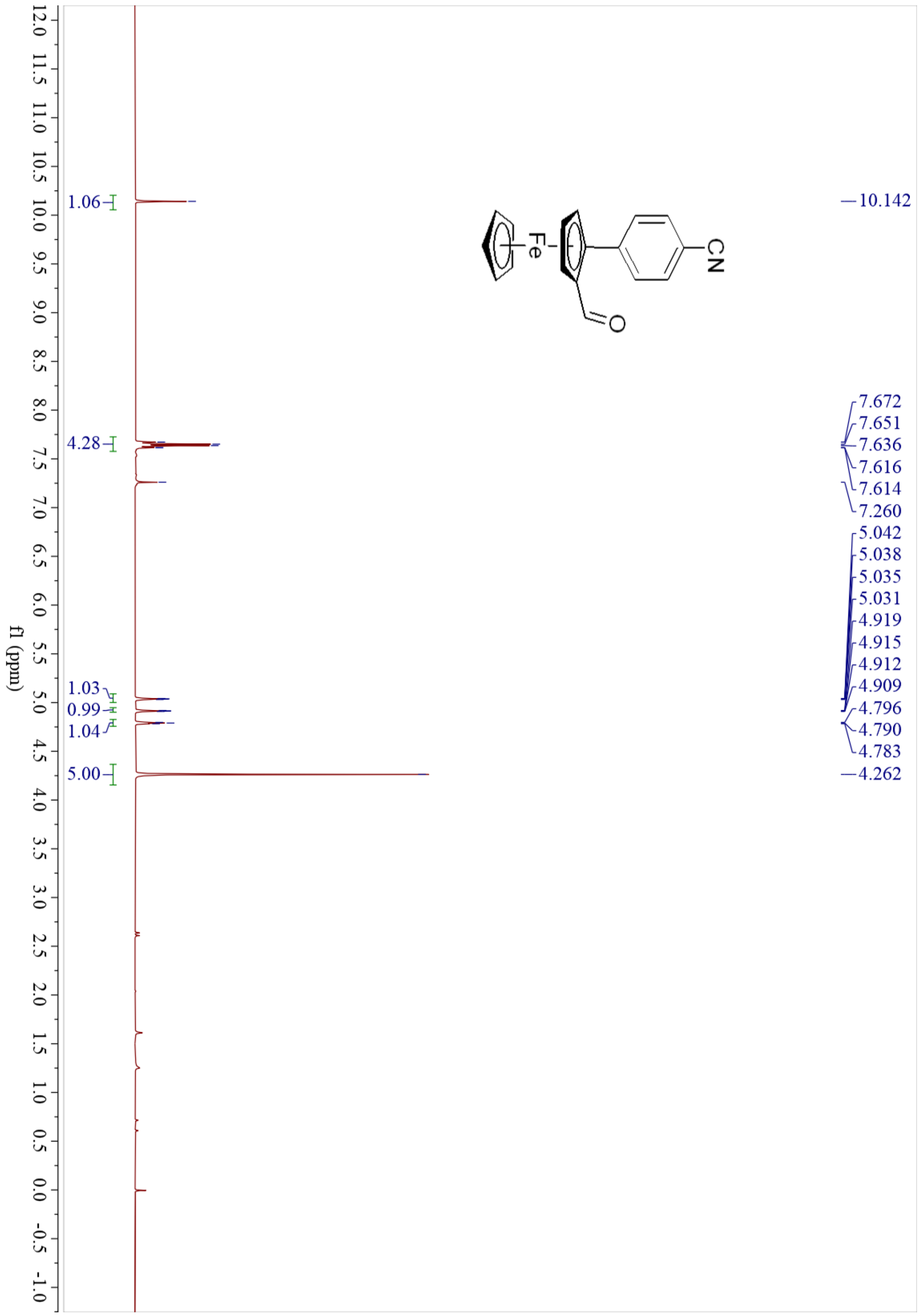

^{13}C NMR spectra of 3an

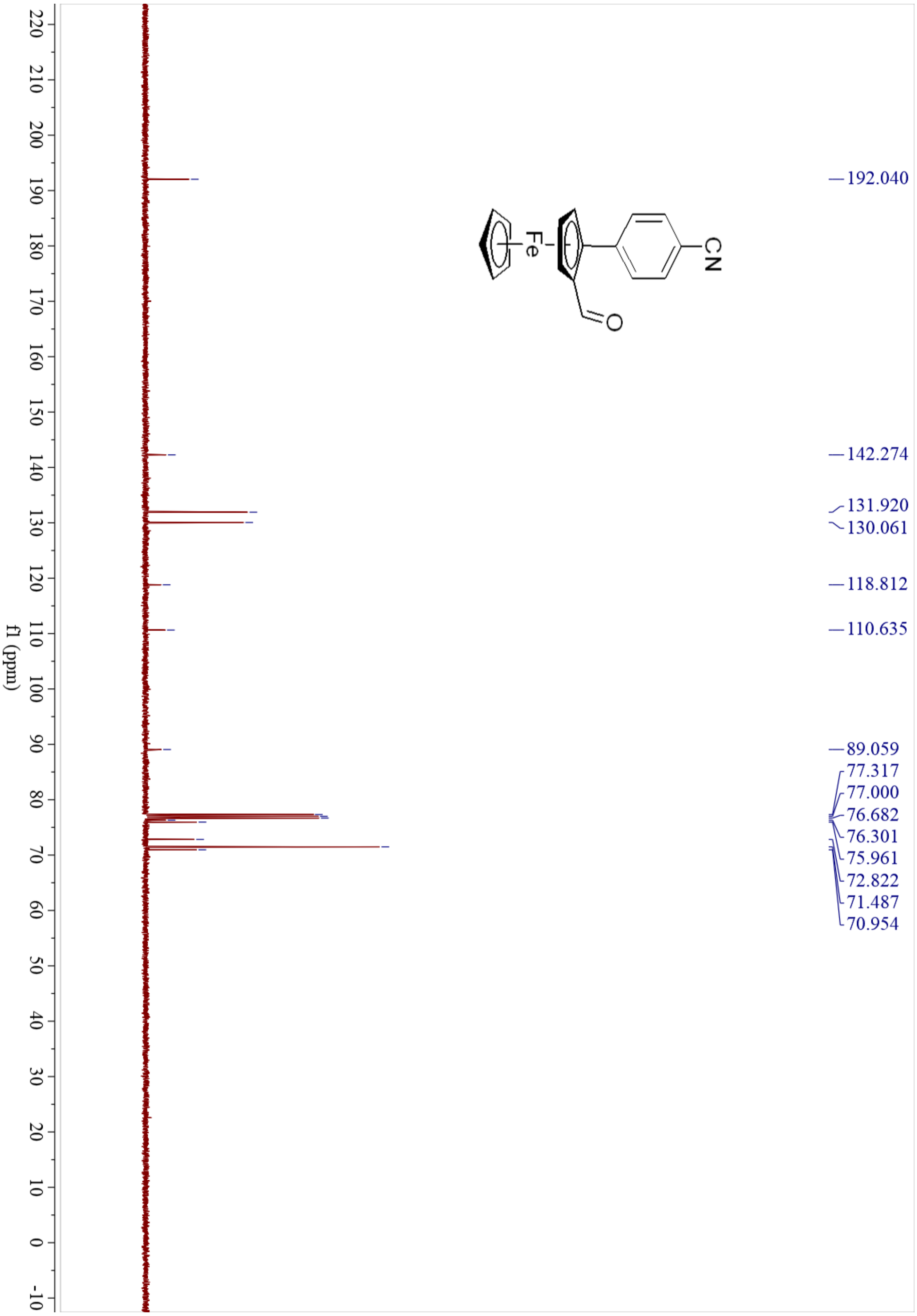

HPLC analysis of 3an

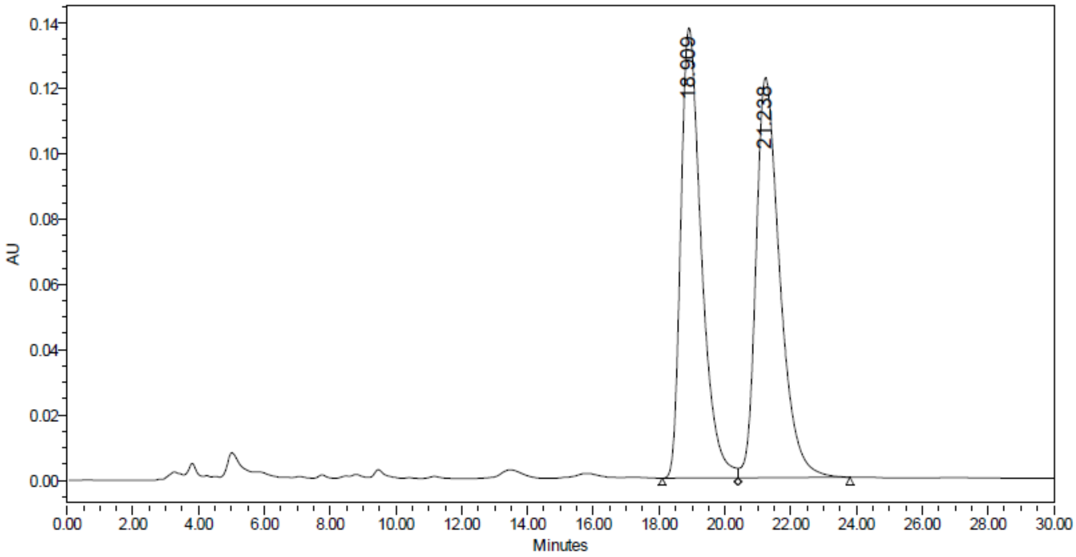

	RT	Area	% Area	Height
1	18.909	6008054	49.35	137864
2	21.238	6167370	50.65	122457

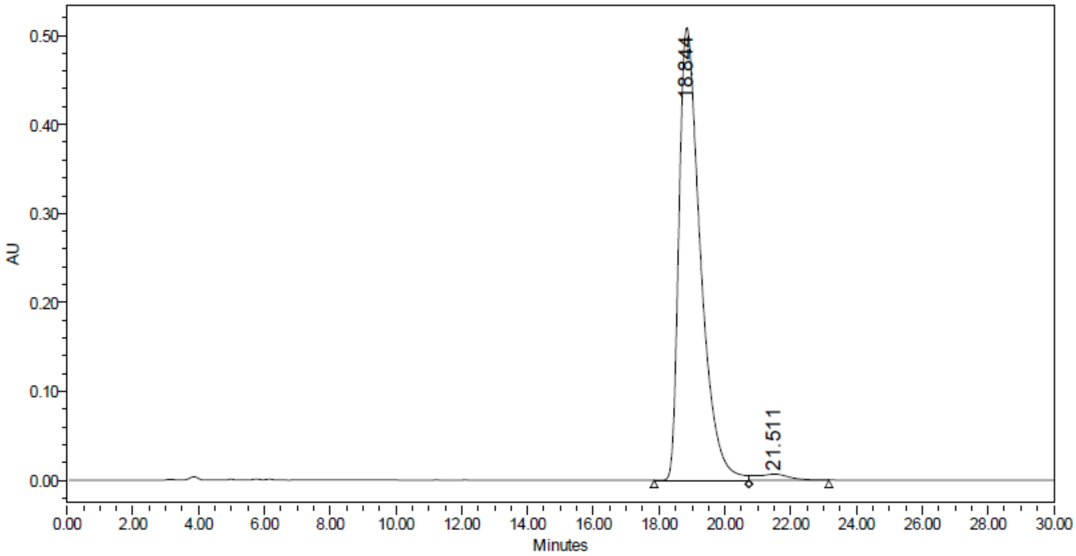

	RT	Area	% Area	Height
1	18.844	22976646	97.98	508602
2	21.511	474871	2.02	6872

^{1}H NMR spectra of 3ao

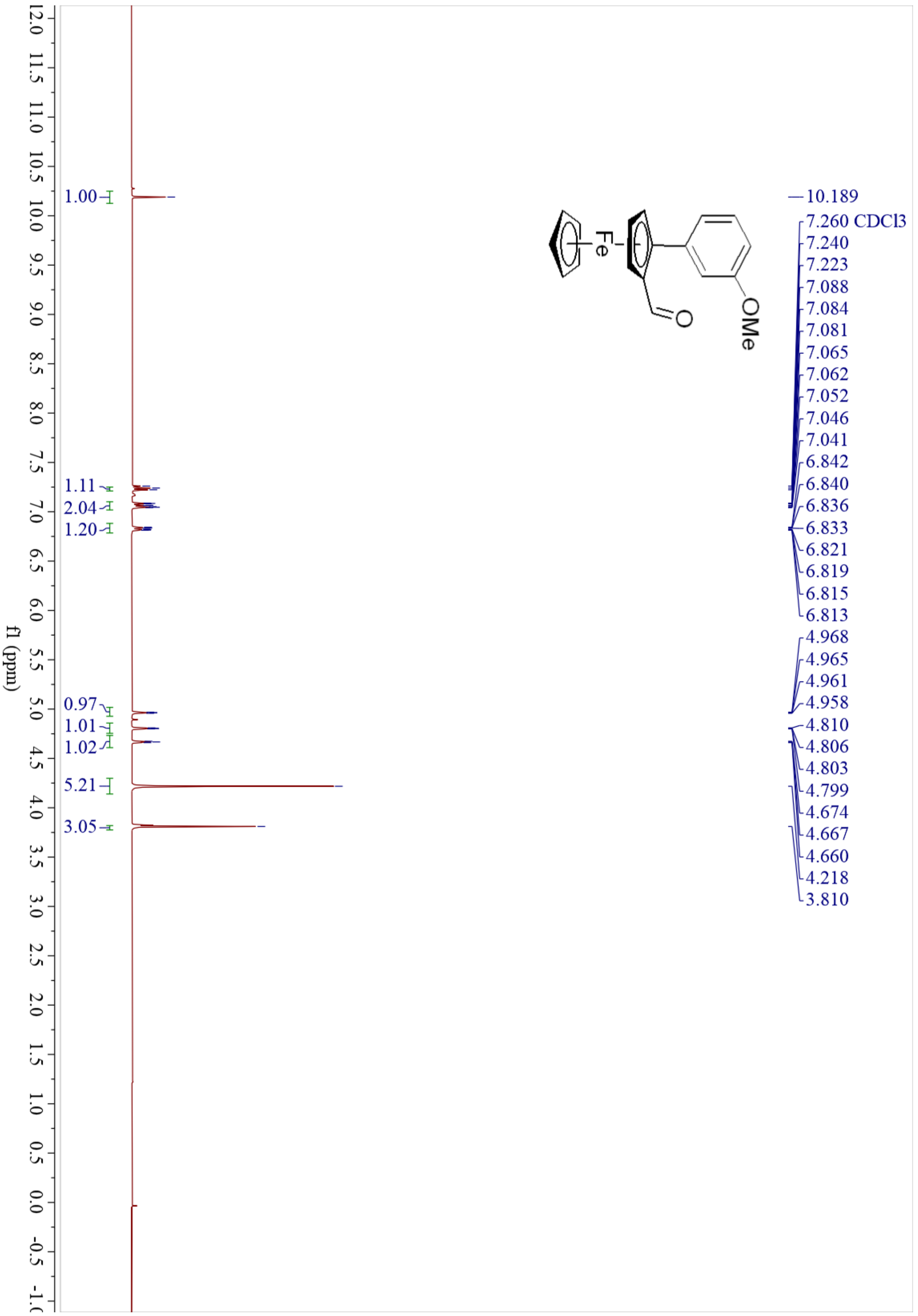

^{13}C NMR spectra of 3ao

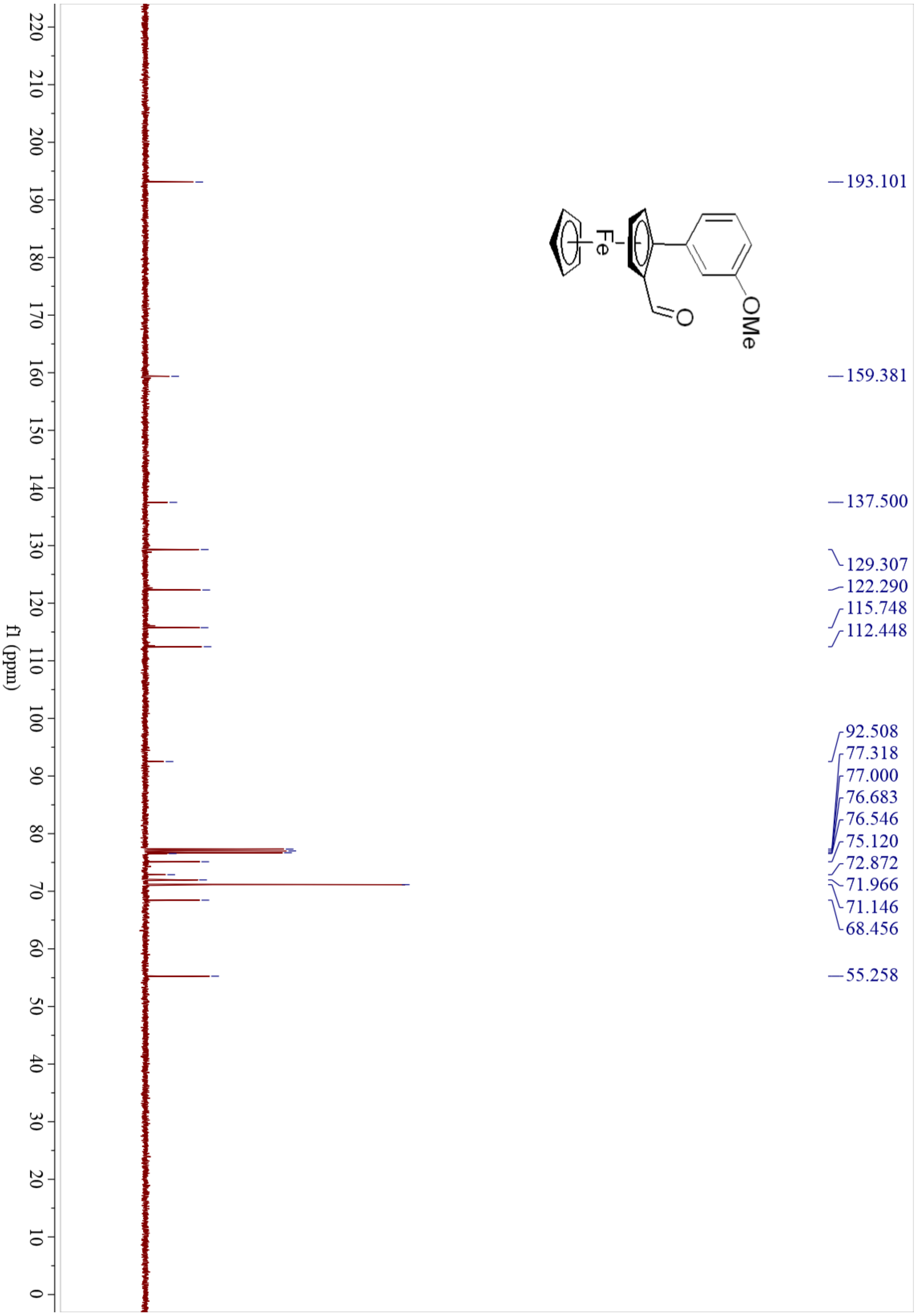

HPLC analysis of 3ao

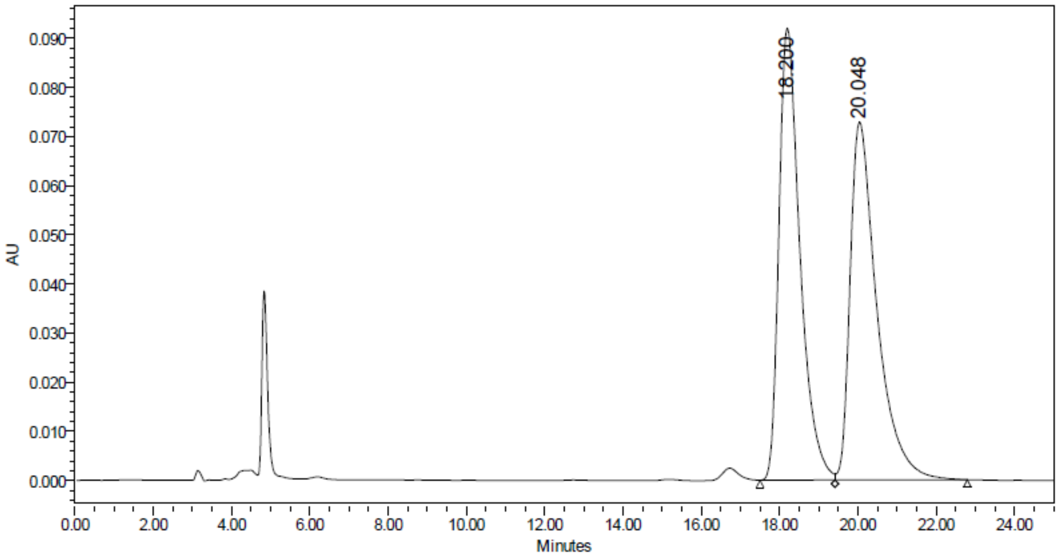

	RT	Area	% Area	Height
1	18.200	3441331	49.88	92019
2	20.048	3458458	50.12	72820

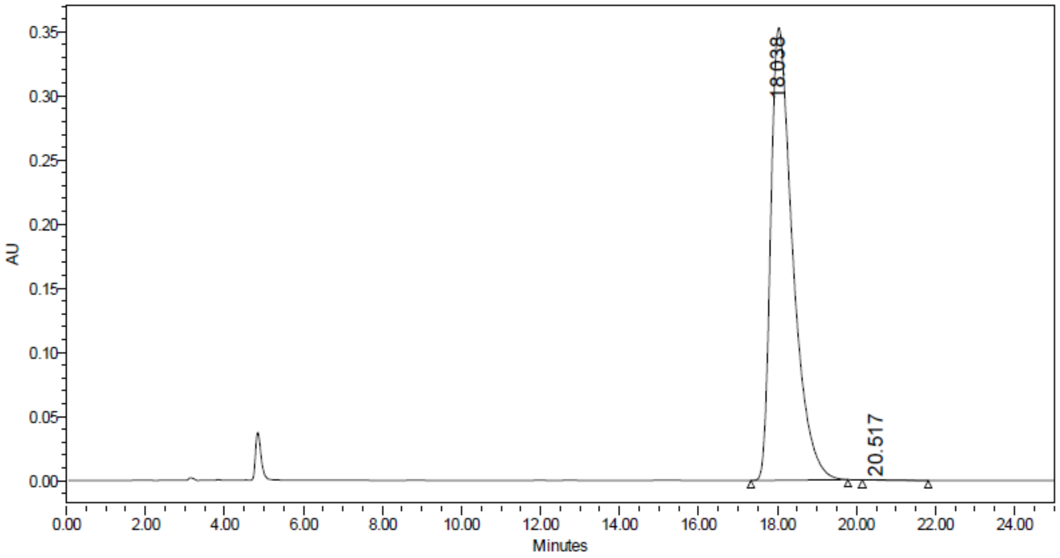

	RT	Area	% Area	Height
1	18.038	13644405	99.96	352718
2	20.517	5320	0.04	134

^{1}H NMR spectra of 3ap

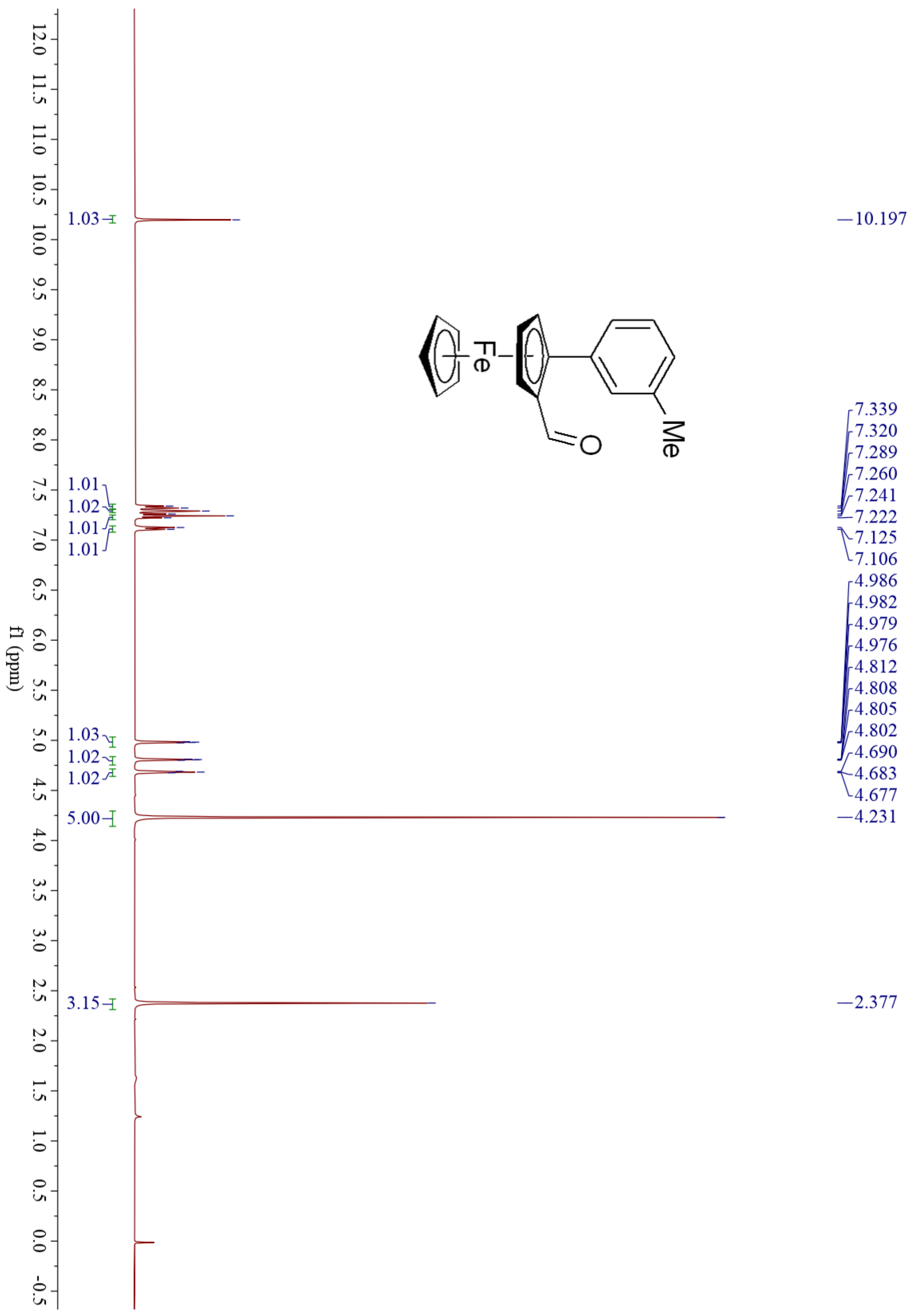

^{13}C NMR spectra of 3ap

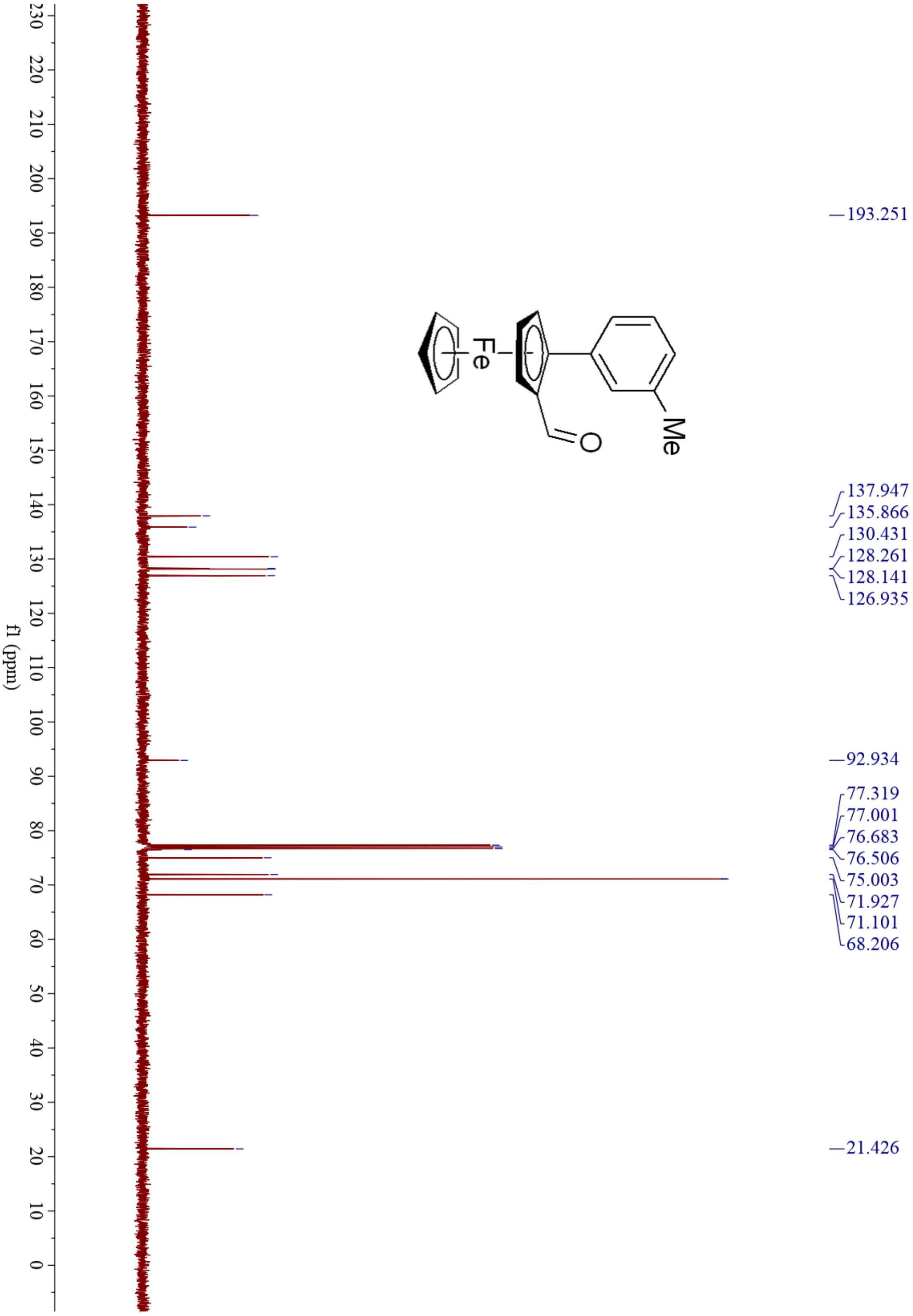

HPLC analysis of 3ap

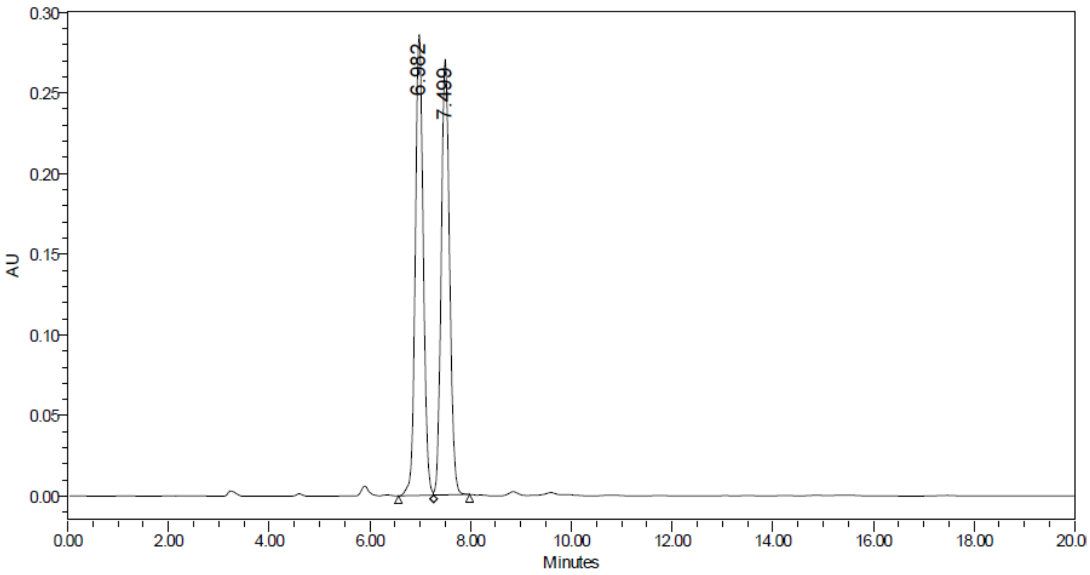

	RT	Area	% Area	Height
1	6.982	3157684	50.38	285890
2	7.499	3109975	49.62	270171

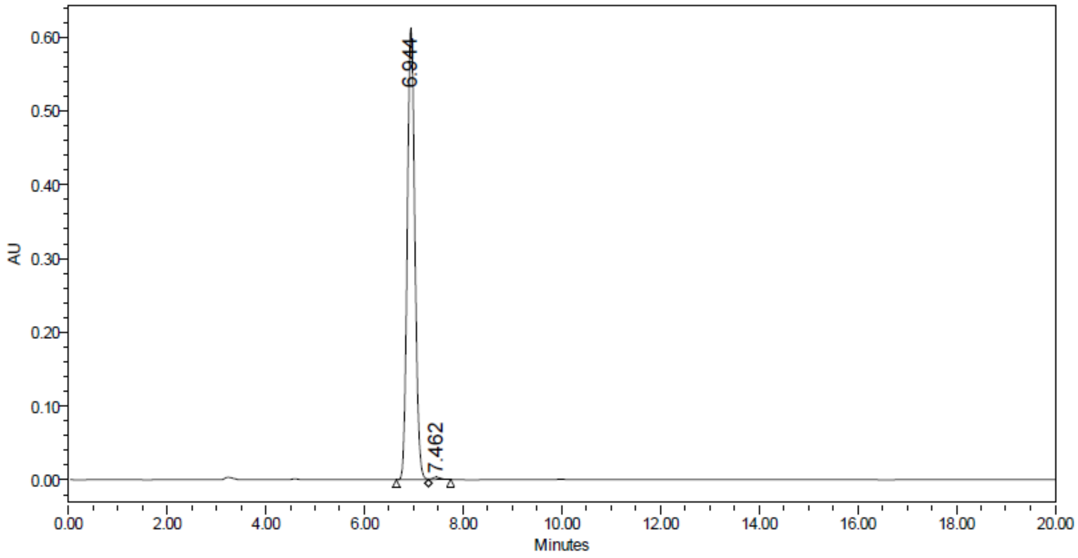

	RT	Area	% Area	Height
1	6.944	6578473	99.46	613711
2	7.462	35408	0.54	3110

^{1}H NMR spectra of 3aq

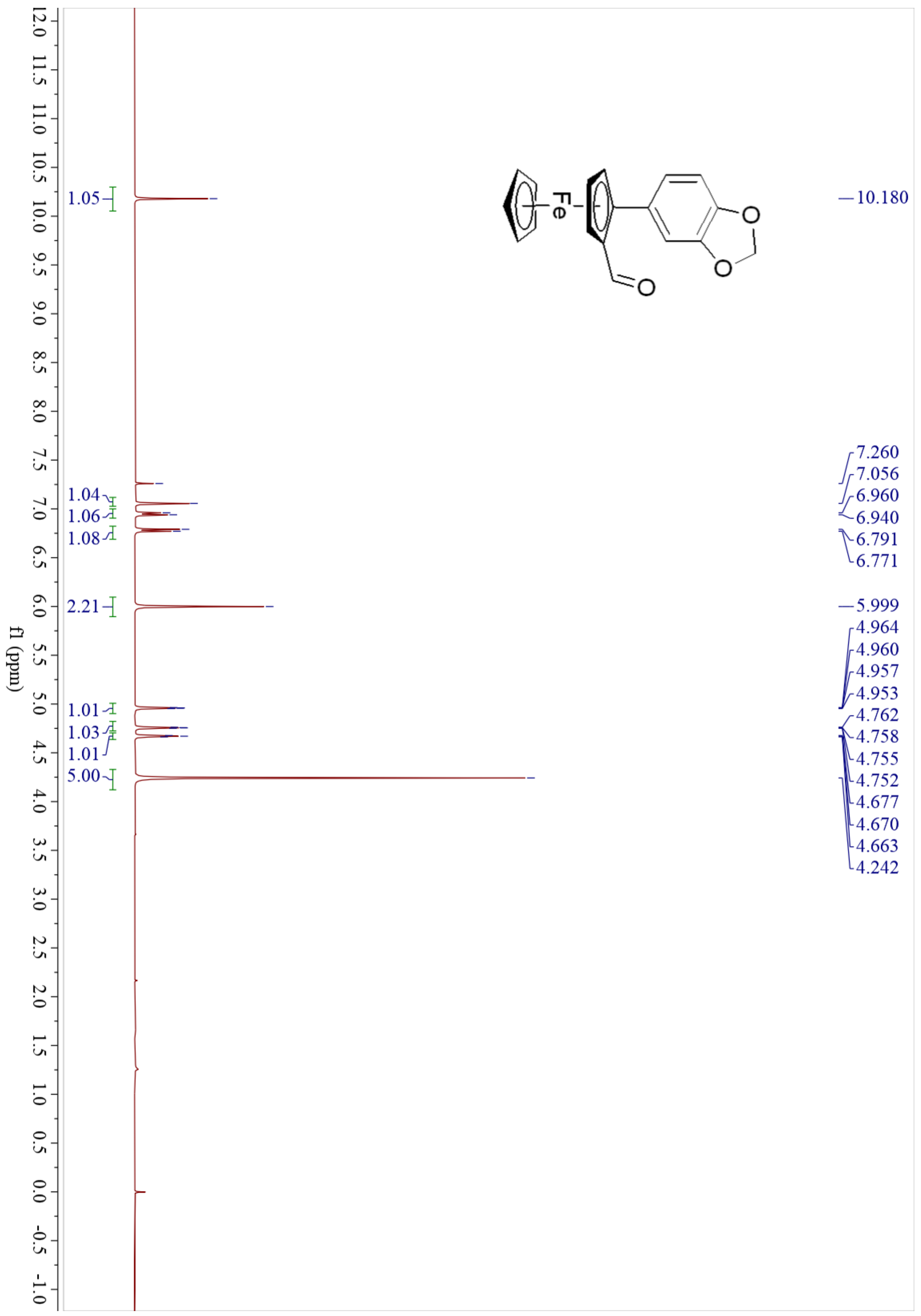

^{13}C NMR spectra of 3aq

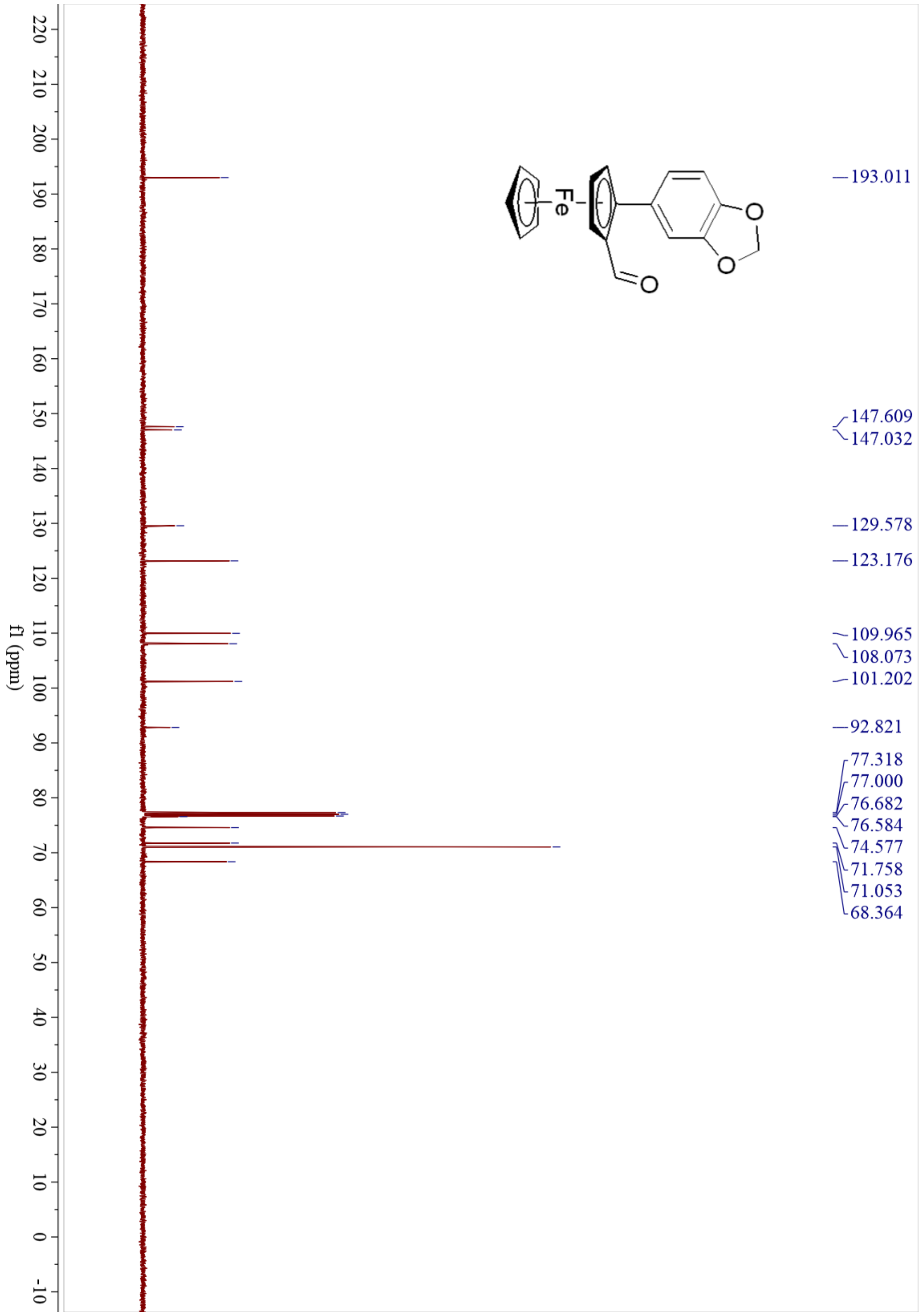

HPLC analysis of 3aq

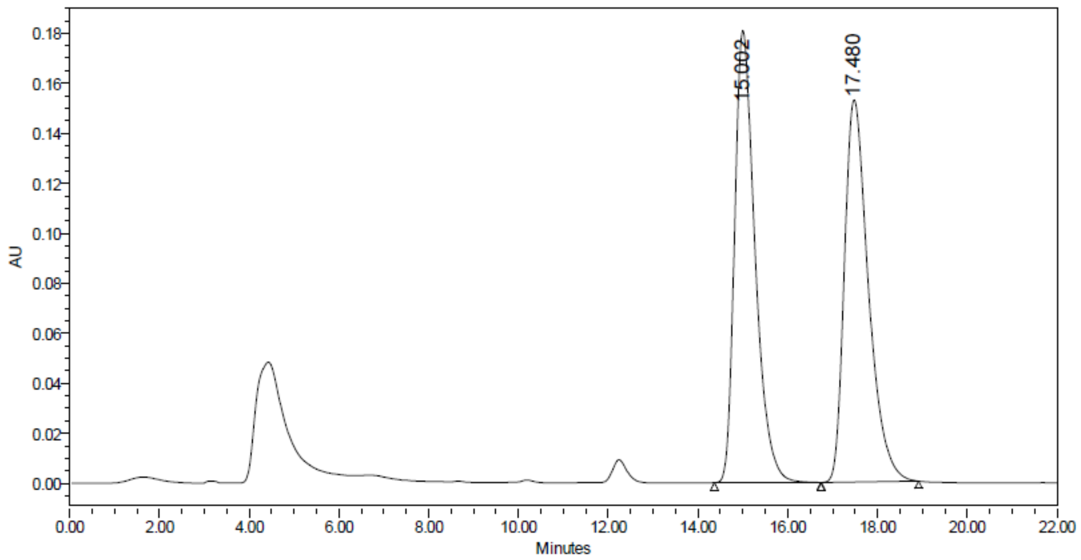

	RT	Area	% Area	Height
1	15.002	5732144	50.24	180808
2	17.480	5677154	49.76	152837

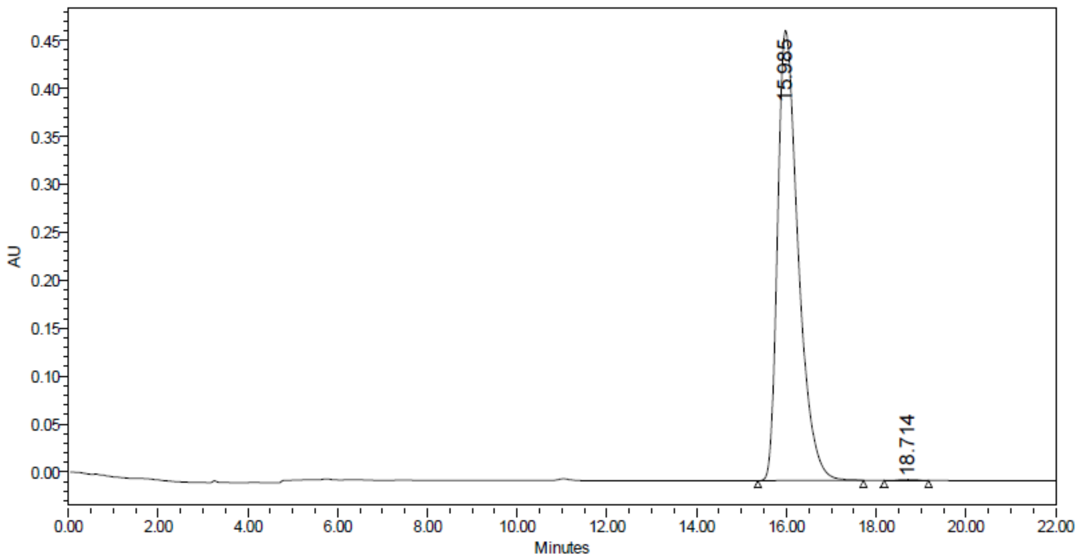

	RT	Area	% Area	Height
1	15.985	14988876	99.87	469110
2	18.714	19894	0.13	733

^{1}H NMR spectra of 3ar

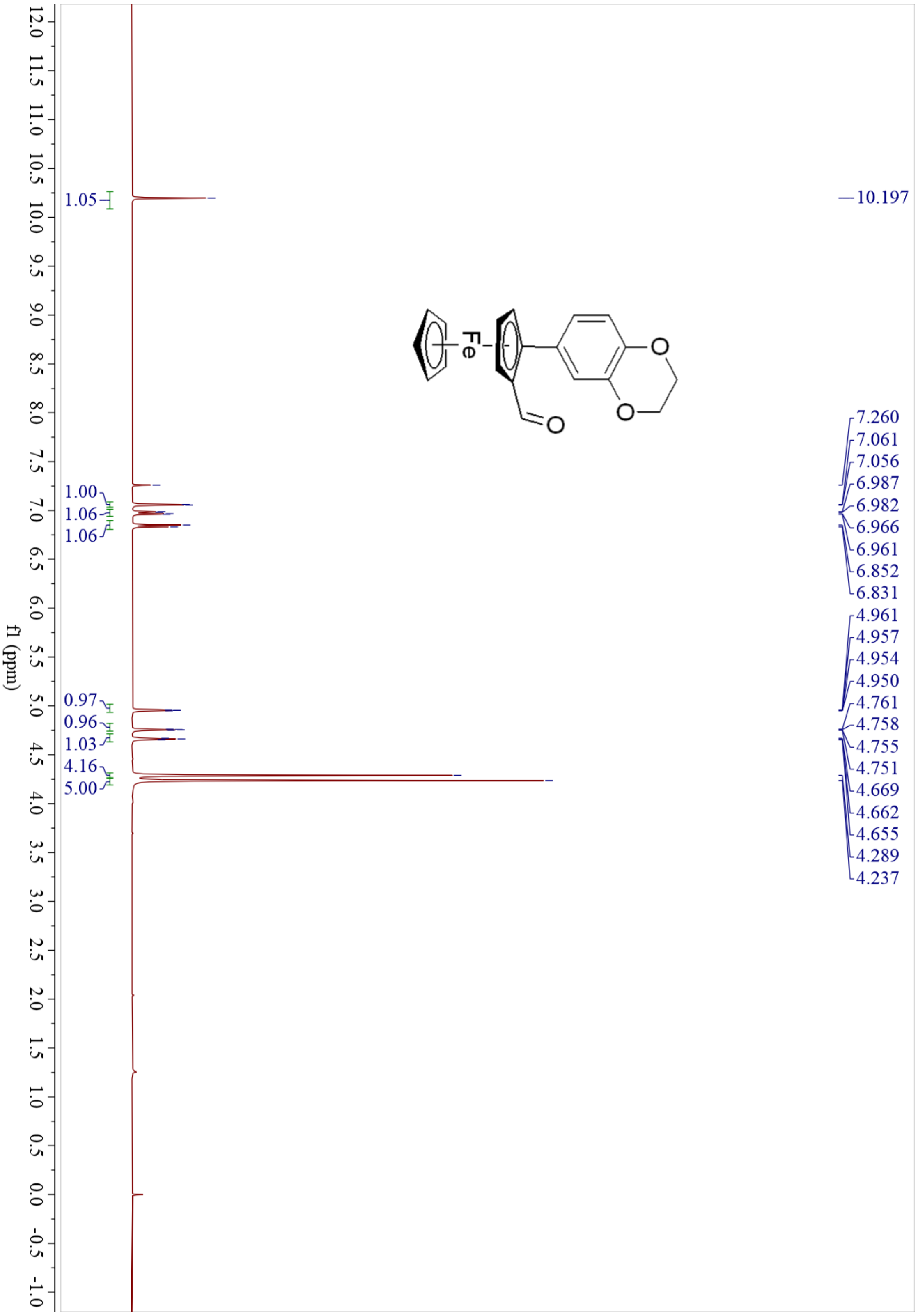

^{13}C NMR spectra of 3ar

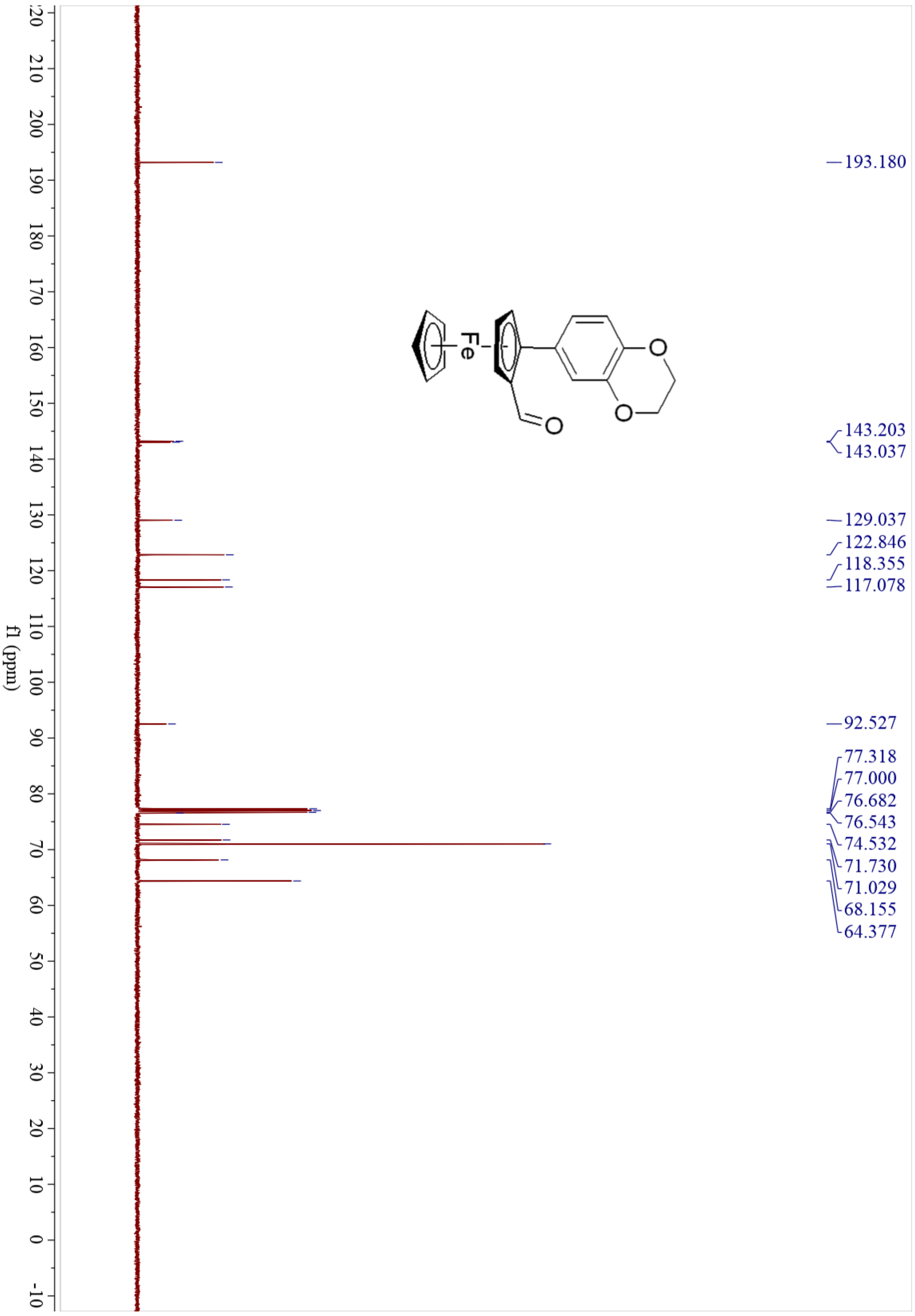

HPLC analysis of 3ar

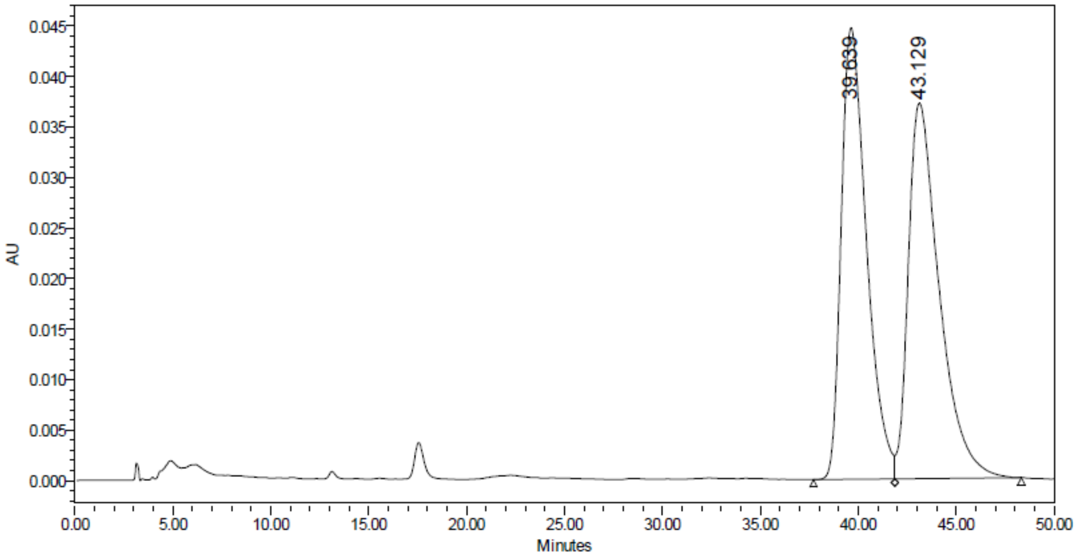

	RT	Area	% Area	Height
1	39.639	4001742	49.46	44688
2	43.129	4088519	50.54	37187

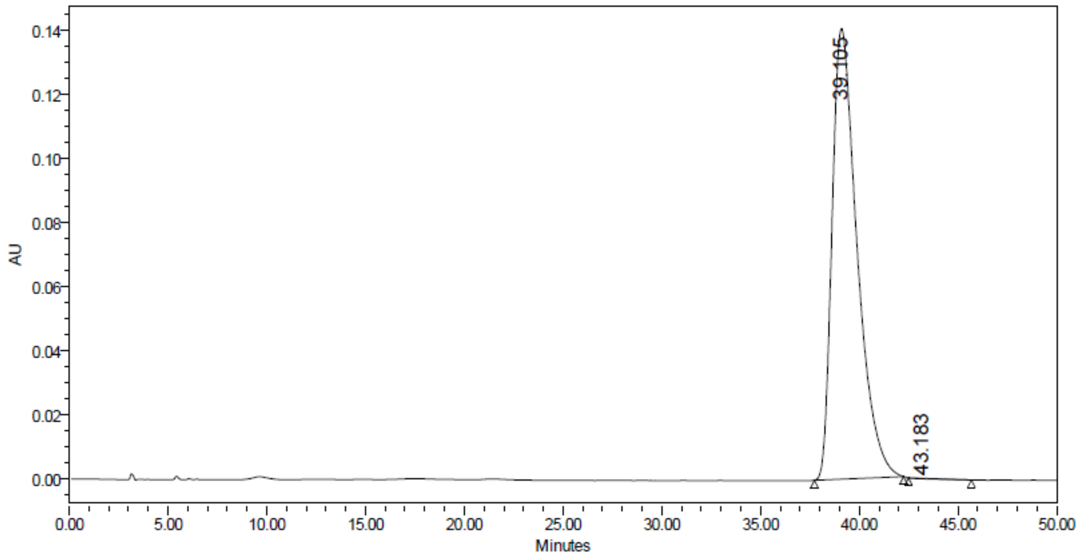

	RT	Area	% Area	Height
1	39.105	12367164	99.89	140562
2	43.183	13957	0.11	-153

^{1}H NMR spectra of 3as

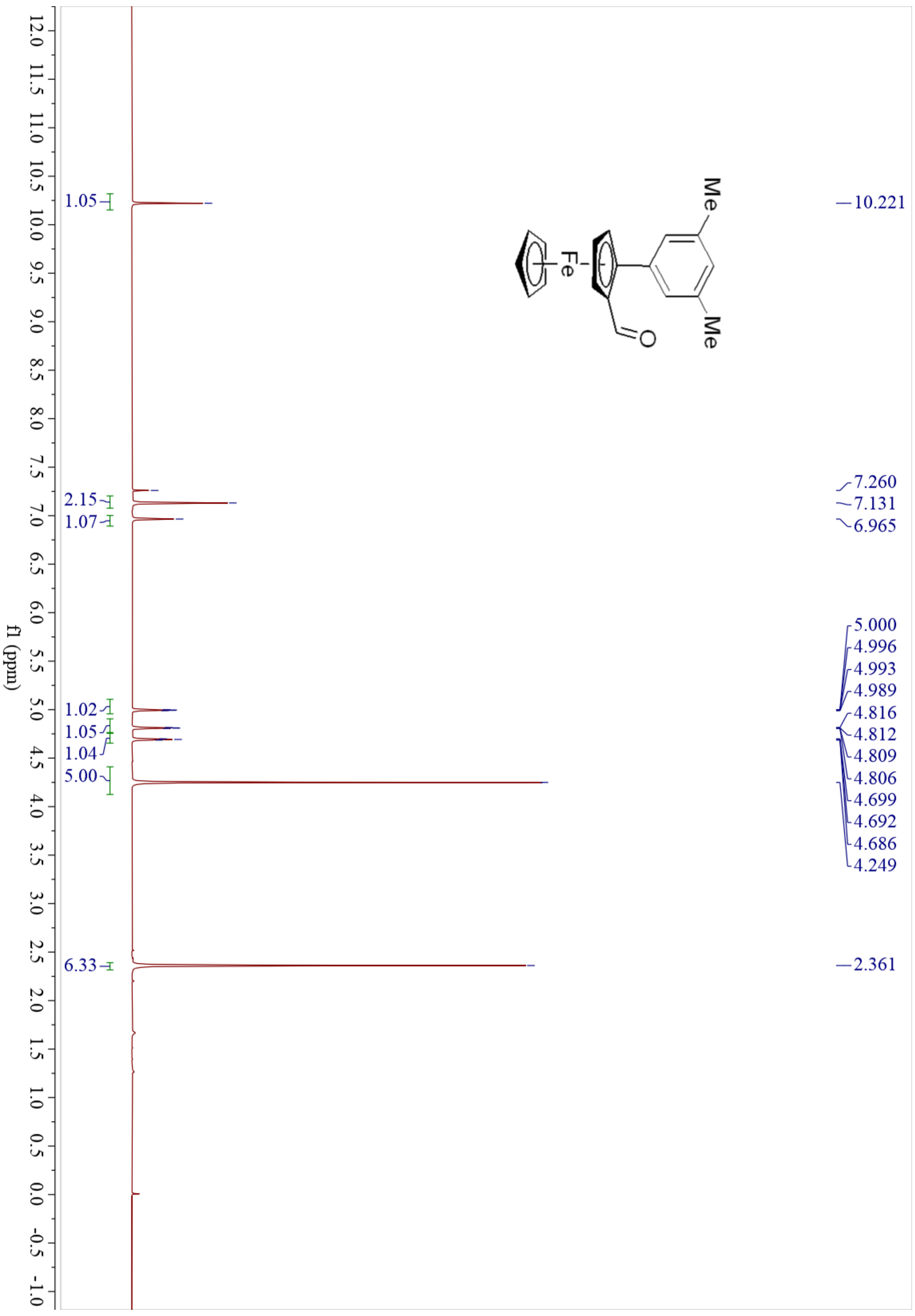

^{13}C NMR spectra of 3as

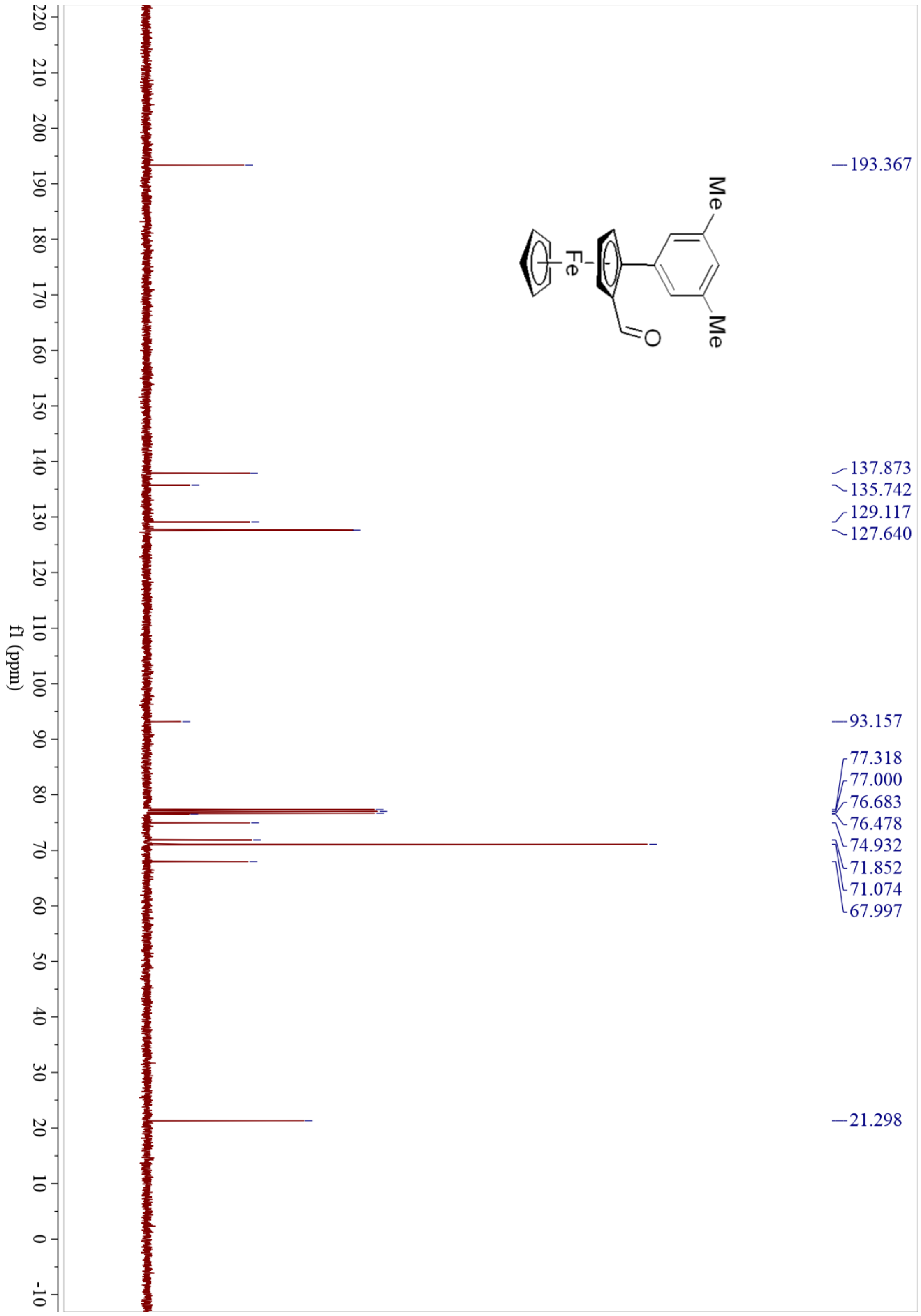

HPLC analysis of 3as

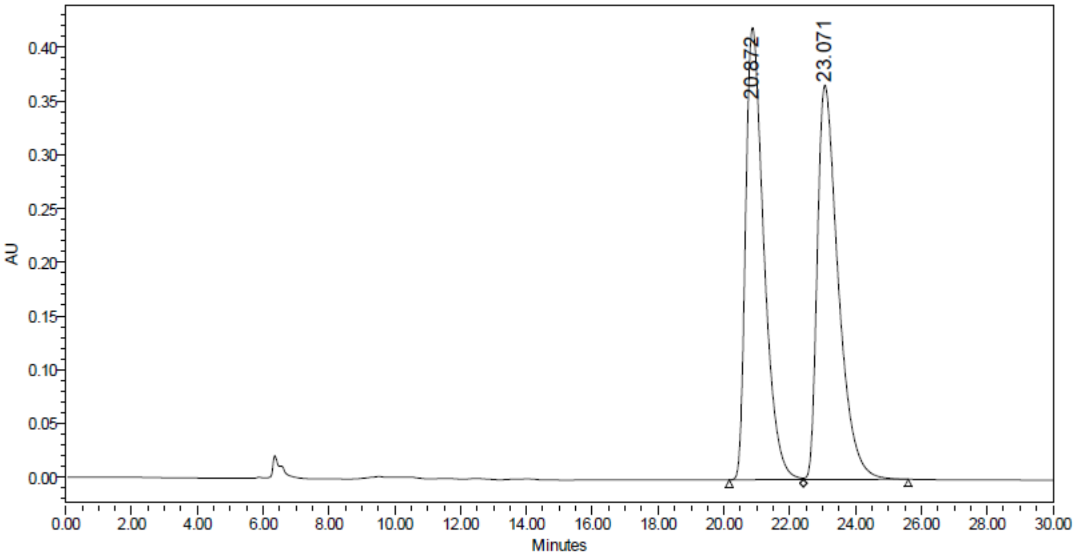

	RT	Area	% Area	Height
1	20.872	16181238	49.98	420362
2	23.071	16196688	50.02	366908

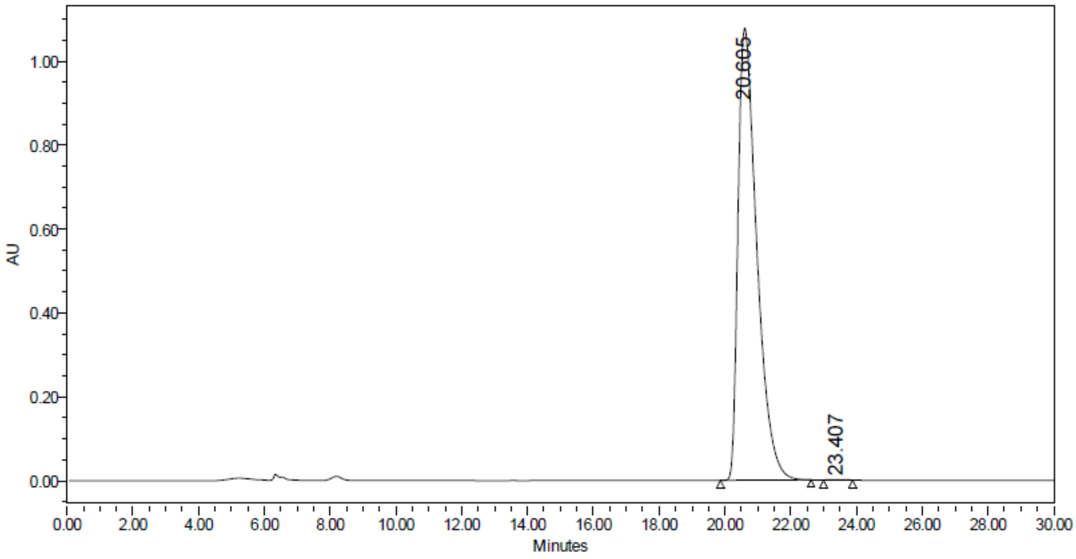

	RT	Area	% Area	Height
1	20.605	42727033	99.92	1077279
2	23.407	32432	0.08	1051

^{1}H NMR spectra of 3at

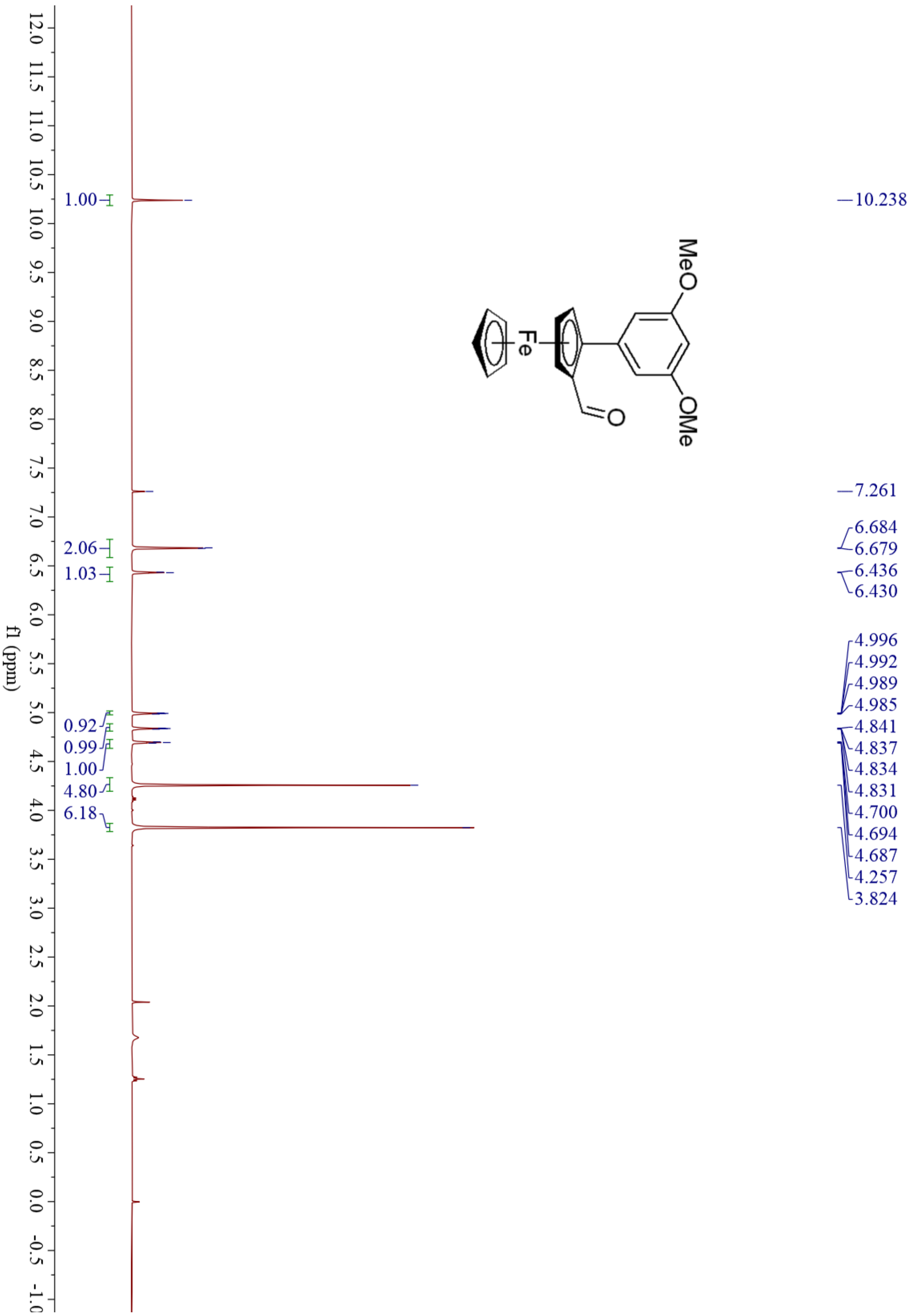

^{13}C NMR spectra of 3at

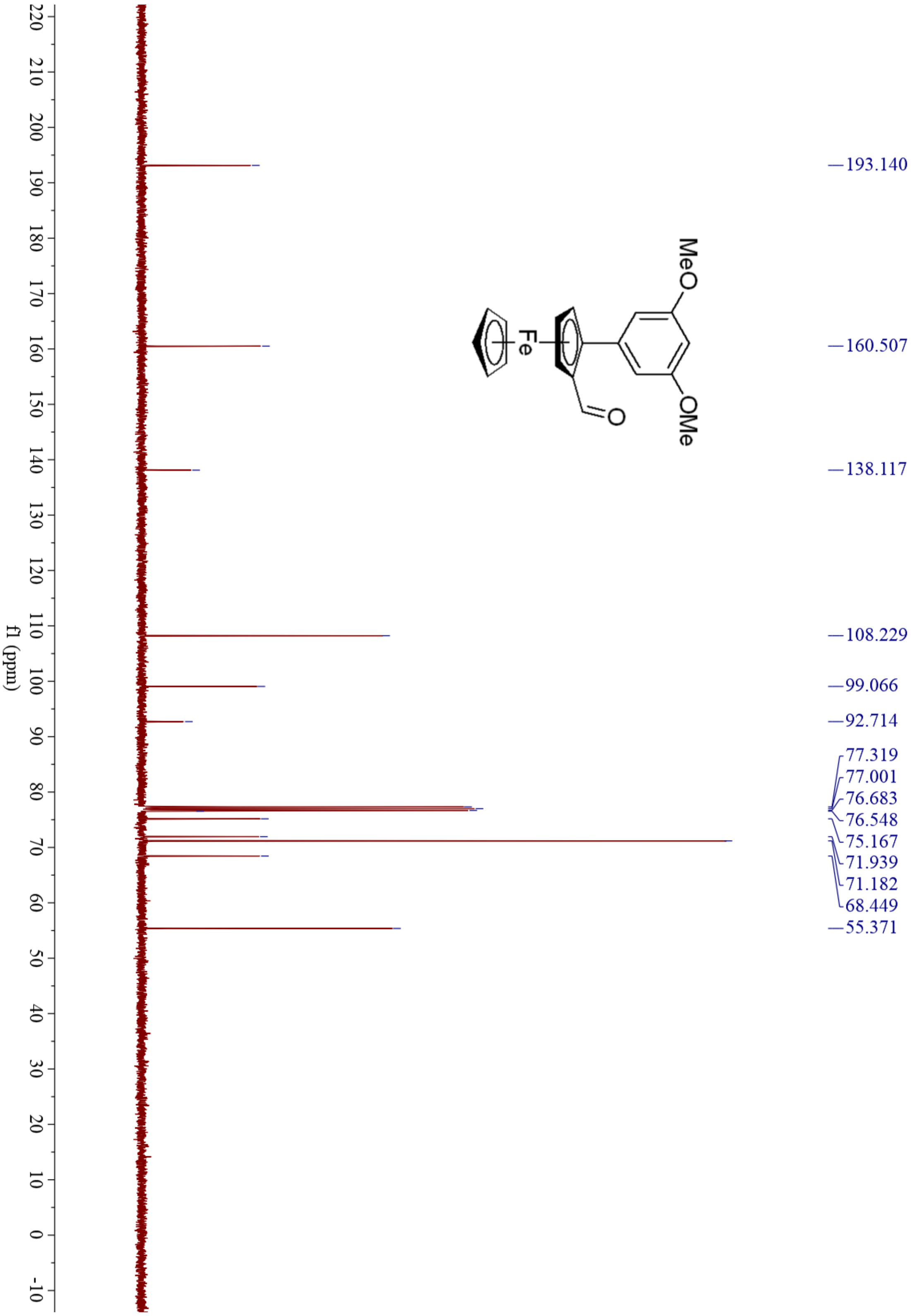

HPLC analysis of 3at

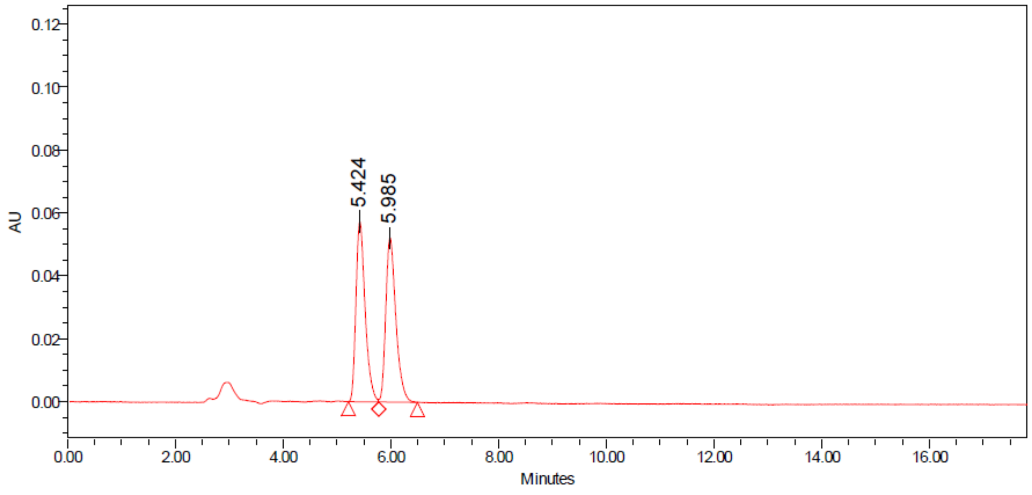

Peak Results

	SampleName	RT	Width (sec)	Height	Area	% Area
1	lcx-23-60-rac	5.424	33.700	57285	681407	50.02
2	lcx-23-60-rac	5.985	43.200	52210	680755	49.98

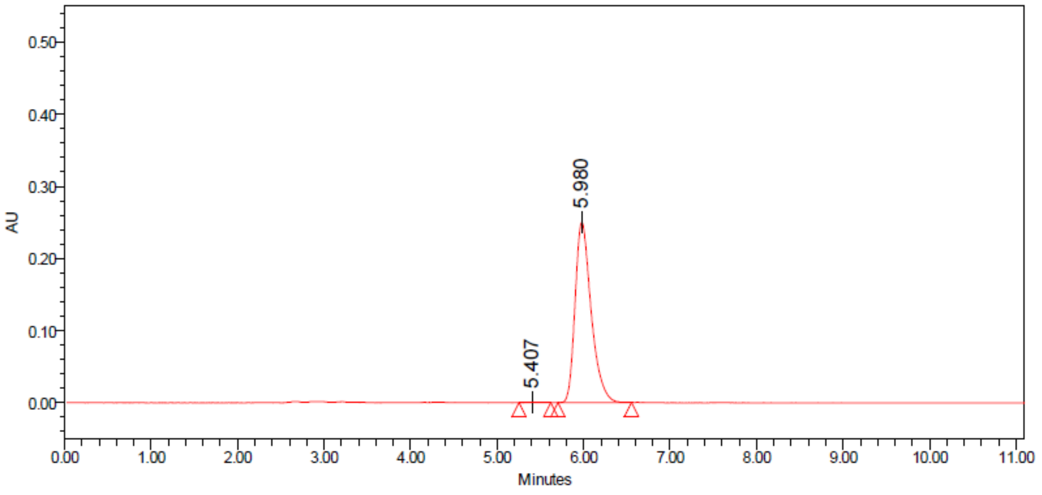

Peak Results

	SampleName	RT	Width (sec)	Height	Area	% Area
1	lcx-23-60-CHIRAL	5.407	22.000	184	1268	0.04
2	lcx-23-60-CHIRAL	5.980	51.000	250027	3241718	99.96

^{1}H NMR spectra of 3au

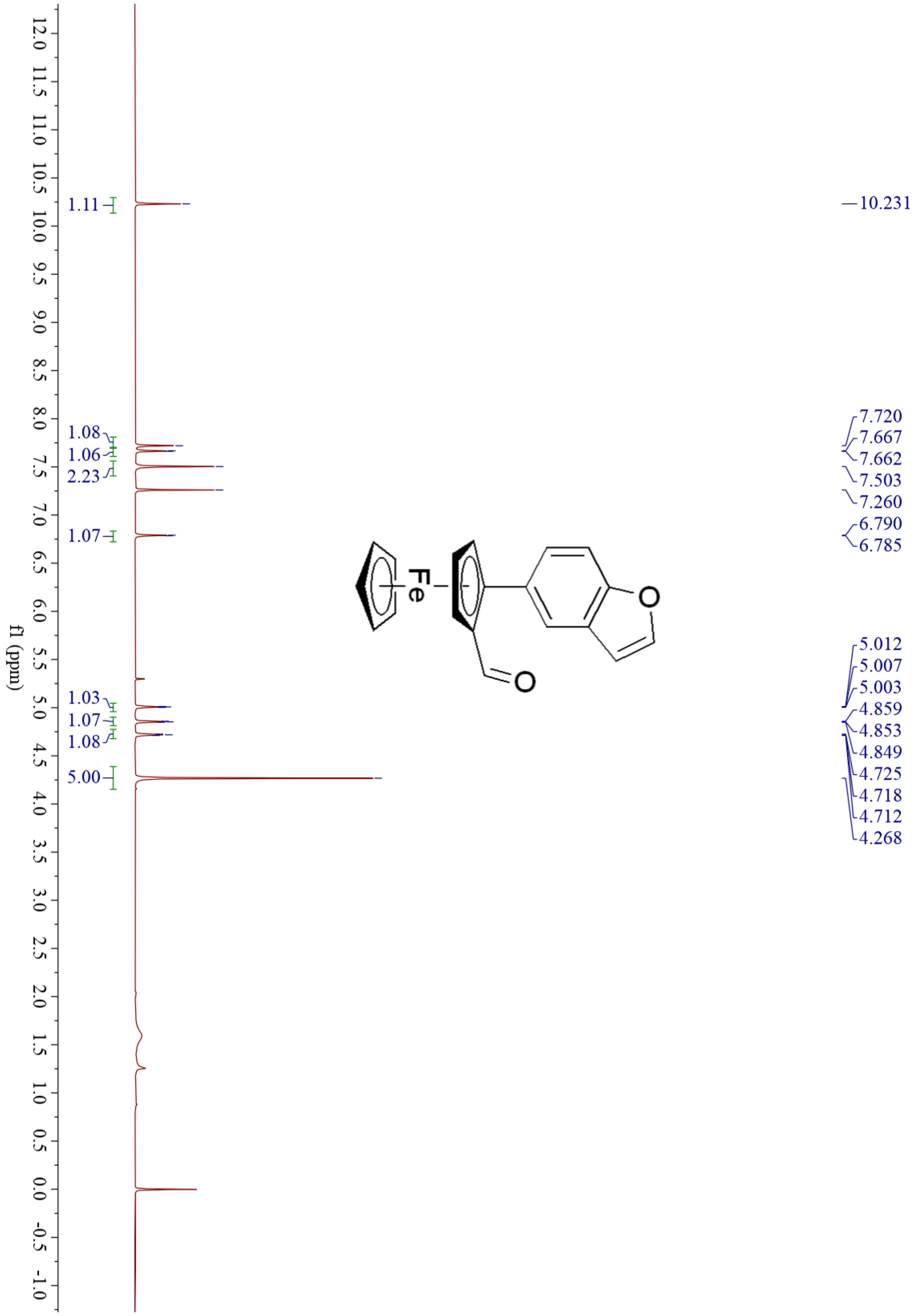

^{13}C NMR spectra of 3au

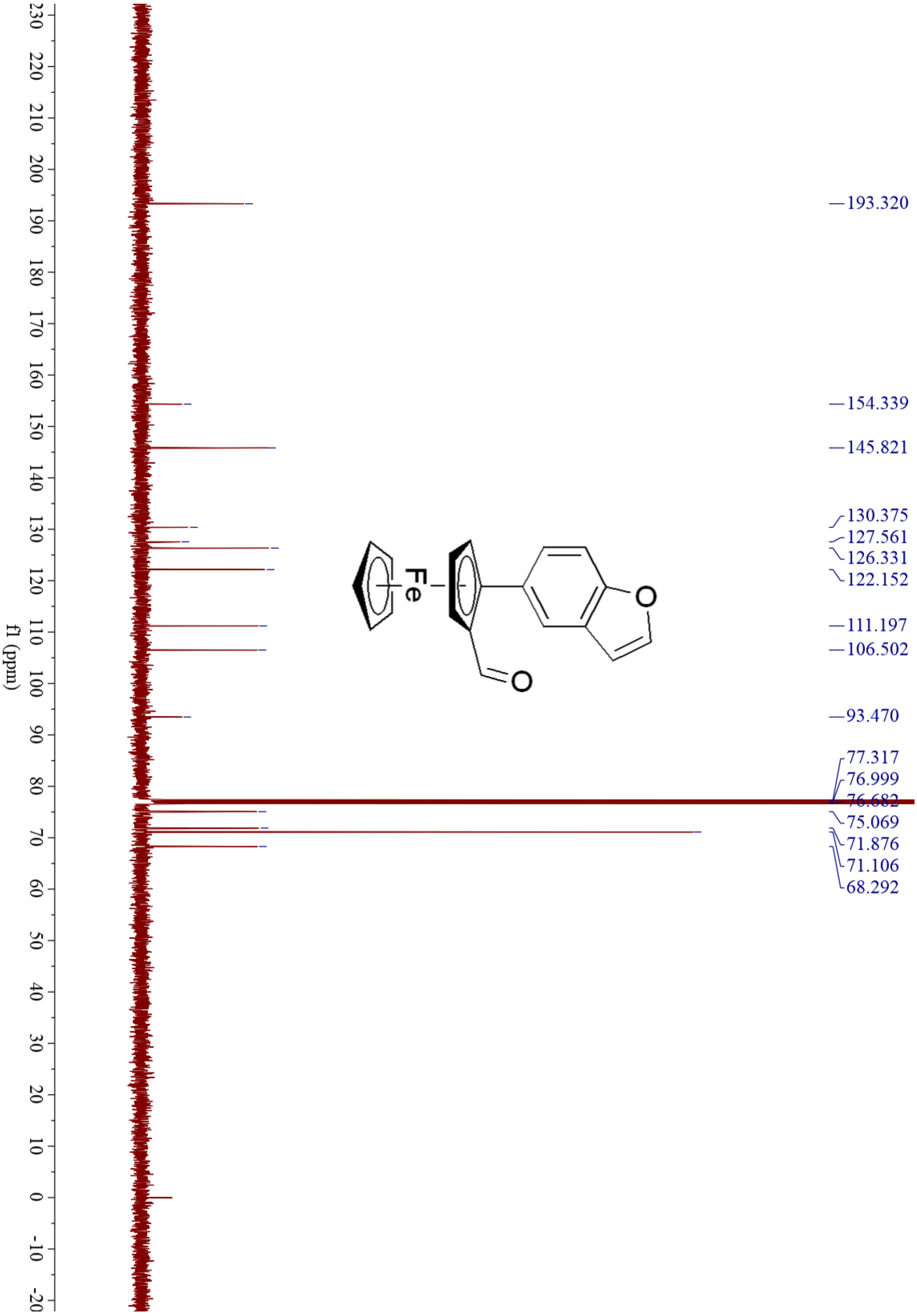

HPLC analysis of 3au

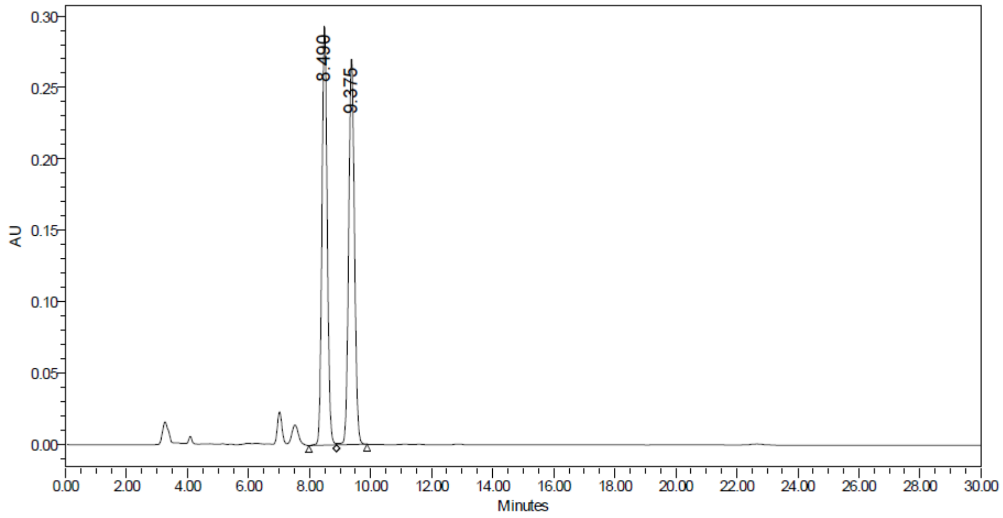

	RT	Area	% Area	Height
1	8.490	3532428	49.82	294242
2	9.375	3557383	50.18	270904

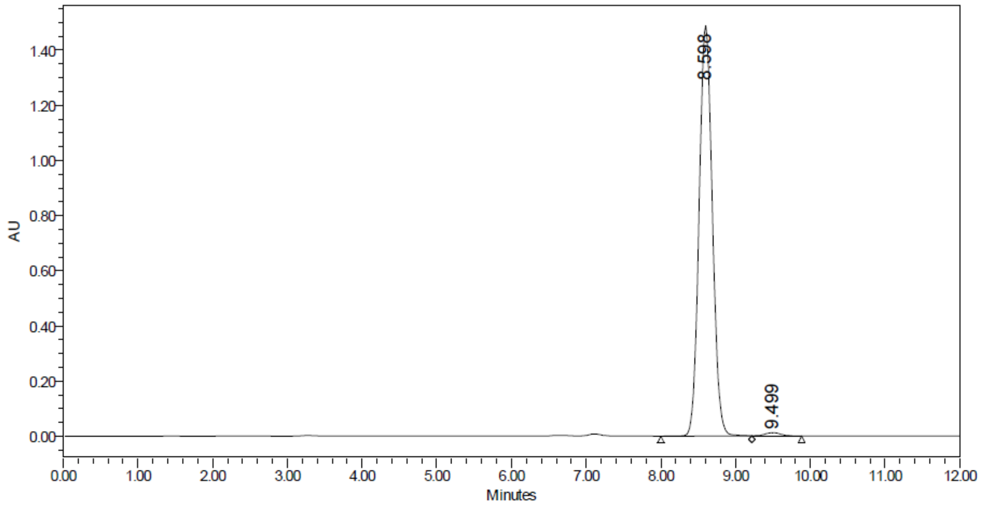

	RT	Area	% Area	Height
1	8.598	18323502	99.05	1486970
2	9.499	175445	0.95	12170

^{1}H NMR spectra of 3av

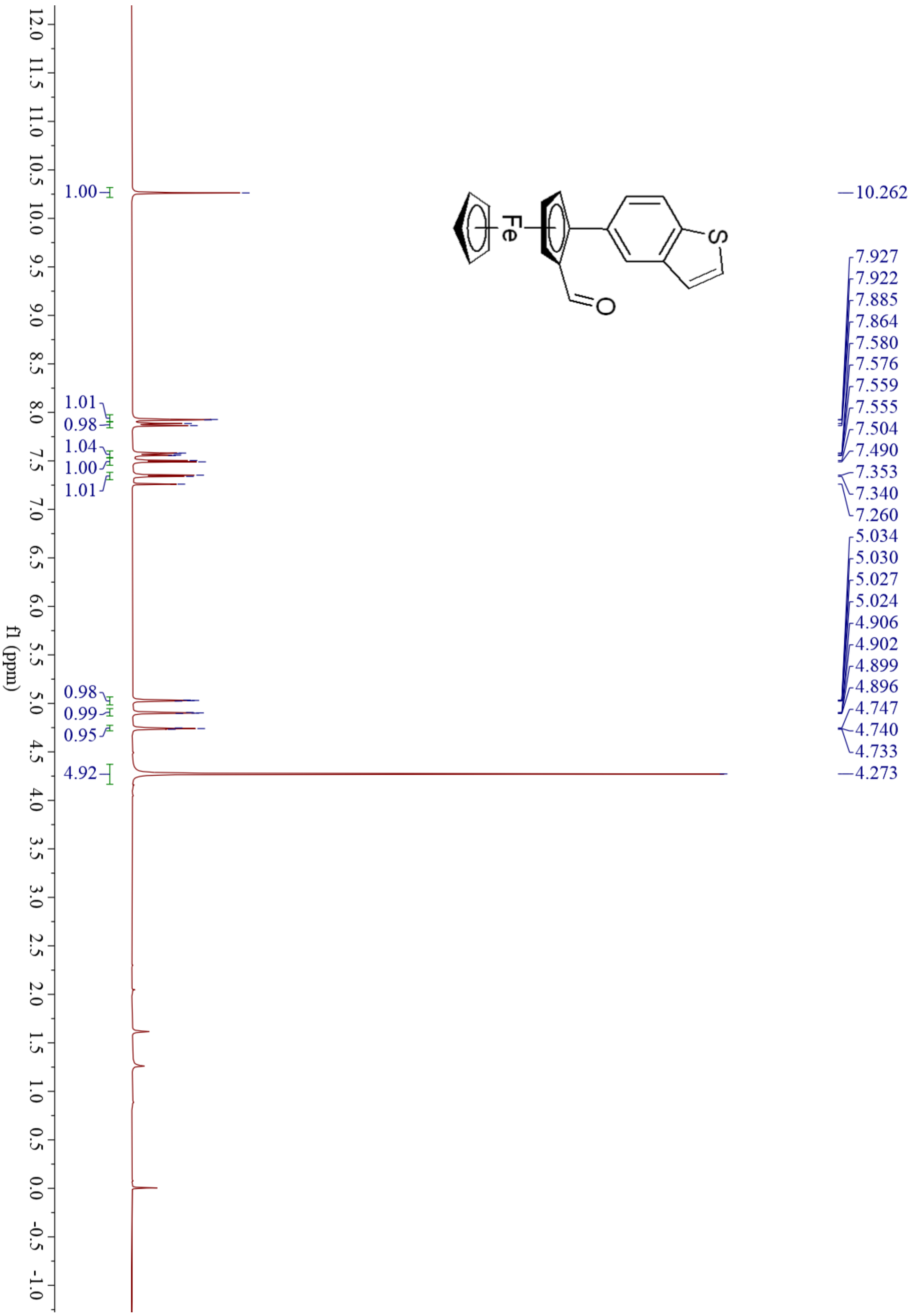

^{13}C NMR spectra of 3av

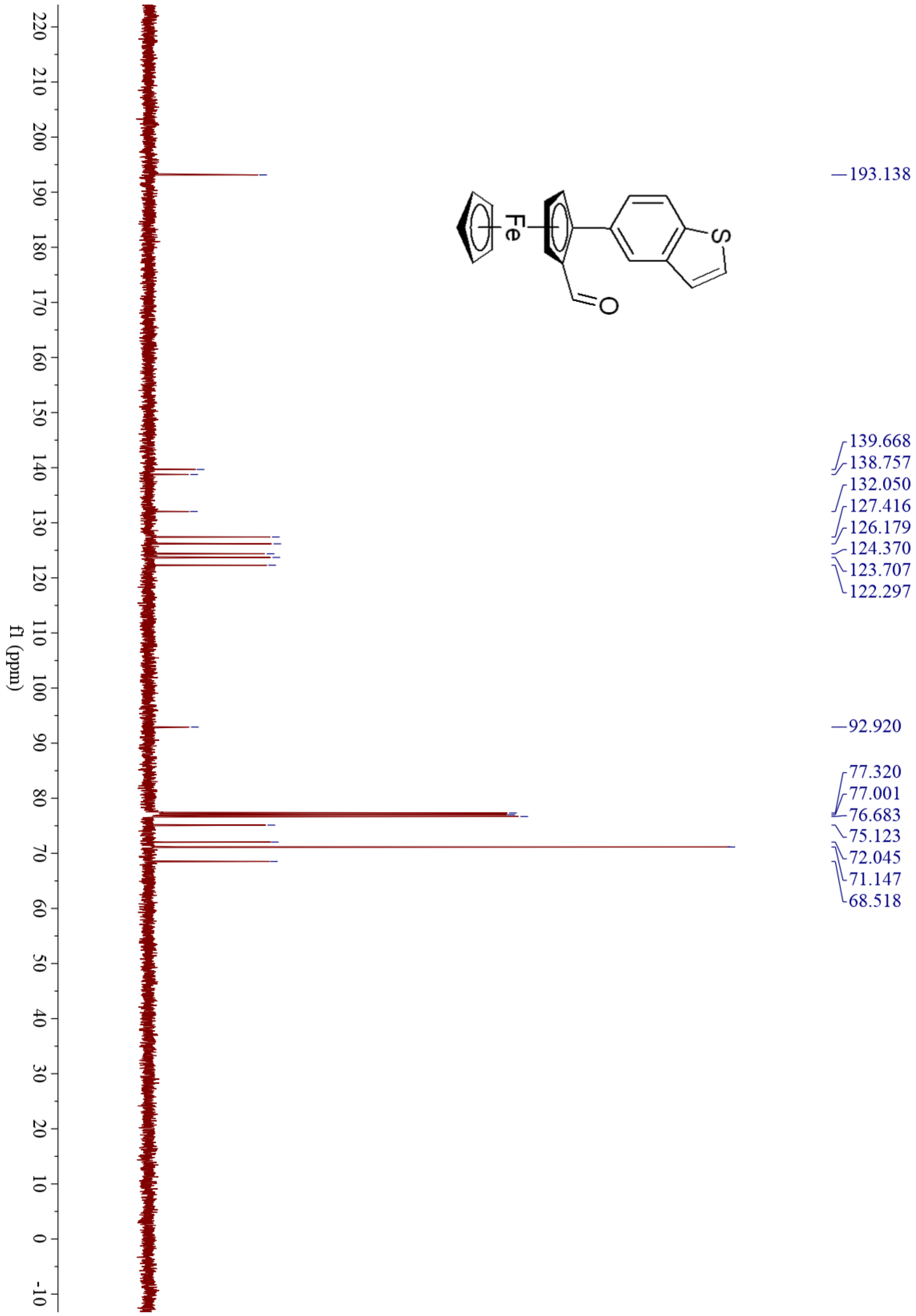

HPLC analysis of 3av

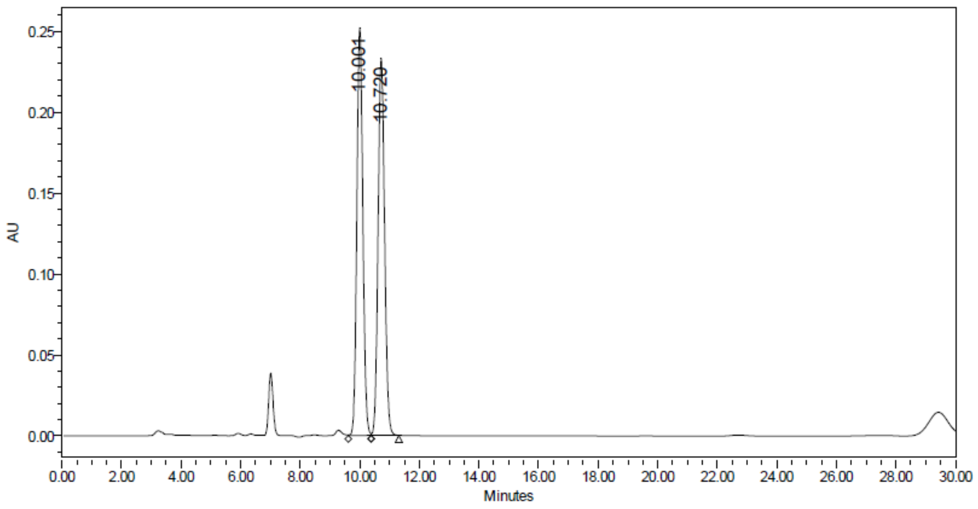

	RT	Area	% Area	Height
1	10.001	3516027	49.94	251753
2	10.720	3525025	50.06	233118

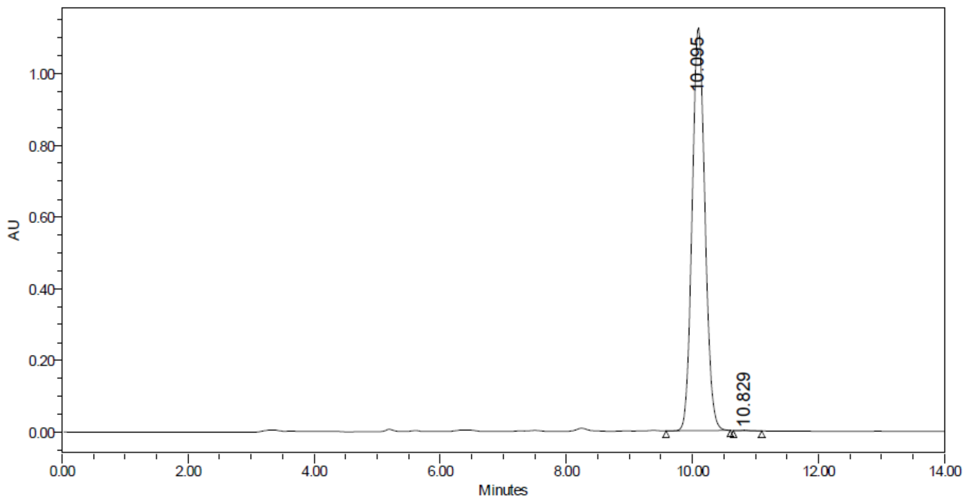

	RT	Area	% Area	Height
1	10.095	15872619	99.94	1125175
2	10.829	9212	0.06	669

^{1}H NMR spectra of 3aw

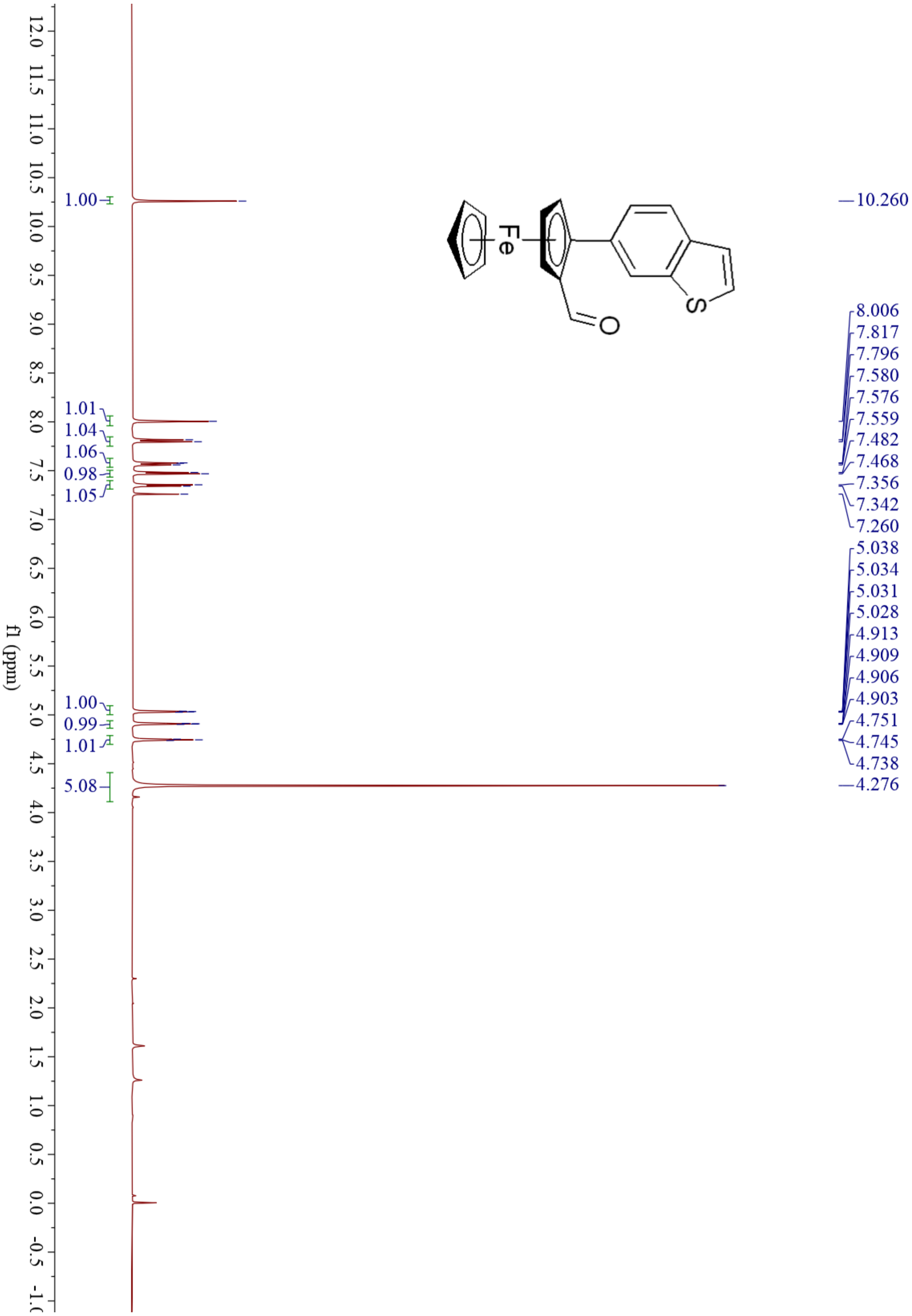

^{13}C NMR spectra of 3aw

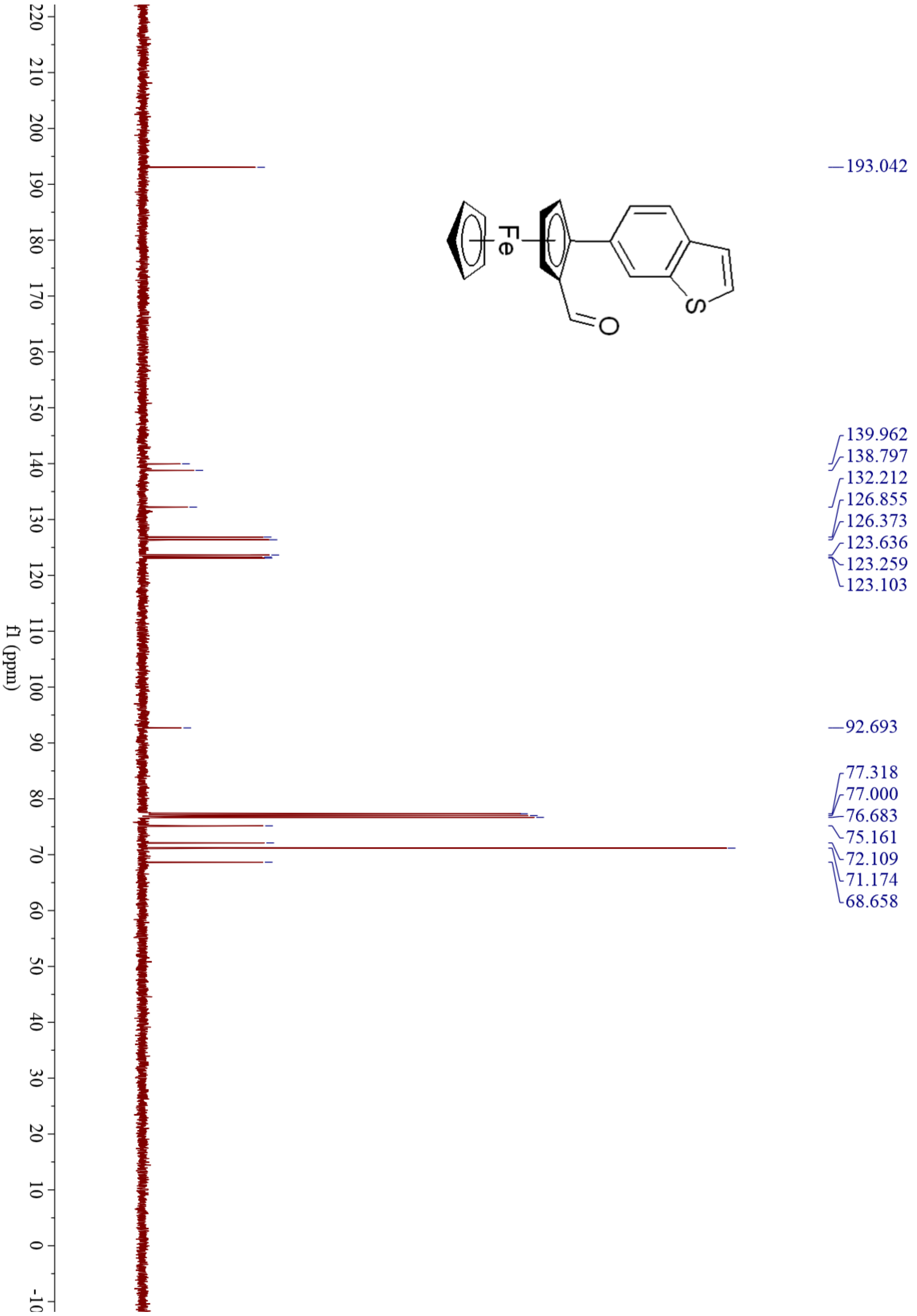

HPLC analysis of 3aw

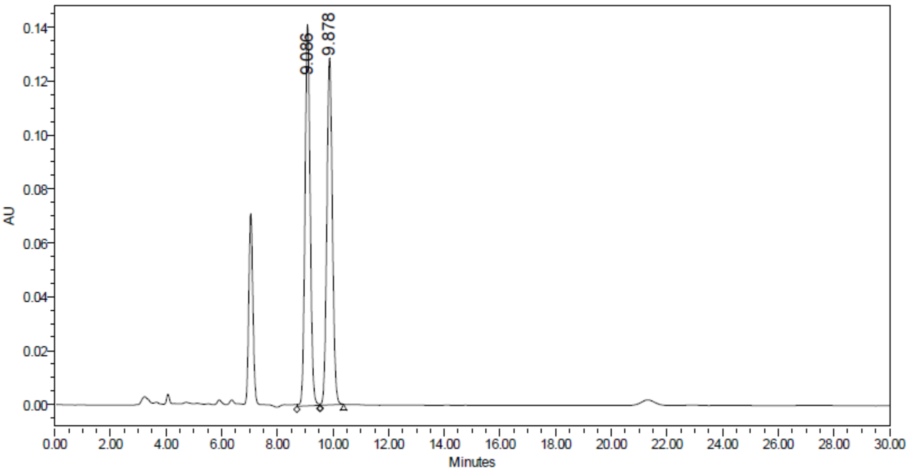

	RT	Area	% Area	Height
1	9.086	1825999	50.40	141526
2	9.878	1797253	49.60	129041

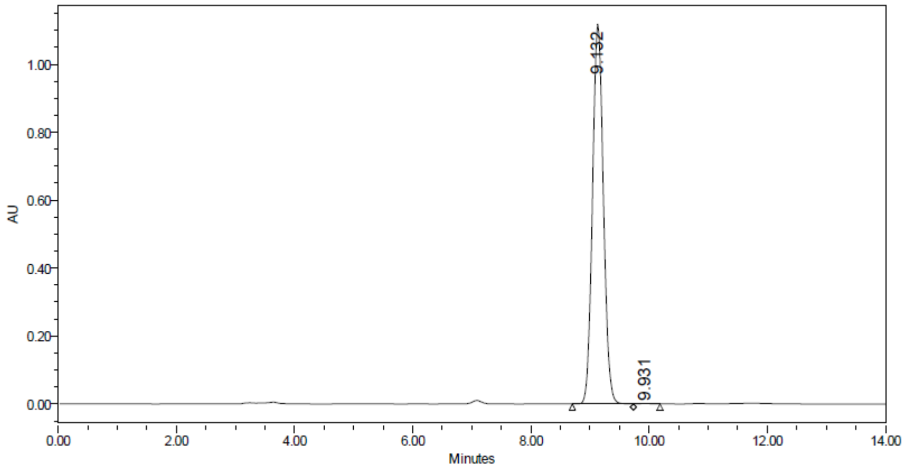

	RT	Area	% Area	Height
1	9.132	14363145	99.89	1117437
2	9.931	16004	0.11	1183

^{1}H NMR spectra of 3ax

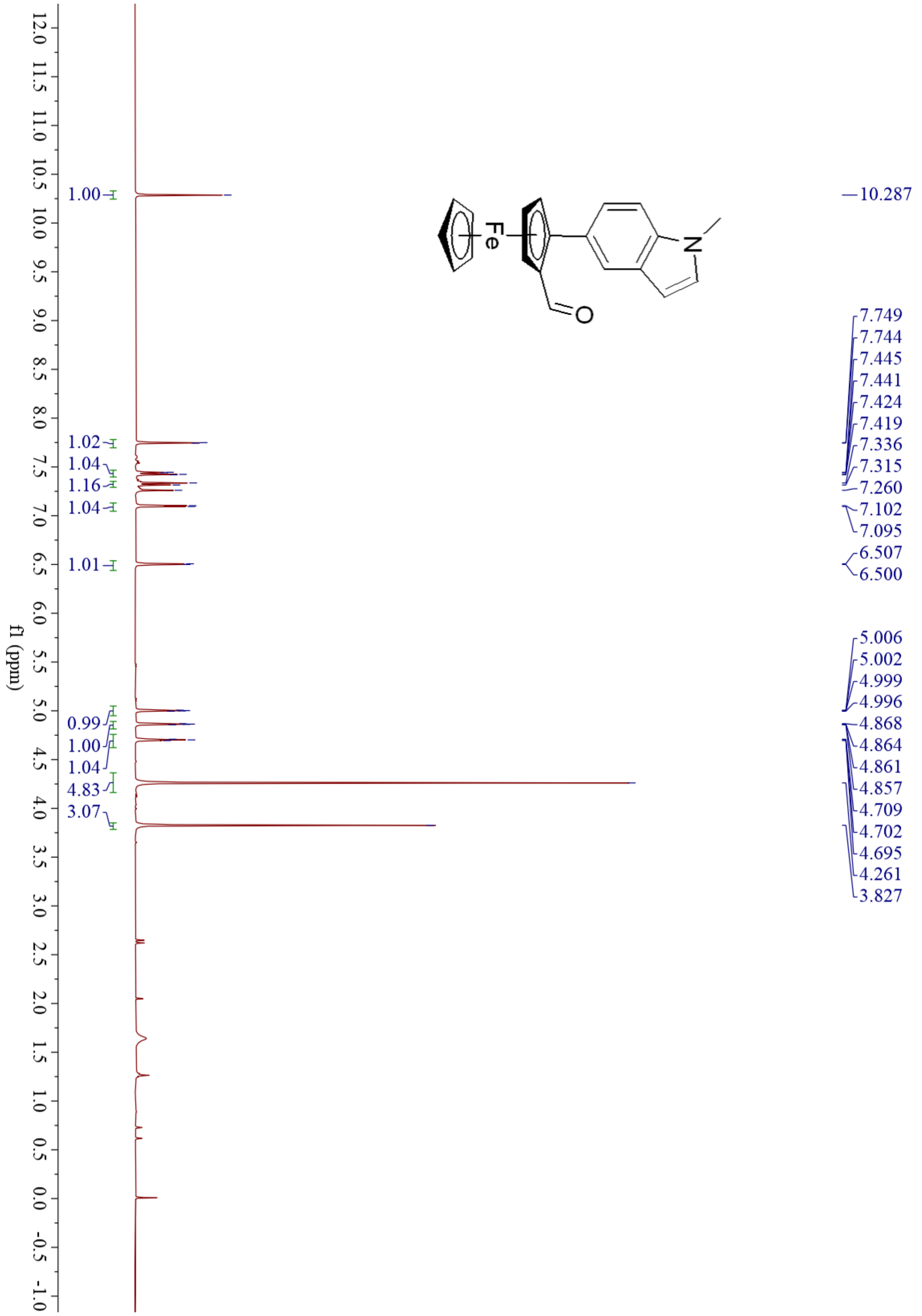

^{13}C NMR spectra of 3ax

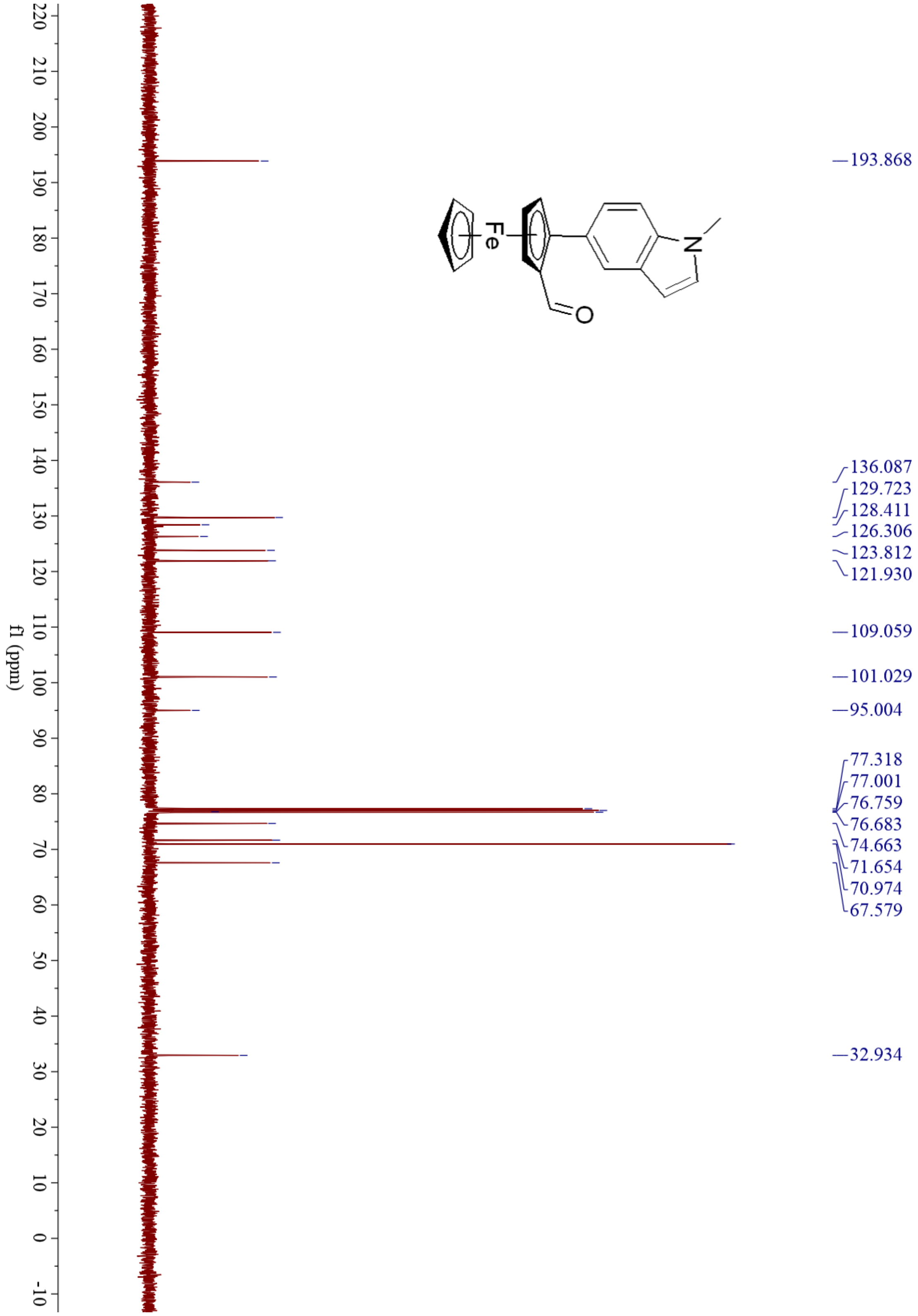

HPLC analysis of 3ax

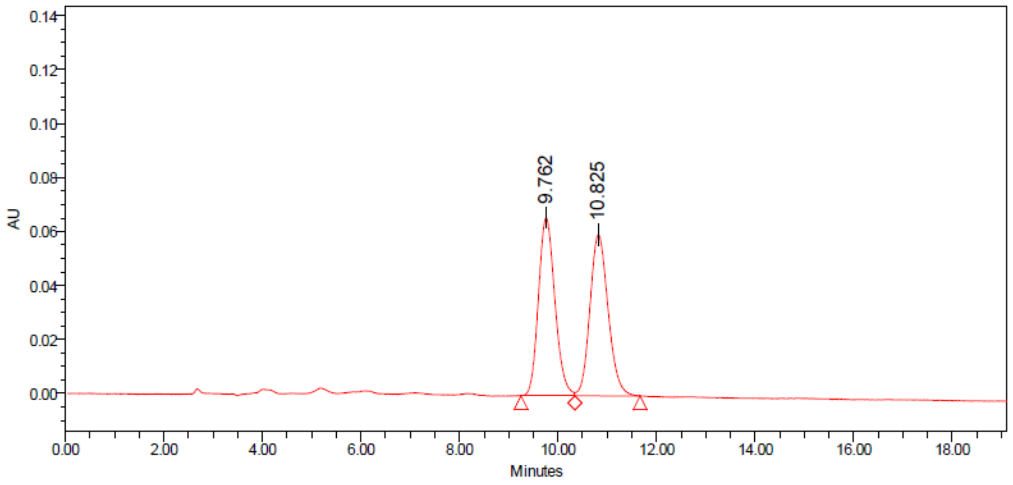

Peak Results

	SampleName	RT	Width (sec)	Height	Area	% Area
1	LCX-23-59-RAC	9.762	65.600	65950	1529343	49.99
2	LCX-23-59-RAC	10.825	79.400	59597	1529728	50.01

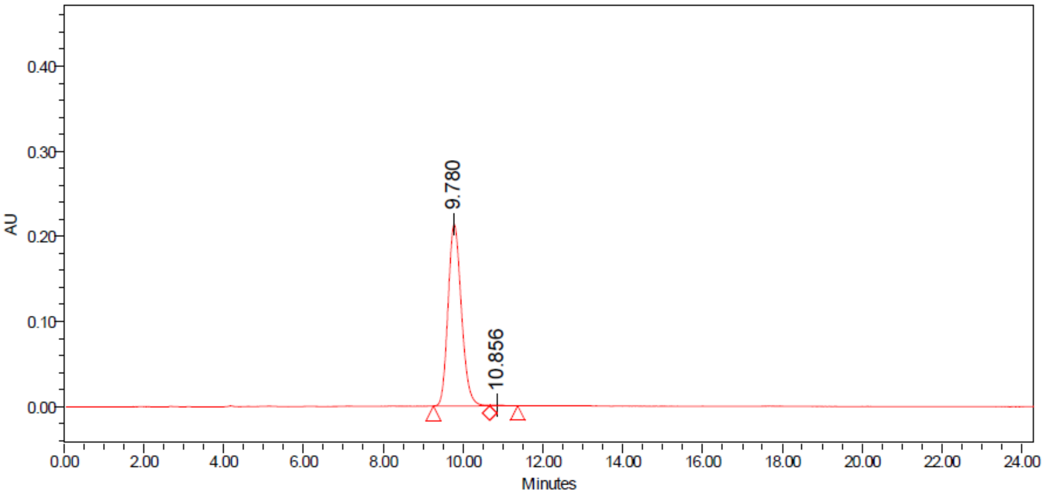

Peak Results

	SampleName	RT	Width (sec)	Height	Area	% Area
1	LCX-23-59-chiral	9.780	84.700	213285	4965578	99.57
2	LCX-23-59-chiral	10.856	42.000	966	21311	0.43

^{1}H NMR spectra of 3ay

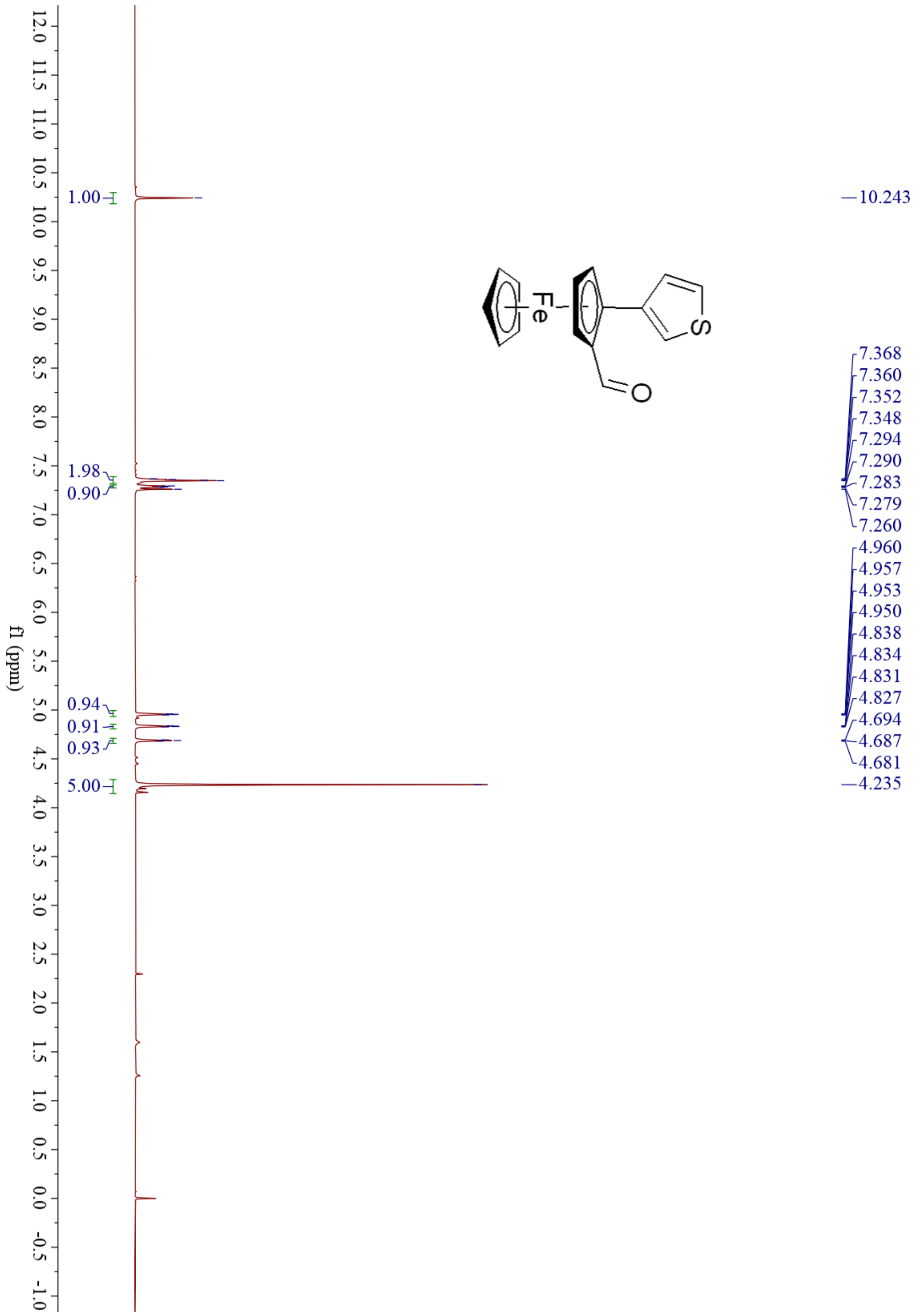

^{13}C NMR spectra of 3ay

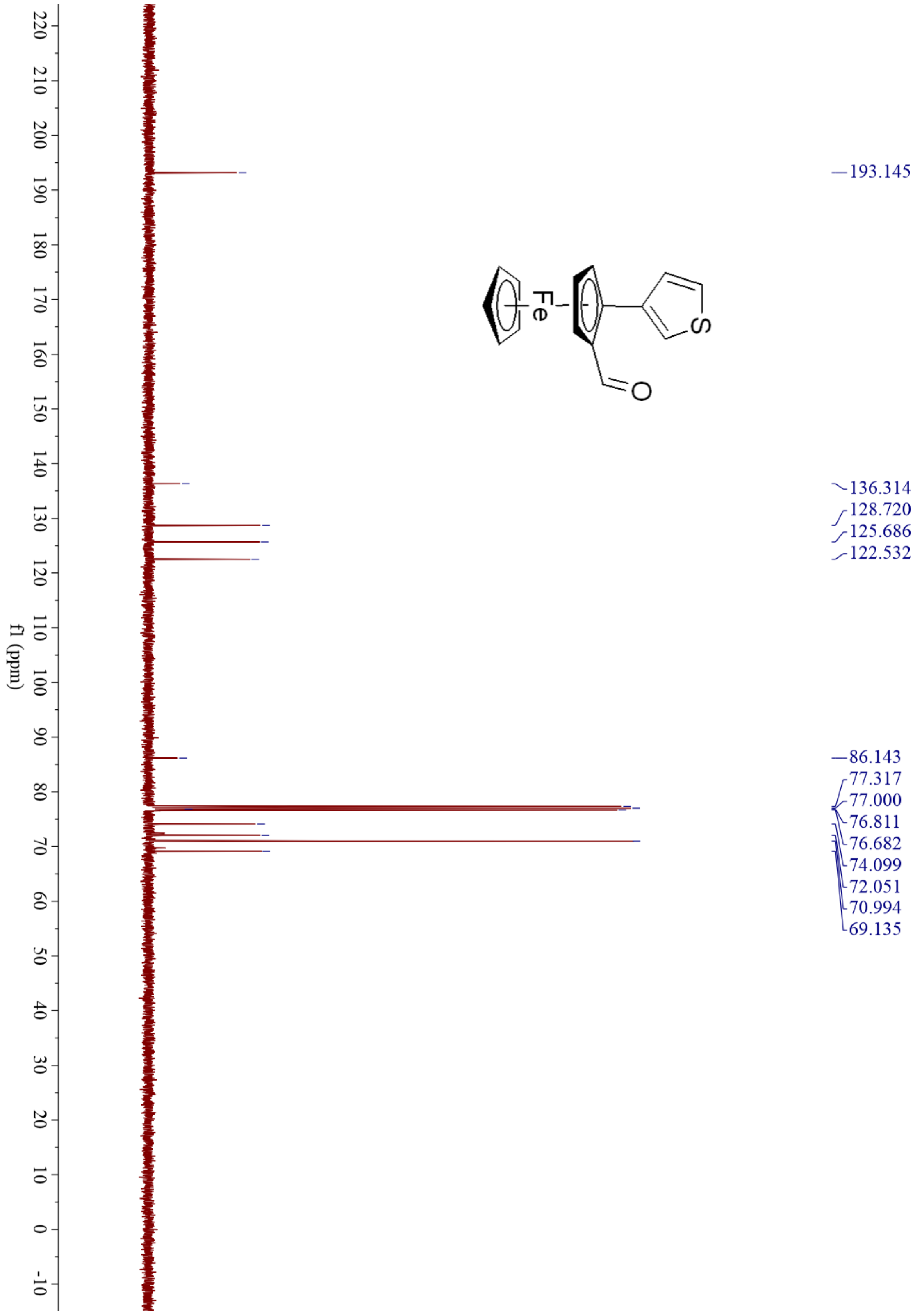

HPLC analysis of 3ay

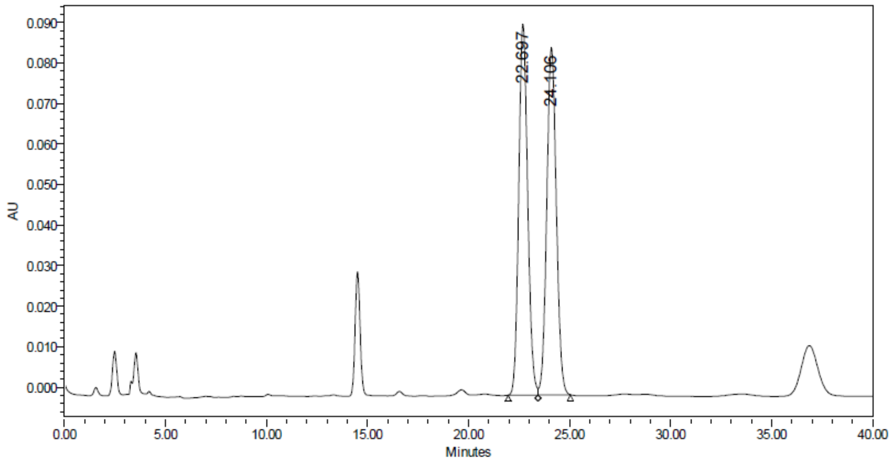

	RT	Area	% Area	Height
1	22.697	2810359	49.94	91497
2	24.106	2816569	50.06	85614

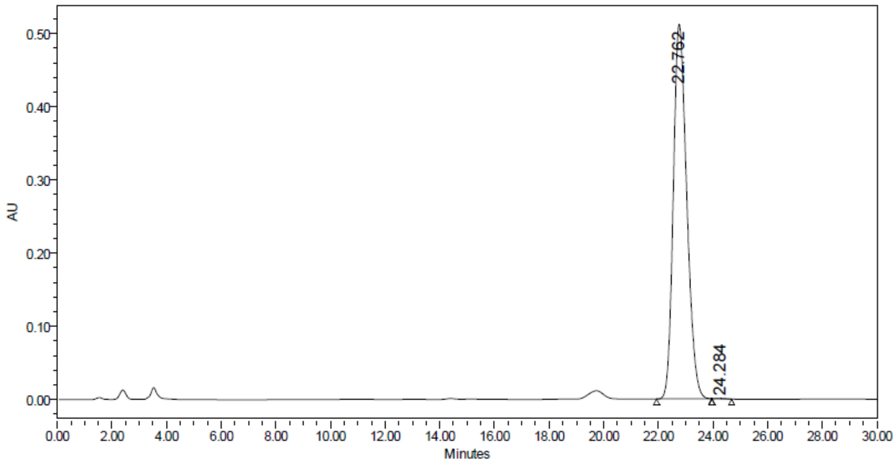

	RT	Area	% Area	Height
1	22.762	17456603	99.91	511870
2	24.284	15178	0.09	609

^{1}H NMR spectra of 3ba

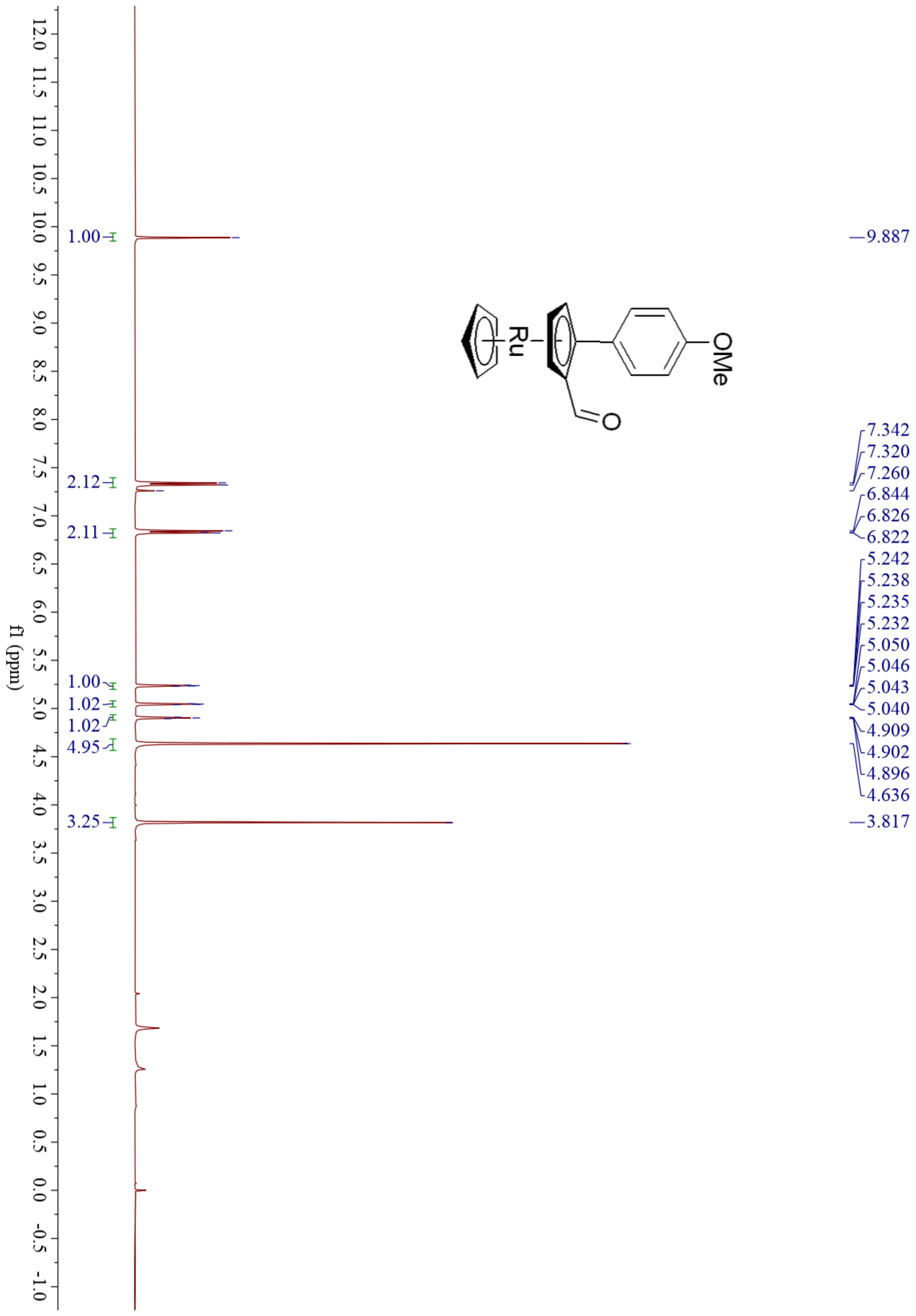

^{13}C NMR spectra of 3ba

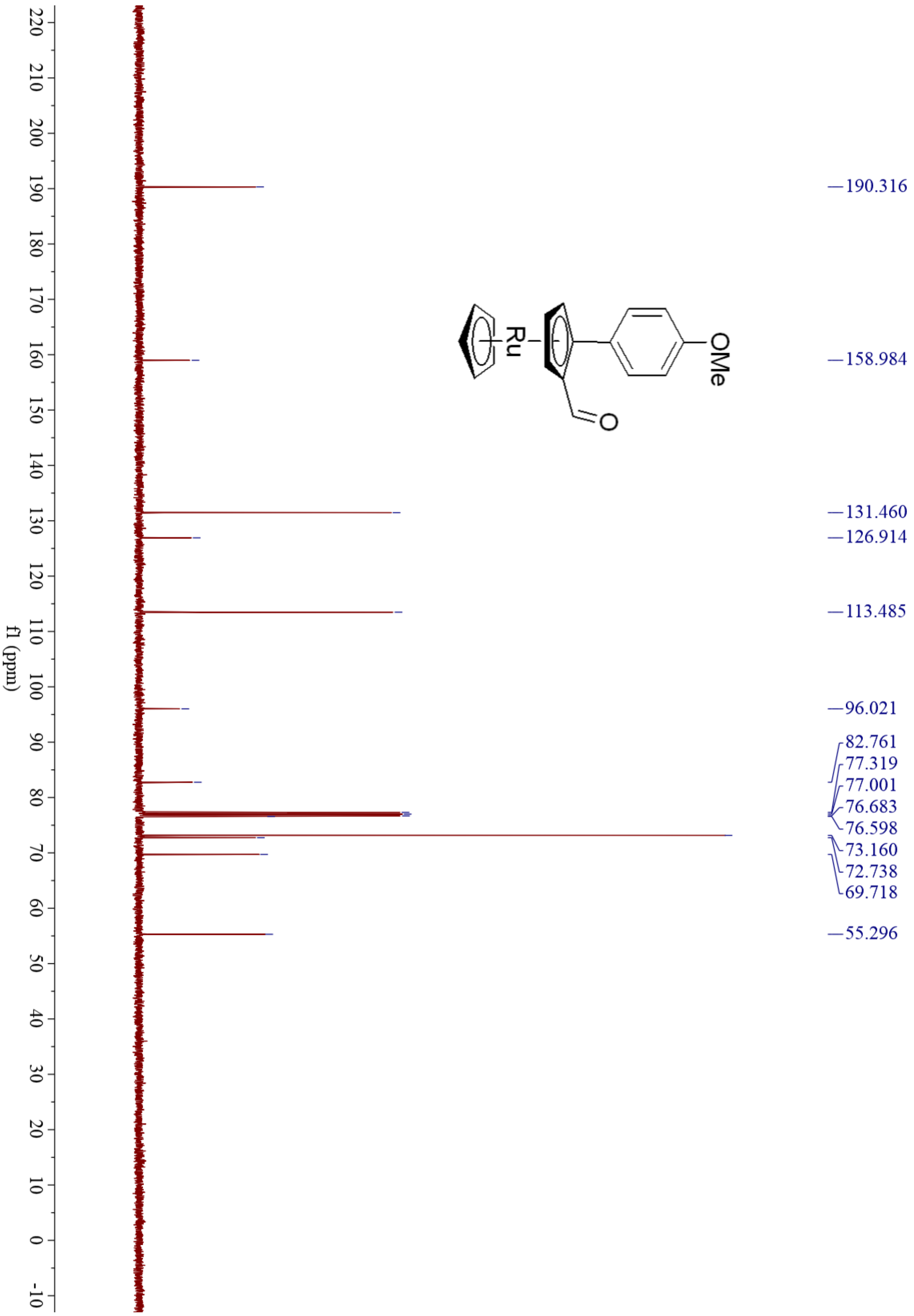

HPLC analysis of 3ba

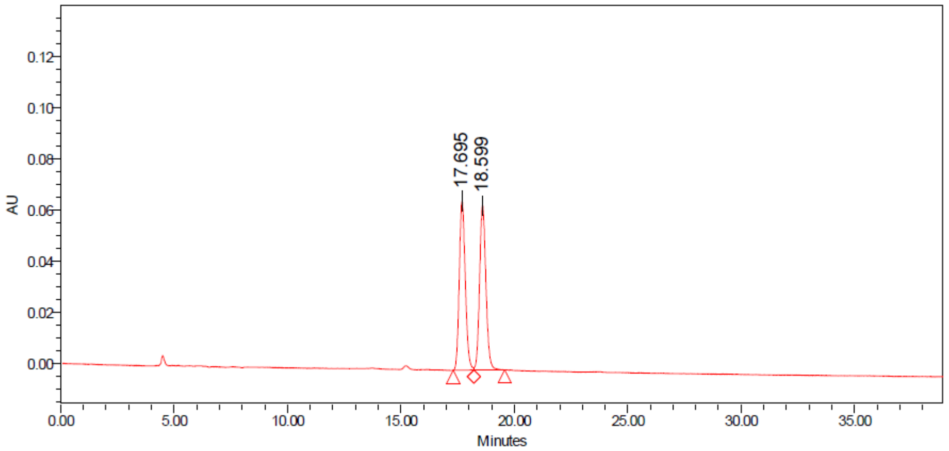

Peak Results

	SampleName	RT	Width (sec)	Height	Area	% Area
1	lcx-23-Ru-rac	17.695	54.800	66099	1219388	49.47
2	lcx-23-Ru-rac	18.599	81.000	64304	1245613	50.53

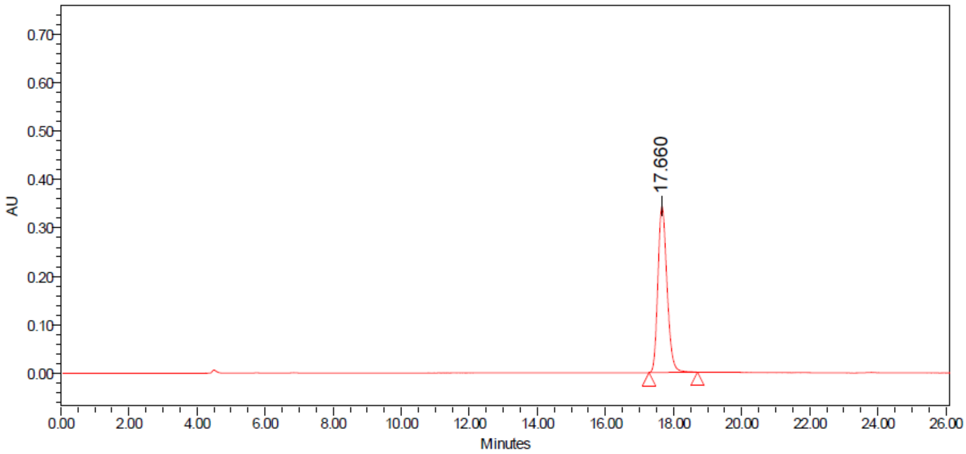

Peak Results

	SampleName	RT	Width (sec)	Height	Area	% Area
1	lcx-23-Ru-chiral	17.660	85.400	342867	6391444	100.00

^{1}H NMR spectra of 3ca

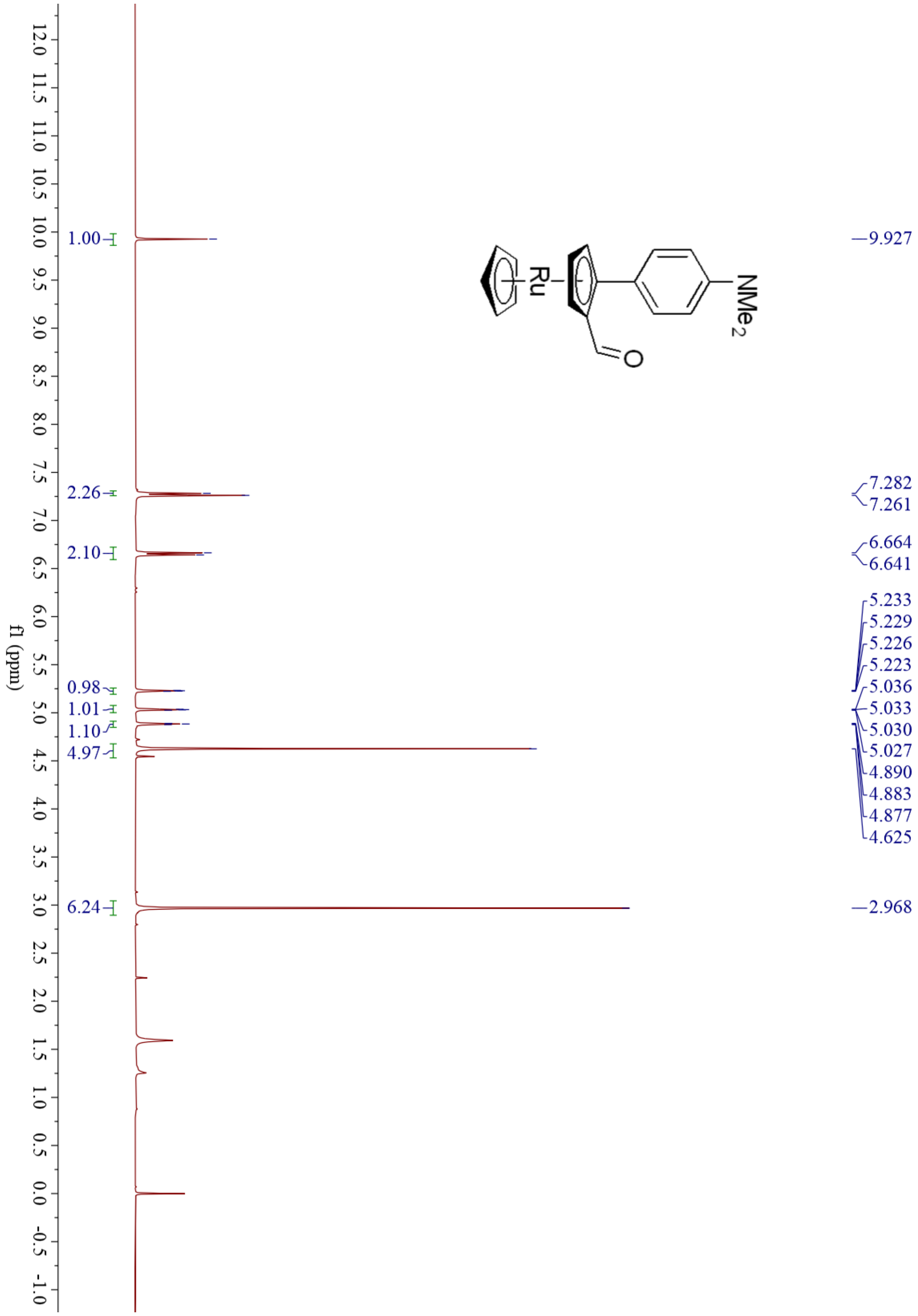

^{13}C NMR spectra of 3ca

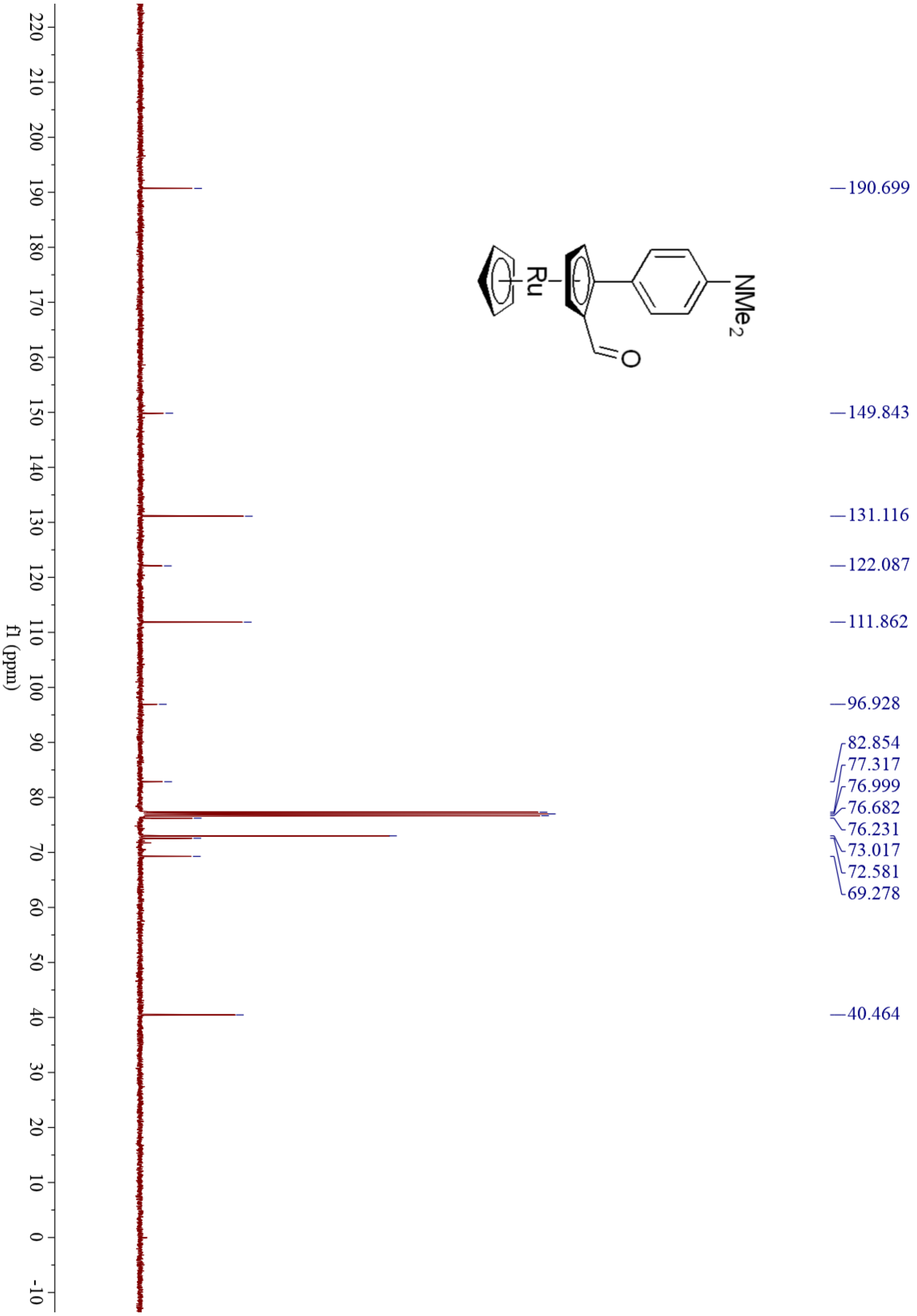

HPLC analysis of 3ca

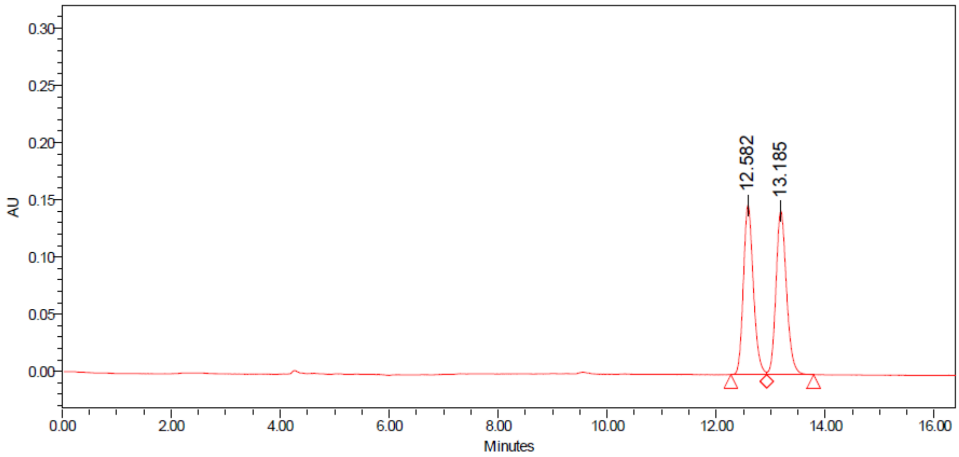

Peak Results

	SampleName	RT	Width (sec)	Height	Area	% Area
1	LCX-23-Ru-NMe2-RAC	12.582	39.100	147942	1879636	49.41
2	LCX-23-Ru-NMe2-RAC	13.185	51.900	142968	1924193	50.59

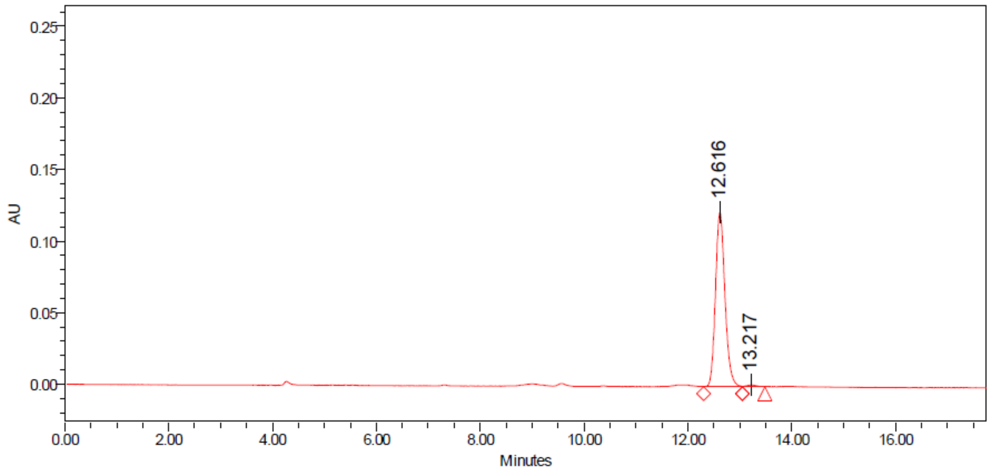

Peak Results

	SampleName	RT	Width (sec)	Height	Area	% Area
1	LCX-23-Ru-NMe2-chiral	12.616	44.900	121790	1545164	98.97
2	LCX-23-Ru-NMe2-chiral	13.217	26.000	1242	16100	1.03

^{1}H NMR spectra of 3da

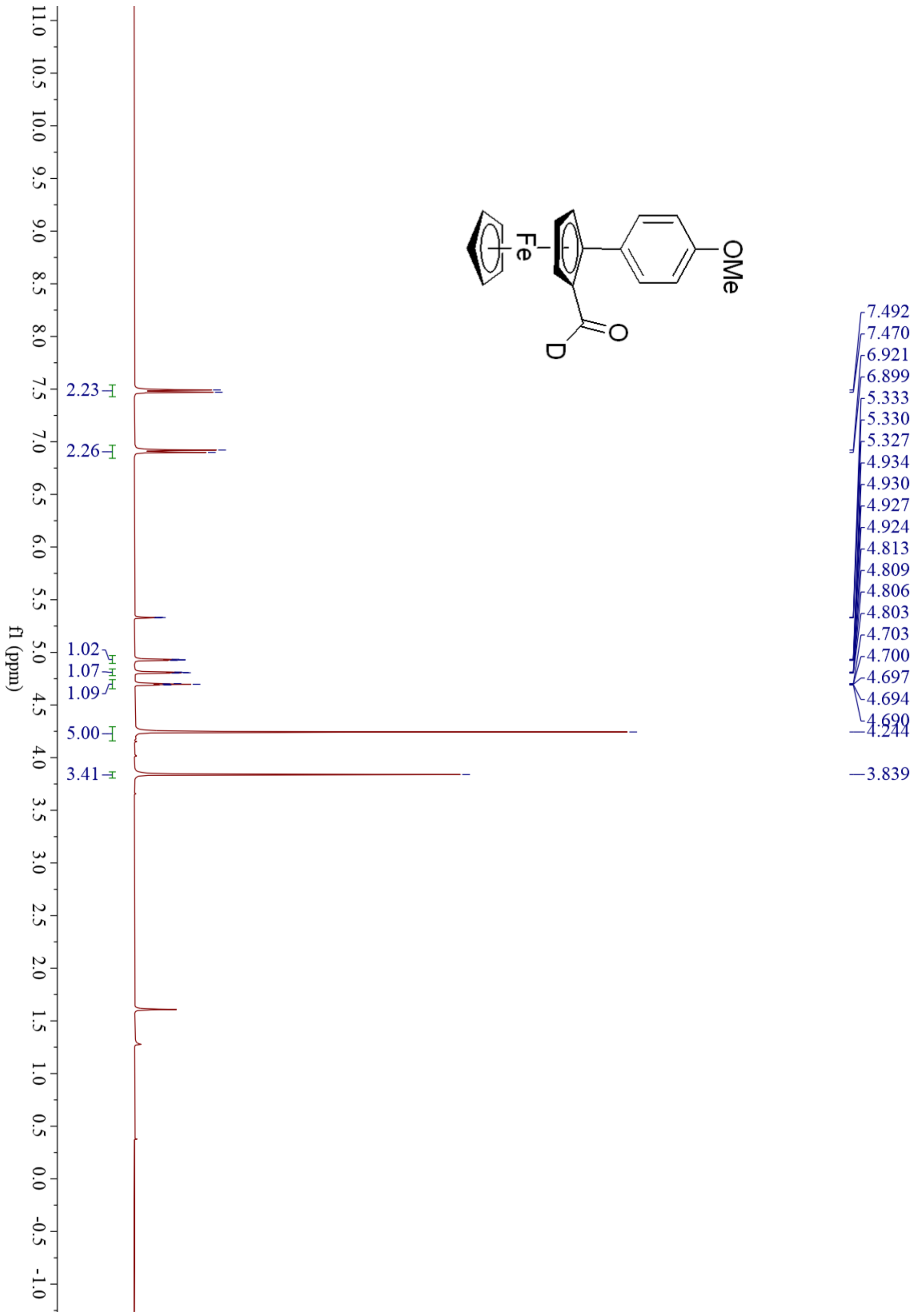

^{13}C NMR spectra of 3da

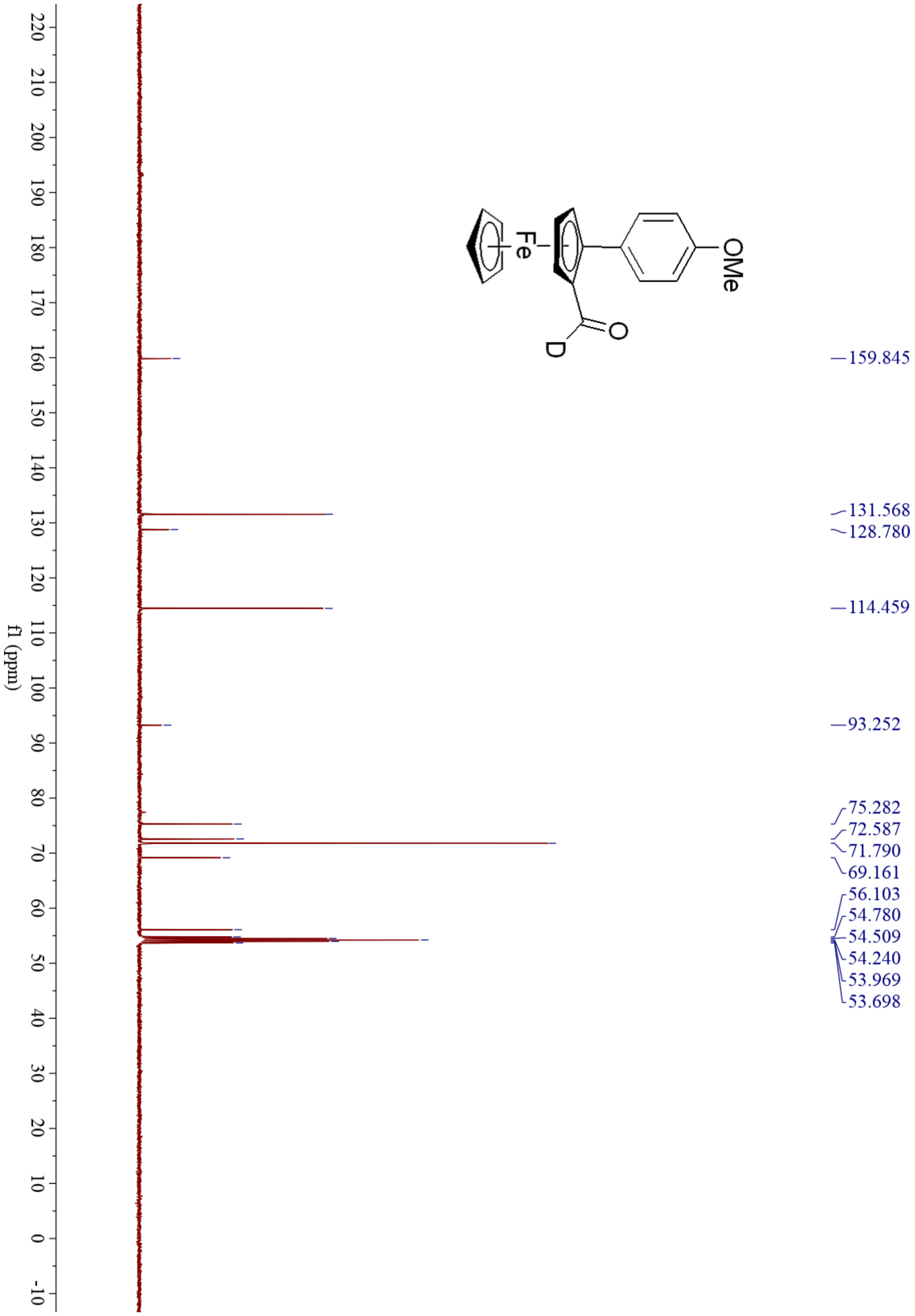

HPLC analysis of 3da

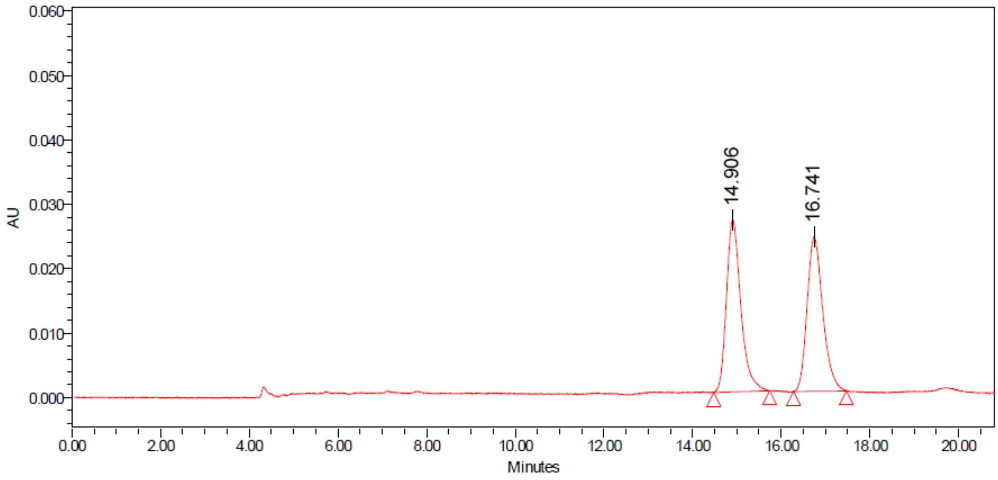

Peak Results

	SampleName	RT	Width (sec)	Height	Area	% Area
1	LCX-24-1'-Fe-CHD-rac	14.906	75.516	26653	600882	50.52
2	LCX-24-1'-Fe-CHD-rac	16.741	71.615	23991	588630	49.48

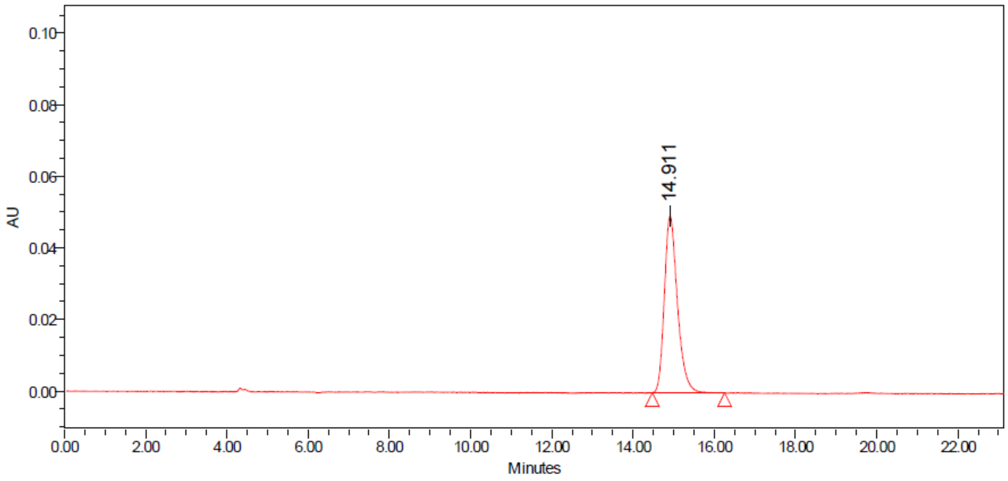

Peak Results

	SampleName	RT	Width (sec)	Height	Area	% Area
1	LCX-24-1'-Fe-CHD-CHIRAL	14.911	106.923	49382	1101497	100.00

^{1}H NMR spectra of 3fa

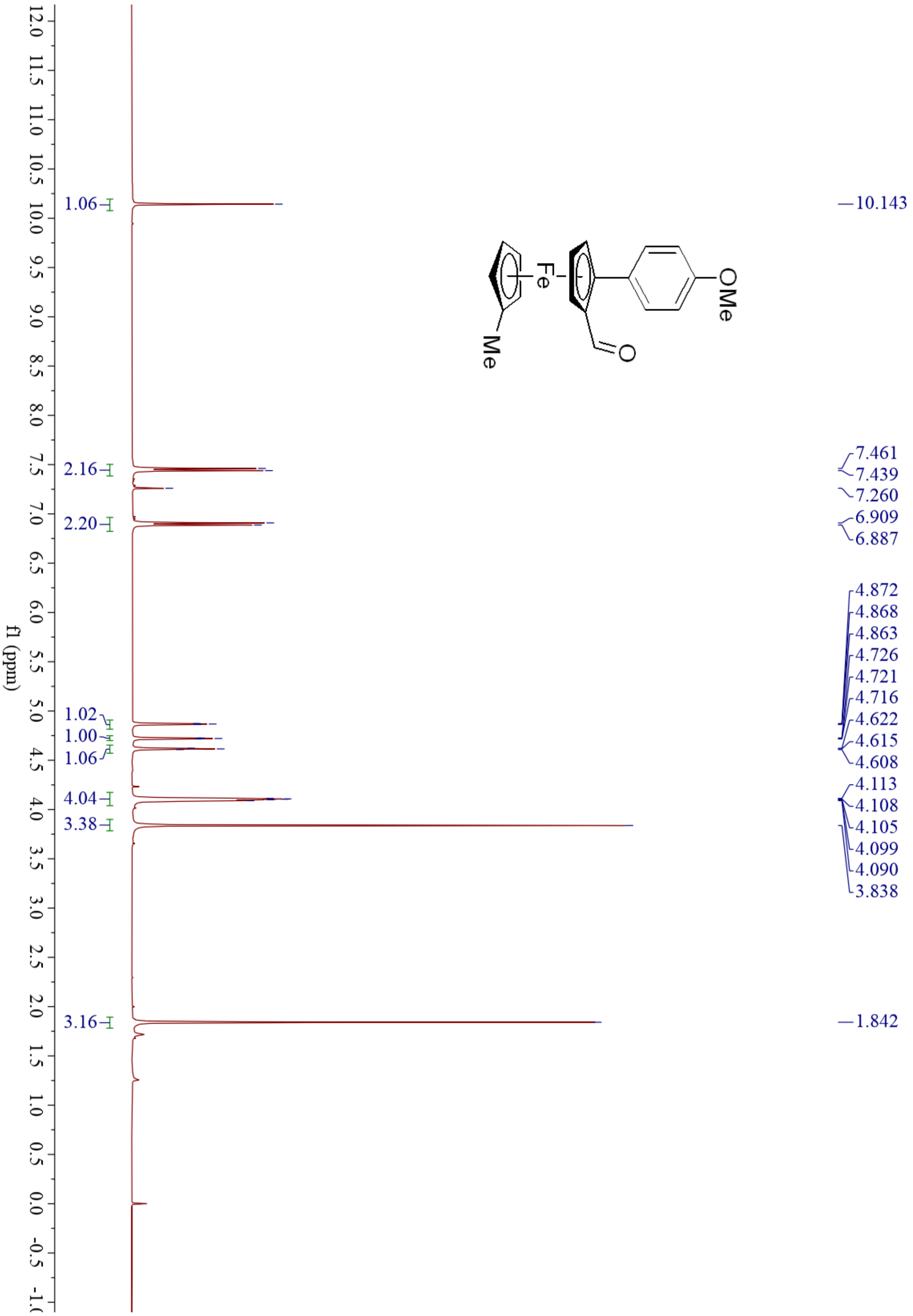

^{13}C NMR spectra of 3fa

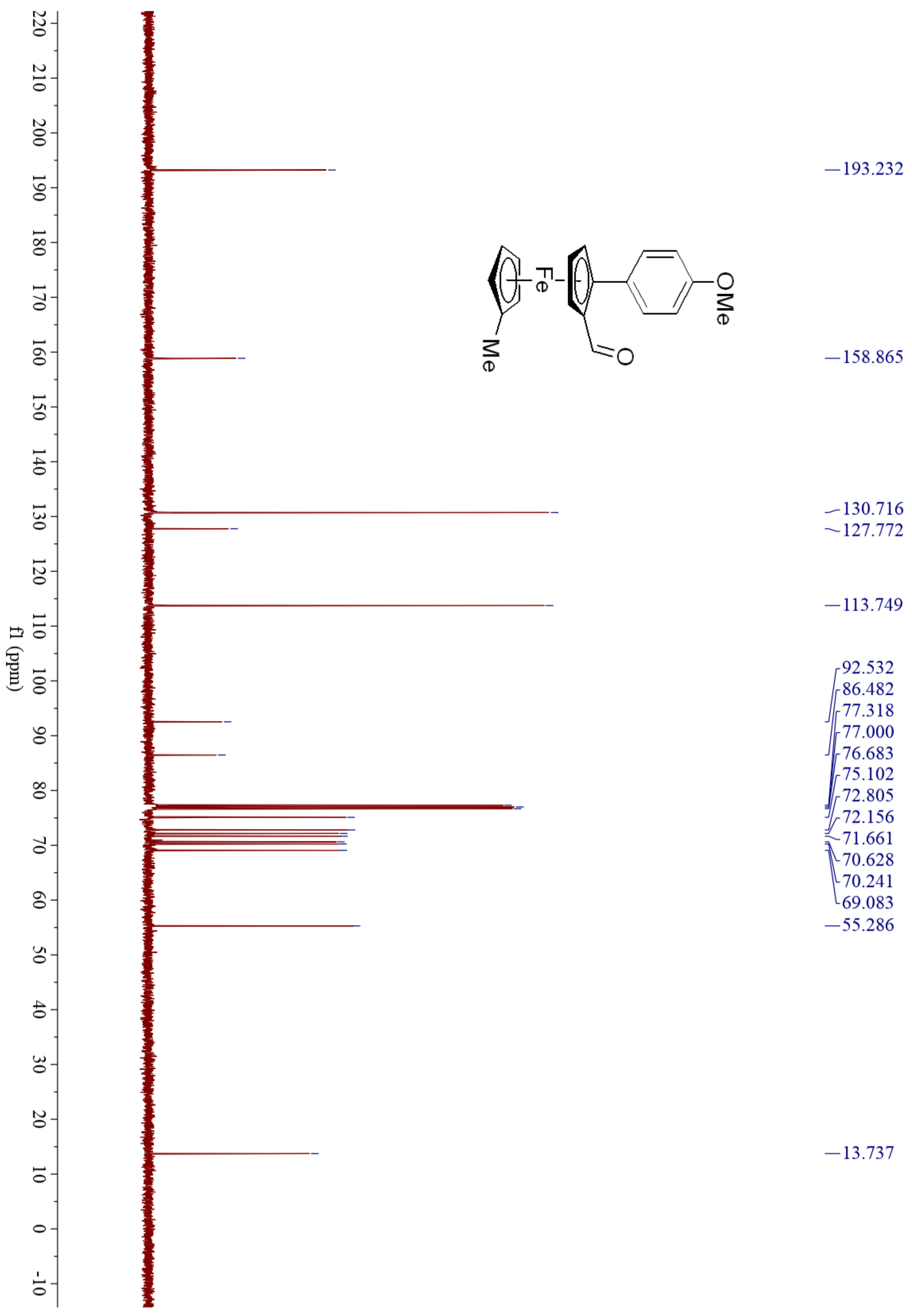

HPLC analysis of 3fa

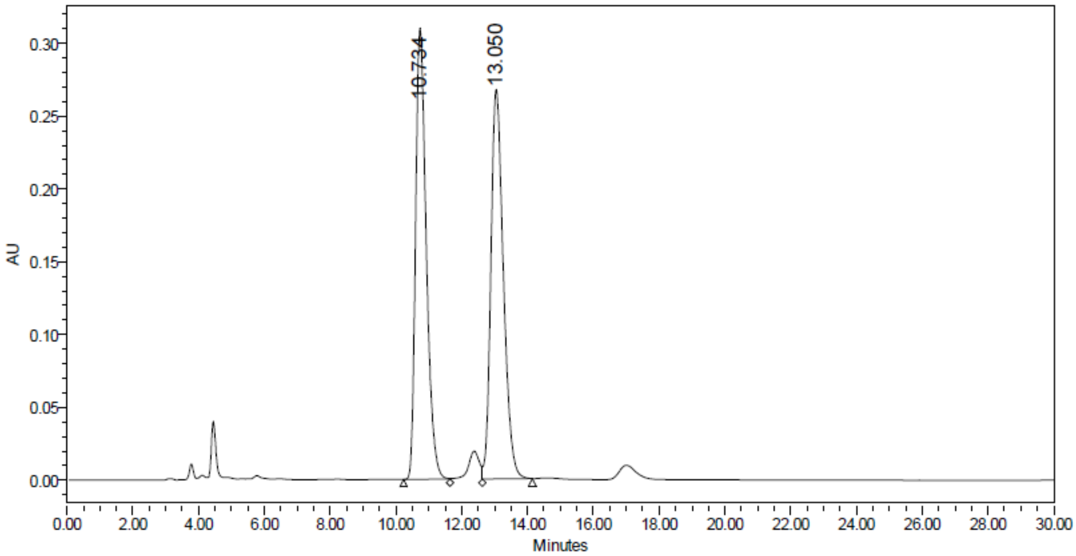

	RT	Area	% Area	Height
1	10.734	6976508	49.75	309860
2	13.050	7045942	50.25	267382

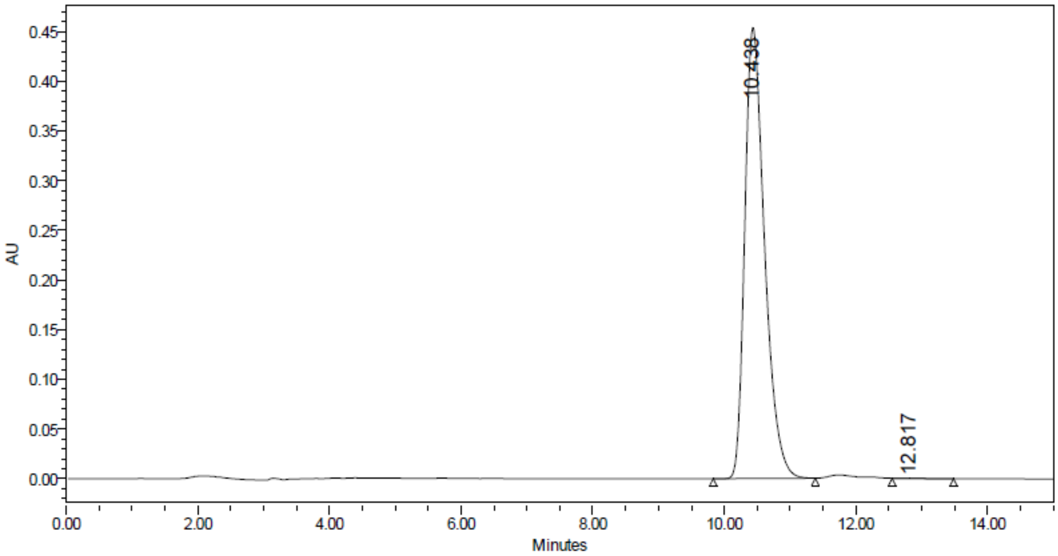

	RT	Area	% Area	Height
1	10.438	9440224	99.96	453674
2	12.817	4018	0.04	-127

^{1}H NMR spectra of 3ga

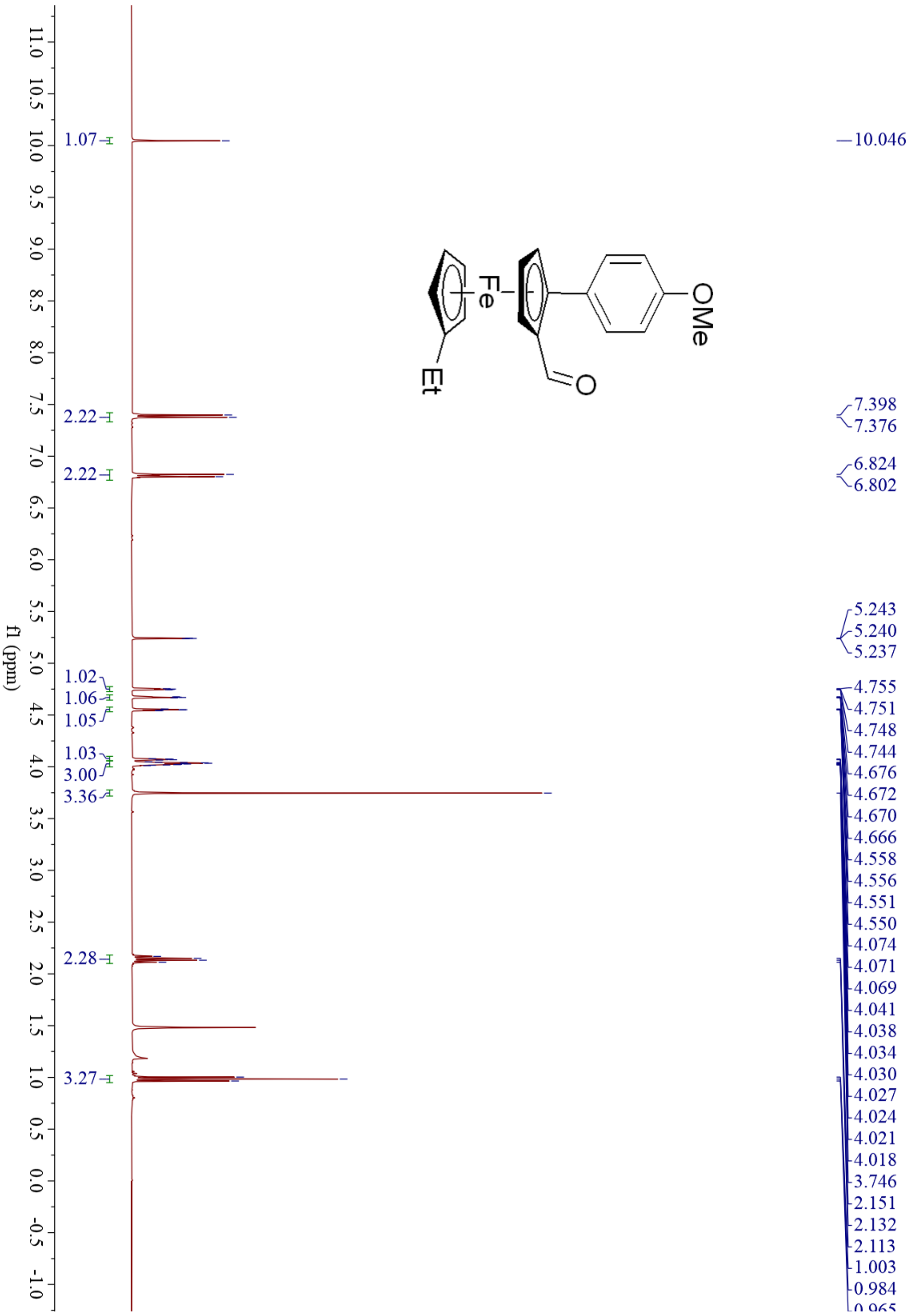

^{13}C NMR spectra of 3ga

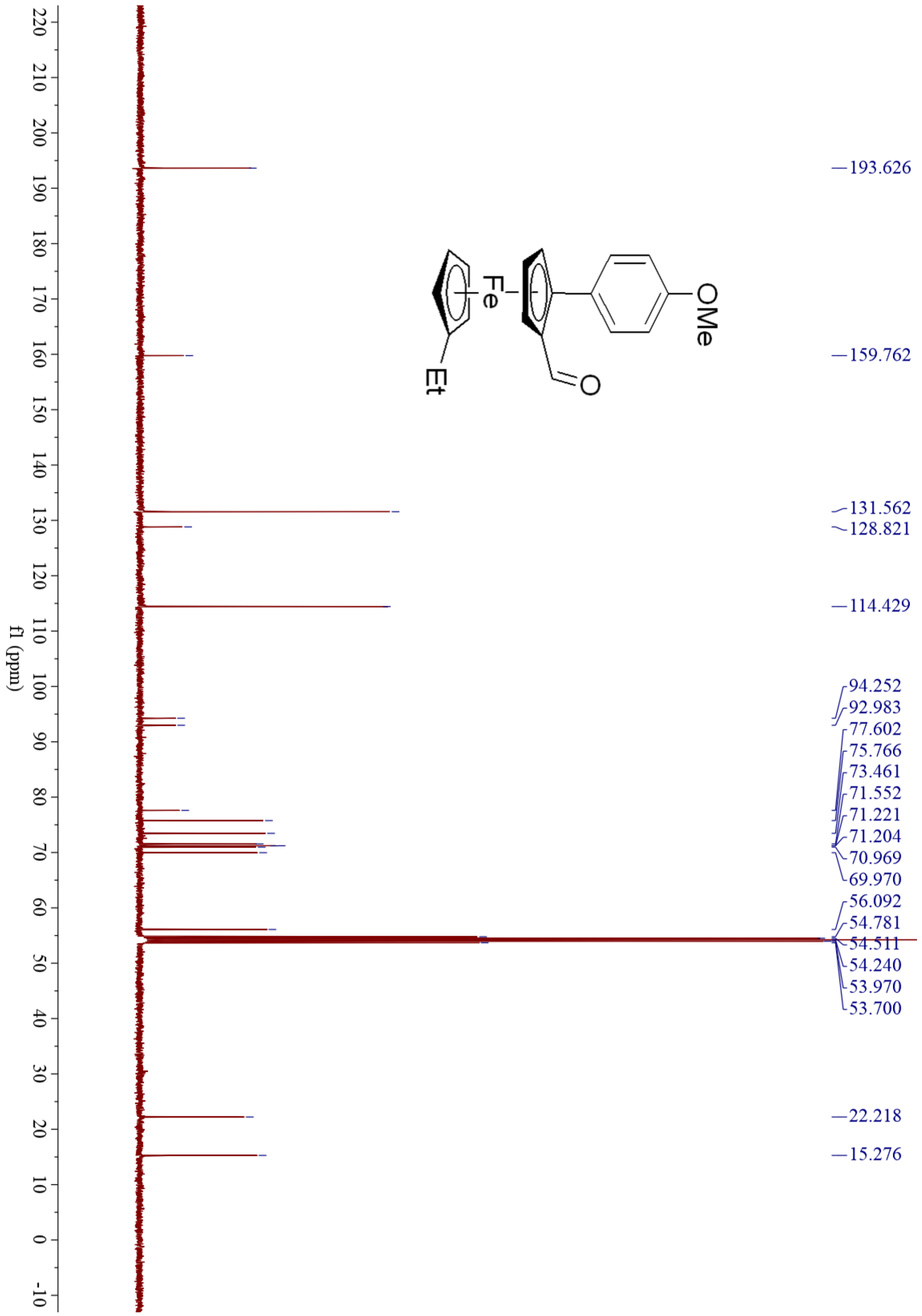

HPLC analysis of 3ga

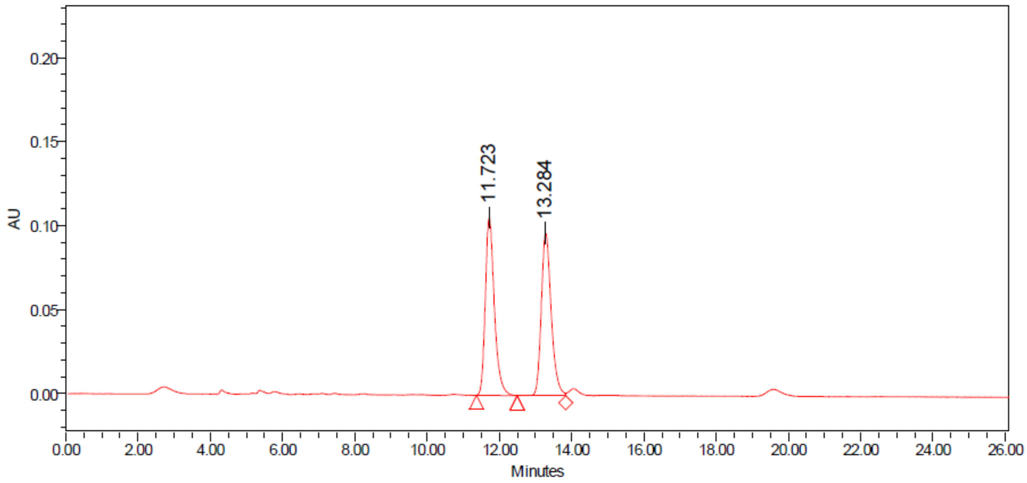

Peak Results

	SampleName	RT	Width (sec)	Height	Area	% Area
1	lcx-24-1'-Fc-Et-rac	11.723	67.414	105927	1850446	50.06
2	lcx-24-1'-Fc-Et-rac	13.284	80.817	96746	1846369	49.94

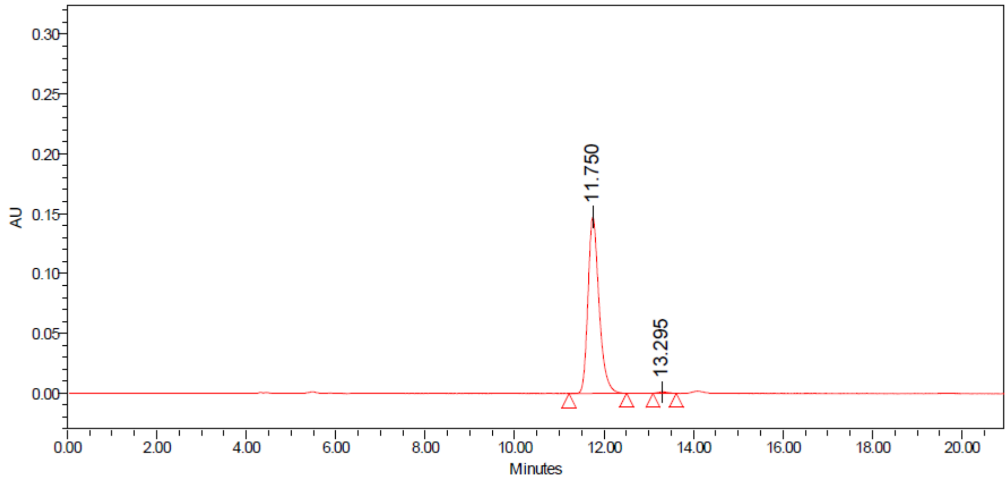

Peak Results

	SampleName	RT	Width (sec)	Height	Area	% Area
1	lcx-24-1'-Fc-Et-chiral	11.750	77.417	147449	2535930	99.31
2	lcx-24-1'-Fc-Et-chiral	13.295	31.207	1139	17613	0.69

^{1}H NMR spectra of 3ha

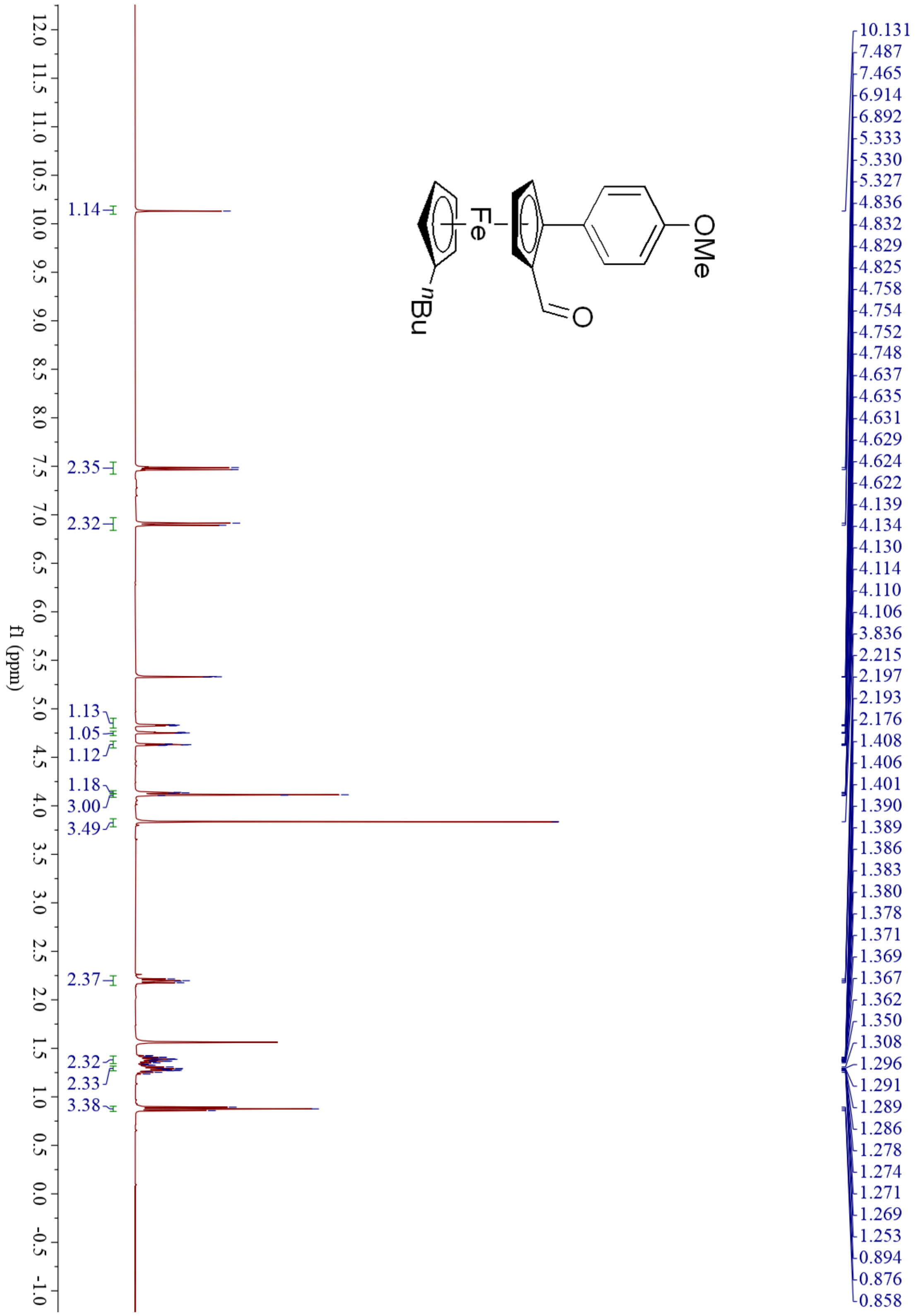

^{13}C NMR spectra of 3ha

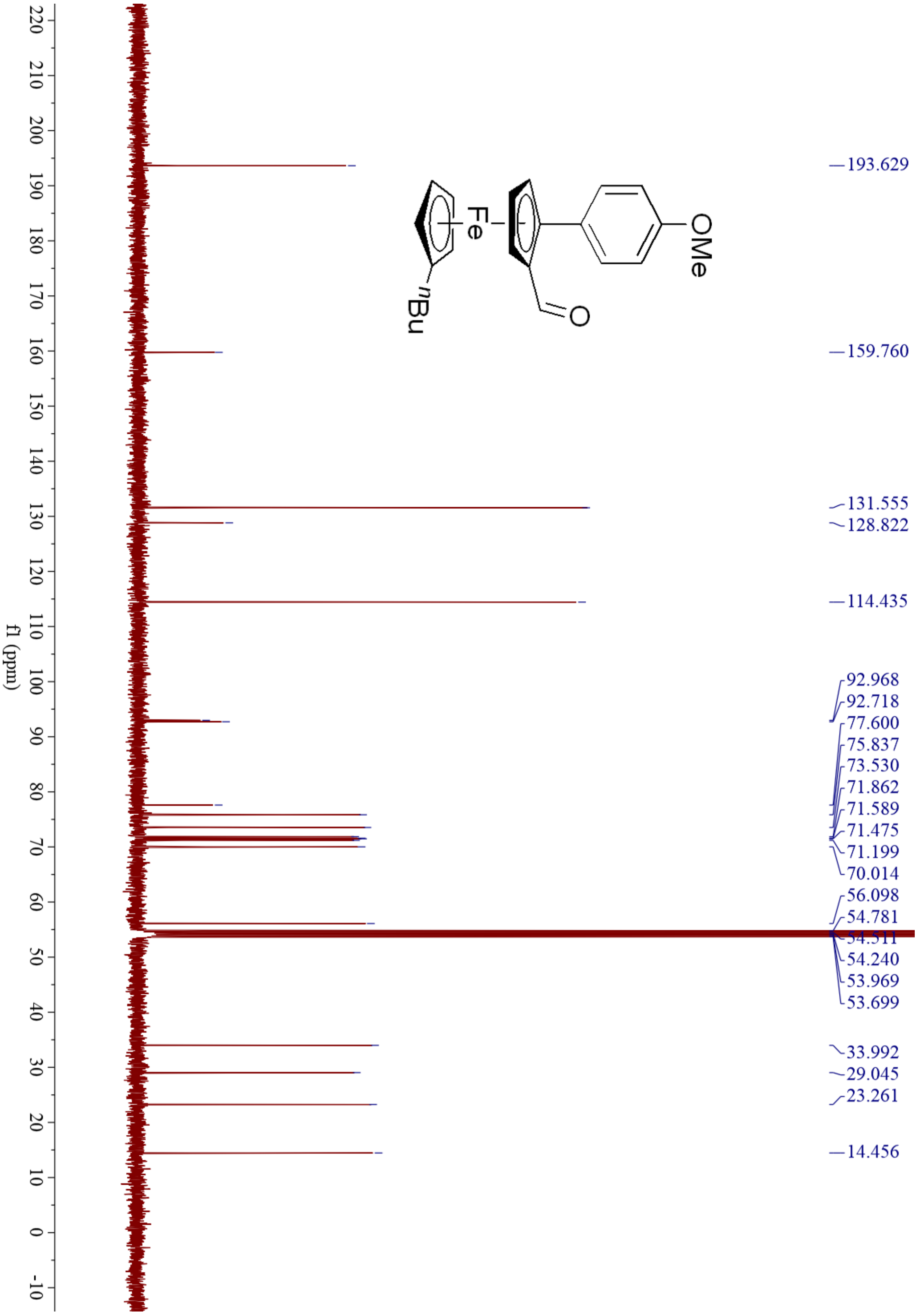

HPLC analysis of 3ha

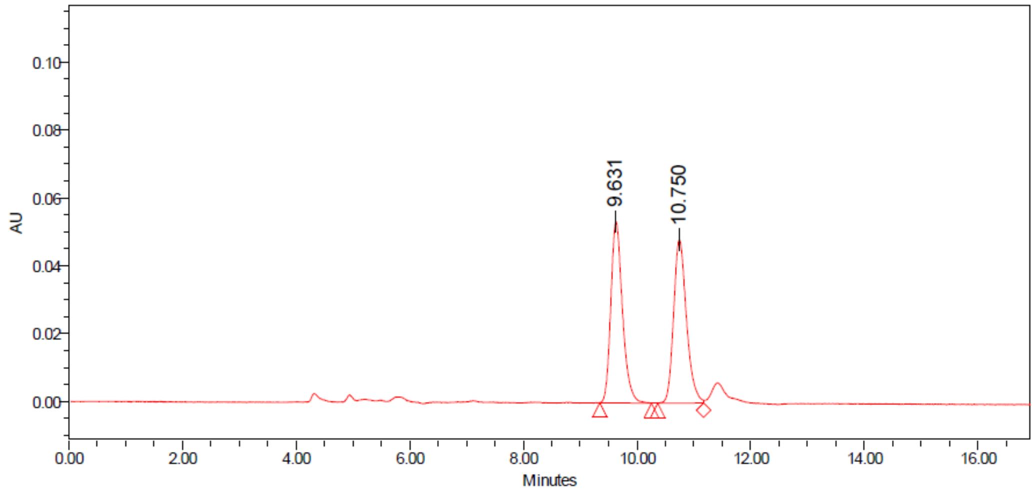

Peak Results

	SampleName	RT	Width (sec)	Height	Area	% Area
1	lcx-24-1'-Fc-Bu-rac	9.631	54.612	53413	762064	50.11
2	lcx-24-1'-Fc-Bu-rac	10.750	48.710	48156	758636	49.89

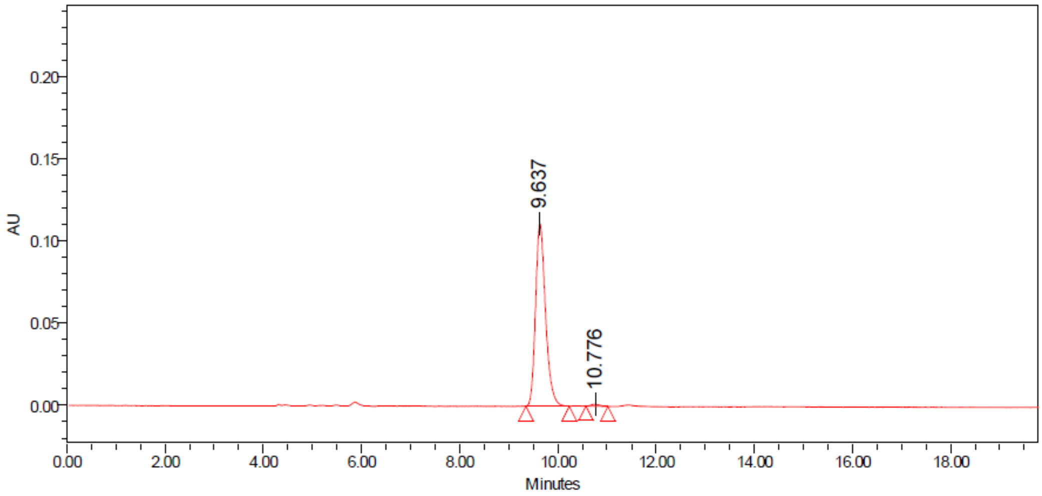

Peak Results

	SampleName	RT	Width (sec)	Height	Area	% Area
1	lcx-24-1'-Fc-Bu-chiral	9.637	52.911	111117	1564263	99.01
2	lcx-24-1'-Fc-Bu-chiral	10.776	27.006	1138	15665	0.99

^{1}H NMR spectra of 3ia

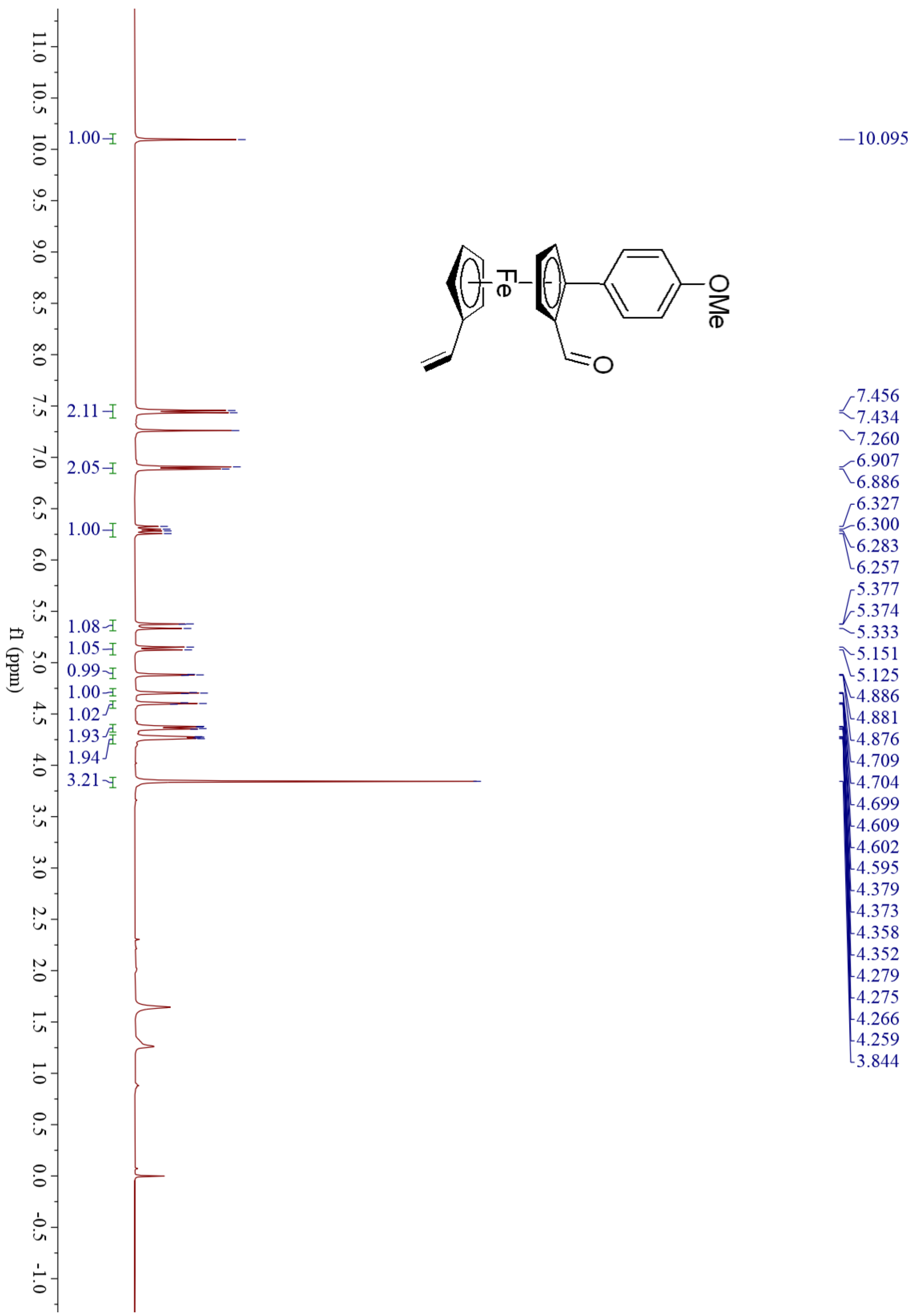

^{13}C NMR spectra of 3ia

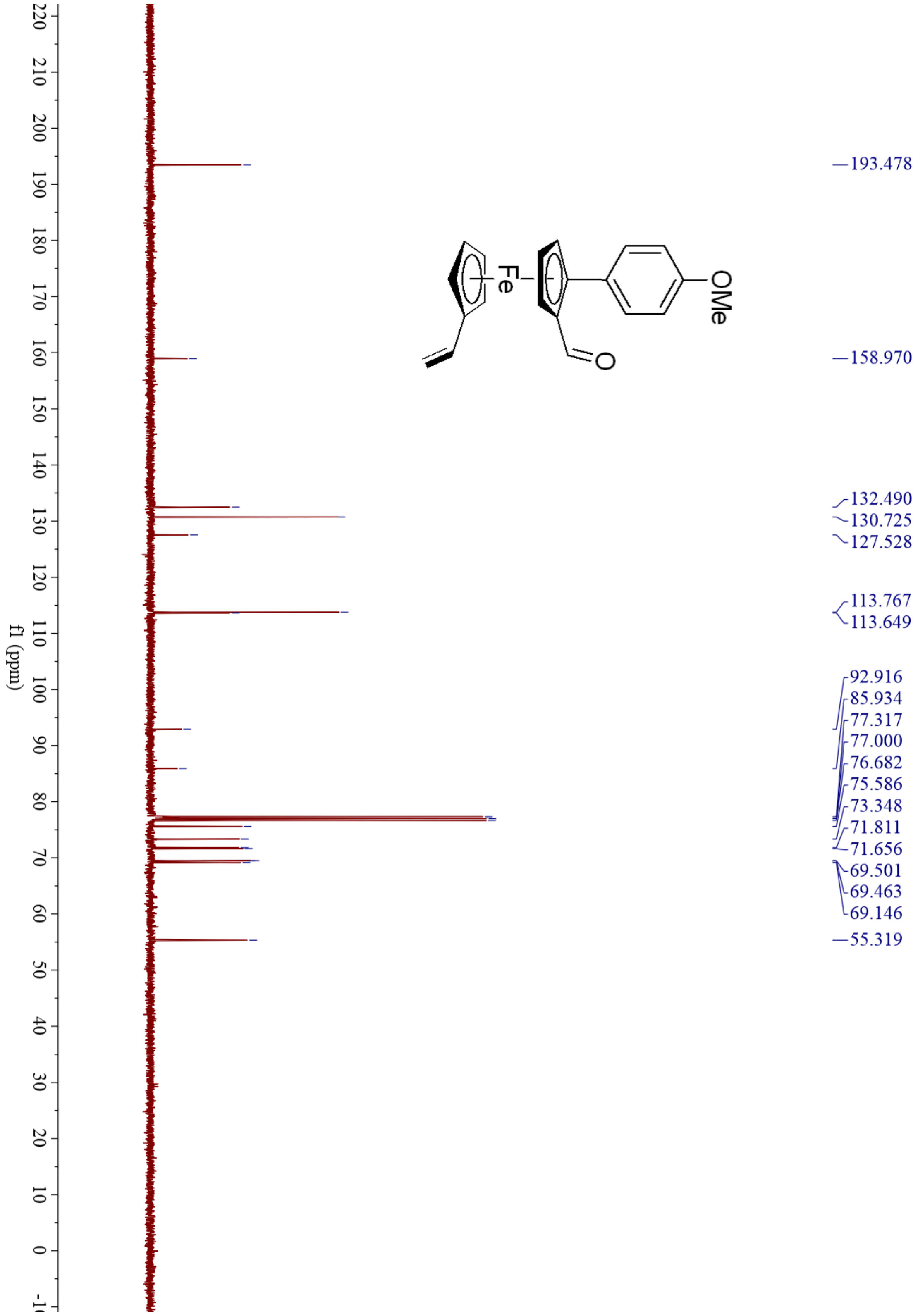

HPLC analysis of 3ia

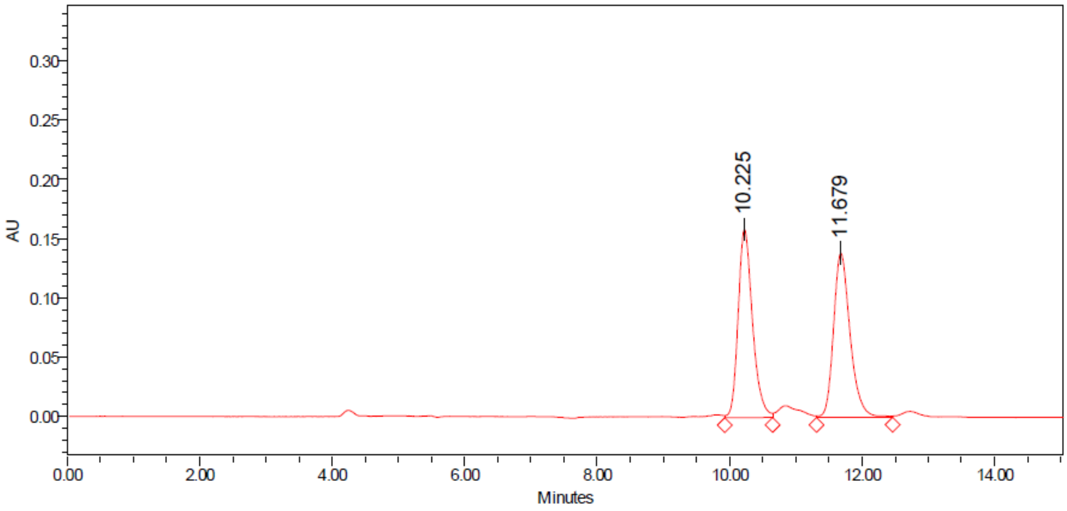

Peak Results

	SampleName	RT	Width (sec)	Height	Area	% Area
1	LCX-23-ch=ch2--rac	10.225	43.300	158711	2392596	49.92
2	LCX-23-ch=ch2--rac	11.679	68.900	138485	2400307	50.08

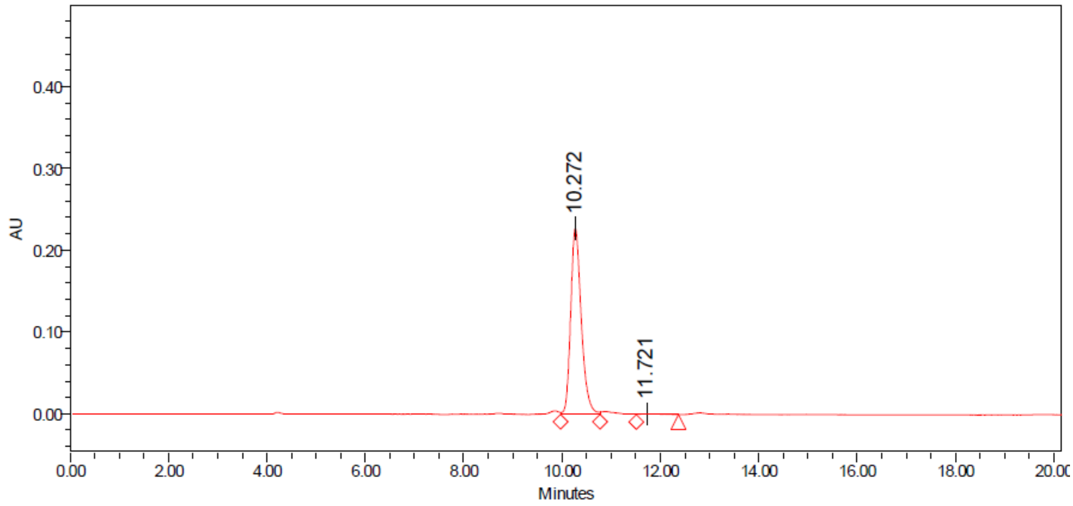

Peak Results

	SampleName	RT	Width (sec)	Height	Area	% Area
1	LCX-23-ch=ch2-chiral	10.272	48.000	227419	3405940	99.60
2	LCX-23-ch=ch2-chiral	11.721	51.400	521	13736	0.40

^{1}H NMR spectra of 3ja

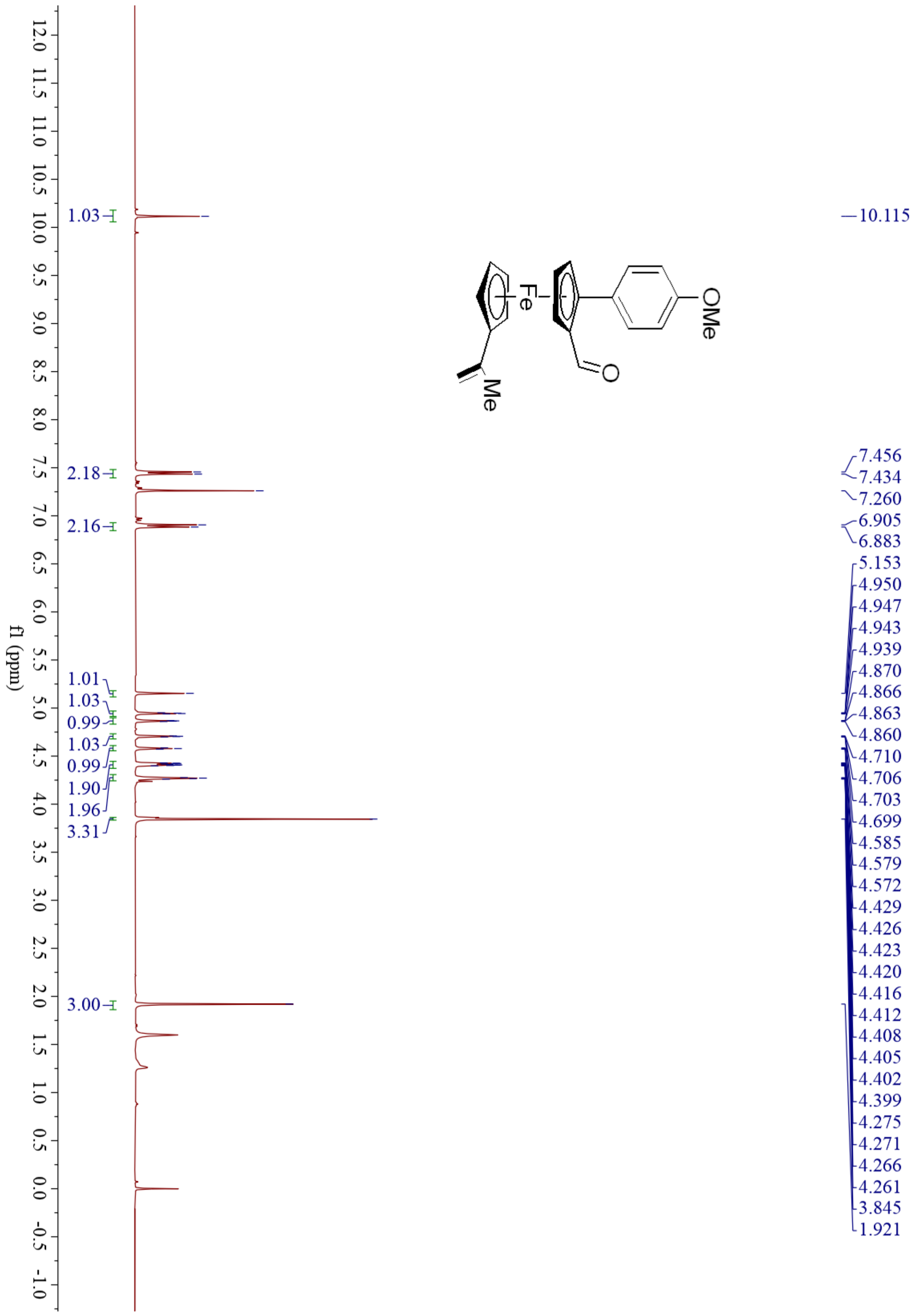

^{13}C NMR spectra of 3ja

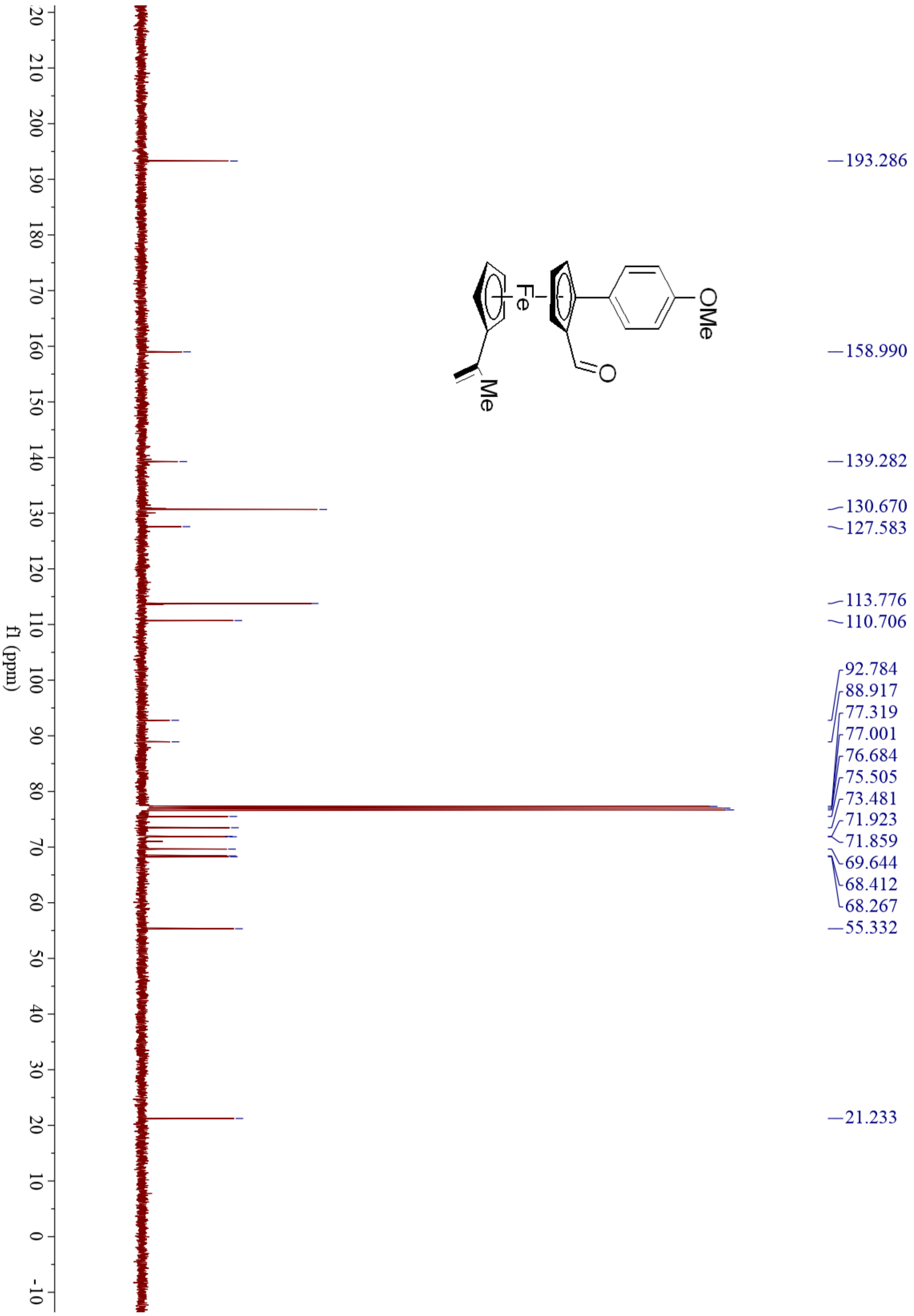

HPLC analysis of 3ja

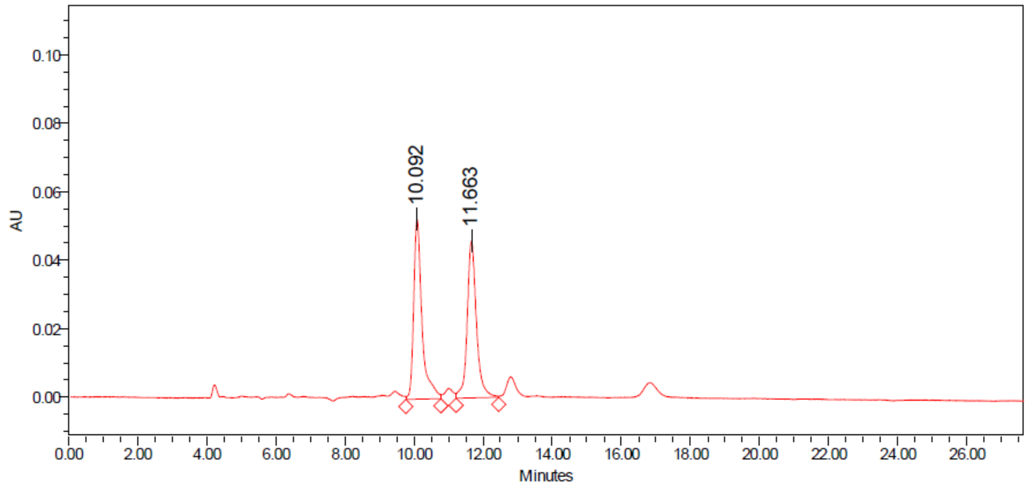

Peak Results

	SampleName	RT	Width (sec)	Height	Area	% Area
1	LCX-23-ch3c=ch2-rac	10.092	61.100	52587	877980	50.77
2	LCX-23-ch3c=ch2-rac	11.663	74.200	45818	851339	49.23

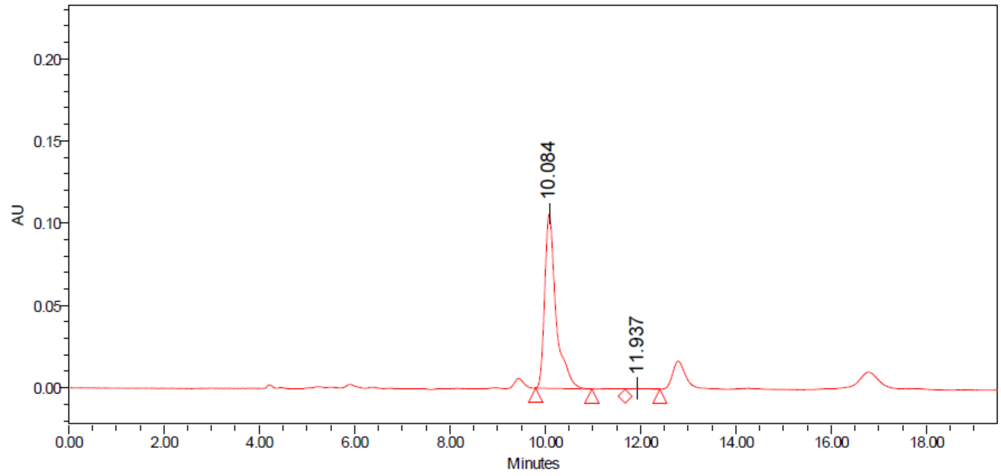

Peak Results

	SampleName	RT	Width (sec)	Height	Area	% Area
1	LCX-23-ch3c=ch2-chiral	10.084	70.800	105965	1760484	99.43
2	LCX-23-ch3c=ch2-chiral	11.937	43.700	474	10065	0.57

^{1}H NMR spectra of 3ka

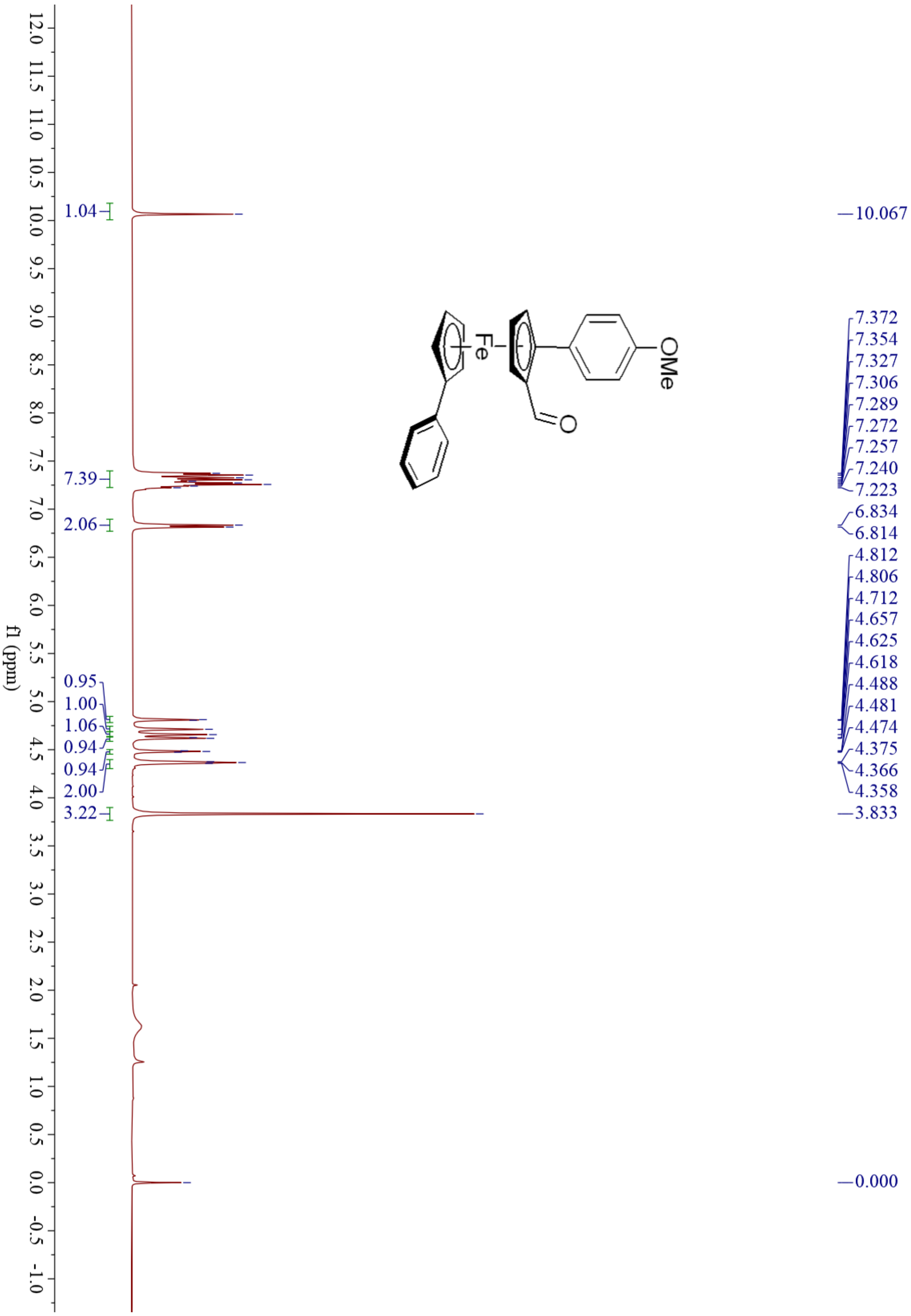

^{13}C NMR spectra of 3ka

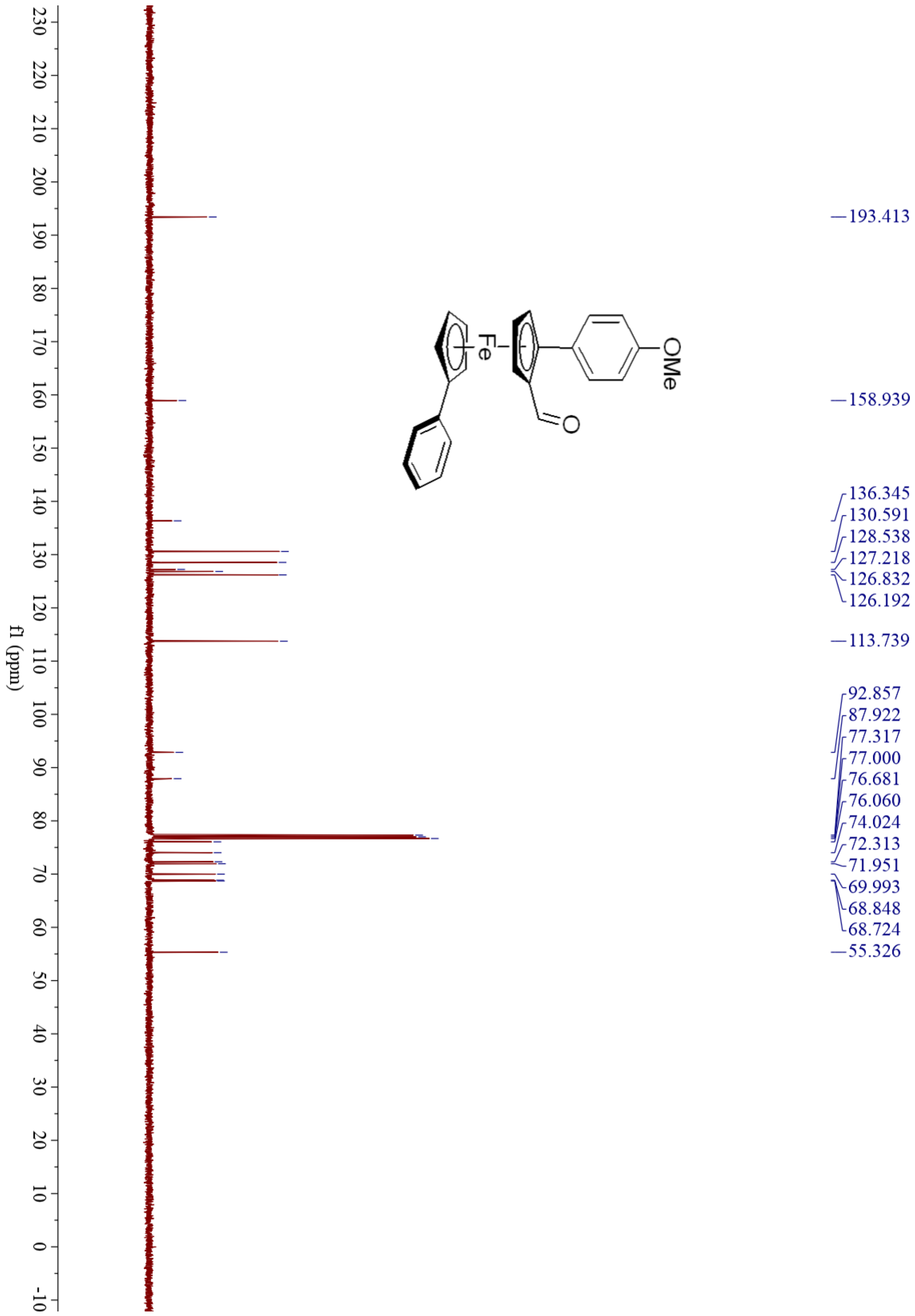

HPLC analysis of 3ka

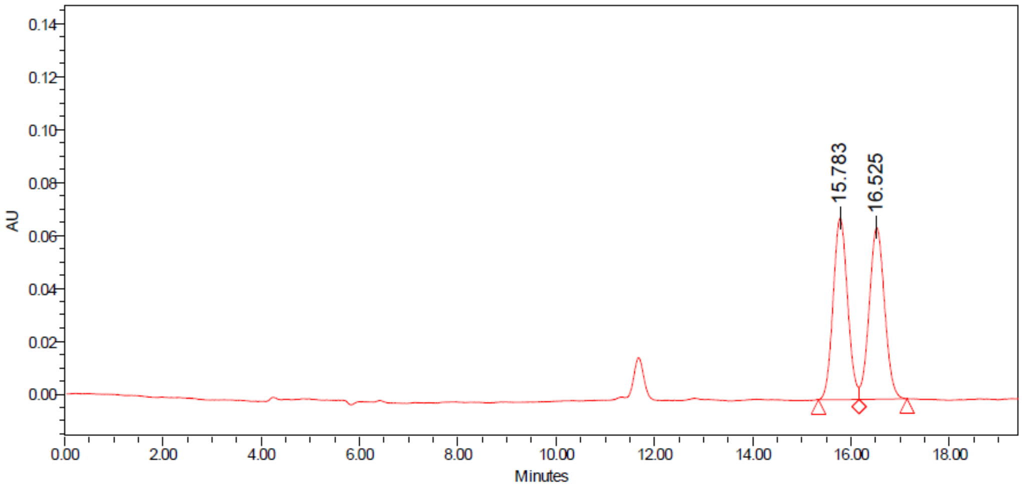

Peak Results

	SampleName	RT	Width (sec)	Height	Area	% Area
1	lcx-23-1'-ph-rac	15.783	49.700	68642	1397802	49.86
2	lcx-23-1'-ph-rac	16.525	58.500	64840	1405774	50.14

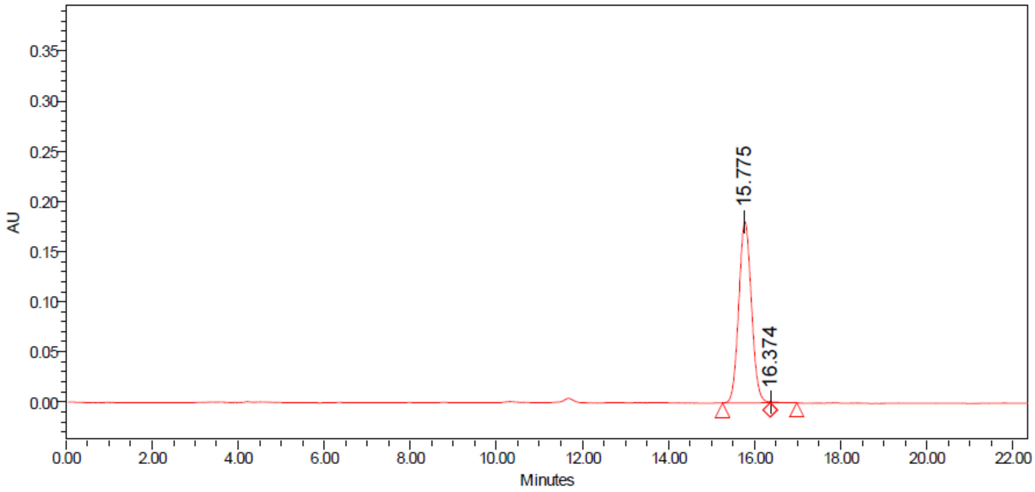

Peak Results

	SampleName	RT	Width (sec)	Height	Area	% Area
1	lcx-23-1'-ph-chiral	15.775	66.700	180643	3693268	99.57
2	lcx-23-1'-ph-chiral	16.374	36.800	989	15828	0.43

^{1}H NMR spectra of 3la

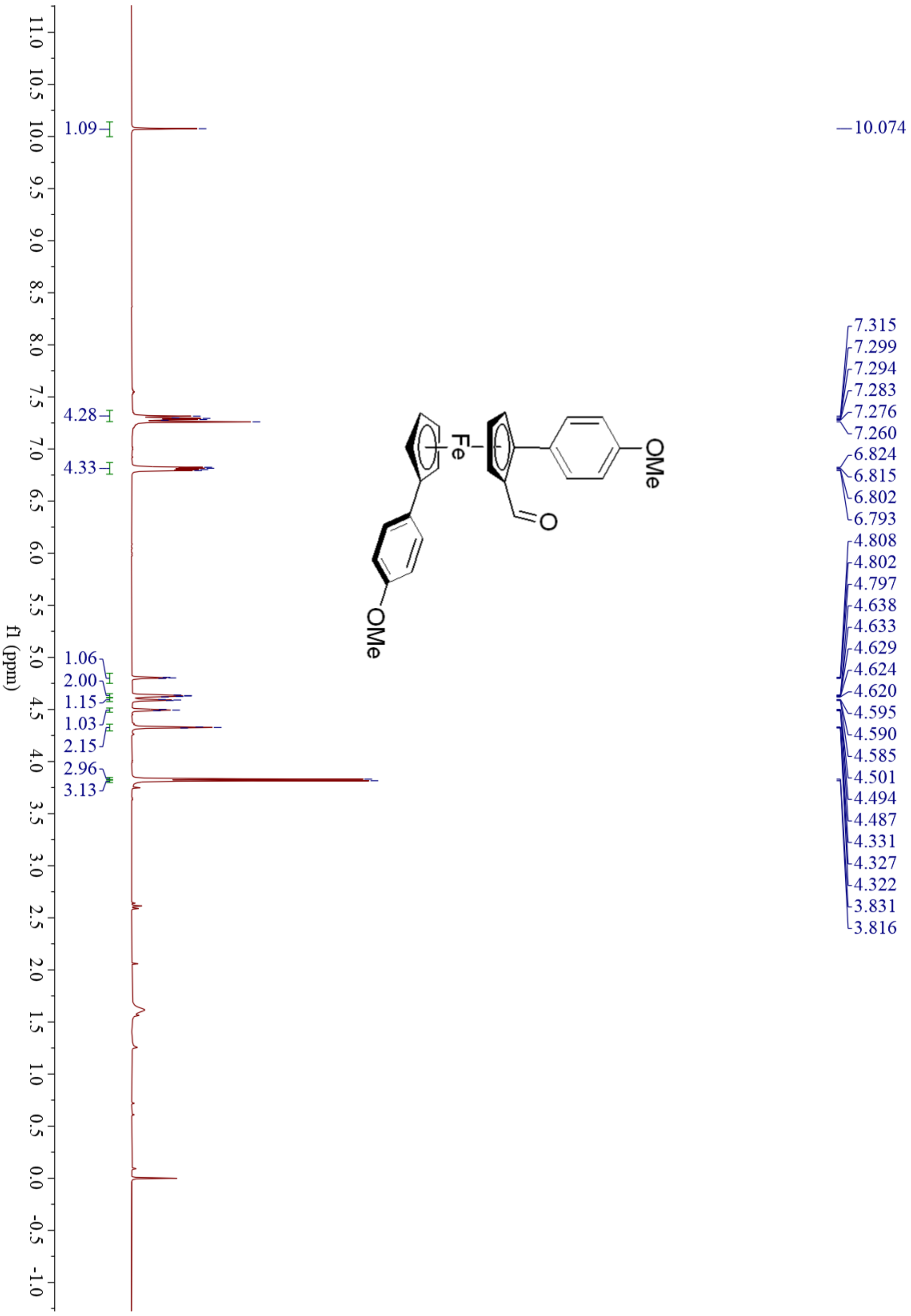

^{13}C NMR spectra of 3la

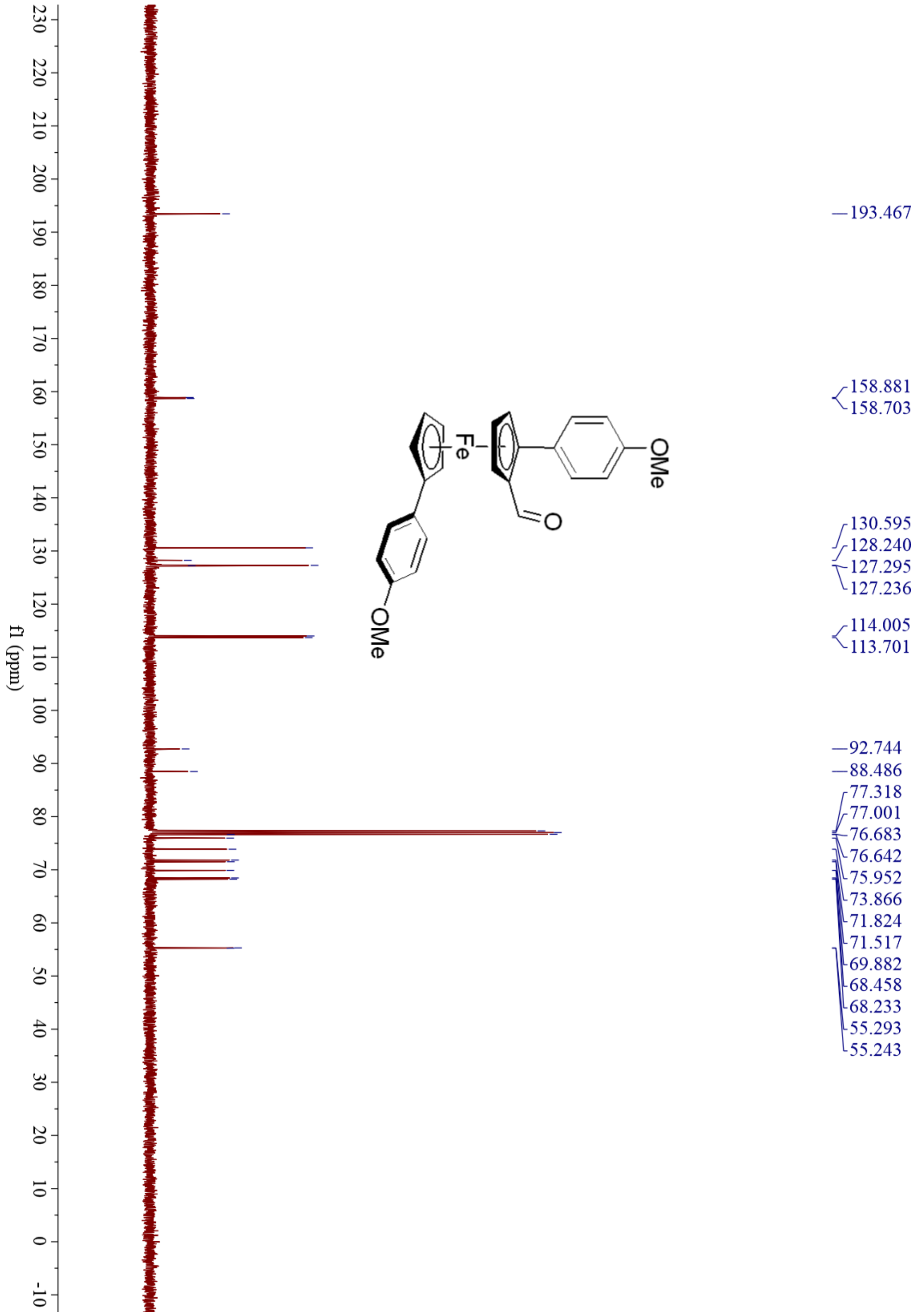

HPLC analysis of 3la

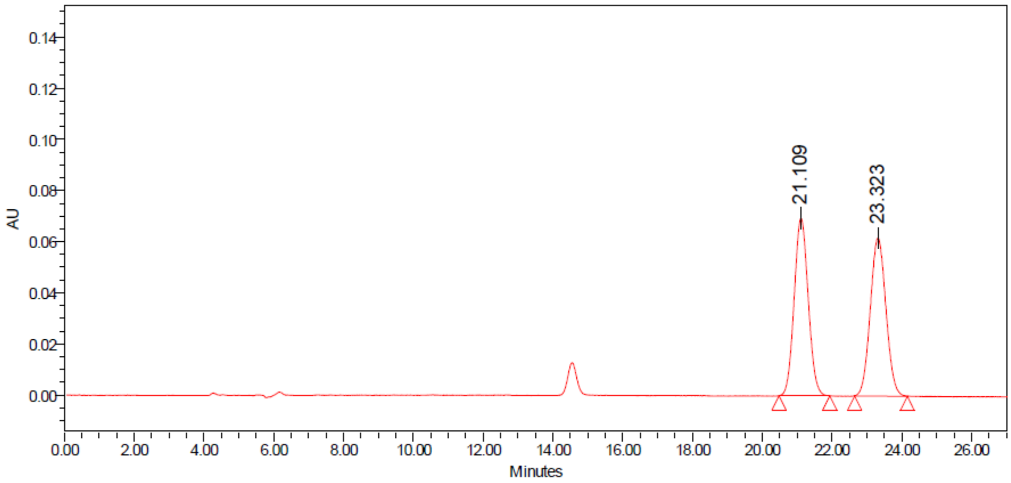

Peak Results

	SampleName	RT	Width (sec)	Height	Area	% Area
1	LCX-23-33-rac	21.109	87.100	69480	1944814	50.33
2	LCX-23-33-rac	23.323	90.600	61783	1919581	49.67

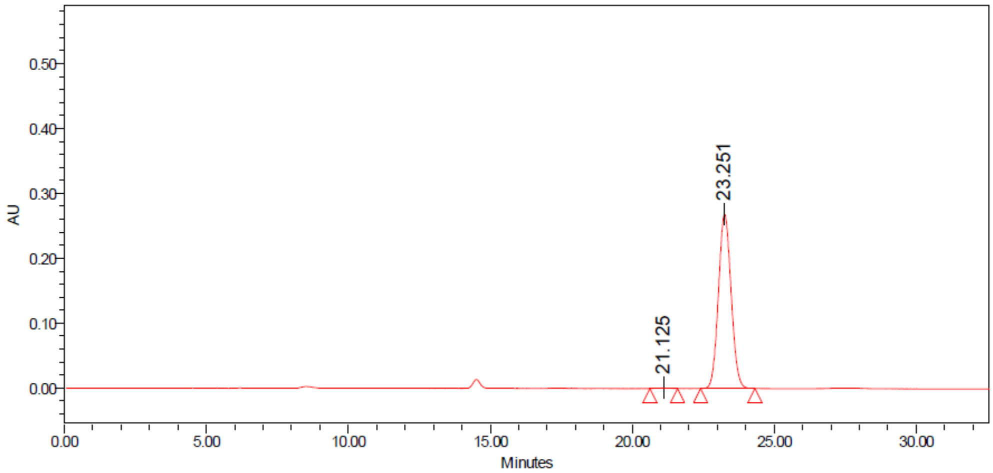

Peak Results

	SampleName	RT	Width (sec)	Height	Area	% Area
1	LCX-23-33-chiral	21.125	58.300	1045	26980	0.32
2	LCX-23-33-chiral	23.251	114.900	268627	8330819	99.68

^{1}H NMR spectra of 3ma

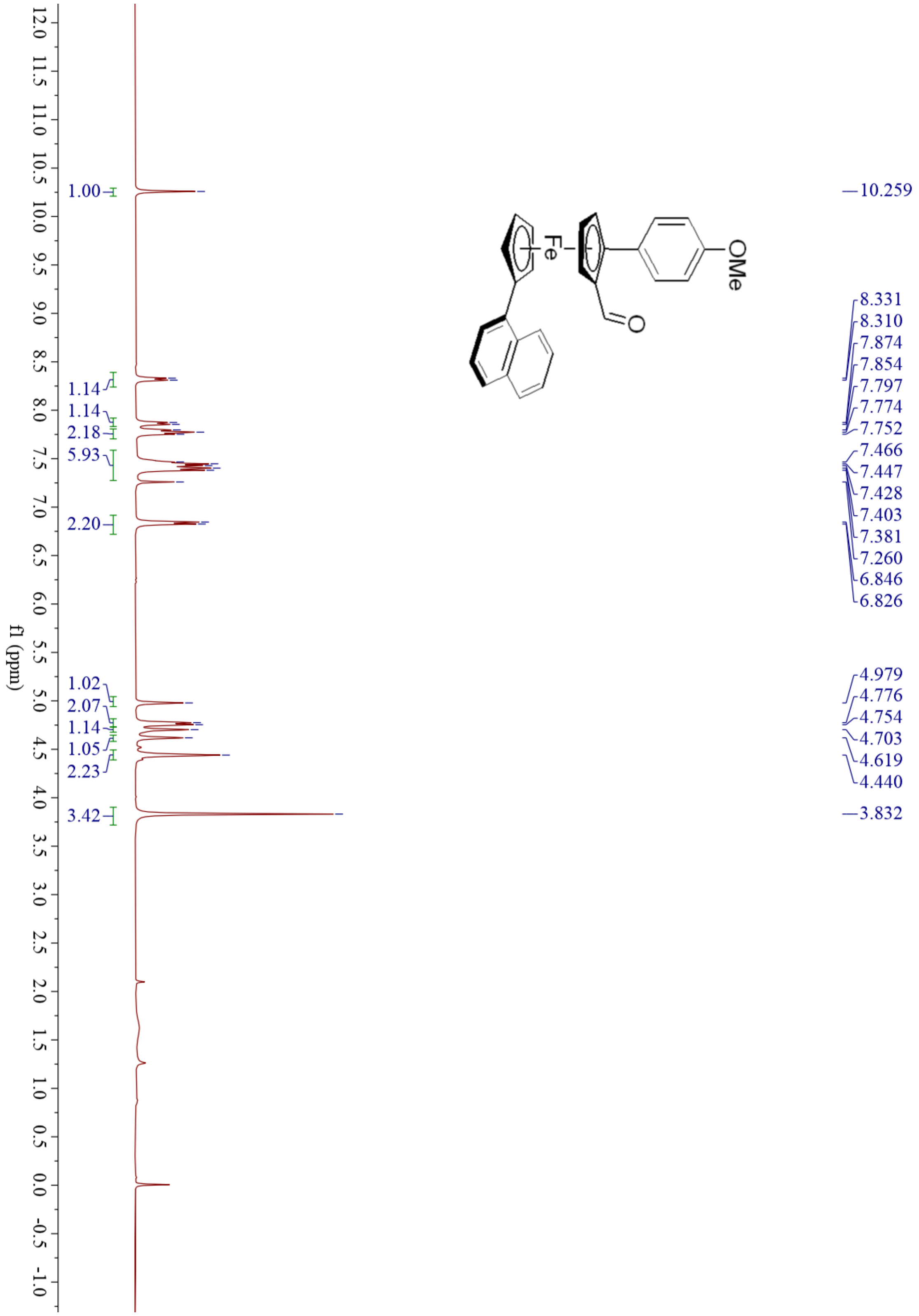

^{13}C NMR spectra of 3ma

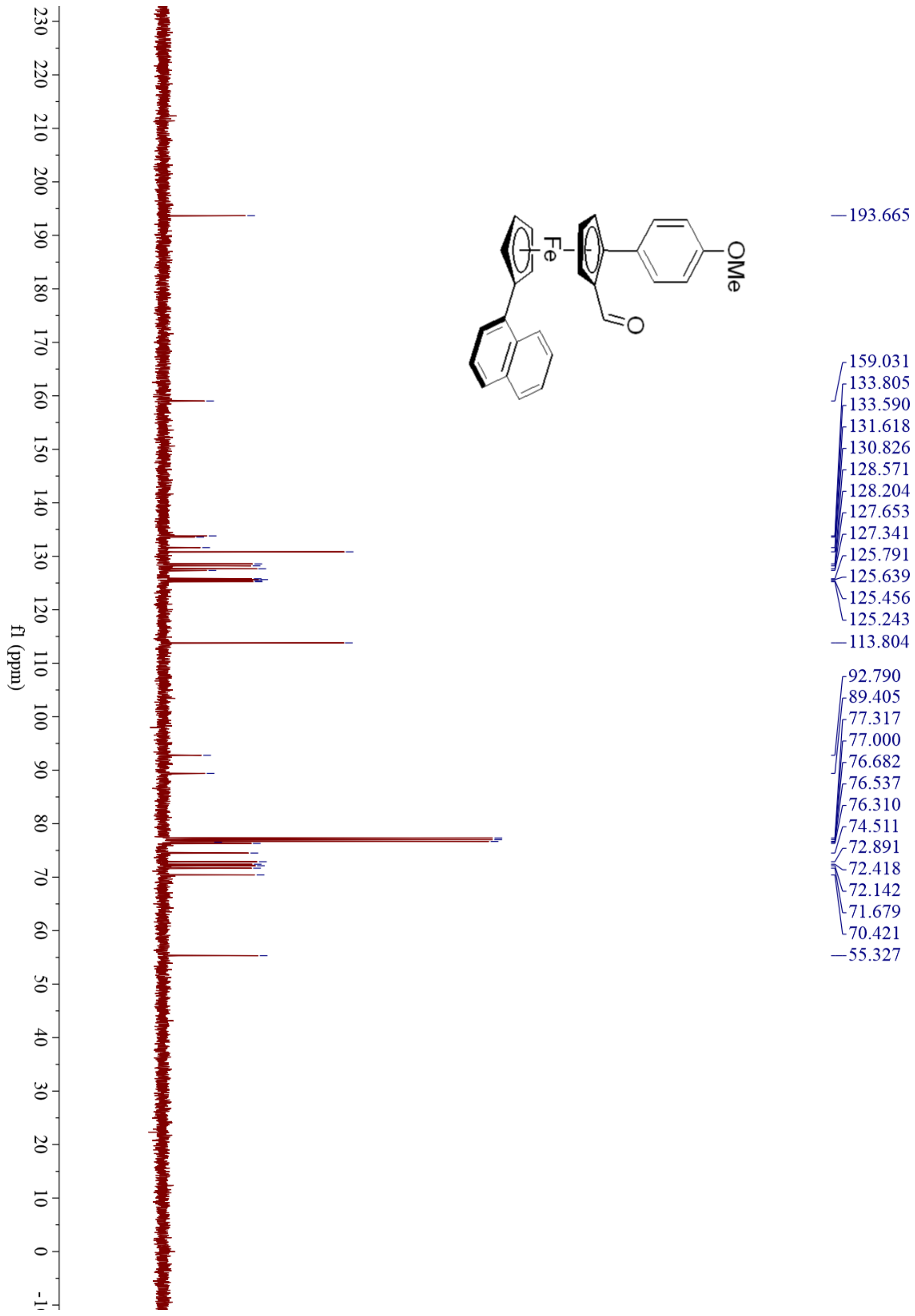

HPLC analysis of 3ma

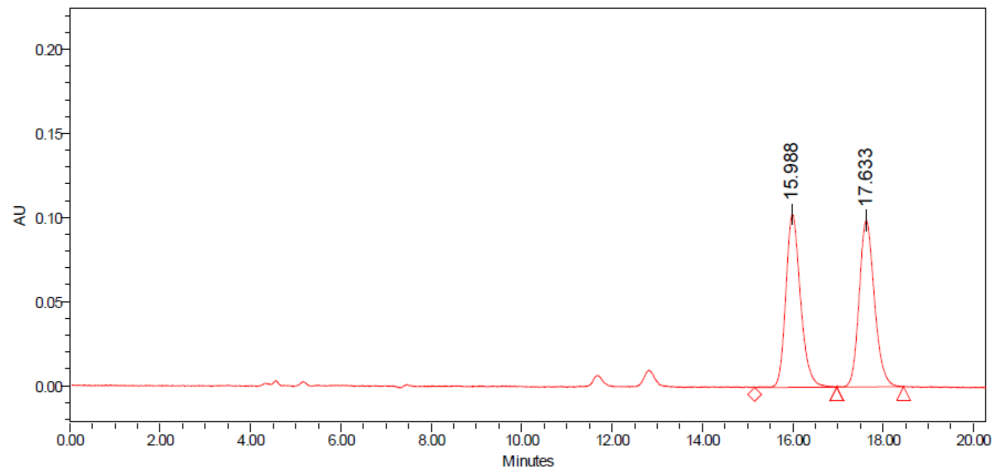

Peak Results

	SampleName	RT	Width (sec)	Height	Area	% Area
1	lcx-23-34-rac	15.988	109.000	102957	2327564	50.02
2	lcx-23-34-rac	17.633	88.800	99083	2325563	49.98

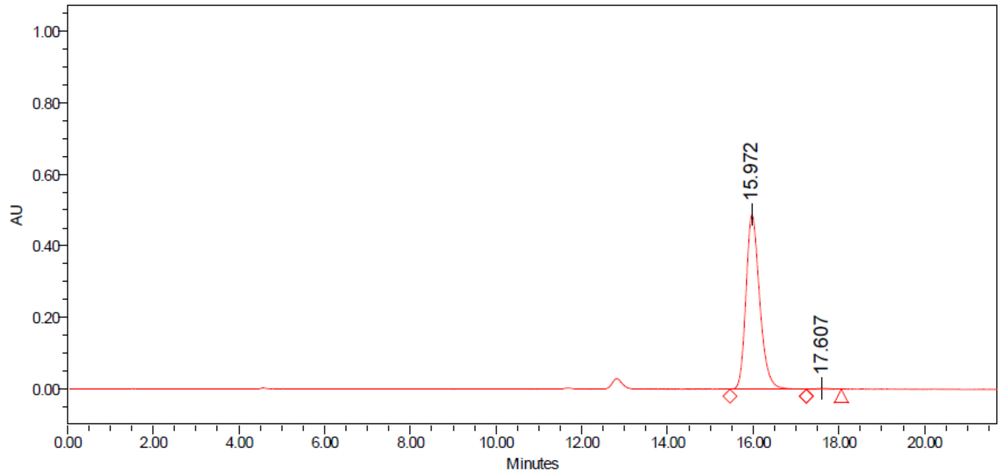

Peak Results

	SampleName	RT	Width (sec)	Height	Area	% Area
1	lcx-23-34-chiral	15.972	106.900	488143	10867764	99.46
2	lcx-23-34-chiral	17.607	49.000	2346	59335	0.54

^{1}H NMR spectra of 3na

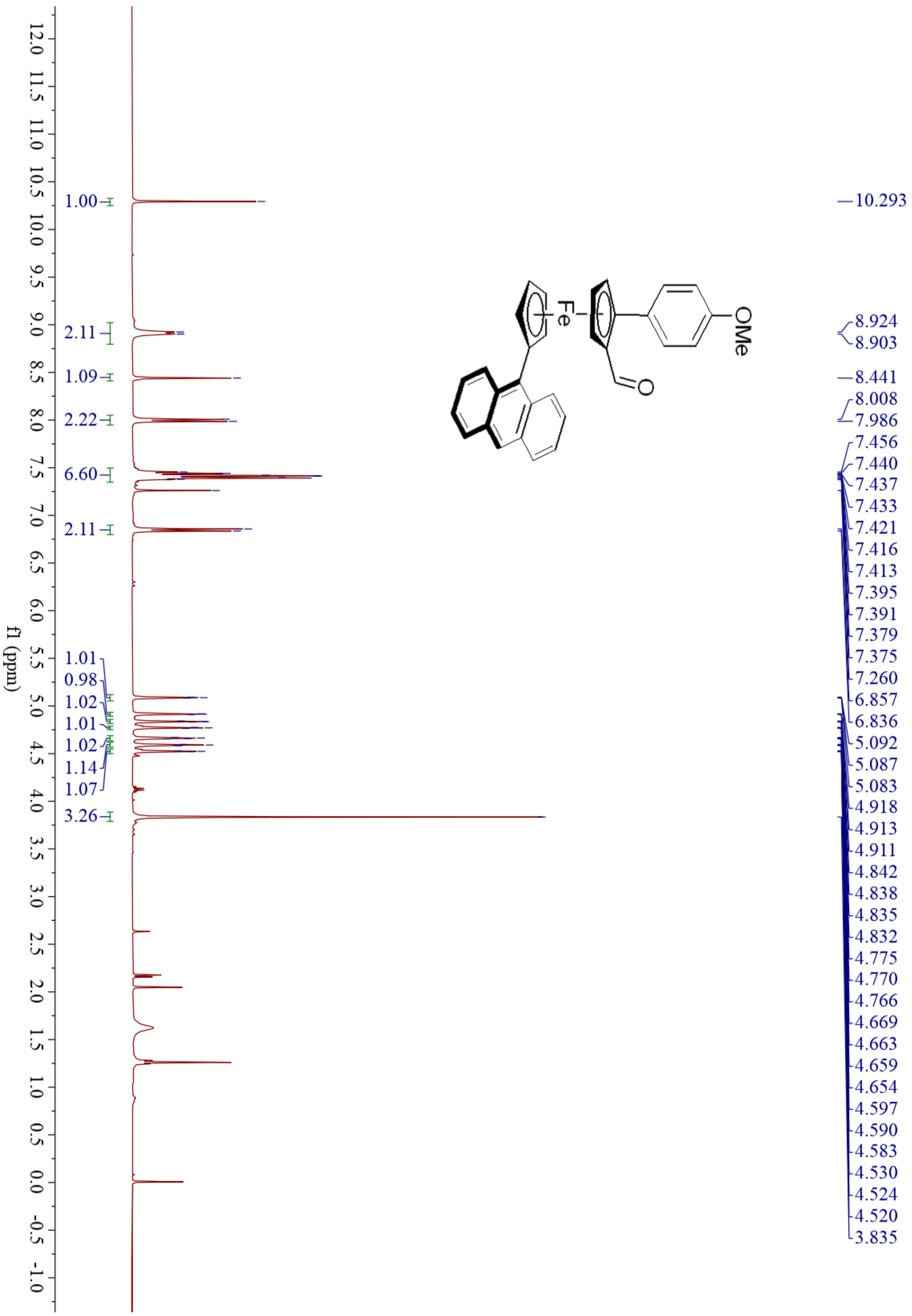

^{13}C NMR spectra of 3na

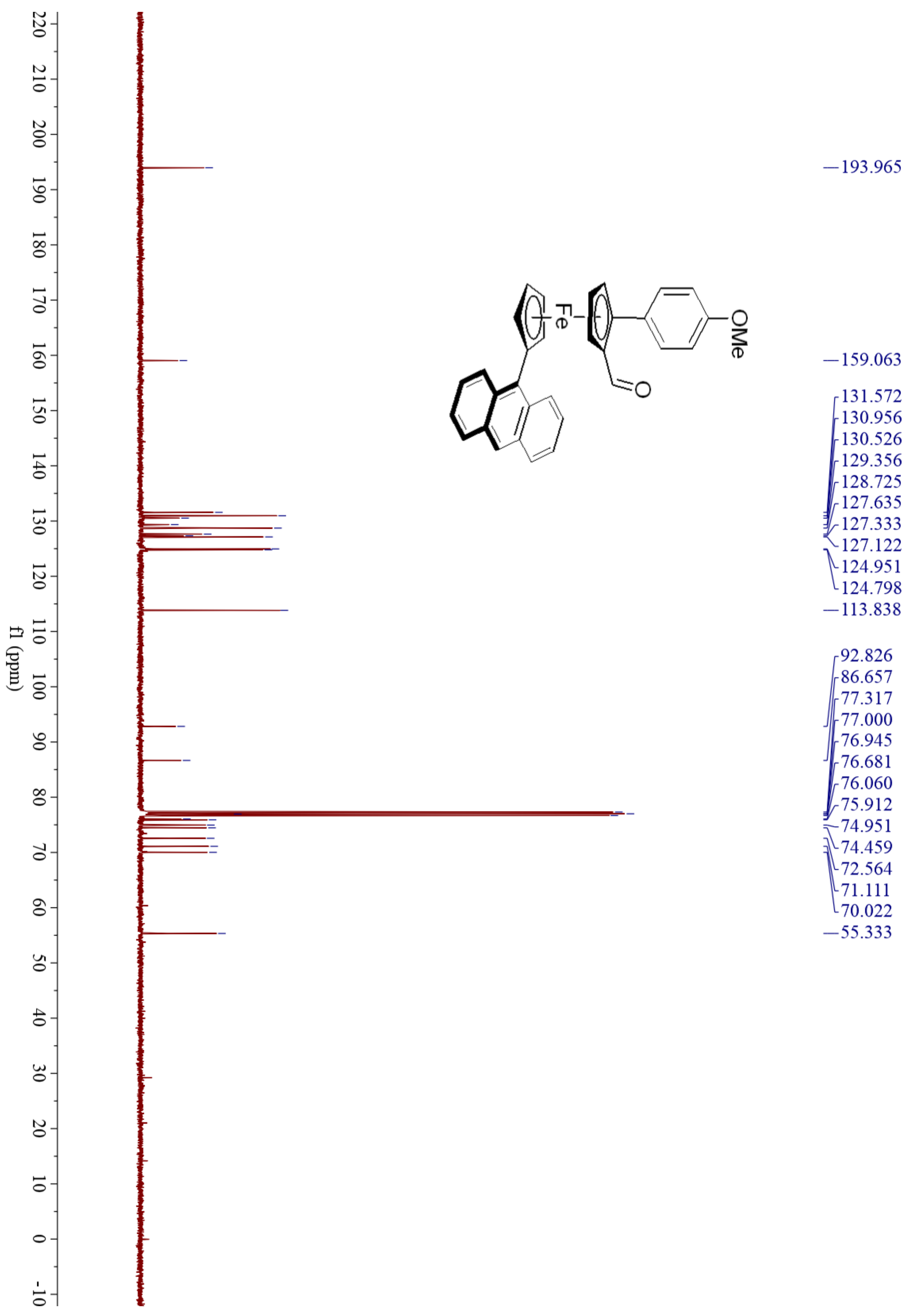

HPLC analysis of 3na

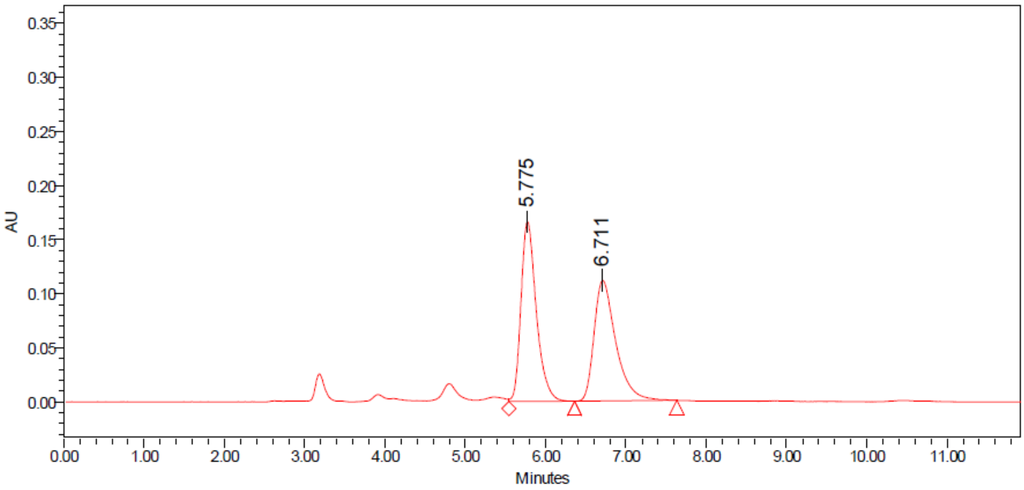

Peak Results

	SampleName	RT	Width (sec)	Height	Area	% Area
1	lcx-23-43-rac	5.775	49.000	166085	2198962	51.28
2	lcx-23-43-rac	6.711	76.400	111415	2089039	48.72

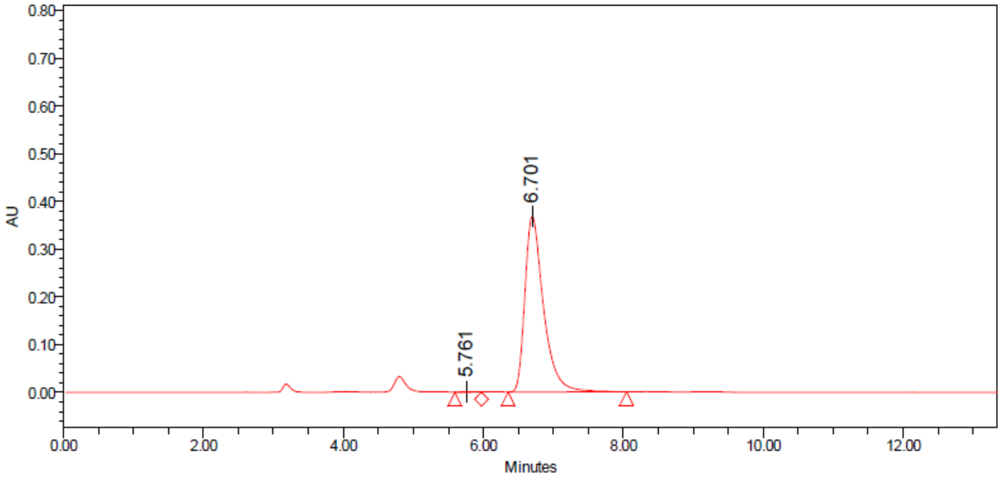

Peak Results

	SampleName	RT	Width (sec)	Height	Area	% Area
1	lcx-23-43-CHIRAL	5.761	22.800	1207	14265	0.21
2	lcx-23-43-CHIRAL	6.701	101.500	367725	6805010	99.79

^{1}H NMR spectra of 3oa

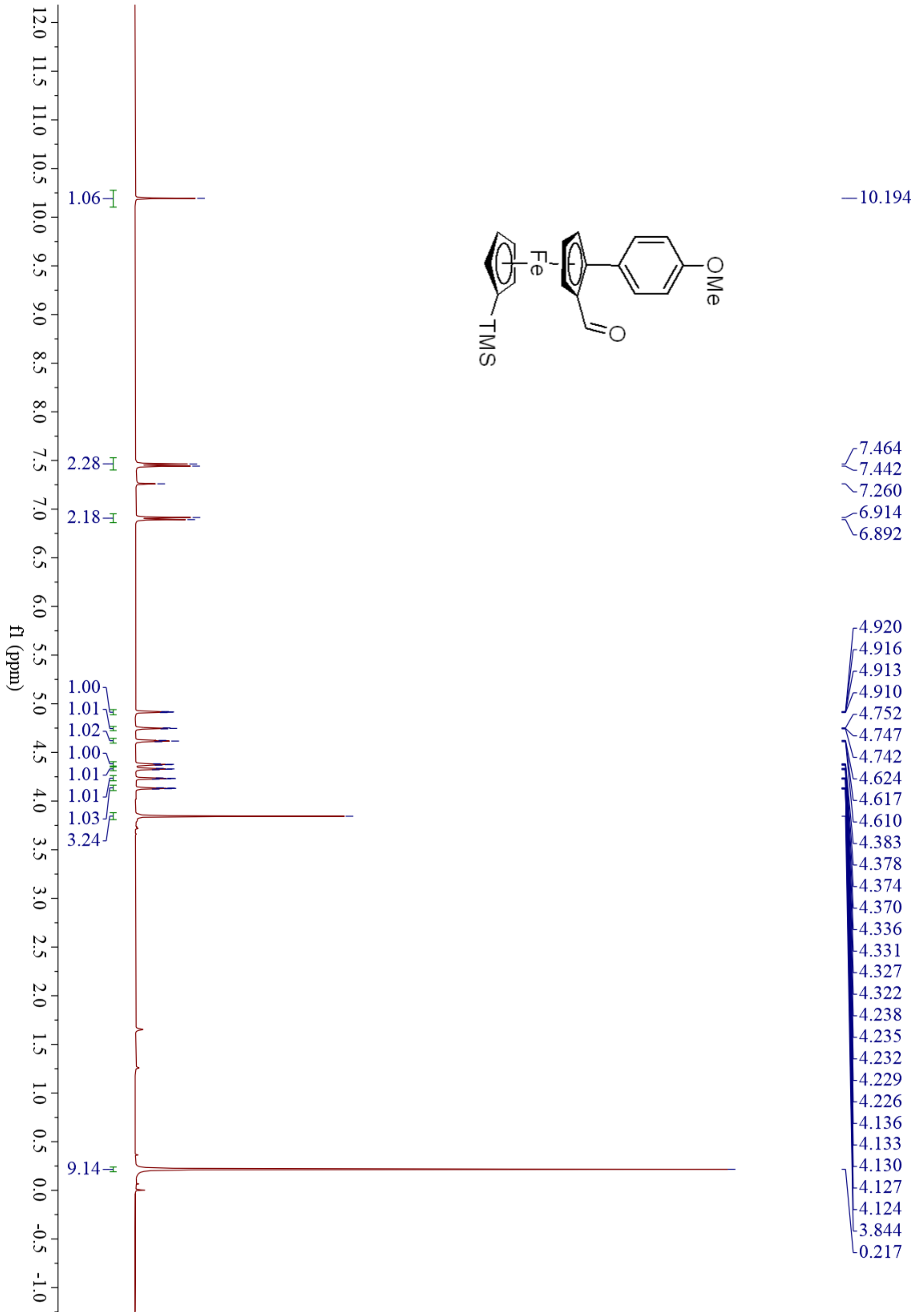

^{13}C NMR spectra of 3oa

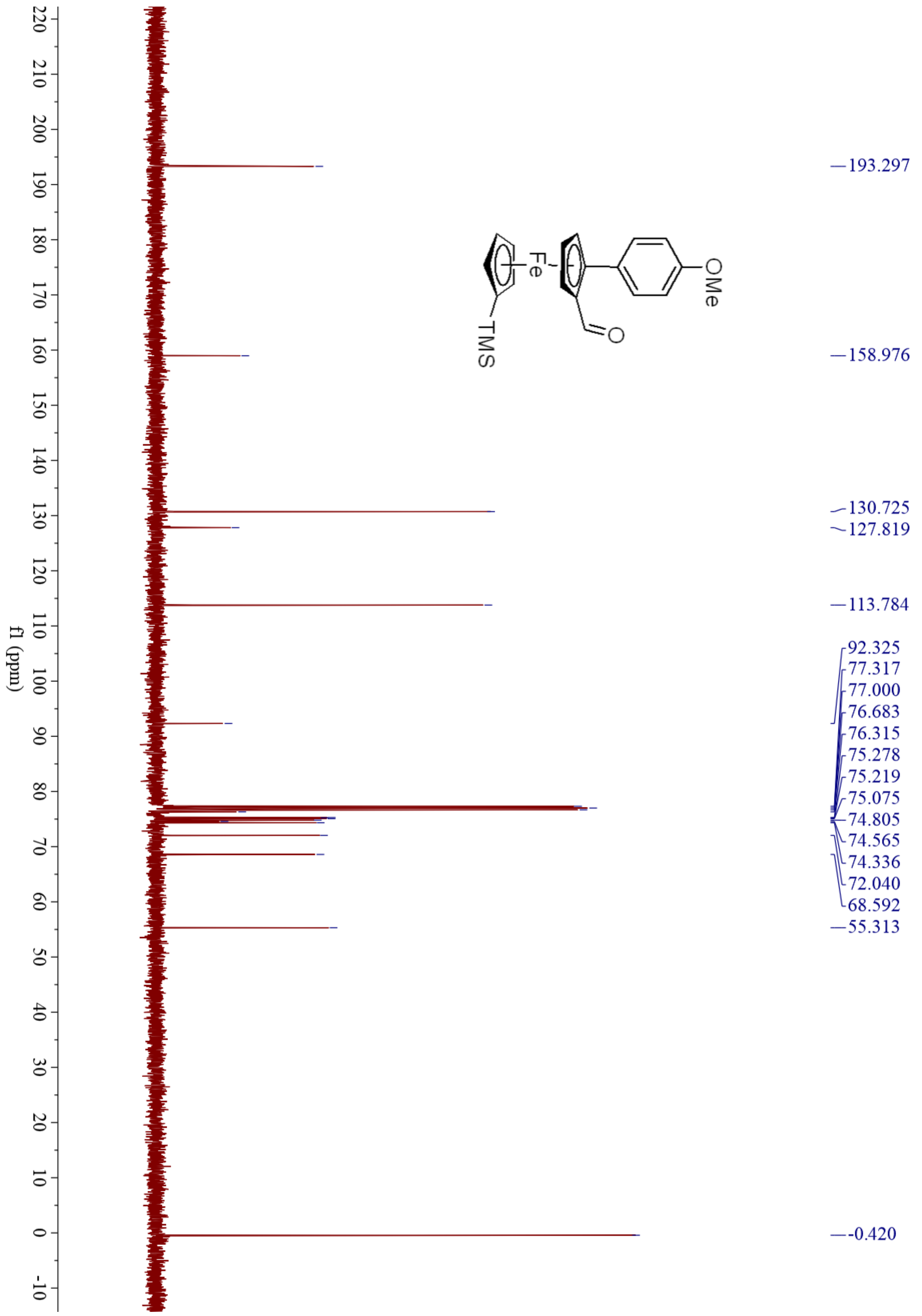

HPLC analysis of 3oa

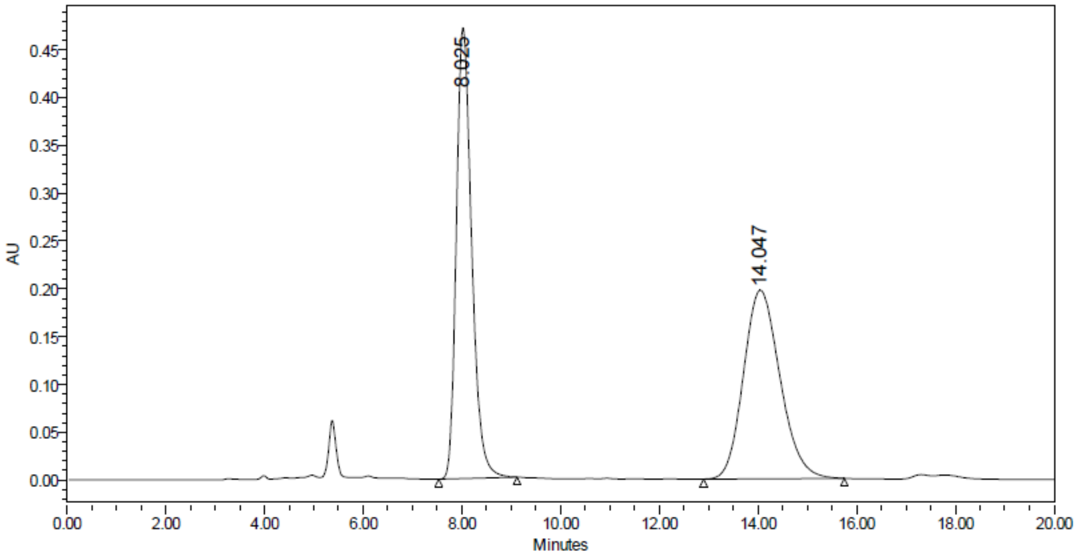

	RT	Area	% Area	Height
1	8.025	10171429	50.07	472235
2	14.047	10142611	49.93	197191

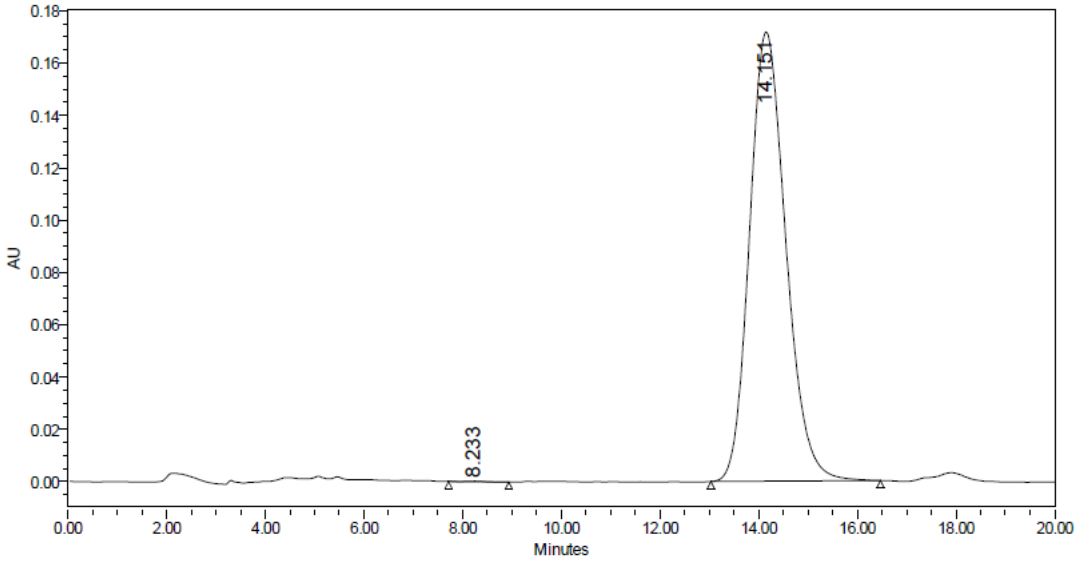

	RT	Area	% Area	Height
1	8.233	4156	0.05	192
2	14.151	8787166	99.95	171692

^{1}H NMR spectra of 4a

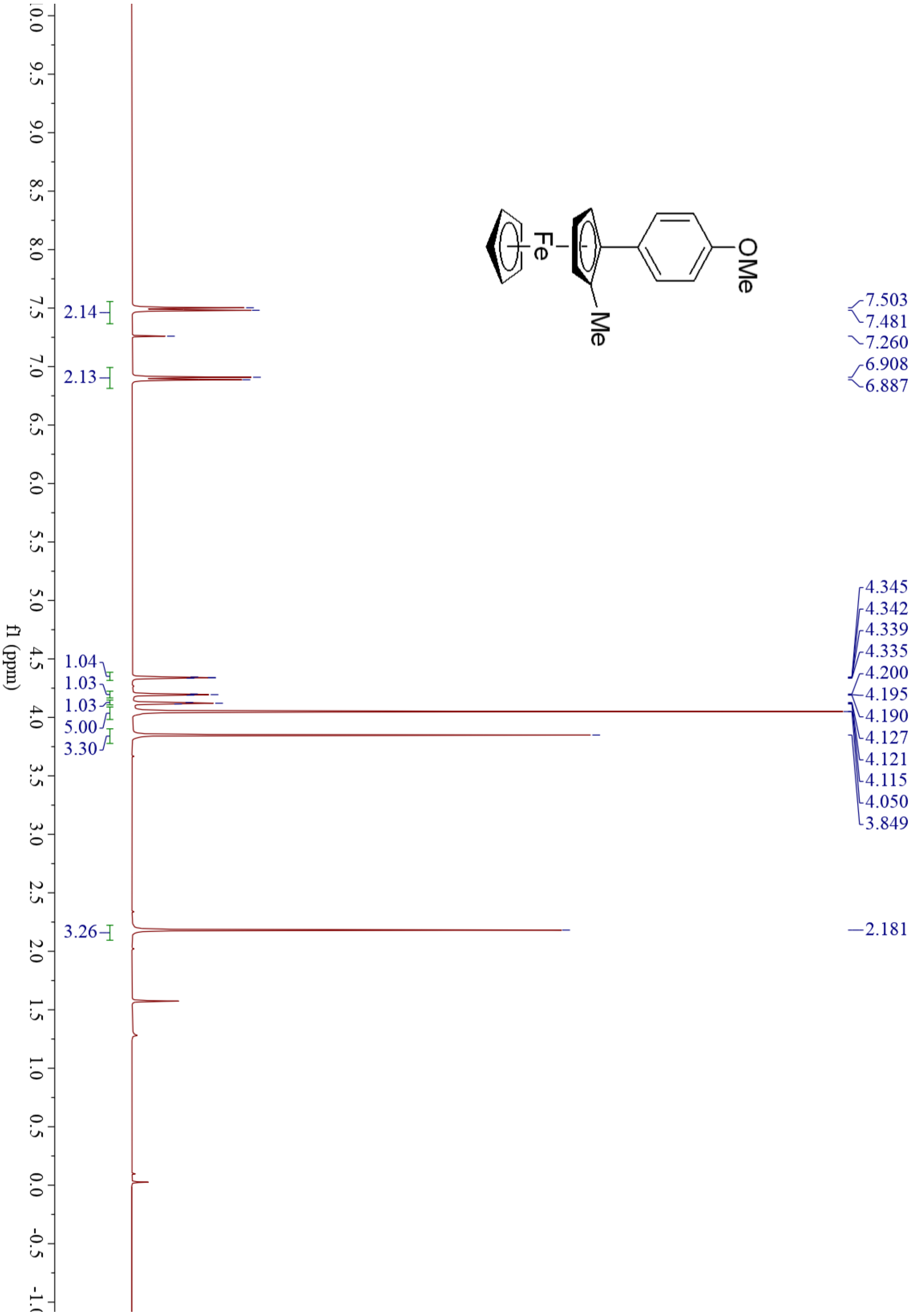

^{13}C NMR spectra of 4a

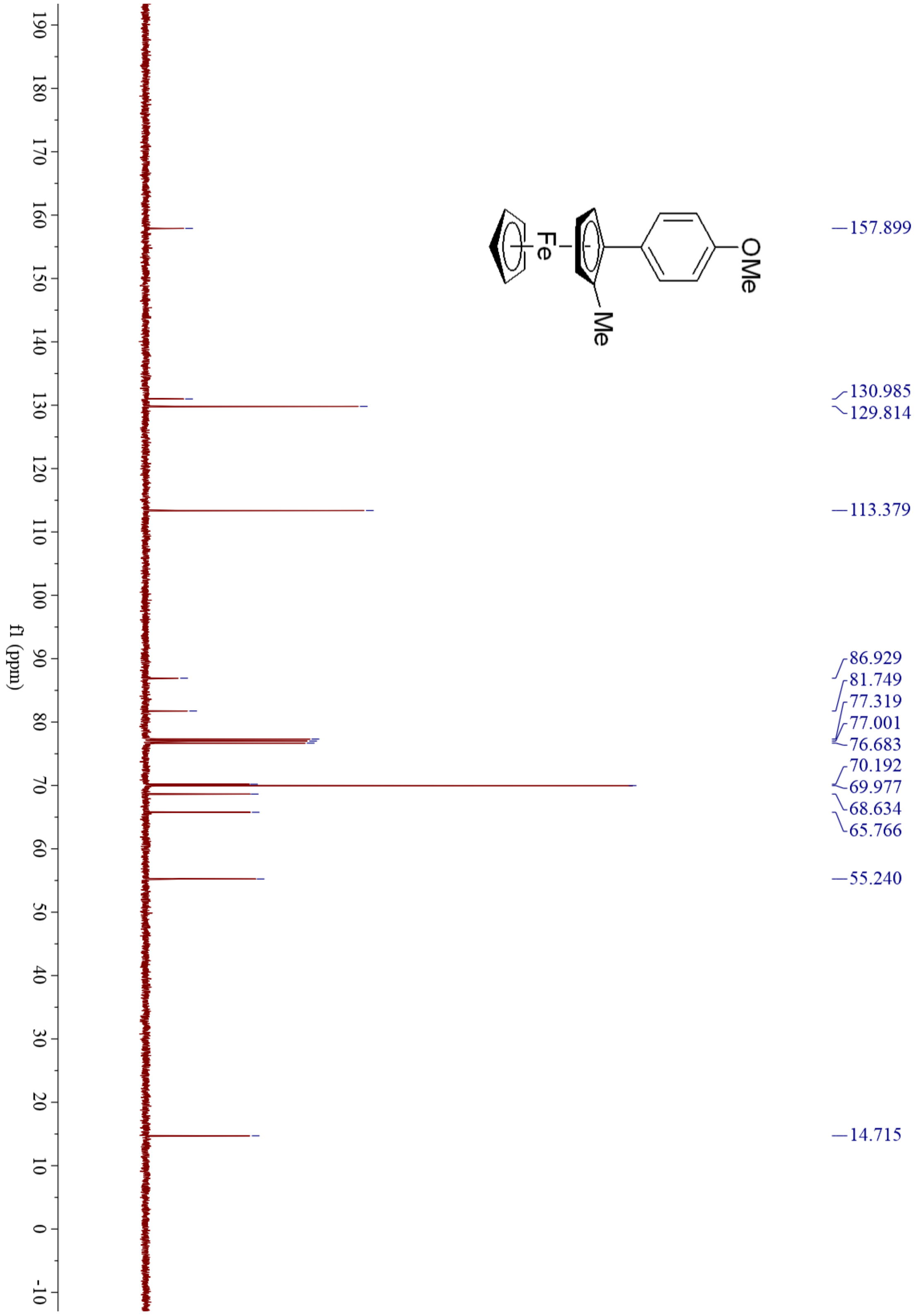

HPLC analysis of 4a

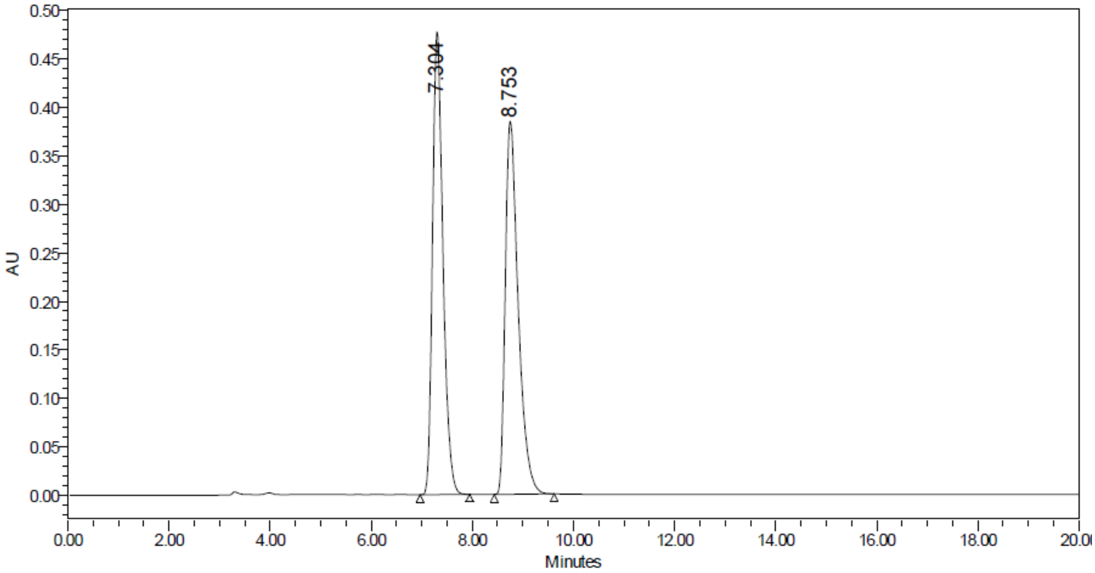

	RT	Area	% Area	Height
1	7.304	6840883	49.97	477661
2	8.753	6848415	50.03	385118

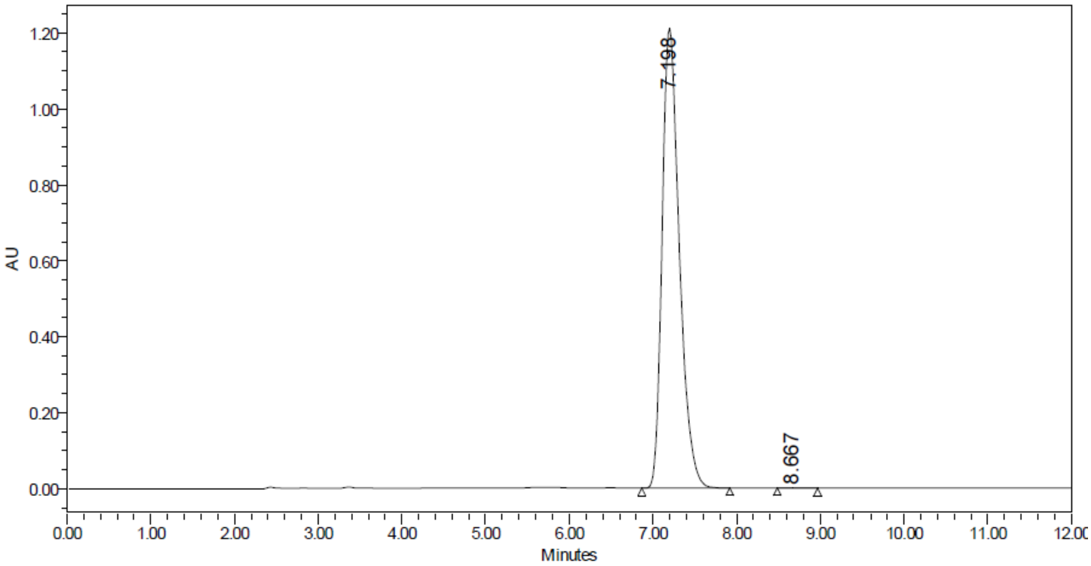

	RT	Area	% Area	Height
1	7.198	17283942	99.99	1210064
2	8.667	1782	0.01	-119

^{1}H NMR spectra of 4b

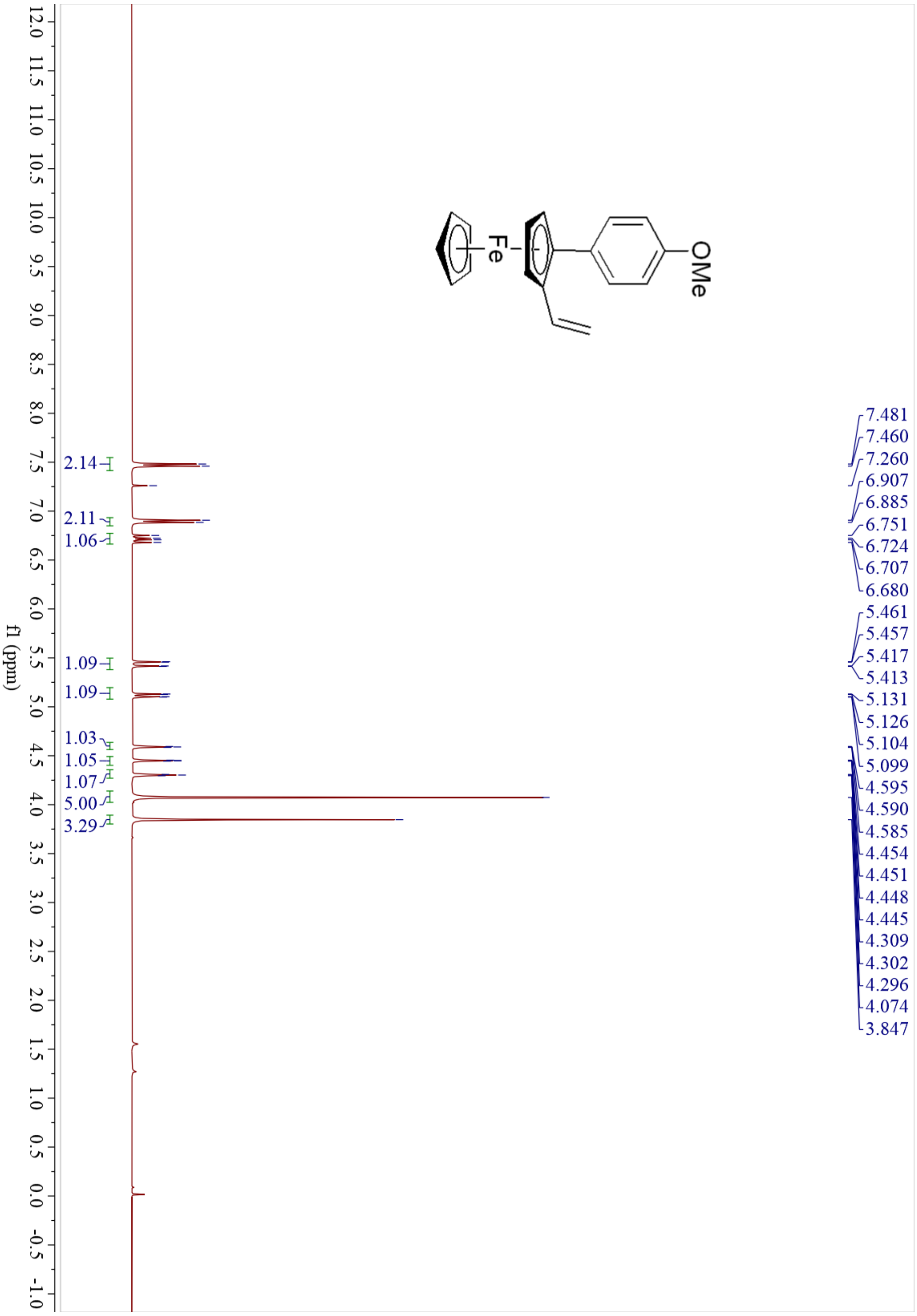

^{13}C NMR spectra of 4b

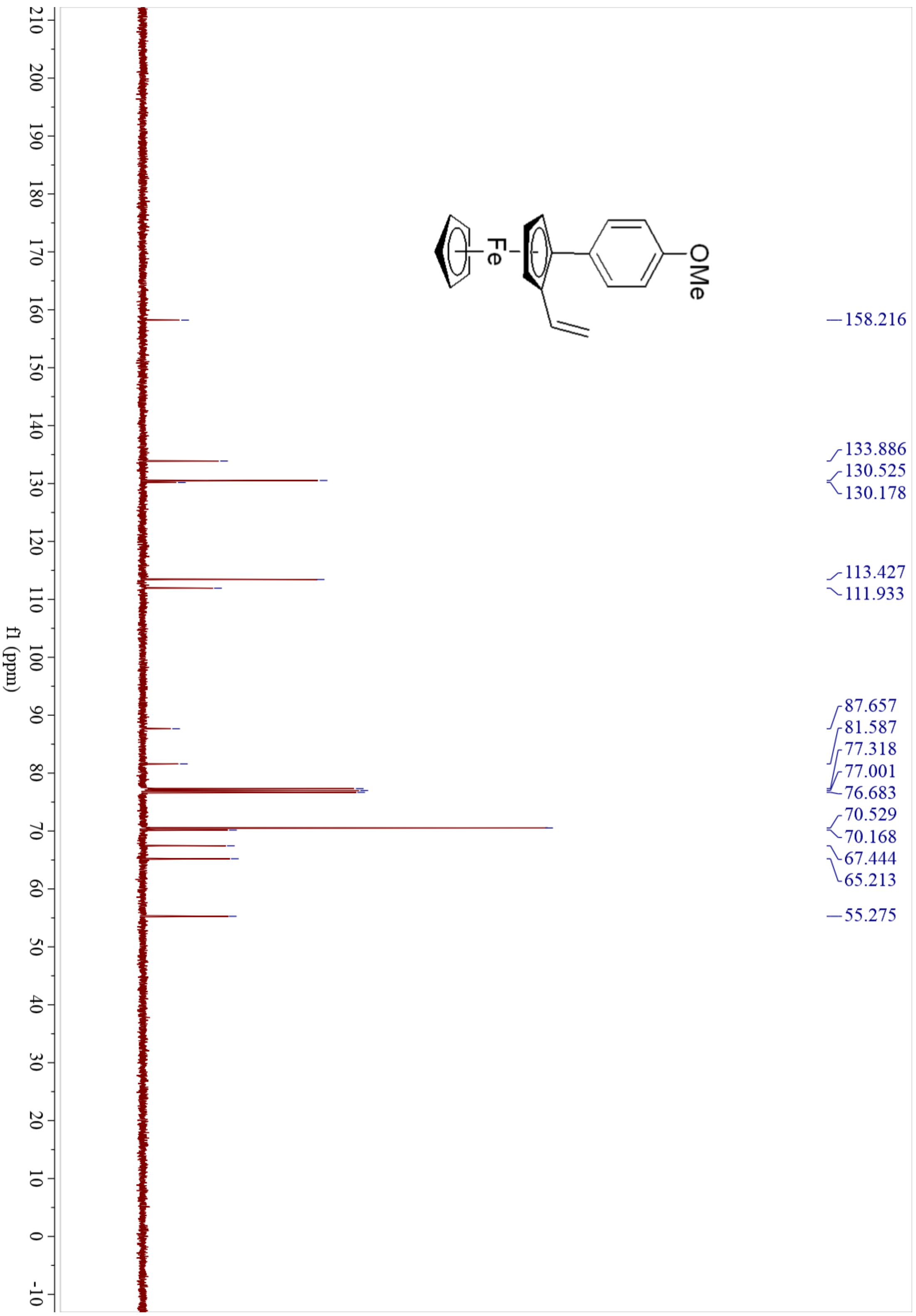

HPLC analysis of 4b

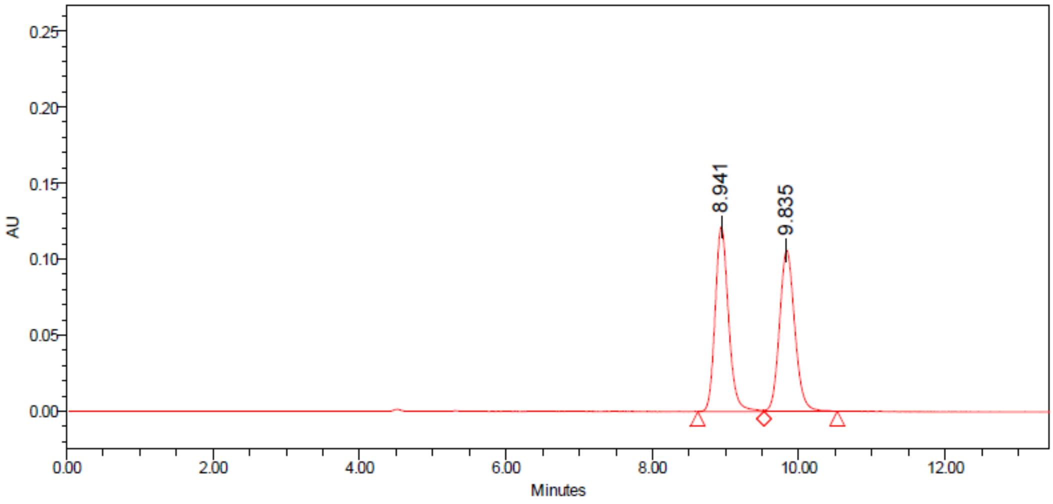

Peak Results

	SampleName	RT	Width (sec)	Height	Area	% Area
1	lcx-22-62-rac	8.941	54.000	121496	1538679	50.10
2	lcx-22-62-rac	9.835	60.200	105968	1532758	49.90

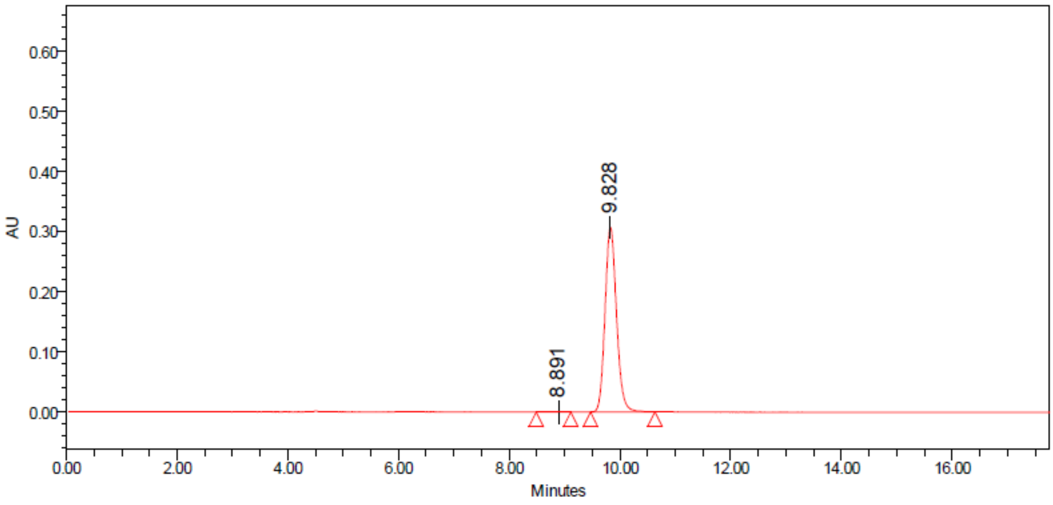

Peak Results

	SampleName	RT	Width (sec)	Height	Area	% Area
1	lcx-22-62-chiral	8.891	37.400	129	1960	0.04
2	lcx-22-62-chiral	9.828	70.000	307187	4414985	99.96

¹H NMR spectra of 4c

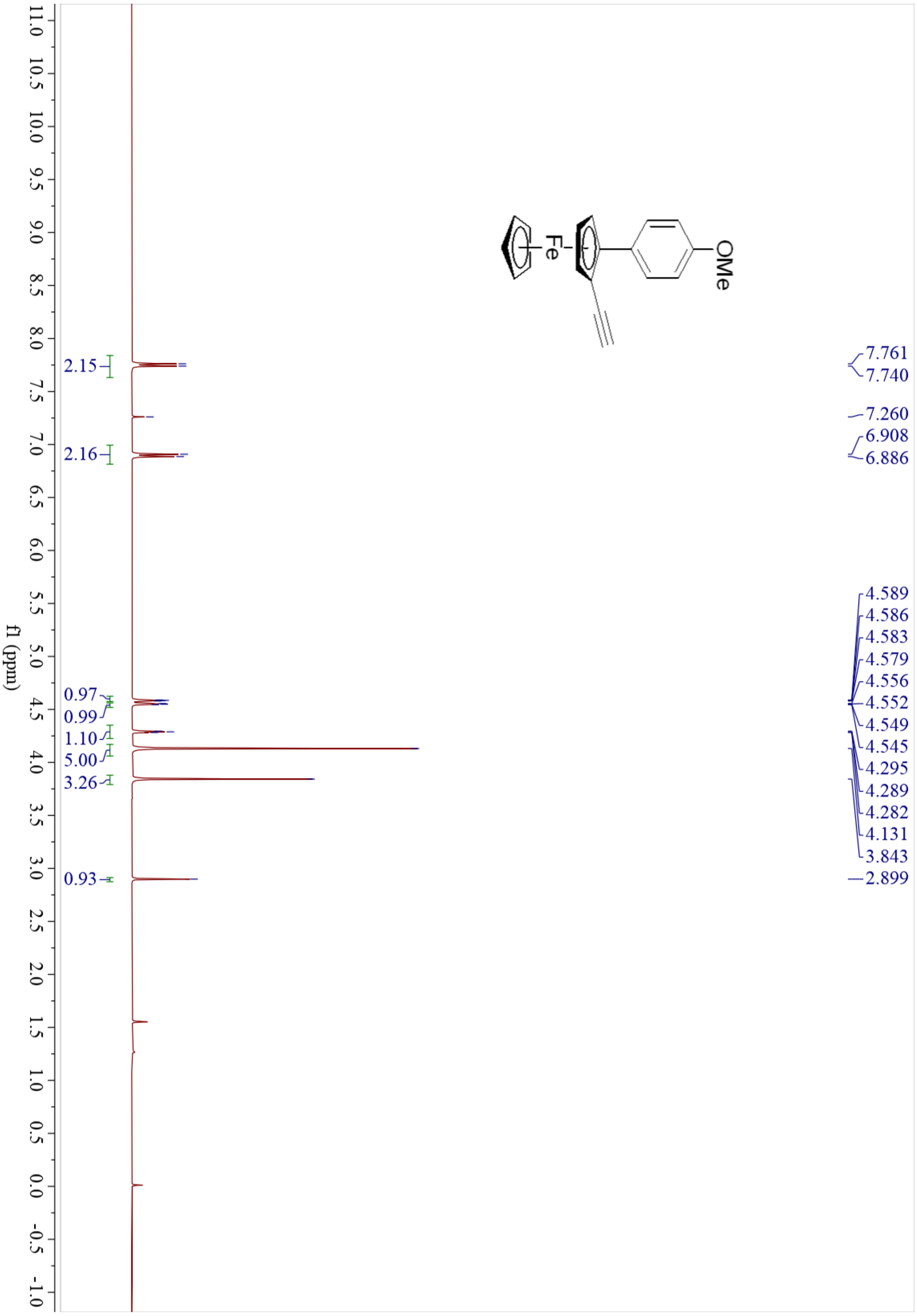

^{13}C NMR spectra of 4c

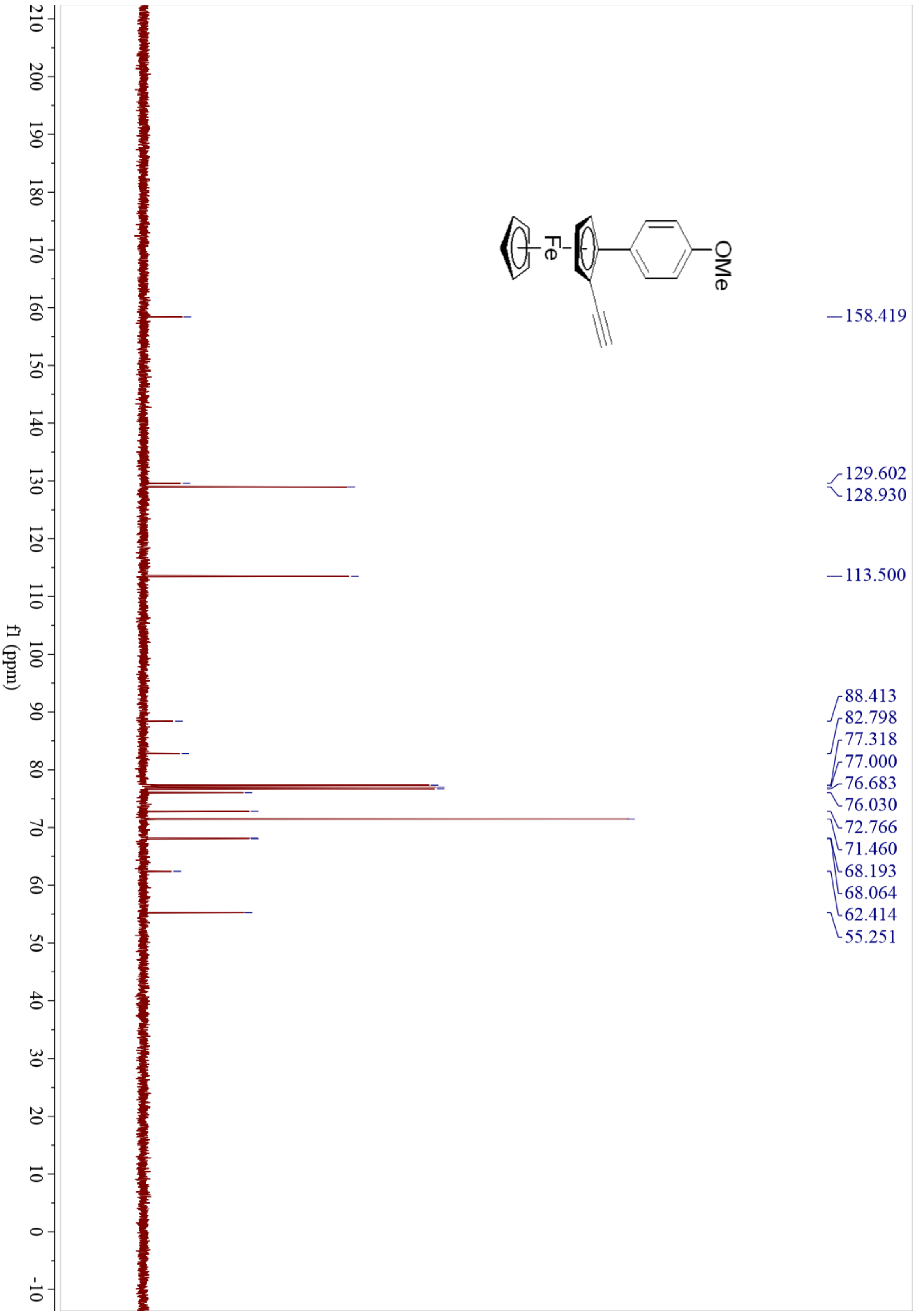

HPLC analysis of 4c

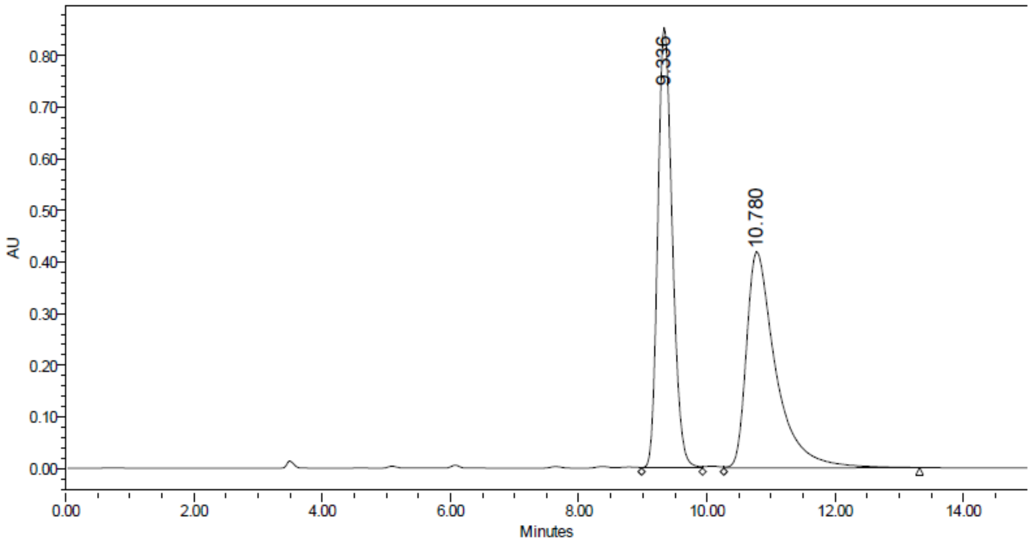

	RT	Area	% Area	Height
1	9.336	13378538	50.12	853734
2	10.780	13313126	49.88	418641

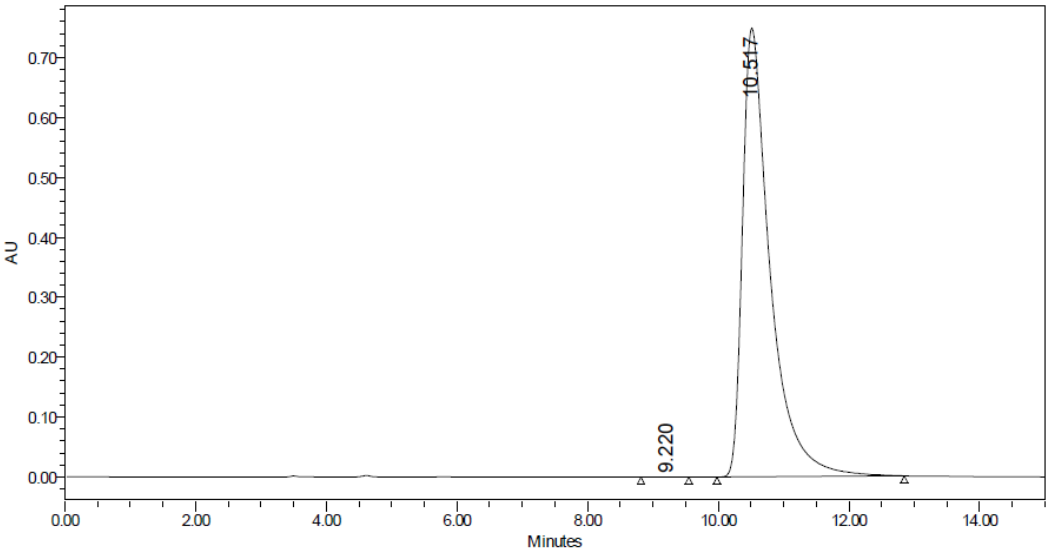

	RT	Area	% Area	Height
1	9.220	2737	0.01	181
2	10.517	21951572	99.99	749276

^{1}H NMR spectra of 4d

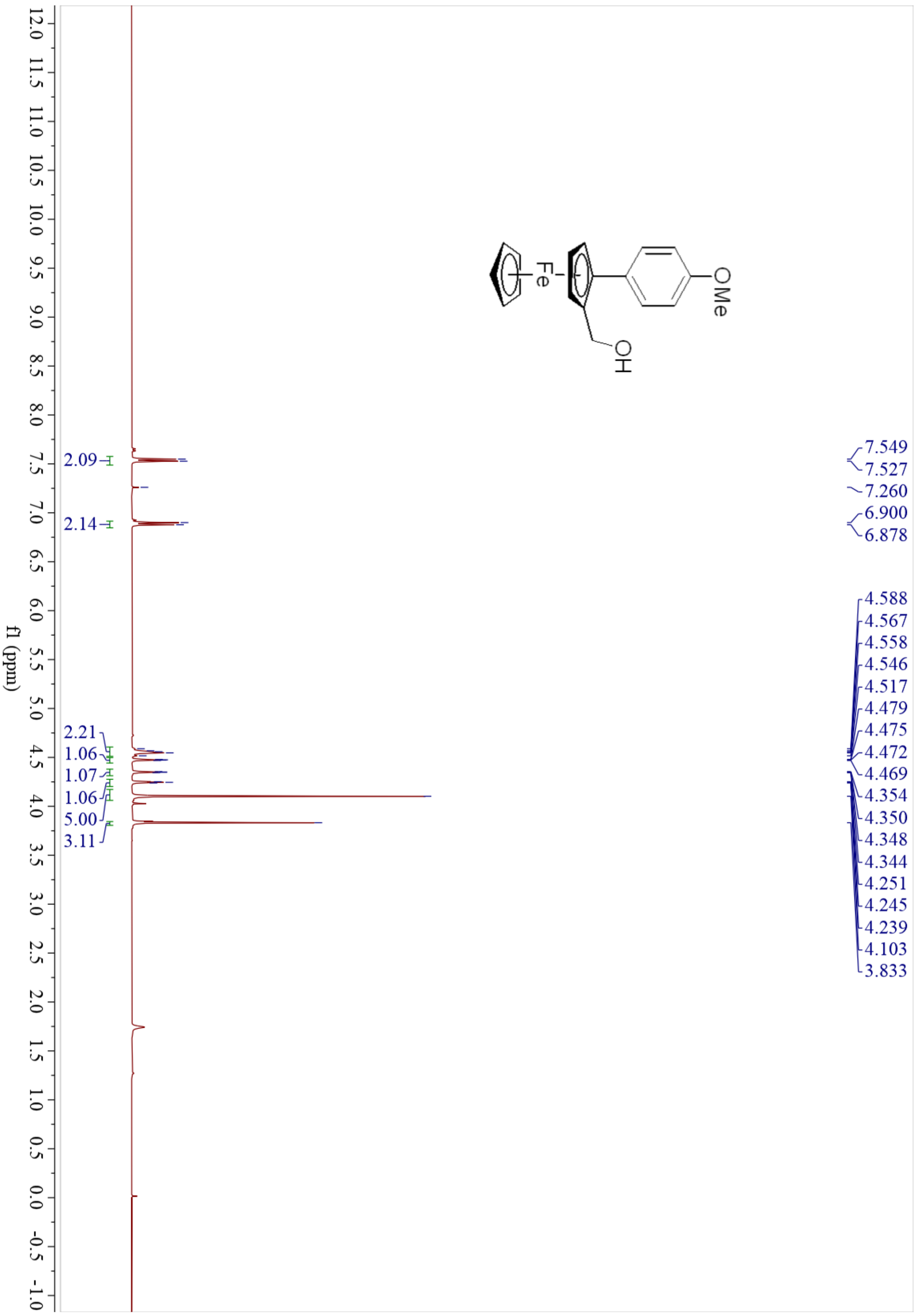

^{13}C NMR spectra of 4d

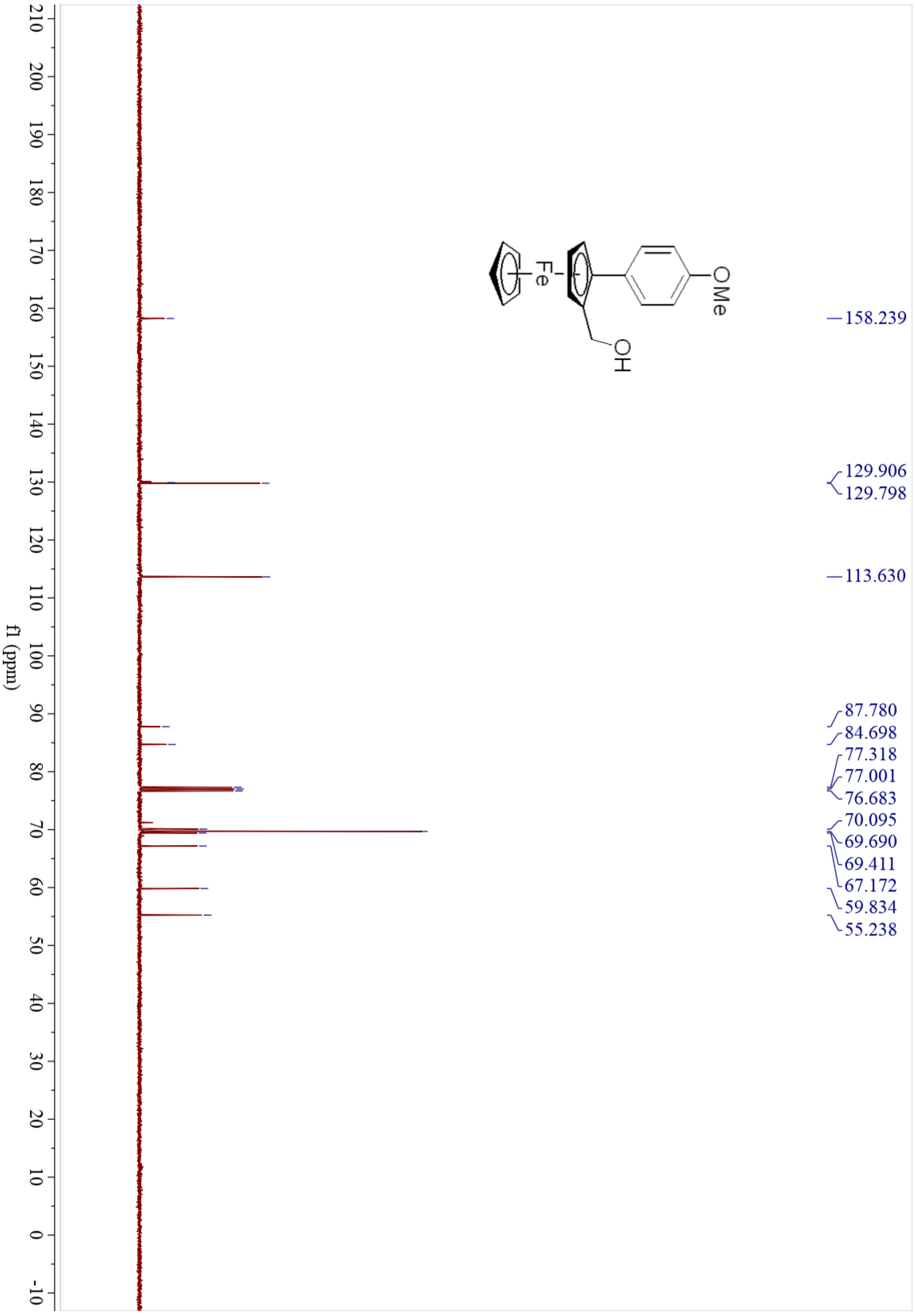

HPLC analysis of 4d

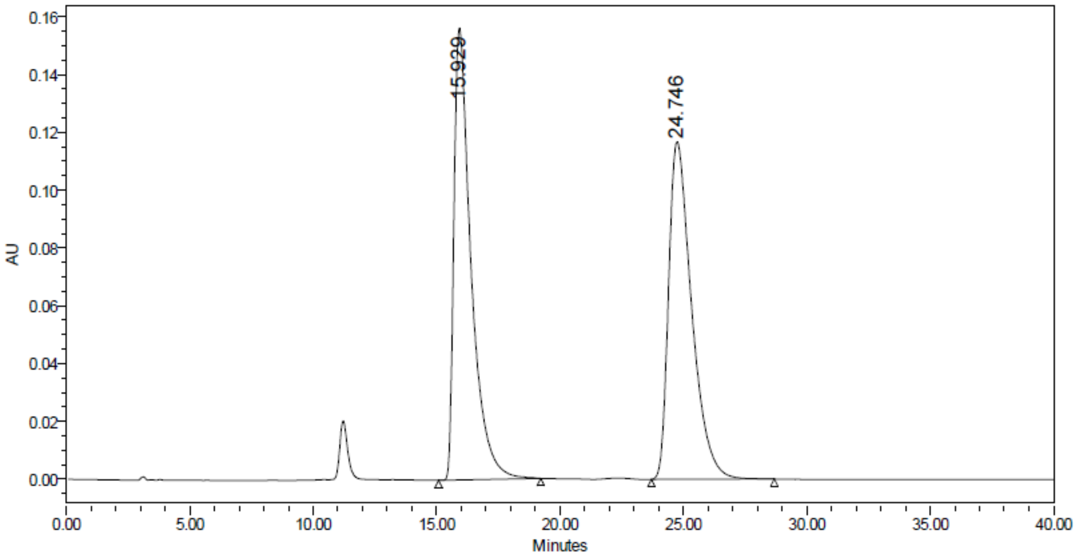

	RT	Area	% Area	Height
1	15.929	7528433	49.87	156300
2	24.746	7569033	50.13	116750

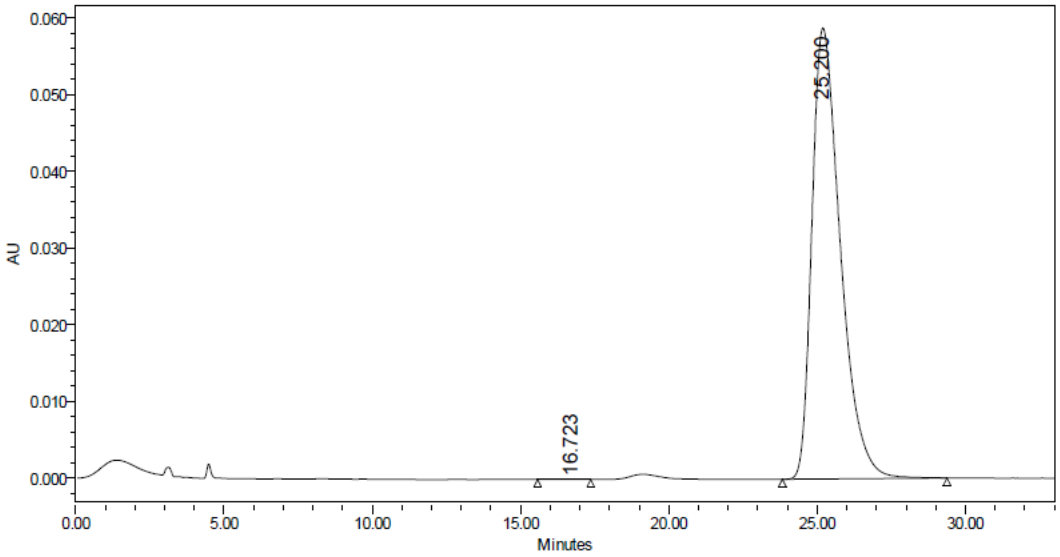

	RT	Area	% Area	Height
1	16.723	1767	0.05	34
2	25.200	3921577	99.95	58768

^{1}H NMR spectra of 4e

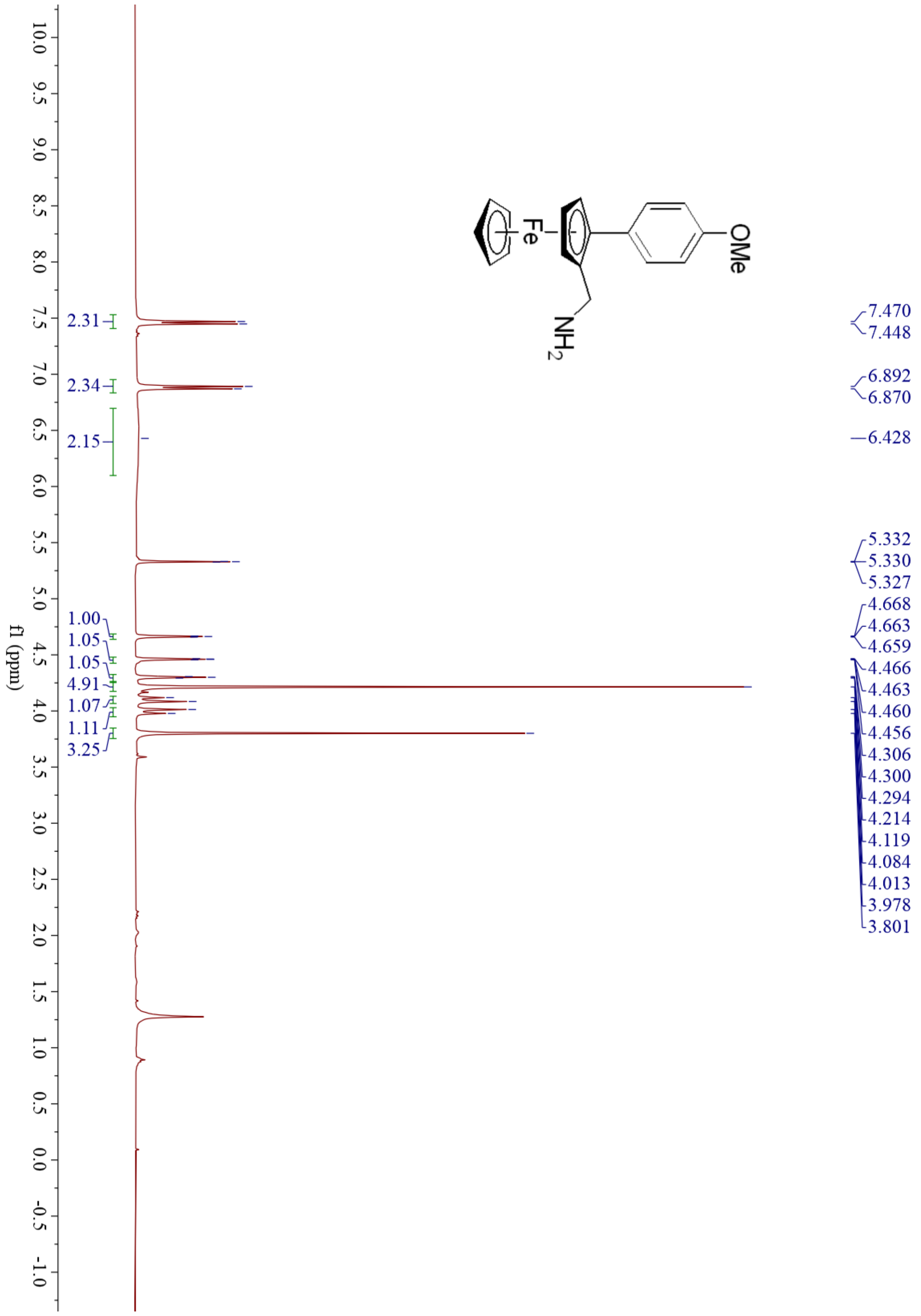

^{13}C NMR spectra of 4e

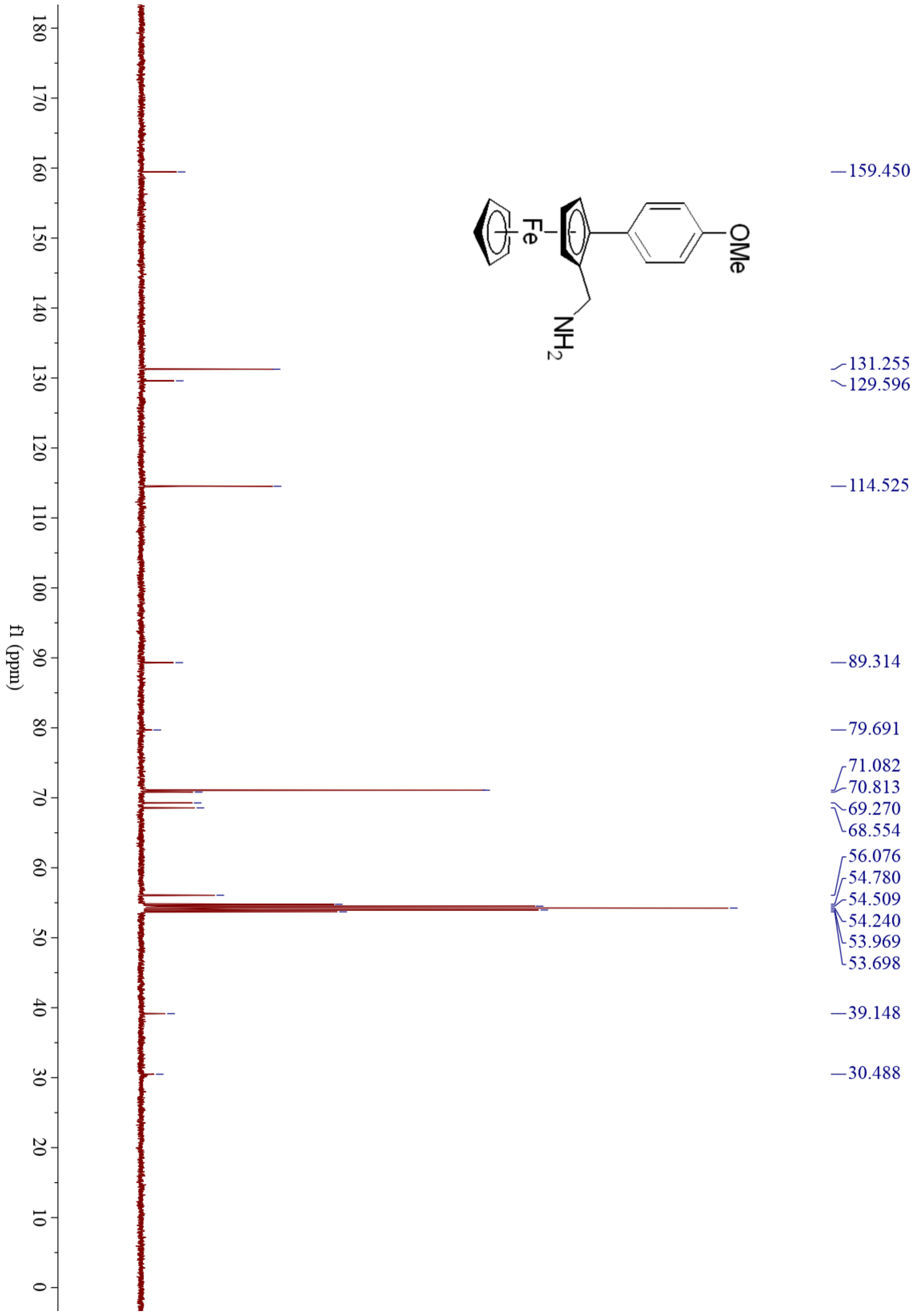

HPLC analysis of 4e

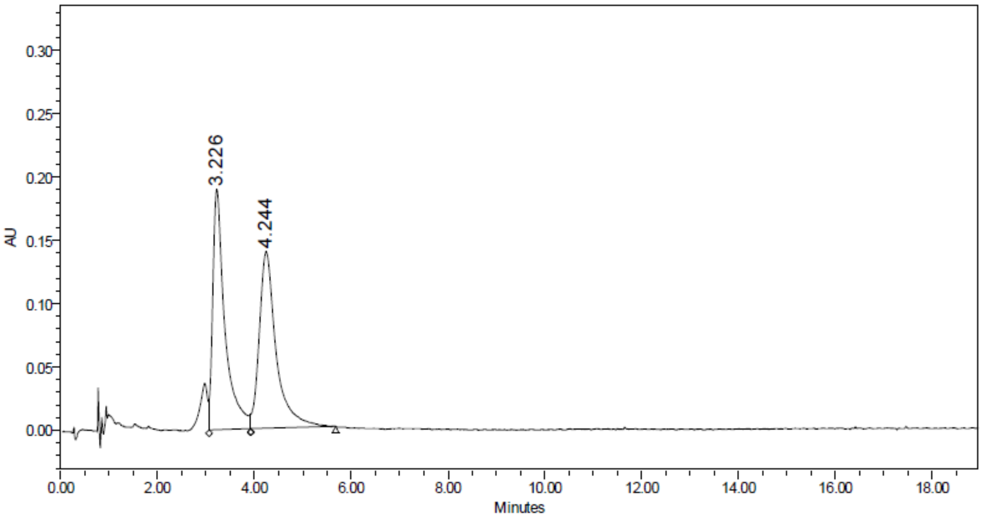

	RT	Peak Type	Height	Width (sec)	Area	% Area
1	3.226	Unknown	189899	51.650	3319191	49.42
2	4.244	Unknown	139318	105.200	3397682	50.58

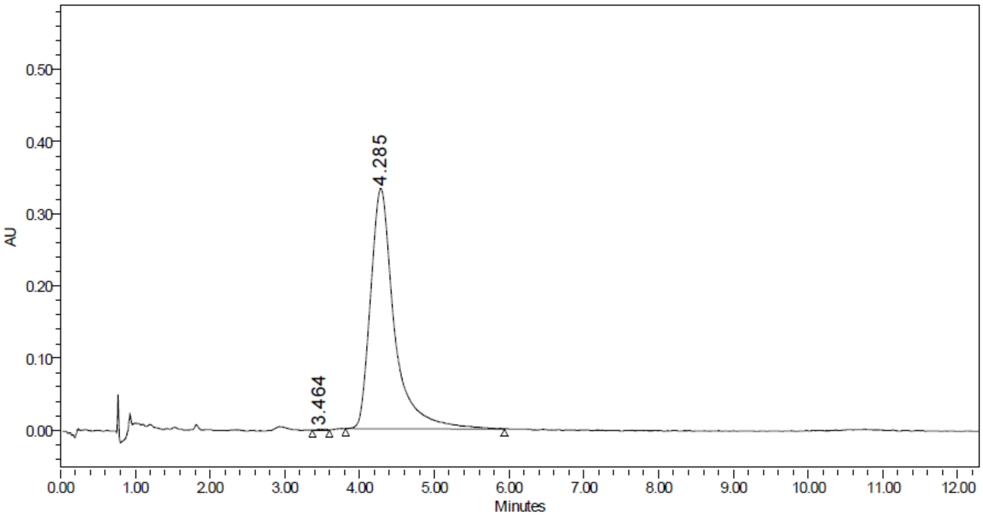

	RT	Peak Type	Height	Width (sec)	Area	% Area
1	3.464	Unknown	1084	13.550	7281	0.10
2	4.285	Unknown	332842	127.450	7417843	99.90

^{1}H NMR spectra of 4f

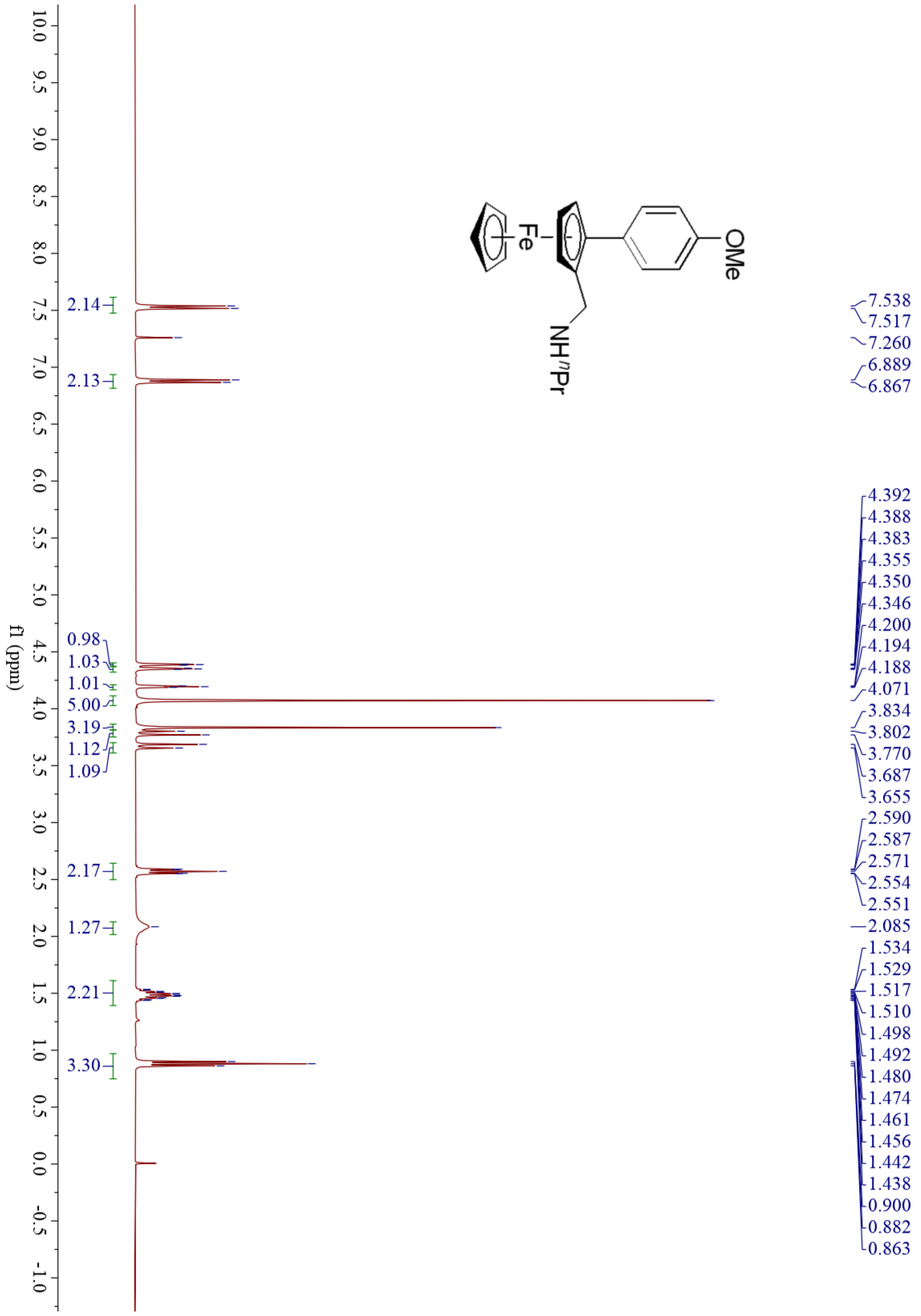

^{13}C NMR spectra of 4f

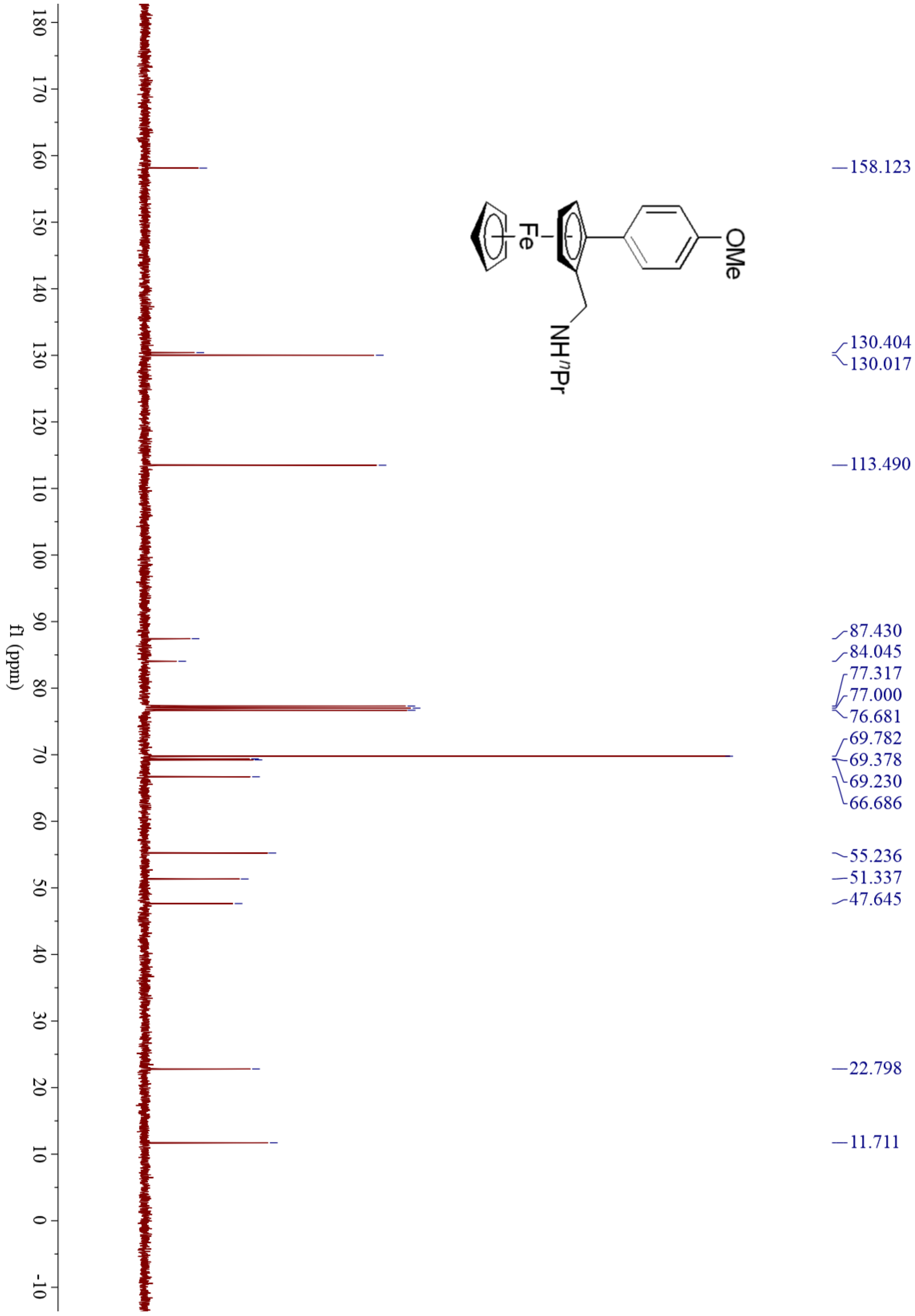

HPLC analysis of 4f

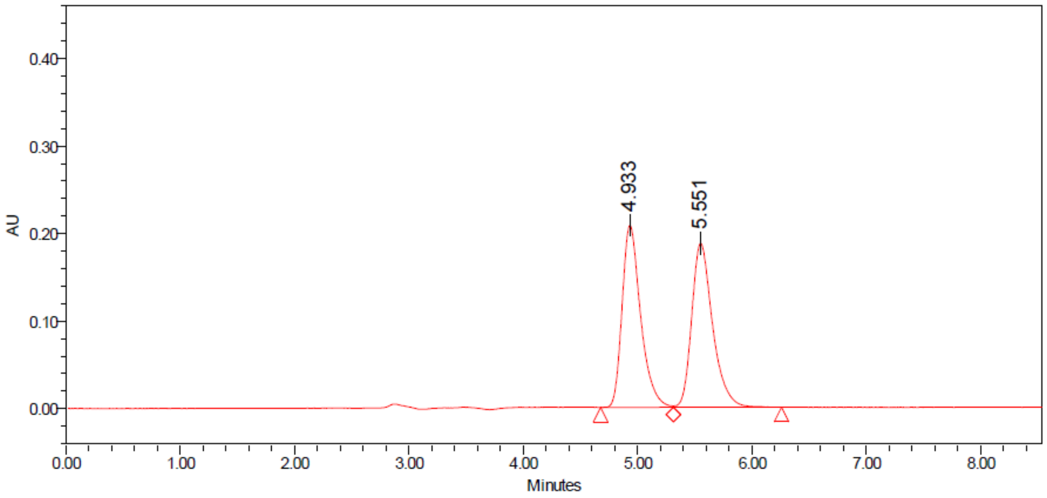

Peak Results

	SampleName	RT	Width (sec)	Height	Area	% Area
1	LCX-23-PrNH2-RAC	4.933	38.200	208061	2343764	49.59
2	LCX-23-PrNH2-RAC	5.551	56.700	187486	2382799	50.41

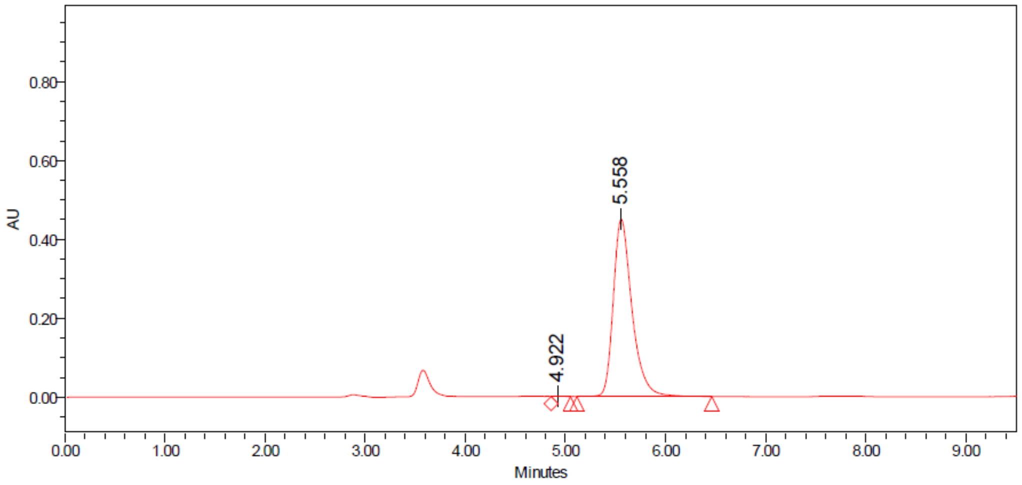

Peak Results

	SampleName	RT	Width (sec)	Height	Area	% Area
1	LCX-23-PrNH2-CHIRAL	4.922	11.500	196	1052	0.02
2	LCX-23-PrNH2-CHIRAL	5.558	80.800	449764	5837207	99.98

^{1}H NMR spectra of 4g

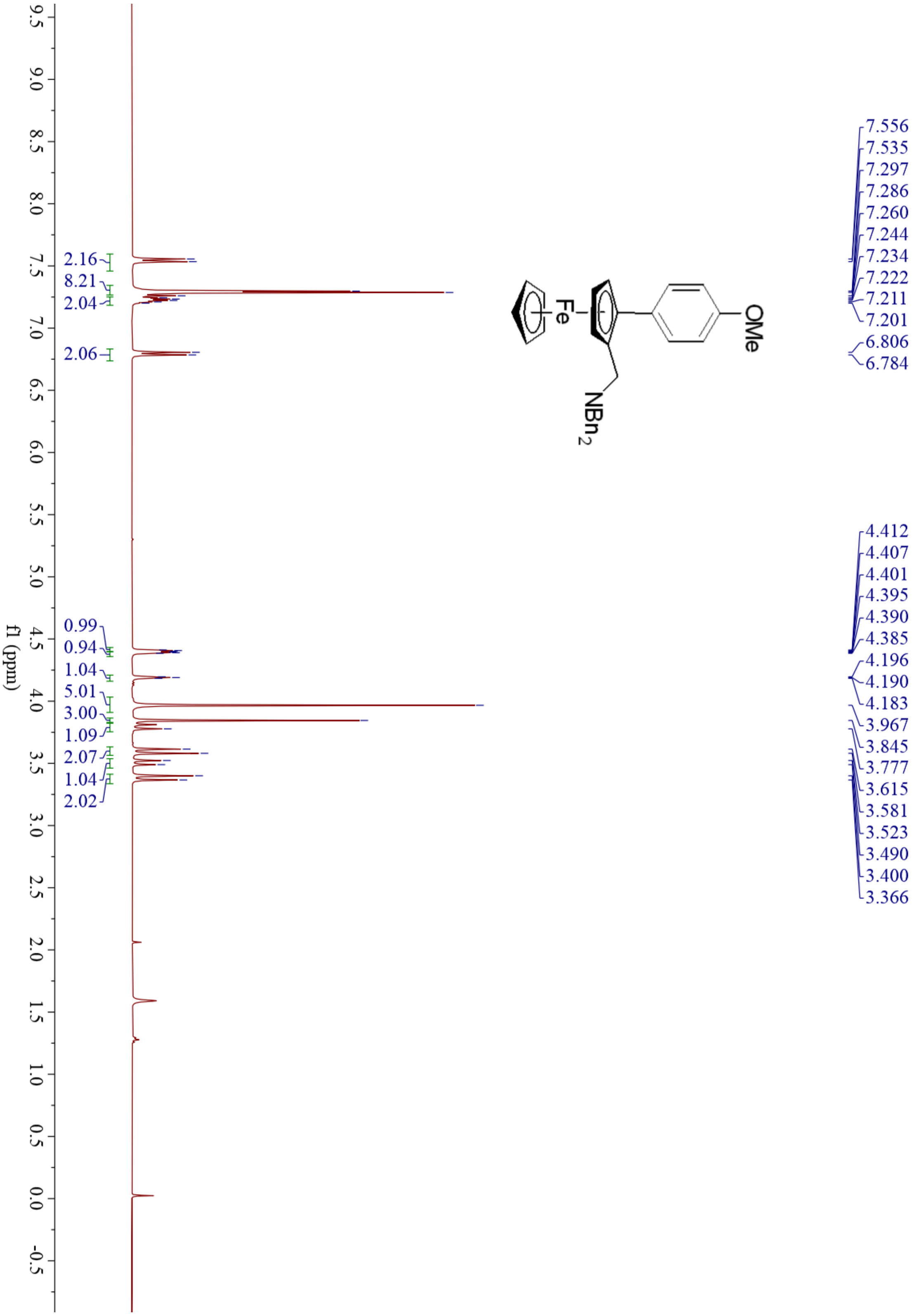

^{13}C NMR spectra of 4g

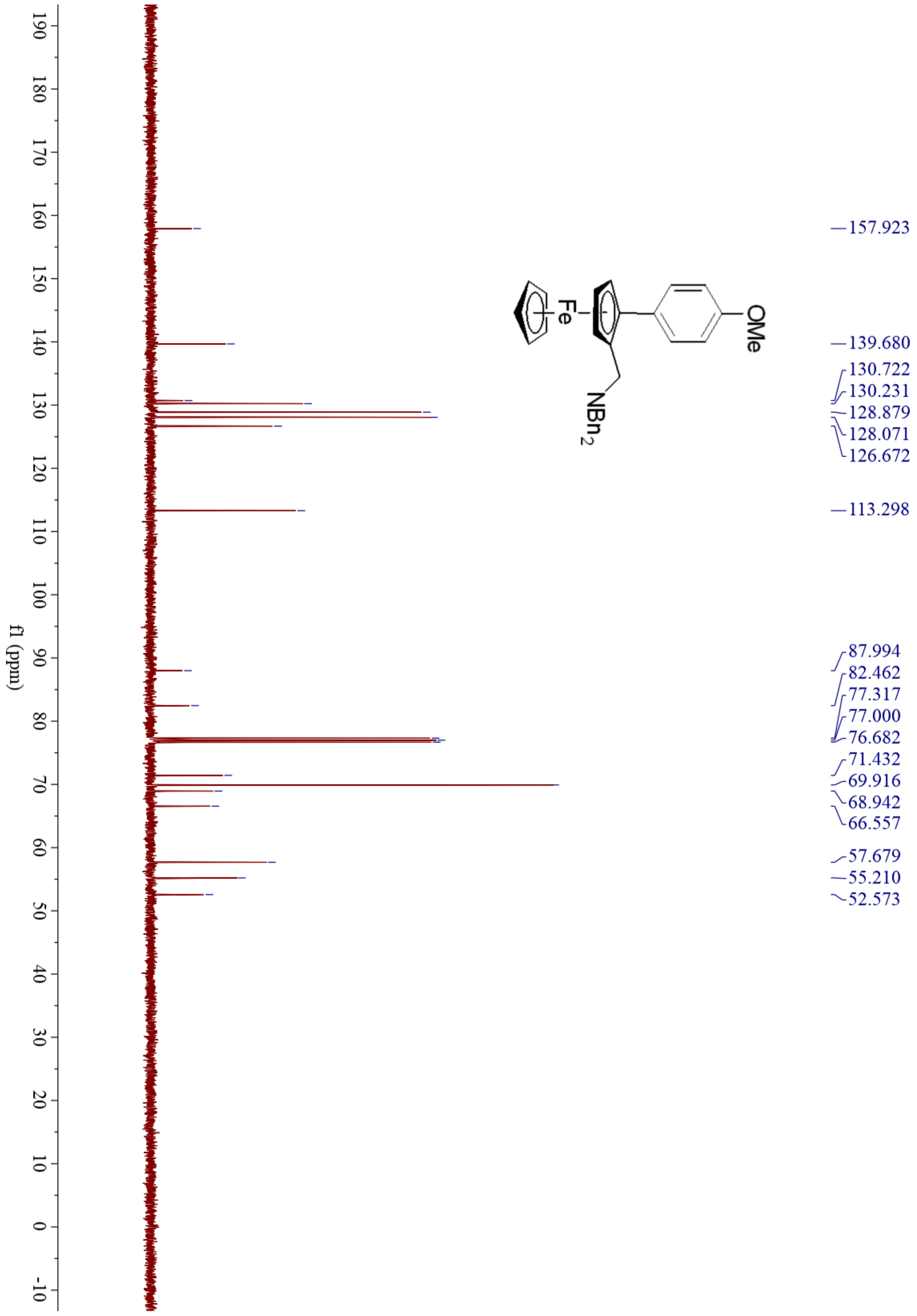

HPLC analysis of 4g

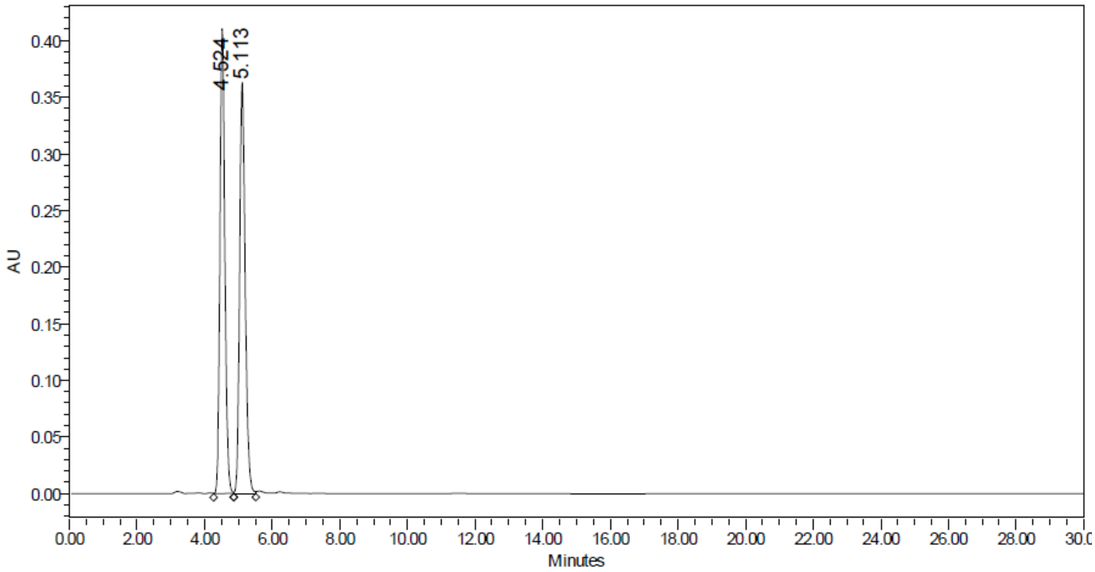

	RT	Area	% Area	Height
1	4.524	4186113	49.99	412414
2	5.113	4187649	50.01	363061

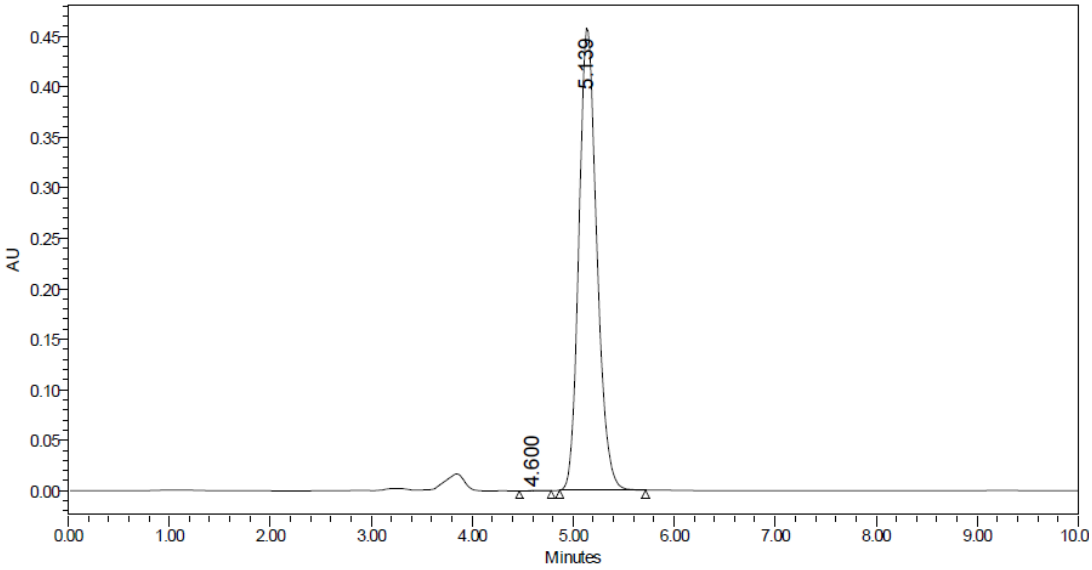

	RT	Area	% Area	Height
1	4.600	976	0.02	84
2	5.139	5643762	99.98	458581

^{1}H NMR spectra of 4h

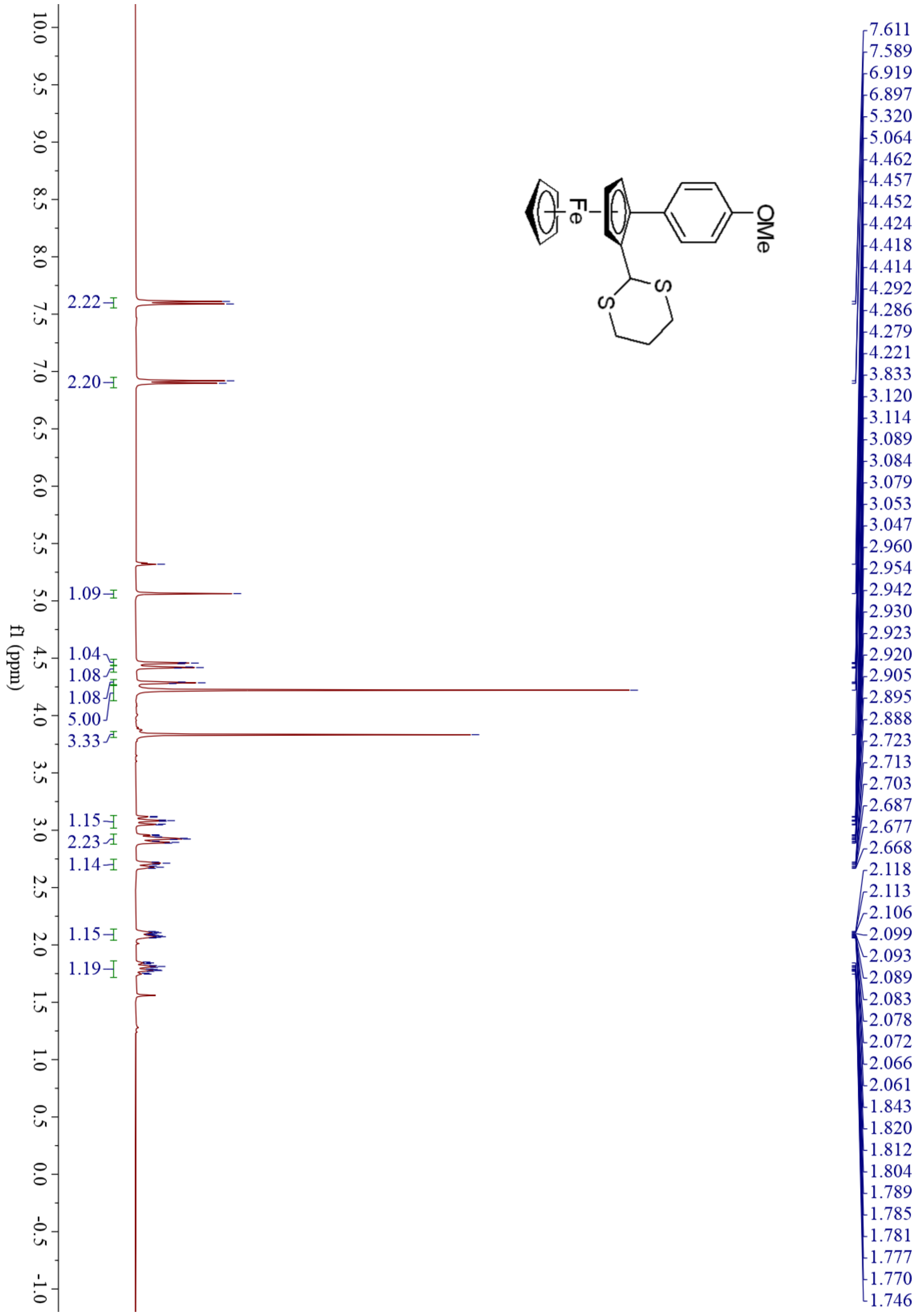

^{13}C NMR spectra of 4h

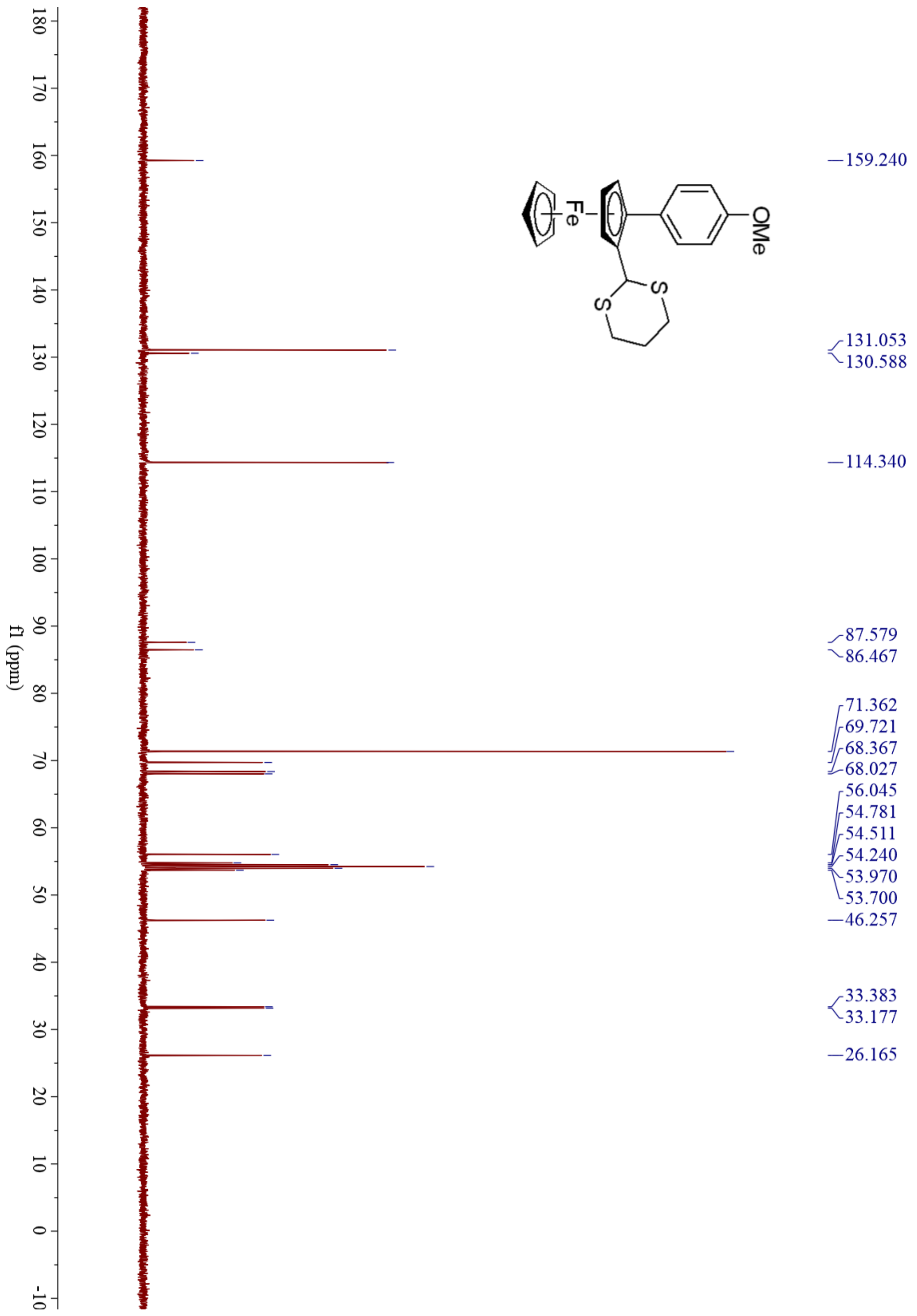

HPLC analysis of 4h

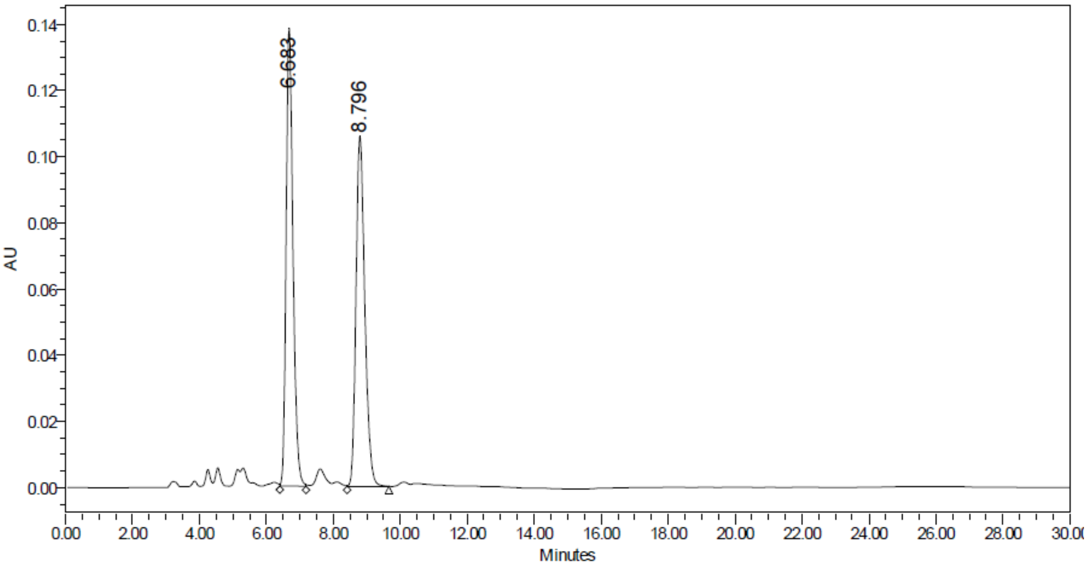

	RT	Area	% Area	Height
1	6.683	1960142	50.44	138347
2	8.796	1926264	49.56	105960

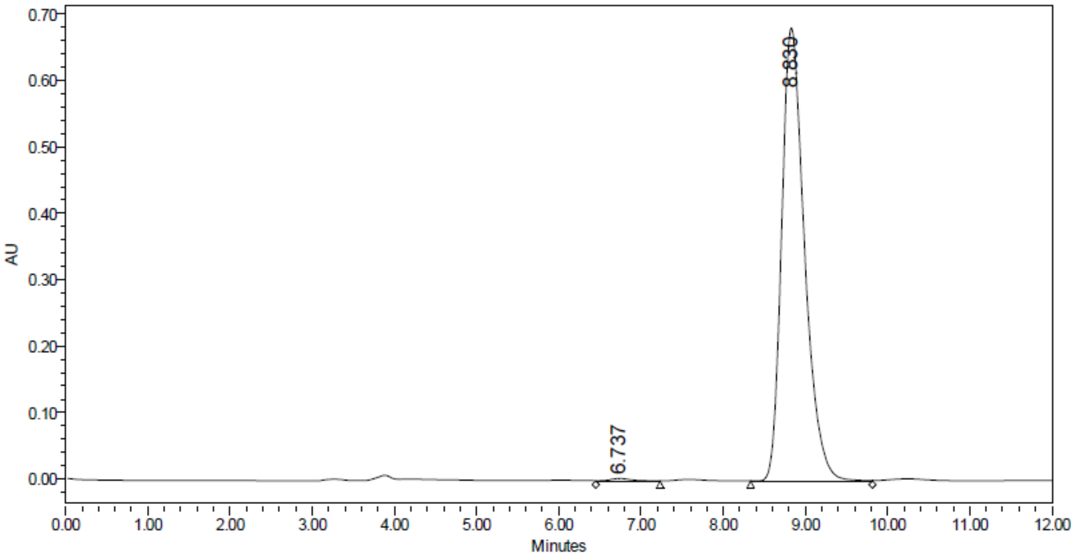

	RT	Area	% Area	Height
1	6.737	56554	0.43	3662
2	8.830	13189384	99.57	682329

^{1}H NMR spectra of 4i

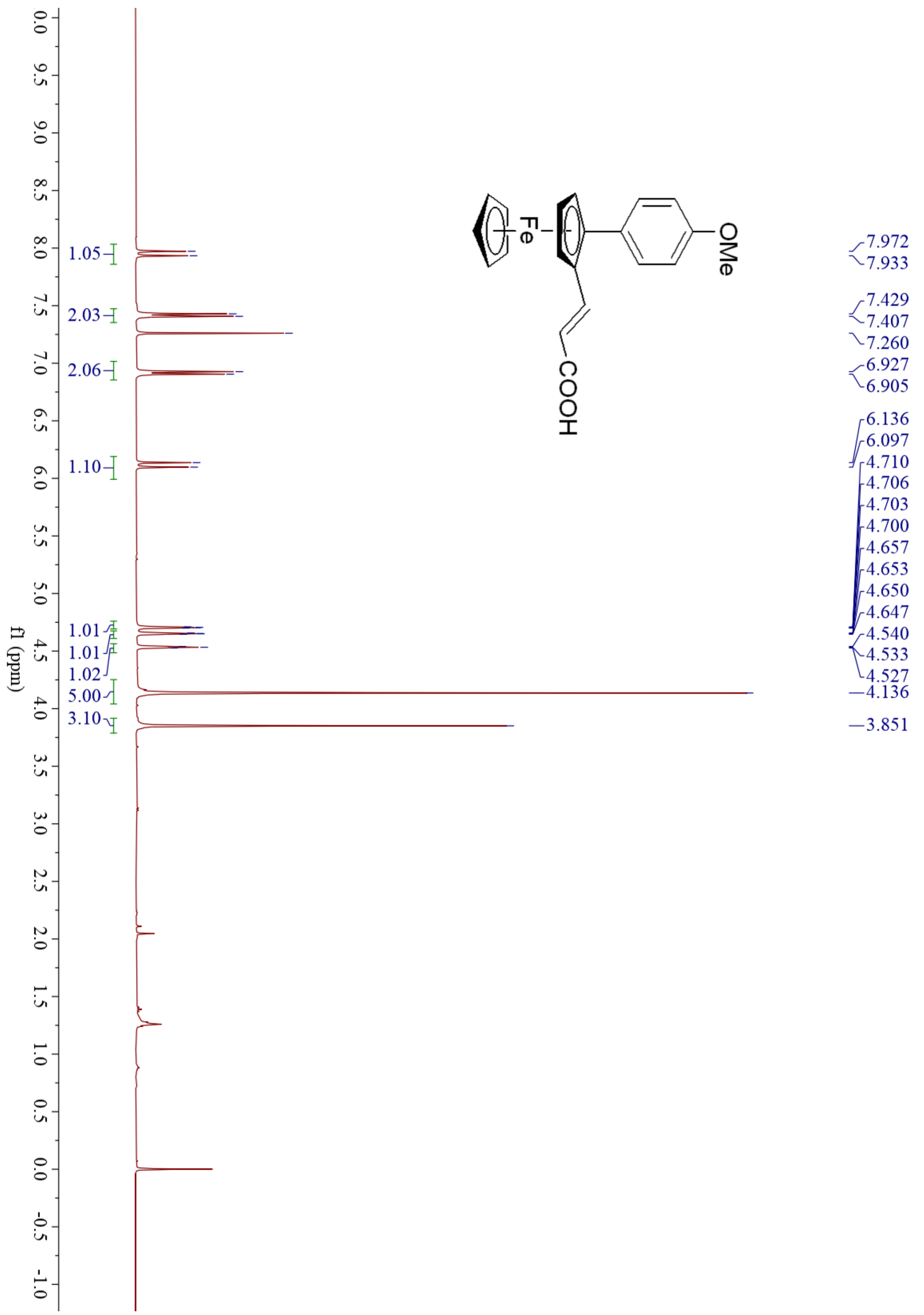

^{13}C NMR spectra of 4i

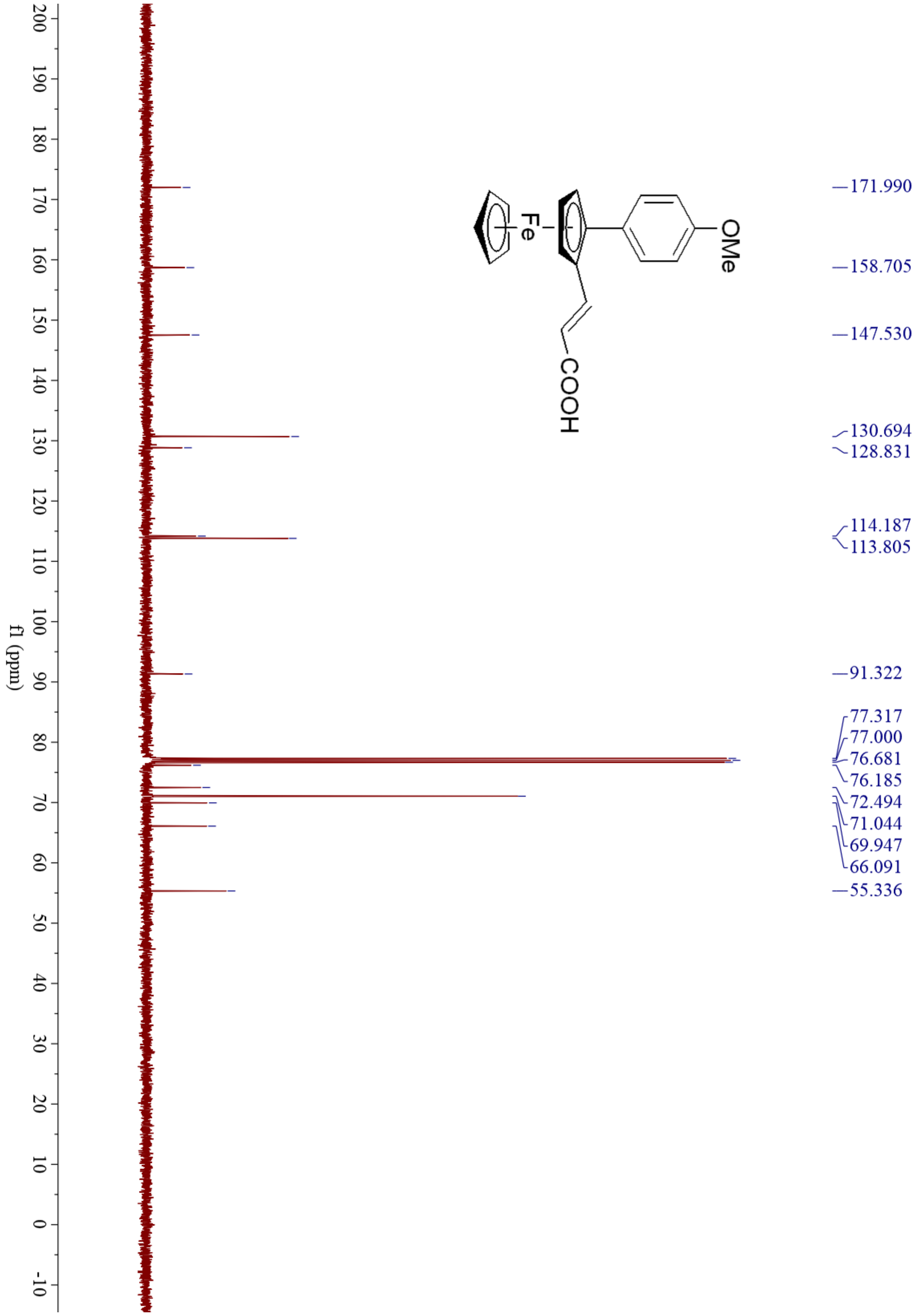

HPLC analysis of 4i

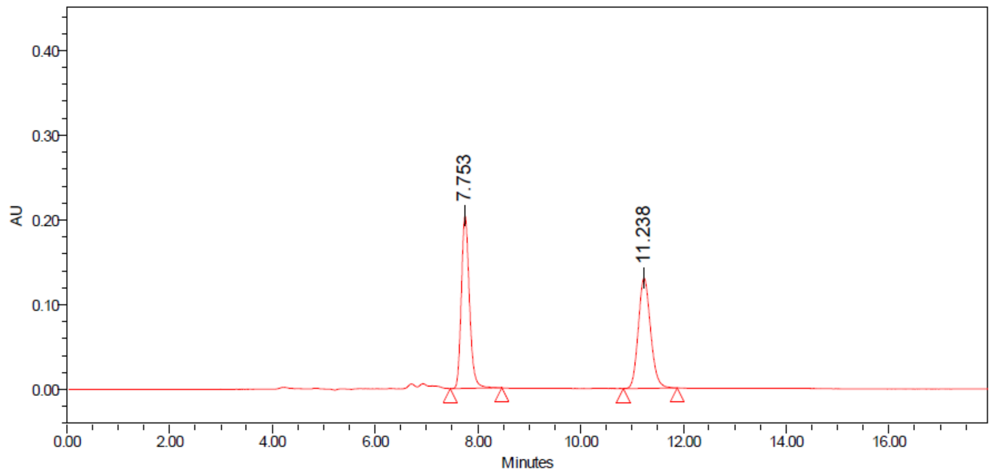

Peak Results

	SampleName	RT	Width (sec)	Height	Area	% Area
1	lcx-23-COOH-RAC	7.753	60.200	203823	2221218	50.64
2	lcx-23-COOH-RAC	11.238	62.700	130119	2165016	49.36

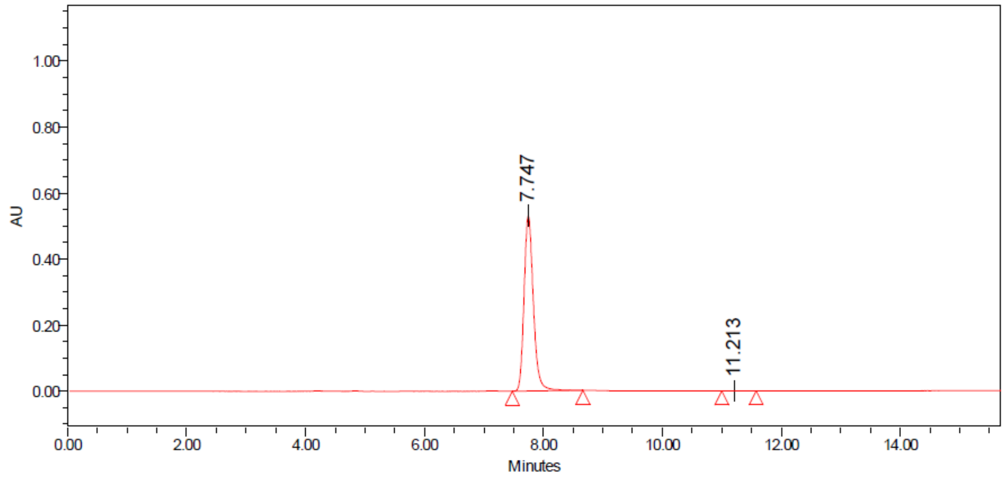

Peak Results

	SampleName	RT	Width (sec)	Height	Area	% Area
1	lcx-23-COOH-CHIRAL	7.747	71.400	530543	5746867	99.91
2	lcx-23-COOH-CHIRAL	11.213	34.500	280	5271	0.09

^{1}H NMR spectra of 5

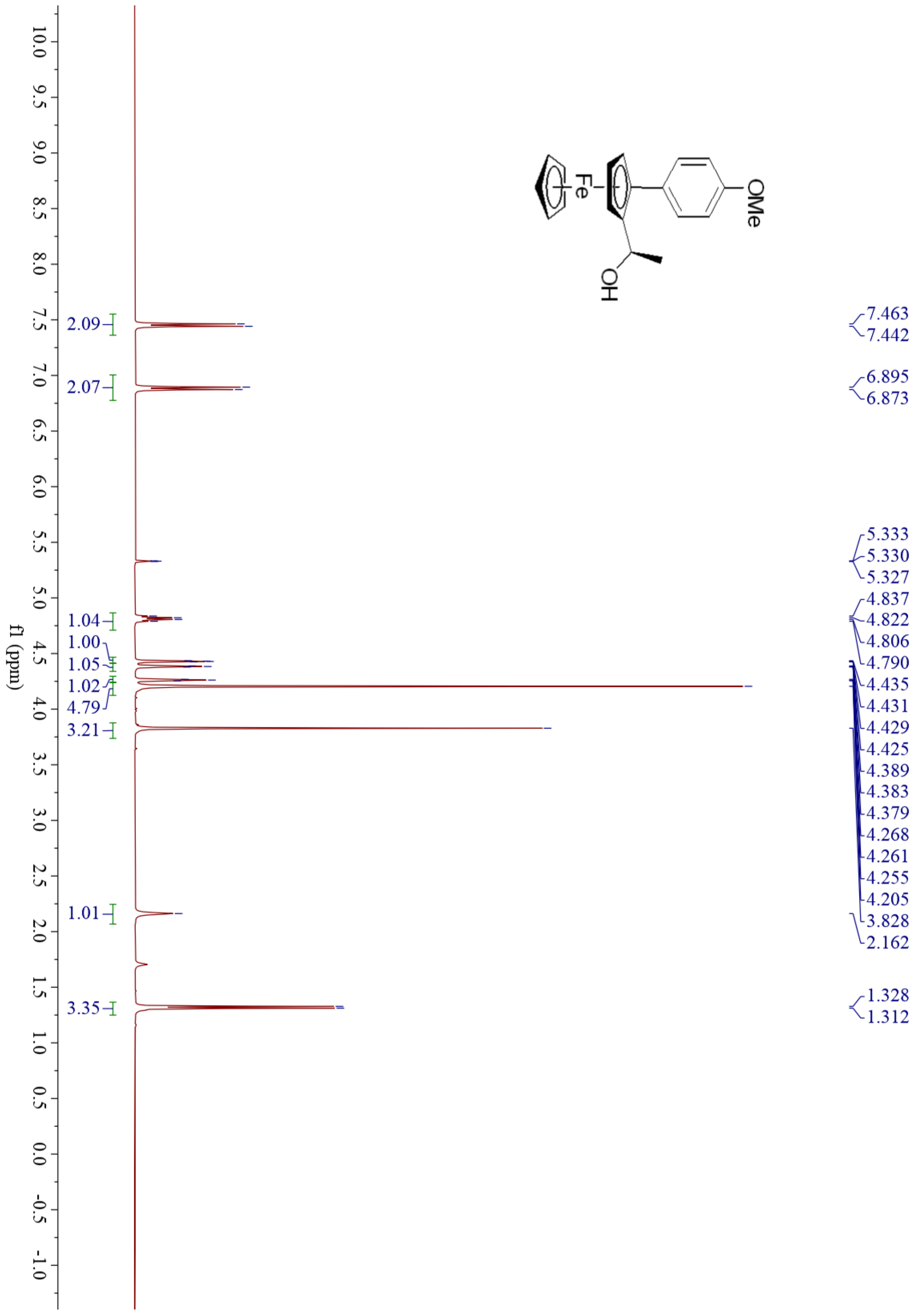

^{13}C NMR spectra of 5

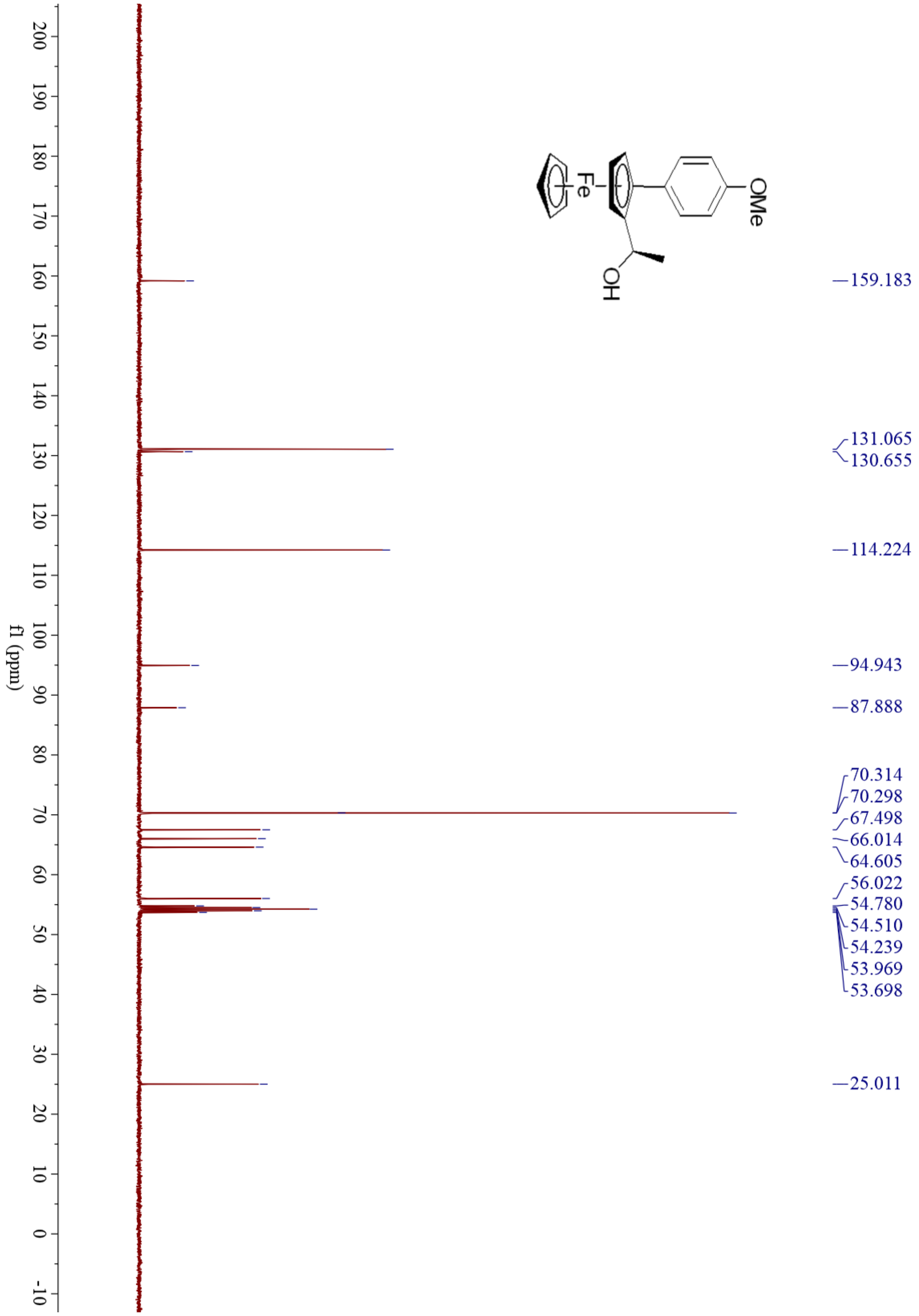

^{1}H NMR spectra of 6

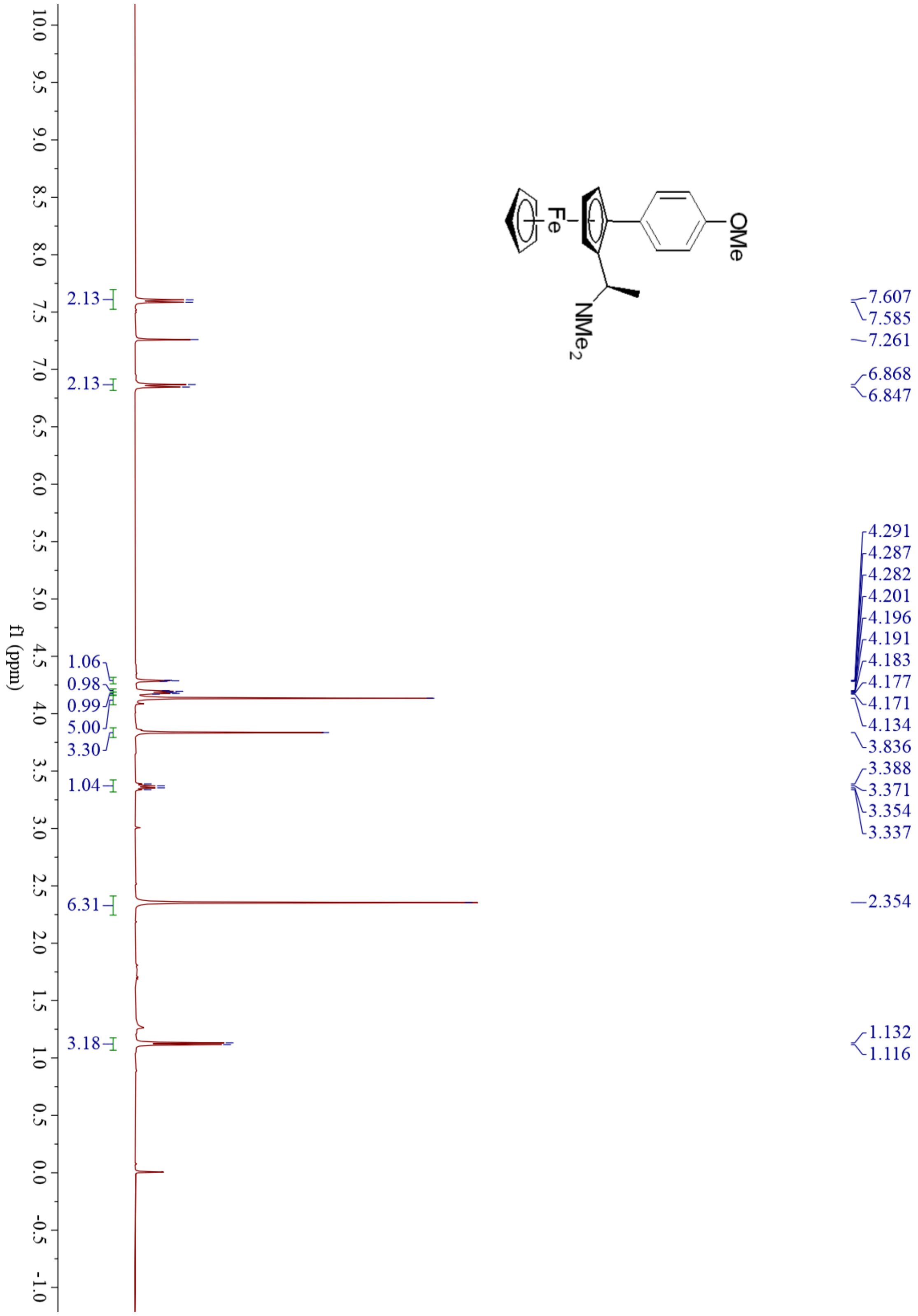

^{13}C NMR spectra of 6

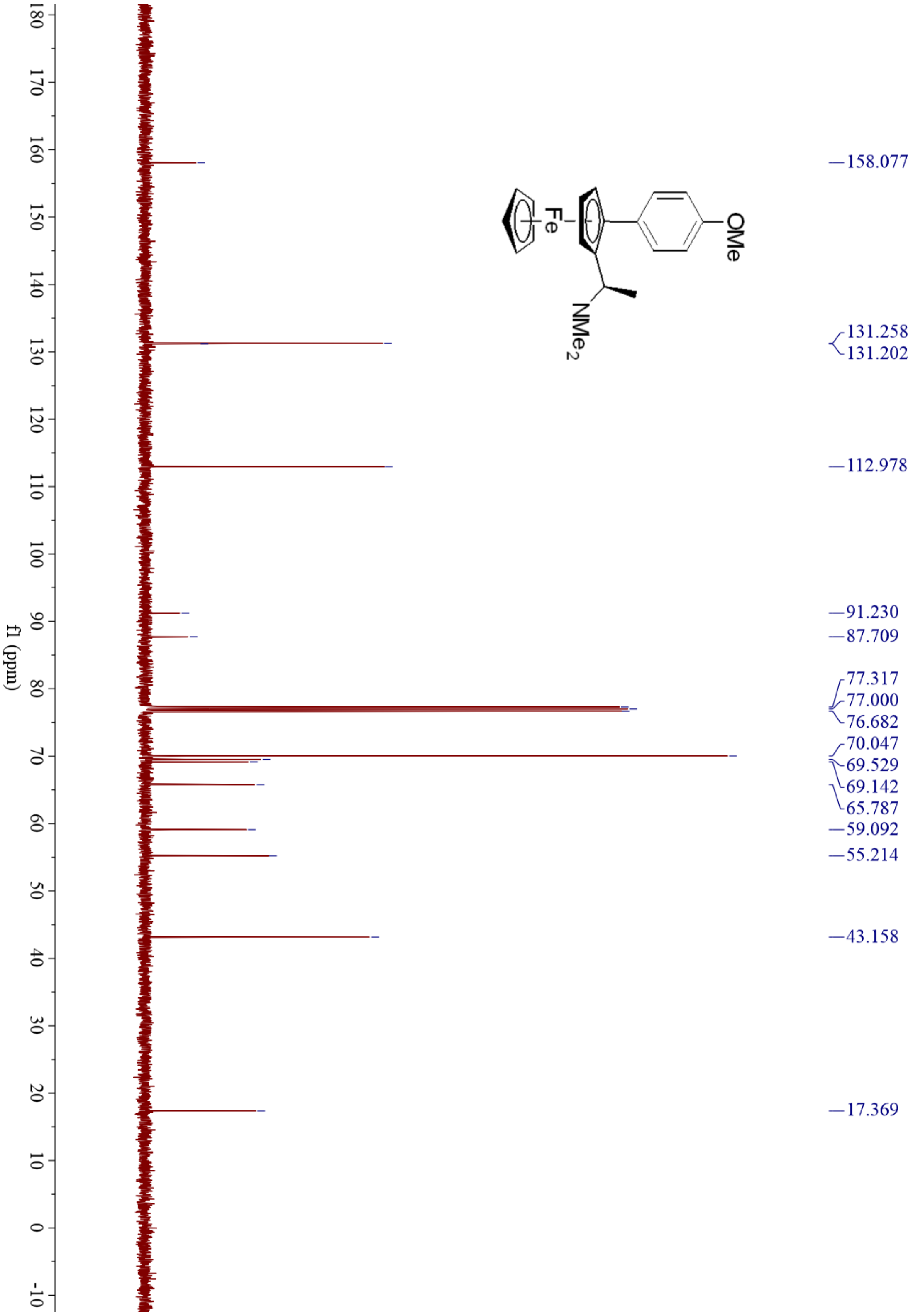

^{1}H NMR spectra of 7

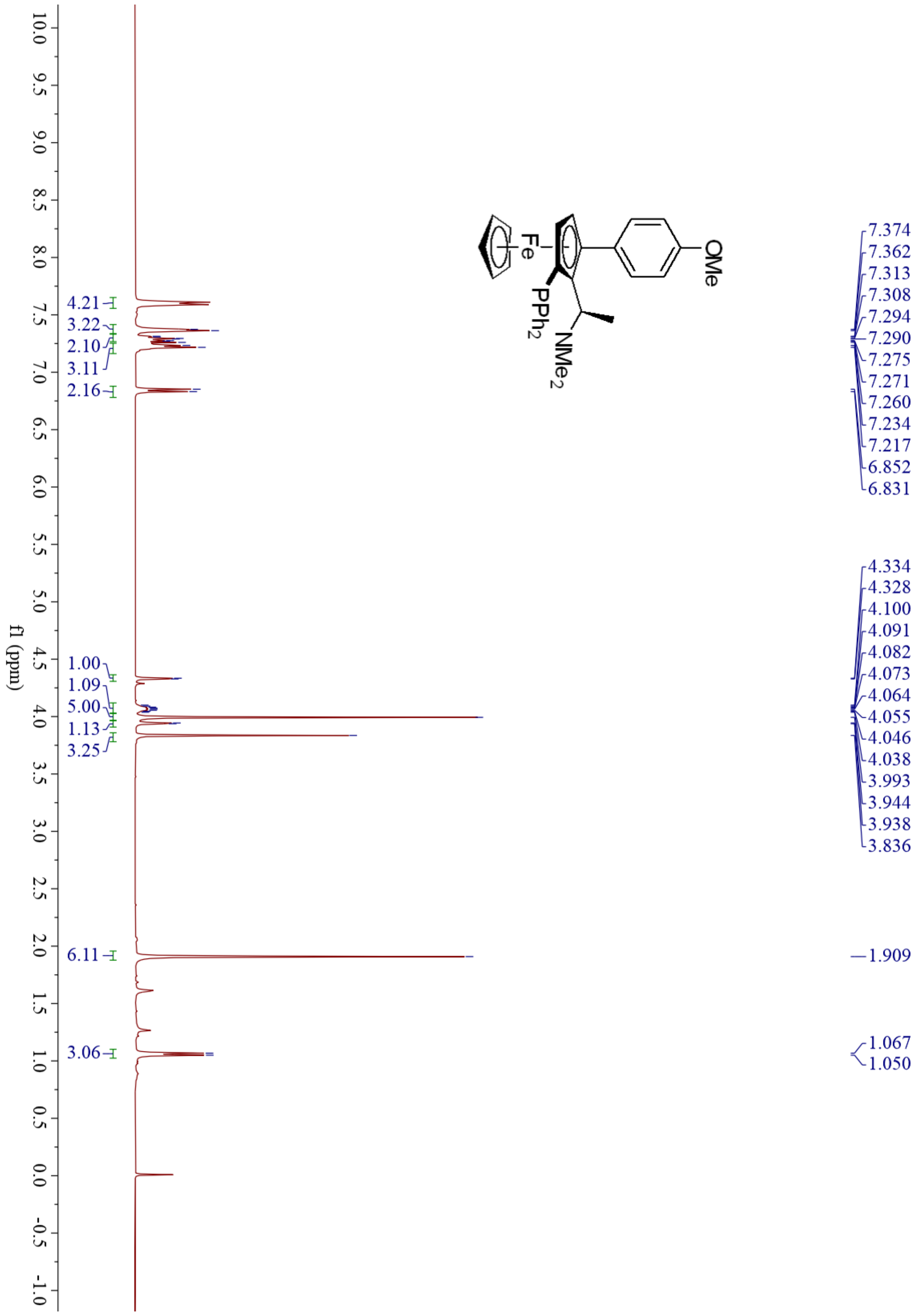

^{13}C NMR spectra of 7

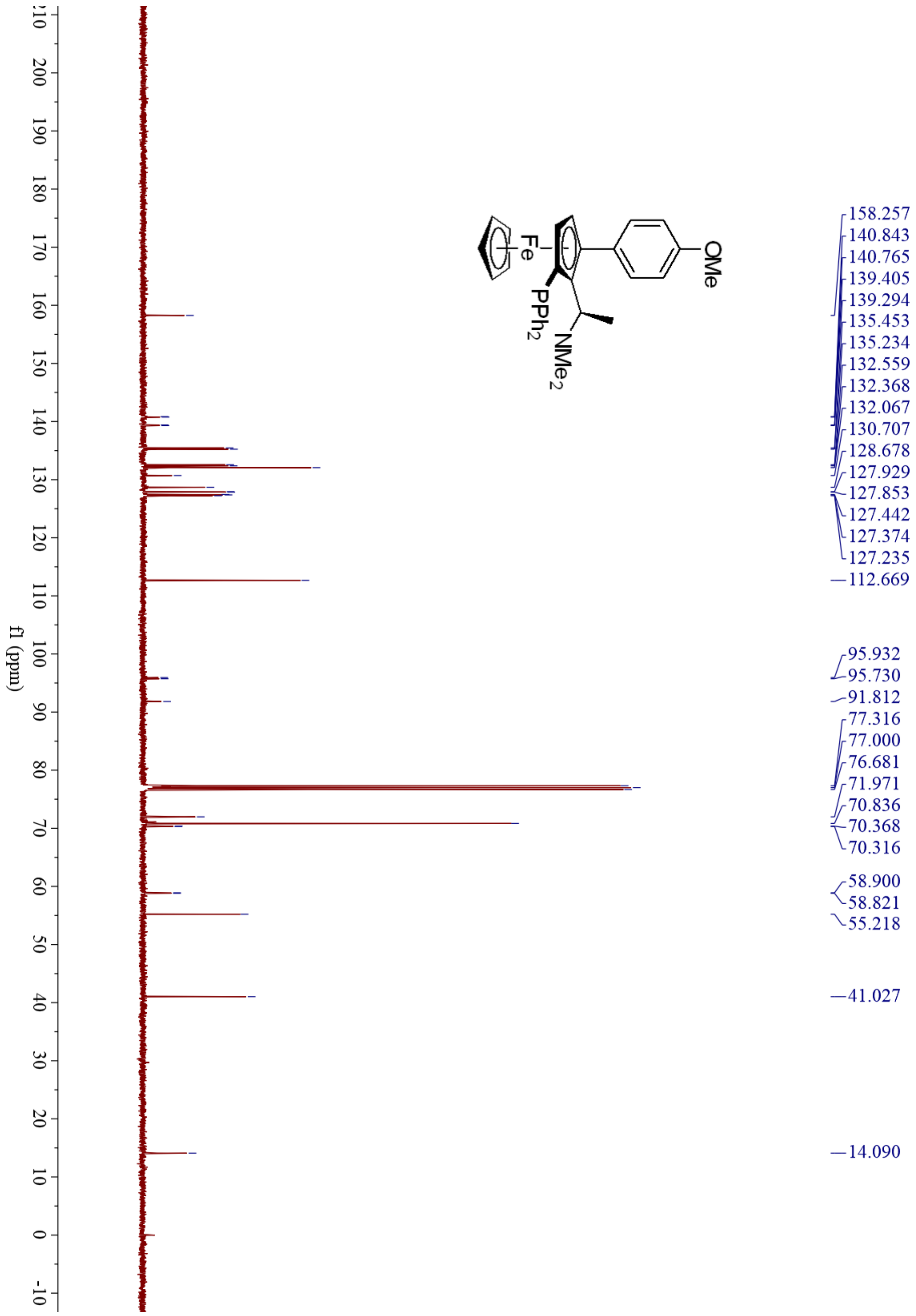

^{31}P NMR spectra of 7

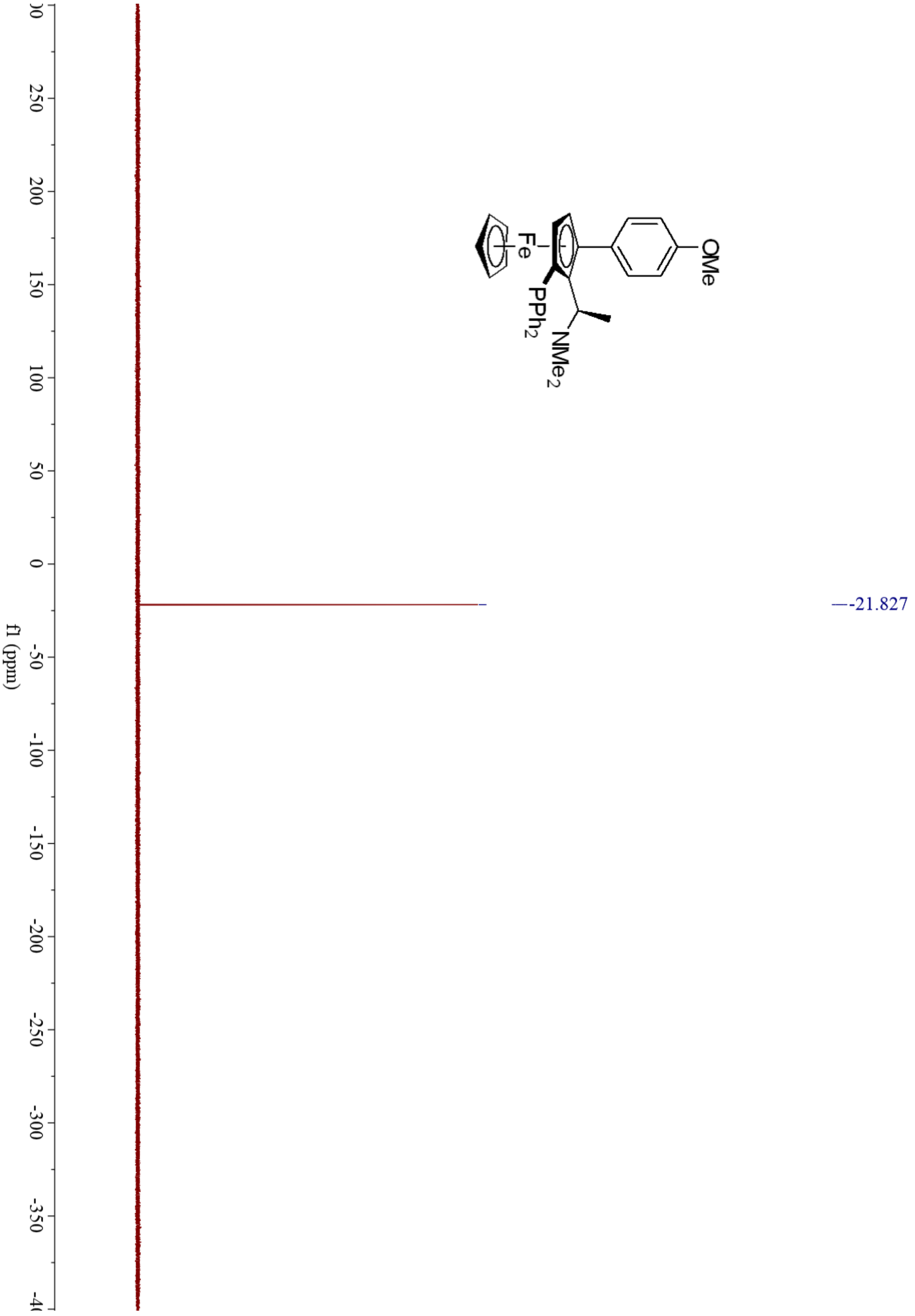

^{1}H NMR spectra of 8

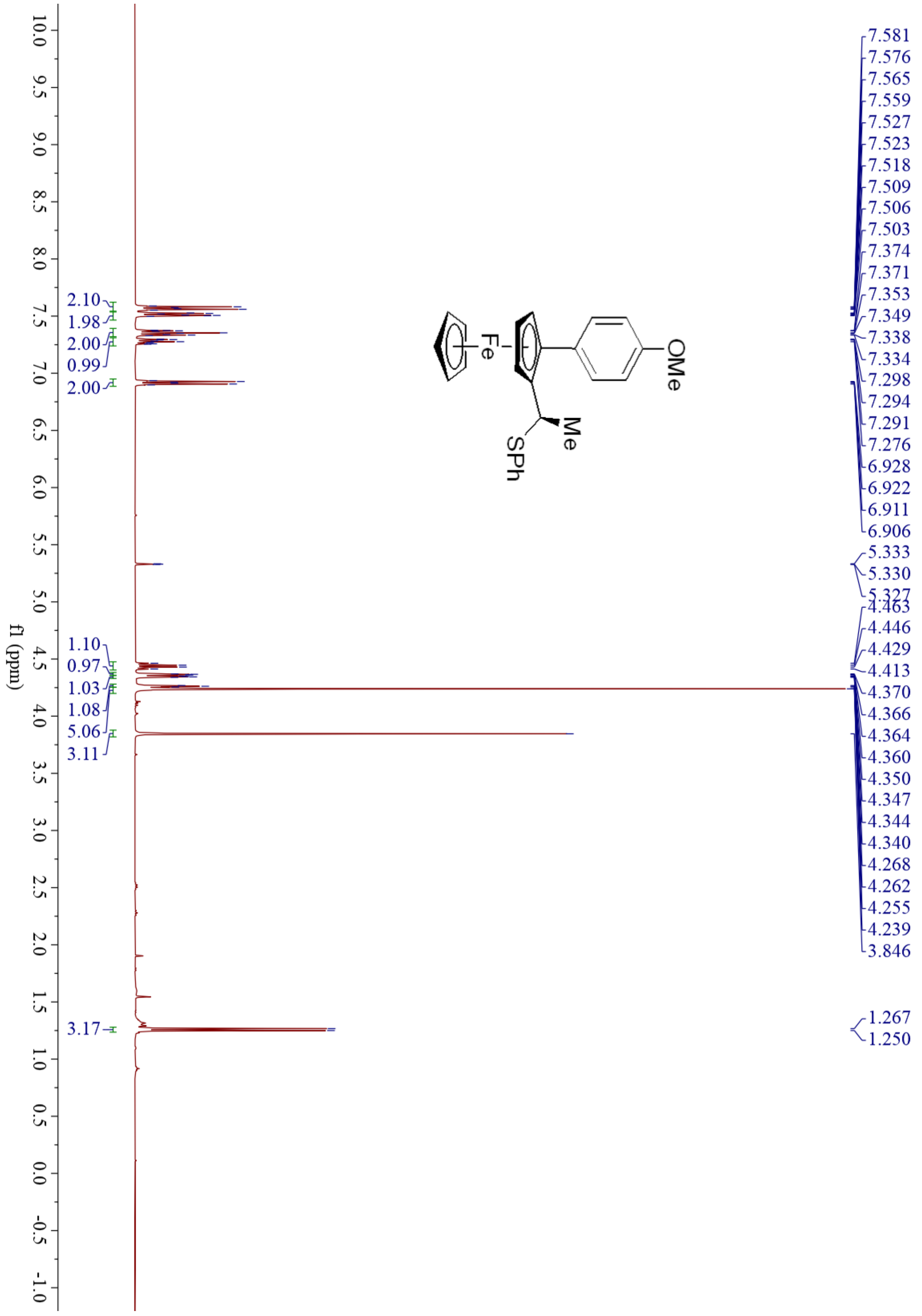

^{13}C NMR spectra of 8

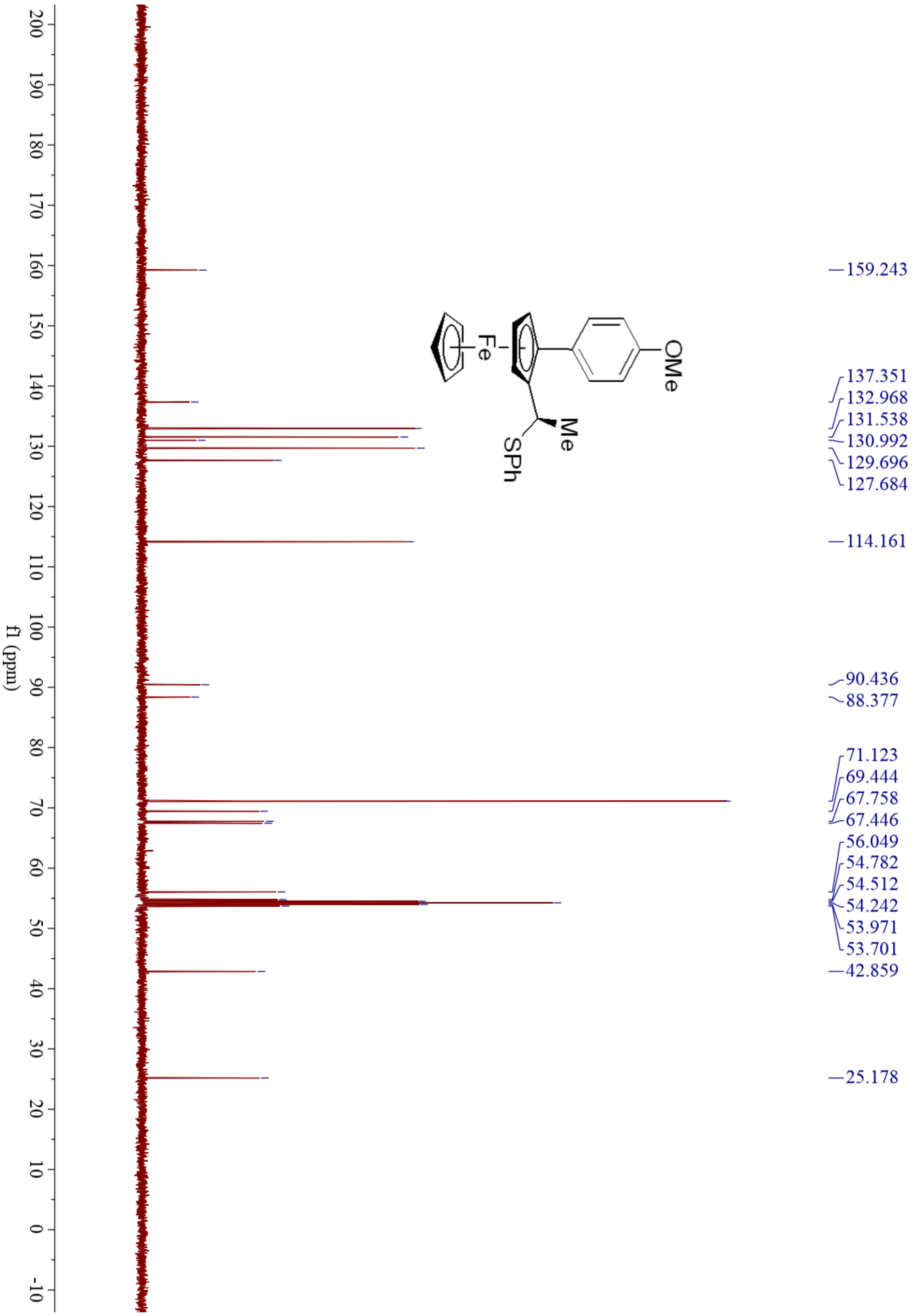

^{1}H NMR spectra of 9

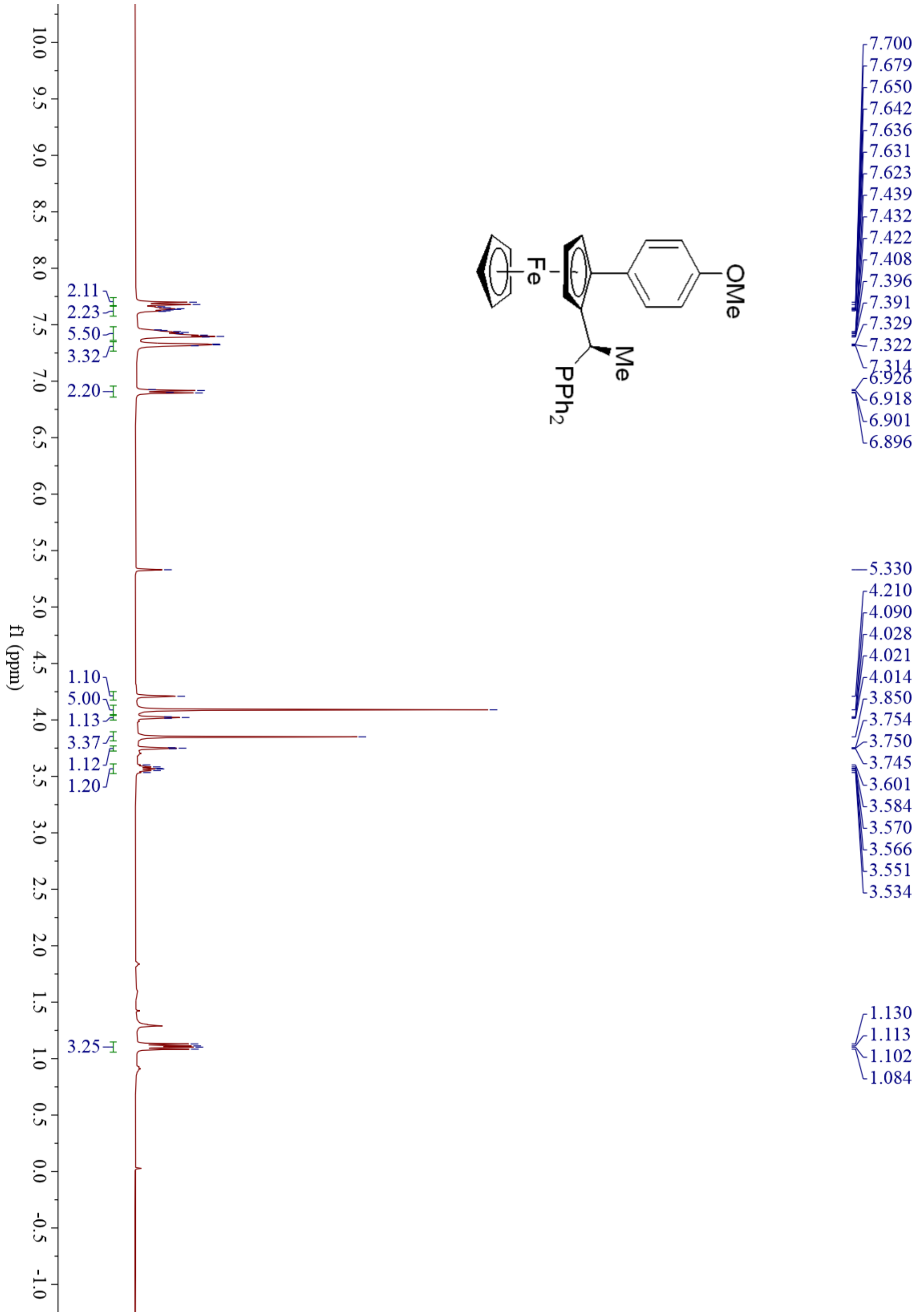

^{13}C NMR spectra of 9

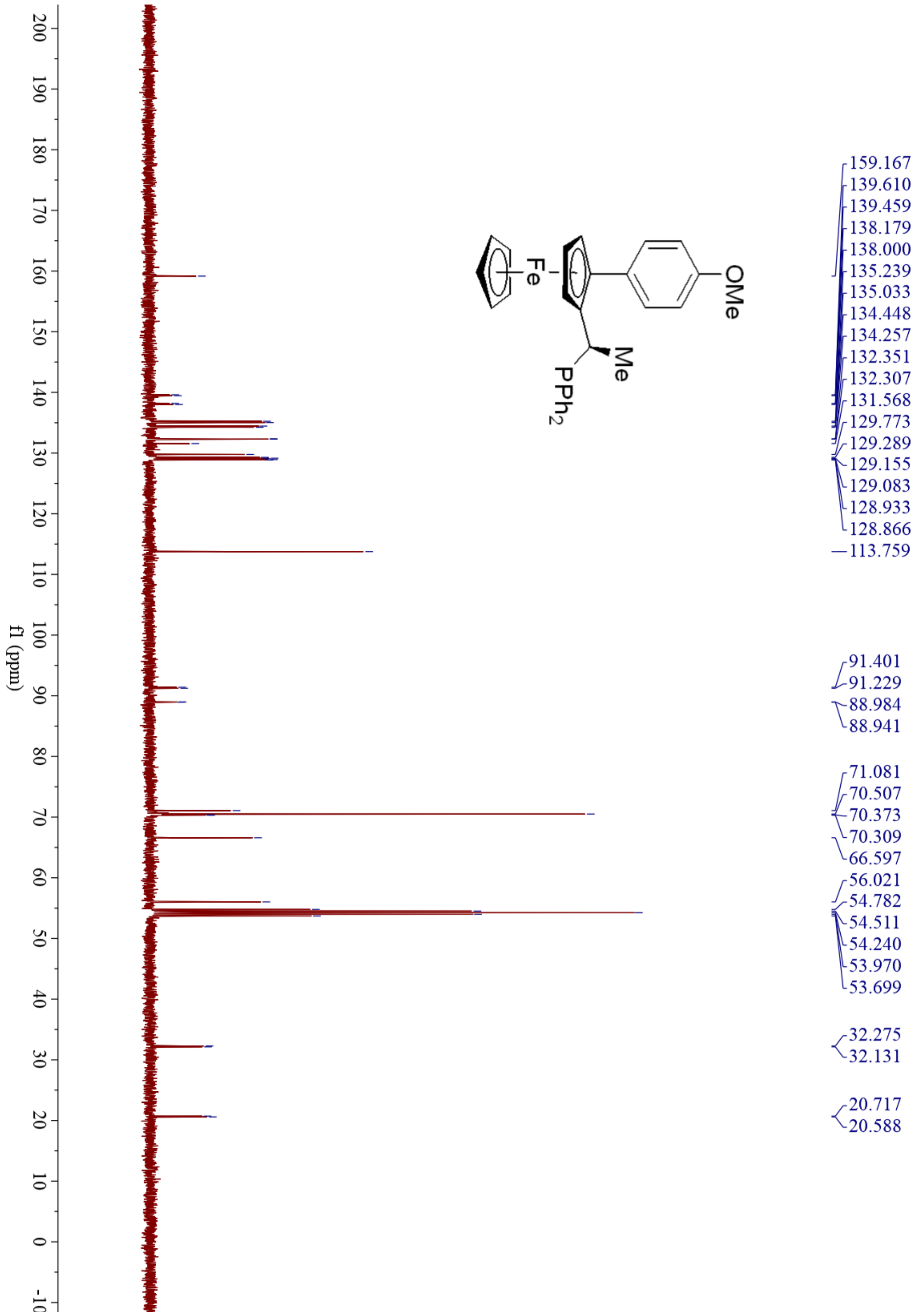

^{31}P NMR spectra of 9

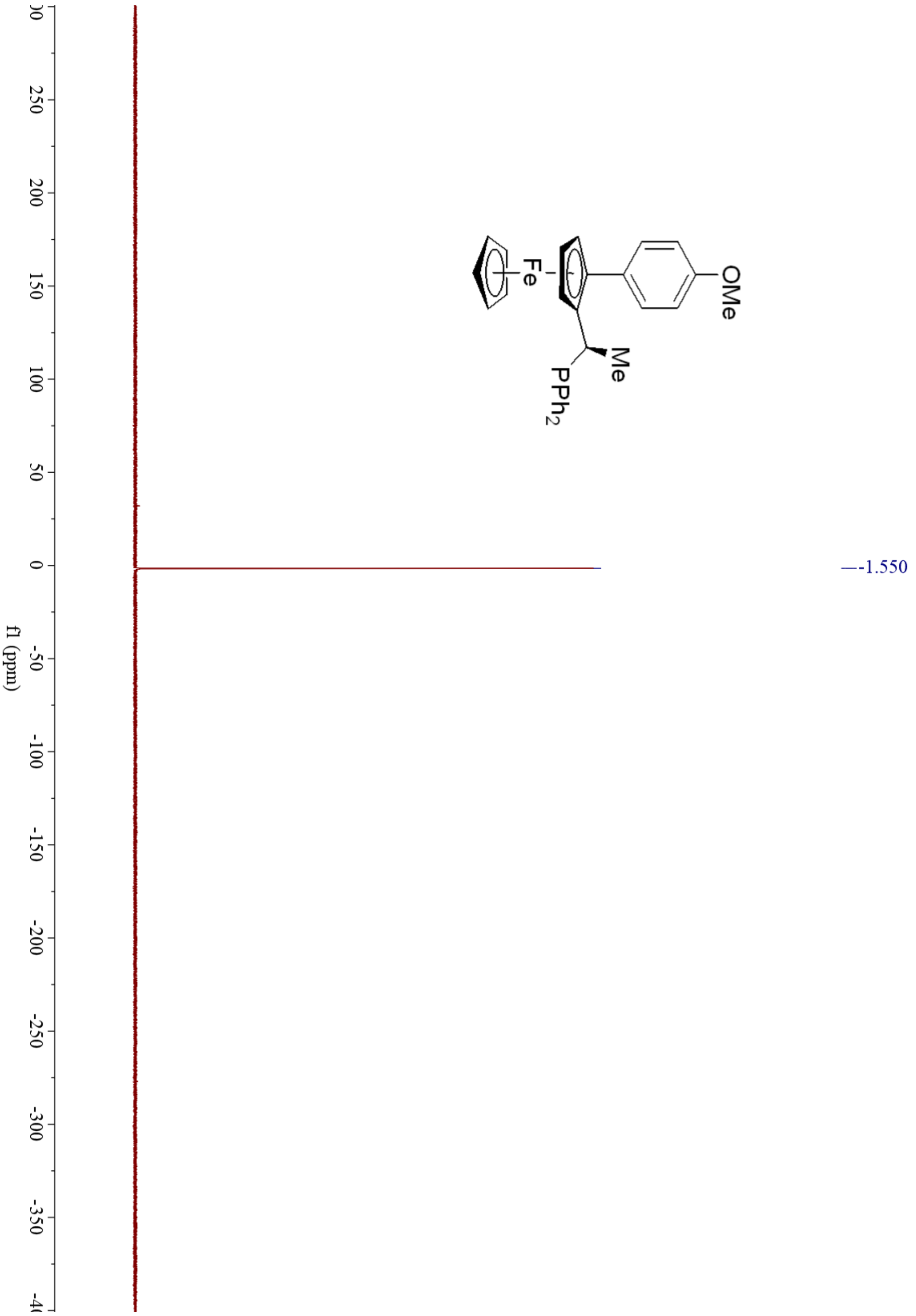

^{1}H NMR spectra of 12

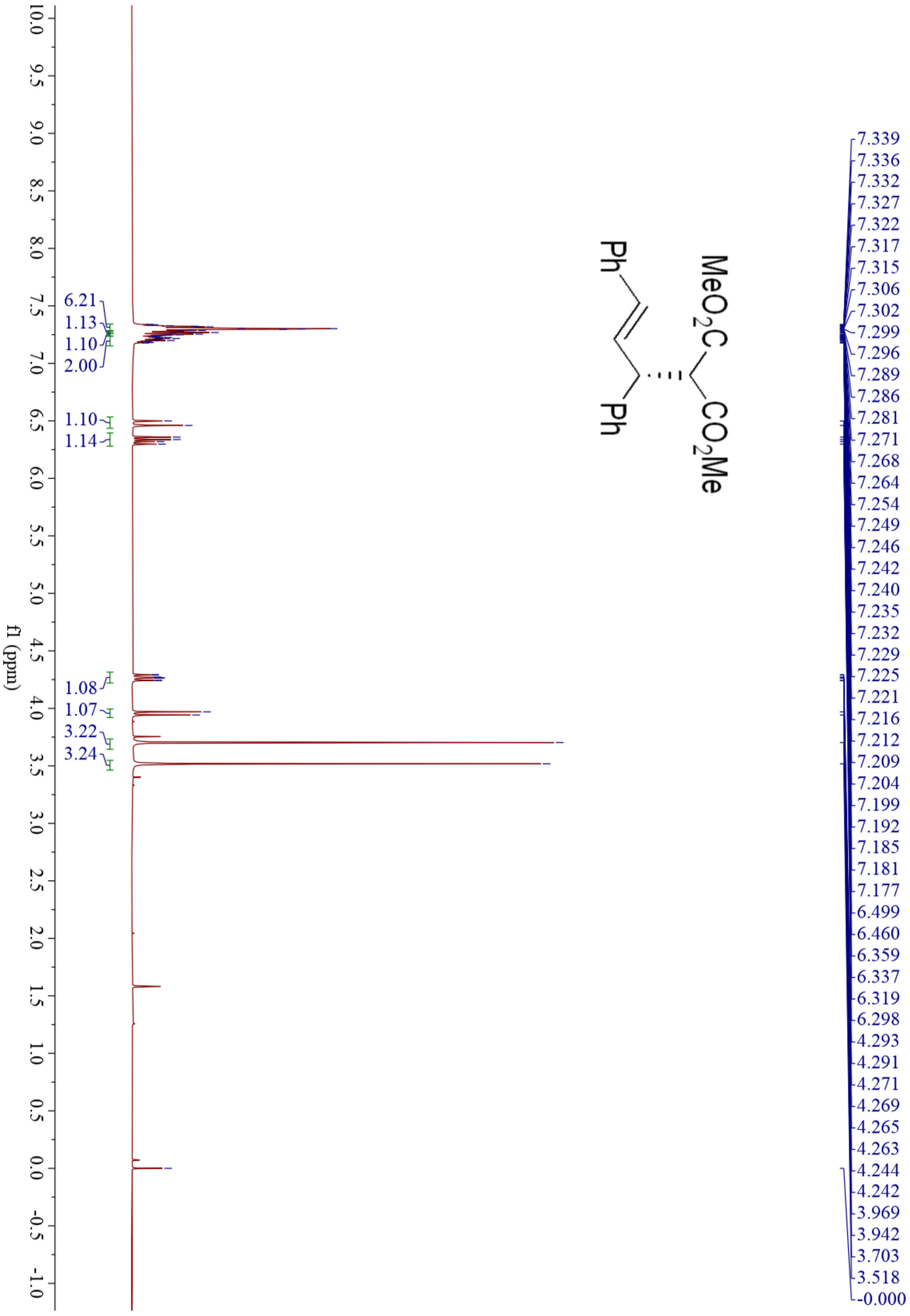

HPLC analysis of 12

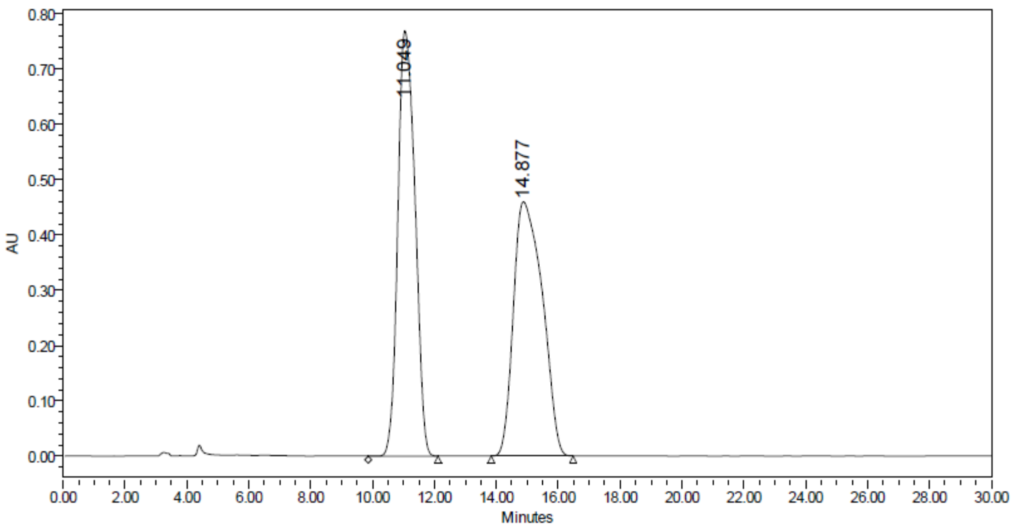

	RT	Area	% Area	Height
1	11.049	29743343	49.91	769250
2	14.877	29852690	50.09	460143

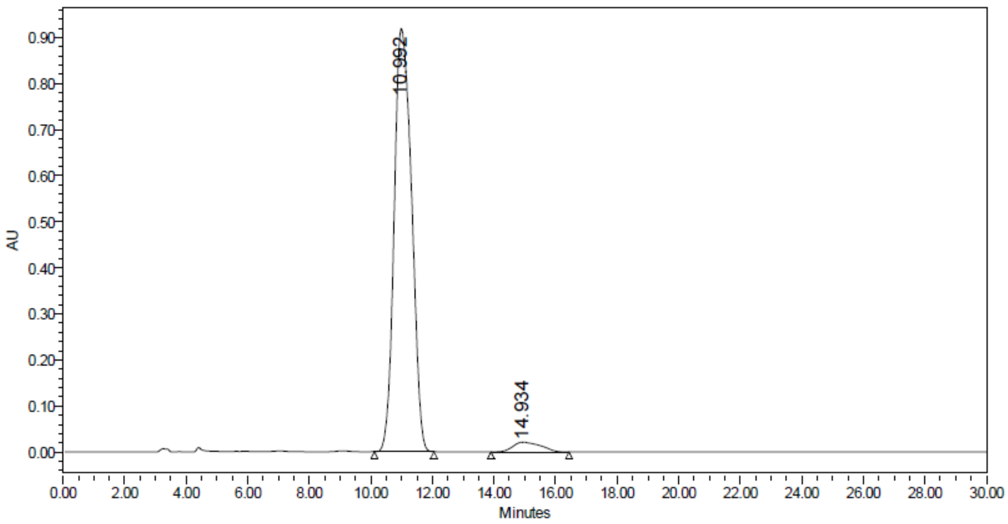

	RT	Area	% Area	Height
1	10.992	36191845	96.44	919249
2	14.934	1336834	3.56	21242
