# Supplementary figures and images for: Enantioselective Rh(I)-Catalyzed C–H Arylation of Ferroceneformaldehydes
Source: ACS Cent Sci. 2023 Sep 28;9(11):2036–43. doi: 10.1021/acscentsci.3c00748 (PMC10683487; doi:10.1021/acscentsci.3c00748)

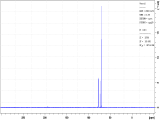

Supplement: Supplementary file 3 — oc3c00748_si_003.zip [file oc3c00748_si_003.zip › NMR fid files/1d/1d-C/1/pdata/1/thumb.png]

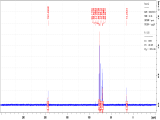

Supplement: Supplementary file 3 — oc3c00748_si_003.zip [file oc3c00748_si_003.zip › NMR fid files/1f/1f-C/1/pdata/1/thumb.png]

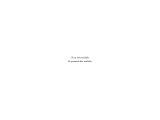

Supplement: Supplementary file 3 — oc3c00748_si_003.zip [file oc3c00748_si_003.zip › NMR fid files/1g/1g-C/1/pdata/1/thumb.png]
